# The miR-34a-5p promotes the multi-chemoresistance of osteosarcoma via repression of the AGTR1 gene

**DOI:** 10.1186/s12885-016-3002-x

**Published:** 2017-01-10

**Authors:** Youguang Pu, Fangfang Zhao, Yinpeng Li, Mingda Cui, Haiyan Wang, Xianghui Meng, Shanbao Cai

**Affiliations:** 1 Cancer Epigenetics Program, Anhui Cancer Hospital, West Branch of Anhui Provincial Hospital, Anhui Medical University, Hefei, 230031 Anhui China; 2 Xinxiang Medical University, Xinxiang, Henan 453000 China; 3 Department of Clinical Geriatrics, Anhui Provincial Hospital, Anhui Medical University, Hefei, 230031 Anhui China; 4 Department of Orthopedic Surgery, Anhui Cancer Hospital, West Branch of Anhui Provincial Hospital, Anhui Medical University, Hefei, 230031 Anhui China

**Keywords:** miR-34a-5p, AGTR1, Osteosarcoma, Multi-chemoresistance

## Abstract

**Background:**

Chemoresistance hinders the curative cancer chemotherapy. MicroRNAs (miRNAs) are key players in diverse biological processes including the chemoresistance of cancers.

**Methods:**

A RNA-seq-based miR-omic analysis of osteosarcoma (OS) cells was performed to detect the levels of miR-34a-5p. Bioinformatics analysis revealed that AGTR1 is one of the target genes of miR-34a-5p. The mRNA and protein levels of AGTR1 were detected in both the miR-34a-5p-mimic transfected G-292 and miR-34a-5p-antagomiR transfected SJSA-1 cells. The involvement of AGTR1 with OS chemoresistance was validated by the experiments with siRNA-mediated repression or overexpression of the AGTR1 gene.

**Results:**

We showed that miR-34a-5p promotes the multi- chemoresistance of OS. The angiotensin II type 1 receptor (AGTR1) gene, is one of the targets of miR-34a-5p in OS and thus negatively correlates with OS chemoresistance by systematic investigations of a multi-drug sensitive (G-292) and resistant (SJSA-1) OS cell lines. Down-regulation of the AGTR1 expression by siRNA passivates G-292 cells and suppresses cell apoptosis, while over-expression of AGTR1 sensitizes SJSA-1 cells and thus promotes the drug-triggered cell death.

**Conclusions:**

The miR-34a-5p and its target gene AGTR1 are the potential targets for an effective chemotherapy of OS. Our results also provide novel insights into the effective chemotherapy for OS patients.

**Electronic supplementary material:**

The online version of this article (doi:10.1186/s12885-016-3002-x) contains supplementary material, which is available to authorized users.

## Background

MiRNAs are a large group of small non-coding RNAs that play vital roles in various biological processes [1]. MiRNAs regulate the expression of a variety of target genes and their dysregulation is closely related to the development of diseases including cancer. The abnormal expression of miRNAs in cancer contributes to almost every field of tumor pathology [2, 3], including drug resistance [4], which remains a major obstacle to effective therapy of patients [5]. The multi-chemoresistance property differs dramatically among the cancer patients, even in the different cancer lesions of a single patient [6]. Despite of intensive efforts, our knowledge of the multi-chemoresistance of cancers remains very poor due to the diverse mechanisms that induce the multi-chemoresistance [7, 8]. To date, the emerging studies have been focused on the role of miRNAs in the occurrence of chemoresistance in different cancers. The prominent examples for bladder cancer chemoresistance are miR-181, miR-199a-5p, miR-30d [9] and miR-193a-3p [5, 10]. In hepatocellular carcinoma (HCC) cells, miR-193a-3p contributes to the 5-FU resistance regulated by the DNA methylation in particular *via* repressing SRSF2 expression [10]. In addition, overexpressed miR-21 in colorectal cancer tissues contributes to the resistance to 5-FU [11]. The expression of miR-130a is higher in SKOV3/DDP, and suppression of miR-130a could conquer the cisplatin resistance by targeting the MDR1/P-gp pathway [12]. The miR-140 participates in the drug resistance to osteosarcoma (OS) xenografts by decreased cell proliferation via G- and G2-phase arrest [13].

The miR-34 family members are down-regulated in a variety of cancers and the expression of miR-34 is directly regulated by the transcription factor p53 [14–16]. Moreover, miR-34a negatively regulates the Delta-like ligand 1 (DLL1) of the Notch pathway and thus down-regulates cell proliferation by inducing apoptosis and neural differentiation in medulloblastoma cells. In gliomas, miR-34a down-regulates c-Met and CDK6, suggesting that miR-34a provides a therapeutic biomarker for brain tumors [17]. Furthermore, miR-34a-5p, derived from miR-34a, has been found to prevent cell migration and invasion [18–21], which indicated that miR-34a-5p might involve in inhibiting tumor development.

OS is the most common malignant primary bone tumor which is frequently occurred in children and adolescents [22, 23], and the mechanism for the OS chemoresistance remains limited. In the present study, we set up a RNA-seq assay and identified several differentially expressed genes in a multi-chemosensitive (G-292) versus a resistant (SJSA-1) OS cell lines. We showed that miR-34a-5p promotes the OS multi-chemoresistance *via* its repression of the AGTR1 gene, a new target of miR-34a-5p.

## Methods

### Cell lines and culture

The two cell lines (SJSA-1 (ATCC NO. CRL-2098) [24] and G-292 (ATCC NO. CRL-1423) [25] used in this study) were purchased from ATCC. The cells were cultured in Dulbecco’s modified Eagle’s medium (Invitrogen, Carlsbad, CA, USA) implemented with 10% fetal bovine serum and 1% glutamine at 37 °C in 5% CO_2_.

### RNA-seq analysis

RNA-seq analysis was performed by BGI-Tech (Shenzhen, China). Sample preparation and data analysis were done as reported previously [26].

### The transient transfection assays

All the sequences including the antagomiR, mimic, siRNA, the scramble sequence (negative control, NC) were supplied by Guangzhou Ribobio, China. The expression constructs for AGTR1 (EX-A0417-M98-5) fused with a GFP tag were supplied by Guangzhou Fulengen (Guangzhou, China). The transfection method mentioned above was performed according to the manufacturer’s instruction. The partial sequences used in this study are as follows:

- si-ATGR1:
  - 5' CUGUAGAAUUGCAGAUAUU dTdT 3'
  - 3' dTdT GACAUCUUAACGUCUAUAA 5'
  - hsa-miR-34a-5p
  - antagomiR: 5'ACAACCAGCUAAGACACUGCCA 3'
- mimics:
  - sense 5'UGGCAGUGUCUUAGCUGGUUGU 3'
  - antisense 5'ACAACCAGCUAAGACACUGCCA 3'

### Chemotherapeutics and drug resistance profiling (IC_50_ determination)

Clinical grades of the following drugs were used, Dox (Haizheng, Zhejiang, China); Etop (Hengrui, Jiangsu, China); Carb: carboplatin (Qilu, Shandong, China) and CDDP (Haosen, Jiangsu, China) [5, 27, 28]. The method of MTT assay has been described in our previous report [26].

### Apoptosis analysis

The annexin V-FITC/propidium iodide (PI) staining assay was used to detect the apoptosis of G-292 cells transfected with either 5PM, si-AGTR1 or their corresponding NC. Cells growing to the logarithmic growth phase were harvested and rinsed after washing with cold PBS. Then, FITC-labeled enhanced annexinV (3 μl) and propidium iodide (3 μl, 20 μg/ml) were added to the cell suspension (100 μl) for labeling (Vazyme, China). After incubation in the dark for 15 min at room temperature, the samples were diluted with 50 μl PBS. Apoptotic cells were then evaluated by gating PI and Annexin V-positive cells on a FACSCalibur instrument. The results were analyzed according to the manufacturer’s instructions. The experiments were performed at least three times independently, and a representative is shown.

### Luciferase reporter assay

A luciferase reporter assay was performed to test the binding of miR-34a-5p to AGTR1. The detailed methods were described previously [29]. The full-length AGTR1 3’-untranslated region (UTR, 894 bp) containing the target sequence of miR-34a-5p was inserted into the pGL3 -reporter plasmid to construct pGL3-luc-AGTR1 WT and pGL3-luc-AGTR1 Mut. Cells were seeded into 96-well plates at approximately 1x10^4^ cells per well. Then the cells were transfected with a mixture of pGL3-luc-AGTR1 WT or Mut (50 ng), Renilla (5 ng), mimic or NC nucleotides (5 pmol) using the riboFECT CP transfection kit according to the manufacturer’s instruction. After transfection in twenty-four hours, the cells were assayed by the Dual-Luciferase Reporter Assay System (Promega) using a Promega GloMax 20/20 luminometer. The relative luciferase activities of the UTR construct and pathway reporter constructs were analyzed as reported previously [5].

### RNA analysis

The total RNA was extracted from the cells using Trizol (Tiangen, China) according to the manufacturer’s instructions. The mRNAs were analyzed as previously reported [29]. The sequences of primers and probes used for the qRT-PCR analysis are as follows:

- HAGTR1 F: 5′-TGCTTCAGCCAGCGTCAG-3′
- HAGTR1 R: 5′-GCGGGACTTCATTGGGTG-3′
- HAGTR1 probe: 5′-CY5-CTCACGTGTCTCAGCATTGATCGATAC-3′
- hACTB F: 5′-GCCCATCTACGAGGGGTATG-3′
- hACTB R: 5′-GAGGTAGTCAGTCAGGTCCCG-3′
- hACTB probe: 5′-HEX-CCCCCATGCCATCCTGCGTC-3′

To detect and quantify the expression of miR-34a-5p, Total RNA was reverse transcribed using a Bulge-Loop™ miRNA qRT-PCR Primer Set (Ribobio) and quantified by SYBR Green-based real-time PCR analysis. The Ct values of the target miRs were normalized to the Ct values of U6 RNA before quantification using the 2^−ΔΔ^ Ct method.

### Protein analysis

Cells were lysed with a lysis buffer [29]. Anti-AGTR1 (25343-1-AP) was purchased from San Ying Biotechnology, China. The target proteins were then detected with anti-rabbit IgG peroxidase-conjugated antibody (SA00001-2; San Ying Biotechnology, China). The target bands were detected by an enhanced chemiluminescence reaction (Pierce), and the relative density (level) of proteins over the GAPDH (10494-1-AP; San Ying Biotechnology, China) band was quantified with the Gel-Pro Analyzer.

### *In vivo* study

The xenograft model on nude mice was generated and analyzed in accordance with the National Institutes of Health Guide for the Care and Use of Laboratory Animals. The analysis was performed as previously reported [29]. The expression of AGTR1 protein was measured using immunochemical analysis. Antigens were retrieved by pretreating dewaxed sections and processed with the Super Sensitive Link-Labeled Detection System (Biogenex, Menarini, Florence, Italy). Pictures were taken using a LEICA DM 4000B microscope. The animal study proposal was approved by the Institutional Animal Care and Use Committee (IACUC) of the University of Science and Technology of China. All of the mouse experimental procedures were performed in accordance with the Regulations for the Administration of Affairs Concerning Experimental Animals approved by the State Council of People’s Republic of China.

### Statistical analysis

Apoptosis assays, cell viability, quantitative RT-PCR, and luciferase reporter assays were performed in triplicate, the data are presented as the means, and the error bars indicate the S.D. Excel was used to process the data. The differences were considered statistically significant at *p* < 0.05 using Student’s *t* -test.

## Results

### AGTR1 negatively regulates the multi-chemoresistance of OS

**Fig. 1:**
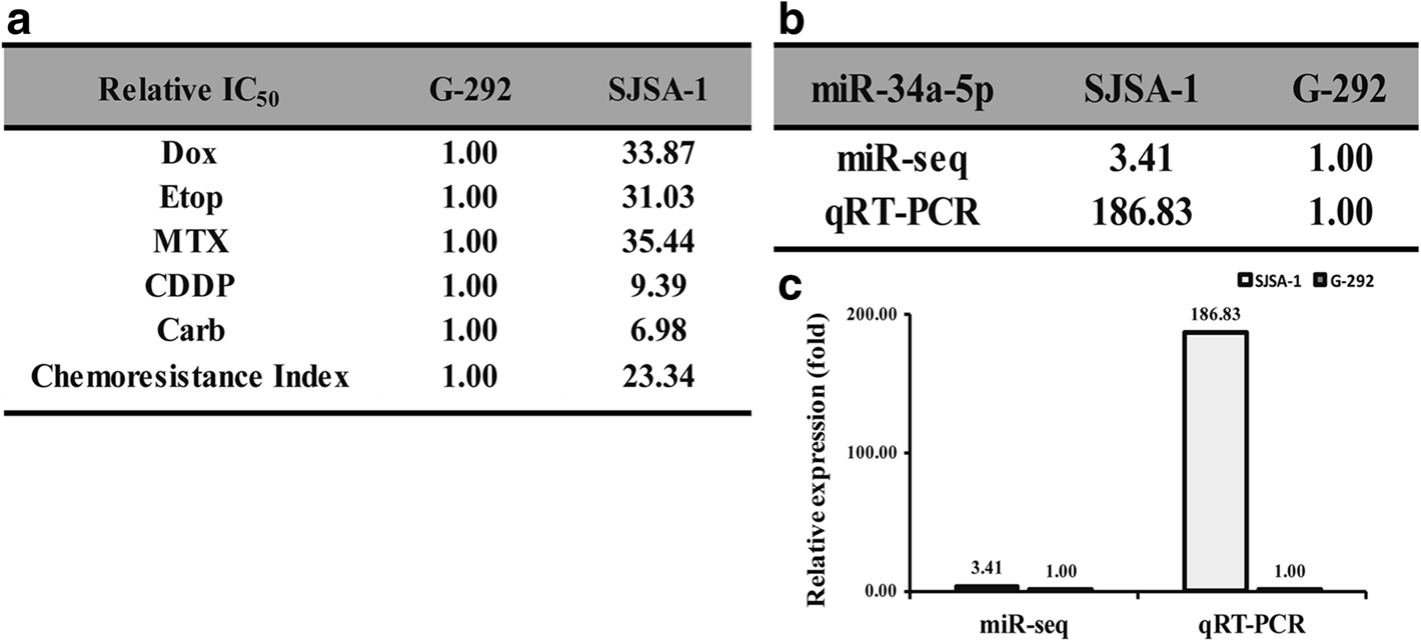
The miR-34a-5p expression differs in SJSA-1 and G-292 cell lines. Relative IC_50_ values (fold) of the two cell lines to four chemotherapeutics with the lowest IC_50_ as a reference (**a**). The relative miR-34a-5p level (fold) in two cell lines by both miR-seq and qRT-PCR analyses were shown in Table (**b**) and by qRT-PCR in plot (**c**)

Our previous result suggested that G-292 and SJSA-1 cell lines are the multi-chemosensitive and multi-drug resistant OS cell lines, respectively [29]. Indeed, the IC_50_ profiling experiments against the following four drugs: Doxorubicin (Dox), Etoposide (Etop), Cisplatin (CDDP), Carboplatin (Carb) demonstrated that SJSA-1 cells is more resistant against all the four drugs. The chemoresistance index of the SJSA-1 cells is 20.32, which is drastically higher than that of the G-292 cells (Fig. 1a). To find the mechanistic insights that govern the multi-chemoresistance of OS cells, we performed an RNA-seq-based miR-omic analysis of G-292 and SJSA-1 cells, and several related miRNAs were selected based on a reference survey (Additional file 1: Figure S1). MiR-34a-5p was selected as our target, which correlated well with the probe and RNA-seq analyses. The miR-34a-5p expression is 3.41-fold higher in the SJSA-1 cells than in the G-292 cells by miR-omic and 186.83-fold higher by qRT-PCR analysis (Fig. 1b and c).

**Fig. 2:**
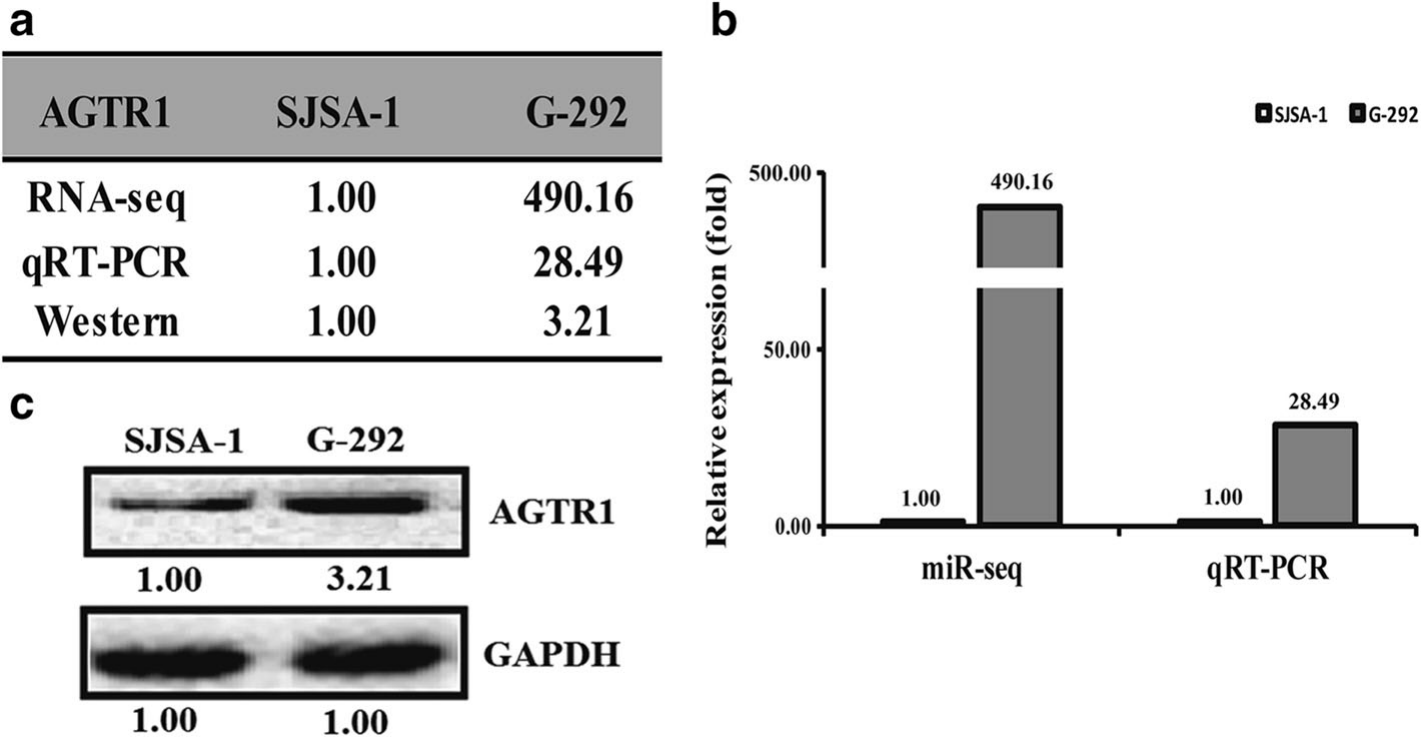
The AGTR1 level is higher in G-292 than in SJSA-1 cells. The relative level (fold) of the AGTR1 gene in SJSA-1 versus G-292 cells summarized in table (**a**), analyzed by Western analysis (**b**), by miR-seq and qRT-PCR analyses in plot (**c**)

A given miRNA usually suppresses the expression of various target genes and thus regulates related pathways. We thus proposed the target genes of miR-34a-5p based on the following websites: TargetScan (http://www.targetscan.org/), miRDB (http://mirdb.org/miRDB/) and microRNA.org (http://www.microrna.org/microrna/getMirnaForm.do). We subsequently compared the expression pattern of shared predicted mRNAs between G-292 and SJSA-1 cells by the RNA-seq based miR-omic analysis. Dozens of genes have been found that differentially expressed in the two cell lines. Among them, the AGTR1 gene is one of the most significantly differentiated genes that negatively correlate with miR-34a-5p expression (Additional file 1: Figures S1, Additional file 2: Figures S2 and Additional file 3: Figures S3. Consequently, the expression level of AGTR1 was higher in G-292 than SJSA-1 at both mRNA (RNA-seq based miR-omic: 490.16:1, and qRT-PCR analysis: 28.49:1) and protein level (western blot: 3.21:1) (Figs. 2a b and 2c). The lower expression of AGTR1 in multi-chemoresistant cells SJSA-1 suggests that AGTR1 is a negative regulator of OS multi-chemoresistance.

### MiR-34a-5p directly targets the AGTR1 gene in OS cells

**Fig. 3:**
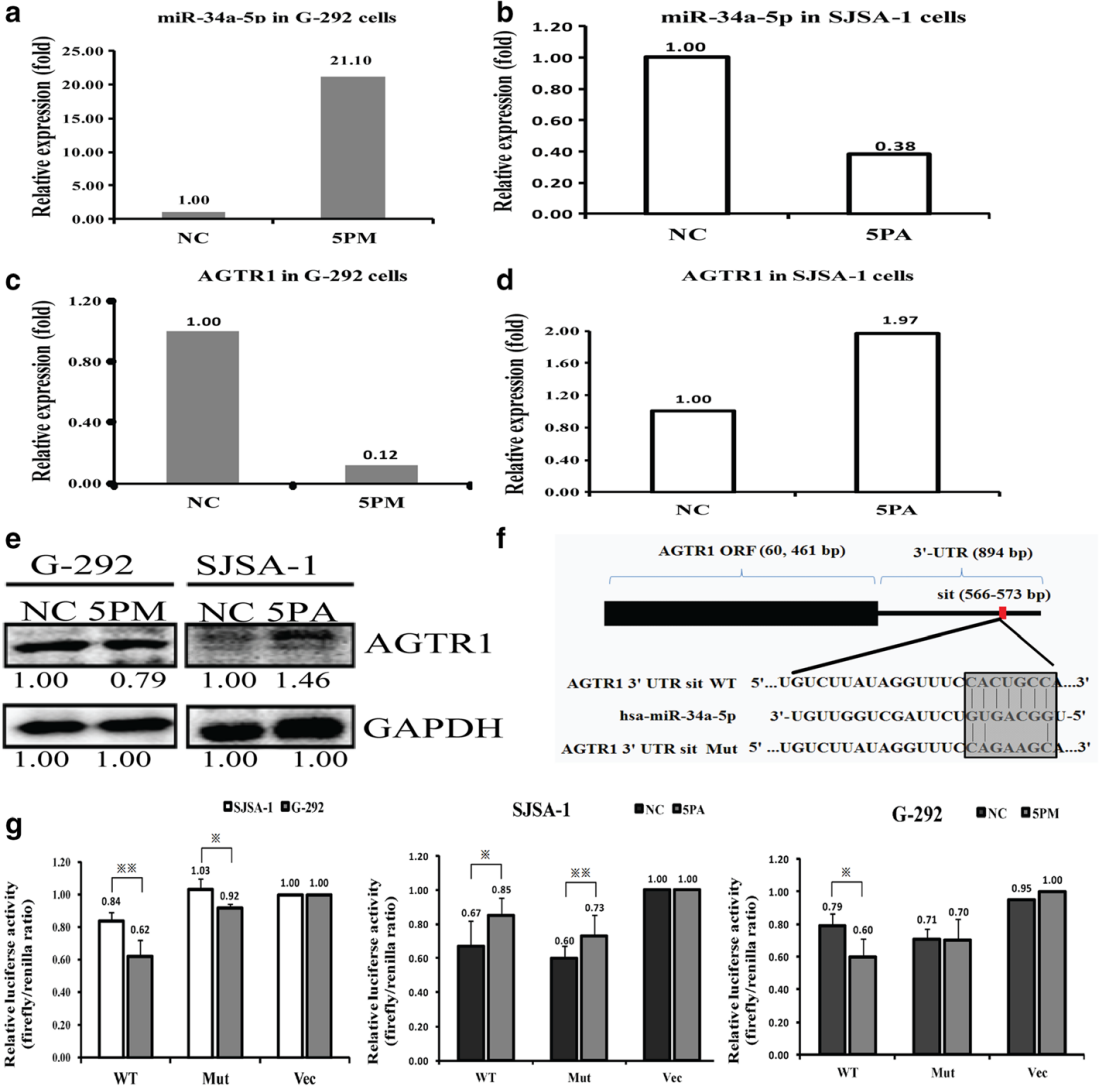
The AGTR1 is a direct target of miR-34a-5p in OS cells. The levels of miR-34a-5p (**a** and **b**), the AGTR1 mRNA (**c** and **d**) and protein (**e**) in the miR-34a-5p mimic (5PM) transfected G-292 cells and the miR-34a-5p antagomiR (5PA) transfected SJSA-1 cells versus the negative control (NC), determined by qRT-PCR or Western analyses. **f** The sequences of the wild-type and mutant 3'-UTR region of AGTR1 gene. The perfectly matched region of AGTR1 3'-UTR with miR-34a-5p were marked in shadow. **g** The relative luciferase activity (fold) of the reporter with wild-type (WT) AGTR1-UTR or mutant were determined in the miR-34a-5p mimic (in G-292) or antagomiR (in SJSA-1) or Mock transfected OS cells. The reporter without AGTR1-UTR (Vec) was used as a reference. The Renilla luciferase activity of a co-transfected control plasmid was used to control the transfection efficacy. The representative results from three independent experiments shown. *P value < 0.05; **P value < 0.01

The miR-34a-5p level was dramatically higher in SJSA-1 cells than G-292 cells. We found that AGTR1 negatively correlates with the level of miR-34a-5p. To check whether AGTR1 is one of the authentic targets of miR-34a-5p, we detected the AGTR1 level in the miR-34a-5p mimic transfected G-292 and the antagomiR transfected SJSA-1 cells versus the NC (scramble sequence control) transfected. The transfection of miR-34a-5p mimic in G-292 cells increased its expression to about 21-fold, whereas the transfection of miR-34a-5p antagomiR in SJSA-1 significantly decreased its level to 38% (Fig. 3a and b). In agreement with the changes of the miR-34a-5p level, a miR-34a-5p mimic transfection decreased the AGTR1 mRNA to 12% (Fig. 3c) and protein to nearly 79% (Fig. 3e) compared to that in the NC transfected G-292 cells. By contrast, miR-34a-5p antagomiR transfection increased the mRNA level of AGTR1 by 1.97 folds (Fig. 3d) and the protein level by 1.46 folds in SJSA-1cells (Fig. 3e).

To further confirm whether AGTR1 is a direct target of miR-34a-5p, we cloned the wild-type AGTR1 gene at the downstream of the Renilla luciferase gene in pGL3-control vector (Promega) to create pGL3-AGTR1 UTR WT or pGL3-AGTR1 UTR Mut (Fig. 3f). The constructs pGL3-AGTR1 UTR WT or pGL3-AGTR1 UTR Mut and pGL3 enhancer control were transfected into G-292 and SJSA-1 cells respectively, to determine the function of miR-34a-5p in different OS cells. The pGL3-AGTR1-UTR WT gave the relative luciferase activities of 0.84 and 0.62 in SJSA-1 and G-292 cells, respectively (Fig. 3g). The transfection of miR-34a-5p-mimic into G-292 cells significantly brought down the luciferase activity of pGL3-AGTR1-UTR WT construct, whereas the control cells showed almost the same activity upon the transfection of miR-34a-5p-mimic (Fig. 3g). Meanwhile, the transfection of miR-34a-5p-antagomiR into SJSA-1 cells raised the luciferase activity of pGL3-AGTR1-UTR WT construct (Fig. 3g). Furthermore, the mutation of the 3'-UTR showed similar effect as the wild type with the transfection miR-34a-5p-antagomiR into SJSA-1 cells. By contrast, the comparable luciferase activity was detected in the pGL3-AGTR1-UTR Mut with the transfection of miR-34a-5p-mimic into G-292 cells, suggesting that miR-34a-5p indeed targets the 3’-UTR region of AGTR1 (Fig. 3g). Getting together, AGTR1 is indeed, a direct target of miR-34a-5p and may dedicate the miR-34a-5p’s promoting effect on the OS drug resistance.

### The AGTR1 expression negatively correlates with the miR-34a-5p’s promoting effect on OS drug resistance

**Fig. 4:**
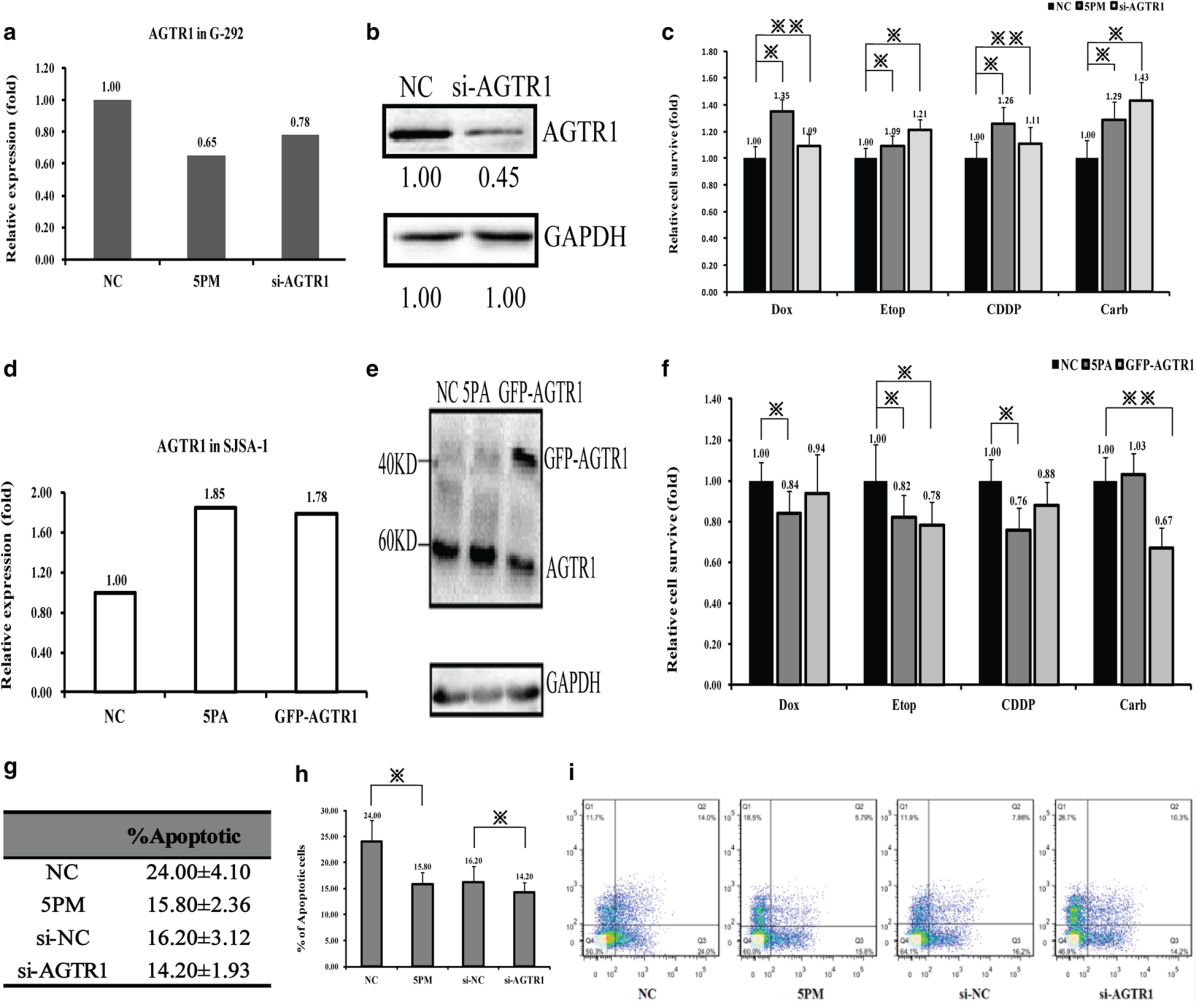
The effects of forced reversal of miR-34a-5p or AGTR1 levels on the chemoresistance of G-292 and SJSA-1 cells. **a** The mRNA level of AGTR1 detected by qRT-PCR in the 5PM-, or siRNA- versus the NC-transfected G-292 cells. **b** The levels of AGTR1 protein detected by Western analysis in the siRNA- versus the NC-transfected G-292 cells. **c** The IC_50_ dosed drug-triggered cell death of G-292 cells transfected with miR-34a-5p mimic (5PM) or the gene specific siRNAs versus the negative control (NC). **d** The level of AGTR1 by qRT-PCR in the 5PA, or GFP-AGTR1 versus the NC transfected SJSA-1 cells. **e** The levels of AGTR1 protein by Western analysis in the GFP-AGTR1 versus the NC transfected SJSA-1 cells. **f** The IC_50_ dosed drug-triggered cell death of SJSA-1 cells transfected by miR-34a-5p mimic (5PA) or GFP-AGTR1 versus the negative control (NC). **g**, **h** and **i** The effects of the forced reversal of both miR-34a-5p and AGTR1 levels on the apoptosis by FACS analysis of G-292 cells in plot and in the original. (*, *P* < 0.05)

To investigate the role of AGTR1 in the OS chemoresistance, we first transfected si-AGTR1 into G-292 cells and tested the level of AGTR1. The transfection of si-AGTR1 indeed decreased the level of AGTR1 at both mRNA (0.78:1) and protein level (0.45:1), compared to the control cells (Fig. 4a and b). A similar effect was also found with the transfection of miR-34a-5p-mimic into G-292 cells. We then compared the cell apoptosis triggered by an IC_50_ dosed drug in the miR-34a-5p mimic or si-AGTR1 transfected G-292 cells. The transfection of miR-34a-5p mimic or si-AGTR1 in G-292 cells increased the chemoresistance to some extent against the following four drugs: Dox, Etop, CDDP, Carb (Fig. 4c). Afterwards, we increased the level of AGTR1 by transfection of miR-34a-5p antagomiR or overexpression of AGTR1 in SJSA-1 cells. In agreement with the elevated level of AGTR1 in both mRNA and protein levels (Fig. 4d and e), the cell survival rate was slightly decreased for all the four drugs, except for Carb (Fig. 4f). The results correlate well with the negative regulation of AGTR1 in the multi-drug resistance of OS cells. In accordance with its negative effect on drug resistance, a siRNA-mediated AGTR1 repression reduced the apoptotic cells from 16.2 to 14.2%, indicating an elevated cell survival rate upon the addition of si-AGTR1 into G-292 cells (Fig. 4g, h and i). A similar effect was also found in the miR-34a-5p-mimic transfected G-292 cells (Fig. 4g, h and i). Taken together, The AGTR1 gene does contribute a great deal to the miR-34a-5p’s promoting effect on the OS drug resistance.

### MiR-34a-5p promotes both growth and Dox drug resistance of the G-292 and SJSA-1-derived tumor xenografts in nude mice

**Fig. 5:**
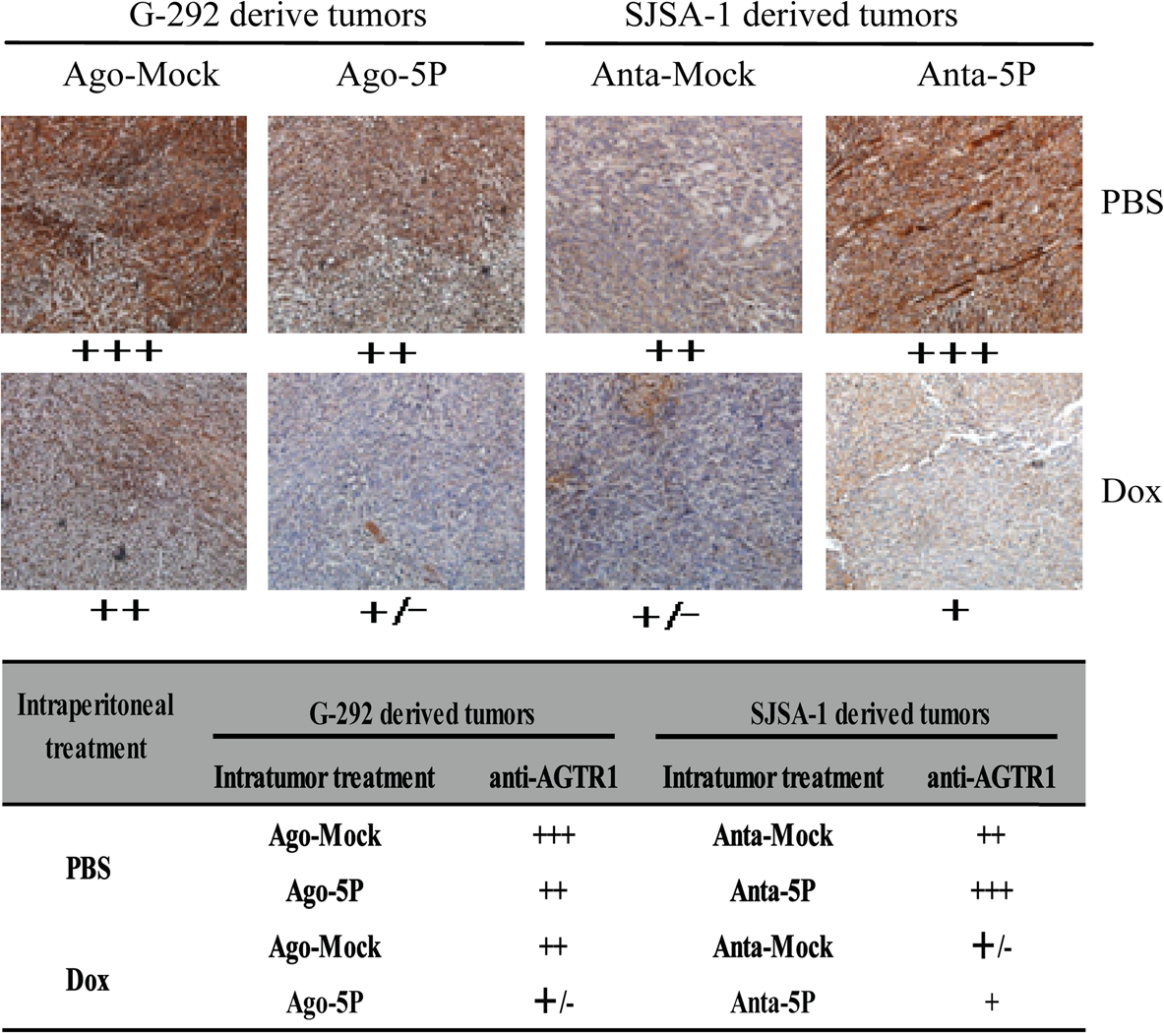
The AGTR1 level (immunohistochemical staining) in tissue slides of the miR-34a-5p agomiR-injected G-292 and miR-34a-5p antagomiR-injected SJSA-1 tumor xenografts versus the NC-injected tumor xenografts. The levels of AGTR1 protein in each group are summarized in the table

Recently, miR-34a-5p was shown to promote Dox chemoresistance of OS in tumor xenografts of nude mice by repressing its target gene CD117 [29]. In this study, we semi-quantified the levels of AGTR1 protein by immuno-histological analysis in the same section of mice tumor tissues that were injected with either Dox or PBS. The intratumoral injection of miR-34a-5p’s agomiR into G-292 decreased AGTR1 expression. By contrast, the injection of miR-34a-5p’s antagomiR into SJSA-1 increased AGTR1 expression in Dox- or PBS-treated mice (Fig. 5). The results further confirmed that miR-34a-5p has a significant positive effect on both the growth and chemoresistance of OS cells in vitro and cell-derived tumor xenografts in nude mice (Additional file 4: Figures S4).

## Discussion

As the well studied miRNA, the miR-34a has been associated with different types of cancer, including Ewing’s sarcoma [30]; colorectal cancer [31] and etc. MiR-34a has several direct targets, such as Notch, c-Myc, c-Met, c-Kit and etc. [32]. The miR-34a targets Notch1 and Notch2 in glioblastoma and medulloblastoma [17]. The miR-34a suppresses invasion of cervical carcinoma and choriocarcinoma cells by targeting Notch1 and Jagged1 [33]. Besides, evidence showed that miR-34a is also involved in cancer drug resistance [34–36], which correlates well with our present work. Here we showed that miR-34a-5p also involves in the multi-drug resistance of OS [29]. We performed a RNA-seq assay of SJSA-1 and G-292 cell lines and found that the expression of a dozen of genes vary dramatically, including the AGTR1 gene that negatively correlates with the OS drug resistance (Fig. 2). In addition, we systematically performed experiments in cultured cells and tumor xenografts to address the role and mechanism of the AGTR1 gene in the context of OS drug resistance.

AGTR1 was reported to be involved in diverse cancers, and is a potential therapeutic target for anticancer treatment. For example, inhibition of the AGTR1 expression in human epithelial ovarian carcinomas reduces cell survival and angiogenesis by repressing the level of VEGF [37]. AGTR1 is also involved in the invasion, migration or tumorigenesis of endometrial carcinoma and breast cancer *via* the up-regulation of VEGF [38–40]. Up-regulation of AGTR1 expression by nuclease domain containing-1 promotes cell invasion and migration, which in return activates the ERK signaling pathway in hepatocellular carcinoma [41]. All these studies suggest that AGTR1 might serve as a target for the above mentioned cancers. In agreement with the previous findings, here we demonstrated that the expression of AGTR1 is associated with the multi-drug resistance of OS cell lines. However, the detailed mechanism for the AGTR1-mediated OS drug-resistance remains to be clarified.

## Conclusion

In this work, we identified that AGTR1 is a direct target of miR-34a-5p, and negatively regulates the multi-drug resistance of OS. We conclude that increased expression of miR-34a-5p in the OS cells can be potentially used as an indicator of chemoresistance and for relapse in serious OS patients. Targeting miR-34a-5p and its target gene miR-34a-5p through novel therapeutics may provide an important strategy to overcome OS chemoresistance.

## Additional files

Additional file 1: Figure S1. The interested miRNA and mRNA genes based on the websites and RNA-seq analysis. A dozen of miRNAs were differentially expressed in the multi-chemoresistant OS cells SJSA-1 and the multi-chemosensitive OS cells G-292 and MG63.2 based on the websites, and the ratio over 2 of SJSA-1/G-292 based on RNA-seq-based miR-omic analysis were showed in descending order, has-miR-34a-5p was one of them (A). Reference to similar methods, the downstream genes of has-miR-34a-5p were also showed, the ratio of G-292/SJSA-1 based on RNA-seq analysis were showed in descending order, AGTR1 is located (B). (TIF 88.5 mb)
Additional file 2: Figure S2. The 410 differentially expressed miRNAs were showed through the miR-omic analysis between SJSA-1 and G-292 cells, the ratio of G-292/SJSA-1 was also presented. The target gene miR-34a-5p located in. (PDF 284 kb)
Additional file 3: Figure S3. The 17030 differentially expressed mRNAs were showed through the RNA-seq analysis between SJSA-1 and G-292 cells, the ratio of G-292/SJSA-1 was also presented, and the target gene AGTR1 also located in. (PDF 11.3 mb)
Additional file 4: Figure S4. The protein level of p53 detected by western in NC, 5PA, GFP, GFP-AGTR1 transfected SJSA-1 cells, and the NC, 5PM, NC, si-AGTR1 transfected G-292 cells. (TIF 17.8 mb)
