# Supplementary material for: The miR-34a-5p promotes the multi-chemoresistance of osteosarcoma via repression of the AGTR1 gene
Source: BMC Cancer. 2017 Jan 10;17:45. doi: 10.1186/s12885-016-3002-x (PMC5223322; doi:10.1186/s12885-016-3002-x)
Supplement: Additional file 2: Figure S2. — The 410 differentially expressed miRNAs were showed through the miR-omic analysis between SJSA-1 and G-292 cells, the ratio of G-292/SJSA-1 was also presented. The target gene miR-34a-5p located in. (PDF 284 kb) [file 12885_2016_3002_MOESM2_ESM.pdf]

	miR_name	SJSA-1	G-292	G292/SJSA
1	hsa-miR-138-5p	2	1135	567.50
2	hsa-miR-708-5p	3	1395	465.00
3	hsa-miR-296-5p	2	512	256.00
4	hsa-miR-139-3p	1	213	213.00
5	hsa-miR-378a-3p	61	7359	120.64
6	hsa-miR-708-3p	1	120	120.00
7	hsa-miR-18a-5p	1	102	102.00
8	hsa-miR-9-5p	1	88	88.00
9	hsa-miR-935	5	426	85.20
10	hsa-miR-129-2-3p	3	239	79.67
11	hsa-miR-4745-5p	1	69	69.00
12	hsa-miR-340-5p	9	605	67.22
13	hsa-miR-378c	4	244	61.00
14	hsa-miR-7111-5p	2	98	49.00
15	hsa-miR-1180-3p	2	94	47.00
16	hsa-miR-129-5p	2	87	43.50
17	hsa-miR-183-3p	1	43	43.00
18	hsa-miR-31-3p	1	35	35.00
19	hsa-miR-100-3p	1	33	33.00
20	hsa-miR-148a-3p	6	188	31.33
21	hsa-miR-92a-1-5p	322	9929	30.84
22	hsa-miR-139-5p	1	30	30.00
23	hsa-miR-9-3p	3	86	28.67
24	hsa-miR-6799-5p	1	27	27.00
25	hsa-miR-1910-3p	3	79	26.33
26	hsa-miR-363-5p	1	26	26.00
27	hsa-miR-2355-5p	1	24	24.00
28	hsa-miR-5088-5p	1	24	24.00
29	hsa-miR-4706	1	23	23.00
30	hsa-miR-17-3p	31	666	21.48
31	hsa-miR-1275	8	159	19.88
32	hsa-miR-5001-5p	2	39	19.50
33	hsa-miR-365b-5p	92	1750	19.02
34	hsa-miR-3200-5p	1	19	19.00
35	hsa-miR-345-5p	1	19	19.00
36	hsa-miR-589-3p	2	37	18.50
37	hsa-miR-598-3p	54	987	18.28
38	hsa-miR-1260b	2	36	18.00
39	hsa-miR-6720-3p	3	53	17.67
40	hsa-miR-1304-5p	3	52	17.33
41	hsa-miR-942-5p	5	77	15.40

42	hsa-miR-6871-5p	1	15	15.00
43	hsa-miR-92a-3p	488	7259	14.88
44	hsa-miR-4741	7	104	14.86
45	hsa-miR-19b-3p	19	279	14.68
46	hsa-miR-3679-5p	4	58	14.50
47	hsa-miR-7-1-3p	3	42	14.00
48	hsa-miR-6862-5p	1	14	14.00
49	hsa-miR-3151-5p	1	14	14.00
50	hsa-miR-101-3p	509	6937	13.63
51	hsa-miR-1	53	700	13.21
52	hsa-miR-210-5p	1	13	13.00
53	hsa-miR-4458	1	13	13.00
54	hsa-miR-760	14	179	12.79
55	hsa-miR-17-5p	124	1576	12.71
56	hsa-miR-193a-3p	15	190	12.67
57	hsa-miR-4425	8	101	12.63
58	hsa-miR-421	12	151	12.58
59	hsa-miR-6805-5p	9	113	12.56
60	hsa-miR-941	25	312	12.48
61	hsa-miR-29a-5p	6	72	12.00
62	hsa-miR-8072	4	48	12.00
63	hsa-miR-10a-3p	3	36	12.00
64	hsa-miR-3183	1	12	12.00
65	hsa-miR-106a-5p	1	12	12.00
66	hsa-miR-6777-5p	3	35	11.67
67	hsa-miR-1268a	303	3514	11.60
68	hsa-miR-125b-1-3p	4	46	11.50
69	hsa-miR-652-3p	3	33	11.00
70	hsa-miR-6812-5p	1	11	11.00
71	hsa-miR-548a-3p	1	11	11.00
72	hsa-miR-6876-5p	1	11	11.00
73	hsa-miR-6832-5p	1	11	11.00
74	hsa-miR-1268b	342	3743	10.94
75	hsa-miR-877-5p	457	4903	10.73
76	hsa-miR-20a-5p	68	714	10.50
77	hsa-miR-95-3p	2	21	10.50
78	hsa-miR-589-5p	11	114	10.36
79	hsa-miR-19a-3p	4	41	10.25
80	hsa-miR-1301-3p	24	243	10.13
81	hsa-miR-92a-2-5p	3	30	10.00
82	hsa-miR-590-5p	2	20	10.00
83	hsa-miR-885-3p	1	10	10.00
84	hsa-miR-20b-3p	5	48	9.60

85	hsa-miR-4707-3p	6	56	9.33
86	hsa-miR-6747-5p	2	18	9.00
87	hsa-miR-212-5p	1	9	9.00
88	hsa-miR-6858-5p	1	9	9.00
89	hsa-miR-3187-3p	1	9	9.00
90	hsa-miR-3173-3p	3	26	8.67
91	hsa-miR-625-5p	11	95	8.64
92	hsa-miR-196b-5p	47	402	8.55
93	hsa-miR-3138	11	92	8.36
94	hsa-miR-193a-5p	50	416	8.32
95	hsa-miR-210-3p	32	256	8.00
96	hsa-miR-6890-5p	1	8	8.00
97	hsa-miR-4748	1	8	8.00
98	hsa-miR-769-5p	1	8	8.00
99	hsa-miR-342-3p	183	1414	7.73
100	hsa-miR-6735-5p	3	23	7.67
101	hsa-miR-339-3p	21	160	7.62
102	hsa-miR-1246	3	22	7.33
103	hsa-miR-324-5p	10	73	7.30
104	hsa-miR-29b-3p	309	2182	7.06
105	hsa-miR-100-5p	546	3845	7.04
106	hsa-miR-219a-1-3p	1	7	7.00
107	hsa-miR-4469	1	7	7.00
108	hsa-miR-6865-5p	1	7	7.00
109	hsa-miR-548aa	1	7	7.00
110	hsa-miR-548t-3p	1	7	7.00
111	hsa-miR-4477b	1	7	7.00
112	hsa-miR-31-5p	1328	9228	6.95
113	hsa-miR-5187-5p	7	48	6.86
114	hsa-miR-342-5p	19	130	6.84
115	hsa-miR-455-3p	21	140	6.67
116	hsa-miR-641	5	33	6.60
117	hsa-miR-629-5p	22	144	6.55
118	hsa-miR-149-3p	18	117	6.50
119	hsa-miR-449c-5p	31	199	6.42
120	hsa-miR-301a-3p	13	83	6.38
121	hsa-miR-363-3p	8	51	6.38
122	hsa-miR-301a-5p	57	363	6.37
123	hsa-miR-29b-1-5p	11	70	6.36
124	hsa-miR-324-3p	3	19	6.33
125	hsa-miR-3175	2	12	6.00
126	hsa-miR-6880-5p	2	12	6.00
127	hsa-miR-6767-5p	1	6	6.00

128	hsa-miR-4722-5p	1	6	6.00
129	hsa-miR-330-3p	615	3677	5.98
130	hsa-miR-499a-5p	12	71	5.92
131	hsa-miR-4485	6	35	5.83
132	hsa-miR-339-5p	21	122	5.81
133	hsa-miR-3615	9	52	5.78
134	hsa-miR-942-3p	3	17	5.67
135	hsa-miR-449a	5	28	5.60
136	hsa-miR-455-5p	12	67	5.58
137	hsa-miR-132-5p	2	11	5.50
138	hsa-miR-940	7	38	5.43
139	hsa-miR-500a-3p	3	16	5.33
140	hsa-miR-153-3p	3	16	5.33
141	hsa-miR-3654	3	16	5.33
142	hsa-miR-222-5p	28	148	5.29
143	hsa-miR-3935	5	26	5.20
144	hsa-miR-1254	10	51	5.10
145	hsa-miR-664b-5p	10	51	5.10
146	hsa-miR-1293	4	20	5.00
147	hsa-miR-664a-3p	2	10	5.00
148	hsa-miR-196b-3p	4	19	4.75
149	hsa-miR-6852-5p	3	14	4.67
150	hsa-miR-191-3p	3	14	4.67
151	hsa-miR-125b-5p	362	1677	4.63
152	hsa-miR-1292-5p	16	74	4.63
153	hsa-miR-3154	8	37	4.63
154	hsa-miR-425-3p	13	60	4.62
155	hsa-miR-7-5p	49	224	4.57
156	hsa-miR-548j-5p	11	50	4.55
157	hsa-miR-766-5p	24	109	4.54
158	hsa-miR-30c-1-3p	8	36	4.50
159	hsa-miR-4466	4	18	4.50
160	hsa-miR-449b-5p	2	9	4.50
161	hsa-miR-4734	2	9	4.50
162	hsa-miR-3661	2	9	4.50
163	hsa-miR-33a-5p	146	651	4.46
164	hsa-miR-197-5p	60	263	4.38
165	hsa-miR-769-3p	6	26	4.33
166	hsa-miR-625-3p	23	98	4.26
167	hsa-miR-3064-5p	4	17	4.25
168	hsa-miR-3141	9	38	4.22
169	hsa-miR-5010-5p	62	260	4.19
170	hsa-miR-106b-5p	263	1082	4.11

171	hsa-miR-505-5p	76	310	4.08
172	hsa-miR-374b-3p	16	65	4.06
173	hsa-miR-4521	77	310	4.03
174	hsa-miR-497-5p	10	40	4.00
175	hsa-miR-301b	4	16	4.00
176	hsa-miR-2355-3p	3	12	4.00
177	hsa-miR-6875-5p	2	8	4.00
178	hsa-miR-624-5p	2	8	4.00
179	hsa-miR-3928-3p	12	46	3.83
180	hsa-miR-25-5p	2242	8525	3.80
181	hsa-miR-93-5p	552	2076	3.76
182	hsa-miR-6511b-5p	8	30	3.75
183	hsa-miR-3193	4	15	3.75
184	hsa-miR-3143	4	15	3.75
185	hsa-miR-3140-3p	3	11	3.67
186	hsa-miR-221-5p	1272	4639	3.65
187	hsa-miR-183-5p	32	116	3.63
188	hsa-miR-6511a-5p	8	29	3.63
189	hsa-miR-3127-5p	5	18	3.60
190	hsa-miR-766-3p	5	18	3.60
191	hsa-miR-1307-3p	794	2826	3.56
192	hsa-miR-146b-5p	98	343	3.50
193	hsa-miR-203a	4	14	3.50
194	hsa-miR-3177-3p	2	7	3.50
195	hsa-miR-548n	2	7	3.50
196	hsa-miR-671-3p	2	7	3.50
197	hsa-miR-3934-5p	9	31	3.44
198	hsa-miR-96-5p	22	75	3.41
199	hsa-miR-10a-5p	1511	5094	3.37
200	hsa-miR-1262	27	91	3.37
201	hsa-miR-29a-3p	32565	1E+05	3.33
202	hsa-miR-590-3p	6	20	3.33
203	hsa-miR-3163	3	10	3.33
204	hsa-miR-148b-3p	59	195	3.31
205	hsa-miR-629-3p	4	13	3.25
206	hsa-miR-502-3p	5	16	3.20
207	hsa-miR-548l	5	16	3.20
208	hsa-miR-1271-5p	21	67	3.19
209	hsa-miR-484	23	73	3.17
210	hsa-miR-374b-5p	129	409	3.17
211	hsa-miR-3129-5p	18	57	3.17
212	hsa-miR-362-3p	6	19	3.17
213	hsa-miR-1307-5p	224	699	3.12

214	hsa-miR-186-5p	109	334	3.06
215	hsa-miR-27a-3p	1039	3182	3.06
216	hsa-miR-21-3p	149	454	3.05
217	hsa-miR-6721-5p	32	97	3.03
218	hsa-miR-584-5p	345	1045	3.03
219	hsa-miR-6825-5p	12	36	3.00
220	hsa-miR-103a-2-5p	3	9	3.00
221	hsa-miR-4667-5p	2	6	3.00
222	hsa-miR-6779-5p	2	6	3.00
223	hsa-miR-5585-3p	2	6	3.00
224	hsa-miR-16-1-3p	2	6	3.00
225	hsa-miR-30e-5p	117	340	2.91
226	hsa-miR-24-3p	3920	11386	2.90
227	hsa-miR-222-3p	9136	26485	2.90
228	hsa-miR-1285-3p	18	52	2.89
229	hsa-miR-15b-3p	18	51	2.83
230	hsa-miR-221-3p	17810	50035	2.81
231	hsa-miR-1277-5p	5	14	2.80
232	hsa-miR-887-3p	9	25	2.78
233	hsa-miR-194-5p	9	25	2.78
234	hsa-miR-4750-5p	4	11	2.75
235	hsa-miR-6730-5p	4	11	2.75
236	hsa-miR-3605-5p	22	60	2.73
237	hsa-miR-125a-3p	14	38	2.71
238	hsa-miR-3130-3p	7	19	2.71
239	hsa-miR-3190-3p	6	16	2.67
240	hsa-miR-106b-3p	189	503	2.66
241	hsa-miR-192-5p	640	1674	2.62
242	hsa-miR-151a-3p	263	683	2.60
243	hsa-miR-15b-5p	357	918	2.57
244	hsa-miR-5090	9	23	2.56
245	hsa-miR-3192-5p	31	79	2.55
246	hsa-miR-25-3p	5897	15010	2.55
247	hsa-miR-532-3p	25	63	2.52
248	hsa-miR-30e-3p	27	68	2.52
249	hsa-miR-32-5p	17	42	2.47
250	hsa-miR-320d	6	14	2.33
251	hsa-miR-195-5p	14	32	2.29
252	hsa-miR-660-5p	14	32	2.29
253	hsa-miR-103a-3p	41833	95214	2.28
254	hsa-miR-30b-3p	75	169	2.25
255	hsa-miR-24-2-5p	20	45	2.25
256	hsa-miR-505-3p	4	9	2.25

257	hsa-miR-1306-3p	21	47	2.24
258	hsa-miR-7845-5p	5	11	2.20
259	hsa-miR-23a-3p	3519	7721	2.19
260	hsa-miR-128-3p	1979	4276	2.16
261	hsa-miR-182-5p	111	236	2.13
262	hsa-miR-181d-5p	744	1581	2.13
263	hsa-miR-320a	129750	3E+05	2.11
264	hsa-miR-29c-3p	310	644	2.08
265	hsa-miR-26b-5p	1966	4041	2.06
266	hsa-miR-1255a	57	117	2.05
267	hsa-miR-361-5p	39	80	2.05
268	hsa-miR-151a-5p	202	414	2.05
269	hsa-miR-197-3p	24	49	2.04
270	hsa-miR-483-5p	1631	3313	2.03
271	hsa-miR-320c	123	249	2.02
272	hsa-miR-6785-5p	10	20	2.00
273	hsa-miR-1306-5p	7	14	2.00
274	hsa-miR-148b-5p	7	14	2.00
275	hsa-miR-628-5p	7	14	2.00
276	hsa-miR-204-3p	6	12	2.00
277	hsa-miR-671-5p	4	8	2.00
278	hsa-miR-23a-5p	275	541	1.97
279	hsa-miR-196a-5p	1492	2884	1.93
280	hsa-miR-6087	349	673	1.93
281	hsa-miR-107	21324	40909	1.92
282	hsa-miR-130a-3p	721	1376	1.91
283	hsa-miR-628-3p	16	29	1.81
284	hsa-miR-425-5p	38	68	1.79
285	hsa-miR-219b-3p	4	7	1.75
286	hsa-miR-4454	4	7	1.75
287	hsa-miR-132-3p	2482	4320	1.74
288	hsa-miR-548h-5p	34	57	1.68
289	hsa-miR-181c-3p	9	15	1.67
290	hsa-miR-27a-5p	20	33	1.65
291	hsa-miR-320b	1002	1646	1.64
292	hsa-miR-1277-3p	11	18	1.64
293	hsa-miR-744-5p	989	1606	1.62
294	hsa-miR-30c-2-3p	111	180	1.62
295	hsa-miR-374a-3p	165	267	1.62
296	hsa-miR-4687-3p	5	8	1.60
297	hsa-miR-1273c	5	8	1.60
298	hsa-miR-155-5p	1411	2253	1.60
299	hsa-miR-185-5p	8000	12173	1.52

300	hsa-miR-619-5p	8	12	1.50
301	hsa-miR-3164	4	6	1.50
302	hsa-let-7a-3p	23	34	1.48
303	hsa-miR-548k	19	28	1.47
304	hsa-miR-181c-5p	72	105	1.46
305	hsa-miR-99b-5p	850	1225	1.44
306	hsa-miR-99a-5p	56	80	1.43
307	hsa-miR-4286	10	14	1.40
308	hsa-miR-30d-5p	3205	4451	1.39
309	hsa-miR-2110	65	90	1.38
310	hsa-miR-16-5p	2175	3000	1.38
311	hsa-miR-30b-5p	17	23	1.35
312	hsa-miR-15a-5p	238	318	1.34
313	hsa-miR-6734-5p	23	30	1.30
314	hsa-miR-16-2-3p	47	60	1.28
315	hsa-miR-27b-3p	226	279	1.23
316	hsa-miR-21-5p	37038	45700	1.23
317	hsa-miR-423-3p	390	472	1.21
318	hsa-miR-331-3p	200	239	1.20
319	hsa-miR-374a-5p	118	141	1.19
320	hsa-miR-181b-3p	11	13	1.18
321	hsa-miR-615-3p	6	7	1.17
322	hsa-miR-32-3p	6	7	1.17
323	hsa-miR-365a-3p	155	179	1.15
324	hsa-miR-365b-3p	155	179	1.15
325	hsa-miR-6780a-5p	7	8	1.14
326	hsa-miR-664a-5p	83	93	1.12
327	hsa-miR-185-3p	34	38	1.12
328	hsa-miR-130b-3p	371	414	1.12
329	hsa-let-7a-5p	844639	9E+05	1.09
330	hsa-miR-574-3p	28	30	1.07
331	hsa-miR-486-5p	47	50	1.06
332	hsa-let-7g-5p	21532	22873	1.06
333	hsa-miR-548e-3p	17	18	1.06
334	hsa-miR-2682-5p	43	45	1.05
335	hsa-miR-765	22	23	1.05
336	hsa-miR-27b-5p	157	164	1.04
337	hsa-miR-423-5p	45439	45578	1.00
338	hsa-miR-181a-3p	125	125	1.00
339	hsa-miR-122-5p	35	35	1.00
340	hsa-miR-504-3p	25	25	1.00
341	hsa-miR-7641	13	13	1.00
342	hsa-miR-6889-5p	7	7	1.00

343	hsa-miR-30a-3p	272	264	0.97
344	hsa-miR-23b-3p	218	205	0.94
345	hsa-miR-92b-3p	1207	1135	0.94
346	hsa-miR-1278	48	45	0.94
347	hsa-miR-22-5p	115	107	0.93
348	hsa-miR-4743-5p	12	11	0.92
349	hsa-miR-30c-5p	86	78	0.91
350	hsa-miR-30a-5p	1689	1501	0.89
351	hsa-miR-34a-3p	8	7	0.88
352	hsa-miR-181a-2-3p	177	154	0.87
353	hsa-miR-503-5p	546	474	0.87
354	hsa-miR-181b-5p	6936	5925	0.85
355	hsa-miR-191-5p	12142	10091	0.83
356	hsa-miR-615-5p	61	49	0.80
357	hsa-miR-454-3p	15	12	0.80
358	hsa-let-7c-5p	23765	18768	0.79
359	hsa-miR-4435	14	11	0.79
360	hsa-miR-137	49	38	0.78
361	hsa-miR-450a-5p	13	10	0.77
362	hsa-miR-10b-5p	338	259	0.77
363	hsa-miR-146a-5p	294	225	0.77
364	hsa-miR-424-5p	193	137	0.71
365	hsa-miR-1294	10	7	0.70
366	hsa-miR-574-5p	106	73	0.69
367	hsa-let-7e-5p	117098	79153	0.68
368	hsa-miR-3664-3p	12	8	0.67
369	hsa-miR-140-3p	36059	23635	0.66
370	hsa-miR-1228-5p	26	17	0.65
371	hsa-miR-125a-5p	676	430	0.64
372	hsa-miR-22-3p	778	486	0.62
373	hsa-miR-224-5p	256	159	0.62
374	hsa-miR-28-5p	119	72	0.61
375	hsa-miR-92b-5p	1292	767	0.59
376	hsa-miR-205-5p	36	21	0.58
377	hsa-miR-130b-5p	19	11	0.58
378	hsa-miR-28-3p	311	178	0.57
379	hsa-miR-23b-5p	515	293	0.57
380	hsa-miR-99b-3p	154	87	0.56
381	hsa-miR-504-5p	16	9	0.56
382	hsa-let-7d-5p	26557	14753	0.56
383	hsa-miR-424-3p	1105	570	0.52
384	hsa-miR-181a-5p	10911	5625	0.52
385	hsa-miR-200c-3p	26	13	0.50

386	hsa-let-7i-5p	15762	7838	0.50
387	hsa-miR-642a-3p	30	14	0.47
388	hsa-miR-3179	13	6	0.46
389	hsa-let-7d-3p	162	71	0.44
390	hsa-miR-452-5p	985	422	0.43
391	hsa-miR-335-5p	40	17	0.43
392	hsa-miR-1255b-5p	15	6	0.40
393	hsa-miR-152-3p	1093	404	0.37
394	hsa-miR-6758-5p	45	14	0.31
395	hsa-miR-34a-5p	157	46	0.29
396	hsa-miR-1287-5p	28	8	0.29
397	hsa-miR-365a-5p	492	133	0.27
398	hsa-let-7b-5p	823266	2E+05	0.27
399	hsa-miR-450a-2-3p	26	7	0.27
400	hsa-miR-532-5p	100	26	0.26
401	hsa-miR-4421	27	7	0.26
402	hsa-let-7f-5p	803209	2E+05	0.26
403	hsa-miR-98-5p	827	211	0.26
404	hsa-miR-214-3p	297	56	0.19
405	hsa-miR-193b-3p	314	54	0.17
406	hsa-miR-193b-5p	1976	275	0.14
407	hsa-miR-199a-5p	58	8	0.14
408	hsa-miR-199b-3p	30478	3641	0.12
409	hsa-miR-199a-3p	30480	3641	0.12
410	hsa-miR-26a-5p	23063	1677	0.07
