# Supplementary material for: The miR-34a-5p promotes the multi-chemoresistance of osteosarcoma via repression of the AGTR1 gene
Source: BMC Cancer. 2017 Jan 10;17:45. doi: 10.1186/s12885-016-3002-x (PMC5223322; doi:10.1186/s12885-016-3002-x)
Supplement: Additional file 3: Figure S3. — The 17030 differentially expressed mRNAs were showed through the RNA-seq analysis between SJSA-1 and G-292 cells, the ratio of G-292/SJSA-1 was also presented, and the target gene AGTR1 also located in. (PDF 11.3 mb) [file 12885_2016_3002_MOESM3_ESM.pdf]

	SJSA-1	G-292	Symbol	G-292/SJSA-1
1	544.21717	0.01	CD248	54421.7173
2	113.75792	0.01	IFI27	11375.79227
3	2731.3045	0.3841446	KRT17	7110.095436
4	342.34305	0.0746197	TMEM119	4587.836555
5	45.135465	0.01	NPTX2	4513.546516
6	45.127401	0.01	GSTT1	4512.740106
7	40.978944	0.01	MRGPRF	4097.894441
8	439.32972	0.1117434	COL1A2	3931.594287
9	32.370141	0.01	PTN	3237.014059
10	88.086973	0.0275076	COL11A1	3202.283021
11	30.761876	0.01	SLC15A3	3076.187561
12	27.890865	0.01	CNN1	2789.086494
13	27.328399	0.01	HCP5	2732.839932
14	26.528211	0.01	ANXA3	2652.821055
15	25.629255	0.01	VCAM1	2562.92553
16	20.900406	0.01	THY1	2090.040574
17	345.66208	0.1671209	GLI1	2068.334997
18	19.359516	0.01	TRIM22	1935.951641
19	16.037547	0.01	LIMS2	1603.754657
20	15.344484	0.01	DPEP1	1534.448375
21	15.081543	0.01	FBLL1	1508.154278
22	14.616404	0.01	TEX11	1461.640417
23	14.441442	0.01	SELENBP1	1444.144235
24	12.957705	0.01	SYT1	1295.770493
25	11.888081	0.01	P2RX1	1188.808145
26	11.38534	0.01	ATP10A	1138.534041
27	11.023276	0.01	EIF1AY	1102.327592
28	10.897682	0.01	WFDC1	1089.76816
29	10.712124	0.01	LY96	1071.212384
30	10.609905	0.01	NR0B1	1060.990541
31	10.463172	0.01	CFI	1046.317159
32	261.58123	0.2569827	COL3A1	1017.894387
33	9.8976016	0.01	CARD16	989.7601591
34	9.7173368	0.01	FAP	971.7336809
35	9.1852381	0.01	AKR1C1	918.5238086
36	9.0421271	0.01	SUN3	904.2127074
37	8.9103979	0.01	CPXM1	891.039789
38	8.8494049	0.01	ARHGAP9	884.9404942
39	8.5937938	0.01	MIR616	859.3793822
40	163.48921	0.1904989	LUM	858.2159914
41	8.5344557	0.01	CFH	853.4455721

42	8.33598	0.01	PCDHB5	833.5980007
43	8.2768596	0.01	TNNC1	827.6859581
44	8.0488325	0.01	GFRA1	804.883247
45	7.6642326	0.01	PLA1A	766.4232565
46	7.624372	0.01	SNORD32A	762.4371958
47	7.1947446	0.01	LCN2	719.4744649
48	7.1637328	0.01	LYPD6B	716.3732819
49	7.1343973	0.01	ARSE	713.4397303
50	98.078727	0.1376693	CDKN2A	712.4226685
51	7.106741	0.01	SLC6A17	710.6740975
52	7.0393119	0.01	DDX3Y	703.931186
53	6.9721579	0.01	KRT1	697.2157901
54	6.9191929	0.01	MGMT	691.9192891
55	6.6170928	0.01	SLC1A7	661.7092833
56	6.4453454	0.01	SNORD88B	644.5345366
57	6.2402459	0.01	DRD1	624.0245929
58	6.1458298	0.01	KDM5D	614.5829764
59	275.33525	0.4495491	ACTG2	612.4698054
60	266.33914	0.4429623	RPS4Y1	601.268191
61	6.0085764	0.01	ACSL5	600.8576351
62	5.9823687	0.01	KERA	598.2368664
63	5.9271758	0.01	C1orf51	592.7175773
64	5.8993497	0.01	ZNF71	589.9349719
65	5.8530532	0.01	FOXS1	585.305322
66	5.8224425	0.01	NDN	582.2442546
67	5.7821827	0.01	TNFRSF14	578.2182664
68	5.7505381	0.01	NKX2-6	575.0538084
69	5.6780921	0.01	DMRTA2	567.8092123
70	5.657905	0.01	CYYR1	565.7904982
71	5.4841974	0.01	SNORD100	548.4197373
72	5.412974	0.01	SNORD69	541.2974031
73	5.3586576	0.01	PADI3	535.8657577
74	5.3435769	0.01	SNORD45C	534.3576928
75	5.1456667	0.01	SNORD76	514.5666671
76	5.1281229	0.01	HNMT	512.8122892
77	5.1062665	0.01	SDPR	510.6266467
78	5.0649459	0.01	CLCN4	506.4945873
79	5.001588	0.01	RNU6ATAC	500.1588004
80	4.9679073	0.01	CXCL5	496.7907277
81	19.165961	0.0391051	SPON1	490.1137147
82	4.8170201	0.01	C5orf56	481.7020077
83	4.8092192	0.01	SNORA31	480.9219235
84	4.7907931	0.01	ACVRL1	479.0793107

85	4.7697069	0.01	IP6K3	476.970687
86	4.7042777	0.01	CDX1	470.4277656
87	4.6177153	0.01	LOC100188947	461.7715322
88	4.5587391	0.01	HMSD	455.8739066
89	4.5236882	0.01	TAGAP	452.3688229
90	4.4794694	0.01	CDKN2B	447.9469386
91	4.463047	0.01	CTF1	446.3047
92	24.99159	0.0564718	TNFRSF10D	442.5499323
93	4.3851062	0.01	GALC	438.5106239
94	4.3830054	0.01	LRRC3	438.3005361
95	4.3570175	0.01	FAIM3	435.7017533
96	4.3435415	0.01	LRRC17	434.3541471
97	4.2680689	0.01	PTPLAD2	426.806886
98	4.2580166	0.01	KRT34	425.8016641
99	4.2493867	0.01	EDA2R	424.9386707
100	4.2400712	0.01	BST2	424.0071214
101	4.1728993	0.01	HOXC12	417.2899297
102	4.1328171	0.01	ATP6V0D2	413.2817092
103	931.13473	2.2783374	COL1A1	408.6904486
104	4.0270435	0.01	ALOX5AP	402.7043482
105	4.0076827	0.01	SULT1C4	400.7682696
106	4.0076827	0.01	SNORD13	400.7682696
107	3.932066	0.01	ETV7	393.2066041
108	3.924661	0.01	LOC729444	392.466102
109	3.8698669	0.01	CARD11	386.9866922
110	3.8093272	0.01	FAM26E	380.9327197
111	3.7585102	0.01	FMO3	375.8510207
112	3.7088047	0.01	HCG26	370.8804664
113	3.6884867	0.01	SAA1	368.8486729
114	3.6116073	0.01	C15orf56	361.1607263
115	3.5626069	0.01	PRKCB	356.2606878
116	3.473325	0.01	MIR3648	347.3325003
117	3.4675458	0.01	FMOD	346.754576
118	3.4426348	0.01	LOC100506013	344.2634847
119	3.3326146	0.01	PINLYP	333.2614608
120	3.2061462	0.01	RNU4ATAC	320.6146157
121	3.1893914	0.01	TGM3	318.939138
122	3.1575682	0.01	SNORA20	315.7568184
123	3.1537455	0.01	CX3CL1	315.3745463
124	3.1352137	0.01	METTL7A	313.5213719
125	3.0945138	0.01	TNFSF10	309.4513827
126	3.0630898	0.01	SNTG2	306.3089766
127	3.0369776	0.01	INHBC	303.6977641

128	23.02453	0.0759984	FAM19A5	302.9605847
129	3.0202826	0.01	LOC645638	302.0282611
130	2.9928878	0.01	CFB	299.2887758
131	2.9884623	0.01	PODN	298.846225
132	2.9848887	0.01	FUOM	298.4888674
133	2.9569074	0.01	ENPEP	295.6907377
134	2.9428395	0.01	EFCC1	294.2839467
135	2.9397047	0.01	ITGA8	293.9704694
136	2.926046	0.01	BEX4	292.6046024
137	2.9128266	0.01	HSPB3	291.2826559
138	11.789865	0.0405774	LYNX1	290.5526434
139	2.9025	0.01	DUSP23	290.2500002
140	2.8994713	0.01	AKR1E2	289.9471307
141	2.8901367	0.01	CASP1	289.0136704
142	9.8191294	0.0342827	TRPM2	286.4166628
143	2.8053779	0.01	F10	280.5377887
144	2.7193292	0.01	KRT76	271.9329184
145	2.706487	0.01	SNORA62	270.6487015
146	2.6940719	0.01	GPC3	269.4071937
147	2.6717885	0.01	SNORD84	267.1788464
148	56.393021	0.2111186	VASN	267.1154185
149	2.6497076	0.01	KCNMB1	264.9707567
150	2.6379684	0.01	MIR4517	263.7968357
151	34.836113	0.1326409	APOL1	262.6347723
152	10.922949	0.0417543	PCDHB16	261.6007782
153	2.6049938	0.01	MIR1909	260.4993752
154	2.5728333	0.01	SNORD79	257.2833335
155	2.5456907	0.01	GPR183	254.569073
156	2.5444125	0.01	KRT16	254.4412502
157	2.516634	0.01	NLGN4Y	251.6633993
158	2.4848907	0.01	EDN2	248.4890702
159	2.4809464	0.01	MIR26A2	248.0946431
160	2.4809464	0.01	SNORD45A	248.0946431
161	2.4735846	0.01	GMFG	247.3584572
162	2.4336758	0.01	CDO1	243.3675786
163	2.42325	0.01	MIR1273D	242.3250002
164	2.4158817	0.01	TTTY15	241.5881701
165	2.3998698	0.01	TSPAN2	239.986975
166	2.3681761	0.01	VTRNA1-2	236.8176138
167	41.223857	0.1740983	CLIP3	236.7848934
168	2.3415674	0.01	MIR4754	234.1567418
169	2.3120469	0.01	MOV10L1	231.2046901
170	2.300514	0.01	LOC339535	230.0513963

171	10.79969	0.0476923	SLC14A1	226.4449434
172	2.2604869	0.01	SLC44A3	226.048686
173	2.2454783	0.01	SLCO2B1	224.5478338
174	2.2408548	0.01	SNORD83B	224.0854841
175	2.222928	0.01	CPXM2	222.2928002
176	2.217016	0.01	AQP7P3	221.7015959
177	58.060647	0.2640365	MXRA8	219.8963058
178	2.1936789	0.01	SNORD83A	219.3678949
179	2.1708281	0.01	SNORD119	217.0828127
180	2.1484485	0.01	MIR570	214.8448455
181	2.1265255	0.01	MIR635	212.6525512
182	2.1265255	0.01	MIR639	212.6525512
183	2.1050455	0.01	KRTAP20-2	210.5045456
184	2.078798	0.01	SLC16A14	207.8798007
185	14.332442	0.0700062	OLFM1	204.7310436
186	2.0402748	0.01	KCNE1	204.0274827
187	425.22126	2.0860053	DTX3	203.8447618
188	2.030253	0.01	LYPD3	203.025301
189	2.0298653	0.01	C9orf53	202.9865261
190	40.395529	0.2003458	UBD	201.6290574
191	2.0038413	0.01	-	200.3841348
192	2.0029243	0.01	AK8	200.2924258
193	1.9968013	0.01	GALNT5	199.6801343
194	1.9942536	0.01	C8orf48	199.425359
195	1.9614071	0.01	HIST1H2BH	196.140706
196	1.9591022	0.01	CCL20	195.9102234
197	1.9493151	0.01	SERPINA5	194.9315053
198	1.9367983	0.01	LIPC	193.6798329
199	1.8991893	0.01	PRR26	189.9189276
200	1.8945409	0.01	MIR2682	189.4540911
201	1.8945409	0.01	SNORD23	189.4540911
202	1.8945409	0.01	MIR7-1	189.4540911
203	1.8945409	0.01	MIR5006	189.4540911
204	1.8682922	0.01	CXCL10	186.8292178
205	96.575378	0.5174528	RARRES3	186.6361278
206	1.8654687	0.01	PPP1R14A	186.5468672
207	1.8607098	0.01	SNORD11B	186.0709823
208	1.8573931	0.01	SERPINB7	185.739305
209	1.8538132	0.01	NTF3	185.3813197
210	1.8161176	0.01	LINC00515	181.6117649
211	1.8151761	0.01	GJC2	181.517607
212	21.069359	0.1167716	CLDN1	180.4321569
213	1.802524	0.01	HEPH	180.2523954

214	1.7811923	0.01	RNU5A-1	178.1192309
215	1.7811923	0.01	RNU5B-1	178.1192309
216	1.770787	0.01	SOSTDC1	177.0787043
217	10.324152	0.0597887	MMP19	172.6771942
218	1.7132993	0.01	ZIK1	171.3299329
219	1.7081926	0.01	ARL17B	170.8192624
220	1.6655305	0.01	IGSF23	166.5530471
221	1.6435292	0.01	NPB	164.3529181
222	1.6393274	0.01	VNN2	163.9327435
223	24.805702	0.1528035	BDKRB1	162.3372417
224	1.5928624	0.01	HIST1H1E	159.2862422
225	34.403995	0.2162917	NES	159.0629232
226	1.5669135	0.01	SNORA66	156.6913535
227	1.5552202	0.01	SNORA5A	155.5220151
228	1.55088	0.01	LINC00592	155.0880001
229	1.5437	0.01	SNORA63	154.3700001
230	1.5437	0.01	RNU11	154.3700001
231	3.7816984	0.0245461	GAS7	154.0652807
232	44.449105	0.2887505	COL16A1	153.9360309
233	1.5351713	0.01	C1orf54	153.5171272
234	1.5347893	0.01	USP9Y	153.4789313
235	1.5291368	0.01	FTH1P3	152.9136794
236	1.5267363	0.01	DNASE1L2	152.6736265
237	1.5101413	0.01	SNORA71A	151.0141306
238	17.908374	0.1186273	SARDH	150.9632945
239	1.5079559	0.01	CRYAB	150.7955862
240	1.5054659	0.01	FAM167B	150.5465946
241	1.5046895	0.01	GLP2R	150.4689532
242	1.499277	0.01	SCARA5	149.927698
243	1.499277	0.01	SNORA7B	149.927698
244	1.4885679	0.01	TMEM52B	148.8567858
245	1.4885679	0.01	SNORA29	148.8567858
246	1.4809282	0.01	OLR1	148.092815
247	48.873965	0.3313115	CTSK	147.5166438
248	96.342064	0.6608126	GDF15	145.7933187
249	1.4393059	0.01	BCAS1	143.93059
250	40.583061	0.2828742	C1S	143.4668299
251	1.4081047	0.01	MIR3689B	140.8104731
252	1.4068373	0.01	ZNF284	140.683731
253	1.4055147	0.01	LOC100233156	140.5514716
254	1.3699228	0.01	TNNT3	136.9922762
255	1.3695476	0.01	PSORS1C1	136.9547646
256	350.61651	2.5781983	ACTA2	135.9928428

257	1.3514883	0.01	SPTLC3	135.1488328
258	1.3347782	0.01	LINC00654	133.4778197
259	1.3316262	0.01	TNN	133.1626199
260	113.61894	0.8540163	NPPB	133.0407116
261	1.3106887	0.01	ZNF285	131.068868
262	1.2841519	0.01	GSTT2	128.415185
263	9.2921748	0.0724732	ASB2	128.2154011
264	51.732358	0.40621	ITGB2	127.3537382
265	16.778796	0.1324665	SH3RF2	126.6644084
266	1.2645601	0.01	GYG2P1	126.4560074
267	1.2554187	0.01	LOC100505679	125.5418676
268	1.2502047	0.01	LOC344887	125.0204662
269	55.124041	0.4442642	C11orf96	124.0794203
270	1.2263769	0.01	INPP5D	122.6376929
271	1.2085023	0.01	C1orf110	120.8502256
272	95.537063	0.7978933	C1R	119.7366406
273	1.1940033	0.01	TMEM100	119.4003306
274	1.193615	0.01	MAP9	119.3615029
275	1.1840881	0.01	PLCE1-AS1	118.4088069
276	1.171097	0.01	ZFP112	117.1097004
277	1.1707837	0.01	SNORA81	117.0783709
278	1.1688138	0.01	GYPE	116.8813798
279	61.083869	0.5287194	PDGFRB	115.5317269
280	1.1387951	0.01	KEL	113.8795083
281	1.1318371	0.01	MGP	113.1837068
282	1.1241208	0.01	ZNF300	112.4120768
283	1.122284	0.01	ZNF223	112.2283969
284	41.448023	0.3737559	FGF19	110.8959819
285	1.0957581	0.01	LPAR6	109.5758134
286	1.0856599	0.01	AMZ1	108.565991
287	1.0788885	0.01	CLIC5	107.8888528
288	1.0748398	0.01	CCRL2	107.4839829
289	22.883954	0.2148698	NUPR1	106.5015024
290	1.0571947	0.01	KCNK7	105.7194674
291	1.0458322	0.01	SEMA3B	104.5832219
292	1.0274421	0.01	RCVRN	102.7442072
293	1.0262499	0.01	IFI44L	102.6249874
294	1.0198997	0.01	LOC654342	101.9899675
295	2.0901802	0.0206271	SCN9A	101.3315283
296	545.54992	5.3838124	B4GALNT1	101.3315265
297	1.0089765	0.01	P4HA3	100.8976453
298	0.9950863	0.01	THEGL	99.508629
299	0.9866362	0.01	BPI	98.6636245

300	21.294125	0.2160213	TES	98.57420625
301	0.9853404	0.01	NANOS3	98.5340426
302	0.9845646	0.01	C10orf107	98.4564568
303	0.9830165	0.01	ST7-AS1	98.301651
304	0.9816935	0.01	LRRC29	98.1693473
305	0.9702025	0.01	IGLL5	97.0202515
306	34.486038	0.3586261	CCL26	96.16155056
307	0.960999	0.01	MBLAC1	96.0999013
308	7.0513991	0.0741257	CYS1	95.12755592
309	0.9405017	0.01	GRIP2	94.0501693
310	0.9277663	0.01	NYAP1	92.7766278
311	0.9172513	0.01	NTM	91.7251321
312	0.9160418	0.01	ACCSL	91.6041759
313	0.9055223	0.01	AGXT	90.5522347
314	0.9021623	0.01	HIST1H3E	90.2162338
315	11.041874	0.1227453	DIRAS3	89.9575799
316	0.8964902	0.01	STYK1	89.6490239
317	0.8950587	0.01	ZNF93	89.5058698
318	0.8868064	0.01	HIST1H2AL	88.6806384
319	0.8761541	0.01	CYP3A5	87.6154055
320	0.8717986	0.01	ZFY	87.1798632
321	0.8632954	0.01	PCDHB4	86.3295361
322	0.8584943	0.01	HRCT1	85.8494336
323	0.8523497	0.01	CCIN	85.2349694
324	0.8518912	0.01	TXLNG2P	85.1891244
325	0.8514791	0.01	TMEM249	85.1479061
326	0.8471524	0.01	LOC101059948	84.715244
327	0.8455243	0.01	ESPNL	84.5524344
328	0.8417752	0.01	DDO	84.1775246
329	0.8395951	0.01	MYOCD	83.9595109
330	0.8359865	0.01	HTR3C	83.5986534
331	0.8322664	0.01	GPR15	83.2266374
332	0.8321714	0.01	MAGEA3	83.2171421
333	10.108457	0.12297	ENPP2	82.20261623
334	0.8182403	0.01	ZNF808	81.824026
335	0.8083766	0.01	LOC201651	80.8376649
336	4.0975954	0.0508061	LMOD1	80.65162349
337	0.8056682	0.01	GRAP2	80.5668171
338	0.8002175	0.01	LGALS12	80.0217499
339	0.7994866	0.01	FLJ27352	79.9486574
340	0.7964312	0.01	HRASLS2	79.6431211
341	0.789392	0.01	DEFB109P1B	78.9392046
342	0.788894	0.01	SNX29P2	78.8894007

343	135.94127	1.7466069	LCP1	77.83163631
344	0.7747193	0.01	CCR1	77.4719332
345	0.7674443	0.01	PTGFR	76.7444302
346	0.7656628	0.01	CCDC36	76.5662826
347	0.7649693	0.01	ADHFE1	76.4969324
348	0.7623393	0.01	WISP1	76.2339334
349	0.7578164	0.01	LINC00483	75.7816364
350	0.7543873	0.01	MTNR1A	75.4387331
351	0.7518019	0.01	FLJ22447	75.1801949
352	12.972899	0.1726566	DYSF	75.13698208
353	0.7481893	0.01	AKR1C2	74.8189313
354	0.7441658	0.01	PRUNE2	74.4165808
355	0.7411078	0.01	DBH	74.1107753
356	0.7408598	0.01	TDRD9	74.0859787
357	0.736394	0.01	CD74	73.6393994
358	92.821382	1.2621392	CPZ	73.54290637
359	0.7310553	0.01	CDH3	73.1055309
360	0.7286696	0.01	C2orf15	72.8669581
361	0.7286696	0.01	CYP39A1	72.8669581
362	0.7241122	0.01	TNFAIP6	72.4112231
363	0.7223553	0.01	LOC100130880	72.2355287
364	0.7196115	0.01	PRKY	71.9611534
365	1.0210584	0.0143114	DMD	71.345669
366	52.503421	0.7394744	INHBE	71.00100155
367	0.7040524	0.01	NPFF	70.4052365
368	0.698741	0.01	KRTAP1-5	69.874099
369	13.802196	0.1982438	GBP1	69.6223414
370	0.6954377	0.01	LOC285441	69.5437709
371	0.6935092	0.01	C1orf94	69.3509152
372	0.6912854	0.01	GPR65	69.1285383
373	202.56176	2.9389172	ALPL	68.92394111
374	0.6839177	0.01	ZNF846	68.3917722
375	5.0363848	0.0738	F2RL3	68.24368137
376	26.923205	0.3951144	C3	68.14028155
377	0.6810441	0.01	P2RX6	68.1044118
378	20.572973	0.3037647	CTSW	67.72668349
379	0.6770614	0.01	PCDHGA10	67.7061404
380	0.6733425	0.01	LOC643733	67.3342488
381	5.8237178	0.08665	DSC3	67.20968617
382	14.032425	0.2087858	SYTL3	67.20968605
383	2.5169022	0.0374485	FSTL4	67.20968549
384	0.6710196	0.01	FAM218A	67.101955
385	0.6686615	0.01	LOC646736	66.8661498

386	0.6665229	0.01	ZNF542	66.6522922
387	0.6660259	0.01	CAPN12	66.6025887
388	0.6631647	0.01	PPP5D1	66.3164678
389	0.6609862	0.01	BMP5	66.0986181
390	11.482315	0.1748788	DCN	65.65869324
391	0.6546581	0.01	LRRIQ3	65.4658116
392	0.6520635	0.01	FLJ26245	65.2063517
393	0.6512484	0.01	LOC100289361	65.1248438
394	0.6497256	0.01	ZNF439	64.9725644
395	0.6492196	0.01	ARL14EPL	64.9219627
396	0.6492196	0.01	LOC100133445	64.9219627
397	0.6472034	0.01	CXCL11	64.7203417
398	0.6472034	0.01	LINC00854	64.7203417
399	0.6462	0.01	STARD13-AS	64.6200001
400	0.6402442	0.01	LOC650293	64.024424
401	2.9577407	0.0468934	PTCH2	63.07370561
402	0.6267654	0.01	LAG3	62.6765414
403	0.6225527	0.01	MGARP	62.2552652
404	390.71474	6.2845398	LGALS3BP	62.17077992
405	4.1349107	0.0666494	FIBIN	62.03971044
406	0.6172514	0.01	C8orf34	61.7251389
407	0.6161109	0.01	ASB15	61.6110865
408	8.8035288	0.143094	CPA4	61.5227126
409	0.615151	0.01	SOX8	61.5151034
410	0.6138424	0.01	ANKRD7	61.3842416
411	0.6087619	0.01	MDS2	60.8761928
412	0.6040565	0.01	ZNF154	60.4056522
413	44.934651	0.747369	COL7A1	60.1237779
414	0.5898099	0.01	HCG4B	58.9809906
415	0.5868906	0.01	DIRC3	58.6890553
416	0.5853919	0.01	HIST1H4J	58.5391854
417	0.5852654	0.01	FILIP1	58.5265393
418	64.429941	1.102861	KISS1	58.420727
419	0.5828192	0.01	MAG	58.2819217
420	10.513849	0.1815746	UBA7	57.90372947
421	0.5738159	0.01	ZNF528	57.3815895
422	0.5735108	0.01	WNT2	57.3510836
423	6.9327289	0.1211711	NEDD9	57.21439951
424	7.4139538	0.1303673	SOD3	56.86973421
425	0.5683623	0.01	BEX2	56.8362273
426	0.5675024	0.01	KRT77	56.7502421
427	41.203988	0.726735	STOM	56.69740172
428	0.5647683	0.01	TMEM220-AS1	56.4768293

429	0.5579639	0.01	CD38	55.7963856
430	0.5563678	0.01	MAGIX	55.6367849
431	0.555732	0.01	LOC100507377	55.5732
432	0.5554358	0.01	LOC100289230	55.5435768
433	0.554254	0.01	RAB33A	55.425399
434	21.27743	0.3846333	MAP1LC3A	55.31874152
435	0.5527838	0.01	C4BPB	55.278382
436	0.5527838	0.01	HIST1H4E	55.278382
437	0.550905	0.01	ZCCHC5	55.0905023
438	0.5505931	0.01	PMCH	55.0593131
439	0.5498668	0.01	LOC100652791	54.9866755
440	0.5472197	0.01	ZNF880	54.7219694
441	0.5465022	0.01	RAET1G	54.6502186
442	6.8168061	0.125575	SYN1	54.28474619
443	0.5417195	0.01	PADI1	54.1719522
444	0.5395139	0.01	FLG	53.9513886
445	0.5391966	0.01	C17orf82	53.9196637
446	0.538732	0.01	ZNF433	53.8732013
447	0.5362828	0.01	FAM57B	53.6282811
448	0.5349063	0.01	WFIKKN1	53.4906315
449	0.5327186	0.01	NPY4R	53.2718559
450	24.815201	0.4660066	GBP2	53.25075118
451	0.5316314	0.01	HERC2P4	53.1631378
452	2411.1861	45.46192	DCTN2	53.03748967
453	0.5292686	0.01	C16orf93	52.9268572
454	2.5227568	0.0478395	MAPK10	52.73375357
455	1.1836925	0.0224466	SOX6	52.73375264
456	0.5262614	0.01	MPZ	52.6261364
457	0.5249358	0.01	MIR497HG	52.4935769
458	0.5178914	0.01	SEC16B	51.7891402
459	0.5135522	0.01	CLDN7	51.3552243
460	0.5114098	0.01	FLJ13224	51.1409816
461	0.5112007	0.01	MAMDC4	51.1200736
462	0.5076724	0.01	VMO1	50.7672351
463	255.95133	5.0517611	IL32	50.66576326
464	41.203398	0.8132395	EDIL3	50.66576325
465	5.0253816	0.0991869	CTSF	50.66576308
466	3.2781944	0.0647024	IQUB	50.66576297
467	0.5027732	0.01	SLAMF8	50.2773221
468	0.4988618	0.01	ZNF69	49.886176
469	11.455111	0.230802	SYNC	49.63176805
470	0.4961893	0.01	SPANXA2	49.6189286
471	0.4955993	0.01	SAP25	49.5599287

472	0.4954029	0.01	LOC100133315	49.5402932
473	16.04629	0.3260237	COL8A1	49.21817004
474	0.4921716	0.01	STBD1	49.2171559
475	0.4907367	0.01	ZNF600	49.0736657
476	11.931655	0.2444782	MATN2	48.80457197
477	7.5899082	0.1561781	GPR1	48.59777293
478	2.8597888	0.0588461	PLAGL1	48.59777273
479	0.4855534	0.01	FAM65C	48.5553355
480	0.4855534	0.01	HERC2P3	48.5553355
481	0.4836658	0.01	PABPC5	48.3665797
482	0.4835255	0.01	ZFHX4-AS1	48.3525522
483	0.4835255	0.01	HIST1H2BB	48.3525522
484	0.4815702	0.01	LOC100130000	48.1570191
485	1.9478546	0.0405121	SYNPO2	48.08077544
486	0.4801832	0.01	FAM222A-AS1	48.018318
487	41.359011	0.8620524	JAG2	47.97737582
488	97.393436	2.0358368	CALB2	47.8395098
489	0.4757979	0.01	ZCWPW2	47.5797946
490	23.313174	0.4901455	HSPB8	47.56377772
491	0.4741741	0.01	PTPRR	47.4174062
492	0.4725612	0.01	SCGB2B2	47.2561225
493	0.4725612	0.01	LOC100130348	47.2561225
494	7.1889138	0.1528035	SELPLG	47.04678024
495	0.4704278	0.01	CLEC3B	47.0427766
496	1983.1729	42.501376	OS9	46.66138196
497	0.4652719	0.01	TP63	46.527187
498	0.460043	0.01	RTP1	46.0043047
499	86.307724	1.895176	SLC26A10	45.54074373
500	2.8744759	0.0631811	KCNE4	45.49578763
501	3.0483969	0.0670039	KLHDC7B	45.49578711
502	0.4540294	0.01	LINC00173	45.4029412
503	63.782276	1.4062964	ESRG	45.35478807
504	18.652667	0.4113221	CACNG4	45.34807381
505	0.453324	0.01	TMPRSS3	45.3323959
506	0.4528455	0.01	PCDHGA1	45.2845502
507	0.4525505	0.01	SPATA3	45.2550489
508	394.7517	8.7997381	DDIT3	44.8594827
509	0.4466615	0.01	GUCY1B3	44.6661513
510	0.4462516	0.01	HIST1H3G	44.6251606
511	0.4443486	0.01	HIST1H2AI	44.4348614
512	0.4443486	0.01	SPANXE	44.4348614
513	0.4424618	0.01	PSPN	44.2461784
514	41.518036	0.9392521	SAMD11	44.20329347

515	2.7497305	0.062328	MEG3	44.11712731
516	0.4403249	0.01	SEMA4A	44.0324932
517	0.4402806	0.01	TOB1-AS1	44.0280634
518	76.849217	1.7457672	MAP1A	44.02031115
519	0.4378141	0.01	LINC00340	43.7814076
520	0.4367436	0.01	LOC283335	43.6743626
521	0.435072	0.01	PCDHGA2	43.5072025
522	0.434709	0.01	CORO6	43.4709012
523	0.7411329	0.0170659	IGFN1	43.42779817
524	0.4326334	0.01	TEK	43.2633382
525	3047.4135	70.503429	HLA-B	43.2236205
526	0.4296897	0.01	SLC51A	42.9689691
527	0.4290999	0.01	TXK	42.9099863
528	0.4279251	0.01	C4orf47	42.7925052
529	0.4270482	0.01	KLHL35	42.7048156
530	0.4261748	0.01	USP30-AS1	42.6174847
531	0.424547	0.01	HAL	42.4546983
532	0.4244389	0.01	ZNF726	42.4438901
533	0.4235762	0.01	SEC24B-AS1	42.357622
534	0.422717	0.01	HSD11B1	42.2717039
535	0.4220322	0.01	MIR137HG	42.20322
536	0.4201603	0.01	C8orf56	42.0160283
537	0.4193149	0.01	TNFRSF17	41.931489
538	0.4184729	0.01	MEG8	41.8472892
539	0.4173207	0.01	SIM1	41.7320651
540	0.4172998	0.01	SH3GL1P1	41.729976
541	0.416799	0.01	BZRAP1-AS1	41.6799
542	0.4163826	0.01	PMF1-BGLAP	41.6382618
543	0.4151384	0.01	B3GNT6	41.5138447
544	0.4138195	0.01	LYPD5	41.38195
545	0.4114501	0.01	LOC100128993	41.1450148
546	6.4104315	0.1559666	RHBDD1	41.10130806
547	47.120286	1.150059	ID2	40.97205856
548	4.6414143	0.1132928	IL34	40.96831645
549	56.113632	1.374537	EMILIN1	40.8236611
550	0.4071893	0.01	AOAH	40.7189332
551	0.4062368	0.01	ZFP28	40.6236842
552	0.4046592	0.01	UPK1B	40.4659224
553	0.403454	0.01	MYO7B	40.3453984
554	2.2420912	0.0555994	MTL5	40.32581165
555	2.340219	0.0580328	FOXE1	40.32581132
556	0.4030938	0.01	SCGB3A1	40.3093811
557	0.4023156	0.01	AHSP	40.2315637

558	0.4012827	0.01	CP	40.1282735
559	0.4007683	0.01	LOC729059	40.076827
560	5.928046	0.1479522	JAK3	40.06731268
561	0.3995389	0.01	KCNIP2	39.9538919
562	0.3984694	0.01	LHB	39.8469408
563	0.3982156	0.01	LOC285084	39.8215605
564	2.8426504	0.0714076	ZNF608	39.8088141
565	0.3965737	0.01	LOC339240	39.657374
566	0.3940989	0.01	GCSAML	39.4098904
567	1.6505171	0.0420066	RASGRP3	39.29181602
568	0.3924661	0.01	LOC100506136	39.2466102
569	0.3917284	0.01	NLGN4Y-AS1	39.1728384
570	0.3917284	0.01	FITM1	39.1728384
571	0.3914831	0.01	SPEF1	39.1483094
572	0.3884427	0.01	GRIK1-AS2	38.8442684
573	0.388081	0.01	C1orf227	38.8081006
574	0.3873597	0.01	KANSL1-AS1	38.7359666
575	0.386641	0.01	COL4A2-AS1	38.6641002
576	10.751149	0.2785092	IL20RB	38.60248636
577	16.683877	0.432197	BDKRB2	38.60248629
578	0.385925	0.01	LOC654433	38.5925
579	0.3841465	0.01	RNF5P1	38.4146544
580	0.3837928	0.01	LGALS2	38.3792818
581	82.3905	2.1535596	HLA-F	38.25782123
582	0.3823844	0.01	TMEM244	38.2384404
583	0.3814512	0.01	KCNRG	38.145119
584	0.381285	0.01	NCALD	38.1285024
585	0.3797713	0.01	PMEL	37.9771299
586	0.3795984	0.01	LOC441455	37.9598361
587	0.3788022	0.01	MYT1L	37.8802223
588	0.378564	0.01	CDRT15P1	37.8564033
589	0.3778395	0.01	ZNF229	37.7839545
590	0.3752242	0.01	TGM5	37.5224163
591	0.373699	0.01	FBXO39	37.3699044
592	2.3522126	0.063191	DIO2	37.22382598
593	1.631662	0.0438338	CD163L1	37.22382568
594	0.372142	0.01	CYP2D7P1	37.2141965
595	0.3679803	0.01	CPA3	36.7980283
596	0.3675476	0.01	HSD17B3	36.7547619
597	0.3669005	0.01	ZNF724P	36.6900528
598	0.3669005	0.01	ZNF610	36.6900528
599	0.365186	0.01	CHAD	36.518604
600	0.3634452	0.01	TLR1	36.3445239

601	3.4429939	0.0951371	ADAMTS10	36.18983096
602	0.3615093	0.01	MXRA5	36.1509337
603	0.3613864	0.01	KIAA1257	36.1386416
604	0.3602411	0.01	C8orf46	36.0241141
605	0.3600337	0.01	SOWAHA	36.0033689
606	183.19225	5.1024771	LOC100506844	35.90261002
607	0.3583826	0.01	CCDC70	35.8382631
608	0.3580747	0.01	S100Z	35.8074743
609	0.3562385	0.01	MB	35.6238462
610	0.3556305	0.01	MORN3	35.5630546
611	0.355176	0.01	SGIP1	35.517597
612	18.202768	0.515248	PLCB2	35.32816828
613	0.3529204	0.01	GRIK2	35.2920407
614	0.3520262	0.01	ARMC12	35.2026183
615	0.3502513	0.01	KRT78	35.0251261
616	0.3502513	0.01	LINC00331	35.0251261
617	0.3493705	0.01	CEL	34.9370495
618	0.3490779	0.01	CLEC1A	34.907789
619	0.3484941	0.01	SLPI	34.8494147
620	0.3470433	0.01	C6orf58	34.7043298
621	0.3470433	0.01	SLC35G5	34.7043298
622	0.3461786	0.01	VPREB3	34.6178572
623	0.3451593	0.01	KLF8	34.5159275
624	0.3436667	0.01	RAB7B	34.3666722
625	0.343327	0.01	S100A1	34.3327018
626	0.3425745	0.01	LOC388942	34.2574521
627	0.3421995	0.01	LOC100506368	34.2199508
628	0.3405221	0.01	LOC100129138	34.0522059
629	0.3383109	0.01	HSPB9	33.8310877
630	0.3377626	0.01	GBP1P1	33.7762561
631	0.3365806	0.01	PCDHB7	33.6580619
632	1.8182507	0.0541068	MEGF6	33.60484274
633	0.335317	0.01	SPATA9	33.5316975
634	0.3345096	0.01	SPANXN3	33.4509631
635	23.147513	0.6952323	ALDH3B1	33.29464441
636	0.3329066	0.01	IFLTD1	33.290655
637	0.3318463	0.01	SLIT2-IT1	33.1846338
638	0.3313188	0.01	BANF2	33.131876
639	0.3313188	0.01	CETN4P	33.131876
640	0.8594901	0.025976	PTGER3	33.08784556
641	837.19701	25.359227	MYL9	33.01350587
642	0.3292251	0.01	LOC388849	32.9225119
643	0.3292251	0.01	NRN1L	32.9225119

644	117.69546	3.582436	COL5A1	32.85347316
645	0.3276722	0.01	MGC39372	32.767217
646	185.20373	5.6783915	UBE3B	32.61552661
647	0.3256242	0.01	MAGOH2	32.5624219
648	0.3252854	0.01	VNN1	32.528538
649	0.3251162	0.01	RPL23AP64	32.5116225
650	0.324231	0.01	TEX22	32.4231039
651	212.45072	6.5684547	TSPAN31	32.34409448
652	0.3228497	0.01	LGALS4	32.2849729
653	0.3222257	0.01	AGT	32.2225744
654	0.3221012	0.01	CPSF4L	32.2101237
655	0.3211086	0.01	CIDEC	32.1108629
656	0.3208614	0.01	STEAP1B	32.0861432
657	1.4570105	0.0454551	CCDC120	32.05385026
658	13.532435	0.4221781	C10orf54	32.0538502
659	2.7738877	0.0865384	ME3	32.05385018
660	0.3176822	0.01	NME1-NME2	31.7682165
661	0.3148029	0.01	PATE4	31.480287
662	0.3143281	0.01	MEG9	31.4328055
663	0.3143281	0.01	GUSBP5	31.4328055
664	10.61834	0.3385462	ANGPTL2	31.36452008
665	0.3136185	0.01	FAM71E1	31.361851
666	620.94545	19.800856	MBD6	31.35952496
667	0.310928	0.01	GRPR	31.0928012
668	5.2938061	0.1706586	LOC100505817	31.01985512
669	3.812186	0.122895	SOX30	31.01985499
670	0.3078279	0.01	NLRC4	30.7827918
671	0.3072229	0.01	ZNF525	30.722285
672	0.3067706	0.01	HGFAC	30.6770609
673	0.3064699	0.01	ZNF501	30.6469853
674	0.3053473	0.01	LILRA5	30.5347253
675	20.206924	0.6634824	CPM	30.45585768
676	0.3027596	0.01	MATN1-AS1	30.2759564
677	0.3023569	0.01	ADAP2	30.235691
678	16.077756	0.5321005	MX1	30.21563657
679	162.45628	5.3780239	TAGLN	30.20743028
680	0.3012424	0.01	PLEKHS1	30.1242411
681	0.3002875	0.01	CST2	30.0287464
682	8.7041558	0.2902753	PYROXD2	29.98585988
683	8.0473842	0.2683726	TP53TG1	29.98585985
684	422.9303	14.106477	PIP4K2C	29.98128468
685	54.362687	1.8147846	FOSB	29.95544826
686	0.2985666	0.01	CHRNA1	29.8566619

687	18.067145	0.6067063	CD14	29.77906086
688	0.2972889	0.01	TMEM239	29.7288873
689	34.156765	1.1499099	CPQ	29.70386121
690	80.926709	2.728228	XRCC6BP1	29.6627364
691	0.2964431	0.01	FAM27A	29.6443101
692	54.496892	1.8409309	LBH	29.60289871
693	0.2935204	0.01	S100A5	29.3520423
694	0.2931076	0.01	ZNF630	29.3107595
695	0.2920124	0.01	CFL1P1	29.2012378
696	12.464566	0.4277496	ABCC3	29.13986387
697	1.9360272	0.0668706	ZNF558	28.95186478
698	8.3568722	0.2886471	PCDHB14	28.95186469
699	4.7986727	0.1657466	SOCS1	28.95186469
700	0.2894438	0.01	XAGE2	28.944375
701	0.2874476	0.01	AMBP	28.7447586
702	0.2867883	0.01	ZNF280A	28.6788303
703	0.2866568	0.01	LOC158696	28.6656809
704	106.7495	3.7262788	HLA-H	28.6477485
705	0.2854788	0.01	RAX2	28.5478767
706	11.417571	0.4008908	DYX1C1	28.4805014
707	0.283344	0.01	FAM170A	28.3343984
708	0.283344	0.01	TEKT4	28.3343984
709	19.052445	0.6728613	C16orf62	28.31556002
710	0.2827673	0.01	CACNA1C-AS4	28.27673
711	0.2823841	0.01	PPIAL4G	28.2384147
712	0.2819067	0.01	ZNF80	28.1906662
713	111.39219	3.9748415	BCAM	28.02431022
714	2.9444199	0.1054672	OSR1	27.91786945
715	0.2791688	0.01	REN	27.9168788
716	0.2789819	0.01	LOC100289473	27.8981928
717	0.277127	0.01	LOC730102	27.7126995
718	0.277127	0.01	TUBB8	27.7126995
719	3.28613	0.1185855	MAGI2-AS3	27.71107042
720	3.2961931	0.1191709	ITIH5	27.65937075
721	0.2761303	0.01	CCDC170	27.6130324
722	0.2756607	0.01	LINC00597	27.5660715
723	0.2751149	0.01	FLJ12334	27.5114852
724	0.2743302	0.01	SLC5A2	27.4330189
725	0.2734902	0.01	SPRR2E	27.3490158
726	8.1742716	0.3007536	GCNT2	27.17930155
727	0.2717073	0.01	CD200	27.1707301
728	1.5263062	0.056774	SOX2-OT	26.88387459
729	1.4871379	0.0553171	KIF26B	26.88387446

730	1.2091915	0.0449783	CD96	26.88387434
731	3.2925179	0.1224719	MTAP	26.88387431
732	284.3385	10.626936	DKK3	26.75639554
733	0.2664955	0.01	LOC440356	26.6495525
734	7.2103287	0.2708066	GRAMD3	26.62537561
735	0.2661552	0.01	ZRANB2-AS2	26.6155173
736	0.2659288	0.01	ZNF253	26.5928754
737	0.2655616	0.01	SYCP2L	26.5561644
738	11.756163	0.4436937	NTN4	26.49612617
739	45.57387	1.7202469	ABLIM3	26.49263296
740	0.2637968	0.01	CST5	26.3796836
741	0.2637968	0.01	HIST1H1B	26.3796836
742	4.096502	0.1553655	SEMA3C	26.36687683
743	1.897924	0.0719814	EGF	26.36687678
744	13.479457	0.5145902	PARP10	26.19454427
745	0.2614799	0.01	SMIM18	26.1479925
746	206.86551	7.9572557	MDM2	25.99709209
747	1717.3768	66.197332	MARS	25.9432931
748	0.2593647	0.01	SOWAHD	25.9364655
749	0.2593647	0.01	TRIM61	25.9364655
750	0.258801	0.01	LINC00514	25.8800994
751	0.2587742	0.01	TACR2	25.8774214
752	3.5298018	0.13655	RRAD	25.8498792
753	0.2582931	0.01	SLC6A15	25.8293121
754	0.2563339	0.01	RNF112	25.6333949
755	0.2563339	0.01	CLIC3	25.6333949
756	0.2563339	0.01	CCL3	25.6333949
757	0.2563339	0.01	UPK1A-AS1	25.6333949
758	0.2548983	0.01	EPPK1	25.489832
759	0.2547671	0.01	NMU	25.4767115
760	0.2538362	0.01	LOC643723	25.3836176
761	127.97525	5.0445619	SOX18	25.36895122
762	0.252147	0.01	SLC9A7P1	25.2147006
763	0.2500294	0.01	CFHR3	25.0029394
764	0.2488848	0.01	C14orf105	24.8884753
765	1.0496512	0.0422976	PCDHGB7	24.81588434
766	1.8704518	0.0753732	ZNF415	24.8158842
767	3.6086494	0.1454169	FAM228B	24.81588407
768	1.5417965	0.0621294	ZNF425	24.81588392
769	0.2476034	0.01	C2orf54	24.7603367
770	0.2472117	0.01	LSMEM1	24.7211744
771	0.2469188	0.01	KRT36	24.6918839
772	0.2464322	0.01	KCNJ6	24.6432204

773	0.2463351	0.01	C4orf51	24.6335107
774	0.2448878	0.01	C9orf135	24.4887779
775	0.2444569	0.01	LOC145837	24.4456892
776	0.2443136	0.01	TAS1R3	24.4313599
777	0.2442182	0.01	C6orf25	24.4218164
778	0.2433152	0.01	SPANXA2-OT1	24.3315237
779	0.2431733	0.01	SIGLEC16	24.3173279
780	34.548063	1.4244547	PTCH1	24.2535358
781	13.866887	0.5747562	NGF	24.12655393
782	0.2412031	0.01	LINC00639	24.1203125
783	0.2406461	0.01	LOC728606	24.0646074
784	0.2403685	0.01	FAM71F1	24.0368512
785	0.2400916	0.01	SIRPD	24.009159
786	0.2398153	0.01	LOC440900	23.9815305
787	0.2398153	0.01	RDH16	23.9815305
788	7.6150185	0.3178988	SBSPON	23.95422137
789	0.2392646	0.01	PLN	23.9264639
790	0.239196	0.01	PCDHB13	23.9195983
791	0.238307	0.01	LOC285548	23.8307033
792	0.2381709	0.01	TMEM150B	23.8170857
793	0.2368176	0.01	LINC00161	23.6817614
794	0.2368176	0.01	LYG2	23.6817614
795	0.236013	0.01	P2RY6	23.6013024
796	0.235746	0.01	OCLM	23.5746041
797	0.2354797	0.01	THRB-AS1	23.5479661
798	16.547108	0.7029777	SLIT3	23.53859589
799	0.2352139	0.01	LYZL4	23.5213883
800	0.2349487	0.01	ARHGAP15	23.4948704
801	0.2346841	0.01	HSPA6	23.4684122
802	0.2346841	0.01	RPS4Y2	23.4684122
803	0.2346064	0.01	CACNA1E	23.4606417
804	142.35396	6.0768195	9-Mar	23.42573498
805	290.84326	12.437018	MMP2	23.385288
806	0.2337191	0.01	TNFSF13B	23.3719066
807	0.2330569	0.01	SPATA31E1	23.3056923
808	7.1342545	0.3066532	SLC16A4	23.26489128
809	0.2325887	0.01	C10orf11	23.2588728
810	0.232459	0.01	LINC00466	23.2459007
811	0.2324331	0.01	ECT2L	23.2433081
812	0.2307857	0.01	ZG16	23.0785714
813	0.2302757	0.01	EPHX3	23.0275691
814	90.856629	3.9533812	CREB3L1	22.98200582
815	0.2282579	0.01	MC2R	22.8257941

816	1.3884885	0.0610381	NPAS4	22.74789386
817	6.1831275	0.271811	TPTEP1	22.74789374
818	1.5578624	0.0684838	ENPP5	22.74789374
819	3.7641946	0.1654744	APOL3	22.7478937
820	0.2260298	0.01	IL36B	22.6029827
821	0.2245684	0.01	C19orf18	22.4568427
822	0.2245684	0.01	KLRC4	22.4568427
823	0.2244878	0.01	OR51E2	22.4487792
824	0.2244072	0.01	ZNF563	22.4407215
825	0.2237647	0.01	RASA4B	22.3764674
826	0.2231258	0.01	ZNF404	22.3125803
827	0.223078	0.01	SLC7A11-AS1	22.3078035
828	4.9839702	0.2241912	PRR16	22.23089609
829	0.2219377	0.01	APOC1P1	22.19377
830	0.2212309	0.01	OR2B6	22.1230892
831	245.55115	11.129893	APPL2	22.06230996
832	0.2205286	0.01	OR10A3	22.0528572
833	0.2201791	0.01	H2BFM	22.0179081
834	0.2200628	0.01	UPK2	22.006283
835	5.575687	0.2537587	VSTM4	21.9723973
836	0.2194603	0.01	ANKRD62	21.9460299
837	0.2186773	0.01	MGC45922	21.8677335
838	0.2185245	0.01	NALCN-AS1	21.8524467
839	0.2184481	0.01	C4orf22	21.8448113
840	0.2179911	0.01	KRTCAP3	21.7991109
841	40.139253	1.8438053	NOTCH3	21.76979016
842	0.2172525	0.01	NBPF16	21.7252541
843	1.2736873	0.0586577	GCNT4	21.71389857
844	1.8686548	0.086058	FZD9	21.71389848
845	0.2166315	0.01	GNG3	21.6631497
846	0.2164065	0.01	LRRC38	21.6406542
847	0.2152887	0.01	RBP5	21.528874
848	0.2151221	0.01	SELE	21.5122065
849	0.214491	0.01	FYB	21.4491046
850	0.2144028	0.01	ALDH3A1	21.4402778
851	3.5516133	0.1662022	XAF1	21.36923347
852	0.2134147	0.01	MAGEB10	21.3414747
853	1.9977506	0.0942473	TLR5	21.196901
854	35.571938	1.6781669	IL6	21.19690095
855	0.2117881	0.01	TBC1D10C	21.178811
856	125.00799	5.903075	ASB8	21.17675819
857	0.2105045	0.01	GFRA3	21.0504546
858	7.8037873	0.3711747	SERPINA3	21.02456845

859	4.8262603	0.2295534	SLC8A3	21.0245684
860	0.2101861	0.01	SPDYE2	21.0186082
861	0.2100801	0.01	LOC100506241	21.0080141
862	0.2096574	0.01	ACTG1P4	20.9657445
863	13.646208	0.6517311	CCL5	20.93840218
864	40.511005	1.9357265	RASL12	20.92806221
865	0.2079835	0.01	BREA2	20.7983533
866	0.2075692	0.01	MAGEC2	20.7569223
867	0.2073627	0.01	ZNF702P	20.7362687
868	0.2070194	0.01	ARHGAP25	20.7019371
869	1.3333301	0.0644747	DNM3OS	20.67990342
870	4.2271704	0.2044096	TIAM2	20.6799034
871	1.7973221	0.0869115	ASMTL-AS1	20.67990335
872	0.2063361	0.01	GDF9	20.6336139
873	10.738678	0.5224154	TENM2	20.55582397
874	2045.307	99.676386	CDK4	20.51947369
875	0.2051176	0.01	AFP	20.5117618
876	0.2051176	0.01	FGF20	20.5117618
877	0.2047146	0.01	GGT3P	20.4714637
878	0.2045137	0.01	RLN1	20.4513739
879	0.2041131	0.01	TAS2R31	20.4113125
880	0.2037539	0.01	MCMDC2	20.3753911
881	0.2037141	0.01	MRAP	20.3714076
882	0.2034158	0.01	LOC100272216	20.3415813
883	0.2032175	0.01	GCGR	20.3217455
884	0.2024082	0.01	PLB1	20.2408217
885	0.2022314	0.01	MYOC	20.2231441
886	0.202068	0.01	KCNJ15	20.2068035
887	0.2019375	0.01	LOC440335	20.19375
888	0.2018397	0.01	NHLRC4	20.183971
889	2.8378424	0.1407457	KRT42P	20.16290583
890	0.2015469	0.01	DKKL1	20.1546905
891	0.2014495	0.01	LOC100132215	20.1449493
892	0.2011578	0.01	FGF8	20.1157819
893	1431.409	71.453393	HLA-A	20.03276501
894	5.0133434	0.2507854	C1QTNF2	19.99057325
895	0.1998078	0.01	HSD52	19.9807766
896	0.1988545	0.01	BEST4	19.8854485
897	0.1982869	0.01	CDRT15L2	19.828687
898	0.1981926	0.01	PTGIR	19.8192582
899	0.1980984	0.01	ST7-OT3	19.8098384
900	0.1980984	0.01	LOC100507299	19.8098384
901	0.196789	0.01	FCN3	19.6788952

902	0.1966033	0.01	AQP7P1	19.6603302
903	0.1965106	0.01	PXT1	19.6510608
904	1.6161594	0.0822644	TNIP3	19.6459083
905	2.5998624	0.1323361	SIGLEC15	19.64590824
906	2.9894983	0.152169	DDIT4L	19.64590821
907	183.94948	9.3632464	SRGN	19.6459082
908	3.8554922	0.1962491	RTP4	19.64590819
909	3.5676578	0.181598	RUNX1T1	19.64590818
910	1.6287908	0.0829074	GGT1	19.64590811
911	0.1959563	0.01	C17orf47	19.5956277
912	0.1954051	0.01	KCTD4	19.5405063
913	0.194584	0.01	PYY2	19.4584034
914	0.1942213	0.01	NINJ2	19.4221342
915	0.194161	0.01	ADAM28	19.4161025
916	0.1940405	0.01	GZMK	19.4040503
917	120.23357	6.2182136	ARHGEF25	19.33570965
918	0.1931413	0.01	CLEC4E	19.3141335
919	7.8800621	0.4082671	EN1	19.30124316
920	5.5467548	0.2873781	TCN2	19.30124315
921	0.1922505	0.01	LOC100129480	19.2250461
922	0.1901455	0.01	ZNF502	19.0145529
923	0.189684	0.01	DENND2D	18.9684011
924	4.4599115	0.2352699	FERMT3	18.95657813
925	0.1890531	0.01	COL10A1	18.9053069
926	0.1889816	0.01	GPR113	18.8981637
927	0.1889388	0.01	GRIK1-AS1	18.8938803
928	0.1888532	0.01	CCR7	18.8853195
929	0.1888532	0.01	TMEM45B	18.8853195
930	6.7166285	0.3559344	CEACAM19	18.87041182
931	0.1885542	0.01	KIAA1683	18.8554173
932	0.1881711	0.01	GIMAP1	18.8171106
933	0.1868216	0.01	MSTO2P	18.6821605
934	0.1867379	0.01	SPIC	18.6737903
935	0.1864038	0.01	CRB3	18.6403846
936	0.1864038	0.01	BET3L	18.6403846
937	0.1861264	0.01	CLDN18	18.6126377
938	0.5194836	0.0279113	PLCB1	18.61191333
939	12.690091	0.6818263	GSTM2	18.61191303
940	6.5914444	0.3541519	ERAP2	18.61191301
941	21.134656	1.1400686	SLIT2	18.53805624
942	0.1850795	0.01	CCDC116	18.5079485
943	163.55781	8.8465658	EFEMP2	18.48828317
944	0.1842613	0.01	LINC00619	18.4261273

945	46.264689	2.5193481	PCBP3	18.3637542
946	6.9906161	0.3826858	HLA-J	18.26724796
947	0.1817966	0.01	SLC36A2	18.1796598
948	0.1816118	0.01	PCDHGB1	18.1611765
949	0.1816118	0.01	NYAP2	18.1611765
950	0.1812564	0.01	PCDHGB6	18.125636
951	1.8688144	0.1032784	P2RX7	18.09491552
952	0.1804325	0.01	TNFRSF6B	18.0432468
953	0.1791143	0.01	KRT3	17.911431
954	0.1781954	0.01	HAPLN1	17.8195383
955	0.1781192	0.01	LOC388553	17.8119231
956	0.1776637	0.01	HTR1B	17.7663683
957	0.1776132	0.01	PVRL4	17.761321
958	0.1775124	0.01	LOC100131434	17.7512351
959	0.1763109	0.01	PROKR1	17.6310914
960	0.1763109	0.01	RERGL	17.6310914
961	0.1760131	0.01	C9orf24	17.6013091
962	35.357201	2.0114556	LOC100505806	17.57791787
963	9.2501084	0.5262346	EBI3	17.57791786
964	10.491979	0.596884	MOXD1	17.57791785
965	2.6146063	0.1487438	GBP5	17.57791782
966	0.828142	0.0471126	ZNF287	17.57791776
967	0.1751992	0.01	ZNF883	17.5199244
968	0.1748318	0.01	MAP1LC3C	17.4831795
969	1.6571389	0.0949354	OAS2	17.45543744
970	0.1741015	0.01	LCE1B	17.4101504
971	0.1739562	0.01	LOC100144602	17.3956177
972	1085.8849	62.650119	HLA-C	17.33252752
973	0.1730893	0.01	LINC00525	17.3089286
974	0.1723378	0.01	C10orf105	17.2337813
975	0.172231	0.01	C5orf60	17.2230992
976	261.81081	15.21986	METTL1	17.20191962
977	0.1718523	0.01	C6orf223	17.1852254
978	0.1715928	0.01	FCGR2A	17.1592837
979	0.1715928	0.01	CYP21A1P	17.1592837
980	10.123259	0.5908019	CYP27A1	17.13477707
981	0.170261	0.01	EGFL6	17.026103
982	0.1695683	0.01	ZNF709	16.9568348
983	10.885638	0.6433623	F2RL2	16.91992093
984	4.3086816	0.2551239	HOTAIR	16.88858776
985	52.211597	3.0974115	PAMR1	16.85652589
986	0.1677935	0.01	CAPN9	16.7793478
987	0.1672548	0.01	MMP23B	16.7254816

988	0.1672548	0.01	PDC	16.7254816
989	0.1672548	0.01	PRDM12	16.7254816
990	0.1671207	0.01	CLDN14	16.712069
991	0.1664533	0.01	IL20	16.6453275
992	0.1663536	0.01	ZFP3	16.6353622
993	23.751414	1.429042	TFAP2A	16.62051492
994	0.1660554	0.01	HAVCR1P1	16.6055379
995	0.1656594	0.01	AQP7	16.565938
996	1.3795581	0.0833876	IL4I1	16.54392278
997	1.2200483	0.073746	PCDHB2	16.54392263
998	0.1650036	0.01	CD37	16.5003563
999	0.1645476	0.01	SIX3	16.4547572
1000	0.1642234	0.01	LINC00310	16.4223404
1001	9.1072223	0.5554468	RGS9	16.3962091
1002	452.45608	27.683825	CTDSP2	16.3436982
1003	0.1633225	0.01	CYP4A11	16.3322492
1004	0.1633225	0.01	GSTA1	16.3322492
1005	0.1622417	0.01	SLA2	16.2241728
1006	4.8754487	0.3009674	EVI2B	16.19925766
1007	2.853748	0.1761654	CACNA1C	16.19925759
1008	0.1619266	0.01	LOC100506801	16.1926574
1009	0.1609263	0.01	HYPK	16.0926255
1010	0.1606781	0.01	SPATA32	16.0678103
1011	0.1606162	0.01	LOC100287639	16.0616185
1012	0.1603073	0.01	H1FNT	16.0307308
1013	1.3920242	0.0868554	GALNT15	16.02692516
1014	2.3172111	0.1445824	CCDC81	16.02692508
1015	1.8445066	0.115088	PDE5A	16.02692507
1016	0.1598156	0.01	RPE65	15.9815568
1017	10.16905	0.638618	HLA-G	15.9235256
1018	11.274759	0.7100327	ARID5B	15.87921153
1019	71.109794	4.478841	PLA2G16	15.87682904
1020	0.1587201	0.01	CSNK1G2-AS1	15.8720107
1021	2.5003592	0.1577057	TSLP	15.85459255
1022	0.158118	0.01	LOC286059	15.8117982
1023	0.1579382	0.01	ZNF763	15.7938234
1024	0.1576396	0.01	STMND1	15.7639561
1025	4.1455977	0.2633847	MAF	15.73970425
1026	0.1569273	0.01	WDR38	15.6927334
1027	185.99308	11.889597	TSFM	15.64334626
1028	19.060075	1.2198592	RBP1	15.62481588
1029	0.1557545	0.01	PCDHA4	15.5754484
1030	0.1553288	0.01	ZNF658	15.532882

1031	0.1552902	0.01	CPA2	15.5290239
1032	0.4996791	0.0322167	PPL	15.50992775
1033	1.0525227	0.0678612	INSC	15.50992752
1034	3.9531995	0.2548819	AVIL	15.50992751
1035	1.0960703	0.070669	BEST1	15.50992744
1036	0.1550592	0.01	SYS1-DBNDD2	15.5059152
1037	0.154599	0.01	SULT1A2	15.4599036
1038	0.15437	0.01	ANXA2P3	15.437
1039	0.1542557	0.01	CCL8	15.4255737
1040	0.1542557	0.01	CLIC2	15.4255737
1041	0.1542557	0.01	LOC100128593	15.4255737
1042	0.153914	0.01	RAB40A	15.3913959
1043	0.153914	0.01	NMBR	15.3913959
1044	4.8177329	0.3148201	EPSTI1	15.30312849
1045	0.1522275	0.01	FGFBP1	15.2227538
1046	0.1522275	0.01	SMIM21	15.2227538
1047	0.1520609	0.01	GCK	15.2060927
1048	0.1516736	0.01	GHRL	15.1673581
1049	0.1513431	0.01	SPANXN2	15.1343137
1050	14.217342	0.9401649	NAPRT1	15.12217933
1051	0.1508684	0.01	ZNF585A	15.0868364
1052	36.465102	2.4193186	CYGB	15.07246803
1053	0.150252	0.01	ZNF835	15.0251983
1054	35.395854	2.3565594	PHLDA3	15.02014034
1055	2.3598538	0.1573978	FKBP9L	14.99292995
1056	8.004749	0.5339016	LIN7B	14.99292994
1057	18.934183	1.2738556	NID2	14.86368055
1058	0.1485385	0.01	ZNF233	14.8538489
1059	0.148327	0.01	PPP1R36	14.8327046
1060	54.978198	3.7115878	ADAMTS1	14.81258195
1061	0.148011	0.01	AKR1C6P	14.8011009
1062	6.2049538	0.4204488	COL15A1	14.75793106
1063	0.147383	0.01	NR1I3	14.7382956
1064	7.5372916	0.5115428	PIR	14.73443116
1065	113.36841	7.6960716	FADS3	14.7306848
1066	0.1472788	0.01	LINC00856	14.7278799
1067	0.1470709	0.01	ZNF844	14.7070925
1068	0.147019	0.01	HEXA-AS1	14.7019048
1069	0.1469672	0.01	LOC255130	14.6967207
1070	0.1458359	0.01	RSPH4A	14.5835899
1071	0.1455304	0.01	FRK	14.5530377
1072	0.1454627	0.01	GRIN2C	14.5462657
1073	0.1452261	0.01	RNF126P1	14.5226133

1074	0.1451587	0.01	LOC643201	14.5158695
1075	0.145024	0.01	SPTY2D1-AS1	14.5024008
1076	0.1449232	0.01	APOL5	14.4923157
1077	1.2909704	0.0891805	DSCR6	14.47593241
1078	1.5899689	0.1098353	LGALS9	14.47593237
1079	3.149048	0.2175368	MUC1	14.47593237
1080	0.4555892	0.0314722	ZNF117	14.47593235
1081	1.7032067	0.1176578	IL3RA	14.47593234
1082	0.7131736	0.0492662	ERCC5	14.47593227
1083	0.7283058	0.0503115	CSF1R	14.47593223
1084	0.1447219	0.01	HPCA	14.4721875
1085	0.1446214	0.01	SGCA	14.4621444
1086	0.1442211	0.01	LOC400456	14.4221107
1087	18.318047	1.2759299	CTSS	14.35662523
1088	0.1431807	0.01	KCTD19	14.3180694
1089	16.005214	1.1228292	MEST	14.25436197
1090	0.1422522	0.01	BTBD8	14.2252219
1091	64.942421	4.5870483	METTL21B	14.15778
1092	0.1414796	0.01	ZACN	14.1479634
1093	0.1411921	0.01	NXNL2	14.1192073
1094	0.1404309	0.01	IL1R2	14.043093
1095	0.1403364	0.01	LINC00482	14.0336364
1096	0.1403364	0.01	AIM2	14.0336364
1097	30.272099	2.15724	CCDC80	14.03279157
1098	15.826168	1.1291196	PBXIP1	14.01637895
1099	0.1400534	0.01	CASP5	14.0053428
1100	0.1393977	0.01	NOD2	13.9397659
1101	0.1392114	0.01	CCL16	13.9211423
1102	0.1388404	0.01	FTLP10	13.884044
1103	3.4659964	0.2501525	FAT4	13.85553524
1104	0.1381044	0.01	ATP4B	13.8104374
1105	0.1381044	0.01	LINC00518	13.8104374
1106	0.1375574	0.01	PCDHB6	13.7557426
1107	0.1371049	0.01	TMEM56-RWDD3	13.7104934
1108	0.1370148	0.01	TOP1P2	13.7014793
1109	0.1370148	0.01	GPR87	13.7014793
1110	0.1369248	0.01	HPGD	13.692477
1111	0.1366554	0.01	GPR84	13.665541
1112	0.1361643	0.01	BTBD18	13.6164325
1113	21.492899	1.5787038	HSBP1L1	13.61426971
1114	0.1357651	0.01	PCDHGA7	13.5765147
1115	0.1357651	0.01	KCNE3	13.5765147
1116	0.1355885	0.01	TICAM2	13.5588484

1117	0.1354563	0.01	NEBL	13.5456289
1118	0.1354563	0.01	MOBP	13.5456289
1119	0.1352365	0.01	SYNE4	13.5236535
1120	0.1350612	0.01	ADIPOQ	13.5061244
1121	0.1350612	0.01	LAMB2P1	13.5061244
1122	0.1349738	0.01	FKBP6	13.497377
1123	0.1347992	0.01	LOC158376	13.4799159
1124	0.5581363	0.041522	FAM198B	13.44193725
1125	1.6764811	0.1247202	TEKT4P2	13.44193724
1126	4.551081	0.3385733	SERPINB9	13.4419372
1127	0.735195	0.0546941	KLHL30	13.4419372
1128	0.5861518	0.0436062	LOC284837	13.4419372
1129	2.0594401	0.1532101	DHX58	13.4419372
1130	26.672791	1.9842967	EMID1	13.44193719
1131	1.7550379	0.1305644	IFIT1	13.44193718
1132	0.1341052	0.01	TUBA3E	13.4105212
1133	0.1341052	0.01	FAS-AS1	13.4105212
1134	181.02262	13.508569	CARS2	13.40057738
1135	0.133718	0.01	LOC645752	13.3717998
1136	291.5901	21.818441	SELM	13.36438755
1137	0.1332904	0.01	HRASLS5	13.3290374
1138	5.3824344	0.4043086	ABCB4	13.3126878
1139	0.1331201	0.01	KLF17	13.312009
1140	0.1330776	0.01	NKAIN3	13.3077586
1141	0.1329078	0.01	FAM110D	13.2907844
1142	0.1322332	0.01	PATE1	13.2233185
1143	0.131982	0.01	TP53AIP1	13.1981951
1144	0.1312339	0.01	FXYD3	13.1233942
1145	7.0527057	0.5384866	MR1	13.09727212
1146	62.813872	4.8081958	FBN1	13.06391745
1147	0.1303311	0.01	ITIH2	13.0331145
1148	0.1303311	0.01	ST13P4	13.0331145
1149	0.1302497	0.01	TEX101	13.0249688
1150	19.869075	1.5285319	COPZ2	12.9987964
1151	0.1299249	0.01	C1orf101	12.9924875
1152	0.1299249	0.01	C20orf202	12.9924875
1153	18.355959	1.4131313	SECTM1	12.98956431
1154	0.1298439	0.01	ZNF860	12.9843925
1155	34.83018	2.6873046	GNAZ	12.9610092
1156	0.1292801	0.01	PSG2	12.9280087
1157	4.228886	0.3271881	GBP4	12.92493959
1158	48.517026	3.7591615	LRP1	12.90634257
1159	6.5188504	0.5066138	PITPNM3	12.86749543

1160	0.1283248	0.01	C9orf50	12.8324815
1161	0.1282458	0.01	ATP6V1C2	12.8245846
1162	0.1282458	0.01	TMPRSS2	12.8245846
1163	3.9153845	0.3053755	CD22	12.82154009
1164	0.128167	0.01	KRT33B	12.8166974
1165	26.002139	2.0339691	SERPINB2	12.78394026
1166	0.1265328	0.01	FAM183B	12.6532787
1167	2.3493568	0.186239	QRICH2	12.61474107
1168	84.389912	6.7054892	CDKN2C	12.58519834
1169	0.1248649	0.01	PSG5	12.4864889
1170	0.1243434	0.01	CD69	12.4343377
1171	0.2831194	0.0228176	VWF	12.40794228
1172	0.4961893	0.0399897	LPPR4	12.40794208
1173	1.2598458	0.1015354	C3AR1	12.40794207
1174	3.3718571	0.2717499	TRAF1	12.40794203
1175	0.8686329	0.0700062	C1orf213	12.40794203
1176	4.2512435	0.3426228	ZNF423	12.40794201
1177	0.6356873	0.0512323	PABPC1P2	12.40794194
1178	0.1239735	0.01	DSCR9	12.3973528
1179	0.1236059	0.01	TNF	12.3605872
1180	0.1229496	0.01	LOC731424	12.2949558
1181	0.1229496	0.01	ZNF157	12.2949558
1182	0.1227323	0.01	C1orf228	12.2732332
1183	0.1226601	0.01	SNX15	12.2660094
1184	0.1224439	0.01	TMLHE-AS1	12.244389
1185	0.1224439	0.01	TMSB4Y	12.244389
1186	0.1220138	0.01	COLEC10	12.2013759
1187	0.1220138	0.01	ABHD12B	12.2013759
1188	0.1219424	0.01	C19orf45	12.1942364
1189	0.1215157	0.01	PPEF2	12.1515744
1190	0.1213742	0.01	CETP	12.1374199
1191	24.926783	2.0556572	SH2B2	12.12594334
1192	0.1210218	0.01	LEP	12.1021777
1193	17.480345	1.4454632	FOXL1	12.09324784
1194	0.1208113	0.01	AADAC	12.0811304
1195	117.14022	9.7131493	FOS	12.05996288
1196	10.168496	0.8459511	GLT8D2	12.02019383
1197	0.1197698	0.01	ELN	11.9769828
1198	1.29799	0.108484	NRXN3	11.96480126
1199	0.1195637	0.01	LGALS9C	11.9563683
1200	0.1195637	0.01	SVOP	11.9563683
1201	0.1193582	0.01	LOC100130950	11.9358248
1202	0.1193241	0.01	TNFSF4	11.9324077

1203	0.5571208	0.0468525	ABCA13	11.89094455
1204	0.1185435	0.01	YBX3P1	11.8543515
1205	84.826623	7.1599003	CLU	11.84745866
1206	9.7216308	0.8211361	IL1R1	11.83924468
1207	4.9607461	0.4192121	LRRC32	11.83350027
1208	34.061935	2.879255	C12orf68	11.83012119
1209	0.1182744	0.01	ZNF878	11.8274404
1210	0.1180734	0.01	LHFPL1	11.8073371
1211	0.1178064	0.01	ADORA2A-AS1	11.7806388
1212	0.1176733	0.01	ANAPC1P1	11.7673348
1213	0.1176733	0.01	A4GNT	11.7673348
1214	0.1176733	0.01	TRIM63	11.7673348
1215	0.1175406	0.01	LDHAL6B	11.7540609
1216	0.1174744	0.01	KYNU	11.7474352
1217	0.1173751	0.01	GTF2IRD2	11.7375106
1218	0.1172101	0.01	NXPE2	11.7210068
1219	5.1348854	0.4391757	TGFB2	11.69209922
1220	0.1168486	0.01	DNM1P35	11.6848612
1221	0.1166851	0.01	WASH7P	11.668505
1222	6.2470612	0.5357149	ADAMTS2	11.66116773
1223	0.1165545	0.01	AVPR2	11.655453
1224	0.1164243	0.01	FAM74A1	11.6424302
1225	10.350969	0.889836	PQLC3	11.63244564
1226	0.1162295	0.01	TTLL13	11.6229504
1227	0.1159385	0.01	GTF2IRD2B	11.5938526
1228	113.01604	9.7610271	KANK2	11.57829373
1229	19.390378	1.6750137	BAI1	11.57625026
1230	231.68119	20.044949	TIMP2	11.55808298
1231	0.1152015	0.01	CEACAM20	11.5201493
1232	0.1150108	0.01	LRRC19	11.5010762
1233	0.114884	0.01	FGB	11.4883958
1234	16.445497	1.4340403	UCN2	11.46794641
1235	0.1146312	0.01	FAM180A	11.4631188
1236	0.1140665	0.01	MKRN9P	11.4066503
1237	0.1138173	0.01	LOC100129935	11.3817313
1238	0.1138173	0.01	FLJ37201	11.3817313
1239	0.3553549	0.0312429	XIRP1	11.37394701
1240	0.4139391	0.0363936	ZNF701	11.37394696
1241	0.4017516	0.0353221	NLGN4X	11.37394696
1242	0.5735288	0.0504248	AFAP1L2	11.37394694
1243	1.1804297	0.1037836	LOC100506394	11.3739469
1244	0.9453173	0.0831125	ITGB2-AS1	11.37394689
1245	2.3288803	0.2047557	PCDH18	11.37394687

1246	0.8951169	0.0786989	AGSK1	11.37394686
1247	5.3460693	0.4700276	KRT79	11.37394685
1248	8.4374656	0.7418239	SERPINA1	11.37394685
1249	0.9943156	0.0874205	ZFP30	11.37394684
1250	2.3273041	0.2046171	CDA	11.37394684
1251	0.4190083	0.0368393	FAM65B	11.37394677
1252	0.5378683	0.0472895	GPR78	11.37394675
1253	0.1136311	0.01	KLHL38	11.3631134
1254	0.1136311	0.01	CD40LG	11.3631134
1255	0.1134765	0.01	SLC26A1	11.347645
1256	0.1133222	0.01	HRK	11.3322186
1257	0.1133016	0.01	GDA	11.3301649
1258	53.188636	4.6951879	ARHGEF16	11.32832942
1259	38.171692	3.373589	P2RX4	11.31486141
1260	242.42202	21.427907	RBM19	11.3133781
1261	0.1127093	0.01	C1QTNF9	11.2709302
1262	0.1126484	0.01	DDX25	11.2648378
1263	0.1124053	0.01	CRHBP	11.240534
1264	131.28906	11.68148	IL11	11.23907792
1265	0.1119224	0.01	GPR171	11.1922395
1266	0.1118923	0.01	MOB4	11.1892349
1267	0.1118623	0.01	LOC284632	11.1862319
1268	123.89255	11.094377	MLLT11	11.16714782
1269	0.1116227	0.01	CHI3L1	11.1622657
1270	111.94327	10.052262	MFGE8	11.13612797
1271	0.1112057	0.01	SLC35G3	11.120571
1272	0.1110872	0.01	DIO1	11.1087154
1273	0.1109689	0.01	SLC6A7	11.096885
1274	10.795058	0.9732337	CA8	11.09194818
1275	0.110733	0.01	SKP1P2	11.0732997
1276	58.244222	5.2627989	SDC2	11.06715708
1277	0.1106154	0.01	LOC100505474	11.0615446
1278	1.0459197	0.0948312	MRVI1	11.02928183
1279	35.472255	3.2161891	RNU2-1	11.0292818
1280	9.8178638	0.8901635	FLJ23867	11.02928179
1281	12.203271	1.1084806	RAB37	11.00900738
1282	0.1099734	0.01	CYP17A1	10.9973351
1283	0.1099153	0.01	HMX1	10.9915348
1284	0.1099153	0.01	GPT	10.9915348
1285	177.707	16.213626	TK1	10.96034878
1286	39.225563	3.6028532	IAH1	10.88736089
1287	377.78303	34.769284	PFKM	10.86542464
1288	1.0201374	0.0939617	RAB3D	10.8569493

1289	27.352434	2.5193481	CSF2	10.85694927
1290	10.133463	0.933362	RIT1	10.85694926
1291	1.379694	0.1270793	SLC25A34	10.85694925
1292	0.7881126	0.0725906	NXPH3	10.85694921
1293	0.1085414	0.01	JAKMIP2-AS1	10.8541406
1294	0.1083157	0.01	LINC00535	10.8315749
1295	0.108222	0.01	TMEM132D	10.8222001
1296	0.1082033	0.01	KRT81	10.8203271
1297	3.9995864	0.3732368	LRRC15	10.71594993
1298	0.1071463	0.01	OMG	10.7146273
1299	0.1069813	0.01	FLJ42969	10.6981263
1300	1.7213921	0.1611094	LOC100126784	10.68461676
1301	1.093868	0.1023778	CASP10	10.68461675
1302	10.894409	1.019635	GPX3	10.68461675
1303	0.1068168	0.01	CLUL1	10.6816761
1304	0.1067074	0.01	NXPE1	10.6707373
1305	0.1066528	0.01	APOA5	10.6652764
1306	28.048928	2.6346124	HEYL	10.64632062
1307	0.1061097	0.01	C3orf65	10.6109725
1308	0.1056793	0.01	ZNF474	10.567926
1309	0.1052257	0.01	VIPR2	10.5225701
1310	0.105093	0.01	RIBC1	10.5093041
1311	0.1050401	0.01	SERPING1	10.5040071
1312	0.1049872	0.01	PLA2G2D	10.4987154
1313	0.1049343	0.01	NTN3	10.493429
1314	0.1048287	0.01	FUT5	10.4828722
1315	0.1045657	0.01	CASQ1	10.456573
1316	72.423383	6.933716	CD82	10.44510373
1317	0.1043563	0.01	SLC22A16	10.4356285
1318	0.1043041	0.01	EPPIN	10.4304054
1319	0.1041998	0.01	LINC00475	10.419975
1320	19.279897	1.8503687	CDKN2D	10.41948978
1321	0.1040697	0.01	L1TD1	10.4069663
1322	0.1039399	0.01	TTC24	10.39399
1323	0.1039399	0.01	MT1IP	10.39399
1324	0.1038881	0.01	SLC38A5	10.3888086
1325	13.353394	1.2854579	NLRC5	10.38804449
1326	0.103733	0.01	DCSTAMP	10.3732952
1327	0.1035783	0.01	LOC100128076	10.357828
1328	0.1035268	0.01	DCAF8L2	10.3526826
1329	56.604387	5.4689759	TP53I11	10.35008889
1330	0.2915086	0.0281925	ZNF470	10.33995172
1331	1.1867853	0.1147767	AP1M2	10.33995172

1332	0.6794897	0.065715	LOC400958	10.33995171
1333	1.3106887	0.1267597	EFNA1	10.33995171
1334	2.6513931	0.2564222	MGC16121	10.3399517
1335	1.8817111	0.1819845	AMT	10.33995169
1336	47.256122	4.570246	PTPLA	10.33995169
1337	1.8169093	0.1757174	MMP7	10.33995167
1338	0.1032703	0.01	LOC440173	10.3270317
1339	106.38068	10.31175	IFI6	10.3164518
1340	5443.3949	528.05536	BCYRN1	10.30837931
1341	0.103066	0.01	TRH	10.3066024
1342	0.103066	0.01	DOC2B	10.3066024
1343	0.1029642	0.01	GC	10.296418
1344	0.1027864	0.01	HLA-DPB1	10.2786437
1345	0.102458	0.01	LOC286359	10.2457965
1346	0.1024076	0.01	LOC392452	10.2407617
1347	106.39413	10.418236	LOC100130776	10.21229796
1348	8.4312902	0.8272266	GYG2	10.19223809
1349	0.1019068	0.01	LRRC43	10.1906846
1350	0.1016335	0.01	CHRDL1	10.1633504
1351	0.1016087	0.01	HIF1A-AS2	10.1608728
1352	0.1015097	0.01	KCNIP1	10.1509742
1353	2.9702081	0.2931179	PLSCR4	10.13315264
1354	230.01797	22.712246	LMNA	10.12748693
1355	0.1008222	0.01	FAM194A	10.0822206
1356	17.116684	1.7004037	ZBTB7B	10.06624708
1357	0.1002403	0.01	CHGA	10.024026
1358	0.1000958	0.01	HCAR2	10.0095821
1359	2.4174342	0.2418574	GGT5	9.995286611
1360	0.0996174	0.01	RGS22	9.9617352
1361	16.044088	1.6121141	TNFRSF9	9.952203495
1362	0.0994272	0.01	TRPM3	9.9427242
1363	5.2518436	0.5283707	SEMA5A	9.939695482
1364	0.0992615	0.01	GREM2	9.9261491
1365	0.0990492	0.01	SMCO3	9.9049192
1366	1632.1688	165.72253	MYL6	9.848803984
1367	0.0984255	0.01	MYO3B	9.8425457
1368	0.8378313	0.0852932	ZNF28	9.822954048
1369	0.0981165	0.01	LOC100505716	9.8116526
1370	0.0979781	0.01	MGC16025	9.7978138
1371	0.0977025	0.01	SLFNL1	9.7702532
1372	0.0977025	0.01	SMC1B	9.7702532
1373	0.0977025	0.01	NME9	9.7702532
1374	86.429836	8.8487988	CARKD	9.767408905

1375	0.0976567	0.01	FAM83F	9.7656748
1376	0.097474	0.01	LOC100132781	9.7474041
1377	0.0970654	0.01	LMCD1-AS1	9.706544
1378	0.0968399	0.01	TRIM72	9.6839916
1379	0.0965259	0.01	PLCZ1	9.6525938
1380	1.8015394	0.186676	PLA2G6	9.650621577
1381	1.2799267	0.1326264	ZNF611	9.65062154
1382	1.1785874	0.1221255	GPR85	9.650621538
1383	0.0964366	0.01	TMEM139	9.6436604
1384	4.9424421	0.513548	MGAT3	9.624108875
1385	0.0961696	0.01	ARSF	9.6169589
1386	0.0961696	0.01	UPB1	9.6169589
1387	0.0961252	0.01	PGAM1P5	9.6125231
1388	0.0958599	0.01	TDGF1	9.5859936
1389	0.0958599	0.01	CLCNKB	9.5859936
1390	71.174693	7.4341419	HIST1H1C	9.574029338
1391	1.3863325	0.1449463	TSPAN11	9.564455321
1392	50.082214	5.2391748	GABARAPL1	9.559179822
1393	341.97188	35.79788	EGR1	9.552853029
1394	3396.2573	357.15014	B2M	9.509326633
1395	0.0949428	0.01	NRG4	9.4942825
1396	10.044548	1.0579939	BTN3A1	9.49395564
1397	0.0944694	0.01	LOC100505715	9.4469402
1398	8.9876901	0.9525696	APOL6	9.435205911
1399	14.614104	1.551252	JUP	9.42084487
1400	0.0940856	0.01	C6orf165	9.4085553
1401	0.0940431	0.01	LOC115110	9.4043096
1402	3.940989	0.4192561	EBF1	9.39995607
1403	0.093747	0.01	NR1I2	9.3746964
1404	0.0936206	0.01	ZBED2	9.362062
1405	9.0630249	0.9685144	FGF1	9.357656279
1406	0.0934527	0.01	CACNA1C-AS1	9.3452691
1407	0.0932436	0.01	PRAM1	9.3243624
1408	0.5045993	0.0542233	PLEKHA6	9.305956557
1409	0.7624372	0.08193	CHRNE	9.305956538
1410	0.7177939	0.0771327	MYCN	9.305956528
1411	1.1627994	0.1249522	TSPAN10	9.305956524
1412	3.4297723	0.3685567	GPR55	9.305956523
1413	2.0222054	0.2173023	ZNF774	9.305956522
1414	1.0484044	0.1126595	CLUHP3	9.305956512
1415	4.011969	0.4311184	LINC00460	9.305956508
1416	1.4086335	0.151369	DNALI1	9.3059565
1417	1.465309	0.1574593	LOC100505933	9.305956495

1418	0.7238887	0.0777877	EXOC3L4	9.305956492
1419	0.648995	0.0697397	ZNF596	9.305956446
1420	0.092622	0.01	C8orf86	9.2622
1421	27.420987	2.9776229	MEIS3	9.209019472
1422	0.0920086	0.01	LINC00511	9.2008609
1423	0.0919274	0.01	CCDC17	9.1927437
1424	0.0918869	0.01	FCGR1A	9.1886905
1425	0.0917655	0.01	ZPBP2	9.1765522
1426	0.0913232	0.01	ST20-MTHFS	9.1323181
1427	101.93005	11.17493	FSTL3	9.121314525
1428	0.0912033	0.01	OXGR1	9.1203282
1429	0.0910041	0.01	TISP43	9.1004149
1430	0.0910041	0.01	LRRC70	9.1004149
1431	1.6298574	0.1791218	ST8SIA2	9.099157485
1432	0.0909644	0.01	ZNF571	9.0964426
1433	0.0908059	0.01	LOC253573	9.0805882
1434	0.0906873	0.01	PCDHGB3	9.0687337
1435	0.0906479	0.01	AIRE	9.064789
1436	0.0906085	0.01	HAS2-AS1	9.0608478
1437	0.0905691	0.01	PCDHGA5	9.05691
1438	0.0905691	0.01	PCDHGA4	9.05691
1439	0.0904512	0.01	KCNF1	9.0451172
1440	0.0903727	0.01	SPTSSB	9.0372723
1441	0.0901577	0.01	PCDHGC4	9.015769
1442	0.0899825	0.01	TMEM155	8.9982513
1443	0.0899437	0.01	LOC100652999	8.9943677
1444	0.0898855	0.01	C9orf84	8.9885486
1445	0.0895187	0.01	NDUFAF4P1	8.9518686
1446	0.0892886	0.01	MST1L	8.928856
1447	0.0889646	0.01	TXLNB	8.8964568
1448	1.2562987	0.1412785	SAMD9L	8.892358465
1449	4.4406236	0.4993752	VSTM2L	8.892358444
1450	0.0889076	0.01	PRDM14	8.8907637
1451	56.523794	6.3646689	PLK2	8.880869613
1452	0.0886429	0.01	MIA2	8.8642918
1453	0.0885299	0.01	BSPRY	8.8529949
1454	2.5407311	0.2879529	TRANK1	8.823425437
1455	0.0882301	0.01	LOC100144595	8.8230102
1456	2765.4363	313.57284	FTH1	8.819119349
1457	0.3915552	0.0445508	F8	8.78895897
1458	1.1589112	0.1318599	TLR3	8.78895896
1459	4.7300287	0.5381785	LOC100507463	8.788958933
1460	1.7529894	0.1994536	B4GALNT2	8.78895891

1461	0.0877472	0.01	FCRLA	8.7747158
1462	98.401928	11.214409	P4HA2	8.774597885
1463	5.7448961	0.6562226	CRISPLD2	8.754492427
1464	1.66697	0.1912737	LAMA4	8.715102142
1465	59.644881	6.8473702	CTIF	8.710625965
1466	6.9628205	0.8003406	ALPK2	8.699821415
1467	0.0869418	0.01	ITGB6	8.6941802
1468	0.0869237	0.01	TACR1	8.6923671
1469	1.150017	0.1324056	NCKAP5	8.685559424
1470	0.0868331	0.01	CHRNA6	8.6833125
1471	0.0867608	0.01	CCRL1	8.6760824
1472	4.5899264	0.5294245	AOX1	8.669651795
1473	0.0866166	0.01	FCGR3A	8.6616584
1474	0.0864728	0.01	RFPL2	8.6472822
1475	0.086437	0.01	PCDHB17	8.6436956
1476	1.6904567	0.1961854	STXBP5-AS1	8.616626402
1477	0.0859025	0.01	GTF2IRD1P1	8.5902514
1478	26.832837	3.1242786	LINC00346	8.588490481
1479	73.269215	8.5467476	NINJ1	8.572759942
1480	0.0857258	0.01	TOP1P1	8.5725833
1481	17.791839	2.0778129	PLEKHA4	8.562772489
1482	0.0855499	0.01	PCDHGA11	8.5549877
1483	22.219598	2.598352	GPRC5B	8.551419501
1484	0.7435597	0.0871652	ABCA5	8.530460149
1485	17.611225	2.0645106	CRYBB2	8.530460142
1486	6.3677625	0.7464735	CCDC159	8.530460139
1487	3.5762785	0.4192363	PDGFRL	8.53046013
1488	1.0725489	0.1257317	KIAA1755	8.530460117
1489	0.085235	0.01	DCAF13P3	8.5234969
1490	0.0852001	0.01	BCMO1	8.5200123
1491	15.38087	1.8087228	WWC3	8.503718887
1492	0.0849224	0.01	GBP7	8.4922372
1493	0.0847497	0.01	KRT2	8.4749695
1494	367.67757	43.429395	IGFBP7	8.466099215
1495	0.0846121	0.01	ARL2-SNX15	8.4612059
1496	22.577367	2.6692181	ZBED1	8.458419496
1497	0.0845777	0.01	LOC158435	8.4577719
1498	6.2189863	0.736472	TNFSF12	8.444293874
1499	0.0843381	0.01	CORIN	8.4338122
1500	0.0842699	0.01	SLC30A2	8.4269915
1501	0.0842359	0.01	ZP4	8.4235853
1502	821.33921	97.586291	SPARC	8.416542907
1503	37.72316	4.4860476	HPS1	8.408996853

1504	19.943919	2.3760842	PLAC8	8.393607838
1505	2.0689737	0.2466926	CGNL1	8.386849713
1506	7.7861437	0.9296487	ZNF513	8.375360869
1507	0.0836274	0.01	NR1H4	8.3627408
1508	9.3560364	1.1210448	PKDCC	8.345818147
1509	0.0831602	0.01	SH3GL1P2	8.3160216
1510	257.44021	31.042374	LTA4H	8.293187054
1511	0.0827475	0.01	DKFZP434A062	8.2747469
1512	0.5969194	0.0721618	MYLK2	8.271961398
1513	0.7442839	0.0899767	LAPTM5	8.27196139
1514	0.6265299	0.0757414	PCDHB15	8.27196137
1515	0.6427124	0.0776977	NHLH1	8.271961366
1516	1.4273938	0.1725581	LINC00601	8.271961364
1517	0.762321	0.0921572	LOC100507156	8.271961363
1518	0.3596195	0.0434745	ZNF546	8.271961361
1519	0.5994951	0.0724732	LEMD1-AS1	8.271961359
1520	2.3749231	0.2871052	GAS5-AS1	8.271961359
1521	1.5351713	0.1855873	FOXN3-AS1	8.271961358
1522	4.8641751	0.5880316	NBR2	8.271961354
1523	1.6318395	0.1972736	TMEM130	8.271961351
1524	1.3076047	0.1580767	NDUFA4L2	8.271961341
1525	1.3461413	0.1627354	ZNF671	8.271961337
1526	2.2491683	0.2719027	ZNF83	8.271961336
1527	1.4586142	0.1763323	ANKRD63	8.271961332
1528	1.0597146	0.1281092	TMEM184A	8.271961323
1529	0.8767794	0.1059941	LOC100130705	8.271961321
1530	0.6497256	0.0785455	SLC7A7	8.271961321
1531	0.4556425	0.0550828	DKK2	8.271961315
1532	0.6779976	0.0819633	GPR37L1	8.271961313
1533	0.0826982	0.01	LOC286186	8.2698214
1534	0.0825672	0.01	LINC00488	8.2567155
1535	0.0824691	0.01	LINC00277	8.2469133
1536	0.0820148	0.01	ASPN	8.2014758
1537	0.0819825	0.01	OMD	8.1982494
1538	0.0819503	0.01	KCNMB2	8.1950256
1539	0.0814697	0.01	M1AP	8.1469703
1540	1.0114695	0.1245001	PTPRB	8.124247758
1541	32.711109	4.0263554	ZFP36	8.124247752
1542	21.104945	2.6085893	S1PR3	8.090558688
1543	93.231355	11.530197	BLVRB	8.085842214
1544	44.946127	5.5623086	PALLD	8.08048076
1545	0.0806812	0.01	PCSK1	8.0681185
1546	0.0806812	0.01	CDSN	8.0681185

1547	0.0806812	0.01	LDHAL6A	8.0681185
1548	3.9511816	0.4899073	ACBD4	8.065162312
1549	38.522044	4.7834268	TMEM173	8.0532316
1550	12.265413	1.5235798	OLFML3	8.050390957
1551	137.00839	17.042863	COL5A2	8.039047758
1552	11.293908	1.4086677	KIAA1462	8.017439462
1553	0.0798772	0.01	GPR17	7.9877156
1554	27.349609	3.4247103	PPP2R5B	7.985962685
1555	5.1335717	0.6442861	ATXN1	7.967845126
1556	0.079562	0.01	EPHA5	7.9562039
1557	0.0795418	0.01	CYP4F22	7.9541794
1558	2.3763949	0.2997737	LINC00085	7.927296299
1559	22.824707	2.879255	SOX2	7.927296292
1560	33.951752	4.2828918	ATF3	7.927296291
1561	0.079089	0.01	RANBP3L	7.9088994
1562	0.0788794	0.01	BLK	7.8879447
1563	24.004566	3.0525381	RASSF4	7.86380536
1564	3.9477473	0.5023625	C10orf114	7.858363286
1565	0.0783457	0.01	ALDH3B2	7.8345677
1566	0.0782868	0.01	LOC158434	7.8286814
1567	0.0779938	0.01	WDR49	7.7993825
1568	0.07779	0.01	IL1RAPL1	7.7790034
1569	2.8469877	0.3671181	ACCS	7.754963756
1570	1.0249156	0.1321625	ZSCAN31	7.75496375
1571	0.0775007	0.01	LOC100506874	7.7500744
1572	0.0774144	0.01	PARP15	7.7414376
1573	0.0773473	0.01	OTOGL	7.7347334
1574	0.0772422	0.01	C9orf152	7.7242217
1575	0.077185	0.01	C3orf27	7.7185
1576	29.599467	3.8401149	BAMBI	7.707963981
1577	364.4568	47.374605	FN1	7.693083744
1578	0.0767868	0.01	LOC100128264	7.6786846
1579	0.0767303	0.01	CASQ2	7.6730302
1580	2.099886	0.2744388	CDK15	7.651564238
1581	0.0763928	0.01	PPT2-EGFL8	7.6392779
1582	0.0762809	0.01	KNG1	7.628093
1583	0.0759473	0.01	MKRN3	7.594734
1584	4.36508	0.5756682	VAMP1	7.582631236
1585	1.7725842	0.233769	POU6F1	7.582631233
1586	4.9997699	0.6593714	CDC42EP5	7.582631232
1587	0.0757816	0.01	FAM188B	7.5781636
1588	0.0756716	0.01	SLAMF6	7.5671569
1589	38.963853	5.1553043	UBE2L6	7.558012304

1590	0.0752888	0.01	ALOX12P2	7.5288837
1591	29.771357	3.9577388	USP18	7.522314851
1592	0.0749638	0.01	GCM1	7.4963849
1593	0.07491	0.01	CHRM2	7.4909957
1594	0.074883	0.01	FCAMR	7.488304
1595	17.039658	2.2767643	C1orf85	7.484155502
1596	92.580688	12.40571	ECM1	7.462747735
1597	5.8418955	0.7831446	LHX6	7.459536572
1598	0.0744816	0.01	BLOC1S5	7.4481594
1599	0.0744816	0.01	CCDC33	7.4481594
1600	0.0743487	0.01	TAS1R1	7.4348734
1601	50.076834	6.7589167	HS6ST1	7.409002975
1602	88.291391	11.923632	PSMB9	7.404739593
1603	3.20516	0.4339695	THBS3	7.385679779
1604	0.073822	0.01	GPR61	7.3821998
1605	80.534709	10.909862	CSF1	7.38182638
1606	0.0737436	0.01	IL23R	7.3743631
1607	45.200629	6.1334184	KIAA1522	7.369565565
1608	0.0736394	0.01	CTAGE7P	7.3639399
1609	0.0735874	0.01	VIT	7.3587394
1610	0.073406	0.01	ABCC13	7.3405953
1611	0.0729946	0.01	CDH20	7.2994571
1612	0.0728415	0.01	FBXO40	7.2841489
1613	0.0728415	0.01	PHEX	7.2841489
1614	1106.3888	152.15789	TMSB4X	7.271320861
1615	0.072689	0.01	BMP3	7.2689048
1616	0.0725625	0.01	CNGA1	7.25625
1617	0.0723861	0.01	MORF4L2-AS1	7.2386072
1618	0.3583386	0.0495082	ZNF569	7.237966239
1619	0.4495521	0.0621103	INA	7.237966226
1620	0.6285207	0.0868366	LOC148709	7.237966215
1621	0.7954179	0.1098952	OLAH	7.237966213
1622	0.8606469	0.1189073	LEF1-AS1	7.237966204
1623	0.6670309	0.0921572	RABL2A	7.237966198
1624	0.4865899	0.0672274	LOC338758	7.237966185
1625	0.2551236	0.035248	KY	7.237966184
1626	6.4791098	0.8951561	LMF1	7.23796618
1627	0.9442049	0.1304517	RARRES1	7.237966178
1628	7.7958397	1.077076	BST1	7.237966176
1629	1.4429243	0.1993549	PTGES3L	7.237966174
1630	0.630422	0.0870993	ASIC3	7.237966173
1631	0.4843282	0.066915	RCSD1	7.237966173
1632	0.3156202	0.0436062	SLC16A12	7.237966172

1633	0.9318406	0.1287434	RFTN2	7.237966169
1634	0.5886992	0.0813349	LIPH	7.237966166
1635	0.7096177	0.098041	RPS6KA6	7.237966166
1636	0.666726	0.0921151	LINC00685	7.237966165
1637	0.693015	0.0957472	FAAH	7.237966165
1638	0.9128889	0.1261251	ARIH2OS	7.237966164
1639	0.5756892	0.0795374	LOC283663	7.237966163
1640	0.8598015	0.1187905	FHAD1	7.237966157
1641	0.0722856	0.01	ALPP	7.228564
1642	100.58022	13.960915	ITGB3	7.204414713
1643	0.0720358	0.01	CDC14C	7.2035776
1644	0.0720109	0.01	TNFSF14	7.2010885
1645	0.0716642	0.01	SLC13A4	7.1664202
1646	0.7016818	0.0981724	SAA2	7.147448096
1647	5.8122577	0.8140998	ARSD	7.139490446
1648	1.4245611	0.1999924	MIR143HG	7.123077832
1649	37.615475	5.2914578	DCAKD	7.108716784
1650	8.1914254	1.1650165	ARSA	7.031167143
1651	0.0702391	0.01	LOC150622	7.023913
1652	0.0701682	0.01	C5orf64	7.0168182
1653	0.0701446	0.01	LILRB1	7.0144564
1654	215.91727	30.942329	RHOB	6.978054827
1655	9.3786989	1.348924	FCHSD1	6.952726134
1656	51.285815	7.3809026	EMC10	6.948447532
1657	0.0692357	0.01	ACSBG1	6.9235714
1658	63.75216	9.226797	ARHGEF2	6.909457297
1659	0.0689836	0.01	LOC100131551	6.8983615
1660	63.199458	9.1702106	SDC3	6.891821874
1661	0.0688241	0.01	FRMD1	6.8824141
1662	0.0686202	0.01	ITIH3	6.8620184
1663	106.2479	15.499871	SUN2	6.854760302
1664	0.0684624	0.01	CLEC12B	6.8462385
1665	0.0684624	0.01	ERVW-1	6.8462385
1666	0.0683725	0.01	NEUROD2	6.8372539
1667	0.0682159	0.01	CHRM5	6.8215876
1668	131.46419	19.272653	SLC16A3	6.82128156
1669	0.0681267	0.01	ADAM20P1	6.8126675
1670	0.0680822	0.01	PIK3R6	6.8082163
1671	38.764141	5.6958159	GIPC3	6.805722297
1672	4.8791792	0.7172521	CPED1	6.802599793
1673	16.65597	2.4484713	TMEM45A	6.802599791
1674	0.0678826	0.01	ANKRD26P1	6.7882573
1675	63.013751	9.2967832	KIAA0930	6.778016604

1676	0.0677281	0.01	ZNF20	6.7728144
1677	8.9924802	1.3306625	SLC2A10	6.757896992
1678	0.0675306	0.01	ACTN3	6.7530622
1679	0.0674869	0.01	CPN2	6.7486885
1680	0.0673778	0.01	LHCGR	6.7377789
1681	0.8249676	0.1227453	PCDHB10	6.720968615
1682	1.6310617	0.2426825	SGCG	6.7209686
1683	9.4069219	1.3996378	LOC100506100	6.720968597
1684	18.543419	2.7590397	ING4	6.720968596
1685	4.1048386	0.6107511	RPL13AP20	6.720968595
1686	2.6251875	0.3905966	YJEFN3	6.720968595
1687	1.5908359	0.2366974	ACOT4	6.720968594
1688	11.241467	1.6725962	THNSL2	6.720968592
1689	0.740824	0.1102258	LOXL4	6.720968574
1690	0.0668805	0.01	10-Mar	6.6880456
1691	25.636446	3.8390066	IFI35	6.677885462
1692	0.0667733	0.01	C17orf72	6.677331
1693	0.0667305	0.01	KLK10	6.6730548
1694	3.6178303	0.5429304	LAMP3	6.663524422
1695	0.0665388	0.01	WNT16	6.6538793
1696	12.36113	1.8596242	MMP11	6.647111795
1697	0.0661796	0.01	LOC283038	6.6179581
1698	0.0659701	0.01	LOC284798	6.5970086
1699	0.065845	0.01	SH2D3C	6.5845024
1700	0.0658242	0.01	PCDHB18	6.5824226
1701	8.243358	1.2540755	DNAJB4	6.573255002
1702	0.065679	0.01	PCDHB3	6.567901
1703	26.474204	4.0328327	ASMTL	6.564667001
1704	0.0655757	0.01	CD4	6.5575677
1705	0.0655344	0.01	TMPRSS9	6.5534434
1706	4.9474267	0.7554896	SLC45A1	6.548636071
1707	0.8822617	0.1347245	ABI3BP	6.548636058
1708	1.7645234	0.269449	LOC100292680	6.548636055
1709	4.8790796	0.7475117	STAT5A	6.527094499
1710	0.0652676	0.01	TMEM232	6.5267617
1711	0.0652676	0.01	CDH26	6.5267617
1712	85.482115	13.113438	PDPN	6.518665192
1713	0.0651452	0.01	PPP1R1C	6.5145202
1714	0.0651045	0.01	SUSD2	6.5104499
1715	9.7390999	1.5003562	PDCD1LG2	6.491191892
1716	0.0648815	0.01	TMPRSS6	6.4881538
1717	0.0648008	0.01	IL2RA	6.480084
1718	1.3389842	0.2071939	HCN3	6.462469813

1719	6.9698829	1.0785169	DHRS1	6.462469802
1720	0.0644402	0.01	CCDC114	6.4440167
1721	0.0643506	0.01	LOC100130992	6.4350625
1722	5.2162975	0.8136772	SRR	6.410770045
1723	19.912144	3.1060456	TRIM16	6.410770043
1724	1.7095487	0.2666682	ZBTB20	6.410770036
1725	6.8339021	1.0681569	BTN3A3	6.397845106
1726	0.0638479	0.01	CX3CR1	6.3847886
1727	27.220415	4.2822775	SERINC2	6.356527674
1728	0.0634784	0.01	ITIH4	6.3478373
1729	125.96725	19.966693	GADD45B	6.308869071
1730	2.685901	0.4258353	MIRLET7BHG	6.307370531
1731	5.4713035	0.8687407	IFIT3	6.297970568
1732	46.457647	7.4032775	NNMT	6.275281023
1733	0.0624761	0.01	CACNA1I	6.2476117
1734	72.326015	11.586498	PTGR1	6.242267126
1735	60.053252	9.6233337	TAP1	6.240379292
1736	0.4494597	0.0724471	TMEM92	6.203971049
1737	0.5818506	0.0937868	C7orf57	6.203971047
1738	0.5103661	0.0822644	ZMIZ1-AS1	6.203971043
1739	0.3441775	0.055477	PYGM	6.203971039
1740	0.491895	0.0792871	IL1RL1	6.203971036
1741	0.5184067	0.0835605	BNIPL	6.203971036
1742	0.7350952	0.1184879	FLJ30403	6.203971035
1743	0.4648316	0.0749249	PTPRG-AS1	6.203971032
1744	0.6008635	0.0968514	PRCD	6.20397103
1745	0.542707	0.0874774	ADRA1A	6.20397103
1746	0.6713541	0.1082136	NPR3	6.203971027
1747	0.6150502	0.0991381	ZSCAN12P1	6.203971027
1748	1.9741033	0.3181999	GABRE	6.203971014
1749	0.8196637	0.1321192	N4BP2L1	6.203971012
1750	9.6992657	1.5633964	CNPY4	6.203971011
1751	0.8471524	0.13655	ZSWIM8-AS1	6.20397101
1752	1.2462429	0.2008783	EVI2A	6.203971009
1753	1.698909	0.2738422	TEX29	6.203971003
1754	1.2355702	0.199158	GIPR	6.203971002
1755	0.5940128	0.0957472	ST6GALNAC2	6.203970997
1756	0.7248678	0.1168393	BVES-AS1	6.203970997
1757	0.8255262	0.1330642	STX1B	6.203970994
1758	0.6308764	0.1016891	EFCAB13	6.203970993
1759	0.5033804	0.0811384	ZNF137P	6.203970992
1760	0.4874842	0.0785762	ZNF283	6.203970991
1761	0.2323294	0.0374485	PTGS1	6.203970978

1762	0.2221743	0.0358116	LRRTM2	6.203970978
1763	0.2374923	0.0382807	MRO	6.203970968
1764	0.0620052	0.01	LOC100379224	6.2005207
1765	47.199586	7.6206014	SLC12A7	6.193682502
1766	0.9061582	0.1469682	TSIX	6.165674891
1767	101.49236	16.549482	MRC2	6.132660997
1768	0.061204	0.01	EN2	6.1203965
1769	0.060989	0.01	TLR2	6.0989026
1770	21.839219	3.5857938	MRAS	6.090483735
1771	164.15148	27.018487	TMEM14C	6.075524405
1772	0.0606871	0.01	LINC00550	6.06871
1773	2.2426193	0.3702979	KIF17	6.056257413
1774	1.0413625	0.1719482	LOC389834	6.05625741
1775	48.275424	7.9861479	TGM2	6.044894831
1776	4.0209385	0.6666412	RASD1	6.031638486
1777	15.419694	2.5607723	LOC100506714	6.021501276
1778	0.0602137	0.01	HAPLN4	6.0213667
1779	5.9971079	0.9968644	PICK1	6.015971889
1780	32.334545	5.3820328	PCTP	6.007868479
1781	0.0600575	0.01	FGD3	6.0057493
1782	13.348615	2.2258183	MSRB2	5.997171977
1783	0.0599711	0.01	LINC00478	5.9971079
1784	0.0598849	0.01	COPG2IT1	5.9884914
1785	40.891628	6.8294385	CEBPD	5.987553417
1786	20.394422	3.4173244	NIPAL3	5.967950374
1787	0.960174	0.1614967	TLR7	5.945472232
1788	4.0076827	0.6740731	FAM212B	5.945472223
1789	0.0592718	0.01	LOC100506776	5.9271758
1790	9.7561542	1.6536262	TMEM140	5.899854785
1791	21.183844	3.5919418	GMPR	5.897602071
1792	0.0589032	0.01	PLG	5.8903194
1793	4.2424762	0.720373	MTUS1	5.889276826
1794	0.0588201	0.01	FBXL16	5.8820068
1795	0.0587372	0.01	GOLGA6L10	5.8737176
1796	176.68009	30.103164	TM9SF2	5.869153525
1797	238.56169	40.679989	PLOD1	5.864349834
1798	1.3882412	0.2369293	SLC7A5P2	5.859305967
1799	1.3511791	0.2306039	HSF4	5.859305958
1800	17.874831	3.0506738	TRIM16L	5.859305954
1801	38.273464	6.5320815	KDELC1	5.859305954
1802	4.0465922	0.6906265	TMEM86B	5.85930595
1803	21.012938	3.5920393	BTN3A2	5.849863076
1804	5.0879798	0.8703279	IQSEC2	5.846049605

1805	2.7835133	0.4762766	PIEZO2	5.844320513
1806	54.912681	9.4196415	PPIC	5.82959345
1807	7.1282894	1.2255874	FNDC1	5.816222824
1808	17.226263	2.9617612	B3GNT9	5.816222822
1809	10.763819	1.8506546	DENND2A	5.816222819
1810	8.0600266	1.3872517	GBP3	5.810068089
1811	0.0579693	0.01	LOC157381	5.7969263
1812	328.76662	56.719908	COL6A2	5.796317884
1813	0.6894938	0.1190759	DCLK1	5.790372966
1814	1.2600272	0.2176073	SEC31B	5.790372948
1815	109.18198	18.860693	MAGED1	5.788863462
1816	0.0578566	0.01	STK31	5.7856607
1817	8.2990951	1.4367964	TMEM47	5.776110941
1818	0.0577444	0.01	KCNJ13	5.7744389
1819	30.255182	5.2406807	CCDC53	5.773139691
1820	58.944171	10.213386	SCARA3	5.771266511
1821	0.0577124	0.01	CCDC129	5.7712407
1822	84.274406	14.658025	SCAMP3	5.749369686
1823	0.0574737	0.01	CCDC158	5.7473662
1824	94.890839	16.553642	VAMP5	5.73232409
1825	42.053711	7.3433913	SGCE	5.726742472
1826	24.036261	4.2040655	HSPA1A	5.717385049
1827	68.630058	12.012786	TCF7	5.713084417
1828	12.170411	2.1312438	FAM102A	5.710473319
1829	0.0569709	0.01	MS4A2	5.6970886
1830	7.4562886	1.3111172	VPS9D1	5.686973428
1831	0.5467194	0.0961354	CARD14	5.686973427
1832	8.231219	1.4473813	AAMDC	5.686973427
1833	1.3006494	0.2287068	CEP112	5.686973425
1834	0.9821742	0.172706	BCRP2	5.686973425
1835	0.9387365	0.1650679	SELL	5.686973421
1836	1.8427609	0.3240319	IL15	5.686973421
1837	1.1331658	0.1992564	GPBAR1	5.686973416
1838	67.662175	11.924187	SFTA1P	5.674363727
1839	209.64414	37.014386	HLA-E	5.663855649
1840	102.8287	18.160061	SHC1	5.662354491
1841	17.907008	3.1644681	RUSC1	5.658773558
1842	0.0564768	0.01	LINC00607	5.6476829
1843	0.4505239	0.080234	MASP1	5.615123827
1844	5.9054295	1.0520768	FAM229B	5.61311663
1845	79.326261	14.140857	BAD	5.609720914
1846	5.7655516	1.0285815	NRBP2	5.605342229
1847	0.0560364	0.01	LINC00485	5.6036435

1848	0.0558862	0.01	LOC401557	5.5886163
1849	0.9382669	0.1680406	GPRASP1	5.583573894
1850	0.0557516	0.01	AOX2P	5.5751605
1851	18.88396	3.3911355	ZCCHC24	5.568624581
1852	0.0556771	0.01	FLJ46361	5.5677131
1853	28.00136	5.0327049	GAS6	5.563878762
1854	9.639252	1.744531	ABCD1	5.52541168
1855	0.0552491	0.01	HOTTIP	5.5249072
1856	0.478049	0.0866872	MYO5C	5.514640908
1857	1.8141415	0.3289682	RHBDL2	5.514640908
1858	1.6278561	0.2951881	ZNF320	5.514640906
1859	1.637449	0.2969276	SEMA6D	5.514640903
1860	1.6377171	0.2969762	GBAP1	5.514640902
1861	3.8020433	0.6894453	SYCE1L	5.514640901
1862	2.8067273	0.5089592	APOLD1	5.514640898
1863	0.9578834	0.1736982	MROH8	5.514640895
1864	0.8071634	0.1463674	TOX	5.514640877
1865	0.2142512	0.0388513	RYR3	5.514640869
1866	178.1177	32.309856	SERPINH1	5.512797771
1867	3.1111133	0.5668806	HLA-L	5.488128201
1868	0.0548709	0.01	FLJ36000	5.4870853
1869	0.0547555	0.01	ESRP1	5.4755518
1870	70.192333	12.821267	SYDE1	5.474679735
1871	49.291411	9.0168087	TCIRG1	5.4666138
1872	0.0546407	0.01	ELAVL2	5.4640666
1873	20.963997	3.8390066	TNFSF9	5.460786983
1874	14.249002	2.6104089	POMT1	5.458532633
1875	101.77821	18.735434	UBAC2	5.432391283
1876	0.0541579	0.01	CNGA3	5.4157874
1877	38.007286	7.021134	SCPEP1	5.413268823
1878	0.0540736	0.01	NEFL	5.407356
1879	131.09927	24.256103	MALAT1	5.404795358
1880	67.119932	12.442674	SLC44A2	5.394333287
1881	73.060663	13.544399	FBLIM1	5.394160634
1882	13.523851	2.5076702	HDAC6	5.39299441
1883	2.115731	0.3934944	GAL3ST4	5.376774878
1884	2.8147465	0.5235009	TMEM17	5.376774873
1885	0.0536007	0.01	SLC26A4-AS1	5.3600694
1886	4.3268567	0.8119374	RGMA	5.329052024
1887	69.816661	13.129135	FOXF1	5.317689439
1888	0.053136	0.01	TLR9	5.3136028
1889	4.3599406	0.8214149	KIAA0513	5.307841863
1890	0.0530279	0.01	LILRA6	5.3027863

1891	0.0530279	0.01	ANKRD20A3	5.3027863
1892	8.1582236	1.5395125	ABCG1	5.299225239
1893	0.052947	0.01	ENPP6	5.2947027
1894	36.539291	6.9014482	RBPMS	5.294438223
1895	43.327238	8.1841302	EPHX1	5.294055263
1896	8.8876257	1.6795654	TCTN1	5.291622333
1897	2.5327284	0.4792419	WDR66	5.284864192
1898	17.143786	3.247064	IRS2	5.279780638
1899	16.485334	3.1285039	MIB2	5.269398456
1900	7.25611	1.3775786	UTRN	5.267293034
1901	0.052666	0.01	ZNF334	5.2666035
1902	242.13854	46.153154	ICAM1	5.246413657
1903	2.913431	0.5558619	ABCA1	5.241285858
1904	0.052388	0.01	KCNK3	5.2388009
1905	25.639996	4.9017359	LAMTOR2	5.230799087
1906	0.0521912	0.01	ANKRD22	5.219121
1907	4.0968968	0.7866135	PBX1	5.208271958
1908	0.0519052	0.01	LOC100128946	5.190523
1909	0.0517249	0.01	LOC100652824	5.172487
1910	0.051712	0.01	CTCFL	5.1712035
1911	0.0847773	0.016398	NOS1	5.169975952
1912	0.2287088	0.0442379	SHC4	5.169975903
1913	0.5171203	0.1000237	LOC400548	5.169975869
1914	0.3206146	0.0620147	CHST5	5.169975865
1915	0.3737437	0.0722912	ZNF396	5.169975864
1916	0.1599873	0.0309455	MGAM	5.169975864
1917	0.6172971	0.1194004	GFRA2	5.169975862
1918	0.2525442	0.0488482	LINC00282	5.169975861
1919	0.3221012	0.0623023	RASGRP4	5.169975859
1920	0.6380879	0.1234218	FAM66C	5.169975856
1921	1.0371574	0.2006117	CMPK2	5.169975854
1922	1.4052562	0.271811	TM6SF2	5.169975852
1923	0.5772839	0.1116609	LINC00092	5.169975849
1924	0.4132451	0.0799317	FREM1	5.169975849
1925	0.1805575	0.0349243	ZNF43	5.169975848
1926	0.3776722	0.0730511	TTC39A	5.169975846
1927	1.7751235	0.3433524	LOC729683	5.169975845
1928	6.4395365	1.2455641	MSTO1	5.169975844
1929	1.1922168	0.2306039	LINC00439	5.169975844
1930	0.4166323	0.0805869	HOGA1	5.169975844
1931	1.481513	0.2865609	PCDHB9	5.169975843
1932	3.3255239	0.6432378	SLC51B	5.169975843
1933	5.7290384	1.1081364	SSPN	5.169975842

1934	41.835165	8.0919459	ST6GALNAC6	5.169975842
1935	7.7761008	1.5040884	SNORA64	5.169975841
1936	0.3044996	0.0588977	PCDHB12	5.169975841
1937	0.2219377	0.0429282	DNAJC27-AS1	5.16997584
1938	0.7667384	0.148306	LDB3	5.169975839
1939	0.6683756	0.1292802	RHBDL1	5.169975838
1940	1.0430405	0.2017496	LOC541473	5.169975834
1941	0.4007683	0.0775184	LRRN4CL	5.169975831
1942	0.8700714	0.1682931	CNNM1	5.169975831
1943	0.7948112	0.153736	VN1R2	5.169975831
1944	0.6237638	0.1206512	ZNF506	5.169975824
1945	0.6500296	0.1257317	ATP6V1G2	5.169975823
1946	0.6837254	0.1322492	MLANA	5.169975822
1947	0.4887418	0.0945346	GNA15	5.169975814
1948	0.2712829	0.0524728	MYOZ3	5.169975813
1949	0.3538192	0.0684373	SLC6A16	5.169975803
1950	0.0581602	0.0112496	MUC5B	5.169975717
1951	0.0516736	0.01	PTPRQ	5.1673568
1952	0.0516608	0.01	C7	5.1660759
1953	35.618636	6.9063617	F3	5.157366145
1954	59.74306	11.610024	LEPREL1	5.145817074
1955	239.92211	46.632639	RAB34	5.144939638
1956	111.64518	21.707314	PVRL2	5.14320574
1957	19.603845	3.8292223	ANXA4	5.119537054
1958	0.0510533	0.01	LINC00689	5.1053283
1959	11.824716	2.3215836	KCNS3	5.093383608
1960	0.0509161	0.01	MTTP	5.0916076
1961	8.9162948	1.7538609	MAFIP	5.083809578
1962	103.38314	20.375919	ECH1	5.073790243
1963	0.0506439	0.01	MEIS1-AS3	5.0643864
1964	1.0448471	0.2066922	NHS	5.055087491
1965	13.387045	2.6499529	MXD3	5.051804966
1966	12.755054	2.5254188	MSC	5.050668709
1967	5.1342365	1.0175073	TENC1	5.045896421
1968	0.0504111	0.01	CDH5	5.0411103
1969	5.5233303	1.0957409	CTSO	5.040726447
1970	51.481112	10.213034	PRAF2	5.040726445
1971	0.0503624	0.01	SLC9C1	5.0362373
1972	37.175526	7.4000941	ING1	5.023655771
1973	9.6731509	1.9260545	SLC26A11	5.022262247
1974	0.0501563	0.01	GOLGA6L4	5.0156318
1975	2.2355131	0.4461301	ATOH8	5.010899664
1976	328.15302	65.607229	ACTN4	5.00178143

1977	37.589682	7.5214815	CXXC5	4.997643315
1978	2.871062	0.5744832	TMEM71	4.997643313
1979	7.84508	1.5775269	LYPD1	4.973024381
1980	0.0497018	0.01	COL21A1	4.9701765
1981	33.448947	6.7348226	GPSM1	4.966566957
1982	2.2398513	0.4512939	HSD3B7	4.963176806
1983	25.662185	5.1818797	TP53I13	4.952292649
1984	172.74458	34.955447	PXDN	4.941850113
1985	5.7591122	1.1657652	TMEM150A	4.940199137
1986	0.0493137	0.01	EDAR	4.9313654
1987	31.112186	6.3316393	OPTN	4.913764651
1988	6.654774	1.3549435	VANGL2	4.911477051
1989	3.7481924	0.7631497	ARHGEF6	4.911477051
1990	37.121619	7.5680956	NOMO1	4.905014581
1991	262.3084	53.536147	SSR2	4.899650309
1992	25.414573	5.1983185	IRF1	4.888998894
1993	0.0488284	0.01	FGL2	4.8828374
1994	0.0487029	0.01	HSPE1-MOB4	4.8702851
1995	4.7002988	0.965975	SHANK2	4.865859614
1996	1.9656385	0.4044709	DIAPH2	4.859777295
1997	124.27972	25.580796	GSN	4.858320959
1998	59.978076	12.353742	CYB561A3	4.855053456
1999	27.189559	5.6180585	GBA	4.839671831
2000	0.0483189	0.01	CDHR2	4.831892
2001	45.618954	9.4424678	GPC4	4.831253287
2002	0.0482965	0.01	CTAGE1	4.8296524
2003	0.5308575	0.1100152	ATP1A2	4.825310803
2004	0.7498311	0.1553954	ID4	4.825310793
2005	6.5368028	1.3546905	DUSP10	4.825310784
2006	2.8594508	0.5925941	DYNC1I1	4.825310782
2007	0.5369144	0.1112704	ZNF772	4.825310767
2008	101.56766	21.063198	PEPD	4.822043818
2009	8.385991	1.7400238	SNED1	4.819469005
2010	0.0479631	0.01	CDH12	4.7963061
2011	0.0477981	0.01	IGSF22	4.779805
2012	0.0477324	0.01	ITK	4.7732364
2013	1.5834782	0.3329168	NPL	4.756377778
2014	0.9852392	0.2071406	ARHGEF37	4.756377764
2015	9.6214593	2.0256065	NGEF	4.749915305
2016	90.59665	19.119857	C5orf15	4.738354006
2017	0.0473635	0.01	GRIN1	4.7363523
2018	12.090616	2.5651544	GPR124	4.713406548
2019	18.073585	3.8347599	FLAD1	4.713094256

2020	2.0392314	0.432919	BOLA3-AS1	4.710422439
2021	0.0470853	0.01	KIF4B	4.7085291
2022	9.7272751	2.0667919	CLGN	4.706460767
2023	36.776382	7.8292726	CSRP2	4.697292336
2024	2.4137568	0.5149408	ROR1	4.687444765
2025	0.0468419	0.01	MAATS1	4.6841875
2026	3.7093762	0.7920281	TGFA	4.683389883
2027	4.3457716	0.9279116	HUNK	4.683389881
2028	11.954522	2.5568673	BCAS3	4.675456416
2029	11.656384	2.4961979	TMEM44	4.669655601
2030	33.432379	7.1711781	ARL4D	4.662048391
2031	17.269242	3.704462	JAZF1	4.661740929
2032	0.592044	0.1272398	ZNF818P	4.65297828
2033	0.355968	0.0765033	ADAMTS14	4.652978279
2034	0.4436129	0.0953396	PAK6	4.652978268
2035	1.0003176	0.2149844	C8orf31	4.652978265
2036	2.3817086	0.5118675	CBR3-AS1	4.65297826
2037	5.6885479	1.2225606	HLX	4.652978258
2038	4.3720175	0.939617	PTGER4	4.652978258
2039	1.6021602	0.34433	TMEM255A	4.652978257
2040	0.6941508	0.1491842	KLHDC1	4.652978253
2041	1.123784	0.2415193	C18orf32	4.652978249
2042	0.0464866	0.01	SGK3	4.6486616
2043	0.0464762	0.01	CILP	4.6476249
2044	9.8699319	2.1377793	KLF11	4.616908659
2045	92.672724	20.088559	CD70	4.613209212
2046	0.7024095	0.1523415	CDKN2B-AS1	4.610755234
2047	2.191482	0.4793952	PARD3B	4.57134706
2048	14.334357	3.1364433	LOC100506990	4.570258643
2049	22.56166	4.9473185	LZTS1	4.560381677
2050	31.374135	6.8871927	TMCO3	4.555431544
2051	2.0708171	0.4551668	SLC22A4	4.549578743
2052	0.3492906	0.0767743	DNAH1	4.549578731
2053	0.9039952	0.1992017	HMCN1	4.538089902
2054	13.443735	2.9682425	AGFG2	4.529190103
2055	0.045255	0.01	PCDHGA3	4.5255049
2056	11.657937	2.5770636	TTLL1	4.523728863
2057	4.1608571	0.9197848	KRT7	4.523728863
2058	41.250087	9.121902	FKBP9	4.522092794
2059	6.7894391	1.5037144	TMCC2	4.515112237
2060	88.287001	19.561452	MAGED2	4.513315403
2061	48.852942	10.832188	TCEA2	4.509978926
2062	6.945588	1.5406501	COL4A5	4.508218935

2063	6.044069	1.3444317	PPP1R3F	4.495631168
2064	104.92316	23.339476	EHD2	4.495523359
2065	22.884091	5.0977555	TUBB2A	4.489052194
2066	93.510368	20.843981	SAT1	4.486204843
2067	1.8049257	0.4028271	ICAM4	4.480645729
2068	0.6450461	0.1439627	GPR156	4.480645729
2069	1.2264344	0.2737182	PODXL2	4.480645729
2070	1.0476386	0.2338142	SEC14L1P1	4.480645727
2071	356.61502	79.670489	MXRA7	4.476124392
2072	153.71547	34.376001	CAPN1	4.471592613
2073	8.7526343	1.9588128	STPG1	4.468336264
2074	4.5823982	1.02765	CCBE1	4.459104162
2075	0.0445108	0.01	ELAVL3	4.4510786
2076	157.29741	35.344666	BOK	4.450386057
2077	0.0441805	0.01	CDKL2	4.4180517
2078	5.9622566	1.3514608	SLC35B3	4.41171272
2079	18.784952	4.2674983	SHISA4	4.401865146
2080	0.9317006	0.2120161	KIAA1656	4.394479469
2081	1.8480916	0.4205485	ADPRHL1	4.394479468
2082	1.759956	0.4004925	DEPDC7	4.394479467
2083	0.5683015	0.1293217	PCDH9	4.394479453
2084	37.258979	8.4887782	LAMB2	4.389203981
2085	5.8993497	1.3462165	MPPE1	4.382170001
2086	28.68199	6.5660064	AJUBA	4.36825495
2087	61.782153	14.157399	TMEM98	4.363948112
2088	2.330472	0.5348123	RNF165	4.35755107
2089	14.952052	3.431297	RBM24	4.357551066
2090	1.6131181	0.3714483	DRD2	4.342779709
2091	40.671202	9.3801356	UBE2Q1	4.335886407
2092	0.0432903	0.01	C4orf40	4.3290299
2093	0.0432723	0.01	HRNR	4.3272321
2094	27.624105	6.3843129	CLEC2B	4.32687209
2095	320.98505	74.195303	CD99	4.32621788
2096	25.039239	5.8017528	TRAFD1	4.31580592
2097	106.52103	24.6946	PLD3	4.313535399
2098	71.558865	16.593297	TUBA1A	4.312516435
2099	11.394177	2.6446956	DDX19B	4.308313204
2100	2.8299769	0.6568642	TNFRSF1B	4.308313204
2101	0.7385863	0.1714328	KIAA1324	4.308313199
2102	2.0030709	0.4649316	PALMD	4.308313199
2103	145.33123	33.752032	FURIN	4.305851309
2104	73.259609	17.02995	POR	4.301810088
2105	47.5843	11.08293	SOCS3	4.293476626

2106	20.105075	4.6933963	LOC728392	4.28369427
2107	8.346987	1.9516077	HEXDC	4.276980015
2108	12.334863	2.8861267	NKX2-2	4.273846697
2109	17.610527	4.1259497	LARGE	4.268235871
2110	24.284826	5.7027686	APOL2	4.25842747
2111	12.436744	2.9256946	CLCF1	4.250869028
2112	9.4379211	2.2202333	SERTAD4-AS1	4.250869027
2113	52.493329	12.366844	PLXNB2	4.244682729
2114	157.05379	37.015271	QSOX1	4.242945692
2115	40.89733	9.6419495	SPOCD1	4.241603837
2116	10.911989	2.5754906	NEURL1B	4.236858251
2117	3.6921171	0.872845	DYRK1B	4.229980236
2118	20.487116	4.8433125	TMBIM4	4.229980235
2119	4.5679384	1.0798959	SLC22A23	4.229980233
2120	19.962863	4.7272292	FBXL7	4.222952231
2121	8.8645052	2.1028267	SH3BGR	4.215518763
2122	8.7167356	2.0687488	KIF5A	4.213530311
2123	46.260508	10.987126	ATF5	4.210428324
2124	14.260671	3.3914301	RGS12	4.204913687
2125	22.146985	5.2669298	SNAI1	4.204913685
2126	126.39929	30.064392	MVP	4.204285595
2127	35.881284	8.5391366	RETSAT	4.201980365
2128	5.7072416	1.3593049	PIGN	4.198647049
2129	12.934172	3.08191	SYNGR1	4.196803919
2130	13.445129	3.2036591	C19orf66	4.196803917
2131	27.914061	6.6566261	LHFP	4.193424849
2132	42.56464	10.173914	STK40	4.183703526
2133	26.619862	6.3768084	DAPK1	4.174480495
2134	43.603078	10.462209	FARP1	4.167674012
2135	0.0416383	0.01	HYMAI	4.1638262
2136	158.04975	38.018069	DYNLT1	4.157227149
2137	0.0414148	0.01	LOC390660	4.1414845
2138	0.0432006	0.0104451	XIST	4.135980863
2139	0.1810202	0.0437672	PCDHGA6	4.135980718
2140	0.2344861	0.0566942	ANGPTL1	4.135980706
2141	0.3591547	0.0868366	GRK4	4.135980696
2142	0.2817161	0.0681135	SNAP25-AS1	4.135980694
2143	0.2231855	0.0539619	PCA3	4.135980693
2144	0.2894438	0.0699819	CXCR2	4.135980692
2145	0.4550207	0.1100152	CCDC65	4.135980692
2146	0.249655	0.0603617	CPNE5	4.135980691
2147	0.378049	0.0914049	USP51	4.13598069
2148	0.5158403	0.1247202	CD40	4.135980689

2149	0.3023569	0.073104	TAT	4.135980687
2150	0.77544	0.1874864	LOC100505702	4.135980686
2151	0.2582398	0.0624374	CCDC62	4.135980685
2152	0.460043	0.1112295	CLK2P	4.135980684
2153	0.3505458	0.0847552	SLC23A1	4.135980684
2154	0.3709826	0.0896964	ZNF570	4.135980684
2155	0.6502325	0.1572136	TMIGD2	4.135980683
2156	0.3380365	0.0817307	CCDC88B	4.135980681
2157	0.643705	0.1556354	ZNF583	4.13598068
2158	0.7626697	0.1843988	ROPN1L	4.13598068
2159	0.4412906	0.1066955	FAM228A	4.13598068
2160	0.7675856	0.1855873	PEX11G	4.135980679
2161	1.2864167	0.3110306	IDO1	4.135980679
2162	0.2647183	0.0640038	PLEKHG6	4.135980679
2163	0.4389668	0.1061337	GOLGA8I	4.135980678
2164	0.5003589	0.1209771	PODNL1	4.135980678
2165	0.4248716	0.1027257	CHRNA10	4.135980678
2166	0.795165	0.1922555	SPDYE6	4.135980678
2167	0.4644	0.1122829	TEKT3	4.135980678
2168	0.624418	0.1509722	JMJD1C-AS1	4.135980678
2169	2.1936789	0.5303891	EME2	4.135980677
2170	0.397709	0.0961583	LDHD	4.135980677
2171	1.4200988	0.3433524	C5orf46	4.135980677
2172	2.1821937	0.5276122	KDM5B-AS1	4.135980676
2173	0.676073	0.1634614	SPATA4	4.135980675
2174	0.341219	0.0825001	FLJ39639	4.135980675
2175	6.9730631	1.6859516	N6AMT1	4.135980674
2176	6.0260096	1.4569724	DGCR6	4.135980674
2177	7.0551914	1.7058086	ENO3	4.135980674
2178	10.116481	2.445969	HIST1H2BD	4.135980673
2179	1.6806411	0.4063465	LOC100130417	4.135980673
2180	0.5602137	0.1354488	RPL13AP3	4.135980673
2181	1.6848873	0.4073731	IL24	4.135980673
2182	0.3895318	0.0941812	FLJ36777	4.135980673
2183	8.33598	2.0154785	MIR5047	4.135980673
2184	5.7969263	1.4015845	SPATA24	4.135980672
2185	2.9249053	0.7071854	SNCG	4.135980672
2186	0.9231429	0.2231981	C11orf91	4.135980672
2187	10.006657	2.4194157	SPPL2B	4.135980672
2188	0.2644664	0.0639428	ATP6V0A4	4.135980671
2189	0.9814733	0.2373012	ELMO3	4.135980671
2190	2.1651896	0.5235009	GUSBP4	4.135980671
2191	1.6485129	0.3985785	LOC91948	4.135980671

2192	0.5958528	0.1440657	UBAP1L	4.135980671
2193	1.132606	0.2738422	ABCC6P2	4.135980668
2194	1.0019207	0.242245	MAP1LC3B2	4.135980667
2195	0.5870408	0.1419351	STAT4	4.135980666
2196	0.8462924	0.2046171	OR2A9P	4.135980666
2197	0.5498668	0.1329471	C12orf77	4.135980665
2198	0.7697119	0.1861014	GNRH1	4.135980665
2199	0.1823866	0.0440975	SCN3A	4.135980664
2200	0.59799	0.1445824	ZNF547	4.135980664
2201	0.4897756	0.1184182	SHANK2-AS3	4.135980663
2202	0.5296048	0.1280482	EGFLAM-AS4	4.135980659
2203	0.1665198	0.0402613	ATP8A2	4.135980656
2204	0.2399534	0.0580161	DNAH11	4.135980655
2205	0.182247	0.0440638	LDLRAD4	4.13598065
2206	0.1704699	0.0412163	N4BP2L2-IT2	4.135980645
2207	0.1291199	0.0312187	SEC14L5	4.135980634
2208	0.1297429	0.0313693	COL11A2	4.135980623
2209	0.0996531	0.0240942	IMPG2	4.135980617
2210	12.819276	3.1058698	MYLK	4.12743526
2211	51.637216	12.528532	DAB2	4.12156959
2212	14.932968	3.6282881	TSPYL2	4.11570626
2213	8.0855357	1.9649512	SNPH	4.114878732
2214	43.206113	10.51277	BHLHE41	4.109869685
2215	0.0408947	0.01	ANKRD20A19P	4.0894721
2216	2693.9562	658.82795	FTL	4.08901324
2217	8.9374155	2.1863162	CBX7	4.087887875
2218	2.6858531	0.6585335	STX11	4.078536497
2219	3.5020734	0.8593713	RNF207	4.075157429
2220	22.164633	5.4427129	TBX3	4.072350201
2221	65.273735	16.035357	FOXF2	4.070613163
2222	43.287389	10.660382	ARL8A	4.060585192
2223	11.640188	2.8726003	DHRSX	4.052143228
2224	211.2298	52.151766	ANXA1	4.050290465
2225	9.3736177	2.3190652	CXCR4	4.041981113
2226	251.84755	62.335139	VAT1	4.040217932
2227	3.6459908	0.9030307	EEPD1	4.037504942
2228	10.456842	2.5930891	BRSK1	4.032581156
2229	19.894162	4.9414443	FZD2	4.025981188
2230	40.932055	10.173827	TNFAIP2	4.023270302
2231	119.63455	29.739709	ECE1	4.022720895
2232	9.3793174	2.3325297	CCDC22	4.021092321
2233	19.618217	4.8853199	KRCC1	4.015748678
2234	16.820559	4.1916363	SHROOM1	4.012886012

2235	145.51732	36.285395	LAPTM4A	4.010355092
2236	2.3180425	0.5785371	NRIP2	4.006731277
2237	16.569221	4.1400597	FAM114A1	4.002169533
2238	20.778948	5.1990938	ALX4	3.996647991
2239	41.994862	10.508918	CHPF2	3.996116592
2240	8.7182933	2.1859853	NICN1	3.988267079
2241	27.983923	7.0262	STX12	3.982796204
2242	1060.734	266.50814	CTGF	3.980118668
2243	15.886547	4.0013338	H6PD	3.97031294
2244	37.842636	9.5340538	IL10RB	3.96920726
2245	0.0396197	0.01	PCDHA3	3.9619677
2246	0.0396197	0.01	PCDHA1	3.9619677
2247	29.562102	7.4695049	SDF2	3.957705644
2248	13.458285	3.4018595	SOX13	3.956155429
2249	59.154265	14.984601	STIM1	3.947670296
2250	26.539255	6.7375116	ADAM15	3.939029214
2251	26.570801	6.751408	FZD7	3.935594013
2252	20.614247	5.2387888	SGSH	3.934926058
2253	1.2666636	0.3223734	ANKDD1A	3.929181641
2254	4.2168163	1.0732047	PAOX	3.92918164
2255	216.03572	54.985905	ANXA6	3.928929139
2256	5.646674	1.4402705	KBTBD11	3.920565012
2257	320.51228	81.763743	SLC7A5	3.919980598
2258	0.0390334	0.01	GOLGA8DP	3.9033433
2259	35.336764	9.0569734	TWIST1	3.901608436
2260	16.809678	4.3090891	ADRB2	3.900981773
2261	13.935529	3.5735434	DIP2C	3.899638922
2262	17.156666	4.4092218	KCTD13	3.891087082
2263	19.784536	5.0862719	TOM1L2	3.889791348
2264	0.0388805	0.01	PCDHA2	3.8880504
2265	6.1563649	1.5834997	LPAR1	3.887821833
2266	66.474497	17.110179	PSMB8	3.885084789
2267	1.0364697	0.2673048	CA5B	3.877481884
2268	0.99112	0.2556092	AGMAT	3.877481882
2269	4.3326299	1.1173824	LOC728431	3.877481882
2270	1.5521313	0.4002936	VSNL1	3.87748188
2271	1.0827823	0.2792488	EXD3	3.877481878
2272	0.9683992	0.2497495	MCF2L	3.877481877
2273	1.0790447	0.2782849	RGL4	3.877481874
2274	900.30375	232.72174	TIMP1	3.868584714
2275	4.3938115	1.1364551	ELOVL2	3.866242805
2276	16.561859	4.2863321	OSTM1	3.863876681
2277	0.038614	0.01	LOC399829	3.8613952

2278	780.38575	202.42605	CYR61	3.855164632
2279	4.4758405	1.1613548	TM6SF1	3.853981991
2280	311.33209	80.883093	HINT1	3.849161501
2281	1.861349	0.4846565	PSMG3-AS1	3.840553487
2282	4.2068222	1.0953687	PPP1R3C	3.840553481
2283	34.935097	9.0998357	TEAD2	3.839090972
2284	33.367897	8.6957365	SIRT2	3.837270958
2285	11.018824	2.872636	CLK2	3.83578853
2286	4.2795467	1.1160933	CRISPLD1	3.834398749
2287	17.162312	4.478841	RAB20	3.831864448
2288	59.233883	15.471515	GNG11	3.828576704
2289	237.01118	61.937996	COL4A2	3.826587834
2290	189.59446	49.546957	TAPBP	3.826561238
2291	8.3785672	2.1936572	ARHGEF10L	3.81945154
2292	74.236009	19.45474	EXOC7	3.815831542
2293	4.463002	1.1705138	FAS	3.812857183
2294	367.05956	96.302404	CTSD	3.811530577
2295	44.401535	11.656685	PINK1	3.809104782
2296	189.20127	49.801319	GRN	3.79912162
2297	9.7264611	2.5603979	BTN2A2	3.798808336
2298	127.31143	33.565879	CD9	3.792882278
2299	0.2722882	0.0718189	ZNF737	3.791315635
2300	0.774196	0.2042025	MX2	3.791315624
2301	3.4317283	0.905155	RWDD3	3.791315618
2302	31.740847	8.3719875	DNAJC4	3.791315618
2303	20.361757	5.370631	DDAH2	3.791315617
2304	3.1994341	0.8438849	FN3K	3.791315617
2305	3.4113013	0.8997672	ADIRF	3.791315617
2306	1.5842395	0.4178601	ANXA10	3.791315617
2307	10.942217	2.8861267	TRAPPC6A	3.791315616
2308	1.6635664	0.4387834	ASMT	3.791315616
2309	1.805035	0.4760973	AREG	3.791315616
2310	0.5349812	0.141107	STAG3	3.791315614
2311	0.6178961	0.1629767	PDK4	3.791315612
2312	0.3418933	0.090178	TNFSF15	3.791315605
2313	396.53498	104.72786	SQSTM1	3.786336872
2314	3.6386606	0.9631031	ALPK1	3.778059268
2315	38.13428	10.104752	ARHGEF7	3.773895725
2316	8.4201818	2.2328533	PANX2	3.771041203
2317	21.129969	5.6096816	HIBADH	3.766696684
2318	12.772002	3.3924756	CRYZ	3.764802921
2319	11.500518	3.0556071	PHF11	3.763742412
2320	1.2197805	0.3244112	ABCA7	3.75998243

2321	23.248308	6.193187	TMEM120A	3.753852024
2322	19.134975	5.1009755	IRF9	3.751238286
2323	1.9939246	0.531964	LRRC37B	3.748232484
2324	18.592232	4.9648937	SGSM3	3.744739258
2325	7.5680952	2.0232143	FGF2	3.74062958
2326	7.330069	1.9631284	APOBEC3G	3.733871443
2327	9.9675993	2.6695079	BTG2	3.733871442
2328	1.5962724	0.4275113	ANK1	3.733871442
2329	21.077442	5.6464022	A4GALT	3.732897812
2330	14.545185	3.9009261	2-Mar	3.728649245
2331	19.793289	5.311803	DNASE2	3.726284476
2332	0.711936	0.1912582	SMPD3	3.722382613
2333	3.3265069	0.8936499	ACBD7	3.722382607
2334	13.610238	3.6563243	CAMKK1	3.722382607
2335	8.5448542	2.2955336	ATAT1	3.722382606
2336	11.026429	2.9706599	FAM69A	3.711777527
2337	8.9908348	2.4225541	TMEM19	3.711304087
2338	13.87949	3.7397878	SMAD6	3.711304085
2339	20.664614	5.5716352	NIPSNAP3A	3.708895713
2340	0.0370752	0.01	TRPM8	3.7075165
2341	37.397392	10.094698	RARA	3.704656976
2342	18.13062	4.8964349	CALCOCO1	3.702820536
2343	0.0369437	0.01	CRB2	3.6943716
2344	4.0830623	1.10567	EFNA4	3.692839887
2345	45.924845	12.484203	B3GAT3	3.678636658
2346	43.461387	11.819035	TXLNA	3.677236339
2347	62.308634	16.954532	LRRC41	3.675043068
2348	5.7640688	1.5702988	PLA2G4C	3.670682848
2349	54.847938	14.959914	EXT2	3.666327192
2350	11.285421	3.0801332	SUSD1	3.663939402
2351	17.313747	4.7340512	MSRB3	3.657279208
2352	18.986089	5.1932574	GRIPAP1	3.655911488
2353	34.924723	9.5551901	CCDC92	3.655052688
2354	190.63164	52.169204	NFE2L1	3.654102989
2355	25.38101	6.9499258	SLC50A1	3.651982935
2356	0.0364717	0.01	ESRRG	3.6471736
2357	50.603708	13.890981	COLEC12	3.642918163
2358	160.32166	44.016715	9-Sep	3.642290357
2359	10.209774	2.8073427	BTD	3.636810591
2360	85.375906	23.477439	RRAS	3.636508432
2361	28.377804	7.82606	MAPRE3	3.626065248
2362	0.2846432	0.0786528	ABCC6	3.618983111
2363	0.6670309	0.1843144	A2M-AS1	3.618983099

2364	0.9066479	0.2505256	1-Sep	3.618983097
2365	0.4040988	0.1116609	GRHL1	3.618983096
2366	0.9363264	0.2587264	PPP1R32	3.618983095
2367	0.9060848	0.25037	AKR1B10	3.618983095
2368	1.7101952	0.4725624	RPS14P3	3.618983093
2369	1.8465779	0.5102477	VAMP8	3.618983093
2370	1.3573357	0.37506	CSGALNACT1	3.618983092
2371	1.9373128	0.5353196	FLJ39051	3.618983091
2372	3.0950421	0.8552242	ARHGAP33	3.618983091
2373	0.5272123	0.1456797	LINC00670	3.618983091
2374	10.664831	2.9469137	TBC1D25	3.61898309
2375	3.4244049	0.946234	FAHD2CP	3.61898309
2376	0.4671138	0.1290732	CCDC176	3.618983089
2377	0.6460569	0.1785189	ADAMTS9-AS2	3.618983088
2378	2.492604	0.6887581	MTMR9LP	3.618983088
2379	3.1816718	0.8791618	PTPRCAP	3.618983088
2380	0.952839	0.2632892	RINL	3.618983086
2381	1.2619347	0.3486987	LOC100505648	3.618983086
2382	0.2751408	0.0760271	MTMR7	3.618983083
2383	0.9751314	0.269449	C14orf64	3.618983083
2384	0.4399266	0.1215608	LOC285696	3.618983081
2385	0.3263527	0.090178	ZNF441	3.618983078
2386	0.3670852	0.1014332	MAK	3.618983077
2387	18.940223	5.2414578	SLC25A44	3.613541011
2388	81.874247	22.69352	TRIB3	3.607824869
2389	47.30791	13.114415	ATP6AP2	3.607321491
2390	19.980777	5.5395062	DNAJB2	3.606959889
2391	4.9495957	1.3736223	MVB12B	3.603316497
2392	216.70363	60.178315	JUNB	3.60102522
2393	22.475278	6.2451446	WDR13	3.598840327
2394	4.934882	1.371447	OSBPL7	3.598303186
2395	4.9050987	1.3646638	PER3	3.594364156
2396	39.062124	10.878441	PSMB10	3.590783221
2397	0.0359062	0.01	GRIA1	3.5906185
2398	336.65182	94.023128	CNN2	3.580521332
2399	86.412745	24.140123	MGAT1	3.579631567
2400	24.991358	6.9874502	ME1	3.576606239
2401	18.504818	5.1741368	CYTH1	3.576406819
2402	3.9301146	1.1002612	DHRS12	3.57198331
2403	0.0357093	0.01	FAM225B	3.5709304
2404	19.552202	5.477198	YY1AP1	3.569745225
2405	6.465632	1.8124806	HDHD1	3.567283331
2406	0.0356665	0.01	GNRHR	3.5666524

2407	30.074821	8.4338691	BCL3	3.565957696
2408	1.0130758	0.2844489	PLXNB3	3.561538917
2409	12.045991	3.387046	LMO4	3.556488876
2410	12.120355	3.4141363	PLEKHM1	3.550050078
2411	0.9759196	0.2752849	TTC28-AS1	3.545126299
2412	1.7617429	0.4969478	SLC27A6	3.545126295
2413	0.7921425	0.2234455	KAL1	3.545126289
2414	52.495971	14.812183	LGALS3	3.544107579
2415	67.178621	18.974047	TFE3	3.540553115
2416	23.950872	6.766844	BMP1	3.539445
2417	4.5724471	1.294278	RASA4	3.532816826
2418	77.883609	22.067283	ISG15	3.529370174
2419	156.1363	44.407318	DNAJB1	3.516003895
2420	1.3486073	0.3836084	ST8SIA5	3.515583575
2421	6.2630374	1.7815072	FNDC4	3.515583574
2422	14.498535	4.1279306	KREMEN1	3.512301049
2423	39.959481	11.392904	NCKAP5L	3.507400877
2424	23.624791	6.7415082	IQGAP3	3.504377713
2425	0.0350192	0.01	HEPHL1	3.501924
2426	29.982352	8.5704258	ZNF428	3.49835032
2427	3.8704688	1.1066904	COL5A3	3.4973366
2428	33.581537	9.6142661	HTATIP2	3.492886118
2429	15.709183	4.499127	RHBDF1	3.491606873
2430	15.773357	4.5199313	GLIPR1	3.489733692
2431	21.614341	6.2094939	MGAT5B	3.480853965
2432	34.290901	9.8566446	FN3KRP	3.478962911
2433	3.5160882	1.0109559	RHOU	3.477983747
2434	21.978314	6.3222148	SCRN2	3.476363066
2435	4.445758	1.2792176	SEMA3A	3.475372649
2436	1.3603724	0.3923859	ATRNL1	3.466924979
2437	35.911637	10.358354	ATP2B4	3.466924978
2438	41.287542	11.923333	COL12A1	3.462751702
2439	0.0346179	0.01	UTY	3.4617857
2440	61.902588	17.909047	CANT1	3.456498134
2441	44.653576	12.92754	SLC38A10	3.45414328
2442	0.2212544	0.064194	LOC100216546	3.446650574
2443	0.9693	0.2812296	UBE2Q2P1	3.446650564
2444	2.2460877	0.6516726	CDH23	3.446650563
2445	0.9128318	0.2648461	SOX5	3.446650563
2446	2.0062527	0.5820876	KIAA1614	3.446650562
2447	5.9330819	1.7214051	SVIP	3.446650562
2448	0.3747518	0.1087293	DSG3	3.446650559
2449	0.5717407	0.165883	LOC100093631	3.446650556

2450	24.013802	6.9835273	SMPD1	3.438635096
2451	25.507404	7.4264667	CAV2	3.434662212
2452	28.669324	8.3514301	FAH	3.432863959
2453	486.3415	142.00964	HSPB1	3.424707599
2454	0.0342312	0.01	ZNF626	3.4231193
2455	6.0025059	1.7536416	SLC17A5	3.422880559
2456	15.876396	4.6400718	GPRC5C	3.421584012
2457	2.1766283	0.6364154	RBMS3	3.420137866
2458	6.5891018	1.9265603	HDAC10	3.420137865
2459	3.445282	1.0073518	NOV	3.420137864
2460	0.7582652	0.221706	SOGA3	3.420137862
2461	32.135553	9.3999206	GALNS	3.418704746
2462	6.6767393	1.9539593	ZNF469	3.417030908
2463	10.211686	2.9948734	MAPK8IP3	3.409722161
2464	35.754816	10.497284	MTX1	3.40610173
2465	3.5067224	1.0295413	CROCC	3.40610173
2466	25.391636	7.457998	MPP1	3.404618238
2467	9.6053995	2.8233092	CCDC71L	3.402177651
2468	14.466934	4.2522572	TRIB2	3.402177651
2469	1.0991031	0.3235118	OXTR	3.3974127
2470	3.8717193	1.1396082	LOC100288778	3.397412696
2471	2.430623	0.7154335	CLDN23	3.397412695
2472	386.13955	113.75497	TNIP1	3.394485039
2473	16.903355	4.9796741	TRIOBP	3.394470194
2474	12.356297	3.6433089	B3GNTL1	3.391504153
2475	4.5860441	1.3539235	NOMO2	3.387225552
2476	7.3842343	2.1821125	FKBP1B	3.383984188
2477	2.9409573	0.8690813	TMEM25	3.383984186
2478	5.1354408	1.5220227	LOC100499489	3.374089497
2479	26.439181	7.8414443	SESN2	3.371723375
2480	29.044017	8.6169818	NAGK	3.370555679
2481	16.258117	4.8242836	CBR3	3.370058326
2482	100.0866	29.701788	CCL2	3.369716399
2483	11.946784	3.5477024	CRY2	3.367470753
2484	223.65743	66.494394	CTTN	3.363553232
2485	0.7494311	0.2230128	LOC100216545	3.360484302
2486	1.6795992	0.4998087	CAPS	3.360484299
2487	7.7185	2.2968416	LOC100127983	3.360484297
2488	1.6213007	0.4824604	FCAR	3.360484297
2489	42.199951	12.573819	F2R	3.356175985
2490	0.0335587	0.01	ZNF585B	3.3558696
2491	9.4229006	2.8081062	MBOAT2	3.355606961
2492	14.316701	4.268317	CCBL1	3.354179449

2493	13.810231	4.1173202	BRF2	3.354179448
2494	4.5634927	1.3660699	SLC35A1	3.340599776
2495	3.151883	0.9435081	PPP1R12B	3.340599775
2496	12.61336	3.7783474	PORCN	3.338327257
2497	17.38237	5.2077186	SRPX2	3.337808964
2498	140.16133	42.066033	CYB5R3	3.331936018
2499	1.3996261	0.4200858	PTAFR	3.331762212
2500	25.256775	7.5806056	CYB5R1	3.33176221
2501	2.2961951	0.6891834	OPN3	3.33176221
2502	5.206995	1.5628351	PITX2	3.331762209
2503	73.055175	21.93971	RPS27L	3.329814949
2504	57.145721	17.16802	ARAF	3.328614584
2505	2449.5832	736.37191	LGALS1	3.326557085
2506	18.054685	5.4299737	FCGRT	3.325004071
2507	209.84557	63.133545	ITGA3	3.323836368
2508	0.0332164	0.01	LCT	3.3216369
2509	46.736101	14.074976	RBCK1	3.320510257
2510	18.31839	5.5228414	SERPINB1	3.316841645
2511	29.386875	8.8638844	CRAT	3.315349588
2512	7.1020064	2.1464094	NEAT1	3.308784539
2513	4.5776936	1.383497	B3GALNT1	3.308784539
2514	2.0123066	0.6081709	DLEU2L	3.308784539
2515	14.206174	4.2934721	SLC35D2	3.308784538
2516	1.2507097	0.3779967	GPC2	3.308784534
2517	51.388036	15.538985	SYVN1	3.3070394
2518	52.694817	15.946282	GSTK1	3.30452064
2519	51.9541	15.73359	MAP3K11	3.302113601
2520	25.591425	7.7550134	TMUB2	3.29998458
2521	434.26671	131.62787	IL8	3.299200272
2522	12.538097	3.8027896	PDGFA	3.297078934
2523	136.96545	41.591088	CTSL1	3.293144275
2524	11.453415	3.480085	TLE4	3.291130963
2525	377.57461	114.72961	GANAB	3.290995375
2526	10.198997	3.1000135	FGD1	3.289984629
2527	140.84098	42.960489	NUCB1	3.278383956
2528	52.85366	16.123828	IER5	3.277984684
2529	2.7338945	0.8349508	DCLK2	3.274318035
2530	1.1156919	0.3407402	GIN1	3.274318032
2531	2.3182614	0.7080135	VMAC	3.274318032
2532	79.777934	24.406185	COMT	3.268758919
2533	13.931644	4.2645819	DMPK	3.266825315
2534	10.288235	3.154873	CASP6	3.261061685
2535	10.936637	3.3554835	PELI3	3.259332597

2536	1.8501973	0.5693442	PIK3IP1	3.249699101
2537	3.8568151	1.1868222	BDNF	3.249699099
2538	59.243368	18.238791	PCBP4	3.248207044
2539	15.965521	4.9191339	MAGEH1	3.245595945
2540	2.7729719	0.8546407	HECW1	3.244605528
2541	17.625513	5.4571824	AKAP17A	3.22978266
2542	29.410293	9.1107627	ZFAND2A	3.228082477
2543	0.03224	0.01	COL14A1	3.2240022
2544	10.549354	3.2725056	RNASEK	3.223631996
2545	30.317393	9.4094868	TSC22D4	3.222002802
2546	17.262633	5.3638815	C1QTNF1	3.218309962
2547	3.4878577	1.0842383	OAS1	3.216873859
2548	44.188126	13.745982	ADAR	3.214621146
2549	130.13128	40.481343	TMEM132A	3.214598841
2550	29.280938	9.1139207	SPG7	3.212770702
2551	75.060705	23.365281	ANAPC11	3.212488805
2552	13.679308	4.2594512	MEGF8	3.211518893
2553	5.6402868	1.7596285	PXMP4	3.205385023
2554	0.0319582	0.01	PRICKLE2-AS1	3.1958212
2555	6.94665	2.1763383	CXorf38	3.191898128
2556	1.8776083	0.5883442	SYNE1	3.191343115
2557	118.10509	37.026121	KRTCAP2	3.189777549
2558	2.8442573	0.8921336	LOC374443	3.188151771
2559	2.5310295	0.7938862	ADAM12	3.18815177
2560	5.0102544	1.5715232	EP400NL	3.188151769
2561	50.872391	15.960268	MGST3	3.187439652
2562	37.27648	11.700738	TSPAN13	3.185822951
2563	65.943556	20.730636	ZFPL1	3.180971247
2564	32.110319	10.101281	TXNIP	3.178836498
2565	6.240375	1.9649512	MEIS3P1	3.175842303
2566	21.838271	6.879178	CYHR1	3.17454657
2567	28.696579	9.0431473	WIPI1	3.173295517
2568	1.6997122	0.5360315	PLCB4	3.170918518
2569	3.195459	1.0077392	FBXL2	3.170918516
2570	7.0744754	2.2379996	CTBS	3.161070943
2571	15.621206	4.9455438	GPKOW	3.158642774
2572	15.512236	4.9114451	ACSF2	3.158385241
2573	84.100535	26.645901	PDLIM4	3.156227875
2574	1.0999714	0.3487892	ZC3H6	3.153685263
2575	10.789915	3.4286986	GSTA4	3.146941817
2576	32.85645	10.457263	PLXND1	3.141974269
2577	20.075181	6.3946756	TTC38	3.139358825
2578	18.642562	5.9479229	DNM1	3.134297854

2579	9.625843	3.0720919	CASP9	3.133318692
2580	17.452175	5.5752152	YPEL5	3.13031414
2581	275.53084	88.023808	CTNNA1	3.130185374
2582	5.740229	1.8348193	CLIP4	3.128498202
2583	45.190703	14.451395	PIGS	3.127082475
2584	33.872577	10.834996	HBEGF	3.126219768
2585	12.575832	4.0236412	WTIP	3.125485396
2586	9.34738	2.991675	FENDRR	3.124463661
2587	11.333868	3.6280156	C21orf2	3.123985404
2588	29.262928	9.3698715	EVA1B	3.123087448
2589	64.389214	20.623501	GGH	3.122128268
2590	105.73549	33.937865	SKP1	3.115560934
2591	0.3532794	0.1134203	HIF3A	3.114781188
2592	511.25601	164.3178	AES	3.111385462
2593	0.031109	0.01	LOC642366	3.1109046
2594	0.1177177	0.0379491	LUZP2	3.101985545
2595	0.09841	0.0317248	PLIN4	3.101985543
2596	0.0739617	0.0238434	TG	3.101985539
2597	0.1357651	0.0437672	PCDHGA9	3.101985538
2598	0.1618427	0.0521739	F13A1	3.101985536
2599	0.1680189	0.054165	C2orf91	3.101985531
2600	0.1319263	0.0425296	PROM2	3.101985522
2601	0.250681	0.0808131	SERINC4	3.10198552
2602	0.0855382	0.0275753	GOLGA6L5	3.10198552
2603	0.2384434	0.076868	CLEC7A	3.10198552
2604	0.1664533	0.0536602	KCNB1	3.101985519
2605	0.2065406	0.0665834	TGM4	3.101985518
2606	0.366363	0.118106	LOC286367	3.101985518
2607	0.2733706	0.0881276	GUCA1B	3.101985517
2608	0.2858704	0.0921572	TPTE2P5	3.101985517
2609	0.3178437	0.1024646	PATL2	3.101985516
2610	0.3664704	0.1181406	LOC727896	3.101985516
2611	0.2038802	0.0657257	TRPV3	3.101985516
2612	0.17817	0.0574374	ZNF677	3.101985513
2613	0.5041923	0.1625386	WNT9A	3.101985513
2614	0.3037894	0.0979338	NDP	3.101985512
2615	0.4725612	0.1523415	PILRA	3.101985511
2616	0.0520089	0.0167663	SLC1A2	3.101985511
2617	0.4913427	0.1583962	MDGA1	3.10198551
2618	0.225378	0.072656	KCP	3.10198551
2619	0.4743539	0.1529195	LDHC	3.10198551
2620	1.0071663	0.3246844	BTN2A3P	3.10198551
2621	0.8220888	0.2650202	IFNLR1	3.101985509

2622	0.4752554	0.1532101	DEPTOR	3.101985509
2623	1.1840881	0.3817194	ADM5	3.101985509
2624	0.4930588	0.1589494	FAM86B2	3.101985509
2625	0.3705978	0.1194712	LOC154092	3.101985508
2626	0.7800356	0.2514633	ACOT1	3.101985508
2627	0.4805523	0.1549176	AKR7A3	3.101985508
2628	1.6551319	0.5335718	TP53INP1	3.101985508
2629	0.5410632	0.1744248	MLLT4-AS1	3.101985508
2630	1.6973715	0.5471887	ADC	3.101985508
2631	0.4736352	0.1526878	CD27	3.101985508
2632	0.1492833	0.0481251	ZNF813	3.101985507
2633	0.7051111	0.2273096	TUBBP5	3.101985507
2634	0.26925	0.0867992	BDNF-AS	3.101985507
2635	0.33649	0.1084757	INHBA-AS1	3.101985506
2636	2.8073574	0.9050195	RRAGB	3.101985506
2637	0.2306155	0.0743445	PCDHB8	3.101985506
2638	0.2485879	0.0801383	BHMT	3.101985506
2639	4.1959631	1.3526701	RNU12	3.101985506
2640	2.3832217	0.7682891	INCA1	3.101985506
2641	5.10734	1.6464745	ANO8	3.101985506
2642	0.2394479	0.0771918	LOC100506655	3.101985506
2643	9.035904	2.912942	ZSCAN25	3.101985506
2644	0.9530465	0.3072376	NAPA-AS1	3.101985505
2645	6.1293971	1.9759593	RNY3	3.101985505
2646	6.8675181	2.2139104	ADCK4	3.101985505
2647	2.7123579	0.8743941	RAMP1	3.101985505
2648	13.04438	4.2051712	C19orf71	3.101985505
2649	3.9569525	1.2756193	RNU105A	3.101985505
2650	0.65329	0.2106038	HLA-F-AS1	3.101985505
2651	2.4711403	0.7966318	PMS2P4	3.101985505
2652	4.4577433	1.4370613	SMIM14	3.101985505
2653	3.8434334	1.2390237	KIAA0895L	3.101985504
2654	0.2497797	0.0805225	IL21R	3.101985504
2655	0.2497797	0.0805225	LOC283299	3.101985504
2656	4.3720175	1.4094255	TREX1	3.101985504
2657	35.918047	11.57905	TMEM134	3.101985504
2658	1.8802962	0.6061589	TSSK3	3.101985504
2659	0.2760258	0.0889836	LOC729987	3.101985504
2660	0.4494597	0.1448942	LOC100130015	3.101985503
2661	0.8098426	0.2610723	LOC728875	3.101985503
2662	0.758736	0.2445969	NDUFB2-AS1	3.101985503
2663	0.758736	0.2445969	CHKB	3.101985503
2664	0.5912043	0.190589	LINC00324	3.101985503

2665	0.8369458	0.2698097	ZP3	3.101985502
2666	0.4573508	0.1474381	DCDC2B	3.101985502
2667	0.3594128	0.1158654	CCR6	3.101985501
2668	1.3924243	0.4488816	HIST1H2BN	3.101985501
2669	0.9915916	0.3196635	AURKC	3.101985501
2670	0.9915916	0.3196635	LOC100652768	3.101985501
2671	0.1578784	0.0508959	MLC1	3.101985501
2672	0.2366383	0.0762861	MST1P2	3.101985501
2673	0.5670735	0.1828098	ZNF660	3.101985501
2674	0.214771	0.0692366	LOC100630923	3.101985501
2675	0.2608254	0.0840834	FOXP3	3.101985501
2676	0.6788257	0.2188359	HIST1H3D	3.101985501
2677	0.8570233	0.2762822	CPT1C	3.101985501
2678	0.4590297	0.1479793	MIRLET7DHG	3.1019855
2679	0.3316703	0.1069219	REM2	3.1019855
2680	0.363699	0.1172471	EXOC3L2	3.1019855
2681	0.2455611	0.0791625	NOS3	3.1019855
2682	0.2553915	0.0823316	GP2	3.101985498
2683	0.2412962	0.0777877	LINC-ROR	3.101985497
2684	0.1839901	0.0593137	TMEM213	3.101985497
2685	0.3939499	0.1269993	PVRIG	3.101985496
2686	0.5501087	0.1773408	LOC284276	3.101985496
2687	0.3504476	0.1129753	GLIPR1L2	3.101985496
2688	0.4614011	0.1487438	PAX8	3.101985495
2689	0.4276324	0.1378576	PRSS27	3.101985494
2690	0.4540294	0.1463674	AARSD1	3.101985494
2691	0.4835255	0.1558761	SCG2	3.101985494
2692	0.3927126	0.1266004	CIDEA	3.101985494
2693	0.4750748	0.1531519	FMO5	3.101985493
2694	0.2546633	0.0820969	HNF1A-AS1	3.101985492
2695	0.2200241	0.0709301	NBPF24	3.101985492
2696	0.1544844	0.0498018	PRND	3.101985489
2697	0.0804528	0.0259359	GRIN3A	3.101985488
2698	0.1208113	0.0389464	LRRC37A2	3.101985486
2699	0.2484891	0.0801065	LOC654841	3.101985485
2700	0.2411101	0.0777277	LTF	3.101985483
2701	92.841637	29.961086	TGOLN2	3.098740751
2702	0.0309566	0.01	PI15	3.0956551
2703	39.874759	12.89802	EFEMP1	3.091541111
2704	6187.1304	2002.0977	TMSB10	3.090323905
2705	89.145023	28.865212	UQCRQ	3.088320371
2706	4.6910853	1.5207333	ARMCX4	3.084752253
2707	30.985964	10.049983	PGS1	3.083185594

2708	39.052534	12.670304	TNKS1BP1	3.082209642
2709	260.00632	84.455118	BASP1	3.078633043
2710	25.12048	8.1629793	SURF1	3.077366573
2711	124.51373	40.466086	CALU	3.076989651
2712	12.668986	4.1184745	ARHGEF17	3.076135627
2713	3.5874145	1.1682907	PIK3R3	3.070652319
2714	19.343422	6.312805	ASAH1	3.064156413
2715	7.8970283	2.5788602	TRNAU1AP	3.062216461
2716	44.818776	14.649787	LIPA	3.059346528
2717	26.211452	8.5689076	ACTR1B	3.058902373
2718	3.3797087	1.1048763	ATP9B	3.058902373
2719	168.17012	55.017411	FLOT1	3.056670976
2720	27.360091	8.9518312	TMEM127	3.056368071
2721	3.585119	1.1743909	L3MBTL4	3.052747641
2722	3.9260921	1.2860847	CHFR	3.05274764
2723	14.05419	4.6091615	TNS1	3.049185753
2724	17.092567	5.6078512	ERBB2	3.047970833
2725	386.22139	126.7617	PEA15	3.046830235
2726	14.759939	4.8455298	RGS3	3.046093875
2727	18.912503	6.2135897	FBXW4	3.043732257
2728	67.912936	22.322658	RHBDD2	3.042331938
2729	3.5315963	1.1612653	TLCD2	3.041162262
2730	16.779348	5.5174129	AAED1	3.04116226
2731	6.1679953	2.0307088	YPEL3	3.037360808
2732	90.482924	29.789982	PPP1R7	3.037360807
2733	7.2339561	2.3816585	LCAT	3.037360807
2734	44.47212	14.664528	UXS1	3.032632172
2735	115.52206	38.130674	TGFB1I1	3.029635989
2736	33.188278	10.957296	SIPA1	3.028874736
2737	35.236091	11.635381	ARHGEF10	3.028357367
2738	42.529625	14.045877	ZNF395	3.027908239
2739	33.985149	11.231555	BSDC1	3.025863163
2740	8.1061352	2.6793658	RNFT1	3.025393271
2741	90.603176	29.955034	TMEM59	3.02463941
2742	11.108839	3.6747067	CLN8	3.023054577
2743	1.7245603	0.5705841	RAB43	3.022447416
2744	5.7396118	1.9022774	MID1	3.017231804
2745	27.728469	9.1919311	TBC1D17	3.016609757
2746	51.493485	17.07446	CALD1	3.015819242
2747	8.4518917	2.8025193	CRYZL1	3.015819241
2748	2.8998477	0.9627415	MITF	3.012072881
2749	90.456966	30.047053	COL6A1	3.010510419
2750	3.2231919	1.0715449	SNAP25	3.007985944

2751	42.701231	14.202349	FAT1	3.006631484
2752	0.0300461	0.01	LY75	3.0046064
2753	29.60451	9.8711299	PPP6R2	2.999100414
2754	50.341904	16.795654	SLC35F6	2.997317283
2755	8.690963	2.9003949	NEXN	2.996475794
2756	1.7674591	0.5905026	IGDCC4	2.993143909
2757	71.418996	23.888163	POMGNT1	2.989723174
2758	6.885856	2.3038154	BVES	2.988892284
2759	1.3590135	0.4549613	MICU3	2.987097155
2760	30.554814	10.228932	DPM3	2.987097155
2761	1.4561642	0.4874847	IFITM10	2.987097152
2762	34.521677	11.560866	COL18A1	2.986080442
2763	45.173302	15.145214	GPR108	2.98267837
2764	16.20885	5.4385927	FAM174A	2.980339014
2765	39.922902	13.405056	ARL6IP5	2.978197351
2766	6.8048816	2.285123	ORAI3	2.977906085
2767	22.095929	7.4230792	SNX7	2.976652758
2768	5.8317745	1.9617532	ARHGAP26	2.97273611
2769	83.225781	27.996357	FDPS	2.97273611
2770	0.6966844	0.234358	MAGI2	2.972736109
2771	3.1338271	1.0541895	AHSA2	2.972736109
2772	32.425206	10.943035	RAB32	2.963090632
2773	18.218843	6.1503261	TOM1	2.962256428
2774	19.119925	6.460819	CASP7	2.959365483
2775	16.42322	5.5514327	PMVK	2.958375065
2776	63.04169	21.326953	MAFF	2.955963287
2777	29.919843	10.124064	RND3	2.955319525
2778	2.4648078	0.8343199	AGER	2.954271909
2779	44.30924	15.001726	GTPBP2	2.953609516
2780	51.124371	17.32834	LMF2	2.95033288
2781	243.92905	82.753076	PPP1R15A	2.947673536
2782	5.5150769	1.8724884	VWA5A	2.945319571
2783	24.469169	8.3224425	RNASEH2C	2.940142783
2784	115.54414	39.300138	COL4A1	2.940044211
2785	38.626973	13.142468	FAM213A	2.939095854
2786	1.9595286	0.6667959	HCFC2	2.938723111
2787	4.7761022	1.6264352	SLC37A1	2.936546279
2788	74.146867	25.25304	NDUFA2	2.936156092
2789	1.9759892	0.6732015	DSEL	2.935212092
2790	0.029352	0.01	RP1	2.9352042
2791	26.613789	9.0819475	PIP5K1C	2.930405521
2792	1.087413	0.3711747	KLRD1	2.92965298
2793	5.1181617	1.7470198	ADPRH	2.929652978

2794	2.3697602	0.8088877	LYRM9	2.929652977
2795	1.156641	0.3948048	SCNN1D	2.929652974
2796	21.156203	7.2245042	TNFAIP1	2.928395076
2797	26.851006	9.1790962	SIN3B	2.925234195
2798	184.04992	62.922255	LASP1	2.925036927
2799	30.956348	10.586017	C17orf62	2.924267586
2800	3.2520078	1.113886	GLRB	2.919515771
2801	44.163198	15.146791	FAM89B	2.91568007
2802	6.4636163	2.2181354	KLHDC8B	2.913986385
2803	43.131276	14.829607	MAFG	2.908456998
2804	68.708684	23.636066	USP11	2.906942489
2805	12.188031	4.1944486	ADCY6	2.905752845
2806	34.041353	11.7235	GNPTG	2.903685063
2807	25.896763	8.9278349	HSPA1B	2.900676711
2808	1.1941443	0.4124585	FAXC	2.895186474
2809	1.7012204	0.5876031	GEMIN8P4	2.895186472
2810	2.0360035	0.7032374	TMEM44-AS1	2.895186472
2811	6.9703587	2.4075681	TCTN2	2.895186472
2812	4.4866325	1.5496869	KSR1	2.895186472
2813	4.3886778	1.5158532	PPAP2B	2.895186472
2814	1.0360771	0.3578619	CD34	2.895186469
2815	6.0306782	2.0848867	GON4L	2.892568762
2816	10.391278	3.5933302	NACC2	2.891823884
2817	37.442688	12.949644	ZBTB4	2.891406855
2818	6.8606299	2.3740157	MANBA	2.889883932
2819	19.115665	6.6204127	PYGO2	2.887382735
2820	9.3053263	3.2243806	SLC1A3	2.885926813
2821	38.353206	13.294713	CRYBB2P1	2.884846521
2822	26.487227	9.1832352	DECR1	2.884302312
2823	12.951808	4.4918255	AP5Z1	2.883417422
2824	30.831914	10.696469	PIM1	2.882438585
2825	4.4470328	1.5430443	CDK19	2.881986534
2826	23.534771	8.1661628	IGSF8	2.881986533
2827	14.008665	4.8613755	FBXO25	2.881625879
2828	20.809645	7.2295398	NECAB3	2.878418982
2829	116.8974	40.615379	ATP6AP1	2.878155964
2830	91.569477	31.823344	FAM127A	2.877430999
2831	12.52942	4.354721	VRK3	2.877203948
2832	6.252962	2.1732773	FUCA1	2.877203947
2833	81.84152	28.462096	C11orf84	2.875456529
2834	0.0286854	0.01	FAM186A	2.868541
2835	3.5517949	1.2382052	TMEM242	2.868502725
2836	4.3372124	1.5128122	EBF4	2.866986603

2837	10.324184	3.602745	OSGIN1	2.865643751
2838	30.534725	10.663907	RCAN1	2.863371235
2839	22.866587	7.9879514	SCP2	2.862634772
2840	12.333686	4.3095378	SLC35A2	2.861950913
2841	121.23453	42.36356	CKS1B	2.861764407
2842	2.8489751	0.9966008	RCAN2	2.858692524
2843	4.4569215	1.5606581	LOC100133091	2.855796179
2844	2.4171399	0.846982	ATP8B1	2.853826665
2845	135.31543	47.430838	CTSZ	2.852899834
2846	93.558522	32.810115	TMEM14B	2.851514626
2847	32.224901	11.301748	TEX30	2.85132001
2848	0.0284466	0.01	ABCA4	2.844656
2849	0.9660322	0.3397351	MAFA	2.843486716
2850	1.9394201	0.682057	CD27-AS1	2.843486714
2851	1.0973645	0.3859222	IL7	2.843486714
2852	4.0645293	1.4294174	HIST1H2AE	2.843486713
2853	2.160598	0.7598411	BRICD5	2.843486712
2854	1.3351162	0.4695349	ZNF575	2.843486712
2855	0.9713536	0.3416065	CBLN3	2.843486711
2856	1.2154796	0.427461	PLEKHA8P1	2.843486709
2857	0.5021675	0.1766027	ZNF347	2.843486709
2858	55.121423	19.391295	PPP1R12C	2.842586021
2859	21.577946	7.6083135	CLIP2	2.836101034
2860	20.491973	7.2292982	HMGCL	2.834572963
2861	25.234378	8.9081921	TCTA	2.83271593
2862	13.329298	4.7091848	DOCK9	2.830489568
2863	16.123153	5.6974656	PCYOX1	2.829881514
2864	2.709871	0.9575917	SLC38A4	2.829881513
2865	6.0506834	2.1381402	SLC31A2	2.829881513
2866	30.929301	10.933507	PXMP2	2.828854706
2867	22.885997	8.0967222	HDAC5	2.826575578
2868	4.6809125	1.6577805	TMPO-AS1	2.823602192
2869	92.393142	32.725736	WSB2	2.823256373
2870	4.16799	1.4780175	MIR155HG	2.819986822
2871	24.530827	8.7061705	NTPCR	2.817636833
2872	9.5200343	3.3807098	HSPA2	2.815986841
2873	6.1128792	2.1725722	SLC7A11	2.81365993
2874	2.1589223	0.7676259	SORBS2	2.812466858
2875	24.974529	8.885877	ANKRD10	2.810586867
2876	1.2990782	0.4628724	ZNF132	2.806558317
2877	0.9127687	0.325227	ANGPT1	2.806558315
2878	4.999483	1.7813572	U2AF1L4	2.806558315
2879	90.586037	32.322534	GNA11	2.802566055

2880	71.729253	25.603798	PRDX4	2.801508277
2881	78.934369	28.189949	HPCAL1	2.800089105
2882	2.4361822	0.8707277	PTPRH	2.79786928
2883	2.967	1.0604498	TXNDC5	2.79786928
2884	30.128613	10.768414	CD97	2.797869279
2885	9.3452691	3.3440674	DYNLT3	2.794581537
2886	42.671305	15.271772	DDA1	2.794129338
2887	126.89081	45.430078	ARPC5	2.793101355
2888	3.2079741	1.1490755	LMCD1	2.791786956
2889	2.495249	0.893782	NIPAL2	2.791786955
2890	14.872563	5.3272559	KDM4B	2.791786955
2891	6.7386665	2.4137467	PHLDB3	2.791786954
2892	2.0446172	0.7323686	GALNT12	2.791786953
2893	79.166395	28.388752	CDK16	2.788653636
2894	17.597155	6.3169495	PDK2	2.785704631
2895	24.172877	8.6782465	GINM1	2.785456373
2896	15.50262	5.5688034	RBP4	2.783833147
2897	87.310391	31.387055	ASPH	2.7817325
2898	1.4885679	0.5357465	IL12RB1	2.77849273
2899	17.21243	6.200832	POFUT2	2.775825956
2900	7.2007957	2.5944506	NMRK1	2.775460715
2901	6.3425935	2.2881036	SFXN5	2.771987047
2902	17.924517	6.4745941	ITGB1BP1	2.768438677
2903	100.51618	36.307893	SERPINB6	2.768438677
2904	35.215924	12.734448	SYNPO	2.765406434
2905	155.24862	56.146855	CNN3	2.7650457
2906	7.4105633	2.6803125	HCN2	2.764813168
2907	11.173992	4.0415	P4HTM	2.764813167
2908	56.053253	20.27768	PVR	2.76428338
2909	0.0920086	0.0332972	PAX7	2.763255048
2910	10.141728	3.6718225	JDP2	2.762041888
2911	0.2388875	0.0866376	TMEM51-AS1	2.757320465
2912	0.7082396	0.2568579	REC8	2.757320451
2913	14.410604	5.2263072	DNAL4	2.75732045
2914	2.4809464	0.8997672	STARD5	2.757320449
2915	4.0076827	1.4534701	C3orf14	2.757320449
2916	2.69337	0.976807	ACYP2	2.757320449
2917	0.9246789	0.3353542	LPAR2	2.757320448
2918	2.3851159	0.8650122	RPS10P7	2.757320448
2919	1.6781037	0.6085994	POLR2J2	2.757320448
2920	0.6040565	0.2190737	ZNF816	2.757320447
2921	0.660799	0.2396526	ACAP1	2.757320443
2922	0.5101579	0.1850194	PTCHD4	2.757320441

2923	99.785927	36.202965	NAPA	2.756291598
2924	288.58528	104.91393	CSRP1	2.750685895
2925	11.301266	4.111175	DCBLD1	2.748913985
2926	17.145746	6.2483037	CASKIN2	2.744064101
2927	30.183043	11.00152	LPPR2	2.743533847
2928	148.85679	54.262882	EIF3K	2.743252487
2929	17.396986	6.3424148	STAT2	2.742959406
2930	42.979582	15.680783	MAN2B1	2.740907827
2931	381.40389	139.26338	UBE2S	2.738723414
2932	11.149433	4.072253	PEX6	2.737902699
2933	4.7628123	1.7401287	ADCK5	2.737046033
2934	1.1180231	0.408478	DENND1B	2.737046032
2935	177.68132	64.934525	UBA1	2.736315111
2936	16.074423	5.8777922	CCNDBP1	2.734772268
2937	50.325576	18.407621	VIMP	2.733953326
2938	15.576782	5.7073147	RAB23	2.729266317
2939	118.00283	43.276075	GUK1	2.726745324
2940	93.455494	34.309232	SCYL1	2.723916788
2941	62.829955	23.079989	LRRC8A	2.722269765
2942	8.5425915	3.1381775	DNASE1L1	2.722150546
2943	103.63447	38.268579	NRBP1	2.708082586
2944	35.403776	13.073374	PMM1	2.708082584
2945	0.0270719	0.01	BZRAP1	2.7071902
2946	54.605168	20.174564	TMEM219	2.706634412
2947	0.2756607	0.1018948	MEF2BNB-MEF2B	2.705347152
2948	114.73574	42.452819	NPLOC4	2.702664827
2949	22.467251	8.325906	LENG8	2.698475196
2950	153.94335	57.054583	MDK	2.698176742
2951	29.227726	10.836455	CUEDC1	2.697166641
2952	14.398511	5.3397092	WDR45	2.696497204
2953	54.097805	20.065604	TPGS2	2.696046662
2954	22.008177	8.1668559	TMEM104	2.694816423
2955	18.818684	6.9935081	MPC2	2.690878992
2956	49.839343	18.530742	CITED2	2.68954923
2957	3.7775546	1.4051377	AGPAT4	2.688387438
2958	0.7416352	0.2758662	LOC646471	2.688387438
2959	1.6213007	0.6030755	CROT	2.688387437
2960	33.746728	12.564215	VPS37C	2.685940113
2961	28.511097	10.633813	STX4	2.681173519
2962	5.9658933	2.2292215	GNB1L	2.67622279
2963	3.7078763	1.3854886	CCDC126	2.676222789
2964	7.8372462	2.928473	FUNDC1	2.676222789
2965	369.83336	138.38099	CD63	2.672573395

2966	5.8730768	2.1987038	FRS3	2.671154185
2967	6.1970115	2.3199752	ABHD3	2.671154185
2968	47.609192	17.833372	ATF6B	2.66966856
2969	63.999847	23.984581	FAM64A	2.668374628
2970	18.060823	6.7701662	NTN1	2.667707535
2971	11.179961	4.1913205	GNAI1	2.667407826
2972	4.8301702	1.8109261	TNRC6C	2.667237537
2973	6.7225645	2.5210066	NUDT18	2.666619119
2974	5.0063702	1.8788971	ELMOD3	2.664526011
2975	34.79212	13.066449	RRAS2	2.662706513
2976	38.9342	14.643277	LTBP3	2.658844719
2977	1.4980795	0.5634325	DNAJC27	2.658844719
2978	3.7177314	1.3982507	NUDT10	2.658844719
2979	3.1351367	1.1791349	LHX8	2.658844718
2980	130.55202	49.176587	HN1L	2.65475972
2981	2.8876399	1.0886802	RNASEL	2.65242239
2982	25.415556	9.5826769	UBE2H	2.65223964
2983	95.321138	35.959553	STK24	2.650787614
2984	0.0264887	0.01	LRP2	2.6488656
2985	7.2723926	2.7473807	TAPBPL	2.647027632
2986	2.4526605	0.9265716	MAOA	2.64702763
2987	10.410291	3.9335546	IL17RA	2.646535253
2988	47.869543	18.087627	STX5	2.646535253
2989	1.4960014	0.5661456	RCBTB2	2.642432098
2990	3.6039011	1.3638576	HEXIM2	2.642432098
2991	18.271532	6.9173988	L3MBTL2	2.641387658
2992	7.4861956	2.8373241	C10orf10	2.63847043
2993	10.906339	4.1335839	SDSL	2.638470429
2994	6.8792068	2.6090336	TIFA	2.63668768
2995	4.338112	1.6452885	C10orf32	2.63668768
2996	4.671813	1.7718492	LINC00673	2.636687679
2997	17.084241	6.4861265	ZNF672	2.63396664
2998	3.1816718	1.2088475	LOC284385	2.631987701
2999	2.0582667	0.7820199	INADL	2.631987699
3000	14.364817	5.4615702	ACAP3	2.630162467
3001	4.9064329	1.8692904	APH1B	2.624756966
3002	33.298733	12.694289	PAPSS2	2.623127088
3003	86.629287	33.048694	TNC	2.621262026
3004	2.0905969	0.7981039	ZNF175	2.619454429
3005	28.046037	10.706824	TULP3	2.619454427
3006	5.0408536	1.9243906	RARB	2.619454426
3007	2.1750017	0.8303262	HHAT	2.619454426
3008	33.037807	12.636229	ANKRD13D	2.61453064

3009	0.0261447	0.01	USP6	2.6144712
3010	8.2500311	3.1555989	RAB11FIP1	2.614410548
3011	25.632024	9.8079434	DDB2	2.613394382
3012	161.87808	62.033099	CLPTM1	2.609543648
3013	1.8954322	0.726925	STS	2.607466078
3014	5.0237619	1.9266835	CDK20	2.607466077
3015	19.270652	7.3919226	SLC2A6	2.606987818
3016	2.7426715	1.0525791	C17orf103	2.605667823
3017	139.01331	53.357754	RELA	2.605306603
3018	40.106349	15.403749	8-Sep	2.60367458
3019	10.865749	4.1764594	TRADD	2.601665262
3020	7.4722576	2.8735647	PGPEP1	2.600344285
3021	84.461471	32.481463	BIRC5	2.600297644
3022	5.5371848	2.1298837	EXTL2	2.59975928
3023	50.568812	19.460959	EBP	2.598474815
3024	33.742103	13.003087	NUCB2	2.594930183
3025	64.440919	24.834834	ESYT1	2.594779542
3026	233.34217	89.952563	ATP6V0E1	2.594058055
3027	26.930003	10.3838	ZDHHC12	2.593463291
3028	13.558899	5.230087	MIR22HG	2.59248064
3029	27.536659	10.627523	RAB24	2.591070245
3030	0.1463685	0.0566225	KIAA1875	2.58498794
3031	0.1854418	0.071738	LOC100506023	2.584987935
3032	0.3195331	0.1236111	SCIN	2.584987932
3033	0.3295375	0.1274812	LOC100335030	2.58498793
3034	0.2936031	0.1135801	TRPC4	2.584987929
3035	0.24964	0.096573	SLC9A4	2.584987928
3036	0.3320578	0.1284562	SEMA3D	2.584987928
3037	0.9933246	0.3842666	IL23A	2.584987925
3038	0.256019	0.0990407	MSH5-SAPCD1	2.584987925
3039	0.7814005	0.302284	SPDYE5	2.584987925
3040	0.1636817	0.0633201	COL22A1	2.584987924
3041	0.2945992	0.1139654	SPIB	2.584987924
3042	0.2387165	0.0923472	LOC100506085	2.584987924
3043	0.4357999	0.1685887	CHP2	2.584987923
3044	0.4092685	0.1583251	TMEM151A	2.584987923
3045	0.6239506	0.2413747	LOC283693	2.584987923
3046	2.3589853	0.9125711	TMEM220	2.584987922
3047	0.7816935	0.3023974	LINC00629	2.584987922
3048	2.216492	0.8574477	EFCAB2	2.584987922
3049	8.1918043	3.1689913	SPSB2	2.584987922
3050	28.844221	11.158358	NUDT14	2.584987921
3051	6.0682051	2.3474791	CCDC84	2.584987921

3052	5.4650219	2.1141383	C6orf226	2.584987921
3053	3.9732984	1.5370665	B9D2	2.584987921
3054	1.2660966	0.4897882	GSDMB	2.584987921
3055	0.2212309	0.085583	KIAA1751	2.584987921
3056	17.081926	6.6081261	ST3GAL3	2.584987921
3057	0.5186648	0.2006449	FAM227B	2.58498792
3058	0.6250735	0.2418091	POPDC2	2.584987919
3059	1.5763956	0.6098271	MYL5	2.584987919
3060	0.5489976	0.2123792	CCT6B	2.584987919
3061	0.6786047	0.2625175	LOC100131094	2.584987919
3062	0.7093244	0.2744014	GUSBP3	2.584987918
3063	1.0820327	0.4185833	TCAP	2.584987918
3064	0.2971193	0.1149403	MEFV	2.584987915
3065	0.3237022	0.1252239	LIMS3-LOC440895	2.584987914
3066	0.3250148	0.1257317	SPDYE3	2.584987911
3067	17.257075	6.6805055	PARVA	2.583199002
3068	126.57393	49.069124	PKN1	2.579502537
3069	15.806822	6.1291071	PLEKHA2	2.578976321
3070	38.01455	14.75487	PCNXL3	2.576407049
3071	46.403483	18.011515	ZNF358	2.576323157
3072	136.18512	52.9382	ITM2B	2.572530148
3073	5.9635316	2.3195242	SNCA	2.571015014
3074	17.868363	6.9499258	ARMC7	2.571015013
3075	108.34348	42.160187	KLC2	2.569805612
3076	140.50026	54.700112	PEBP1	2.568555234
3077	2.8684313	1.1173557	ZIC1	2.567160418
3078	25.058417	9.7635635	PHF1	2.566523722
3079	26.147503	10.190621	CCS	2.565839861
3080	9.1007229	3.5478971	C1QTNF6	2.565103399
3081	416.399	162.47619	COX8A	2.562830881
3082	5.4414895	2.1235	ALDH6A1	2.562509765
3083	4.7936931	1.8707024	RAD51D	2.562509765
3084	93.542957	36.517823	HMG20B	2.561569912
3085	5.5888468	2.1828287	DHRS13	2.560368988
3086	57.89474	22.625909	UBXN6	2.55878067
3087	43.576578	17.054632	FLOT2	2.55511695
3088	29.515622	11.570105	SLC12A4	2.551024577
3089	1.8954723	0.7431705	TMEM229B	2.550521415
3090	1.7982233	0.7050414	LACC1	2.550521415
3091	16.385219	6.4376462	WSB1	2.545218877
3092	335.55225	131.86185	CTSB	2.544725808
3093	50.623933	19.898363	NRP1	2.544125569
3094	51.854814	20.382308	SPRY2	2.544109042

3095	14.44353	5.6795661	FAHD2A	2.543069199
3096	175.61844	69.108636	TFDP1	2.541193846
3097	58.22478	22.925994	KIFC3	2.53968401
3098	6.8224147	2.6881192	CLCN2	2.537988141
3099	3.1575682	1.2441225	IL11RA	2.537988141
3100	32.321219	12.752256	TUSC3	2.534549133
3101	23.565332	9.3144039	JOSD2	2.529988178
3102	9.3653546	3.7017385	TESK1	2.529988178
3103	27.754421	10.970178	ACOT9	2.529988178
3104	4.4729649	1.7696884	ST20	2.527543745
3105	2.8136171	1.1131823	GEMIN8	2.527543745
3106	6.1872996	2.4479496	HIST2H2BE	2.527543745
3107	0.8544146	0.3380415	CLEC2D	2.527543743
3108	16.668061	6.5988537	IFNGR1	2.525902483
3109	3.7851492	1.499145	TLR4	2.524871922
3110	25.408973	10.06629	MMD	2.524164676
3111	137.47247	54.558287	COX7A2	2.519735798
3112	63.489928	25.23392	LEPRE1	2.51605491
3113	12.406873	4.9387048	DTX4	2.512171361
3114	0.6975372	0.2777781	HNRNPU-AS1	2.511131126
3115	153.62212	61.176461	SMS	2.511131124
3116	125.12857	49.849787	PLIN3	2.510112408
3117	2.7953339	1.1145722	ATP7A	2.507988282
3118	280.19986	111.794	CKAP4	2.506394465
3119	62.358741	24.884148	GNA12	2.505962507
3120	24.018623	9.5851804	UNC93B1	2.505808111
3121	16.9179	6.7588888	FNBP1	2.503059342
3122	34.274058	13.695874	GAA	2.502509707
3123	4.5179831	1.8060365	C20orf194	2.501601214
3124	19.544618	7.8157994	FBXO31	2.500654982
3125	5.3864398	2.1555919	DSTNP2	2.498821657
3126	0.024982	0.01	SCN1A	2.4981959
3127	100.93888	40.424248	ATP6V1E1	2.496988333
3128	216.02604	86.536927	SLC3A2	2.496345124
3129	23.739569	9.5116152	LMAN2L	2.495850406
3130	39.564909	15.869909	LOC401397	2.49307724
3131	78.205356	31.370987	IFI27L2	2.492919858
3132	250.73818	100.61853	UBC	2.491968277
3133	14.574085	5.8489147	DTNBP1	2.491758848
3134	29.011298	11.650165	ALDH2	2.490205029
3135	31.940627	12.828841	NFU1	2.489751524
3136	220.16794	88.443938	EMP3	2.489350298
3137	37.145685	14.926347	SCARB2	2.488598541

3138	173.759	69.82866	PSMA1	2.488362168
3139	7.3642779	2.9598582	XKR8	2.488050874
3140	20.880627	8.3923632	SLC1A4	2.488050874
3141	28.729672	11.565377	LZTS2	2.484110344
3142	0.213033	0.0858454	SPTBN5	2.481588409
3143	0.4021862	0.1620681	C2orf16	2.481588409
3144	1.0182386	0.4103173	RASAL2-AS1	2.481588406
3145	0.8979512	0.3618453	MAB21L2	2.481588405
3146	2.4197329	0.9750742	PPM1H	2.481588405
3147	1.6750127	0.674976	SPIN3	2.481588405
3148	231.22562	93.176459	CAPZB	2.481588405
3149	4.5468982	1.8322532	TARSL2	2.481588404
3150	0.2049159	0.0825745	TSHZ2	2.481588403
3151	0.6771714	0.2728782	ACTC1	2.481588402
3152	28.220766	11.381265	KIAA0195	2.479580647
3153	7.2665871	2.9313317	MROH1	2.478937135
3154	37.114959	14.972126	ARL2BP	2.478937134
3155	19.194691	7.7436804	GLIPR2	2.478755542
3156	12.828506	5.1763206	NADSYN1	2.47830588
3157	6.9820019	2.8206442	LMBR1L	2.475321767
3158	0.0247329	0.01	ADARB2	2.473291
3159	25.433892	10.298686	PSMD9	2.469624823
3160	10.891693	4.4137272	BAI2	2.467685947
3161	2.6585944	1.0782395	SLC27A3	2.465680786
3162	24.24971	9.854429	MICAL1	2.46079297
3163	4.2732736	1.7369651	ABHD15	2.460195401
3164	102.79165	41.797085	AP2B1	2.459301793
3165	0.0245899	0.01	COL6A6	2.4589912
3166	17.074079	6.9446042	TMEM216	2.458610734
3167	28.879952	11.748947	FRMD8	2.458088513
3168	5.6204265	2.288691	TPD52L1	2.455738525
3169	1.4616429	0.5951948	CSAD	2.455738523
3170	0.585565	0.2384476	NOTCH4	2.455738522
3171	18.286329	7.4581189	OGFOD3	2.451868782
3172	23.287515	9.4990243	UBE2B	2.45156919
3173	7.3181526	2.986856	MFSD11	2.450118986
3174	20.400405	8.3303139	PCNT	2.448935926
3175	14.461029	5.9050256	ARL3	2.448935925
3176	11.575486	4.7302448	RGL1	2.447121898
3177	67.335415	27.524595	RBBP7	2.446372627
3178	23.617757	9.6564693	AIFM2	2.445796263
3179	3.6401659	1.4894366	FLJ33630	2.443988581
3180	1.5472264	0.6330743	NUDT12	2.443988581

3181	7.4428393	3.0453658	SUZ12P1	2.44398858
3182	35.996921	14.734032	OTUD5	2.443114166
3183	47.413643	19.408311	JUND	2.442955618
3184	13.8933	5.6893386	UNC119	2.441988589
3185	98.850378	40.513595	C12orf75	2.439931045
3186	27.761477	11.379334	MTSS1L	2.439639846
3187	7.3592119	3.0194434	UBXN11	2.437274326
3188	22.391063	9.1942595	MCAM	2.435330725
3189	33.306706	13.676461	PML	2.435330725
3190	83.882299	34.450581	NID1	2.434858791
3191	48.97977	20.116828	CBX4	2.434765981
3192	11.761524	4.8343046	DTX2	2.432929808
3193	24.396878	10.03329	RFNG	2.431593035
3194	8.0444252	3.3097002	ARSJ	2.430560071
3195	64.284759	26.451035	UFD1L	2.430330524
3196	121.81098	50.150403	POLR2G	2.42891318
3197	7.3759536	3.0372896	RBM20	2.428465717
3198	236.93342	97.57084	CST3	2.428321987
3199	11.83341	4.8744485	PDLIM3	2.427640829
3200	4.6985865	1.9395586	RASSF5	2.422502966
3201	5.4256212	2.2424104	ENGASE	2.419548694
3202	7.6094046	3.1454758	INPP5E	2.419158508
3203	10.863643	4.4913169	TPCN2	2.418810127
3204	26.060181	10.778807	ABHD4	2.417723996
3205	54.450952	22.555896	RBM3	2.414045172
3206	0.2079242	0.0861807	AKAP2	2.412655407
3207	0.505824	0.2096545	SPDYE8P	2.412655399
3208	0.7702199	0.3192416	ROM1	2.412655396
3209	2.2408548	0.9287919	LOC100506713	2.412655395
3210	1.0726445	0.4445908	UPK3BL	2.412655394
3211	1.9633869	0.8137867	EID3	2.412655394
3212	1.1423622	0.4734875	SPIN2B	2.412655394
3213	3.4270865	1.4204625	RP2	2.412655394
3214	17.13711	7.1030078	ULBP2	2.412655394
3215	16.530272	6.8514849	SCARNA9	2.412655393
3216	9.5855212	3.9730171	HOXC13	2.412655393
3217	0.9279876	0.3846333	BTBD19	2.412655393
3218	0.6810441	0.2822799	BATF2	2.412655392
3219	2.3586039	0.9775967	WBP1	2.412655392
3220	0.5727509	0.2373944	TAPT1-AS1	2.412655392
3221	1.8630862	0.772214	NAT1	2.412655392
3222	1.1665706	0.4835214	GP1BA	2.412655392
3223	0.9250453	0.3834138	LOC155060	2.412655391

3224	0.4376827	0.1814112	ZNF836	2.412655389
3225	0.5511131	0.228426	OR7E156P	2.412655387
3226	28.215887	11.700343	RAB5B	2.411543569
3227	35.57575	14.758652	UGP2	2.410501237
3228	34.606138	14.383103	ATP6V1H	2.406027218
3229	12.841566	5.337464	ADCY9	2.40593022
3230	81.119992	33.716883	ABHD12	2.405916132
3231	23.1555	9.6267773	PPP1R37	2.405322094
3232	4.105827	1.7070891	LRRC16A	2.405162675
3233	6.8874035	2.8649303	TMEM62	2.404038767
3234	5.3170119	2.211903	AR	2.403817828
3235	238.14051	99.233038	SOD1	2.399810733
3236	75.45384	31.449472	CDC42EP4	2.399208502
3237	9.2982176	3.8775067	PPP2R3B	2.397988795
3238	21.684026	9.0429114	PLCB3	2.397903193
3239	28.4616	11.873898	EPDR1	2.3969888
3240	5.5581001	2.3198938	SLC27A1	2.395842464
3241	2.1147551	0.8831663	ZNF160	2.394515129
3242	776.95392	324.60242	GSTP1	2.393555566
3243	14.415932	6.023269	HEXIM1	2.393373433
3244	30.910931	12.922206	SH3BGRL	2.392078375
3245	29.7854	12.454136	MTFR1L	2.391607145
3246	45.607679	19.0717	ERCC2	2.391379773
3247	18.254691	7.6343882	EDN1	2.391113828
3248	32.879297	13.758531	MLXIP	2.389738834
3249	24.3924	10.211969	TRAPPC12	2.388608994
3250	12.913275	5.4080612	RHBDF2	2.387782657
3251	53.16741	22.268243	STAT3	2.387588844
3252	4.1349107	1.7328849	HSD17B6	2.386142697
3253	4.1603627	1.7435515	SYNGAP1	2.386142696
3254	2.3345724	0.9783876	FRRS1	2.386142695
3255	115.63095	48.473533	RRBP1	2.385444994
3256	153.58458	64.38593	MYADM	2.385374953
3257	17.690438	7.417119	TBC1D10A	2.385082188
3258	10.569612	4.4334094	KIF13B	2.384082125
3259	4.7404178	1.9895281	CHST7	2.382684519
3260	24.629032	10.339143	CAP2	2.382115451
3261	221.50124	93.052542	OST4	2.380388877
3262	15.665074	6.5824336	PEX16	2.37983015
3263	2.3576923	0.9913814	TLE6	2.378188888
3264	2.4593066	1.034109	CASD1	2.378188887
3265	1.5338203	0.6449531	OTUD1	2.378188887
3266	22.691737	9.5514918	UBQLN4	2.375726994

3267	62.797188	26.438786	PRDX2	2.3751918
3268	3.2640886	1.3760295	LOC100506730	2.372106562
3269	201.61804	85.008291	PDLIM1	2.371745594
3270	61.004962	25.737937	NDUFB2	2.370235078
3271	13.151853	5.5487548	PPP3CC	2.370235078
3272	58.15237	24.537888	MARCKS	2.36990114
3273	2.1134644	0.8924415	SARM1	2.368182482
3274	10.360432	4.3764675	TMEM128	2.367304727
3275	15.084879	6.3749927	CCDC115	2.366258174
3276	10.007886	4.2294141	CD83	2.366258173
3277	0.3505458	0.1483216	IKZF2	2.363417534
3278	1.1497903	0.4864948	IGIP	2.363417531
3279	1.1249636	0.4759902	FLJ43663	2.36341753
3280	3.5969709	1.5219363	CCZ1B	2.363417529
3281	17.393803	7.359598	EVI5L	2.363417528
3282	8.8680638	3.7522206	NAAA	2.363417528
3283	6.53162	2.7636336	TP53I3	2.363417528
3284	17.626742	7.4581582	ABHD16A	2.363417527
3285	1.3391133	0.5666004	LOC100996485	2.363417526
3286	96.575378	40.867804	TTYH3	2.363116379
3287	13.090094	5.5425658	ALAD	2.361738963
3288	9.7851531	4.1451439	PCCA	2.360630479
3289	6.110569	2.5889978	PAFAH2	2.360206363
3290	14.915198	6.3222148	RXRA	2.359172885
3291	3.084594	1.3084124	C9orf3	2.357508984
3292	23.133531	9.8160558	BTN2A1	2.356703273
3293	15.001687	6.3655391	SH2B1	2.356703273
3294	4.8077399	2.0458563	SETD4	2.34998902
3295	4.4828666	1.9076117	RPL23AP53	2.349989019
3296	72.237066	30.753772	GALNT2	2.348884766
3297	5.7442422	2.4474777	MYBL1	2.347004906
3298	14.295057	6.099296	SMIM19	2.343722382
3299	12.009463	5.1240978	PARP3	2.343722382
3300	58.208136	24.845985	VPS28	2.342758284
3301	57.78721	24.671653	PAM	2.342251251
3302	14.319311	6.1153545	GNAO1	2.341534033
3303	94.569659	40.393286	HSD17B10	2.341222221
3304	5.6042392	2.3948775	DECR2	2.340094329
3305	4.2409742	1.8123091	AGA	2.340094328
3306	5.4508385	2.3293243	GATSL3	2.340094328
3307	15.123281	6.4673079	ZSWIM8	2.338419842
3308	6.7119163	2.8731742	LINC00847	2.336063159
3309	54.096286	23.169283	BRI3	2.3348278

3310	14.266517	6.112803	TCEAL3	2.333874809
3311	70.910377	30.388441	IGF2R	2.333465391
3312	5.9287986	2.5435985	FOXD2	2.330870465
3313	6.6665305	2.8604448	PKP2	2.330592285
3314	33.023456	14.173548	PNRC1	2.329935779
3315	78.174614	33.580647	RABL6	2.327966265
3316	0.440074	0.189158	ENTPD1-AS1	2.326489134
3317	0.3737735	0.1606599	MYOM2	2.326489132
3318	0.7944072	0.3414618	ZNF429	2.326489131
3319	0.9298937	0.3996983	TPI1P2	2.32648913
3320	2.537446	1.0906761	RUFY3	2.32648913
3321	1.6196852	0.6961929	EPM2A	2.32648913
3322	10.492255	4.5099093	TMEM63A	2.32648913
3323	15.863246	6.8185341	SPPL2A	2.32648913
3324	0.9545015	0.4102755	LOC100133612	2.326489129
3325	2.4311024	1.0449662	RASL11A	2.326489129
3326	4.7603947	2.046171	TFEB	2.326489129
3327	2.3938679	1.0289616	WHAMMP1	2.326489129
3328	2.8119873	1.2086827	MRPL42P5	2.326489128
3329	0.6793175	0.2919925	GSG1	2.326489128
3330	0.3324345	0.1428911	BMP8A	2.326489127
3331	0.4563493	0.1961536	ZNF615	2.326489121
3332	0.1585524	0.0681509	HYDIN2	2.326489117
3333	0.0232537	0.01	ATP2B2	2.3253682
3334	1.4954347	0.6437514	SHOX	2.323000078
3335	10.288235	4.4289563	SLC12A9	2.32294805
3336	11.017027	4.743111	TCF7L1	2.322742769
3337	94.075709	40.520457	SLC35A4	2.321684319
3338	9.9237857	4.2761212	MAN2B2	2.320744711
3339	49.279654	21.245784	GTF3A	2.319502675
3340	97.315332	41.979731	SEPW1	2.318150459
3341	7.7761008	3.3552741	IFI44	2.317575378
3342	13.882086	5.9903595	RNF213	2.317404507
3343	44.549169	19.229807	LINC00152	2.31667272
3344	10.642116	4.59653	AGXT2L2	2.315250051
3345	8.2360493	3.55782	MYO18A	2.314914556
3346	8.4094022	3.6329804	PASK	2.314739184
3347	64.334057	27.839889	USB1	2.31085897
3348	81.259121	35.168192	DPYSL3	2.310585658
3349	102.69191	44.515518	AP3S1	2.306878877
3350	25.848	11.21242	GLTPD1	2.305300704
3351	152.32517	66.09633	TMEM109	2.304593529
3352	84.28897	36.586711	PTOV1	2.303813796

3353	184.22466	79.971692	GNB2	2.303623419
3354	32.8354	14.256191	C15orf52	2.303237916
3355	26.703	11.602569	TRPT1	2.301473117
3356	2.4809464	1.0797206	ZNF222	2.297767041
3357	1.8540881	0.8069086	FAM156A	2.297767041
3358	3.2172829	1.400178	COLGALT2	2.29776704
3359	3.1719863	1.3804647	HOOK2	2.29776704
3360	48.952648	21.342949	TIMP3	2.293621585
3361	6.4449613	2.8102846	NAV2	2.293348258
3362	4.8516621	2.1160684	TGFBR3	2.292771895
3363	38.673255	16.869644	SHARPIN	2.29247613
3364	87.522453	38.193446	MIEN1	2.29155686
3365	14.866884	6.4892299	SAC3D1	2.291008904
3366	71.169204	31.084218	AMFR	2.289560731
3367	1.2568841	0.548963	NECAB1	2.289560729
3368	100.32921	43.858321	CD276	2.287575332
3369	6.880671	3.0081768	MLYCD	2.287322646
3370	0.0228709	0.01	ABCA12	2.2870885
3371	13.67664	5.9805187	IL7R	2.286865226
3372	5.7764587	2.5272457	TK2	2.285673531
3373	11.192812	4.8969426	PYCARD	2.28567353
3374	6.1552595	2.692974	AVPI1	2.28567353
3375	10.120784	4.429623	ZDHHC14	2.284795776
3376	10.804699	4.7329103	PKD1	2.282886923
3377	15.599963	6.834316	SLC22A18	2.282593109
3378	6.6191405	2.9006886	TNFAIP8	2.281920372
3379	14.703634	6.4464975	ATG2A	2.280871696
3380	22.132487	9.7035214	SLC35C1	2.280871695
3381	77.461559	33.992507	VPS51	2.278783343
3382	11.750658	5.160018	NKX3-1	2.277251263
3383	94.500967	41.528156	NANS	2.275587822
3384	0.0227486	0.01	COL6A4P2	2.2748554
3385	0.6913132	0.3039021	SAMD14	2.274789374
3386	12.147435	5.3400262	ZNHIT2	2.274789371
3387	2.9364575	1.2908701	SLC39A4	2.274789371
3388	5.1514483	2.2645826	HIST1H2BG	2.274789371
3389	4.1566537	1.8272697	KIFC2	2.274789371
3390	8.6750975	3.8135827	ABHD8	2.27478937
3391	0.8816902	0.387592	MATN3	2.274789369
3392	46.256324	20.345149	SYNGR2	2.273580019
3393	13.18684	5.8033644	LAMA5	2.272275097
3394	34.70967	15.279142	FAM53C	2.271702818
3395	0.3969514	0.174803	ERBB3	2.270850338

3396	10.075294	4.4446514	LIN37	2.266835561
3397	9.5042671	4.1971647	JPH2	2.264449419
3398	39.800888	17.594136	TPRG1L	2.262167833
3399	2.6698325	1.1803681	ZNF155	2.261864432
3400	597.98454	264.51214	P4HB	2.26070737
3401	16.853354	7.4623107	PDGFC	2.258463132
3402	62.346665	27.636062	LOC550643	2.255989459
3403	14.497357	6.4261632	SNHG9	2.255989458
3404	45.077388	20.008098	JOSD1	2.252957215
3405	130.37256	57.873704	HN1	2.25270816
3406	37.912495	16.834088	CYB5A	2.252126463
3407	43.564379	19.35057	AGRN	2.251322813
3408	6.1636943	2.7388596	AKR1C3	2.250460072
3409	158.2129	70.314009	AKR1B1	2.250090701
3410	8.3321491	3.7049237	SLC2A8	2.248939491
3411	5.2859348	2.3504122	TRIM6	2.248939491
3412	69.64341	30.967878	MAPKAPK2	2.248891886
3413	2.1808236	0.9701968	NACAD	2.247815585
3414	14.665693	6.5244201	TM2D3	2.247815584
3415	4.6229467	2.058059	ENDOV	2.246265366
3416	0.7791756	0.3477961	TMEM169	2.240322868
3417	1.1039908	0.492782	XKR5	2.240322866
3418	10.687154	4.7703632	PPAPDC1A	2.240322866
3419	3.9107809	1.7456327	CLTCL1	2.240322866
3420	25.85368	11.540158	HOMER3	2.240322865
3421	7.5046911	3.3498257	STK19	2.240322865
3422	1.2047107	0.5377398	LYST	2.240322864
3423	1.265387	0.5648235	KCNMB3	2.240322864
3424	9.0608478	4.0476483	F11R	2.238546241
3425	9.9166309	4.4320183	RCCD1	2.237497742
3426	80.702304	36.073718	LAMC1	2.237149603
3427	18.371852	8.2123456	NISCH	2.237101697
3428	9.8882484	4.4207003	PIM2	2.236805875
3429	2.8060161	1.2547499	UNC5B	2.236315131
3430	9.3295803	4.1730668	PTGES	2.235665229
3431	3.1412298	1.4056205	ABCG2	2.234763751
3432	5.4103716	2.4224501	OGFOD2	2.233429564
3433	2.9019012	1.2993028	ITGAE	2.233429563
3434	2.1138989	0.9474045	GJD3	2.231252733
3435	2.3557705	1.0558062	LRP5L	2.231252732
3436	4.6818518	2.0983064	THAP8	2.231252732
3437	2.3297558	1.044147	ADAMTS9	2.231252731
3438	18.720003	8.3928312	HEIH	2.230475292

3439	24.003649	10.762264	AK1	2.230353215
3440	13.779327	6.1836665	SNX19	2.228342528
3441	9.6148997	4.3157045	CPD	2.227886497
3442	13.138612	5.8995148	DRAM1	2.227066517
3443	3.1287861	1.4048912	EID2B	2.227066517
3444	6.8730106	3.0861272	PRR24	2.227066516
3445	2.7182544	1.2205537	FMN2	2.227066516
3446	82.17121	36.899349	CPT1A	2.226901329
3447	14.165978	6.3677156	TANK	2.224656272
3448	53.052249	23.850669	EHBP1L1	2.224350582
3449	259.32002	116.60791	SLC25A6	2.223863026
3450	41.337519	18.60142	DUSP3	2.222277574
3451	0.0222127	0.01	STXBP5L	2.2212695
3452	1.8540881	0.8367941	CA11	2.215703933
3453	67.640182	30.540472	NECAP2	2.214771985
3454	371.67598	167.89812	PTMS	2.213699468
3455	45.010079	20.335018	GNS	2.213427036
3456	8.9634194	4.0516865	NIT1	2.212268732
3457	59.957329	27.110376	NCSTN	2.211600777
3458	15.763321	7.1298203	PLCD3	2.210900238
3459	1.4502186	0.6565076	DDX60L	2.208989679
3460	5.9415151	2.6904047	TBX18	2.208409433
3461	2.9313336	1.3288869	ZNF775	2.20585636
3462	145.01919	65.761835	GRINA	2.20521809
3463	516.81965	234.36483	MYH9	2.205192859
3464	4.6402253	2.1048347	AHCYL2	2.204555736
3465	2.3879416	1.0834373	AGO4	2.204042334
3466	19.560964	8.8768366	AIG1	2.203596261
3467	34.050643	15.468096	ST3GAL1	2.201346722
3468	12.273233	5.5787684	TBX2	2.199989721
3469	19.913118	9.0523472	TANGO2	2.199774035
3470	18.276494	8.3098315	CYBRD1	2.199381988
3471	1.433458	0.6523904	TRAF3IP2-AS1	2.197239734
3472	2.506101	1.1405679	AXIN2	2.197239733
3473	6.298296	2.8664583	IFT20	2.197239733
3474	2.9622003	1.3481461	RBKS	2.197239732
3475	1.8558363	0.8446217	LHX4	2.197239732
3476	2.9375978	1.3383303	DNAL1	2.1949722
3477	8.7733965	4.0000475	SAR1B	2.193323085
3478	57.062591	26.021279	CHPF	2.192920345
3479	35.750542	16.303731	ZBTB8OS	2.192782857
3480	30.17807	13.766929	USF1	2.192069757
3481	17.197548	7.848873	ZER1	2.191085

3482	43.806225	19.992937	TOR1A	2.191085
3483	2.1796578	0.9954426	GUSBP11	2.189636827
3484	23.258643	10.623532	IPO13	2.189351273
3485	17.913997	8.1932954	ERAP1	2.186421502
3486	6.2629876	2.8648333	AKNA	2.186161214
3487	0.0218402	0.01	DNAH12	2.1840233
3488	2.7516265	1.2605494	JMJD7	2.182878689
3489	4.1353426	1.8944445	TSNARE1	2.182878689
3490	1.116321	0.5113985	CHRD	2.182878688
3491	33.002066	15.131983	ALKBH3	2.180947792
3492	14.28057	6.5544015	COMMD6	2.178775534
3493	19.064563	8.7579396	SIRT7	2.176831933
3494	40.864348	18.79547	NRSN2	2.174159484
3495	3.686908	1.6965791	CRTC1	2.173142388
3496	39.898708	18.374733	STARD3NL	2.171389855
3497	23.914697	11.013544	HHLA3	2.171389854
3498	2.2500717	1.0362357	LOC100506746	2.171389854
3499	1.03363	0.4760223	EPHB3	2.171389852
3500	47.881929	22.059963	PXDC1	2.170535312
3501	10.046417	4.6307803	AAK1	2.169486796
3502	52.395601	24.158824	PLOD3	2.168797665
3503	2.8485797	1.3148508	MOSPD2	2.166466068
3504	70.341139	32.50142	LARP6	2.164248145
3505	9.1773747	4.241459	TOR2A	2.16373063
3506	9.1748208	4.241573	NEK7	2.163070352
3507	1.0723017	0.495979	NLRP3	2.161989899
3508	2.5590969	1.1836766	C4orf33	2.161989898
3509	4.2568282	1.9689399	MXD1	2.161989898
3510	4.8711265	2.2530755	HKR1	2.161989898
3511	3.8988065	1.8050857	PPARA	2.159901019
3512	26.202601	12.133674	GTPBP6	2.159494333
3513	16.135621	7.4728755	SMIM11	2.159225205
3514	30.628087	14.189134	GPR137	2.158559257
3515	15.141033	7.0165497	YIPF4	2.15790296
3516	3.8326345	1.7760921	SMCR7	2.15790296
3517	30.399305	14.09582	DGCR2	2.156618495
3518	17.738335	8.2264427	SCOC	2.156258217
3519	11.258628	5.2220225	RBM4B	2.155989925
3520	27.881314	12.967075	KAT5	2.150162138
3521	15.767557	7.3368595	STRADA	2.149087998
3522	263.99252	122.91256	TMED9	2.147807509
3523	3.3452952	1.557742	HOPX	2.147528426
3524	103.52604	48.21511	CHMP2A	2.147170147

3525	21.712444	10.112819	PNKD	2.147021815
3526	17.782267	8.2842247	TMEM159	2.146521615
3527	334.34377	156.01172	TPM1	2.143068347
3528	29.347217	13.69421	RMND5B	2.143038373
3529	0.8227043	0.3841097	PLXNC1	2.141847133
3530	66.791496	31.206365	NKIRAS2	2.140316424
3531	6.572486	3.0710693	MOB3C	2.140129534
3532	60.714815	28.407138	CDKN2AIPNL	2.137308449
3533	18.309301	8.5717047	LOC100507217	2.136016335
3534	173.06761	81.030316	PTTG1IP	2.135837788
3535	310.23107	145.38929	GNAI2	2.133795953
3536	6.2068443	2.9104382	TMEM234	2.132615035
3537	5.8728776	2.755123	MBNL2	2.131620809
3538	5.1332919	2.4088214	IQCE	2.131038823
3539	5.4316466	2.5492829	CEP89	2.130656711
3540	17.889122	8.3960601	TNFAIP3	2.13065671
3541	67.888624	31.872144	ADRBK1	2.130030047
3542	16.474897	7.7360765	NFKBIZ	2.129619188
3543	75.583255	35.493909	DEGS1	2.129471131
3544	11.299741	5.3079991	FAM98C	2.128813582
3545	37.545351	17.640949	UROS	2.128306722
3546	211.71796	99.490179	ATP5H	2.128028767
3547	10.039989	4.7200901	TMCO4	2.127075775
3548	20.205799	9.500167	BAIAP2	2.126888795
3549	13.839029	6.5130874	IFFO1	2.124803259
3550	7.7916064	3.6672465	ZDHHC2	2.124647606
3551	64.541003	30.388225	SDHAF2	2.123881968
3552	23.394922	11.01517	TMEM51	2.123881967
3553	62.681548	29.528461	LGMN	2.122750206
3554	14.605777	6.8905247	OGFRL1	2.119690096
3555	48.347826	22.808913	ATPIF1	2.119690096
3556	3.5527566	1.6760736	SLFN12	2.119690095
3557	11.03925	5.2079547	LY6G5B	2.119690095
3558	6.0656819	2.8632924	FAM114A2	2.118429126
3559	79.224564	37.419001	ATP6V1B2	2.117228202
3560	18.467794	8.725015	TFPI	2.116648933
3561	15.041507	7.1069213	JMJD6	2.11645886
3562	3.5569799	1.681795	ARL17A	2.114990118
3563	3.3576719	1.5875591	ABAT	2.114990118
3564	277.10514	131.34827	PRDX5	2.109697705
3565	42.066013	19.948422	FTSJ1	2.108738915
3566	41.991409	19.913771	EMC1	2.108661856
3567	73.761472	35.036095	TSPO	2.105299441

3568	81.321761	38.647718	PGLS	2.104180167
3569	30.265488	14.384328	FZR1	2.104059936
3570	34.353492	16.3295	XYLT2	2.103768717
3571	62.238772	29.643197	TMEM9	2.099597176
3572	11.251222	5.3594512	FLCN	2.099323524
3573	6.3530977	3.0288427	DPF1	2.097533056
3574	107.7989	51.448187	PSME1	2.09529054
3575	87.850604	41.933933	AP2A1	2.094976475
3576	157.80251	75.338198	ATP6V1F	2.094588283
3577	6.0258347	2.8787534	GLTSCR1	2.093209732
3578	42.697096	20.403759	GOSR2	2.092609269
3579	5.3918886	2.5782306	SDK1	2.091313536
3580	20.536946	9.8223372	KCTD2	2.090841059
3581	4.1968717	2.0076226	PRRG1	2.090468493
3582	40.632666	19.445822	NENF	2.089531903
3583	7.1765792	3.4366233	C21orf58	2.088264752
3584	81.516266	39.069275	SNX17	2.086454536
3585	28.868162	13.844157	B4GALT7	2.085223591
3586	19.155137	9.1950712	FUNDC2	2.083196148
3587	29.443216	14.136623	SIL1	2.082761696
3588	42.921174	20.610383	ENTPD6	2.08250255
3589	7.3826737	3.5460154	POLH	2.081963245
3590	11.842573	5.6905933	NAGLU	2.081078884
3591	122.66034	58.983163	H1FX	2.07958221
3592	0.0207714	0.01	WDFY4	2.0771404
3593	30.427817	14.653658	CHMP6	2.076465707
3594	55.636681	26.837964	TMEM214	2.073058941
3595	33.454725	16.143062	ELK1	2.072390316
3596	29.429057	14.201042	TPBG	2.072316676
3597	57.997023	28.012616	DPP3	2.070389398
3598	0.0438274	0.0211933	CALN1	2.067990361
3599	0.1107624	0.0535604	SPATA6L	2.067990361
3600	0.0847669	0.04099	TMC5	2.067990359
3601	0.1321074	0.063882	AGAP11	2.067990358
3602	0.1256554	0.0607621	TCAM1P	2.067990356
3603	0.1139729	0.0551129	FFAR4	2.067990353
3604	0.1653964	0.0799793	FAM133CP	2.067990352
3605	0.1526177	0.0738	GSDMC	2.06799035
3606	0.1233985	0.0596707	SLC7A14	2.06799035
3607	0.1296017	0.0626704	DERL3	2.06799035
3608	0.1547138	0.0748136	LOC641367	2.067990349
3609	0.1097128	0.0530529	LOC339505	2.067990349
3610	0.1274225	0.0616166	PCDHB11	2.067990348

3611	0.0912232	0.044112	KIAA1107	2.067990348
3612	0.1218354	0.0589149	KCNH6	2.067990348
3613	0.2467726	0.1193297	OTUD6A	2.067990347
3614	0.1715928	0.0829756	SLC52A1	2.067990347
3615	0.1464508	0.0708179	PRSS36	2.067990346
3616	0.3098877	0.1498497	LINC00852	2.067990346
3617	0.0983364	0.0475517	FRMPD4	2.067990346
3618	0.2373571	0.1147767	RSAD2	2.067990346
3619	0.1728022	0.0835605	PIPOX	2.067990345
3620	0.1728022	0.0835605	RRN3P2	2.067990345
3621	0.1399124	0.0676562	LOC643623	2.067990345
3622	0.3108121	0.1502967	PDZD9	2.067990345
3623	0.2044134	0.0988464	BCL2L14	2.067990344
3624	0.0895572	0.0433064	CIITA	2.067990344
3625	0.2391274	0.1156327	LOC100506314	2.067990344
3626	0.1770599	0.0856193	CYP2W1	2.067990343
3627	0.3377626	0.1633289	SMG7-AS1	2.067990343
3628	0.185988	0.0899366	CRX	2.067990343
3629	0.2841166	0.1373878	LOC100996291	2.067990343
3630	0.1645476	0.0795688	CHADL	2.067990342
3631	0.2582398	0.1248748	RBM14-RBM4	2.067990342
3632	0.1992347	0.0963422	LINC00520	2.067990342
3633	0.4570164	0.2209954	OXCT2	2.067990342
3634	0.3660949	0.1770293	PAQR6	2.067990342
3635	0.244744	0.1183487	TMEM178A	2.067990342
3636	0.2649146	0.1281024	KCND1	2.067990342
3637	0.1657252	0.0801383	LHX9	2.067990342
3638	0.4170075	0.2016487	FGF18	2.067990341
3639	0.3845009	0.1859297	FLRT1	2.067990341
3640	0.1081191	0.0522822	FAM184B	2.067990341
3641	0.442932	0.2141847	LOC100128750	2.067990341
3642	0.3380365	0.1634614	ZNF205-AS1	2.067990341
3643	0.1187462	0.057421	LOC100287846	2.067990341
3644	0.5764855	0.278766	LINC00266-1	2.067990341
3645	0.1730893	0.0836993	NRSN1	2.06799034
3646	0.2121115	0.1025689	LOC100130476	2.06799034
3647	0.061204	0.0295959	AFAP1-AS1	2.06799034
3648	0.1801206	0.0870993	MYH11	2.06799034
3649	0.3347783	0.1618858	PNOC	2.06799034
3650	0.8925032	0.43158	HIST1H2BO	2.06799034
3651	0.1420099	0.0686705	LOC100506895	2.06799034
3652	0.200577	0.0969913	CPA5	2.06799034
3653	0.4368962	0.2112661	TAS2R14	2.06799034

3654	0.1282458	0.0620147	ZNF252P-AS1	2.067990339
3655	0.4638831	0.2243159	LINC00311	2.067990339
3656	1.0342407	0.5001187	ATL1	2.067990339
3657	0.449864	0.2175368	MARCO	2.067990339
3658	0.2918761	0.1411399	BCRP3	2.067990339
3659	1.003644	0.4853233	MAB21L1	2.067990339
3660	0.2056236	0.0994316	DYNAP	2.067990339
3661	0.4492982	0.2172632	FES	2.067990339
3662	0.3364674	0.1627026	ZNF493	2.067990339
3663	0.1151219	0.0556685	KIAA0825	2.067990339
3664	0.2675218	0.1293632	FBXL13	2.067990339
3665	0.272062	0.1315586	PRR19	2.067990339
3666	0.5206733	0.2517774	VIP	2.067990339
3667	0.8593794	0.4155626	ODF3B	2.067990339
3668	0.7939029	0.3839007	PIP5KL1	2.067990339
3669	0.1534606	0.0742076	C16orf71	2.067990339
3670	0.8683313	0.4198913	C2orf82	2.067990339
3671	0.2060302	0.0996282	SH3PXD2A-AS1	2.067990339
3672	1.1171083	0.5401903	CCPG1	2.067990339
3673	0.7933991	0.3836571	LOC100506305	2.067990339
3674	1.8310774	0.8854381	UBAC2-AS1	2.067990338
3675	0.5655346	0.2734706	PAN3-AS1	2.067990338
3676	0.0992615	0.047999	CILP2	2.067990338
3677	0.5731165	0.277137	DGKG	2.067990338
3678	0.6997185	0.3383568	MIPEPP3	2.067990338
3679	0.5614715	0.2715059	LOC284865	2.067990338
3680	1.2104521	0.5853277	FUT2	2.067990338
3681	0.1535737	0.0742623	C18orf61	2.067990338
3682	0.2554698	0.1235353	ELF3	2.067990338
3683	1.820083	0.8801216	PRH1-PRR4	2.067990338
3684	0.4462516	0.21579	MYL4	2.067990338
3685	2.9352042	1.419351	PI4KAP2	2.067990338
3686	1.5234537	0.7366832	TXNDC16	2.067990337
3687	0.2587207	0.1251073	LOC100506124	2.067990337
3688	1.0854141	0.5248642	PTCSC3	2.067990337
3689	1.38933	0.6718262	PAM16	2.067990337
3690	0.5738398	0.2774867	WWC2-AS2	2.067990337
3691	5.8052047	2.8071721	NFATC4	2.067990337
3692	1.5908359	0.7692666	LOC100131691	2.067990337
3693	24.592413	11.891938	SAT2	2.067990337
3694	1.119675	0.5414314	FAM221A	2.067990337
3695	1.8307423	0.885276	TMSB15B	2.067990337
3696	0.143823	0.0695472	RIMBP3	2.067990337

3697	0.2088695	0.1010012	NCMAP	2.067990337
3698	0.1514531	0.0732369	HSP90B2P	2.067990337
3699	0.5405953	0.261411	MYLK-AS1	2.067990337
3700	9.7517964	4.7155909	LMBRD1	2.067990337
3701	1.4949749	0.7229119	SOX15	2.067990337
3702	8.8838153	4.2958689	SAP30	2.067990337
3703	1.4450684	0.6987791	C11orf71	2.067990337
3704	7.6336813	3.6913525	HIST1H2AC	2.067990337
3705	4.7517801	2.2977768	KCNAB2	2.067990337
3706	0.1351927	0.0653739	GPD1	2.067990337
3707	1.8260635	0.8830136	LOC100505865	2.067990337
3708	3.0582736	1.4788626	LOC729970	2.067990337
3709	9.4727046	4.5806329	VTRNA1-3	2.067990337
3710	0.3176822	0.1536188	LOC100130275	2.067990337
3711	4.2176089	2.0394723	POLR2J3	2.067990337
3712	6.4603845	3.1239916	EFHD1	2.067990337
3713	0.3164761	0.1530356	SLC10A5	2.067990337
3714	2.8431037	1.3748148	IFITM1	2.067990337
3715	17.028753	8.2344451	GNB5	2.067990337
3716	45.142918	21.829366	METRNL	2.067990337
3717	10.156982	4.9115228	LYRM5	2.067990337
3718	3.6950266	1.7867717	S100A4	2.067990337
3719	0.4472092	0.2162531	LY6G6C	2.067990337
3720	2.7709629	1.3399303	RNF152	2.067990336
3721	55.848552	27.006196	GUSB	2.067990336
3722	19.087381	9.2299181	SNX11	2.067990336
3723	0.1528416	0.0739083	GOLGA8S	2.067990336
3724	1.7439289	0.8432964	HIST1H3J	2.067990336
3725	0.4857797	0.2349043	NPPA	2.067990336
3726	1.178508	0.5698808	TVP23C	2.067990336
3727	0.2756607	0.1332988	TNFRSF10C	2.067990336
3728	27.2114	13.158379	CDK5RAP3	2.067990336
3729	2.022887	0.9781898	DISP1	2.067990336
3730	2.1456834	1.0375694	HIST1H1D	2.067990336
3731	0.3392287	0.1640379	HRH4	2.067990336
3732	2.1103747	1.0204954	APOM	2.067990336
3733	0.4054465	0.1960582	WDR86	2.067990336
3734	0.3430444	0.165883	C21orf49	2.067990336
3735	0.5853919	0.2830728	GATM-AS1	2.067990336
3736	0.5853919	0.2830728	MBL1P	2.067990336
3737	0.1342348	0.0649107	LOC648691	2.067990336
3738	0.1029642	0.0497895	STX16-NPEPL1	2.067990336
3739	0.7690018	0.3718595	HNRNPA1P33	2.067990336

3740	0.7429572	0.3592653	HLA-DMA	2.067990336
3741	0.2112514	0.102153	NCCRP1	2.067990336
3742	0.1265712	0.0612049	MSH4	2.067990336
3743	0.574367	0.2777416	FLJ31813	2.067990335
3744	1.8415272	0.8904912	TRAM1L1	2.067990335
3745	0.4738147	0.2291184	INMT	2.067990335
3746	0.2975011	0.14386	ASB18	2.067990335
3747	0.2407851	0.1164343	YPEL4	2.067990335
3748	0.3464663	0.1675377	OR7E37P	2.067990335
3749	0.4263928	0.2061871	TCEANC	2.067990335
3750	0.6427124	0.3107908	LOC284581	2.067990335
3751	0.13074	0.0632208	LOC100287225	2.067990335
3752	0.776162	0.3753219	FMO4	2.067990335
3753	0.1803544	0.0872124	LOC340508	2.067990335
3754	1.0456573	0.5056394	ZNF254	2.067990335
3755	0.1174744	0.056806	B3GALT2	2.067990334
3756	0.2021334	0.0977439	CSTF3-AS1	2.067990334
3757	0.2162942	0.1045915	CXorf21	2.067990334
3758	0.2162942	0.1045915	WNT11	2.067990334
3759	0.3343755	0.161691	BCL2L15	2.067990334
3760	0.2861844	0.1383877	EMX2OS	2.067990334
3761	0.6788257	0.3282538	LOC338817	2.067990334
3762	0.7058408	0.3413173	LOC100507564	2.067990334
3763	0.5002088	0.2418816	CCDC146	2.067990334
3764	0.2798561	0.1353276	CACNB2	2.067990333
3765	0.265731	0.1284972	FUT6	2.067990333
3766	0.1583583	0.0765759	IL21R-AS1	2.067990333
3767	0.386641	0.1869646	SUGT1P3	2.067990333
3768	0.3408005	0.1647979	UBE2Q2P3	2.067990333
3769	0.4520597	0.2185985	C4orf36	2.067990333
3770	0.1345815	0.0650784	GFAP	2.067990332
3771	0.2341567	0.1132291	ZMYND10	2.067990332
3772	0.3814745	0.1844663	IRF4	2.067990332
3773	0.218907	0.105855	MRPL23-AS1	2.067990332
3774	0.1899722	0.0918632	LOC100506834	2.067990331
3775	0.1301683	0.0629444	TMPRSS11BNL	2.067990331
3776	0.0970428	0.0469262	PIGR	2.067990331
3777	0.2314264	0.1119089	LOC553103	2.06799033
3778	0.2042131	0.0987496	TLDC2	2.06799033
3779	0.1506321	0.0728398	EPGN	2.06799033
3780	0.1935899	0.0936126	AQP11	2.067990329
3781	0.0867247	0.0419367	PNPLA7	2.067990329
3782	0.168608	0.0815323	LCP2	2.067990329

3783	0.1000238	0.0483676	SLC14A2	2.067990329
3784	0.0690065	0.0333688	RASSF6	2.067990328
3785	0.1779671	0.086058	C5AR1	2.067990328
3786	0.1057865	0.0511543	ARMCX5-GPRASP2	2.067990328
3787	0.121233	0.0586236	ANKRD30BP2	2.067990328
3788	0.1429842	0.0691416	CCDC87	2.067990328
3789	0.1677935	0.0811384	GGT8P	2.067990326
3790	0.1258451	0.0608538	LOC100128682	2.067990326
3791	0.13715	0.0663205	SLC1A6	2.067990326
3792	0.1324012	0.0640241	LOC100130331	2.067990326
3793	0.0953337	0.0460997	KCNA7	2.067990326
3794	0.1650689	0.0798209	C17orf77	2.067990325
3795	0.1972546	0.0953847	SIGLEC14	2.067990325
3796	0.1719468	0.0831468	SCIMP	2.067990324
3797	0.1915437	0.0926231	CCDC151	2.067990324
3798	0.0892312	0.0431488	SEPT5-GP1BB	2.067990323
3799	0.1118323	0.0540778	LOC286190	2.067990322
3800	0.0529403	0.0255999	HYDIN	2.067990321
3801	0.07491	0.0362236	PCYT1B	2.067990321
3802	0.1569273	0.075884	NEK10	2.06799032
3803	0.1129231	0.0546052	PIWIL3	2.067990319
3804	0.0417676	0.0201972	CR1	2.067990319
3805	0.1044086	0.0504879	HOXA-AS3	2.067990319
3806	0.1334611	0.0645366	LOC100505989	2.067990318
3807	0.0490698	0.0237283	SRRM4	2.067990318
3808	0.101584	0.0491221	LOC100507557	2.067990316
3809	0.0546693	0.026436	PKD1L2	2.067990313
3810	123.89706	60.072438	UBE2Z	2.062460951
3811	189.95508	92.126836	LMAN2	2.06188647
3812	262.39849	127.28406	CALM3	2.061518879
3813	91.807352	44.548625	SDC4	2.060834661
3814	34.53332	16.769287	NFIX	2.059319517
3815	105.72348	51.350492	CLTB	2.058860138
3816	52.999182	25.744317	LSM10	2.058675065
3817	20.231142	9.8282883	ST3GAL2	2.058460428
3818	19.504425	9.4780454	ORMDL3	2.05785313
3819	70.324926	34.197995	CCND3	2.056404957
3820	52.018086	25.312132	CCDC167	2.055065397
3821	54.151051	26.361683	TCF25	2.054157625
3822	9.0367925	4.3995695	SLC6A9	2.054017429
3823	103.50779	50.3985	ATN1	2.053787106
3824	59.081609	28.773161	ITFG3	2.05335833
3825	12.402577	6.0411822	PSEN2	2.053004899

3826	13.406687	6.5309762	FAM222B	2.052784526
3827	218.78808	106.65309	PTRF	2.051399317
3828	11.086998	5.4048298	TSC22D3	2.051312996
3829	21.085227	10.279232	MAP3K3	2.051245476
3830	36.195703	17.647491	TSEN15	2.051039597
3831	127.11603	62.02935	COL6A3	2.049288402
3832	18.726748	9.1386081	SERTAD1	2.049190425
3833	3.367882	1.6437976	EPB41L5	2.048842279
3834	4.4731688	2.1832665	HERC3	2.048842278
3835	4.9161868	2.3999182	DPYD	2.048480994
3836	8.5254341	4.1642117	TMEM55A	2.047310434
3837	22.075249	10.785354	CC2D1A	2.04678018
3838	12.961679	6.3330551	LINC00707	2.046670849
3839	21.426356	10.470018	POPDC3	2.046448772
3840	25.818192	12.617493	COG1	2.046222018
3841	168.86346	82.530949	SNX3	2.046062218
3842	33.241638	16.25101	NUDT22	2.04551218
3843	80.153654	39.187217	MRPL49	2.045403064
3844	20.25151	9.9037079	TICAM1	2.044841192
3845	20.061885	9.8118095	TMEM185A	2.044667137
3846	48.236613	23.604703	TSSC4	2.043517079
3847	60.994976	29.85894	VPS29	2.042770943
3848	29.434124	14.411563	MIER2	2.042396397
3849	24.821939	12.161278	SH3BP5L	2.041063379
3850	24.338412	11.926416	ITGAV	2.040714685
3851	37.750333	18.503687	PLBD2	2.040152006
3852	24.627643	12.075146	ARHGEF40	2.039531755
3853	22.558255	11.07874	HCCS	2.036175101
3854	81.741163	40.153027	BCL2L1	2.035740975
3855	225.08735	110.66879	PLP2	2.033882857
3856	20.209483	9.9395747	CDKN1B	2.033234197
3857	10.112974	4.9775681	ENTHD2	2.031709804
3858	7.7000157	3.7923817	SCML1	2.030390513
3859	16.303509	8.030128	WDTC1	2.030292596
3860	12.390074	6.1029998	HABP4	2.030161245
3861	7.7336478	3.8116096	LINC00674	2.028971652
3862	16.959681	8.3605978	ZNF362	2.028524873
3863	80.535817	39.70265	UXT	2.028474598
3864	102.53522	50.547943	FAM195B	2.028474597
3865	7.4376309	3.6670707	HBP1	2.028221292
3866	37.243494	18.374461	EXTL3	2.026916262
3867	3.8418268	1.895672	ABHD13	2.02663053
3868	5.677829	2.8016103	FAM73B	2.02663053

3869	27.57215	13.610591	KIRREL	2.025786453
3870	20.181461	9.9653558	UBQLN2	2.025162135
3871	38.418476	18.978643	DUSP14	2.024300401
3872	53.418471	26.394583	LIMK1	2.023842229
3873	5.1368715	2.538386	FAM13B	2.023676259
3874	19.295725	9.5361864	SYNM	2.02342158
3875	4.2559462	2.1037443	ACOT13	2.023034026
3876	8.33598	4.1205338	FLT3LG	2.023034025
3877	11.131131	5.5021964	BBS1	2.023034025
3878	54.101384	26.74791	NDUFA8	2.022639671
3879	24.334621	12.032707	NQO2	2.022372904
3880	72.478845	35.842499	LTBR	2.022148187
3881	80.373302	39.748205	CLIC4	2.022061166
3882	86.423811	42.752574	RPS6KB2	2.021487909
3883	3.9389796	1.9490341	ASB16-AS1	2.020990557
3884	30.688389	15.193691	ATXN7L3B	2.01981135
3885	14.864273	7.3616844	CDYL	2.019140171
3886	14.996819	7.4279747	PKD1P1	2.018964704
3887	5.0483778	2.5007414	WNT3	2.018752472
3888	78.06002	38.673103	PIEZO1	2.018457634
3889	32.818819	16.266657	RNPEPL1	2.017551548
3890	17.397341	8.6265616	MKNK1	2.01671785
3891	1.6785586	0.8324983	ZKSCAN3	2.016290579
3892	4.4195653	2.1919287	ZNF688	2.016290578
3893	57.469974	28.502823	VEGFA	2.016290578
3894	2.4779209	1.2289503	ZNF189	2.016290578
3895	12.055303	5.9815508	C22orf39	2.015414312
3896	3.5120845	1.7442082	SFI1	2.013569538
3897	359.12448	178.36093	MYL12A	2.013470592
3898	0.0201333	0.01	NFASC	2.0133272
3899	15.296664	7.6004575	CTDSPL	2.012597739
3900	2.8772318	1.4299656	MAP3K5	2.012098707
3901	5.1106144	2.5399422	BDH2	2.012098706
3902	21.977968	10.937238	CD55	2.009462309
3903	23.850165	11.868929	C19orf60	2.009462308
3904	26.169323	13.023944	NPEPPS	2.009323945
3905	70.670128	35.171097	COA3	2.009323944
3906	57.431611	28.58374	NTAN1	2.009240611
3907	2.563525	1.2760808	HR	2.008904899
3908	1.448438	0.721633	PTK2B	2.007167091
3909	52.281869	26.049531	RPS6KA4	2.007017649
3910	124.96929	62.302797	NDUFA12	2.005837622
3911	25.691863	12.813179	ILVBL	2.005112252

3912	48.86609	24.378201	FASTK	2.004499406
3913	20.342952	10.150536	C6orf89	2.004125929
3914	21.237548	10.597909	ANKRD54	2.00393754
3915	327.98211	163.71555	CD81	2.003365639
3916	3.3404263	1.6674072	ACADS	2.003365639
3917	14.320057	7.1539168	RNF14	2.001708596
3918	35.597943	17.785095	KCTD10	2.00156048
3919	58.863064	29.41119	IFNGR2	2.001383286
3920	39.822986	19.90596	ASS1	2.00055587
3921	18.964781	9.4797556	ETHE1	2.000555869
3922	32.186647	16.088852	LDB1	2.000555869
3923	32.212142	16.102778	CNIH4	2.000408953
3924	11.574644	5.7863213	SLC25A25	2.000345794
3925	58.020315	29.010675	MRPL33	1.999964339
3926	8.8402777	4.4205481	LRP12	1.999814831
3927	3.9167761	1.9593115	MYLIP	1.999057326
3928	2.3040738	1.1525801	ZNF226	1.999057325
3929	2.6195909	1.3125182	APBA1	1.995851139
3930	59.763898	29.947674	MRPL53	1.995610675
3931	120.82109	60.545952	PSMA4	1.995527063
3932	20.708781	10.379157	MARK4	1.995227713
3933	14.93677	7.4873418	ALDH7A1	1.99493633
3934	57.562672	28.86094	FBLN1	1.994483571
3935	26.296658	13.185922	KDM5C	1.994298031
3936	2.4614114	1.2343263	SLC25A45	1.994133539
3937	48.94089	24.544347	CD59	1.993978051
3938	20.656551	10.362117	CHST12	1.993468163
3939	39.146341	19.651222	ABCC1	1.992056316
3940	15.870624	7.9679756	HS1BP3	1.991801219
3941	17.447853	8.7670485	TMEM18	1.990162743
3942	57.703069	28.99627	C11orf24	1.990016931
3943	86.234276	43.334831	BAX	1.989952966
3944	11.752547	5.9068197	MRPS14	1.989657369
3945	83.378728	41.920488	SPHK1	1.988973239
3946	25.425327	12.792176	GLA	1.98756849
3947	4.5863551	2.3079374	CDC14B	1.987209464
3948	15.63707	7.8708146	BACE1	1.986715607
3949	4.8744516	2.4539629	LOXL3	1.986359139
3950	5.2842979	2.6617518	HAPLN3	1.985270724
3951	3.1162542	1.5696873	NR2F1	1.985270724
3952	3.9413617	1.9853019	DNAJB9	1.985270723
3953	21.624466	10.894478	GSTM3	1.98490144
3954	10.978357	5.5375317	TEF	1.982536191

3955	18.234253	9.198376	ZNF512	1.982333932
3956	8.4535952	4.2655629	TPGS1	1.981824073
3957	21.688636	10.943775	PRADC1	1.981824072
3958	1.2829734	0.64737	ZNF25	1.981824072
3959	156.37149	78.951157	FIBP	1.980610463
3960	24.94143	12.59674	ATL3	1.979990748
3961	19.487029	9.8433136	CCDC130	1.979722456
3962	4.6589144	2.3537453	CCDC57	1.97936218
3963	115.1228	58.168784	NCLN	1.979116445
3964	70.22298	35.493491	USP5	1.978474854
3965	24.032122	12.148089	FOSL2	1.978263483
3966	709.95136	358.95632	FLNA	1.977821073
3967	32.53584	16.452885	IGFBP6	1.97751576
3968	56.233214	28.436917	GPAA1	1.977472242
3969	19.577149	9.9003424	SLC15A4	1.977421417
3970	14.309989	7.2379061	SNX4	1.977089663
3971	6.2208806	3.1470158	UBL3	1.976755469
3972	15.390603	7.7884527	AP1S2	1.976079656
3973	93.087103	47.133858	HDAC1	1.974951917
3974	2.37117	1.2012062	ADAMTS7	1.973990776
3975	3.0561379	1.5482027	RWDD2A	1.973990776
3976	1.6137129	0.8174876	PGBD2	1.973990776
3977	18.593664	9.4242762	RPS6KA2	1.972954016
3978	16.523467	8.3772944	MEX3D	1.972410951
3979	8.1437345	4.1316663	ABCB8	1.97105329
3980	25.768099	13.083477	C11orf31	1.969514607
3981	21.338779	10.839748	PI4KA	1.968567725
3982	8.8044591	4.4753206	PKD2	1.96733594
3983	30.507968	15.514336	MOGS	1.96643724
3984	45.242554	23.020972	ADH5	1.965275585
3985	43.051321	21.911833	SUPT6H	1.96475213
3986	7.6036303	3.8703379	TMEM99	1.96459082
3987	1.4785625	0.7526059	GGACT	1.96459082
3988	25.213216	12.839504	TMEM138	1.963721917
3989	7.9062654	4.0274548	MAP3K10	1.963092276
3990	12.945201	6.5948289	TBL1X	1.962932004
3991	5.6078411	2.8583149	DOK4	1.961939551
3992	41.106323	20.956045	SPC24	1.961549658
3993	26.042242	13.277746	RNF185	1.961345096
3994	11.193333	5.707898	LACTB	1.96102532
3995	14.374182	7.3299317	LIF	1.96102532
3996	304.92925	155.59349	NDUFA13	1.959781541
3997	68.414615	34.9138	PCOLCE	1.959529306

3998	53.923318	27.522718	SLC4A2	1.959229363
3999	81.307799	41.504559	CSNK1D	1.959008841
4000	58.476714	29.857193	PTK7	1.95854693
4001	32.456339	16.575449	NCK2	1.958097131
4002	17.351036	8.8652105	TBC1D20	1.95720514
4003	8.4567913	4.3230553	PHYH	1.956207076
4004	3.5480933	1.814325	STK38L	1.955599558
4005	7.9242817	4.0520983	RTN2	1.955599558
4006	20.384623	10.433065	C4orf3	1.953848013
4007	60.367613	30.904849	LRRC20	1.953337895
4008	14.162451	7.2512602	DDR2	1.953101985
4009	22.249057	11.391651	LEPROT	1.953101985
4010	14.603427	7.477043	MOSPD3	1.953101984
4011	17.510627	8.9688235	LRP11	1.952388393
4012	45.715473	23.41736	PACSIN2	1.95220442
4013	88.792693	45.48342	PACS1	1.95219913
4014	73.908163	37.860259	OTUB1	1.952130413
4015	31.153869	15.962282	SERINC1	1.951717788
4016	29.914003	15.330351	NCS1	1.95129272
4017	28.747136	14.734394	TEAD3	1.951022558
4018	109.06795	55.912329	PYGB	1.950695945
4019	106.12938	54.43036	WBP2	1.949819461
4020	1.9480304	0.999665	YPEL2	1.948683203
4021	2.1412404	1.098814	PCSK5	1.948683202
4022	13.436284	6.8950579	RABEP2	1.948683202
4023	14.67978	7.5373939	OSBPL5	1.94759364
4024	19.931565	10.235185	PCDHGC3	1.947357567
4025	19.89123	10.216178	HGSNAT	1.947032413
4026	6.6884975	3.4354747	AHDC1	1.946891804
4027	29.25587	15.032871	CALCOCO2	1.94612662
4028	15.131076	7.7763112	ARFGAP3	1.945790908
4029	93.048549	47.823415	ARL6IP1	1.945669282
4030	706.84892	363.52478	OAZ1	1.944431166
4031	295.32191	151.93806	PSMD8	1.943699357
4032	6.1878359	3.1843572	SLC25A30	1.943197817
4033	30.433398	15.663622	RAB11FIP5	1.942934874
4034	35.174507	18.106386	WBP5	1.94265759
4035	5.7412385	2.9565158	TMEM106A	1.941893365
4036	11.90653	6.1322874	CACNB3	1.94161315
4037	11.575115	5.9643114	CAPN5	1.940729393
4038	612.23833	315.60789	TAGLN2	1.939870205
4039	39.249698	20.238724	DAP3	1.939336561
4040	0.6792683	0.3503657	PCDHGB2	1.938740943

4041	1.3746669	0.7090514	SSR4P1	1.938740942
4042	2.6909548	1.3879909	ERMAP	1.938740941
4043	4.3720175	2.2550808	CLDN15	1.938740941
4044	2.2800821	1.1760633	PEX11A	1.938740941
4045	2.7325109	1.4094255	RPSAP52	1.93874094
4046	19.718574	10.174876	DMWD	1.937966993
4047	10.472127	5.4068892	TSHZ3	1.936811846
4048	46.178511	23.850265	PPARD	1.936184359
4049	15.547877	8.0320273	APBA3	1.935735142
4050	3.0210375	1.5616054	TIGD6	1.934571606
4051	5.9727917	3.0897137	ZFYVE1	1.933121402
4052	42.597889	22.039428	GLB1	1.932803689
4053	34.222264	17.710566	C7orf73	1.932307713
4054	8.0485324	4.1699555	SPRED3	1.930124315
4055	42.756503	22.162826	MNF1	1.929199039
4056	17.953093	9.3064823	TRAF4	1.929095464
4057	72.909446	37.802463	NDUFA4	1.928695651
4058	54.023437	28.023542	PHPT1	1.927787603
4059	23.113741	11.994732	ACP2	1.926990996
4060	10.561656	5.4809056	2-Mar	1.926990996
4061	4.3596881	2.2631892	SIPA1L2	1.926347164
4062	4.990498	2.5919679	ZNF653	1.925370314
4063	369.3085	191.82168	CAPNS1	1.925269877
4064	33.802064	17.56114	CNP	1.924821775
4065	44.302887	23.047298	KEAP1	1.922259474
4066	112.66895	58.623735	ADIPOR1	1.921899967
4067	0.2997559	0.1561003	SPRY3	1.920276743
4068	0.9613888	0.5006511	ZNF44	1.920276743
4069	2.0920413	1.0894478	WASH3P	1.920276742
4070	3.0270318	1.5763519	MTERFD3	1.920276742
4071	9.2463942	4.8151363	KLHL22	1.920276742
4072	8.2096773	4.2752574	FAM193B	1.920276741
4073	0.5989815	0.3119246	KCNJ3	1.920276741
4074	18.109582	9.4307148	MRPS33	1.920276741
4075	17.329596	9.0245305	FBXL12	1.920276741
4076	3.8869347	2.0241534	C1orf53	1.920276741
4077	11.241467	5.8540868	PTPMT1	1.920276741
4078	0.3265662	0.1700621	CD84	1.920276738
4079	39.359944	20.519143	XRCC1	1.91820599
4080	5.2171203	2.7198908	DNAJC18	1.918135964
4081	11.01376	5.7426317	TAF6L	1.917894264
4082	10.362406	5.4085452	DNAJC30	1.915932225
4083	3.0370082	1.5860658	ZNF438	1.914805867

4084	224.58393	117.29582	PSMB7	1.914679686
4085	69.275433	36.191499	DPF2	1.914135476
4086	46.626526	24.362927	RALB	1.913831057
4087	44.400299	23.242413	LEMD2	1.910313654
4088	29.667669	15.530679	RELB	1.91026226
4089	17.175783	8.9976717	COMMD10	1.908914157
4090	21.58498	11.313077	PIGQ	1.907967275
4091	16.076962	8.4281003	TOR1B	1.90754281
4092	5.1433715	2.6968936	TBC1D2	1.907146644
4093	25.003907	13.114749	CDH13	1.906548712
4094	6.4737452	3.3968738	TRIM7	1.905795017
4095	5.0758403	2.6648637	FLRT2	1.904727942
4096	3.3659356	1.7671477	APBB3	1.904727942
4097	19.022053	9.9882119	IGHMBP2	1.904450285
4098	9.4162159	4.946243	NBPF3	1.903710731
4099	7.5935041	3.9892511	ARID5A	1.903491106
4100	15.237039	8.0062185	FKRP	1.903150527
4101	2.6755169	1.4062786	BBS10	1.902551111
4102	116.60853	61.319206	SNF8	1.901664037
4103	15.195103	7.9923035	TUFT1	1.901216923
4104	6.1341449	3.2264308	BIVM	1.901216923
4105	4.575772	2.4079013	STRA6	1.900315445
4106	24.610877	12.951778	GPR107	1.900192875
4107	19.173557	10.096809	VEGFC	1.898971896
4108	25.864132	13.6282	BNIP3L	1.897839234
4109	13.411435	7.0711454	KIAA0141	1.896642567
4110	51.535107	27.173456	GRK6	1.896523801
4111	0.4540294	0.2395102	ATCAY	1.895657812
4112	1.0309847	0.5438665	COL8A2	1.89565781
4113	1.3106887	0.6914163	LINC00665	1.895657809
4114	2.6109277	1.3773201	CATSPER1	1.895657809
4115	2.8162095	1.4856107	HOXA11-AS	1.895657809
4116	1.167207	0.6157266	KRBA2	1.895657809
4117	1.1828661	0.6239871	LRRC46	1.895657808
4118	11.870858	6.262131	RHBDD3	1.895657808
4119	1.051075	0.5544645	PRKRIP1	1.895657808
4120	169.43458	89.468767	PSME2	1.893784629
4121	9.7780997	5.1634307	RAB27A	1.893721488
4122	32.005592	16.904919	GOLGA2	1.893270931
4123	3.5514571	1.8776328	ANKRD50	1.891454577
4124	25.421264	13.442419	IKBKG	1.891122742
4125	46.783561	24.758429	SCAMP4	1.889601394
4126	83.495565	44.204848	COX6C	1.888832758

4127	21.00667	11.125441	TM2D1	1.888165091
4128	3.1165316	1.650561	FAM46A	1.88816509
4129	6.1070714	3.2367783	TUBGCP6	1.886774689
4130	252.81773	134.10861	CALM2	1.885171444
4131	15.930139	8.450523	TAX1BP1	1.885106837
4132	1.2531539	0.6665744	WDPCP	1.879991216
4133	2.2726227	1.2088475	NFKBID	1.879991216
4134	9.1170033	4.8494925	GHDC	1.879991216
4135	0.3186537	0.1694974	KCNN3	1.879991215
4136	76.5361	40.725745	PSENEN	1.879305087
4137	13.806707	7.3510064	LZTR1	1.878206413
4138	4.2342655	2.2552468	HINT3	1.877517543
4139	50.534923	26.9277	ENG	1.876689123
4140	56.196353	29.95172	CDC25B	1.876231233
4141	50.85233	27.118182	BCL7C	1.875211577
4142	72.9282	38.900458	PRKACA	1.874738832
4143	38.683495	20.63932	STAT1	1.874262123
4144	1.2717983	0.6786123	ARHGAP42	1.874116244
4145	86.89866	46.394033	TMEM179B	1.873056822
4146	49.260465	26.311062	TRAPPC3	1.87223397
4147	45.233399	24.16173	BIN1	1.872109265
4148	43.69298	23.340612	VEGFB	1.871972295
4149	30.379154	16.242215	DNAJC15	1.870382371
4150	14.885679	7.9597151	SMARCA1	1.870127064
4151	21.074107	11.268994	PLEKHO2	1.870096525
4152	2.3609166	1.2625282	GTF2H5	1.869991262
4153	73.281134	39.222221	SPAG7	1.868357606
4154	110.45494	59.151036	CORO1B	1.867337429
4155	8.8991356	4.766707	ZNF618	1.866935721
4156	201.76702	108.09987	SURF4	1.866487219
4157	72.701759	38.963556	NDUFB11	1.865891281
4158	34.594317	18.542402	CLDND1	1.865686934
4159	16.01267	8.5832679	PRMT2	1.865568002
4160	12.826114	6.8753202	PC	1.865529745
4161	85.695141	45.936831	SSBP3	1.865499617
4162	12.425865	6.6610362	LDLRAP1	1.865455201
4163	181.77972	97.467279	LAMP1	1.865033289
4164	160.27746	85.94871	UBL5	1.864803513
4165	42.33585	22.727241	FAM129A	1.862780029
4166	33.459844	17.963493	GTF2A2	1.862657963
4167	8.2971306	4.4549857	TMEM120B	1.86243708
4168	0.3709643	0.1993155	ZNF829	1.861191306
4169	1.1908543	0.6398344	QPRT	1.861191305

4170	0.7520431	0.4040655	PTGER2	1.861191305
4171	18.194079	9.7755019	PSKH1	1.861191304
4172	1.3883016	0.745921	C8orf76	1.861191304
4173	1.3872748	0.7453693	ICAM2	1.861191303
4174	1.5424305	0.8287329	TTLL7	1.861191303
4175	4.117663	2.2123803	LRRC23	1.861191303
4176	10.470557	5.6257287	WWP1	1.861191303
4177	17.260081	9.2736739	SEL1L3	1.861191303
4178	1.9743111	1.0607781	C2orf76	1.861191303
4179	3.2449749	1.7434935	C16orf95	1.861191303
4180	31.301941	16.822744	TAOK2	1.860691789
4181	19.216058	10.339143	FAM3A	1.858573594
4182	5.2464848	2.8237877	NBPF1	1.857960068
4183	18.930444	10.188833	GSTM1	1.857960068
4184	10.584737	5.6978069	MVK	1.857686235
4185	2.9993734	1.615614	CCNG2	1.856491326
4186	7.5116051	4.0461299	SLITRK4	1.856491325
4187	6.4466234	3.4770301	DND1	1.854060302
4188	9.0477352	4.8855665	PLA2G15	1.851931645
4189	4.2916917	2.3194483	FICD	1.850307144
4190	34.73325	18.775928	TMED1	1.849881981
4191	16.95551	9.1717936	TSPAN9	1.848658028
4192	7.194965	3.8931312	MTMR3	1.848117801
4193	5.9260521	3.2067532	CRLF3	1.847991365
4194	63.701062	34.47529	ZC3H7B	1.847731011
4195	63.724207	34.500509	NOSIP	1.847051198
4196	13.424108	7.2686632	SLC31A1	1.846846861
4197	4.4797829	2.4261994	ATG4A	1.846419944
4198	5.1441425	2.7860089	VPS8	1.846419944
4199	3.9923276	2.1621991	CHAC2	1.846419944
4200	76.785601	41.591233	UBALD2	1.846196811
4201	18.488845	10.015067	NNT	1.846102963
4202	244.15432	132.29047	DUSP1	1.845592571
4203	28.153178	15.26233	PGRMC2	1.844618558
4204	17.987534	9.7534892	VPS16	1.844215263
4205	6.6523707	3.6076584	SIX5	1.84395805
4206	53.133918	28.821855	HDAC3	1.843528752
4207	4.838267	2.6249153	SYT11	1.843208779
4208	6.7794601	3.678075	NMNAT1	1.843208779
4209	14.824117	8.0435762	ERLIN2	1.842975845
4210	24.757493	13.44393	FYN	1.841536849
4211	44.110602	23.954572	IQGAP1	1.841427293
4212	7.0309157	3.8200881	RTKN	1.8405114

4213	19.838574	10.780891	UCK1	1.840160893
4214	32.478601	17.650945	SEZ6L2	1.84004891
4215	63.865194	34.721265	NFIC	1.83936829
4216	125.75077	68.399309	LOXL2	1.838480195
4217	0.8329733	0.4531428	ZNF708	1.838213633
4218	4.5120325	2.4545746	EXO5	1.838213633
4219	1.3076047	0.7113453	HSPA1L	1.838213633
4220	24.63987	13.422726	KLF13	1.835683053
4221	7.7512055	4.2226362	DAK	1.835631872
4222	14.989551	8.1679348	NAGA	1.835170233
4223	2.6104322	1.4241377	MEGF9	1.832991435
4224	7.4636876	4.0728957	TSHZ1	1.832526091
4225	5.5946175	3.0544164	WDR91	1.831648584
4226	2.3850058	1.3021088	KALRN	1.831648583
4227	41.712702	22.787807	RAB2A	1.830483327
4228	101.53577	55.50295	FAM50A	1.829376068
4229	1.6545352	0.904426	LPP-AS2	1.829376068
4230	1.3714416	0.7496773	RAP1GAP	1.829376067
4231	1.5850491	0.8664425	LOC100130581	1.829376066
4232	7.2879092	3.9856464	C19orf12	1.828538824
4233	59.666762	32.631556	SLC25A1	1.828498809
4234	22.089077	12.080585	P4HA1	1.828477332
4235	36.145846	19.769047	DERL1	1.82840609
4236	104.06026	56.929847	FKBP2	1.827868236
4237	182.12416	99.652829	PSMC3	1.827586461
4238	42.295522	23.153746	ATXN7L3	1.826724797
4239	9.0901236	4.9770643	MAP3K6	1.826402681
4240	2.6407876	1.4462212	ATP10D	1.825991468
4241	8.0444252	4.4057697	AFF1	1.825884151
4242	92.038738	50.429268	PIN1	1.825105566
4243	7.8871446	4.3250611	C7orf43	1.823591479
4244	58.418984	32.052904	PPP2CB	1.822580092
4245	56.820035	31.179624	CDCA3	1.822345076
4246	102.31093	56.171568	PFDN1	1.821400704
4247	12.966592	7.12033	TBC1D22A	1.821066118
4248	75.221913	41.422807	VDAC3	1.815954015
4249	14.182197	7.8104553	LOC375295	1.815796393
4250	2.9901127	1.6472309	IKZF4	1.815235963
4251	26.594915	14.652276	SPAG5	1.815070655
4252	50.891875	28.038818	SERPINE2	1.815050659
4253	14.630496	8.0619139	LOC284412	1.81476703
4254	13.487233	7.4335671	NUDT16L1	1.814368881
4255	29.2438	16.119967	NEU1	1.814135236

4256	5.3054863	2.9247014	ASH1L	1.814026611
4257	10.761943	5.9335997	HOXC6	1.81372923
4258	22.604889	12.465417	SSH1	1.813408193
4259	365.95165	201.85713	RAB1B	1.812924114
4260	37.866576	20.889796	TMEM101	1.812682888
4261	144.17514	79.542202	ITGA5	1.812561602
4262	306.78227	169.26106	NDUFS8	1.81247997
4263	4.842422	2.6742595	HECTD4	1.810752514
4264	0.3074387	0.1699033	ITGAM	1.809491553
4265	0.4355917	0.240726	GPR82	1.80949155
4266	0.3965198	0.2191333	ADAMTS8	1.809491546
4267	1.460988	0.8074025	ZGLP1	1.809491545
4268	18.980624	10.489479	C19orf47	1.809491545
4269	2.0929649	1.1566591	LINC00704	1.809491545
4270	1.3671945	0.7555683	NHLRC1	1.809491545
4271	0.8900528	0.49188	TSPAN1	1.809491545
4272	5.3047146	2.9316051	KATNBL1	1.809491545
4273	1.1550249	0.6383146	PROCA1	1.809491544
4274	3.0031837	1.6596838	ZNF34	1.809491544
4275	2.4851729	1.3734095	LOC730183	1.809491544
4276	0.7508926	0.4149743	LOC440300	1.809491544
4277	0.4683135	0.2588094	ZNF599	1.809491543
4278	0.6194465	0.3423318	ZNF613	1.809491543
4279	0.5398951	0.2983684	ZNF567	1.809491542
4280	0.2500937	0.1382121	PRELP	1.809491541
4281	16.546939	9.1572246	DOLK	1.806981848
4282	40.958616	22.671019	LOC541471	1.806650899
4283	39.613128	21.931515	TMEM115	1.806219408
4284	66.727441	36.958418	DAPK3	1.805473429
4285	18.209665	10.090821	SALL1	1.804577118
4286	27.442089	15.20872	TMCO1	1.804365506
4287	14.242917	7.8959265	PCSK7	1.803830987
4288	5.7632605	3.1956333	CRELD1	1.803479945
4289	52.36797	29.039484	OSTC	1.803336812
4290	4.9580224	2.7512382	FBXO8	1.802105865
4291	5.1212529	2.8442275	LRIG3	1.800577793
4292	8.1654564	4.535223	TRAF3IP2	1.800453125
4293	39.174963	21.766803	CHST14	1.799757281
4294	8.4881819	4.7178803	ZCCHC10	1.799151593
4295	2.5952615	1.4432131	SESN1	1.798252467
4296	69.773627	38.802821	RTN3	1.798158612
4297	17.114935	9.5202418	BIN3	1.7977416
4298	186.18207	103.56723	PSMB1	1.797692846

4299	23.320015	12.977104	TINAGL1	1.797012292
4300	8.6887979	4.8381669	MPND	1.795886345
4301	8.9264388	4.9704921	KIAA0247	1.795886345
4302	82.968847	46.212117	HEXB	1.795391611
4303	35.930948	20.015786	COMMD1	1.795130501
4304	14.524814	8.0924514	GGT7	1.794859538
4305	6.6752392	3.724485	ANKRD13C	1.792258292
4306	1.2636164	0.7050414	HAS3	1.792258291
4307	13.265777	7.4053386	RAB11FIP3	1.791380165
4308	15.291037	8.537579	TBC1D7	1.791027346
4309	3.4061613	1.9022702	MMP24	1.790577
4310	3.6083022	2.016254	RELL2	1.789607022
4311	9.6744231	5.4078	CSRNP2	1.788975767
4312	6.1690879	3.4492462	KRT10	1.788532183
4313	3.2274817	1.8045421	GPR161	1.788532183
4314	6.0879313	3.4087121	ZNF500	1.785991655
4315	19.185986	10.749219	CRELD2	1.784872611
4316	15.159545	8.5000366	SYAP1	1.783468176
4317	20.873258	11.704164	CD99L2	1.783404511
4318	19.197066	10.767067	YIPF5	1.78294302
4319	5.8473485	3.2799594	MSL3	1.782750291
4320	120.574	67.662492	TMEM258	1.781991673
4321	7.2366145	4.0614176	LATS2	1.781795246
4322	56.141328	31.518387	PSMD11	1.781224641
4323	28.227022	15.848349	SLC39A13	1.781070212
4324	42.476331	23.849115	TRAM2	1.781044309
4325	5.6919687	3.1963535	ZNF524	1.780769457
4326	1.7950499	1.0080192	WDR27	1.780769457
4327	2.3956926	1.3453132	FAM120C	1.780769456
4328	0.7749981	0.4352041	ERVK13-1	1.780769454
4329	15.572093	8.7511519	MAP4K2	1.779433546
4330	11.477255	6.4548398	AACS	1.778085149
4331	36.098685	20.303346	NDEL1	1.777967302
4332	86.271618	48.525088	USF2	1.777876585
4333	18.304593	10.299135	FOXJ2	1.777294186
4334	43.834029	24.708991	SELK	1.774011318
4335	0.8750154	0.4936441	NEK8	1.772563147
4336	0.7834568	0.4419909	ZNF461	1.772563146
4337	12.961959	7.3125515	SSH3	1.772563146
4338	4.3548872	2.4568305	MANSC1	1.772563146
4339	4.0996623	2.3128441	SLC18B1	1.772563146
4340	48.132437	27.154145	FBXW5	1.772563146
4341	3.1259925	1.7635437	C1orf192	1.772563146

4342	2.3659357	1.334754	CXCR7	1.772563146
4343	16.646066	9.390958	MKL1	1.772563146
4344	1.8490159	1.0431312	FGF13	1.772563146
4345	2.1544639	1.2154512	ZSCAN22	1.772563145
4346	1.7742419	1.0009471	LOC100131089	1.772563145
4347	0.512773	0.2892834	ZNF594	1.772563145
4348	0.8579053	0.4839914	CD99P1	1.772563145
4349	0.39532	0.2230216	AKD1	1.772563144
4350	39.745569	22.428343	DAG1	1.772113485
4351	57.174538	32.282556	G6PC3	1.771066049
4352	4.1017928	2.3173191	FLJ45340	1.770059526
4353	597.38564	337.71647	TPM4	1.768896969
4354	19.42058	10.981634	CYTH2	1.768459991
4355	2.1906334	1.2391875	TMEM200A	1.76779819
4356	63.831186	36.119463	STRN4	1.7672241
4357	1.5168435	0.8587162	PLA2R1	1.766408413
4358	8.2586021	4.6771189	DGKA	1.765745595
4359	10.59402	6.0010615	RAB9A	1.765357605
4360	4.1278905	2.3382744	MEX3B	1.765357605
4361	9.2445913	5.2366678	CHAC1	1.765357605
4362	7.3143668	4.1457627	NCOA1	1.764299448
4363	8.5460269	4.8461768	PHF8	1.763457513
4364	8.2354129	4.6715754	CDC42BPA	1.762877008
4365	92.488272	52.468698	APMAP	1.762732378
4366	51.244759	29.110462	ZFAND3	1.760355411
4367	7.0618788	4.0118529	ZNF319	1.760253679
4368	122.61752	69.749416	MYO1C	1.757972029
4369	2.4399391	1.3880706	LOC100507373	1.757791786
4370	8.121012	4.620008	C7orf10	1.757791786
4371	87.218149	49.622632	TADA3	1.757628438
4372	46.764521	26.617399	NSFL1C	1.756915519
4373	48.179952	27.442646	GTF3C1	1.755659837
4374	6.3240687	3.6023082	USP47	1.755560142
4375	290.12479	165.32193	TRMT112	1.754908135
4376	234.20401	133.46102	PFDN5	1.754849523
4377	754.29326	429.90255	ANXA5	1.754568014
4378	45.932132	26.187641	PRKCDBP	1.753962175
4379	32.695597	18.648074	GALNT10	1.753296155
4380	17.98987	10.263106	TPST1	1.752868
4381	9.2457631	5.2756535	RNF135	1.752534184
4382	22.222281	12.69193	POLR3GL	1.750898486
4383	2.0916008	1.1953111	FAM45A	1.749837978
4384	5.4776452	3.1303728	COQ10A	1.749837978

4385	1.9977294	1.1416654	GK	1.749837977
4386	2.271947	1.2983756	HOXA7	1.749837977
4387	19.187443	10.965268	SLC44A1	1.749837977
4388	20.433003	11.682732	VAV2	1.748991828
4389	22.419029	12.82063	ECI1	1.7486683
4390	4.5982238	2.6295575	FBXL17	1.7486683
4391	11.266909	6.4489381	SMUG1	1.747095285
4392	1.9488571	1.1159903	ILDR2	1.746302951
4393	29.960669	17.162493	TRIM26	1.745706129
4394	0.8350826	0.4785939	SHISA9	1.744866846
4395	44.648202	25.597175	ADI1	1.744262878
4396	8.7837163	5.0368168	ZBTB22	1.743902299
4397	394.71992	226.38726	COTL1	1.743560669
4398	14.396018	8.2571935	CCDC97	1.743451707
4399	5.3377775	3.0623723	LRRC37BP1	1.743020427
4400	56.112626	32.193617	SPTAN1	1.742973645
4401	23.513311	13.495846	SEC24D	1.742262922
4402	30.784394	17.669727	C6orf62	1.742211037
4403	1.2712131	0.7299674	WHAMMP2	1.741465547
4404	2.6653813	1.5305392	KRBOX4	1.741465547
4405	19.772249	11.364042	TXNDC15	1.739895717
4406	10.419975	5.9893936	CCDC34	1.739737903
4407	36.055277	20.731723	MEN1	1.739135605
4408	1.6057438	0.9233762	LOC100132891	1.738991874
4409	9.7957369	5.634671	FAM118A	1.738475393
4410	115.73681	66.592259	YIF1A	1.737991879
4411	2.6080986	1.5013993	LOC388152	1.737111884
4412	4.9283666	2.8371037	RAB40B	1.737111883
4413	88.953643	51.240978	ACTR1A	1.735986446
4414	1.5393331	0.8868992	CTNND1	1.735634748
4415	13.491428	7.7731952	AZI1	1.735634747
4416	72.119845	41.552432	SDCBP	1.735634747
4417	8.9115518	5.1395096	LRFN3	1.733930359
4418	27.68785	15.97837	CPSF4	1.732833283
4419	13.115092	7.5704957	NCAM1	1.732395413
4420	75.746682	43.755467	LRP10	1.731136387
4421	18.779031	10.8508	AKR7A2	1.730658579
4422	19.997932	11.558299	ADCY7	1.730179415
4423	7.2279031	4.1824615	CLEC16A	1.728145771
4424	46.175983	26.735939	ACO2	1.727112809
4425	13.640883	7.9003947	SOGA1	1.726607805
4426	48.225984	27.933288	ETS2	1.726470035
4427	108.20659	62.679305	PSMB2	1.726352745

4428	19.827901	11.485631	TPST2	1.726322368
4429	46.826608	27.131441	STX8	1.725916748
4430	16.996579	9.8478559	TLE2	1.725916748
4431	18.7398	10.858586	WDR55	1.725804887
4432	60.421657	35.015315	LITAF	1.725577994
4433	55.982535	32.44637	SH3BP4	1.725386675
4434	0.3001145	0.1741485	SPN	1.723325288
4435	0.3896775	0.2261195	PDE4C	1.723325285
4436	0.2663593	0.1545612	NUGGC	1.723325284
4437	0.5396155	0.3131246	C5orf27	1.723325284
4438	0.5950871	0.3453133	NSUN5P1	1.723325283
4439	0.7960256	0.4619126	LOC100131655	1.723325283
4440	0.9886124	0.5736656	ZNF684	1.723325282
4441	0.7040524	0.4085429	HSPB6	1.723325281
4442	1.918081	1.1130116	ZSCAN30	1.723325281
4443	1.0956861	0.6357976	MFI2-AS1	1.723325281
4444	0.6928175	0.4020236	LOC284648	1.723325281
4445	0.2980542	0.172953	TUBB1	1.723325281
4446	0.9675	0.5614146	FAM153C	1.723325281
4447	1.182744	0.686315	FSCN2	1.723325281
4448	10.505933	6.0963144	NUDT2	1.723325281
4449	2.5169022	1.4604916	LOC285033	1.723325281
4450	5.0338044	2.9209833	SNORA73A	1.723325281
4451	1.0276109	0.5962954	RSPH9	1.723325281
4452	20.362877	11.816038	BCKDHA	1.723325281
4453	0.821117	0.4764725	GNG10	1.723325281
4454	1.0461822	0.6070718	IL22RA1	1.723325281
4455	19.680677	11.420176	UNK	1.72332528
4456	2.4662663	1.431109	FLJ42709	1.72332528
4457	1.9362842	1.1235744	ZNF333	1.72332528
4458	1.4183269	0.8230175	TBC1D19	1.72332528
4459	1.7903737	1.0389064	ARRDC4	1.72332528
4460	2.6626853	1.5450857	TIRAP	1.72332528
4461	0.8269821	0.4798758	MAP3K14-AS1	1.72332528
4462	1.2113433	0.7029104	YPEL1	1.72332528
4463	0.5684656	0.3298655	PRPH	1.72332528
4464	0.620606	0.3601212	EBLN2	1.72332528
4465	1.5988563	0.927774	MFSD8	1.72332528
4466	0.9455513	0.5486784	NPIP	1.72332528
4467	0.918869	0.5331954	GP6	1.72332528
4468	0.4790793	0.277997	A1BG-AS1	1.723325279
4469	0.3616791	0.2098728	IGF2-AS	1.723325279
4470	0.734836	0.4264059	HSD17B7P2	1.723325278

4471	0.2221269	0.1288944	BAIAP3	1.723325278
4472	0.3232002	0.1875445	OR7D2	1.723325277
4473	0.3476802	0.2017496	FAXDC2	1.723325276
4474	0.1460403	0.0847908	SLC22A20	1.722359143
4475	276.81688	160.74546	PFKP	1.722082124
4476	43.699599	25.382311	NOTCH2	1.721655623
4477	95.467927	55.453807	RNF181	1.721575712
4478	44.822216	26.048612	PAK4	1.720714182
4479	23.30358	13.551911	CIC	1.719578921
4480	19.161733	11.14327	GMPPB	1.719578921
4481	35.034231	20.37601	ABHD17A	1.719386251
4482	9.9569424	5.7912178	SLC24A6	1.719317548
4483	10.836774	6.3029509	SLC26A6	1.719317548
4484	153.87722	89.514658	COPS6	1.719016968
4485	41.19525	23.976622	C1orf123	1.718142347
4486	104.76691	60.997004	PLEC	1.717574639
4487	4.8295922	2.8125291	TRAF5	1.717170548
4488	42.298458	24.632648	SEPHS2	1.717170547
4489	15.677602	9.1308044	LRIG1	1.717001151
4490	5.4929052	3.2001881	FAM149B1	1.71643198
4491	31.127633	18.137801	UBE2A	1.716174554
4492	5.613545	3.272977	ACOX1	1.71511897
4493	25.185365	14.687109	LAYN	1.714793968
4494	51.7101	30.156785	MUS81	1.714708654
4495	3.0082556	1.7556433	TERT	1.713477707
4496	10.22315	5.9663163	TRIM56	1.713477707
4497	12.941128	7.5551825	IL17RC	1.712880885
4498	26.447346	15.441302	FOXP4	1.712766614
4499	6.5048136	3.7983104	HOXA3	1.712554498
4500	12.537087	7.3206937	FAM173A	1.712554497
4501	11.695062	6.8334617	THRA	1.711440278
4502	3.9710901	2.3203206	APOBEC3D	1.711440278
4503	90.096979	52.648368	KIAA1191	1.711296702
4504	5.4508385	3.1874964	LOC100505687	1.710068933
4505	26.375624	15.429129	PPM1F	1.709469399
4506	13.459955	7.873955	HIC1	1.709427497
4507	3.399871	1.9890926	IL1RAP	1.709257319
4508	9.1691252	5.3643914	DHRS7B	1.709257319
4509	5.4426732	3.1847789	ZMAT3	1.708964237
4510	116.8141	68.36869	EZR	1.708590529
4511	16.142744	9.4557285	FLYWCH1	1.707192022
4512	3.9592306	2.320643	LOC400657	1.706092028
4513	3.4724481	2.0353229	TMLHE	1.706092028

4514	9.8639383	5.7827142	MCUR1	1.70576273
4515	17.433772	10.224982	UHMK1	1.705017438
4516	23.858579	13.994985	UBXN4	1.704794901
4517	88.098709	51.678935	EPN1	1.704731508
4518	9.5223742	5.5859703	GBX2	1.704694737
4519	78.8005	46.263998	HARS	1.703279095
4520	2.6730124	1.5695435	LINC00338	1.703050866
4521	2.3201535	1.3623513	WNT2B	1.703050866
4522	1.3051188	0.7663416	KIAA0895	1.703050865
4523	0.8440888	0.4956334	ZNF862	1.703050865
4524	8.9427271	5.254914	FARS2	1.701783715
4525	154.49716	90.812745	CUTA	1.701271758
4526	31.942395	18.780286	ECI2	1.700847124
4527	19.077689	11.218819	HNRNPUL2	1.700507733
4528	12.706589	7.4729358	TM2D2	1.700347611
4529	23.953966	14.092905	FLYWCH2	1.699718086
4530	20.385641	11.998237	ANAPC16	1.699053094
4531	5.406868	3.1829327	ABCB9	1.698706348
4532	1.657969	0.9760186	CACFD1	1.698706348
4533	11.75954	6.9226444	FAM127C	1.698706348
4534	1.9035697	1.1205996	RNF144B	1.698706348
4535	1.112625	0.6549837	ZNF468	1.698706348
4536	6.976051	4.1100561	HOXD8	1.697312824
4537	27.520322	16.215518	MARK2	1.697159609
4538	13.593983	8.0209047	MAN2C1	1.694819149
4539	35.070876	20.693552	STUB1	1.694773146
4540	26.337042	15.549246	GALK1	1.693782561
4541	5.0517868	2.9828464	SIDT2	1.693612776
4542	2.0782222	1.2282694	SH3YL1	1.691992093
4543	59.303694	35.059617	APOA1BP	1.691510045
4544	3.1561753	1.867007	PARP9	1.690500038
4545	8.1668776	4.8319447	STOML1	1.69018441
4546	7.9345429	4.695308	TECPR1	1.689887626
4547	54.857116	32.462838	VAMP2	1.689843533
4548	35.016209	20.729463	TPRA1	1.689200028
4549	26.500628	15.691441	TTC9C	1.688858775
4550	9.648125	5.7151878	SMARCD3	1.688155377
4551	30.073912	17.819779	MED16	1.687670275
4552	61.464114	36.458574	SCAF1	1.685861688
4553	2.8353673	1.6818505	PAPPA	1.685861687
4554	1.9000369	1.1275988	HECTD2	1.685029164
4555	3.0483969	1.8091063	AMIGO3	1.685029163
4556	8.8699324	5.265894	TMEM110	1.684411484

4557	5.4587402	3.2410712	DLG3	1.684239552
4558	36.569045	21.731375	COG4	1.682776451
4559	109.55095	65.122093	ARL6IP4	1.68223938
4560	155.3603	92.365533	DAP	1.682015928
4561	26.539808	15.784157	DHX8	1.681420715
4562	3.7316715	2.2209129	FKBP7	1.680242149
4563	11.677558	6.9499258	TMEM160	1.680242149
4564	2.5777293	1.5341416	FGGY	1.680242149
4565	6.7937424	4.0433115	S1PR2	1.680242149
4566	2.3274858	1.3852086	LOC286437	1.680242148
4567	40.541193	24.134193	LRP5	1.679823866
4568	19.778522	11.779863	STK38	1.679011202
4569	15.810182	9.4277491	ORAOV1	1.676983761
4570	94.502527	56.410917	PPP2R4	1.675252446
4571	88.890782	53.081999	POMP	1.674593718
4572	11.943049	7.1378825	SPSB3	1.673192182
4573	58.76063	35.12086	MFSD12	1.67309771
4574	23.930142	14.305159	ATP1B1	1.672832948
4575	4.9421315	2.955354	ZBTB47	1.672263791
4576	2740.9496	1639.8957	CFL1	1.671417083
4577	27.322	16.356199	HSPG2	1.670437022
4578	1.1390915	0.6819682	LOC100289019	1.670299888
4579	0.8481375	0.5077756	RBM47	1.670299888
4580	2.4781368	1.4836478	A1BG	1.670299888
4581	0.8409665	0.5034823	ZXDA	1.670299887
4582	2.7199438	1.6284164	VPS37D	1.670299887
4583	20.21427	12.10218	TSSC1	1.670299887
4584	17.257572	10.335787	GJC1	1.669691178
4585	102.5392	61.42793	NDUFA1	1.669260174
4586	30.865507	18.495918	TMEM30A	1.668773977
4587	11.88294	7.1214326	PHF2	1.668616542
4588	27.574707	16.532573	CAMTA2	1.667901754
4589	8.3829244	5.0265352	CYB561D2	1.667734143
4590	1.9351859	1.1616591	SGMS2	1.665881106
4591	315.46188	189.36638	PTTG1	1.665881105
4592	7.5497633	4.5319941	SLC25A14	1.665881105
4593	13.210023	7.9297514	SUV39H1	1.665881104
4594	61.415747	36.872578	ZNHIT1	1.665621176
4595	29.983169	18.003879	NUBP1	1.665372749
4596	29.439807	17.679636	AGPAT3	1.665181784
4597	96.169589	57.757828	SDF4	1.66504858
4598	9.3632055	5.6266356	VPS37A	1.664085974
4599	163.17207	98.074317	ERGIC1	1.663759473

4600	3.1731611	1.9076545	LOC389831	1.663383532
4601	68.392927	41.134083	GSK3A	1.662682654
4602	59.690717	35.916366	MBOAT7	1.66193644
4603	5.0664384	3.0488059	ZSCAN9	1.661777949
4604	516.29359	310.7591	ARHGDIA	1.661394921
4605	11.541831	6.9475301	GFPT1	1.661285571
4606	799.04472	481.0693	FAU	1.66097632
4607	0.1260354	0.075884	ECM2	1.660895774
4608	5.7929232	3.4883281	RUNX2	1.660658907
4609	84.061707	50.669808	DNAJB6	1.659009792
4610	23.61861	14.242715	MORN2	1.658294138
4611	72.644706	43.837023	TMEM259	1.657154193
4612	36.089203	21.788956	TM9SF4	1.656307075
4613	72.471706	43.763717	NDUFA6	1.655976936
4614	13.372938	8.0766948	FAM134C	1.655743897
4615	32.434269	19.589922	PCYT1A	1.655660975
4616	50.225069	30.346693	SNAI2	1.655042581
4617	0.3252431	0.1965937	ZNF527	1.654392273
4618	20.42342	12.344968	RAPGEF1	1.65439227
4619	3.3244187	2.0094501	ZNF784	1.65439227
4620	0.3867307	0.23376	CCDC40	1.65439227
4621	4.2512435	2.5696708	APOBEC3F	1.65439227
4622	1.8596721	1.1240817	KLC3	1.65439227
4623	53.611793	32.405732	FAM127B	1.65439227
4624	8.3673576	5.0576624	LINC00467	1.65439227
4625	1.0413467	0.6294436	SKAP1	1.654392269
4626	2.3674288	1.4309961	SPDYE7P	1.654392269
4627	6.7998354	4.1101712	HSF2	1.654392269
4628	13.940826	8.4265539	PARP6	1.654392269
4629	1.2046214	0.7281353	DLEU2	1.654392269
4630	0.7432885	0.4492819	TCP11L2	1.654392269
4631	19.221656	11.61856	SPRYD3	1.654392269
4632	0.6647512	0.4018099	FAM45B	1.654392269
4633	55.743486	33.712401	NDUFAF3	1.653500895
4634	43.432437	26.28351	PITHD1	1.652459569
4635	78.906199	47.753497	WDR83OS	1.652364828
4636	11.450962	6.9312324	REEP3	1.652081666
4637	38.451175	23.277357	POLE4	1.65187033
4638	3.9587859	2.3967852	SLC35D1	1.651706568
4639	10.064579	6.0943817	CHST3	1.651451999
4640	20.122215	12.188728	PJA2	1.650887202
4641	334.97579	202.97199	TMBIM6	1.650354705
4642	15.600856	9.4581113	CCDC28B	1.649468483

4643	5.1042831	3.0945018	CNTNAP1	1.649468483
4644	14.799965	8.9748753	ERLEC1	1.649044018
4645	16.321554	9.8990314	TRIM37	1.648803106
4646	21.743654	13.189333	MKNK2	1.648578642
4647	16.955581	10.286096	QTRT1	1.648398095
4648	16.235089	9.8490095	WBP1L	1.648398095
4649	8.8422417	5.3656665	YBEY	1.6479298
4650	114.10136	69.283949	MAP7D1	1.646865788
4651	4.5557593	2.7665439	TRMT11	1.646733046
4652	61.588856	37.400631	ASF1B	1.646733046
4653	16.224222	9.8523691	COQ2	1.646733046
4654	5.5213669	3.3529217	CIB2	1.646733046
4655	327.39101	198.91383	STIP1	1.645893679
4656	47.061524	28.593664	RCN3	1.645872459
4657	4.054465	2.4647319	RNF217	1.644992313
4658	35.09033	21.336407	NDFIP1	1.644622236
4659	4.4770509	2.7236196	DCUN1D3	1.643787191
4660	27.039709	16.456211	MUL1	1.643130936
4661	16.395934	9.9839596	ROBO1	1.642227621
4662	38.715492	23.577887	PGM1	1.64202555
4663	9.4478809	5.7570009	ZNF746	1.641111598
4664	41.703799	25.424614	RING1	1.640292335
4665	13.974311	8.5202443	CHMP4A	1.640130267
4666	4.5218759	2.7570224	RGS20	1.640130267
4667	13.829715	8.4335157	ZFYVE27	1.639851712
4668	14.01005	8.5453824	PTPRJ	1.639487834
4669	18.363264	11.202162	DNAJC1	1.639260633
4670	7.9799514	4.8680187	ARMCX3	1.639260633
4671	47.8547	29.195408	RNPEP	1.639117341
4672	10.245894	6.2532745	PTCHD3P1	1.638484651
4673	12.982264	7.9297514	TCEAL1	1.637159017
4674	11.567603	7.0656563	PRKAG2	1.637159017
4675	2.2188795	1.3553232	NHLRC3	1.637159016
4676	25.094631	15.333963	ZFAND6	1.636539116
4677	14.18682	8.6738119	MGST2	1.635592357
4678	791.03221	483.65214	CALR	1.63553956
4679	12.836201	7.8501426	IFT27	1.63515515
4680	2.9771357	1.8207053	CYP2U1	1.63515515
4681	10.528181	6.448626	FAM219B	1.632623951
4682	1.5752041	0.9648297	SERAC1	1.632623951
4683	4.253051	2.6050402	ZBED6	1.63262395
4684	11.383804	6.9727041	RRAGC	1.63262395
4685	15.391863	9.429139	BRD1	1.632372003

4686	8.6975241	5.3328263	PHF21A	1.630940833
4687	85.120923	52.200577	ATRAID	1.630650991
4688	229.76928	140.91269	ATP6V0C	1.630579048
4689	14.585831	8.945449	TTC31	1.630530843
4690	160.09663	98.245546	TRAPPC1	1.629556111
4691	38.702764	23.753853	C17orf89	1.62932572
4692	13.151425	8.0716978	HAGH	1.62932572
4693	47.914119	29.417343	TSPAN3	1.62877115
4694	343.85918	211.22613	ARF1	1.627919649
4695	141.43952	86.887208	THBS1	1.627852098
4696	58.135085	35.735434	COX17	1.626819065
4697	18.702519	11.498563	APOBEC3B	1.626509255
4698	43.586321	26.80118	HMGXB3	1.626283663
4699	53.40066	32.840911	DYNLL2	1.626040789
4700	30.50304	18.767836	NADK	1.625282728
4701	0.5833807	0.3590368	ZNF81	1.624849553
4702	0.7563162	0.4654685	DLG5-AS1	1.624849551
4703	2.112806	1.3003087	LOC100505695	1.624849551
4704	1.5578624	0.9587733	AKAP7	1.624849551
4705	1.9951214	1.2278807	RAB42	1.624849551
4706	1.2429394	0.7649566	PRX	1.624849551
4707	4.2326339	2.6049389	PGAP3	1.624849551
4708	3.6855217	2.2682234	WWOX	1.624849551
4709	11.54635	7.1061039	NFKBIE	1.62484955
4710	1.845233	1.1356332	RAPGEFL1	1.62484955
4711	3.2920936	2.0260913	ARRDC3	1.62484955
4712	1.9247645	1.1845801	PSCA	1.62484955
4713	18.421344	11.337261	MGRN1	1.62484955
4714	2.665575	1.6405057	C18orf56	1.62484955
4715	1.1678016	0.7187137	SP5	1.62484955
4716	11.094788	6.8281939	ZSWIM4	1.62484955
4717	0.7321605	0.450602	LOC729603	1.624849549
4718	120.18708	74.012872	AHNAK	1.623867214
4719	35.448438	21.832846	TIMM17B	1.623628777
4720	4.5679384	2.8142742	DYRK2	1.623131951
4721	8.8375086	5.4486829	MXD4	1.621953206
4722	5.7530562	3.5493909	TIGD2	1.620857291
4723	19.945827	12.305727	CASP2	1.620857291
4724	1.7477594	1.0785707	NAA38	1.620440188
4725	12.34532	7.6209168	MINOS1	1.619925764
4726	6.9614616	4.2973955	GALT	1.619925764
4727	71.449939	44.119261	PRKAR1A	1.619472716
4728	43.407451	26.806075	MIDN	1.619313932

4729	13.153733	8.1232212	CCDC104	1.619275452
4730	78.910395	48.731916	ARF5	1.619275452
4731	69.193547	42.764573	SHFM1	1.618010958
4732	164.78371	101.84539	FKBP8	1.61797915
4733	64.860165	40.09401	UBE2T	1.617702118
4734	6.6383131	4.1046298	NOL3	1.617274494
4735	210.85357	130.44124	ITM2C	1.616464063
4736	27.567583	17.060424	MAN1B1	1.615879089
4737	2.9434958	1.8219014	SLC16A13	1.615617451
4738	2.679116	1.6582613	RALGPS2	1.61561745
4739	21.764961	13.471605	CLN3	1.61561745
4740	2.499994	1.5473923	C15orf37	1.61561745
4741	63.879665	39.555167	TMEM66	1.614951217
4742	13.318405	8.2472452	INHBA	1.614891331
4743	8.2801587	5.1286729	TRAPPC9	1.614483684
4744	53.05345	32.864707	HSPB11	1.614298579
4745	0.0161412	0.01	CMYA5	1.6141236
4746	30.821882	19.096779	PNPLA6	1.613983263
4747	116.85541	72.413932	GPS1	1.613714486
4748	19.797232	12.268534	MAPRE2	1.613659126
4749	7.7999813	4.8356009	CMC4	1.613032463
4750	16.853459	10.448307	ATG4D	1.613032462
4751	16.378281	10.159322	GM2A	1.612143004
4752	27.612934	17.131567	DPY30	1.611815998
4753	15.478884	9.6040266	ENTPD4	1.611707771
4754	25.00794	15.525345	MAML1	1.610781589
4755	12.945061	8.0371841	CARD10	1.61064632
4756	292.01267	181.3318	EDF1	1.61037761
4757	3.8643616	2.4025571	TRAPPC2P1	1.608436929
4758	5.110997	3.1776173	MTMR9	1.608436929
4759	2.5173365	1.5650825	SUV420H2	1.608436929
4760	2.415226	1.5015982	CCDC125	1.608436928
4761	5.8690963	3.648944	RCOR3	1.608436928
4762	0.8493721	0.528073	TIGD7	1.608436928
4763	2.2425773	1.3942587	HERC6	1.608436928
4764	2.6563821	1.6515302	SLC22A5	1.608436928
4765	0.9134606	0.5679182	USP49	1.608436928
4766	8.2683578	5.1427401	GLI2	1.607772834
4767	39.061816	24.307278	C11orf58	1.607000824
4768	12.687534	7.8974383	SMAD7	1.606537948
4769	36.537575	22.772289	TAF7	1.604475262
4770	2.7088323	1.688298	FAM76A	1.604475261
4771	67.27651	41.936185	NDUFB3	1.60425917

4772	21.260136	13.252544	OAS3	1.60423049
4773	18.567834	11.577778	BLOC1S1	1.603747607
4774	1.8030657	1.1250229	ZNF767	1.602692511
4775	3.4920997	2.1788956	C21orf67	1.602692511
4776	30.846418	19.256405	AKT2	1.601878342
4777	90.754621	56.656307	TRAPPC5	1.601844974
4778	46.737294	29.178057	SARNP	1.601795984
4779	54.406226	33.972594	TRIP6	1.601473998
4780	2.7556959	1.7212075	MGC50722	1.601024777
4781	7.627234	4.7663342	ROGDI	1.600230618
4782	54.005107	33.758127	C6orf106	1.599766109
4783	15.985743	9.99255	HTRA1	1.599766109
4784	277.32698	173.39761	GPX4	1.599370204
4785	90.171086	56.411975	TNFRSF1A	1.598438734
4786	83.473369	52.232806	GRB2	1.598102346
4787	1.0107822	0.6325325	IFIT2	1.597992533
4788	5.4616519	3.4178206	OSCAR	1.597992533
4789	22.287314	13.94707	R3HDM2	1.597992533
4790	7.5165308	4.7037333	ARNTL	1.597992533
4791	2.3170644	1.4499845	RHOBTB1	1.597992533
4792	132.14621	82.706298	FLNB	1.597776883
4793	49.954262	31.285674	DCTN3	1.596713627
4794	16.228972	10.1717	RARG	1.595502478
4795	24.487786	15.352915	CXCL2	1.594992548
4796	184.15013	115.45655	MORF4L1	1.594973451
4797	9.9759887	6.2552988	C12orf65	1.594806107
4798	43.048816	27.000792	KIF20A	1.59435384
4799	34.707426	21.778156	UNC45A	1.593680627
4800	4.1965623	2.6337658	HPSE	1.593369604
4801	3.7434651	2.3494016	ZBTB46	1.593369604
4802	4.4356876	2.7846417	CEP70	1.592911476
4803	38.969502	24.465346	ARHGAP1	1.592844938
4804	49.002045	30.776901	CMC2	1.592169552
4805	153.32546	96.308492	GDI1	1.592024307
4806	12.572158	7.8997963	C1orf216	1.591453434
4807	20.364514	12.797905	IRF2BP1	1.591238074
4808	42.77195	26.883714	SAP30BP	1.590998518
4809	1.0905259	0.6855369	RAB36	1.590761798
4810	2.7420987	1.7237645	RPL23AP82	1.590761798
4811	7.4668398	4.6938768	C11orf95	1.590761798
4812	7.0484611	4.4308715	LOC284454	1.590761798
4813	6.6316468	4.1688497	TMTC4	1.590761798
4814	2.905535	1.8265054	TRAPPC2	1.590761797

4815	1.6579117	1.0422124	HOXA13	1.590761797
4816	34.876776	21.931515	SNRNP27	1.590258392
4817	45.010129	28.307282	ZCCHC17	1.590054793
4818	8.3073954	5.2265944	NDFIP2	1.589447118
4819	19.768181	12.438381	TMEM141	1.589288871
4820	365.9602	230.53171	BSG	1.587461462
4821	6.6497499	4.18908	SH2D4A	1.587401033
4822	17.259203	10.873887	EML2	1.587215513
4823	40.419074	25.466334	SBF1	1.587157116
4824	13.652545	8.6036909	B4GALT4	1.586824269
4825	58.424375	36.818942	RCN1	1.586802109
4826	75.80444	47.791196	TNFRSF10B	1.586159086
4827	49.664891	31.314171	TMBIM1	1.586019689
4828	10.15369	6.4021647	IKBKB	1.585977551
4829	33.371443	21.044823	ENO2	1.585731721
4830	9.586377	6.0464354	CAMK1	1.585459258
4831	14.150583	8.9310223	EML1	1.584430408
4832	328.55118	207.42161	PSAP	1.583977586
4833	31.03041	19.604302	TSC2	1.582836807
4834	34.964036	22.096608	PTRHD1	1.582325939
4835	47.818455	30.221422	LIMA1	1.582270187
4836	15.74574	9.9529801	DEDD2	1.582012608
4837	17.674908	11.175018	ATG12	1.581644784
4838	16.888479	10.679418	C7orf49	1.581404376
4839	2.4617842	1.5567076	RSPH3	1.581404375
4840	5.092469	3.2202194	PCSK1N	1.581404375
4841	4.6133563	2.9172528	ETFDH	1.581404375
4842	3.6178858	2.2877677	ZNF436	1.581404375
4843	8.6694192	5.4821014	RNASET2	1.581404375
4844	4.8682722	3.0784487	LOC729013	1.581404375
4845	11.770573	7.4431138	L3HYPDH	1.581404375
4846	25.188641	15.931415	CHSY1	1.581067405
4847	24.106753	15.25227	ZMYM6NB	1.580535472
4848	14.074786	8.9066447	PCYT2	1.580256767
4849	21.558569	13.642447	RANBP9	1.580256767
4850	4.4186478	2.7971174	EXOC6B	1.579714841
4851	45.569723	28.870663	GPC1	1.578409291
4852	1.3329478	0.8445984	WDR19	1.578203152
4853	2.2677619	1.4369265	ZNF302	1.578203152
4854	13.321643	8.4410193	ORMDL2	1.578203152
4855	62.787029	39.79278	SEC11A	1.57784977
4856	195.93316	124.20443	TXN	1.57750545
4857	38.539255	24.438144	RSU1	1.577012324

4858	3.5097652	2.2258183	PRPF40B	1.576842632
4859	8.6225127	5.4684768	ITPR2	1.576766826
4860	9.8182135	6.2281756	TRMU	1.576418863
4861	0.7506511	0.4764188	SAMD12	1.575611687
4862	17.33052	10.999233	YIPF1	1.575611686
4863	7.5183585	4.7717078	C2orf74	1.575611685
4864	7.0944511	4.5026647	TMEM156	1.575611685
4865	80.2601	50.944586	AGPAT1	1.575439223
4866	18.317037	11.631617	UBTD2	1.574762757
4867	44.428439	28.22034	EMC3	1.57434103
4868	23.287709	14.795519	METTL23	1.573970423
4869	5.825491	3.7011439	ITGBL1	1.573970423
4870	28.776768	18.291926	NARF	1.573195103
4871	5.0271809	3.1959341	IL6R	1.57299265
4872	66.317191	42.172174	PQBP1	1.572534319
4873	34.147804	21.719718	GUCD1	1.57220291
4874	47.011355	29.908329	GSR	1.571848281
4875	9.3254604	5.9334623	OSGIN2	1.571672656
4876	65.387835	41.605387	CPSF7	1.571619426
4877	4.6824689	2.9807987	RNF103	1.570877275
4878	2.7051529	1.7220651	ZNF264	1.570877275
4879	43.577136	27.75225	UBE2G2	1.570219936
4880	2.928163	1.8649047	HDAC11	1.570140811
4881	19.935556	12.696668	BECN1	1.570140811
4882	234.56221	149.41588	GABARAP	1.569861277
4883	6.927633	4.4140319	LOC339803	1.569456952
4884	27.610266	17.592241	ANKLE2	1.569456952
4885	6.9556366	4.4324882	TRIM62	1.569239726
4886	12.943445	8.2482268	ZNF205	1.569239726
4887	129.33565	82.431302	ARPC3	1.569011435
4888	11.570445	7.3752525	SMDT1	1.568820256
4889	44.752815	28.530097	TMEM184B	1.56861767
4890	9.2312572	5.8853368	SLC26A2	1.568518095
4891	134.82172	85.966327	NGFRAP1	1.568308462
4892	10.287146	6.5597347	CFLAR	1.568226006
4893	10.716386	6.8358679	ANKRD39	1.567670095
4894	5.2201687	3.3298898	TRIM38	1.567670094
4895	34.855292	22.236832	PSMC2	1.567457644
4896	14.65191	9.3494048	SPPL3	1.567148927
4897	9.781916	6.2428449	ZNF827	1.566900371
4898	6.00966	3.8362596	ZFHX4	1.566541552
4899	52.477593	33.51013	PLEKHM2	1.566021752
4900	82.011663	52.369845	FADD	1.56600928

4901	32.678028	20.870339	CASP4	1.565764112
4902	132.80964	84.835408	ACADVL	1.565497695
4903	11.286224	7.2096452	NMT2	1.565434026
4904	26.025928	16.625375	RUNX3	1.565434026
4905	78.940083	50.429033	COLGALT1	1.565369771
4906	5.2412426	3.3482799	MBOAT1	1.565353797
4907	180.80654	115.52133	C12orf57	1.565135544
4908	18.440198	11.787495	INTS5	1.564386473
4909	47.550136	30.401072	PFKL	1.564094056
4910	18.602234	11.893393	LDOC1L	1.5640813
4911	43.082045	27.55836	CHD3	1.563302219
4912	60.496514	38.703171	SPTBN1	1.563089339
4913	6.6597677	4.2608444	SLC30A1	1.563015952
4914	24.596907	15.738899	CYTH3	1.562809841
4915	7.7900881	4.9848821	TMEM38B	1.562742698
4916	58.917011	37.71336	TBCB	1.562231831
4917	9.0166755	5.7725465	PPAP2A	1.561992701
4918	1085.1368	694.72775	UBB	1.561959836
4919	51.438862	32.932655	NQO1	1.561940937
4920	5.7414605	3.676785	SLC25A26	1.561543724
4921	72.049676	46.183828	CIB1	1.560062886
4922	23.140807	14.83474	TMEM203	1.559906504
4923	46.783561	29.992239	RNF5	1.559855568
4924	111.01588	71.179911	KDELR2	1.559651826
4925	70.488066	45.200069	DUS1L	1.559468123
4926	11.273627	7.2337069	TNFAIP8L1	1.558485472
4927	10.010877	6.4234649	CCBL2	1.558485471
4928	11.680852	7.4960174	SLC25A20	1.558274409
4929	21.038648	13.508114	EPS15L1	1.557482261
4930	7.7021324	4.9455849	ZNF213	1.557375439
4931	32.700426	20.998804	FAM171A1	1.557251803
4932	20.961348	13.462613	GRK5	1.557004352
4933	53.889925	34.623952	ANXA7	1.556434833
4934	79.147331	50.861415	YIPF3	1.556137007
4935	18.419008	11.840069	TWSG1	1.555650388
4936	108.0702	69.483123	SERF2	1.555344584
4937	28.565225	18.372018	C15orf39	1.554822364
4938	7.7728643	5.000787	AHR	1.554328221
4939	368.74305	237.24019	TPM2	1.554302596
4940	10.579542	6.8070492	TMX3	1.554203918
4941	3849.4519	2476.8645	ACTG1	1.554163301
4942	60.473069	38.91493	NOL7	1.553981178
4943	30.649606	19.73256	IDH1	1.553250384

4944	47.262596	30.446916	MTCH2	1.552295014
4945	62.14156	40.038263	NMT1	1.552054349
4946	45.943892	29.602152	TMEM43	1.552045701
4947	0.0624636	0.0402733	PLAC4	1.550992775
4948	0.0613361	0.0395463	MIAT	1.550992766
4949	0.2011578	0.1296962	LOC284950	1.550992759
4950	0.1851343	0.119365	FGD5P1	1.550992759
4951	0.1531223	0.0987254	ADAMTSL2	1.550992758
4952	0.3209438	0.206928	CATSPER2	1.550992758
4953	0.4700741	0.3030795	CDNF	1.550992756
4954	0.1791913	0.1155333	FLJ43879	1.550992756
4955	0.3584854	0.2311329	ANKRD20A12P	1.550992755
4956	0.5192679	0.3347971	UTS2B	1.550992755
4957	0.5192679	0.3347971	MYZAP	1.550992755
4958	0.6859007	0.4422333	MAMSTR	1.550992755
4959	0.5184067	0.3342419	EPN2-AS1	1.550992754
4960	0.3725855	0.2402239	ZRANB2-AS1	1.550992754
4961	0.6558026	0.4228277	FAM46C	1.550992754
4962	0.7541598	0.4862433	H2BFXP	1.550992754
4963	0.3705978	0.2389423	TMEM37	1.550992754
4964	0.4580209	0.2953082	ZNF674	1.550992754
4965	0.6281298	0.4049856	SLC25A27	1.550992754
4966	0.2085385	0.1344549	CPAMD8	1.550992754
4967	0.2936583	0.1893357	STK4-AS1	1.550992754
4968	1.3342651	0.8602652	GALNT3	1.550992754
4969	0.5552385	0.3579891	PLAC1	1.550992754
4970	0.5513214	0.3554636	RPL19P12	1.550992754
4971	0.605226	0.3902185	ZNF230	1.550992754
4972	0.4510812	0.2908338	GLYATL2	1.550992753
4973	0.3479124	0.2243159	RPL21P44	1.550992753
4974	1.1319225	0.7298051	HOXA5	1.550992753
4975	0.2911963	0.1877483	CARD9	1.550992753
4976	2.8993828	1.8693723	CARD6	1.550992753
4977	1.0463573	0.6746371	LOC100286793	1.550992753
4978	0.9738294	0.6278749	HIGD1B	1.550992753
4979	0.1059479	0.0683097	CNR1	1.550992753
4980	3.2552449	2.0988137	GSAP	1.550992753
4981	10.387514	6.6973324	MITD1	1.550992753
4982	2.5209617	1.6253859	FAM211B	1.550992753
4983	0.7157396	0.4614719	PBX4	1.550992753
4984	1.4754738	0.9513093	RNF150	1.550992753
4985	1.7238929	1.1114771	LOC100288332	1.550992753
4986	14.229269	9.1742975	NARFL	1.550992753

4987	79.652204	51.355626	MTA2	1.550992753
4988	1.211625	0.7811932	ADAMTS15	1.550992753
4989	1.940104	1.2508788	DHRS4	1.550992753
4990	5.1396236	3.3137638	LHPP	1.550992753
4991	7.7185	4.9764901	MIR4737	1.550992753
4992	7.7185	4.9764901	HES4	1.550992753
4993	7.1045284	4.5806329	SNORD35B	1.550992753
4994	3.2360171	2.0864166	BIK	1.550992753
4995	4.4028063	2.8387021	CA5BP1	1.550992753
4996	0.1953135	0.1259281	TMC1	1.550992753
4997	0.9081907	0.5855545	AMZ2P1	1.550992753
4998	8.33598	5.3746093	SNORD26	1.550992753
4999	0.3286482	0.2118954	NR4A3	1.550992753
5000	5.7560768	3.711221	SFT2D3	1.550992752
5001	5.0456225	3.2531567	GLI4	1.550992752
5002	0.65329	0.4212076	TAS2R20	1.550992752
5003	2.2162301	1.4289107	GPR39	1.550992752
5004	0.9248499	0.5962954	PRR18	1.550992752
5005	0.4043975	0.2607346	GSTO2	1.550992752
5006	0.5709575	0.3681239	LOC100272228	1.550992752
5007	3.8993669	2.5141104	HIST1H2BJ	1.550992752
5008	0.3930418	0.2534131	MCF2L2	1.550992752
5009	0.2992812	0.1929611	GRAP	1.550992752
5010	0.5147785	0.3319026	NCF2	1.550992752
5011	0.6218818	0.4009573	CEACAM22P	1.550992752
5012	11.83341	7.6295715	EMC2	1.550992752
5013	13.730995	8.8530363	SNX24	1.550992752
5014	0.2776192	0.1789945	SEPP1	1.550992752
5015	1.4096922	0.9088967	SLC41A2	1.550992752
5016	0.7016818	0.4524082	DTX2P1-UPK3BP1-PMS2P11	1.550992752
5017	0.955961	0.6163543	C6orf99	1.550992752
5018	0.3307929	0.2132781	SMPDL3B	1.550992752
5019	0.1949481	0.1256925	ZNF682	1.550992752
5020	0.1949481	0.1256925	RASGRF1	1.550992752
5021	0.2169322	0.1398667	DHRS9	1.550992752
5022	0.1262007	0.0813677	TEX14	1.550992751
5023	0.5091193	0.3282538	INGX	1.550992751
5024	0.1874939	0.1208864	MBNL1-AS1	1.550992751
5025	0.3745947	0.2415193	TRIM46	1.550992751
5026	0.4536999	0.2925223	CH25H	1.550992751
5027	0.4792629	0.309004	LOC151475	1.550992751
5028	0.2997116	0.1932386	NODAL	1.55099275
5029	0.2791065	0.1799534	GCNT3	1.55099275

5030	0.3601374	0.232198	HVCN1	1.55099275
5031	0.068201	0.0439725	MACC1	1.550992749
5032	0.1285623	0.0828903	GPR12	1.550992749
5033	0.1136311	0.0732635	1-Mar	1.550992749
5034	0.1668976	0.107607	AP3B2	1.550992749
5035	0.2808619	0.1810852	PHKG1	1.550992749
5036	0.2651393	0.1709481	ALOX12	1.550992748
5037	0.2065406	0.1331667	AKAP3	1.550992748
5038	0.1710062	0.1102559	AXDND1	1.550992747
5039	0.2193294	0.1414123	RNF138P1	1.550992747
5040	0.1118023	0.0720844	SH3BP5-AS1	1.550992745
5041	0.1900299	0.1225215	CLDN16	1.550992745
5042	0.0996968	0.0642793	PLXDC1	1.550992743
5043	0.1476614	0.0952045	BMS1P1	1.550992741
5044	93.351718	60.226172	NGRN	1.550019123
5045	38.457763	24.829086	MAPK3	1.548899645
5046	31.056996	20.05239	GLG1	1.548792762
5047	82.750593	53.516968	RABAC1	1.546249656
5048	4.5651588	2.9525766	SLX4	1.546161
5049	5.449858	3.5261594	IFT172	1.545550673
5050	40.72403	26.385146	NR1H2	1.543445343
5051	28.033044	18.173568	TSR2	1.542517382
5052	8.8523681	5.7399789	PTPN21	1.542230082
5053	125.62109	81.462547	GFPT2	1.542071678
5054	15.187205	9.8544931	SGCB	1.541145179
5055	102.74703	66.671721	DBI	1.541088584
5056	16.999355	11.036419	ETNK2	1.540296251
5057	5.3790431	3.4929015	C3orf18	1.539992804
5058	23.890595	15.521909	ADPRHL2	1.539153114
5059	28.311429	18.395615	TYK2	1.539031369
5060	113.5119	73.755953	ASNA1	1.539020177
5061	9.0919741	5.9081929	RREB1	1.538875622
5062	9.9275677	6.451989	RILPL1	1.538683286
5063	3.0346966	1.9730577	SNX29	1.538067813
5064	147.66159	96.04522	RAB5C	1.537417324
5065	6.5647036	4.2710598	IKBKE	1.537019845
5066	112.69902	73.331314	COPG1	1.536847187
5067	5.7276818	3.7284221	LYPLAL1	1.536221393
5068	4.3306088	2.8206442	MOSPD1	1.535326159
5069	15.8319	10.31175	CLEC4GP1	1.535326159
5070	20.819769	13.564469	ANO6	1.534875321
5071	4.5356134	2.9551116	CYLD	1.534836578
5072	2.4492532	1.5963166	RBM43	1.534315411

5073	39.811982	25.949381	NDUFA11	1.53421702
5074	22.199451	14.47949	CDK5	1.53316525
5075	4.3027758	2.8076317	KRBA1	1.532528553
5076	22.545875	14.714776	GALNT6	1.532192841
5077	12.692751	8.287483	SH3GLB1	1.531556753
5078	23.324827	15.229697	ALCAM	1.531535855
5079	5.521158	3.6065964	SEC14L2	1.53084999
5080	2.0259542	1.3238823	LIPE	1.530312849
5081	2.0007217	1.3073939	CORO7	1.530312849
5082	175.59123	114.77705	TECR	1.529846166
5083	8.1459088	5.3250065	TTI2	1.529746277
5084	4.5999469	3.0081768	CDAN1	1.529147784
5085	54.579098	35.703162	UBE2D2	1.528690896
5086	1.0924426	0.7147087	POLR2J4	1.528514597
5087	12.695377	8.3112514	LRCH4	1.527492863
5088	39.893148	26.119199	DVL3	1.527349571
5089	3.3104712	2.1688469	ZNF45	1.52637382
5090	6.1542124	4.0319169	ASAP3	1.52637382
5091	5.0197238	3.2886595	LINC00623	1.52637382
5092	53.560278	35.097856	ACTR3	1.526027075
5093	25.216629	16.538697	RFXANK	1.52470474
5094	12.666921	8.3106168	CCNJL	1.52418547
5095	0.8572331	0.5625693	LOC440297	1.523782353
5096	6.4250011	4.2164822	MBTPS2	1.523782353
5097	3.3958405	2.2285601	VAMP4	1.523782353
5098	22.950749	15.067484	WIBG	1.523197183
5099	12.797846	8.4041938	ZDHHC24	1.522792885
5100	5.8719689	3.8573751	PTHLH	1.522270665
5101	16.193204	10.639139	TMX4	1.522040889
5102	75.668866	49.720468	ZMAT2	1.52188565
5103	80.112842	52.644626	GIPC1	1.521766769
5104	2.005384	1.3188273	ARHGEF26-AS1	1.520581131
5105	39.820273	26.195356	GABARAPL2	1.520127225
5106	18.963	12.47588	GGA1	1.519972898
5107	52.480961	34.539025	GTF3C6	1.51946851
5108	18.209665	11.985248	RNASEH1	1.519339839
5109	13.454363	8.859246	MGC72080	1.518680404
5110	5.1701116	3.4043447	WDR60	1.518680403
5111	16.789552	11.0567	MRPL22	1.518495762
5112	127.0064	83.644075	MAP2K2	1.518414822
5113	6.8474121	4.5108328	HOXC11	1.517992907
5114	7.1590617	4.7172392	LYSMD1	1.51763807
5115	18.686676	12.313795	SERTAD2	1.517539969

5116	1.6177802	1.066767	DACT3	1.516526248
5117	1.9039821	1.2554891	LOC100287015	1.516526247
5118	4.1057812	2.7073591	KLF5	1.516526247
5119	0.0151563	0.01	DNAH9	1.5156327
5120	4.4541005	2.939345	INVS	1.515337747
5121	94.836072	62.587789	DCTN1	1.5152488
5122	15.855452	10.467633	LSS	1.514712221
5123	15.398036	10.174513	INPP1	1.513392928
5124	13.445129	8.8921453	TTC3	1.512023085
5125	4.7838408	3.1641417	ATP9A	1.511892095
5126	105.6646	69.890069	C19orf10	1.511868611
5127	316.91522	209.63096	PPP1CA	1.511776753
5128	1.8331438	1.2130195	CEP19	1.511223709
5129	1.212367	0.8022419	BCDIN3D	1.511223708
5130	0.8801046	0.5823788	FPGT	1.511223708
5131	3.4536332	2.2853223	IQCC	1.511223707
5132	0.6073923	0.4019208	GOLGA8B	1.511223706
5133	22.675136	15.01796	DEDD	1.509867945
5134	11.616864	7.6942252	KIAA0907	1.509815954
5135	181.90102	120.48379	STMN1	1.509755132
5136	138.16084	91.532647	PPP2R1A	1.509416024
5137	16.682469	11.052419	CYB561	1.509395247
5138	7.9984093	5.2990821	RHOT1	1.509395246
5139	4.6254346	3.064429	PPP1R3B	1.509395246
5140	15.3083	10.142486	TMEM131	1.509324291
5141	9.0462806	5.9945903	RECQL5	1.50907403
5142	11.851711	7.8551159	ZDHHC8	1.508788868
5143	183.82116	121.84756	TCEB2	1.508615902
5144	3.9705947	2.6331782	WASH1	1.507909621
5145	1.7903737	1.1873216	GATS	1.50790962
5146	0.9655788	0.6403426	GPR155	1.50790962
5147	130.25914	86.398551	PRNP	1.507654259
5148	4.5400442	3.0117256	EPG5	1.507456114
5149	25.616254	17.000706	MED29	1.506775854
5150	16.226526	10.769732	ACAA1	1.506678674
5151	25.034787	16.617149	SPOP	1.506563273
5152	62.261463	41.328362	AP1B1	1.506506914
5153	3.1139087	2.0676184	ZNF280D	1.506036441
5154	6.2818807	4.1722271	TBC1D9	1.505642088
5155	17.679135	11.744005	UNC50	1.50537532
5156	0.5329059	0.3543274	GNRHR2	1.503992974
5157	0.8530038	0.5671595	DDX12P	1.503992973
5158	1.3510502	0.8983089	FAM133B	1.503992972

5159	4.2314619	2.8134852	FAM24B	1.503992972
5160	1.4310695	0.9515134	TTC12	1.503992971
5161	0.9709936	0.6456105	CDKL5	1.503992971
5162	36.675134	24.39318	COL13A1	1.503499536
5163	20.209144	13.444055	DMAP1	1.50320306
5164	6.1593979	4.0977348	MDFIC	1.503122606
5165	38.960595	25.931889	STK10	1.502420264
5166	50.45612	33.591308	PKIG	1.502058825
5167	3.347122	2.2288072	LOC150776	1.501754887
5168	17.759262	11.829982	CAT	1.5012078
5169	24.098924	16.061033	PON2	1.500459154
5170	91.01958	60.686038	DAZAP2	1.499843829
5171	8.058114	5.3746093	DNLZ	1.499292994
5172	50.245787	33.531537	UFC1	1.498463586
5173	164.57821	109.84785	MLF2	1.498237897
5174	19.676261	13.135935	DSG2	1.497895704
5175	9.7573491	6.5140377	ASAP2	1.497895703
5176	13.475979	8.996921	ST3GAL5	1.497843468
5177	42.397394	28.308168	HSD17B12	1.497708879
5178	88.089434	58.820524	CSNK1E	1.49759689
5179	27.904985	18.645936	STK11	1.496571955
5180	2.3894043	1.596585	MFSD6	1.496571954
5181	10.458004	6.9879728	PAQR5	1.496571954
5182	63.583751	42.488465	MDH1	1.496494432
5183	12.607799	8.4277125	OCEL1	1.495993009
5184	46.569721	31.180587	TMEM50A	1.493548577
5185	4.1916351	2.806494	NR1H3	1.493548577
5186	3.9864531	2.6691151	ITPKC	1.493548576
5187	4.9079592	3.2861062	MED31	1.493548576
5188	3.2759293	2.1933865	ZNF789	1.493548576
5189	0.9689533	0.6487592	IL18BP	1.493548576
5190	33.383981	22.358331	MICB	1.493133817
5191	13.860299	9.287478	DCP1B	1.492364161
5192	30.413194	20.388754	MPC1	1.491665162
5193	8.4619833	5.6748801	RNF24	1.491129874
5194	5.2353197	3.5115711	FAM181A-AS1	1.490876754
5195	12.835888	8.6102979	KIAA0319L	1.490760024
5196	8.7784874	5.8909321	VEZT	1.490169507
5197	60.415953	40.557036	PSMA2	1.489654056
5198	55.460105	37.230191	ACP1	1.489654056
5199	2.3415674	1.5726268	ZNF514	1.488953043
5200	267.96702	180.04941	GPX1	1.48829715
5201	3.7497928	2.5201164	SLC25A42	1.487944266

5202	36.212583	24.345883	SMARCC2	1.487421198
5203	12.043013	8.0993987	FBXO42	1.486902142
5204	72.356411	48.669045	DCAF7	1.486702898
5205	52.649513	35.419152	NUP62	1.486470147
5206	36.312034	24.430042	AMZ2	1.486368055
5207	4.5422303	3.0559257	B3GALTL	1.486368055
5208	21.737816	14.624787	MRPL55	1.486368054
5209	44.190107	29.73183	TSPAN14	1.486289531
5210	138.87985	93.449117	UBXN1	1.486154771
5211	37.772409	25.422513	POLD4	1.48578585
5212	19.550881	13.159481	DAGLB	1.485687794
5213	16.532827	11.130192	MAU2	1.485403507
5214	2.3788251	1.6034592	LOC146880	1.483558286
5215	14.982971	10.099347	ERICH1	1.483558285
5216	76.66159	51.674135	FBXO7	1.483558285
5217	7.641315	5.1506672	C5orf38	1.483558285
5218	6.7511618	4.5506549	RAB4A	1.483558285
5219	15.09162	10.173243	GOLGA3	1.483462019
5220	6.8533416	4.6209442	TMEM65	1.483104181
5221	104.84606	70.701485	BABAM1	1.482940025
5222	24.314005	16.396682	SLC9A1	1.482861288
5223	5.3817064	3.6296418	IL1A	1.482710053
5224	71.252564	48.071587	PMP22	1.482217845
5225	5.0146494	3.3835677	HDHD3	1.482059741
5226	11.968185	8.0753728	CTNNBIP1	1.482059741
5227	69.059311	46.626153	MBD3	1.481128214
5228	15.415455	10.407914	ZNF580	1.481128214
5229	171.17168	115.57192	DDB1	1.481083625
5230	10.937842	7.3870344	NUAK1	1.480681081
5231	21.807571	14.73548	MYD88	1.479936213
5232	55.763846	37.682188	SEC61B	1.479846296
5233	20.285956	13.709666	PAPSS1	1.479682742
5234	31.523682	21.32879	ZDHHC7	1.47798733
5235	32.914999	22.273968	TCEB1	1.477733986
5236	65.32136	44.216151	SLC7A1	1.477318995
5237	0.2018984	0.1366823	FAM227A	1.477135961
5238	1.2872112	0.8714237	RAB30	1.477135956
5239	1.9101696	1.2931576	TMEM91	1.477135956
5240	1.0165829	0.6882122	ZNF837	1.477135955
5241	0.8752604	0.5925388	4-Sep	1.477135955
5242	8.5569037	5.7929019	FIZ1	1.477135955
5243	6.9120896	4.6793862	TMEM175	1.477135955
5244	0.8409988	0.5693442	ZNF503-AS2	1.477135955

5245	8.7793365	5.9434858	ZNF576	1.477135955
5246	3.5333927	2.3920565	KLF4	1.477135955
5247	1.1501076	0.7786065	MYCL1	1.477135954
5248	18.215087	12.339664	VPS39	1.47614125
5249	23.180385	15.703621	PEX19	1.47611724
5250	73.6668	49.918246	DCXR	1.475748973
5251	13.350821	9.0477406	SNAP29	1.475597272
5252	114.23437	77.416218	ANXA11	1.475587003
5253	27.528447	18.656794	GDE1	1.475518653
5254	21.747524	14.739325	DGCR6L	1.475476252
5255	7.6086637	5.156751	SLC12A2	1.475476252
5256	60.459183	40.986409	MYL6B	1.475103199
5257	40.534135	27.493301	COMMD7	1.474327712
5258	61.090917	41.45861	CHMP1A	1.473539931
5259	21.18202	14.378098	SLC25A28	1.473214355
5260	74.903631	50.853988	POLR2K	1.472915567
5261	25.048017	17.010983	EVA1A	1.472461474
5262	288.3947	195.90318	RAD23A	1.472128714
5263	12.371419	8.4037618	XXYLT1	1.472128714
5264	89.857032	61.056345	CLSTN1	1.471706685
5265	13.869308	9.4241289	CABIN1	1.471680623
5266	52.214046	35.480666	ETFA	1.47161968
5267	12.422791	8.445739	C5orf24	1.470894535
5268	4.3383696	2.9497288	DSTYK	1.47076899
5269	21.330553	14.509245	YIF1B	1.470135311
5270	14.564074	9.9085472	DGCR8	1.469849634
5271	7.3915094	5.0304225	FAM214B	1.469361555
5272	9.2851262	6.3191569	FGFR1OP2	1.469361555
5273	5.4051744	3.678587	RPS2P32	1.469361555
5274	1.7366625	1.1819164	OLFML2B	1.469361554
5275	10.606615	7.2201501	HRH1	1.469029722
5276	36.818437	25.06384	ZNF768	1.46898624
5277	31.755669	21.620258	ZBTB7A	1.4687923
5278	5.2322842	3.565183	KLHL20	1.467606046
5279	13.574453	9.2509305	GAS2L1	1.46736079
5280	229.49856	156.44122	SRP14	1.466995433
5281	6.2653374	4.2713442	FDXR	1.466830355
5282	7.1992555	4.9104385	PIGV	1.466112552
5283	18.43182	12.575755	LAMP2	1.465663054
5284	15.242548	10.400884	BPNT1	1.465504964
5285	4.8815591	3.3325169	LOC81691	1.464826489
5286	4.8798781	3.3313694	C9orf9	1.464826489
5287	3.0726726	2.0976359	GPR157	1.464826489

5288	16.62539	11.349734	PHF12	1.464826488
5289	14.952296	10.212689	BCR	1.464090025
5290	49.776449	34.010247	LDLR	1.463572109
5291	13.04794	8.9221357	CC2D1B	1.462423446
5292	16.452592	11.250677	HARS2	1.462364596
5293	12.858497	8.7938461	DOK1	1.46221539
5294	90.625104	62.00524	RAB11B	1.461571699
5295	46.292431	31.678731	CNPY2	1.461309498
5296	7.3606146	5.0382899	USP9X	1.460935109
5297	1.504237	1.0304702	GFOD1	1.459757885
5298	33.658419	23.068215	PRPSAP1	1.459082071
5299	87.983764	60.324606	WDR45B	1.458505412
5300	17.907166	12.278387	FAM160B2	1.458429867
5301	6.8603756	4.7046931	KIAA0355	1.458198315
5302	1.9024021	1.3046251	PLD1	1.458198314
5303	37.336089	25.606079	SRA1	1.458094749
5304	17.713363	12.148824	TSPYL1	1.458031042
5305	13.582688	9.3174829	LRRC45	1.45776368
5306	4.938246	3.3884798	KATNAL1	1.457363269
5307	2.6498706	1.8187254	RABL2B	1.456993192
5308	7.341346	5.0386962	KRTAP2-3	1.456993192
5309	8.6814383	5.9598651	ZNRF1	1.456650128
5310	15.749562	10.812987	C17orf70	1.45654127
5311	3.5984899	2.4714326	PLCE1	1.456034013
5312	77.788817	53.427748	DBNL	1.455962843
5313	250.54771	172.10827	PARK7	1.455756355
5314	14.004446	9.6205506	GPX8	1.455680349
5315	19.077256	13.109242	ZNF282	1.45525246
5316	3.9546472	2.7174991	ERN1	1.455252459
5317	21.397516	14.70641	MAST2	1.454978916
5318	8.6992703	5.9792352	WDFY2	1.454913556
5319	34.294976	23.577495	DESI1	1.45456399
5320	60.610997	41.679866	MED10	1.454203251
5321	4.6288142	3.1833816	ZDHHC1	1.454055706
5322	3.6180469	2.488245	INSIG2	1.454055705
5323	12.243389	8.421572	MAPK7	1.453812755
5324	87.819179	60.409861	STK25	1.453722589
5325	162.66824	111.90506	TBCA	1.45362716
5326	15.675071	10.797206	RARS2	1.451770994
5327	11.82064	8.1433474	CBY1	1.45157014
5328	51.333283	35.366979	R3HDM4	1.451446634
5329	8.6228388	5.9448771	MORN4	1.450465445
5330	12.715548	8.7695237	CCDC117	1.449970237

5331	17.886584	12.336927	COMMD9	1.449841051
5332	9.8402399	6.7871164	GALNT11	1.449841051
5333	189.34583	130.65419	KDELR1	1.449213601
5334	25.534591	17.620967	ACAT2	1.449102718
5335	19.430843	13.412615	NEK6	1.448699113
5336	38.680661	26.704226	PCMT1	1.448484611
5337	0.2928722	0.2023167	FDPSP2	1.447593241
5338	0.8369458	0.5781636	POU5F1	1.447593237
5339	0.8200093	0.5664639	FAM41C	1.447593237
5340	0.2846432	0.196632	RFPL1S	1.447593237
5341	0.585157	0.4042275	C1QL3	1.447593237
5342	0.7179117	0.4959347	DPY19L2	1.447593237
5343	1.6101507	1.112295	LURAP1L	1.447593237
5344	10.436541	7.2095812	PLEKHA3	1.447593236
5345	0.7597898	0.5248642	TMEM200C	1.447593236
5346	6.9715484	4.8159581	ANAPC10	1.447593236
5347	68.997132	47.663342	ICT1	1.447593236
5348	1.6024861	1.1070003	ZNF225	1.447593236
5349	23.077007	15.941638	CDC26	1.447593236
5350	26.908727	18.588597	FUCA2	1.447593236
5351	18.309338	12.648123	NUB1	1.447593236
5352	1.1976983	0.8273721	DNAJC12	1.447593236
5353	110.52343	76.349784	SAE1	1.447593235
5354	0.3854152	0.2662455	14-Sep	1.447593235
5355	13.933109	9.6250166	PEX10	1.447593235
5356	0.7189731	0.4966679	DENND6B	1.447593235
5357	1.0467327	0.7230848	ZNF440	1.447593235
5358	1.1277901	0.7790794	PBLD	1.447593235
5359	20.731182	14.327149	AFF4	1.446985897
5360	47.654194	32.955911	SHKBP1	1.445998385
5361	33.226156	22.981742	POLR2J	1.445763156
5362	20.070451	13.886624	RAB21	1.445308164
5363	14.18682	9.817178	KCTD17	1.445101681
5364	39.612882	27.42226	RBFOX2	1.444552074
5365	139.86876	96.941001	PRR13	1.442823571
5366	57.916648	40.147684	CBR1	1.442590033
5367	15.908749	11.032197	BRD8	1.442029136
5368	219.36789	152.13792	YWHAQ	1.441901519
5369	2.9845508	2.0706971	ARL4A	1.441326598
5370	1.5197173	1.0543879	SLC35E2	1.441326598
5371	20.250339	14.052006	TMEM185B	1.441099547
5372	52.792368	36.6379	TOX2	1.440922299
5373	39.931736	27.71839	MLLT1	1.440622482

5374	80.255849	55.716212	UBE2L3	1.44043981
5375	8.4486284	5.8662575	LIMK2	1.440207556
5376	206.93961	143.74844	CDKN1A	1.439595483
5377	2.9864684	2.0759519	ELAC1	1.438601973
5378	33.953243	23.607101	GPR176	1.438263956
5379	5.5866131	3.8852597	KIAA0556	1.437899531
5380	29.58063	20.578459	NBR1	1.437456028
5381	9.9107281	6.8950579	PLCXD1	1.43736691
5382	14.113131	9.8222685	DEXI	1.436850429
5383	7.0476443	4.9049255	C14orf159	1.436850429
5384	14.547536	10.125849	MADD	1.436673155
5385	13.607386	9.4731585	NDE1	1.436414909
5386	115.70608	80.569405	SEC13	1.436104401
5387	1.4941174	1.0403961	FLJ38109	1.4361044
5388	13.347824	9.2988422	CUL7	1.435428587
5389	9.6533828	6.7274145	C9orf37	1.434932071
5390	18.619333	12.979198	STAT5B	1.434551855
5391	3.528023	2.4598359	C2orf42	1.434251362
5392	2.1443356	1.4950905	TRPC1	1.434251362
5393	60.044182	41.870017	MED15	1.434061578
5394	3.9345	2.7446937	ZNF324	1.433493302
5395	2.919699	2.036772	RGAG4	1.433493302
5396	68.177914	47.582966	SLC35B1	1.432821876
5397	18.833032	13.15054	C9orf69	1.432110955
5398	1.0484044	0.7322868	ZNF497	1.431685618
5399	0.7180687	0.5015548	ALS2CL	1.431685618
5400	32.855777	22.956222	YIPF2	1.43123625
5401	23.1555	16.181619	PI4K2A	1.430975456
5402	32.601607	22.798433	FHL3	1.429993318
5403	9.5798922	6.6992566	C19orf52	1.429993318
5404	38.544108	26.957288	COPRS	1.429821444
5405	315.36562	220.57301	IFITM3	1.429756122
5406	1.6181689	1.1321042	SLC8A1	1.429346263
5407	1.8868766	1.3200976	ZNF248	1.429346262
5408	120.23175	84.124042	MAP4	1.429219786
5409	37.498979	26.261664	MZT2B	1.42789809
5410	233.68843	163.72932	AP2S1	1.427285171
5411	17.914221	12.558912	VAMP7	1.426415021
5412	3.6338187	2.5479022	FRS2	1.426200232
5413	7.9678861	5.5895068	ZNF367	1.425507902
5414	4.0831251	2.8648753	IQCK	1.425236583
5415	10.650902	7.4748636	KLHL36	1.424895781
5416	34.125504	23.954185	CARHSP1	1.424615566

5417	86.116624	60.451155	HMGB2	1.424565396
5418	102.41091	71.940183	GADD45A	1.423556475
5419	18.032936	12.668722	NSMCE2	1.42342192
5420	107.71704	75.686744	SSR4	1.423195597
5421	16.550688	11.632382	CES2	1.422811533
5422	34.866574	24.516542	ZMIZ1	1.422165281
5423	0.4736352	0.3331369	THAP2	1.42174336
5424	0.5407866	0.3803687	ZNF606	1.421743359
5425	0.7084037	0.4982641	VSIG1	1.421743358
5426	0.6227641	0.4380285	SFTPB	1.421743357
5427	1.5536391	1.0927704	ZNF181	1.421743357
5428	2.8637033	2.0142196	CPEB1	1.421743357
5429	51.840672	36.462749	PREB	1.421743357
5430	2.1304782	1.498497	OVCA2	1.421743357
5431	5.8779346	4.1343148	HIST1H4C	1.421743357
5432	6.5559423	4.6111996	FST	1.421743357
5433	1.8561899	1.3055731	AGPHD1	1.421743357
5434	2.7355543	1.9240845	ZC2HC1A	1.421743356
5435	1.0813182	0.7605579	NUDT13	1.421743356
5436	13.734324	9.662853	ARHGEF11	1.421352875
5437	63.959114	45.025136	WASF2	1.420520176
5438	18.264226	12.862829	DOLPP1	1.419922942
5439	39.114983	27.552255	CAST	1.419665392
5440	6.5836036	4.6378015	SUOX	1.419552689
5441	7.1476785	5.0404964	SLC39A11	1.418050517
5442	18.849268	13.296142	MAFK	1.417649484
5443	5.0829146	3.5856432	ZNRF2	1.417574021
5444	3.0028746	2.1183195	DIXDC1	1.417574021
5445	33.999918	23.984581	TSPAN17	1.417574021
5446	13.047008	9.2077697	RHPN2	1.416956343
5447	20.989162	14.81545	APEX2	1.416707666
5448	18.419827	13.003087	MAD2L1BP	1.416573381
5449	13.900064	9.812456	MFSD5	1.41657338
5450	5.3961548	3.8096822	FBF1	1.416431738
5451	12.10921	8.5490952	PNKP	1.416431737
5452	8.8882945	6.2771299	DENND1A	1.415980645
5453	13.790004	9.7402305	HPS6	1.415778
5454	6.1328659	4.3343623	COA5	1.414940757
5455	12.729179	8.9962626	MACROD1	1.414940756
5456	1.5664605	1.1070856	TMEM198B	1.414940756
5457	2.0586577	1.4549427	COX20	1.414940756
5458	11.069257	7.8284437	NR3C1	1.413979259
5459	22.95013	16.23704	FKBP3	1.413442937

5460	30.345109	21.471104	CREB3	1.413299929
5461	3.5675906	2.5246077	PCBD2	1.41312673
5462	29.438	20.831819	LSM1	1.41312673
5463	14.070344	9.9579866	HIP1R	1.412970774
5464	8.7813183	6.2178042	MRPS36	1.412286084
5465	22.479479	15.918192	AGBL5	1.412187954
5466	383.55008	271.60151	DYNLRB1	1.412179448
5467	43.776226	31.007361	NDUFS4	1.411801096
5468	21.910381	15.528566	GPX7	1.410972574
5469	63.538089	45.036424	BNIP3	1.410815565
5470	3.0767643	2.1821125	SLC16A5	1.409993412
5471	0.8712354	0.6179003	TMEM182	1.409993412
5472	9.5693648	6.7868153	ALKBH6	1.409993412
5473	5.8429766	4.1439744	CRNDE	1.409993411
5474	225.4117	159.90148	PSMB3	1.409691162
5475	80.388906	57.041844	ROMO1	1.409297119
5476	28.681872	20.363227	NDUFC1	1.408513103
5477	255.75465	181.58909	SH3BGRL3	1.408425214
5478	14.686495	10.427989	MRPS18C	1.40837273
5479	7.2809884	5.1721544	IRGQ	1.407728362
5480	32.624842	23.181222	RBM10	1.407382312
5481	46.001227	32.692544	MRPL16	1.407086209
5482	18.177231	12.923389	BPGM	1.406537545
5483	4.9285298	3.5047736	TSC1	1.406233429
5484	4.8081314	3.419156	BAIAP2-AS1	1.406233429
5485	828.26443	589.16889	TPI1	1.40581833
5486	13.129377	9.3402685	SIPA1L3	1.405674513
5487	15.26158	10.857796	ACOT8	1.405587182
5488	8.050418	5.7291298	LOC100506054	1.405172921
5489	23.468412	16.704867	LRWD1	1.40488474
5490	13.625715	9.7045339	TBCE	1.404056597
5491	33.383662	23.782166	ELF4	1.403726774
5492	155.98993	111.14161	PSMD3	1.403524121
5493	0.5548754	0.3954134	ZNF793	1.403279159
5494	2.3968466	1.7080326	LOC440434	1.403279157
5495	3.5688062	2.5431905	STAG3L4	1.403279157
5496	1.1587915	0.825774	APOL4	1.403279157
5497	5.2488358	3.7404074	DNAJC16	1.403279157
5498	5.9568308	4.2449364	TIA1	1.403279157
5499	11.346793	8.0884022	SUPT7L	1.402847246
5500	27.913724	19.908155	PLXNA1	1.402125145
5501	6.3809619	4.5517863	SNIP1	1.401858834
5502	15.493442	11.053812	SMYD3	1.401637895

5503	6.0219657	4.2963776	FBXL14	1.401637895
5504	49.208256	35.128593	COPS7A	1.40080351
5505	13.728963	9.8009139	ZNF629	1.400783995
5506	48.103238	34.347382	MBTPS1	1.400492108
5507	10.358237	7.3971642	ARSB	1.400298389
5508	67.560935	48.250856	CUEDC2	1.400201791
5509	9.008743	6.4345039	GLCE	1.400067992
5510	6.2988686	4.4996085	KIAA1033	1.399870382
5511	59.893835	42.786694	ATP6V1G1	1.399823869
5512	91.041873	65.040313	NELFE	1.399776068
5513	15.929035	11.382479	PHF15	1.399434556
5514	5.2099875	3.7242537	CCDC61	1.39893464
5515	2.0361888	1.4555282	DOK3	1.39893464
5516	21.16198	15.127211	C1orf122	1.398934639
5517	5.043005	3.6060176	CPEB4	1.398497062
5518	401.04664	286.7785	AP2M1	1.398454341
5519	19.583123	14.012643	PPP1CB	1.397532375
5520	3.1777905	2.2742514	NCOA7	1.397290769
5521	6.5609163	4.6964545	IFIT5	1.396993472
5522	1.9398146	1.3889899	DMXL1	1.396564903
5523	33.719141	24.151415	R3HCC1	1.396155913
5524	12.661447	9.0690791	CRTC3	1.396111619
5525	17.172288	12.302005	ZNHIT3	1.395893478
5526	42.458478	30.420422	NDST1	1.395722851
5527	289.10594	207.16125	PRKCSH	1.395559931
5528	7.8132974	5.6021703	GXYLT2	1.394691157
5529	9.8030584	7.0288381	C4orf27	1.394691157
5530	8.1252318	5.8321796	TLE1	1.393172438
5531	16.094453	11.55953	GTF2IRD1	1.392310327
5532	4.6295871	3.3251115	SEMA4F	1.392310326
5533	5.6674301	4.0716737	LOC100132352	1.391916573
5534	26.384301	18.961255	RPTOR	1.391484971
5535	430.75878	309.71507	IER3	1.39082277
5536	5.7452266	4.1316359	ZBTB18	1.390545226
5537	7.8503535	5.6500492	RRNAD1	1.389431008
5538	399.19663	287.42608	EIF1	1.388867122
5539	40.06983	28.855388	ZDHHC5	1.388642902
5540	14.332421	10.322175	KCTD9	1.388507799
5541	5.5970151	4.0309569	FBXW9	1.388507798
5542	5.5212945	3.9764231	PLEKHF1	1.388507798
5543	16.074761	11.578968	EPHB4	1.388272398
5544	5.2930152	3.8141525	ZSWIM7	1.387730358
5545	9.8538383	7.1030078	LRSAM1	1.387276851

5546	2.9165324	2.102662	KIAA1919	1.387066689
5547	15.996526	11.534033	LPP	1.386897918
5548	14.179023	10.224192	IFFO2	1.386811088
5549	30.51805	22.013721	CDK9	1.386319448
5550	45.371857	32.762969	RNF11	1.384851813
5551	7.7354861	5.5886947	SLC35B4	1.384131099
5552	84.674749	61.273628	RHEB	1.381911782
5553	26.792336	19.401693	MKRN1	1.380927757
5554	23.503703	17.03018	B3GALT6	1.38012067
5555	33.683907	24.417126	REPIN1	1.379519739
5556	49.313464	35.749734	CDCA5	1.379407871
5557	54.120991	39.238123	TCEA1	1.379296138
5558	107.20244	77.727527	ARPC1A	1.379208181
5559	0.1678273	0.1217321	ZNF471	1.378660229
5560	0.2073111	0.1503714	ESRP2	1.378660229
5561	0.1063534	0.0771426	KIAA1210	1.378660229
5562	0.2248107	0.1630646	LINC00672	1.378660228
5563	0.1712755	0.1242333	GBP6	1.378660228
5564	0.1215512	0.0881662	DNAH10	1.378660228
5565	0.4182629	0.3033836	STAG3L3	1.378660228
5566	0.335587	0.2434153	RBM26-AS1	1.378660227
5567	0.5039891	0.3655644	NUTM2A	1.378660227
5568	0.6206984	0.4502186	ANXA2P1	1.378660226
5569	0.3695027	0.2680158	LOC283143	1.378660226
5570	0.2153444	0.1561983	LOC401320	1.378660226
5571	1.3986544	1.0145026	ZNF419	1.378660226
5572	0.4675255	0.3391158	LOC100506472	1.378660225
5573	0.5129834	0.3720883	UPK3B	1.378660225
5574	11.002276	7.9804118	NUMBL	1.378660225
5575	0.7690018	0.5577892	LOC100506963	1.378660225
5576	3.8150938	2.7672473	HIST2H2AC	1.378660225
5577	0.4950107	0.359052	C12orf61	1.378660225
5578	0.8805613	0.638708	C5orf63	1.378660225
5579	0.4195259	0.3042997	ASH1L-AS1	1.378660225
5580	2.6775096	1.9421099	RNF122	1.378660225
5581	0.254845	0.1848498	NR2E1	1.378660225
5582	1.3401897	0.9720957	SCG5	1.378660225
5583	9.6036636	6.9659394	EHD3	1.378660225
5584	55.975072	40.601064	OAZ2	1.378660225
5585	1.8572551	1.3471449	PEX12	1.378660225
5586	3.4132766	2.4757925	ANKRA2	1.378660225
5587	3.9600855	2.8724159	SCARNA17	1.378660225
5588	0.3021377	0.2191532	MAGEA10	1.378660225

5589	30.178824	21.889965	WTAP	1.378660225
5590	2.7241765	1.9759593	HARBI1	1.378660225
5591	8.4974312	6.1635427	COG7	1.378660225
5592	6.9246669	5.0227509	SEC61A2	1.378660225
5593	1.3901575	1.0083394	PDE4D	1.378660225
5594	0.6749781	0.4895899	PTGER4P2-CDK2AP2P2	1.378660225
5595	1.5720849	1.140299	LOC389791	1.378660224
5596	3.6180469	2.6243209	C19orf40	1.378660224
5597	2.9688888	2.1534594	SH3D21	1.378660224
5598	0.9387365	0.6809049	HMGB3P1	1.378660224
5599	0.1528416	0.1108624	TMEM236	1.378660224
5600	1.5115104	1.0963618	ZNF773	1.378660224
5601	0.5249358	0.3807579	TMEM27	1.378660224
5602	1.0316807	0.7483212	MDP1	1.378660224
5603	0.2824798	0.2048945	LOC286467	1.378660224
5604	1.4522613	1.053386	SOCS2-AS1	1.378660224
5605	0.8220888	0.5962954	INE1	1.378660224
5606	0.4920885	0.3569324	ZNF383	1.378660224
5607	0.3574605	0.2592811	AKR7L	1.378660224
5608	0.8618227	0.6251161	APLF	1.378660224
5609	0.8124737	0.5893212	HUS1B	1.378660224
5610	0.2212896	0.1605106	PALM2-AKAP2	1.378660223
5611	0.3436101	0.2492348	ANKFN1	1.378660223
5612	0.311044	0.2256133	CLHC1	1.378660223
5613	0.5155213	0.3739292	LOC100505666	1.378660222
5614	0.120115	0.0871244	PAPPA2	1.378660222
5615	0.2217016	0.1608095	ARL11	1.378660221
5616	0.2821452	0.2046517	KCNT2	1.378660221
5617	0.2202373	0.1597473	NUDT4P1	1.378660221
5618	0.1743929	0.1264945	DGKI	1.37866022
5619	0.1250522	0.0907056	POTEM	1.378660218
5620	0.1599996	0.1160544	ZNF716	1.378660217
5621	0.1057731	0.0767217	ZNF99	1.378660214
5622	0.0510752	0.037047	MUC12	1.378660202
5623	28.135177	20.419662	WIZ	1.377847335
5624	16.9375	12.300479	MGC2752	1.376978932
5625	80.488325	58.486745	G6PD	1.37618062
5626	12.357725	8.9812549	PRKCD	1.375946327
5627	23.700335	17.230365	GMNN	1.37549816
5628	26.158933	19.02102	NDUFV3	1.37526451
5629	184.11636	133.87745	VCL	1.375260436
5630	5.5071734	4.0054089	NOTCH1	1.374934116
5631	50.210574	36.543709	PLEKHO1	1.3739868

5632	214.53534	156.19428	H2AFZ	1.373515971
5633	4.1981792	3.0565201	RNF19A	1.37351597
5634	8.8617083	6.4518422	MAN2A2	1.37351597
5635	7.9583606	5.7959028	APPBP2	1.373101111
5636	13.210688	9.6255457	SLC36A1	1.372461213
5637	48.843633	35.61837	ERAL1	1.371304568
5638	4.0705634	2.9686843	FIG4	1.371167506
5639	24.298071	17.724661	MPZL1	1.370862373
5640	10.729252	7.8285349	DENND3	1.370531332
5641	140.75149	102.7085	NDUFB7	1.370397707
5642	40.104872	29.266045	SDHC	1.370355042
5643	21.501931	15.692524	SKIV2L	1.370202187
5644	22.152703	16.1751	ZC3H3	1.369555864
5645	9.9566844	7.2711294	MAN2A1	1.369344953
5646	10.771704	7.8663189	PTS	1.369344952
5647	76.425252	55.829456	RNASEH2A	1.368905553
5648	3.9459924	2.8827849	FOXO3	1.368812652
5649	10.9892	8.0282716	CISD3	1.368812651
5650	324.7123	237.22474	ARPC2	1.368796073
5651	115.63388	84.619325	EID1	1.366518615
5652	25.235285	18.469112	IFI16	1.366350758
5653	51.813612	37.928647	MRPL27	1.366081208
5654	16.057978	11.761716	COPS4	1.365275173
5655	0.8260881	0.6052488	ABCC9	1.364873623
5656	2.1147551	1.5494146	ANKMY1	1.364873623
5657	23.04671	16.898844	ZBTB17	1.363803972
5658	84.072277	61.670196	NDUFA7	1.3632562
5659	19.731662	14.474982	ARL4C	1.363156261
5660	6.1858603	4.5384367	LOC100506451	1.362993631
5661	2.991874	2.1950756	ZNF821	1.362993631
5662	1.5975642	1.1720996	SLC10A7	1.362993631
5663	8.5181872	6.2556927	KIF7	1.361669694
5664	6.5933362	4.8429599	NHSL1	1.361426972
5665	181.50924	133.32498	ZYX	1.361404562
5666	58.810872	43.20298	MRPS6	1.361268869
5667	24.523888	18.021236	TRIM21	1.360832722
5668	22.154181	16.281192	DNAJC14	1.360722266
5669	363.02982	266.80449	COX6A1	1.36065861
5670	7.2613066	5.3371555	RILPL2	1.360519959
5671	39.568175	29.087253	PMAIP1	1.360326977
5672	38.372962	28.210664	MOB1A	1.360228938
5673	10.753236	7.9072077	QPCTL	1.359928428
5674	160.54346	118.06442	CDC42	1.359795421

5675	4.756622	3.4980963	RPS6KC1	1.359774468
5676	41.103449	30.238237	TBC1D10B	1.359320302
5677	32.869916	24.183013	ANTXR1	1.359215087
5678	37.288874	27.434774	SKA2	1.359182838
5679	14.004982	10.304215	CPT2	1.359150883
5680	52.313108	38.494814	MMADHC	1.358965078
5681	263.15864	193.72737	FSTL1	1.358396796
5682	45.570959	33.563056	ISCU	1.357771433
5683	7.6290072	5.6189733	CCSER2	1.357722628
5684	15.693833	11.559881	POC1A	1.357611977
5685	48.267241	35.560453	IDH3G	1.357329163
5686	1.976689	1.4565337	DGCR11	1.357118659
5687	2.8944375	2.1327815	ZRSR2	1.357118659
5688	2.4641833	1.8157464	TMEM107	1.357118658
5689	22.647671	16.691197	ASH2L	1.356863225
5690	25.624358	18.889695	GSDMD	1.356525771
5691	30.922816	22.797312	ADAM9	1.356423769
5692	14.771479	10.890844	MEF2D	1.356320822
5693	135.82458	100.18575	ATP6V0B	1.355727521
5694	8.6984139	6.4171756	GGCX	1.355489464
5695	13.703815	10.111753	ST3GAL4	1.355236386
5696	36.953044	27.277152	BTG3	1.354725151
5697	17.941094	13.24848	SLC25A46	1.354200124
5698	2.0290268	1.498497	AHI1	1.354041292
5699	17.244126	12.740274	PTPN18	1.353512989
5700	16.114297	11.907258	IRF2	1.353317206
5701	9.4069888	6.9537829	ORAI2	1.352787245
5702	35.887946	26.53356	IER3IP1	1.352549235
5703	10.068947	7.4448821	MED14	1.35246568
5704	16.311195	12.063177	PDCL3	1.352147528
5705	2.4191134	1.7890898	LYSMD4	1.352147528
5706	0.8823889	0.6525833	LINC00476	1.352147527
5707	7.3133789	5.4123316	CNKSR3	1.351243686
5708	30.060503	22.257262	SPTLC1	1.350593037
5709	211.10759	156.3097	CDC37	1.350572516
5710	438.41177	324.80747	TUBB4B	1.349758903
5711	36.041197	26.704387	SFT2D1	1.349635799
5712	204.80402	151.79616	PSMC5	1.349204269
5713	1.1063502	0.8203151	STON1	1.34868935
5714	27.604335	20.47037	SUCLG2	1.348502032
5715	7.2121271	5.3489344	SMIM13	1.3483297
5716	14.343567	10.639679	ENY2	1.348120284
5717	6.5744008	4.8775624	TUBE1	1.347886559

5718	12.817281	9.5100676	ARHGAP10	1.347759219
5719	5.9661641	4.4281484	CDPF1	1.347327038
5720	4.0121686	2.9778728	DNM1P41	1.347327038
5721	11.876264	8.8208829	ATF6	1.346380415
5722	28.941855	21.497267	LPCAT3	1.346303913
5723	31.487152	23.388341	MLX	1.346275589
5724	78.520191	58.386787	ATP6V0D1	1.344828072
5725	2.0717768	1.5412785	FUK	1.344193719
5726	5.4934644	4.08681	KCTD18	1.344193719
5727	1.0828112	0.805547	ARRDC3-AS1	1.344193719
5728	2.1630287	1.6091644	GLB1L	1.344193719
5729	4.0955306	3.0468306	PMS2P5	1.344193719
5730	5.1860519	3.8581135	JMJD4	1.344193719
5731	1.2944068	0.9629615	LOC100507501	1.344193718
5732	31.632799	23.539013	BLMH	1.343845573
5733	30.166393	22.448232	FPGS	1.343820436
5734	87.94063	65.450635	VPS4A	1.343617677
5735	46.625304	34.715473	SEC31A	1.343069811
5736	26.805008	19.960053	HIATL1	1.342932749
5737	10.274193	7.6537157	NMI	1.342379693
5738	72.292598	53.882752	TRIM8	1.341664927
5739	19.344861	14.422555	TBC1D16	1.341292202
5740	15.123703	11.279738	LYRM4	1.340784944
5741	3.3752811	2.5181823	PTOV1-AS1	1.340364108
5742	17.013234	12.69439	PPIL2	1.340216814
5743	7.0174016	5.2366412	ITGA1	1.340057738
5744	22.730601	16.965917	SH3PXD2B	1.339780325
5745	25.317375	18.907863	CERK	1.338986549
5746	3.0801656	2.3007745	PRICKLE2	1.338751639
5747	90.664525	67.736699	BRMS1	1.338484543
5748	1.8171974	1.3580315	ZNF416	1.338111395
5749	1.6315975	1.2193286	SH3RF3-AS1	1.338111394
5750	7.0256202	5.2539913	PHTF2	1.337196759
5751	2.8228215	2.111211	EIF5A2	1.337062718
5752	18.344342	13.729076	ARL1	1.336167272
5753	3.5940943	2.6910422	PROS1	1.335577093
5754	39.048664	29.237297	RCE1	1.335577093
5755	3.7022261	2.7720048	PLA2G12A	1.335577093
5756	5.3568694	4.0109024	LIME1	1.335577092
5757	12.835201	9.6102285	NFKBIL1	1.335577092
5758	178.26711	133.48785	DDOST	1.335455732
5759	8.6358627	6.4704568	RFX1	1.33466043
5760	10.908113	8.1758483	PLEKHG2	1.334187314

5761	34.912905	26.178054	ARHGAP17	1.333670759
5762	9.4422653	7.0809441	XYLT1	1.333475476
5763	7.050629	5.2897528	RAB8B	1.332884397
5764	26.003668	19.510404	CPNE2	1.332810286
5765	31.500181	23.637265	CD2BP2	1.332649113
5766	18.47334	13.866136	SPIN1	1.332263006
5767	19.022843	14.278594	HAUS8	1.332263005
5768	42.614427	31.994591	SPNS1	1.33192598
5769	6445.3059	4839.216	ACTB	1.331890509
5770	27.259282	20.483533	CSRNP1	1.330790078
5771	33.076565	24.861988	MVB12A	1.330407117
5772	30.735817	23.110133	SNHG3	1.32997148
5773	54.340917	40.860651	MAP1B	1.329908259
5774	0.9248499	0.695678	PRSS53	1.32942236
5775	0.8018792	0.6031787	A2M	1.32942236
5776	1.1457517	0.8618417	NMUR1	1.32942236
5777	11.504287	8.6535983	SUDS3	1.32942236
5778	1.4960879	1.1253669	PRRT3	1.32942236
5779	3.0398631	2.2866044	TYMP	1.32942236
5780	1.3650622	1.0268085	CEMP1	1.329422359
5781	8.9068519	6.699791	HSPA13	1.329422359
5782	2.3136458	1.7403391	ATG10	1.329422359
5783	29.977552	22.549306	UBAC1	1.329422359
5784	0.6931247	0.5213728	TMEM117	1.329422359
5785	2.5763674	1.9379601	C2orf48	1.329422359
5786	0.5181203	0.3897334	TEC	1.329422359
5787	0.9778913	0.7355761	NR6A1	1.329422359
5788	1.1408732	0.8581721	IDNK	1.329422359
5789	0.2578847	0.1939825	ZC3H12B	1.329422357
5790	9.6604926	7.2700222	STAMBP	1.328811973
5791	21.821937	16.43135	RUFY1	1.328067189
5792	6.7038503	5.0496058	SHOX2	1.327598735
5793	140.50853	105.86457	H2AFY	1.327247969
5794	151.79717	114.45927	COPE	1.326211195
5795	51.650922	38.953236	HEXA	1.325972573
5796	7.3900532	5.5747277	C16orf87	1.325634831
5797	45.691869	34.489471	LINC00493	1.32480631
5798	3.463835	2.6157166	PEX2	1.324239426
5799	10.827287	8.177597	CCNE1	1.324018203
5800	15.21551	11.494011	CCDC3	1.323777253
5801	8.749235	6.6101329	ENAH	1.323609556
5802	87.202628	65.883145	ARPC1B	1.323595393
5803	16.506891	12.47202	LIMD2	1.323513816

5804	6.9264479	5.2333778	CNNM4	1.323513816
5805	14.973184	11.321434	ACVR1	1.32255196
5806	20.528906	15.522192	GLE1	1.32255196
5807	2.6343306	1.9918542	IRAK4	1.32255196
5808	13.62356	10.302765	TOR4A	1.322320745
5809	56.617501	42.824586	PPP1R9B	1.322079372
5810	24.017002	18.16735	TCF19	1.321987112
5811	23.94579	18.114084	TAB2	1.32194319
5812	27.549215	20.843527	PRCP	1.321715563
5813	9.6442425	7.2995196	STARD10	1.321216049
5814	5.1045671	3.863537	ZNF764	1.321216049
5815	3.0725567	2.3255521	LOC220729	1.321216048
5816	93.589332	70.866258	VASP	1.320647294
5817	11.724176	8.8775982	ZFYVE19	1.320647294
5818	4.4511473	3.3712326	TRIM59	1.320332292
5819	69.250363	52.487725	ST13	1.319363011
5820	7.3362593	5.5618066	ATF7	1.319042485
5821	8.5409631	6.4759636	S1PR1	1.318871388
5822	6.3469611	4.8134784	TRAPPC11	1.318580995
5823	13.722885	10.409185	TSC22D1	1.31834384
5824	3.0323465	2.3001181	RNF182	1.31834384
5825	1.2401325	0.9410364	KLF12	1.31783698
5826	6.2545911	4.74675	LEMD3	1.31765756
5827	31.643705	24.02047	BRD9	1.317364107
5828	106.81291	81.082467	PSMC4	1.317336702
5829	21.117816	16.034251	FOPNL	1.317044125
5830	11.16359	8.477835	RNF41	1.316797266
5831	34.73325	26.384662	CMTM6	1.316418228
5832	0.4605514	0.3499647	HDX	1.315993853
5833	0.7299457	0.5546726	PDGFD	1.315993851
5834	3.6025226	2.737492	SERINC5	1.315993851
5835	8.0109638	6.0873869	TVP23B	1.315993851
5836	12.730158	9.6734173	CDC42SE2	1.31599385
5837	21.704567	16.508303	PPP3CA	1.314766702
5838	44.11544	33.554272	KIFC1	1.314748823
5839	16.100309	12.246831	WWP2	1.314651
5840	7.0805709	5.385894	ULK1	1.314651
5841	12.960168	9.8601351	PRKAB1	1.314400638
5842	11.210202	8.5311258	APOO	1.314035526
5843	27.187683	20.692093	EIF2B4	1.313916513
5844	5.0796196	3.8673773	PARP14	1.313453322
5845	8.824123	6.7182616	TMEM183A	1.313453322
5846	0.9103798	0.6931193	PAG1	1.313453322

5847	93.335163	71.062266	YARS	1.313427894
5848	3.1107305	2.3691603	REV3L	1.313009738
5849	39.813201	30.323854	STYXL1	1.3129334
5850	518.90077	395.25021	RPS7	1.31284123
5851	3.9456016	3.006451	LOC100505783	1.312378483
5852	0.8946512	0.6817021	TRDMT1	1.312378483
5853	15.651069	11.933341	FAM189B	1.311541241
5854	50.93279	38.839347	EHD1	1.311370921
5855	36.021887	27.475893	DHX16	1.311036068
5856	38.855999	29.639389	CCM2	1.31095816
5857	5.4895811	4.1879216	JARID2	1.310812773
5858	22.2204	16.951802	WDR26	1.310798711
5859	3.5739244	2.7267335	LGR4	1.310698101
5860	9.6195947	7.3409061	SLC38A7	1.310409718
5861	16.267545	12.416998	ESYT2	1.310102871
5862	25.707925	19.625556	TMEM8A	1.309920845
5863	2.42325	1.8501944	ZNF786	1.309727214
5864	1.8864176	1.4403133	ZNF79	1.309727213
5865	3.7179254	2.8387021	PHKA2	1.309727213
5866	3.4978715	2.670687	HHIP-AS1	1.309727213
5867	5.2423477	4.0059199	NRG1	1.308650135
5868	23.674047	18.094992	SOX4	1.308320417
5869	9.0792082	6.9428843	MED30	1.307699772
5870	12.76595	9.7696311	TMEM194A	1.306697191
5871	10.139143	7.7599801	LOC100129034	1.306593895
5872	119.00209	91.112603	PPP4C	1.306099161
5873	3.3101178	2.5343541	DPY19L3	1.30609916
5874	48.239263	36.933844	PI4KB	1.30609916
5875	5849.0793	4479.7368	GAPDH	1.305674763
5876	7.0778257	5.4213386	KIDINS220	1.305549455
5877	0.01	0.0076614	NEB	1.305248391
5878	19.445728	14.903186	ITGB3BP	1.304803427
5879	24.490407	18.769423	LRRC42	1.304803427
5880	6.3453697	4.8630848	MTMR6	1.304803427
5881	8.5813428	6.5778922	SRGAP2	1.304573343
5882	4.9275055	3.7795357	LOC100190986	1.303733039
5883	49.836612	38.229076	CDK2	1.303631096
5884	196.96469	151.09177	EIF3I	1.303609645
5885	83.600219	64.152881	FHL2	1.303140534
5886	19.415497	14.908502	UBA3	1.302310371
5887	42.209089	32.413378	NT5C3B	1.30221196
5888	12.899326	9.9061183	CIT	1.302157467
5889	3.3359619	2.5620489	AMN1	1.30206799

5890	10.759757	8.265055	ARHGAP31	1.30183729
5891	104.38909	80.20408	DDX39A	1.301543385
5892	5.5978715	4.3054849	CEP120	1.300172142
5893	4.0935616	3.1491851	TMEM79	1.29987964
5894	11.369595	8.7466518	RPA3	1.29987964
5895	17.736128	13.644438	BAG2	1.29987964
5896	13.191467	10.150338	ZNF532	1.299608607
5897	2.9192612	2.2471029	DZIP1L	1.299122135
5898	21.625531	16.646264	MED25	1.299122134
5899	19.424282	14.964848	ZDHHC18	1.297993935
5900	20.04168	15.440503	MID1IP1	1.297993935
5901	19.176892	14.775558	LEPREL4	1.297879352
5902	33.438334	25.776058	POLR2F	1.297263311
5903	2.5518306	1.9671928	TTBK2	1.297193939
5904	124.48258	95.975165	BRK1	1.297029027
5905	64.683049	49.872179	DLGAP4	1.296976599
5906	24.905826	19.209371	MOB3A	1.296545666
5907	31.504209	24.317717	SBNO2	1.295524935
5908	77.237489	59.62143	DVL1	1.295465211
5909	31.900618	24.629593	ATP13A1	1.295215
5910	6.9709269	5.3833299	NDST2	1.294909837
5911	15.872619	12.258527	DHRS3	1.294822779
5912	8.069782	6.2334386	NEDD1	1.294595577
5913	8.8740012	6.8550018	TRIM32	1.294529384
5914	20.178364	15.595964	BTBD10	1.293819596
5915	15.417414	11.920554	EHMT1	1.293347092
5916	0.4164658	0.3222188	PCED1B-AS1	1.292493963
5917	0.119208	0.092231	GVINP1	1.292493963
5918	0.2608254	0.2018001	POM121L1P	1.292493962
5919	0.3879365	0.3001457	LOC387723	1.292493962
5920	0.8433812	0.6525224	MASP2	1.292493962
5921	0.6757442	0.5228219	ZHX1-C8ORF76	1.292493961
5922	0.8670668	0.6708478	SYTL2	1.292493961
5923	0.8558501	0.6621695	ZNF85	1.292493961
5924	1.5763956	1.2196541	LINC00526	1.292493961
5925	41.314687	31.965091	TUBGCP2	1.292493961
5926	0.6079332	0.4703567	LOC100132707	1.292493961
5927	0.4992801	0.386292	SLC25A18	1.292493961
5928	0.7960256	0.6158834	ECEL1P2	1.292493961
5929	29.304419	22.67277	GPN1	1.292493961
5930	2.2825794	1.7660271	C17orf49	1.292493961
5931	5.141435	3.977918	RGS16	1.292493961
5932	57.395956	44.407137	PEF1	1.292493961

5933	3.3685264	2.6062222	BCKDHB	1.29249396
5934	7.8394796	6.0653898	C16orf55	1.29249396
5935	0.5445506	0.4213177	LOC100132247	1.29249396
5936	7.4817053	5.7885805	INIP	1.29249396
5937	1.519833	1.1758918	CENPP	1.29249396
5938	5.4973489	4.2532879	CXorf40A	1.29249396
5939	3.3204776	2.5690469	PPM1K	1.29249396
5940	1.0698126	0.8277119	PLEKHM1P	1.29249396
5941	2.1396253	1.6554238	HIST1H2AM	1.29249396
5942	0.7594734	0.5876031	HS3ST5	1.29249396
5943	11.036734	8.5390988	MED19	1.29249396
5944	0.3772619	0.2918868	CCDC121	1.29249396
5945	0.4387358	0.339449	MTHFS	1.29249396
5946	0.861155	0.6662739	C7orf55	1.29249396
5947	0.4641414	0.3591053	CYP26A1	1.29249396
5948	1.0764437	0.8328423	LOC400684	1.29249396
5949	0.5873718	0.4544484	PAXBP1-AS1	1.29249396
5950	0.6104262	0.4722855	FAM151B	1.29249396
5951	0.4540294	0.3512817	FAM172BP	1.292493959
5952	0.6037065	0.4670866	LOC100130451	1.292493959
5953	0.8520012	0.6591917	ZNF182	1.292493959
5954	0.5134257	0.3972365	TMEM254-AS1	1.292493959
5955	0.6136617	0.4747888	LOC100288123	1.292493959
5956	0.1401288	0.1084173	MYO16	1.292493959
5957	68.741729	53.21769	PDZD11	1.29170825
5958	62.107	48.086782	DGUOK	1.291560752
5959	256.32343	198.47078	TNFRSF12A	1.291492027
5960	65.810368	50.976283	TIMM10	1.290999748
5961	158.76988	122.99921	MAZ	1.290820337
5962	5.480722	4.2468098	DUSP16	1.290550361
5963	11.872456	9.2019825	C6orf47	1.29020636
5964	29.643032	22.982141	FAM3C	1.289829024
5965	14.934166	11.578408	KIAA0146	1.289829024
5966	42.13096	32.670892	SRF	1.289556474
5967	4.0441383	3.13709	UGGT2	1.289136833
5968	11.032202	8.5590418	RAB7L1	1.288952881
5969	16.083479	12.47993	DHDDS	1.288747601
5970	7.0265933	5.452738	ZNF865	1.28863577
5971	1.4623176	1.1351139	DBT	1.288256276
5972	41.724765	32.398075	TAF6	1.287877911
5973	2.0064722	1.5582839	SAMD9	1.287616625
5974	42.554718	33.05356	FBRS	1.287447347
5975	69.4665	53.959631	NPTN	1.287379068

5976	16.441952	12.773241	ZDHHC9	1.287218474
5977	25.643909	19.923311	B4GALT3	1.287130915
5978	16.406506	12.747166	ITFG1	1.287070909
5979	17.688787	13.744955	DLG5	1.286929407
5980	258.90989	201.20624	COX6B1	1.286788555
5981	4.9894707	3.8775772	PRELID2	1.286749543
5982	35.729137	27.786054	TNPO2	1.285865787
5983	3.1013952	2.4133338	SOCS6	1.285108281
5984	3.2568628	2.535193	LZTS3	1.284660664
5985	9.8263026	7.6553865	SERTAD3	1.283580209
5986	6.8144297	5.3116563	SLC39A8	1.282919931
5987	26.536802	20.697047	SLC41A1	1.282154009
5988	27.811321	21.696877	MFAP2	1.281812193
5989	24.638654	19.222114	NEO1	1.28178691
5990	36.84149	28.748858	ETFB	1.281494012
5991	36.575759	28.54882	ARGLU1	1.281165328
5992	47.518679	37.095229	PAF1	1.280991672
5993	9.3371976	7.2894498	GLIS2	1.280919388
5994	0.6339519	0.4952036	ZNF555	1.280184492
5995	38.452626	30.049555	TSR3	1.279640429
5996	7.1761915	5.6082879	SLC35F5	1.279569021
5997	162.68529	127.20832	DSTN	1.278888761
5998	1.8508648	1.4472446	ZNF776	1.278888761
5999	250.45044	195.86623	ATF4	1.278681048
6000	144.66567	113.14354	WARS	1.278603044
6001	58.997662	46.143543	TBC1D9B	1.278568099
6002	18.05035	14.118747	ELMO2	1.278466894
6003	24.760337	19.368314	ST6GALNAC4	1.278394026
6004	26.239445	20.526977	STC2	1.27829073
6005	14.914733	11.67063	PIGC	1.277971556
6006	9.2393867	7.2309201	DCHS1	1.277760869
6007	116.08661	90.867749	SRP9	1.277533735
6008	106.70054	83.521428	AIP	1.277522988
6009	1.0003176	0.7831573	ZNF41	1.277288149
6010	3.5493832	2.778843	RRM2B	1.277288149
6011	3.7479785	2.9343249	ARHGAP18	1.277288149
6012	18.642767	14.595584	PCGF1	1.277288149
6013	8.873382	6.9499258	SLC37A3	1.276759251
6014	76.292447	59.776024	RBM42	1.276305147
6015	13.747599	10.772855	RAB2B	1.276133277
6016	36.675871	28.741962	RANBP3	1.276039212
6017	3.6997769	2.8995252	GAS8	1.275994038
6018	22.063097	17.294782	NAA60	1.275708325

6019	43.068559	33.769726	PATL1	1.275360035
6020	3.2330321	2.535193	TBXAS1	1.275260708
6021	2.9274038	2.2955336	FAM171A2	1.275260708
6022	132.19928	103.67468	HDLBP	1.275135603
6023	50.481867	39.603981	PYCR2	1.274666458
6024	4.8001623	3.7664309	LOC550112	1.274459161
6025	10.337083	8.1109569	TMEM206	1.274459161
6026	47.204762	37.054505	HP1BP3	1.273927749
6027	16.609411	13.038687	UBFD1	1.273856117
6028	42.069432	33.02622	SBDS	1.273819143
6029	4.3057748	3.3803601	RFX2	1.273762164
6030	30.497488	23.944709	THAP7	1.273662923
6031	32.441144	25.471333	PRRC2B	1.273633534
6032	40.806369	32.042487	ATXN10	1.273508174
6033	1.1219354	0.8816023	FLJ10038	1.272609439
6034	11.341469	8.9119796	TMEM60	1.272609438
6035	5.2045661	4.0896806	RNF215	1.272609438
6036	0.78697	0.6183908	ZBTB41	1.272609438
6037	69.746607	54.811623	PPP5C	1.272478403
6038	12.002756	9.4395827	WRB	1.271534599
6039	55.160183	43.384762	SEC14L1	1.271418352
6040	2.877503	2.2640526	ADM2	1.270952395
6041	3.3853443	2.6636279	RASAL2	1.270952394
6042	8.5165944	6.7016875	CDS2	1.270813417
6043	6.7580532	5.3198904	HYI	1.270336921
6044	11.823075	9.3088694	MECP2	1.270087089
6045	4.0897014	3.2206973	CDH24	1.269818628
6046	194.767	153.39648	ARF4	1.269696608
6047	19.005822	14.974697	CCNL2	1.269195786
6048	7.428101	5.8535348	MEF2BNB	1.26899407
6049	73.552765	57.975948	SUPT4H1	1.268677218
6050	20.86865	16.455313	OSBP	1.268201303
6051	39.811644	31.404467	CDIPT	1.267706405
6052	2.3849363	1.8816393	PDE7A	1.267477948
6053	52.895293	41.732713	TMED4	1.267477948
6054	16.634698	13.127638	SP2	1.267150942
6055	80.402917	63.46113	ELOF1	1.266963216
6056	13.931208	10.998518	TMEM199	1.266644082
6057	2.725267	2.151565	LPL	1.266644081
6058	10.31705	8.1529233	RBMS2	1.265441761
6059	9.0210287	7.130126	USP33	1.265199057
6060	9.8419554	7.7808951	SP140L	1.264887293
6061	79.907615	63.20037	YWHAG	1.264353586

6062	0.855052	0.6765873	ZNF30	1.263771874
6063	1.4490484	1.146606	RSG1	1.263771873
6064	14.780106	11.695233	AVEN	1.263771873
6065	17.155431	13.574785	TOR3A	1.263771873
6066	2.9439998	2.3295342	GPATCH2	1.263771873
6067	44.2901	35.045961	MRPS11	1.263771873
6068	1.77705	1.4061478	ZFX	1.263771872
6069	1.6429991	1.3000757	G2E3	1.263771872
6070	0.5983802	0.4734875	DNM1P46	1.263771872
6071	20.762143	16.435912	CNDP2	1.263218194
6072	12.498157	9.8946781	SEMA4C	1.263119097
6073	146.52178	116.01704	USMG5	1.262933271
6074	12.36961	9.7972261	POM121C	1.262562522
6075	9.3710248	7.4257547	NPR2	1.261962607
6076	7.5400824	5.9755392	PRICKLE3	1.261824612
6077	29.066798	23.036939	CERS5	1.261747408
6078	18.222179	14.442328	LIMS1	1.261720295
6079	8.1646561	6.4723137	STAP2	1.261474106
6080	24.294085	19.263966	TMUB1	1.261115495
6081	73.856576	58.566258	MRPL38	1.261077263
6082	7.4218628	5.8868702	GNG2	1.260748495
6083	12.885055	10.220888	TMEM245	1.260659133
6084	46.361012	36.78357	CYSTM1	1.260372809
6085	19.501926	15.477657	PITX1	1.260005041
6086	2.6459667	2.1006503	IFT140	1.259594114
6087	13.943789	11.072637	RYBP	1.259301508
6088	41.545593	32.992365	DPYSL2	1.259248709
6089	21.160712	16.807229	MAPK14	1.25902442
6090	27.528032	21.865608	BAK1	1.258964828
6091	3.7309373	2.9639389	POMZP3	1.258776727
6092	0.1953135	0.155186	GRIA3	1.258576592
6093	35.797871	28.443215	RNF25	1.258573305
6094	61.738866	49.055235	SRSF4	1.258558139
6095	16.910258	13.437716	SH3PXD2A	1.258417555
6096	16.345059	12.990881	PDE4A	1.258194769
6097	20.99426	16.690927	BCL2L13	1.257824711
6098	4.4173234	3.5126097	MORC3	1.257561692
6099	129.59189	103.11173	RTN4	1.25681034
6100	5.7535166	4.5795138	FAM167A	1.256359721
6101	20.913982	16.646492	DCTN6	1.25635972
6102	109.96195	87.538639	MEA1	1.256153281
6103	0.488055	0.3887133	FOXO3B	1.255565563
6104	1.7223099	1.3717403	ZNF77	1.255565562

6105	2.5341856	2.0183618	TAX1BP3	1.255565561
6106	36.7003	29.239292	MPG	1.255170606
6107	62.144838	49.517264	POLDIP2	1.255013568
6108	21.185364	16.881024	TTC17	1.254980943
6109	59.602677	47.494659	ARL2	1.254934307
6110	7.1899273	5.730556	DTX3L	1.254664869
6111	9.9341826	7.9183282	UBXN2A	1.254580804
6112	2.7781594	2.2148115	DEPDC5	1.254354795
6113	78.791566	62.825372	HSBP1	1.254136075
6114	31.89896	25.436094	ZDHHC16	1.254082494
6115	225.97536	180.21361	ATP5C1	1.253930618
6116	10.422051	8.3124934	FOXRED2	1.253781581
6117	26.390503	21.053768	TACC1	1.25348126
6118	47.925829	38.234689	AP1M1	1.253464602
6119	1.9225046	1.5339204	NOMO3	1.253327477
6120	1.6759107	1.3371691	DTWD2	1.253327477
6121	3.0826207	2.4612826	C7orf41	1.252444852
6122	15.038587	12.007987	KRT80	1.252382079
6123	10.962208	8.7542994	AKT3	1.252208435
6124	64.201815	51.279644	SLC9A3R1	1.25199415
6125	25.360786	20.261424	CENPW	1.251678362
6126	4.5261459	3.6160615	H3F3AP4	1.251678362
6127	2.2366722	1.7869384	FAM86FP	1.251678362
6128	9.8482743	7.8698098	SUFU	1.251399281
6129	7.8984152	6.3118187	CAMKK2	1.251369153
6130	12.793549	10.224336	ANKFY1	1.251284008
6131	38.33727	30.642014	FOXK2	1.251134154
6132	2.9406985	2.350666	AMOT	1.2510065
6133	38.000647	30.378226	ATG9A	1.250917232
6134	3.0118436	2.4079327	MON2	1.250800607
6135	23.760198	18.999415	ZDHHC4	1.250575238
6136	125.85	100.65148	GNG5	1.250354255
6137	5.5146552	4.4115341	CENPBD1	1.25005386
6138	1.5313762	1.2250482	SHROOM3	1.25005386
6139	36.602015	29.291122	ACBD6	1.249594161
6140	14.321293	11.462437	TMEM171	1.249410828
6141	2.2309286	1.7855845	EPOR	1.249410828
6142	17.285539	13.83877	PHAX	1.249066164
6143	14.064698	11.262108	CUTC	1.248851307
6144	29.395076	23.537691	BRE	1.248851307
6145	21.945179	17.575054	VPS18	1.248654984
6146	63.045227	50.528102	PRCC	1.247726013
6147	514.8209	412.65528	TUBA1B	1.247581043

6148	1.7662944	1.416027	BTBD9	1.247359251
6149	22.68756	18.197117	MESDC1	1.246766737
6150	16.427458	13.177892	VTA1	1.246592306
6151	221.37532	177.65365	GNB1	1.2461062
6152	69.127087	55.474896	DNM2	1.246096741
6153	31.291487	25.11717	NUP85	1.245820567
6154	28.187857	22.651744	MYO9B	1.244401162
6155	12.184329	9.7942766	RTCA	1.244025437
6156	17.043577	13.703702	GBE1	1.243720604
6157	3.1641286	2.5447147	KIF3A	1.243411911
6158	19.364555	15.574731	AGPAT6	1.243331614
6159	152.52427	122.6813	MRPL51	1.243256095
6160	6.3489977	5.1086951	TRPM4	1.242782654
6161	27.332258	21.993345	FAM104A	1.242751291
6162	13.634542	10.972519	MUM1	1.242608229
6163	6.5549425	5.2790183	IRS1	1.241697255
6164	26.330769	21.206414	IP6K2	1.241641739
6165	96.899131	78.05165	FIS1	1.241474462
6166	0.3726966	0.3003694	GCSAM	1.240794203
6167	0.2292203	0.1847368	SLC6A20	1.240794203
6168	0.5317444	0.4285517	ZNF417	1.240794203
6169	2.3908164	1.9268437	RPL23P8	1.240794202
6170	0.6654588	0.5363168	MAP2K6	1.240794202
6171	19.877621	16.020079	PANK4	1.240794202
6172	0.442932	0.3569746	DPPA4	1.240794202
6173	21.470431	17.303781	TFPT	1.240794202
6174	4.0353138	3.2522023	PCNXL2	1.240794202
6175	5.1883693	4.1814906	LINC00869	1.240794202
6176	3.5314276	2.8461026	GANC	1.240794202
6177	7.3408826	5.9162773	EPS8	1.240794202
6178	0.8805613	0.7096755	LOC728407	1.240794202
6179	11.524396	9.2879192	SNORD3A	1.240794202
6180	0.8220888	0.6625505	LOC338799	1.240794202
6181	1.0035289	0.8087795	PRDM5	1.240794202
6182	0.9791676	0.7891458	LINC00260	1.240794201
6183	0.1346685	0.1085341	PDE11A	1.240794201
6184	15.948212	12.864756	ZNF706	1.23968238
6185	18.590904	14.997686	TRAF2	1.239584851
6186	7.1505884	5.769076	LRRC14	1.239468567
6187	17.430173	14.065153	LRRFIP2	1.239245146
6188	22.047656	17.791623	HMGN3	1.239215584
6189	6.2863565	5.0736871	MAMLD1	1.239011452
6190	157.20811	126.97973	TALDO1	1.23805679

6191	18.709706	15.1127	CCHCR1	1.238012152
6192	9.9023521	7.9987942	EIF4ENIF1	1.23798061
6193	25.190147	20.353029	FBXW11	1.237660883
6194	17.855826	14.430069	FDX1L	1.237404054
6195	18.992714	15.348837	POP5	1.237404054
6196	8.8795946	7.1765387	RABGAP1	1.237308825
6197	19.434674	15.711286	SNX2	1.236988085
6198	2.8013155	2.2650816	SIRT5	1.236739319
6199	61.133219	49.432571	SLC35B2	1.236699172
6200	36.517133	29.531935	RAB1A	1.236530304
6201	4.8243382	3.9025055	SZT2	1.236215626
6202	205.49472	166.25176	CAP1	1.236045351
6203	5.2555715	4.2529396	CDKN1C	1.235750323
6204	8.4127925	6.8096464	PQLC2	1.235422799
6205	66.393943	53.746093	NDUFB4	1.235325958
6206	21.725044	17.586801	RAD51C	1.235303963
6207	15.670142	12.693888	TXNDC9	1.234463619
6208	58.35186	47.278342	HES1	1.234219666
6209	121.73088	98.637535	TGFB1	1.234123266
6210	5.7823633	4.6854018	ANKS3	1.234123266
6211	12.486133	10.12052	SNX14	1.233744236
6212	47.486865	38.49004	MAP1LC3B	1.233744235
6213	7.6497522	6.2014722	PILRB	1.233538096
6214	6.7062815	5.4396997	ACOX3	1.232840393
6215	8.5341935	6.9223831	CABLES2	1.232840393
6216	18.440679	14.95788	ZMAT5	1.232840393
6217	22.696975	18.420215	ABCF3	1.232177576
6218	24.466189	19.859012	TMEM223	1.231994244
6219	5.9940277	4.865305	SLC38A6	1.231994243
6220	13.480043	10.943344	MCOLN1	1.23180294
6221	159.73898	129.72487	UQCRC1	1.231367466
6222	105.8307	85.964538	CTSA	1.231097204
6223	1.450442	1.1783143	TOB2P1	1.23094663
6224	3.1914165	2.5926522	ZNF785	1.230946629
6225	1.2337171	1.0022507	ZNF365	1.230946629
6226	5.7499514	4.6730315	ZNF516	1.23045425
6227	41.863917	34.036226	RBX1	1.229981181
6228	57.959779	47.124002	DRG1	1.229941775
6229	27.59909	22.44143	SMG9	1.229827587
6230	25.693603	20.894809	OSMR	1.229664393
6231	12.287514	9.9947585	PCYOX1L	1.229395831
6232	106.62498	86.742111	GHITM	1.229218166
6233	18.626736	15.155973	TIPRL	1.229003029

6234	100.58271	81.88406	SART1	1.228355162
6235	0.8446225	0.6878766	RHBDL3	1.227869264
6236	2.3020875	1.8748637	C12orf73	1.227869263
6237	26.018815	21.190216	NCAPH2	1.227869263
6238	6.3727315	5.1900733	CLP1	1.227869263
6239	2.6681877	2.1730226	LOC100506083	1.227869263
6240	1.2961016	1.0555697	ULK2	1.227869262
6241	1.6436656	1.3386324	FLJ14186	1.227869262
6242	28.77194	23.442445	EIF2D	1.227343859
6243	211.70924	172.51878	PHB	1.22716637
6244	12.938328	10.546628	CAMLG	1.226773929
6245	10.58603	8.6320293	C11orf49	1.226366363
6246	63.253511	51.58551	MPV17	1.226187579
6247	26.935648	21.967598	NXF1	1.226153563
6248	44.102555	35.970062	CBX1	1.226090601
6249	37.587739	30.662078	TXNDC12	1.225870561
6250	5.2170856	4.2561194	CAMK2D	1.225784595
6251	1.315342	1.0733317	ERO1LB	1.225475756
6252	2.7230641	2.2220465	ATP2A3	1.225475755
6253	2.6771513	2.1845812	ACTR6	1.225475755
6254	10.64264	8.6867779	C17orf53	1.22515394
6255	21.123873	17.244148	PIAS4	1.224987907
6256	15.422164	12.590687	PEX11B	1.224886585
6257	8.5778472	7.0038557	SPHK2	1.224732141
6258	2.2990874	1.8776215	DFNB31	1.224467963
6259	26.566509	21.696369	CLCN7	1.224467962
6260	1.8853996	1.5397704	ADAMTS16	1.224467962
6261	7.1300563	5.8250823	ROCK2	1.224026713
6262	2.3287103	1.9026769	FBXL20	1.223912648
6263	17.344216	14.173365	NDUFA5	1.223719052
6264	103.75331	84.798623	MTCH1	1.223525851
6265	13.344573	10.907295	FAM20B	1.223453911
6266	27.687612	22.638233	WBSCR16	1.223046524
6267	15.371853	12.573627	TSKU	1.222547229
6268	15.858762	12.972573	ATG13	1.222483871
6269	96.602685	79.041404	ATP5J	1.222178242
6270	17.209554	14.081138	MLLT4	1.222170649
6271	1.8213066	1.4904379	ZNF766	1.22199429
6272	16.749264	13.7065	C1D	1.22199429
6273	9.8373039	8.0502045	MRPL1	1.22199429
6274	15.065024	12.3357	PHACTR4	1.221254136
6275	11.105087	9.0943378	SELO	1.221099057
6276	11.469056	9.3955617	ZNF346	1.220688741

6277	8.1015127	6.6368374	PDP1	1.220688741
6278	58.616537	48.027598	RER1	1.220476127
6279	18.763773	15.374429	TMEM189	1.220453313
6280	84.445623	69.206951	CIZ1	1.220189911
6281	5.7179835	4.6864327	OCLN	1.220114299
6282	23.016726	18.866351	PDHA1	1.219988202
6283	79.066142	64.819408	CLPP	1.219791175
6284	16.198573	13.280551	PRKCA	1.219721416
6285	21.816543	17.89322	VEZF1	1.219263068
6286	9.0908672	7.4564913	FAM110A	1.219188333
6287	6.1961094	5.0828366	SGMS1	1.219025883
6288	218.74811	179.46041	NDUFS5	1.218921227
6289	10.745977	8.8162587	FOXC1	1.218881721
6290	12.763531	10.476754	NAT9	1.218271535
6291	2.9886166	2.4531613	CASK	1.218271535
6292	7.1746826	5.8899091	ZNF174	1.218131294
6293	19.899258	16.34577	PDXDC1	1.217394923
6294	19.245549	15.809201	RIPK2	1.21736377
6295	7.7125241	6.3354309	CEP104	1.21736377
6296	11.78312	9.6826386	CLASP1	1.216932775
6297	7.467242	6.1361171	STX7	1.216932775
6298	1.7102954	1.4059554	CHRNB1	1.216464904
6299	5.7121837	4.6957242	TMEM70	1.216464904
6300	7.0795613	5.8237176	HOXA1	1.215642968
6301	79.65492	65.540374	ECHS1	1.215356508
6302	17.140574	14.105688	KANSL3	1.215153352
6303	1.5571982	1.2817033	KLHL15	1.214944323
6304	101.93743	83.913918	SF3B14	1.214785735
6305	584.50706	481.16561	CLIC1	1.214773132
6306	60.868041	50.113417	NPC2	1.214605678
6307	7.2130791	5.9389684	ALKBH4	1.214534007
6308	419.48226	345.42821	RPS26	1.214383307
6309	97.8993	80.619139	EMC7	1.214343163
6310	9.3216783	7.676313	OGG1	1.214343163
6311	12.987464	10.695052	ERP44	1.214343163
6312	6.8639869	5.6532758	SIK1	1.214160993
6313	30.702111	25.290586	SYPL1	1.21397385
6314	4.2085165	3.4671657	KLC4	1.213820415
6315	17.727055	14.610893	METTL13	1.213276589
6316	40.55968	33.435648	NSMCE1	1.213066957
6317	3.9223602	3.2337205	ZFPM1	1.212955871
6318	2.332117	1.9226725	C21orf91	1.212955871
6319	221.72253	182.80602	H3F3B	1.2128842

6320	172.62944	142.33426	EIF4H	1.212845278
6321	25.74691	21.23542	DLC1	1.212451187
6322	9.7822131	8.069334	ZNF579	1.212270198
6323	4.8103075	3.968016	ZNF771	1.212270197
6324	2.6184712	2.1599732	MFSD9	1.212270197
6325	19.477862	16.071136	COQ5	1.211977944
6326	12.126604	10.005631	RNF157	1.211977943
6327	42.607101	35.157004	KIAA2013	1.211909317
6328	3.034355	2.504186	ZNF271	1.211713088
6329	22.069212	18.215087	HSD17B4	1.211589758
6330	84.550654	69.79314	STRA13	1.211446484
6331	7.3563319	6.0733316	C11orf80	1.211251483
6332	10.747647	8.8731757	DOM3Z	1.211251483
6333	11.023454	9.1056681	AKAP13	1.210614575
6334	8.8133709	7.280583	FAM92A1	1.210530929
6335	7.2251181	5.9685531	C19orf54	1.210530929
6336	72.011377	59.506986	LONP1	1.210133147
6337	5.6996382	4.7104668	LOC96610	1.209994346
6338	22.611717	18.692904	SCMH1	1.209641784
6339	3.2644692	2.6988469	SPAG1	1.209579254
6340	5.6869582	4.7016003	ZNF641	1.209579254
6341	4.6422862	3.8405521	GULP1	1.208754915
6342	3.337931	2.7614622	IGF2	1.208754915
6343	5.0612798	4.1884004	VPS54	1.208403992
6344	30.819644	25.51531	NF2	1.207888276
6345	7.7911594	6.4508321	DGAT1	1.207775869
6346	54.330451	44.993862	C16orf80	1.207508056
6347	11.08237	9.1780652	GOLT1B	1.207484291
6348	56.655055	46.945229	FAM32A	1.206833071
6349	1.4351171	1.1896577	DDX60	1.206327697
6350	0.7817773	0.6480638	ADTRP	1.206327697
6351	14.835219	12.297835	CENPT	1.206327697
6352	0.9242639	0.7661798	FAM21A	1.206327697
6353	1.5691608	1.3007749	ZNF720	1.206327697
6354	2.9195394	2.4201876	PEX7	1.206327697
6355	8.0994254	6.7141171	ARRDC1	1.206327697
6356	2.4510723	2.0318461	PPFIBP2	1.206327697
6357	40.931439	33.930614	ATP5SL	1.206327697
6358	1.3560109	1.1240817	PIK3CG	1.206327697
6359	3.5609353	2.9518806	C5orf45	1.206327696
6360	4.4205955	3.6645063	SCARNA7	1.206327696
6361	0.3700651	0.3067699	FLJ12825	1.206327696
6362	34.808593	28.855006	ZCRB1	1.206327696

6363	2.3787958	1.9719316	DUSP18	1.206327696
6364	0.4820078	0.3995662	AP3S2	1.206327696
6365	0.9699445	0.8040473	KBTBD7	1.206327696
6366	0.2107174	0.1746767	NALCN	1.206327692
6367	105.45358	87.43526	NFKBIA	1.206076116
6368	33.780722	28.03114	GOLGA7	1.205114087
6369	12.126505	10.064598	AMMECR1L	1.204867251
6370	57.008024	47.315515	EPHA2	1.204848447
6371	19.699047	16.352444	INTS10	1.204654566
6372	50.941283	42.294456	COPA	1.204443489
6373	6.8909654	5.7219663	TMEM241	1.204300255
6374	3.9958606	3.319495	NR2C1	1.203755569
6375	57.84571	48.076779	PDHB	1.203194378
6376	36.321967	30.192765	ABL1	1.203002325
6377	76.202291	63.387308	MMP14	1.202169539
6378	90.254129	75.084163	RRM2	1.202039484
6379	16.232122	13.504043	SNAP47	1.202019383
6380	73.214539	60.921575	ELOVL1	1.201783431
6381	2.1030694	1.7501221	LYSMD3	1.201670061
6382	7.0659058	5.8820831	SLFN5	1.201259093
6383	268.64571	223.63806	HMGN2	1.201252203
6384	13.231714	11.019371	GLRX2	1.200768583
6385	4.221937	3.5160288	PPOX	1.200768583
6386	162.74539	135.55641	RPN2	1.200573183
6387	29.332575	24.432292	CERCAM	1.200565819
6388	0.652655	0.5441357	ACACB	1.199434396
6389	1.273943	1.0621198	TMEM67	1.199434396
6390	133.83827	111.62131	SF1	1.199038707
6391	4.4639564	3.7257965	NAIF1	1.198121386
6392	227.27241	189.72234	PRMT1	1.197921232
6393	36.677188	30.624038	TBCD	1.197660084
6394	160.87559	134.36523	CCNI	1.197300755
6395	34.658232	28.948017	TRA2A	1.197257564
6396	2.6764676	2.2354986	ZNF329	1.197257563
6397	62.341731	52.076465	PAIP2	1.197119088
6398	4.8815383	4.0799159	TRIM4	1.196480124
6399	5.5679256	4.6540629	MED23	1.196358046
6400	55.33366	46.260443	XPO6	1.196133378
6401	27.94284	23.362813	DERL2	1.196039188
6402	114.89037	96.062415	UBE2M	1.195997136
6403	380.97682	318.55454	PSMB4	1.19595474
6404	10.659702	8.913664	SUZ12	1.195883301
6405	3.9280599	3.2855482	KHDRBS3	1.195556914

6406	11.808241	9.8767705	C11orf83	1.195556913
6407	15.121592	12.651543	BCOR	1.195236989
6408	7.3050632	6.1123215	ARRDC2	1.195137273
6409	41.351114	34.610462	LYPLA2	1.194757668
6410	13.165454	11.024574	CCDC109B	1.194191604
6411	32.430083	27.160459	TMEM222	1.19401823
6412	12.135156	10.163688	MFHAS1	1.193971779
6413	34.497059	28.895885	UBR4	1.193839832
6414	66.089656	55.363452	SERINC3	1.193741613
6415	3.6787202	3.0834034	ALDH4A1	1.193071348
6416	11.885903	9.9624411	C6orf57	1.193071348
6417	13.951977	11.69627	KDM5B	1.19285696
6418	37.934157	31.812945	CACYBP	1.192412648
6419	7.5502224	6.3326993	BICC1	1.192259735
6420	3.5043155	2.9395276	FAM160B1	1.192135606
6421	23.92406	20.068863	MBD1	1.192098405
6422	6.7353451	5.6517695	THAP3	1.191723245
6423	2.3681761	1.9871863	GCNT1	1.191723245
6424	33.283119	27.930801	STXBP1	1.191627826
6425	6.5894081	5.530388	PHKG2	1.191491121
6426	26.300257	22.078592	DPM2	1.191210808
6427	528.98137	444.26992	RPL23A	1.190675623
6428	107.29863	90.123891	SZRD1	1.190568071
6429	4.0581335	3.409346	KLHL29	1.190296764
6430	4.0653564	3.4160652	TMEM50B	1.190069911
6431	5.2286613	4.3945618	MRS2	1.18980266
6432	8.4486284	7.1008653	TSPYL4	1.18980266
6433	2.9605859	2.4897819	NKX2-4	1.189094444
6434	23.938888	20.138927	NIT2	1.188687359
6435	3.0555452	2.5720198	KLHL25	1.187994449
6436	35.35073	29.759411	STAT6	1.187884081
6437	15.064199	12.681588	TBC1D2B	1.187879567
6438	34.633728	29.1638	COPB1	1.187558807
6439	55.610988	46.839013	SDC1	1.187279238
6440	1.0122821	0.8526781	ZNF250	1.187179638
6441	87.031087	73.309113	MRPS15	1.187179638
6442	21.483713	18.098765	ACAD9	1.187026453
6443	26.127172	22.024288	LOXL1	1.186289031
6444	4.3117138	3.6353458	GATA6	1.186053282
6445	2.8420181	2.3967614	SSH2	1.185774276
6446	40.08152	33.807645	NFKB2	1.185575612
6447	7.8569484	6.6278814	SMIM12	1.185438905
6448	8.7258588	7.3616586	HDHD2	1.185311535

6449	16.613978	14.018844	MED18	1.185117539
6450	49.521554	41.788504	HDGFRP2	1.185052076
6451	27.045711	22.827505	TMEM248	1.18478613
6452	112.08627	94.631642	HGS	1.184448103
6453	26.452321	22.334046	PPCS	1.184394466
6454	4.6590378	3.9336876	TFB1M	1.184394466
6455	32.8016	27.704898	NFE2L2	1.183963934
6456	5.8840459	4.970265	BLOC1S2	1.183849541
6457	34.728703	29.339077	TNS3	1.183701303
6458	13.286815	11.22926	DERA	1.183231584
6459	21.641487	18.294343	NECAP1	1.182960574
6460	36.723176	31.054464	NREP	1.182540953
6461	14.14774	11.964318	ZNF574	1.182494475
6462	18.456296	15.610078	CHMP1B	1.182332028
6463	11.324722	9.5833442	C12orf23	1.181708764
6464	0.7830888	0.6626749	LOC100128288	1.181708764
6465	3.8414654	3.2507717	D2HGDH	1.181708764
6466	9.4987591	8.0381558	RAB18	1.181708764
6467	2.2744829	1.9247407	TPM3P9	1.181708764
6468	11.139394	9.426514	C1orf86	1.181708764
6469	2.9665409	2.5103824	SLC46A3	1.181708764
6470	0.7273979	0.6155475	LSR	1.181708764
6471	1.3515979	1.1437657	SENP7	1.181708763
6472	1.3106887	1.109147	CCDC107	1.181708763
6473	0.7668795	0.6489581	EGLN2	1.181708762
6474	20.832679	17.651259	PIP4K2B	1.180237513
6475	4.5691004	3.871408	TP73	1.180216707
6476	228.24707	193.44328	C19orf43	1.179917309
6477	23.227997	19.68783	AKR1A1	1.179815
6478	71.991074	61.039305	SH3GL1	1.179421586
6479	7.6486493	6.4851997	NUF2	1.179400739
6480	5.9964442	5.0855366	CYB5R4	1.179117297
6481	17.79147	15.093448	IL13RA1	1.178754492
6482	4.8502599	4.1174761	ZNF628	1.177969179
6483	29.307928	24.88245	GLOD4	1.177855366
6484	27.889609	23.685141	UBE2O	1.177515003
6485	14.24606	12.10468	CREG1	1.17690507
6486	5.3868278	4.5782409	C12orf4	1.176615192
6487	2.2640933	1.9242428	HERC2P2	1.176615191
6488	17.753834	15.090937	SLC39A3	1.176456725
6489	7.0039473	5.9548791	MIA3	1.176169504
6490	18.567083	15.789463	ARMCX6	1.175916074
6491	30.100232	25.603979	IMPAD1	1.17560755

6492	17.760953	15.108252	TMEM63B	1.175579569
6493	22.060639	18.775099	STARD3	1.17499451
6494	2.7816271	2.3673533	IL17D	1.17499451
6495	5.620267	4.7832283	LETM2	1.17499451
6496	38.557302	32.825382	UBE2V2	1.174618511
6497	12.390836	10.550652	NLGN2	1.174414266
6498	8.0912304	6.8908574	EHBP1	1.174197903
6499	30.135467	25.664726	SMIM7	1.174197903
6500	37.390439	31.847497	HADHB	1.174046387
6501	83.399723	71.043686	USP22	1.173921689
6502	3.5754816	3.0462706	COX19	1.173724245
6503	23.955891	20.41401	APOPT1	1.173502453
6504	161.94901	138.00766	ATOX1	1.173478388
6505	38.616032	32.908558	HIGD1A	1.173434351
6506	3.3229271	2.8329101	NSL1	1.172973014
6507	2.4400496	2.0803737	GNAL	1.172890042
6508	18.105739	15.439973	SERPINB8	1.172653524
6509	6.738373	5.7466611	C1orf52	1.172571841
6510	20.184772	17.217012	RBM5	1.172373704
6511	17.998598	15.353232	ASL	1.172300255
6512	0.4431259	0.3781386	DOPEY1	1.171861192
6513	2.5828371	2.2040469	LIG4	1.171861191
6514	1.0816033	0.922979	NAIP	1.171861191
6515	4.1614622	3.5511563	PRKAR1B	1.171861191
6516	2.629484	2.2438528	ZNF70	1.171861191
6517	8.33598	7.1134534	CCDC174	1.171861191
6518	1.3491209	1.1512634	SPRY1	1.171861191
6519	1735.3617	1481.4971	TUBB	1.171356802
6520	50.935632	43.490422	MRPS7	1.171191938
6521	5.4460809	4.6506731	IMPA1	1.171030673
6522	8.4777903	7.2407299	CD109	1.17084747
6523	5.8897645	5.031576	LOC100506190	1.170560568
6524	13.629384	11.649848	VPS26B	1.169919416
6525	34.109739	29.157397	CENPM	1.169848548
6526	5.1063798	4.3659157	CLN5	1.169601092
6527	6.8380627	5.8469028	KIF13A	1.169518807
6528	129.63433	110.86265	PSMB6	1.16932378
6529	6.9499994	5.9440742	NF1	1.169231594
6530	21.114561	18.059631	KDM3B	1.169157935
6531	12.730354	10.888985	CUX1	1.169103871
6532	1.8145971	1.5524449	ZSWIM3	1.168864104
6533	66.315542	56.751261	POLR2E	1.168529856
6534	4.3964007	3.7631388	ACAD10	1.168280255

6535	19.160361	16.40139	RAI1	1.168215695
6536	2.8952753	2.4792427	STX17	1.167806308
6537	8.0771228	6.9174722	C2orf68	1.167640803
6538	3.1264391	2.6780897	CABYR	1.1674139
6539	7.2432795	6.2045514	ABCB6	1.1674139
6540	11.125563	9.5330138	ATXN1L	1.167056209
6541	13.999626	11.996896	BTG1	1.166937405
6542	12.558681	10.762847	NAA40	1.166854883
6543	20.120203	17.24389	KIAA1279	1.166801887
6544	17.694297	15.167945	WIPF1	1.166558651
6545	39.164241	33.591308	C12orf44	1.165904017
6546	68.590097	58.840551	NACC1	1.165694326
6547	50.93128	43.700094	GOT1	1.165473003
6548	5.6539414	4.8519352	BLOC1S3	1.165296142
6549	83.832094	71.940591	DNAJC8	1.165296142
6550	19.077069	16.373046	SAP130	1.165150897
6551	11.290142	9.6938975	CEBPG	1.164664887
6552	19.693485	16.911367	SMC1A	1.164511715
6553	10.781884	9.2607006	INTS9	1.164262277
6554	15.043599	12.922118	FKBP15	1.164174416
6555	83.169336	71.480666	PTP4A2	1.163522122
6556	27.772626	23.869509	SUN1	1.163518979
6557	0.7238887	0.6223013	ANKRD23	1.163244565
6558	1.6438173	1.4131313	NRTN	1.163244565
6559	0.6315136	0.5428898	LOC100506123	1.163244565
6560	18.589627	15.980841	MSL1	1.163244565
6561	2.5091579	2.1570338	ZBTB3	1.163244565
6562	96.93	83.327275	MRPL28	1.163244565
6563	4.1154043	3.5378668	SPP1	1.163244565
6564	1.1984636	1.0302765	FBXO6	1.163244564
6565	0.365115	0.3138763	LRRC2	1.163244564
6566	0.5592115	0.4807343	PHYHIP	1.163244564
6567	25.684241	22.083912	PARP4	1.163029507
6568	151.72078	130.45555	C6orf48	1.16300741
6569	56.387034	48.489545	TXNL1	1.162869929
6570	18.121696	15.588827	ORMDL1	1.162479776
6571	22.758549	19.578934	UBTD1	1.162399797
6572	39.770897	34.232363	SNHG5	1.161792324
6573	16.904395	14.560491	LSM6	1.160977031
6574	78.853865	67.939177	RHOG	1.160653819
6575	1.4981012	1.2907913	THRB	1.160606822
6576	29.231609	25.193481	TMEM165	1.160284655
6577	2.5187538	2.171167	LOC253039	1.16009214

6578	13.857493	11.947424	TDP2	1.159872841
6579	4.0413922	3.4847058	LAMTOR3	1.159751337
6580	20.417347	17.628666	NFKB1	1.158190119
6581	139.77332	120.6849	RUVBL2	1.158167428
6582	2.9862774	2.5786572	HEMK1	1.158074589
6583	2.3118804	1.9963139	CLK4	1.158074589
6584	45.985554	39.728862	CARS	1.157484788
6585	5.4536618	4.713257	ICAM3	1.157089831
6586	33.891787	29.296713	ANKRD40	1.15684608
6587	11.978693	10.361617	RFWD2	1.156064043
6588	34.297408	29.667999	PDXP	1.1560405
6589	13.929958	12.053873	WASH5P	1.15564166
6590	1.0786136	0.9333461	ZNF543	1.155641659
6591	1.017628	0.880574	CREBRF	1.155641659
6592	7.4615399	6.4566207	MED26	1.155641659
6593	2.6954326	2.3324121	MZF1	1.155641659
6594	1.1232881	0.9720038	RPLP0P2	1.155641659
6595	19.603896	16.972817	SGPL1	1.15501719
6596	6.4090701	5.5501618	ARMC9	1.154753728
6597	2.5492294	2.2086028	MCTP1	1.154227165
6598	19.690051	17.062252	ZFAND2B	1.154012465
6599	8.564634	7.4222252	LRPAP1	1.153917288
6600	7.5372295	6.5320815	CNEP1R1	1.153878666
6601	7.3082468	6.3356261	PEAK1	1.153516115
6602	2.8561368	2.4764858	LOC100862671	1.153302303
6603	52.116687	45.200308	TRAM1	1.153016195
6604	20.331659	17.639051	TJAP1	1.152650351
6605	27.649043	23.993232	ARID1A	1.152368392
6606	18.50542	16.061418	TINF2	1.152166045
6607	5.8185615	5.0530514	PVRL3	1.151494619
6608	2.7122379	2.3554064	TMEM8B	1.151494619
6609	14.324753	12.441696	ARAP3	1.151350449
6610	43.277331	37.598093	XPNPEP1	1.151051225
6611	2.2782232	1.9792544	MBTD1	1.151051225
6612	4.5620449	3.9644564	SLC25A40	1.150736559
6613	34.043185	29.587046	CDKN3	1.150611165
6614	45.423507	39.482466	M6PR	1.150472885
6615	7.3983069	6.4315228	ZNF408	1.150319625
6616	5.2527424	4.5681028	ABCC10	1.149873937
6617	2.231258	1.9421099	ANKRD37	1.148883521
6618	1.0332152	0.8993211	ACTRT3	1.148883521
6619	6.9120896	6.0163536	TIGD5	1.148883521
6620	4.1185672	3.5848431	PMS2P1	1.148883521

6621	1.5425574	1.3426578	GDPGP1	1.14888352
6622	2.7100065	2.3588175	ZKSCAN4	1.14888352
6623	0.72512	0.6311519	LOC349196	1.14888352
6624	1.1613235	1.0108279	WHAMMP3	1.14888352
6625	0.7440182	0.6476011	KANK3	1.148883519
6626	21.430125	18.667036	KIF18B	1.148019699
6627	7.5424513	6.5726254	RANBP10	1.147555332
6628	2.8845454	2.514182	TBC1D15	1.147309708
6629	21.180149	18.467259	PBK	1.146902687
6630	5.4948429	4.7925483	LIMCH1	1.14653886
6631	2.808025	2.4494586	TDRD7	1.146385948
6632	13.580173	11.849947	PCED1A	1.146011311
6633	3.4157024	2.9811195	VSIG10	1.14577843
6634	6.4002843	5.5859703	RNF146	1.14577843
6635	5.6547846	4.9353212	DUSP22	1.14577843
6636	29.466172	25.718902	C16orf58	1.145701018
6637	18.045221	15.750848	RPS6KA1	1.145666647
6638	13.075593	11.414703	MIIP	1.145504451
6639	6.6222406	5.7822166	KIAA1217	1.14527716
6640	21.567068	18.831792	TUSC2	1.145247813
6641	10.468251	9.1411606	C10orf76	1.145177444
6642	492.21668	429.88502	RHOA	1.14499611
6643	4.7173308	4.12073	CCNE2	1.144780365
6644	2.2747833	1.9870915	LINC00341	1.144780365
6645	5.0511216	4.4123063	CCDC134	1.144780365
6646	2.1420373	1.8711339	MBD5	1.144780365
6647	5.3645344	4.6871592	DST	1.144517217
6648	11.737698	10.25707	MLL4	1.144351961
6649	51.798085	45.266012	SUPT5H	1.144304149
6650	58.872906	51.452022	MPRIP	1.144229195
6651	0.8267298	0.7226693	CSMD2	1.143994654
6652	11.121087	9.7217486	QKI	1.143938959
6653	3.9835464	3.4831521	HOXD-AS1	1.143661323
6654	4.6715563	4.0861027	ARID4B	1.143279211
6655	47.626056	41.664821	PRPF31	1.143075978
6656	27.000861	23.626174	PEX14	1.142836765
6657	3.6439546	3.1885171	CRADD	1.142836765
6658	11.362016	9.9452526	IFNAR2	1.1424562
6659	3.0527014	2.6723733	PHLPP2	1.142318472
6660	12.305728	10.773357	SPECC1L	1.142237087
6661	30.711505	26.899733	MINK1	1.141702999
6662	102.63987	89.912064	PFDN2	1.141558424
6663	18.76438	16.443816	LEPROTL1	1.141120794

6664	64.415255	56.4628	FLII	1.14084414
6665	5.2984835	4.6445625	ZEB2	1.140792791
6666	10.003924	8.7708126	FAM220A	1.140592609
6667	37.390567	32.804402	HUWE1	1.139803326
6668	16.116825	14.143709	NR1D1	1.13950488
6669	17.678521	15.518491	USP36	1.139190706
6670	1576.5079	1384.2572	PFN1	1.138883638
6671	5.2075987	4.5743322	UBOX5	1.138439125
6672	7.9286372	6.9644806	ZNF668	1.138439125
6673	27.943024	24.548817	NUDCD2	1.138263589
6674	10.699232	9.4038308	WNT5A	1.137752469
6675	0.6642696	0.5840274	BBS12	1.137394686
6676	1.5949866	1.4023159	TBC1D12	1.137394686
6677	1.4976445	1.3167325	KIAA1841	1.137394686
6678	1.0419975	0.9161266	ZNF354B	1.137394685
6679	3.5518973	3.1228362	RABIF	1.137394685
6680	1.1607061	1.0204954	WASH2P	1.137394685
6681	2.2866778	2.0104523	ZSCAN21	1.137394685
6682	0.5684092	0.4997467	LOC100131564	1.137394684
6683	0.2674594	0.2351509	IGSF10	1.137394682
6684	6.8907788	6.0638465	ZNF48	1.136370928
6685	9.3240658	8.2055103	6-Sep	1.136317607
6686	4.0250772	3.5428333	PDLIM2	1.136118148
6687	11.339447	9.9808695	MEAF6	1.136118148
6688	7.2309435	6.3656126	HSD17B1	1.135938354
6689	5.135258	4.5216692	CREM	1.135699611
6690	32.246574	28.393577	NDUFB1	1.135699611
6691	40.798452	35.925447	KLF2	1.135642151
6692	11.142808	9.8149916	SPG20	1.135284491
6693	12.52631	11.034205	POP4	1.135225465
6694	1.5511045	1.3667651	STXBP4	1.134872746
6695	144.15651	127.03915	JUN	1.134740896
6696	7.9682162	7.0222553	SLC25A4	1.134708984
6697	62.758172	55.316817	MRPL40	1.134522477
6698	138.1443	121.80883	COX7A2L	1.134107417
6699	1.5484229	1.365381	HACE1	1.134059217
6700	103.94927	91.689736	CHMP4B	1.133706723
6701	2.1020654	1.8546254	ZXDB	1.133417781
6702	24.720357	21.812125	SUGP1	1.133330949
6703	5.0613413	4.4672242	MMAB	1.132994706
6704	2.609248	2.3040309	SYT15	1.132470899
6705	114.37712	101.00283	SND1	1.132415105
6706	319.05064	282.09136	SUMO2	1.131018844

6707	41.613584	36.798115	POLA2	1.130861818
6708	122.49607	108.32657	C19orf53	1.130803481
6709	9.8182716	8.6826295	TLK2	1.130794716
6710	19.571864	17.313809	RNF216	1.130419322
6711	163.77799	144.92479	LRRC59	1.130089551
6712	3.6184728	3.2029381	C1RL	1.129735462
6713	14.462032	12.807914	PQLC1	1.129148098
6714	22.947488	20.327611	PTPRS	1.128882715
6715	12.582212	11.145974	CCRN4L	1.12885711
6716	44.626532	39.545505	ACAA2	1.128485588
6717	2.2072321	1.9567752	LOC100289187	1.127994729
6718	2.699184	2.3929048	BBC3	1.127994729
6719	5.9489599	5.2739252	C1orf112	1.127994729
6720	1.6885847	1.4969793	HERC2P9	1.127994729
6721	0.7422956	0.6580666	LOC728743	1.127994729
6722	4.3830771	3.8893665	FANCC	1.126938554
6723	58.448179	51.868931	RPS19BP1	1.126843714
6724	6.5932127	5.8543963	DGCR14	1.126198559
6725	2.2819163	2.0267381	RCHY1	1.12590585
6726	6.9184116	6.1447514	WDR83	1.12590585
6727	25.394376	22.557342	COPS5	1.125769888
6728	20.553042	18.262909	NSF	1.125398057
6729	30.85244	27.425306	MICU1	1.124962485
6730	11.676033	10.379657	CHCHD7	1.124895843
6731	11.087149	9.8572962	KANSL1	1.124765737
6732	7.0192874	6.2413771	TFDP2	1.124637602
6733	14.951477	13.294894	PELO	1.124602993
6734	10.711677	9.5270437	SUMF1	1.12434426
6735	55.256496	49.14769	CRTAP	1.12429487
6736	32.688651	29.081922	ADPGK	1.124019623
6737	16.080208	14.307409	TRIM47	1.123907792
6738	1.2464085	1.1089953	USP43	1.123907792
6739	2.218432	1.9738559	ENPP4	1.123907792
6740	1.4488286	1.2890991	HELQ	1.123907792
6741	1.1017102	0.9802496	LCA5	1.123907791
6742	20.965115	18.661838	ATP13A2	1.123421777
6743	5.8339927	5.1933754	SAP30L	1.123352776
6744	27.664729	24.633626	CHMP7	1.123047384
6745	12.505495	11.135648	ANAPC2	1.12301462
6746	5.6203779	5.0047237	ANKIB1	1.12301462
6747	10.69253	9.5299284	C22orf23	1.121994758
6748	15.725684	14.01842	ZFYVE21	1.121787211
6749	5.9041912	5.2639765	ZNF394	1.121621878

6750	13.672409	12.191582	TPCN1	1.121463038
6751	4.3024952	3.8378524	RIC8B	1.121068446
6752	7.5902222	6.7705253	GGPS1	1.121068446
6753	25.852784	23.079517	ARFGAP2	1.120161433
6754	6.6297341	5.9185524	PPIP5K1	1.120161433
6755	8.9481814	7.9882963	SENP1	1.120161432
6756	1.7518225	1.5639018	TESK2	1.120161432
6757	1.996458	1.7822949	USP27X	1.120161432
6758	2.9357343	2.6208136	UTP14C	1.120161432
6759	17.24725	15.405637	SLC39A6	1.119541531
6760	14.627979	13.068573	MRI1	1.119324867
6761	50.227222	44.889026	REEP5	1.118919844
6762	13.353039	11.93434	KDM4A	1.118875369
6763	23.224242	20.757725	RAB14	1.11882404
6764	12.41272	11.096163	RBM4	1.118649744
6765	51.992255	46.489692	SEPN1	1.118360943
6766	35.248206	31.52806	ATP5G3	1.117994776
6767	1.9336534	1.7298238	USP35	1.117832614
6768	14.484504	12.960051	CISD1	1.117627131
6769	54.324364	48.613039	UQCR11	1.117485462
6770	2.1929899	1.9626145	GEN1	1.117381876
6771	0.8582652	0.7685626	OLFML2A	1.116714783
6772	2.5965789	2.3251944	CTPS2	1.116714782
6773	4.7523535	4.2556556	BBIP1	1.116714782
6774	7.6933583	6.8933303	CLDN12	1.116058277
6775	85.749895	76.84772	MRPL18	1.115841752
6776	1.3599203	1.2189747	ZNF814	1.115626366
6777	3.5483304	3.1805723	FBXO3	1.115626366
6778	13.939587	12.499892	DEF8	1.115176607
6779	7.3569409	6.6019577	CISD2	1.114357487
6780	6.2778172	5.6356706	SOAT1	1.113943249
6781	44.834711	40.250895	POLDIP3	1.113881093
6782	47.409443	42.570702	FKBP10	1.113663649
6783	0.8019772	0.7202095	NPIPL3	1.113533259
6784	1.960748	1.7608347	MDM1	1.113533259
6785	0.6078319	0.5458588	SLFNL1-AS1	1.113533259
6786	2.8716467	2.5788602	HS3ST3B1	1.113533258
6787	4.3807703	3.9341171	TMSB15A	1.113533258
6788	12.529925	11.252403	ASB6	1.113533258
6789	191.2018	171.76152	PCBP2	1.113181838
6790	9.2221039	8.2887643	LYN	1.112602988
6791	15.33676	13.785524	TIMM8B	1.112526447
6792	101.7765	91.486404	SDHB	1.112476775

6793	120.71375	108.58586	LMNB2	1.11168945
6794	3.2921302	2.9617612	EGLN3	1.111544806
6795	35.181295	31.650812	CNPY3	1.111544806
6796	19.186873	17.271667	FGFR1	1.110887177
6797	276.24143	248.68252	S100A10	1.110819677
6798	16.009498	14.415343	GPN2	1.110587403
6799	33.812432	30.456594	EIF2B1	1.110184286
6800	15.145313	13.642981	CXorf56	1.110117512
6801	252.11257	227.11001	DYNLL1	1.110090051
6802	48.449565	43.647304	PIH1D1	1.110024225
6803	0.8555307	0.7709892	FRY	1.109653352
6804	2.7803451	2.5055979	ETAA1	1.109653352
6805	6.3372559	5.7117524	LGALS8	1.10951167
6806	44.768456	40.354827	GRAMD1A	1.109370517
6807	11.710067	10.557268	MRPL50	1.109194817
6808	4.3834476	3.9519186	PNPLA3	1.109194817
6809	2.4010097	2.164642	CCSAP	1.109194817
6810	19.711536	17.774153	DNAJB12	1.108999999
6811	15.837935	14.285761	TUBGCP3	1.108651859
6812	99.207379	89.508043	SLC25A39	1.108362732
6813	29.893605	26.979715	APBA2	1.108003003
6814	0.3925154	0.3543031	REPS2	1.107851968
6815	0.8265448	0.7460788	ZNF788	1.107851966
6816	7.5373553	6.8035763	ZNF646	1.107851966
6817	1.4189707	1.2808306	IDUA	1.107851966
6818	10.400165	9.3876849	NFIL3	1.107851966
6819	10.511955	9.4885914	ITFG2	1.107851966
6820	1.1289247	1.0190213	PRRG4	1.107851966
6821	0.7391801	0.6672192	ZNF761	1.107851966
6822	0.8895824	0.8029795	LOC642846	1.107851965
6823	8.5650112	7.7342385	EFNB2	1.107414944
6824	14.150754	12.77848	KCMF1	1.107389397
6825	21.776606	19.665061	RBBP4	1.107375471
6826	4.6330876	4.1866591	TLN2	1.106631192
6827	11.421716	10.321857	C18orf8	1.106556233
6828	2.0093014	1.8165075	ZDHHC17	1.106134367
6829	5.9230009	5.3546848	SNX25	1.106134366
6830	9.0228834	8.1632508	HILPDA	1.10530518
6831	15.208729	13.765289	PRRC1	1.104860881
6832	10.003816	9.0557212	RNF19B	1.104695693
6833	0.3683909	0.3335379	SH3TC2	1.104494837
6834	14.044763	12.717423	ACSS2	1.104371802
6835	21.866123	19.802331	TM7SF3	1.104219665

6836	27.240459	24.670126	SFXN3	1.104188052
6837	63.135948	57.17902	SLC2A1	1.104180309
6838	7.6984207	6.9734297	RUNDC1	1.103964766
6839	214.30926	194.16971	ITGB1	1.103721362
6840	1.8100158	1.6399487	GK5	1.103702708
6841	23.26999	21.093182	COX11	1.103199569
6842	2.0294534	1.8400595	ADAT3	1.10292818
6843	1.4298422	1.2964055	LOC100132832	1.10292818
6844	1.931861	1.7515746	MTM1	1.10292818
6845	1.2785245	1.1592092	CYP1A1	1.102928179
6846	44.733477	40.571768	CSNK1G2	1.102576481
6847	12.811085	11.626128	FAM188A	1.101921858
6848	12.810303	11.626034	POLR3H	1.101863577
6849	108.67856	98.634239	ILK	1.101834005
6850	21.117816	19.16944	CLASRP	1.101639712
6851	5.1288677	4.656555	CHEK2	1.101429636
6852	22.509637	20.450208	FNDC3B	1.100704534
6853	3.3596402	3.0522634	WDFY3	1.100704534
6854	7.1064743	6.4588053	MECR	1.10027691
6855	4.1999093	3.8181172	TMEM254	1.09999486
6856	4.4396996	4.0361094	SLC35E3	1.09999486
6857	95.835091	87.137085	FAM96B	1.099819795
6858	15.711761	14.286619	BICD2	1.099753634
6859	6.5614504	5.9668771	C15orf38	1.099645655
6860	15.186399	13.814607	MSRB1	1.099300126
6861	3.9446809	3.5886768	DPY19L4	1.099202071
6862	271.05948	246.65894	MYL12B	1.098924222
6863	273.91904	249.26277	ATP5J2	1.098916764
6864	0.6327543	0.5759538	SLC2A12	1.098619867
6865	0.5444585	0.4955841	SLC46A1	1.098619866
6866	2.3208592	2.1125225	PLCD1	1.098619866
6867	28.66649	26.097966	KCTD5	1.098418543
6868	276.60853	251.8362	CCT7	1.098366833
6869	26.517977	24.157962	TMED7	1.09769097
6870	3.4013729	3.0997629	AFG3L1P	1.097300995
6871	9.8018938	8.9362137	KLHL12	1.096873253
6872	15.86408	14.463863	SLC4A1AP	1.096807959
6873	66.266831	60.423637	SLC39A7	1.09670377
6874	5.0953423	4.6462305	TRMT44	1.096661542
6875	3.4576831	3.1529173	UFL1	1.096661542
6876	22.765239	20.758673	IP6K1	1.096661542
6877	41.815887	38.13956	LAMTOR4	1.096391429
6878	21.11416	19.270803	VHL	1.095655431

6879	35.990494	32.873482	MAT2B	1.094818414
6880	26.033502	23.778831	PSMD10	1.094818414
6881	12.832357	11.72642	CENPO	1.094311553
6882	56.991659	52.086431	SLC9A3R2	1.094174782
6883	47.0782	43.026216	BTBD2	1.094174781
6884	19.344802	17.680838	TFIP11	1.094111167
6885	5.5211814	5.0484046	GCC1	1.093648736
6886	43.56784	39.853102	SLC2A4RG	1.093210751
6887	16.762568	15.335162	KAT8	1.093080607
6888	3.4411197	3.1487851	KIAA0232	1.092840422
6889	17.746825	16.244854	FOXK1	1.09245831
6890	26.154276	23.948943	MRPS23	1.092084785
6891	64.199846	58.800907	VKORC1	1.091817267
6892	105.94222	97.066522	RN7SL1	1.091439344
6893	18.430707	16.892739	TMEM55B	1.091043178
6894	3.4293913	3.1433841	ZYG11B	1.090987028
6895	6.7057089	6.1483464	ZBTB45	1.090652438
6896	26.93526	24.700509	ISOC1	1.090473896
6897	4.8465	4.4455531	EPC1	1.090190558
6898	42.965387	39.424753	BAP1	1.089807408
6899	18.004178	16.526126	QSOX2	1.089437269
6900	8.3801545	7.6962629	SEC22C	1.088860218
6901	34.169492	31.382691	GYS1	1.088800566
6902	0.7764512	0.7133773	CROCCP3	1.088415967
6903	14.573392	13.389542	DPCD	1.088415967
6904	13.501124	12.404379	SLC10A3	1.088415967
6905	1.6080208	1.4773955	REL	1.088415967
6906	1.6612156	1.5262691	SDCBP2-AS1	1.088415967
6907	110.56178	101.60539	EFHD2	1.088148761
6908	56.273852	51.721822	RNF187	1.088009842
6909	49.447681	45.474342	SAP18	1.087375416
6910	15.557865	14.312393	ANO10	1.087020562
6911	21.353249	19.658361	TMEM14A	1.086217147
6912	66.58008	61.320421	HSF1	1.085773379
6913	1.0601719	0.9764915	ANO7	1.085694926
6914	4.9738148	4.5843015	PGM3	1.084966761
6915	17.485013	16.117486	SEMA4B	1.084847389
6916	37.171544	34.265059	SYMPK	1.084823583
6917	27.197168	25.094728	TRIM44	1.083780121
6918	13.784567	12.720768	CEP85	1.083626937
6919	12.62833	11.655182	TDG	1.083494938
6920	31.227665	28.828206	HDDC2	1.083233034
6921	2.7921979	2.5776521	C1orf74	1.083233034

6922	22.315924	20.607865	FTSJD2	1.082883829
6923	57.108775	52.756033	SRP68	1.082507006
6924	6.6985554	6.1903982	MED7	1.082087967
6925	14.076912	13.022181	CDC42EP2	1.080994949
6926	1.1420511	1.0564814	RFFL	1.080994949
6927	7.0467341	6.5187484	UAP1L1	1.080994949
6928	1.1344825	1.0494799	C1orf106	1.080994949
6929	8.0153654	7.4148037	RAB3IL1	1.080994949
6930	2.3177894	2.144126	RNF170	1.080994949
6931	47.952621	44.370608	NDUFAB1	1.080729413
6932	27.670159	25.604667	RCN2	1.080668561
6933	151.63301	140.46525	ATP5O	1.079505519
6934	14.617565	13.545774	MLL2	1.079123595
6935	1.0601077	0.9825351	ABCB1	1.078951481
6936	3.8787034	3.5948821	LOC154761	1.07895148
6937	2.7511485	2.5498353	ZNF252P	1.07895148
6938	1.3932	1.2912536	C3orf62	1.07895148
6939	12.729692	11.803495	FAM103A1	1.078468079
6940	2.2326333	2.0704945	PPP2R3A	1.078309247
6941	22.01861	20.419943	KPNA6	1.078289473
6942	1794.2734	1664.3373	GNB2L1	1.078070743
6943	10.242302	9.5012526	CETN3	1.077994963
6944	13.956166	12.950133	WIPF2	1.077685105
6945	4.9690632	4.6115235	EOGT	1.077531807
6946	17.108279	15.879527	DKK1	1.077379581
6947	8.0889773	7.5087138	MEF2A	1.077278687
6948	6.6510479	6.1740497	LPGAT1	1.077258565
6949	13.094076	12.157033	ACBD3	1.077078301
6950	8.9641905	8.3226916	MINPP1	1.077078301
6951	6.771494	6.2869097	MORC4	1.077078301
6952	1.3311159	1.235858	TRIM23	1.0770783
6953	6.46698	6.0058442	LIN7C	1.076781175
6954	14.184914	13.174642	LOC100505876	1.076683043
6955	12.950419	12.030248	NSUN5	1.076488121
6956	17.94491	16.673313	FEM1A	1.076265411
6957	3.2642428	3.0331218	FAM109A	1.076199053
6958	3.8052553	3.5367656	L3MBTL3	1.075913891
6959	6.5823823	6.1187479	SLC35A5	1.075772751
6960	6.5522431	6.0907316	FAM177A1	1.075772751
6961	0.6586904	0.612533	LOC646214	1.075354976
6962	4.0955306	3.8085383	CGRRF1	1.075354975
6963	12.445713	11.575556	HEATR6	1.075171967
6964	6.7956359	6.3218424	SPRYD7	1.074945472

6965	200.971	186.98514	PPP1R14B	1.074796639
6966	25.701891	23.924744	POLR3D	1.074280694
6967	17.760248	16.536791	UBE2F	1.073983932
6968	3.1527405	2.9355565	KIAA1549	1.073983932
6969	59.391939	55.305026	RAP1B	1.073897667
6970	307.38484	286.33812	BANF1	1.073503044
6971	22.783225	21.22409	C6orf1	1.073460633
6972	29.654759	27.627316	MOCS2	1.073385461
6973	7.1280924	6.6430572	DHRS11	1.073013854
6974	26.516279	24.713374	UBE3C	1.0729526
6975	110.81061	103.31103	UQCR10	1.072592238
6976	76.219864	71.078892	HNRNPA0	1.072327689
6977	6.1585077	5.743316	LOC100287314	1.072291286
6978	17.878566	16.681398	ARFRP1	1.071766681
6979	33.287714	31.06864	HDAC7	1.071424858
6980	26.940739	25.146913	ANKRD52	1.071333883
6981	22.174854	20.698932	GIT1	1.071304273
6982	5.0616294	4.7264152	SPATA2L	1.070923567
6983	16.169525	15.100988	TRMT2A	1.070759441
6984	18.772444	17.5419	VBP1	1.070148845
6985	30.447564	28.456725	SLC6A6	1.069960218
6986	1.4776613	1.3814435	FAM214A	1.069650175
6987	3.3079286	3.0925331	PELI1	1.069650174
6988	13.008171	12.164611	DDR1	1.069345431
6989	10.267239	9.6014243	BANP	1.069345431
6990	36.268963	33.938913	TMEM54	1.068654225
6991	1.0615157	0.9934991	CADPS2	1.068461675
6992	24.016299	22.477455	MTG1	1.068461674
6993	2.8522669	2.6695079	SEPT7P2	1.068461674
6994	3.0487893	2.8534381	THAP10	1.068461674
6995	2.8211286	2.6403648	PHF7	1.068461674
6996	3.8534951	3.6065824	PKI55	1.068461674
6997	4.47966	4.1941125	ZHX2	1.068082921
6998	95.708624	89.62188	ARPC4	1.067915824
6999	3.2123237	3.0096258	VRK2	1.067349851
7000	1062.7757	995.80155	RPS20	1.067256551
7001	11.508893	10.788033	ECHDC1	1.066820412
7002	35.656114	33.429031	CCNG1	1.06662123
7003	2.5405185	2.3825383	GTDC1	1.066307518
7004	6.5155694	6.1104037	CAMTA1	1.066307518
7005	3.6600232	3.4324274	ANKRD36BP1	1.066307518
7006	8.8056127	8.2580424	TOP3B	1.066307517
7007	296.32968	277.93905	COX4I1	1.066167879

7008	9.7155944	9.1142849	GPATCH3	1.0659744
7009	8.5294847	8.0028162	UGCG	1.065810404
7010	4.4410835	4.1668607	MICALL2	1.065810404
7011	253.5291	237.8813	SNRPD2	1.065779842
7012	5.4948298	5.1578743	CBFA2T2	1.065328355
7013	6.4239193	6.02999	EEF1E1	1.065328355
7014	15.31843	14.382007	BRPF3	1.065110764
7015	9.7510616	9.1571242	SLC11A2	1.064860696
7016	23.639925	22.206806	MRPL43	1.06453511
7017	66.185803	62.175939	FEN1	1.064492217
7018	10.147187	9.5331851	FOXN3	1.064406791
7019	66.876279	62.845728	TXNRD1	1.064134041
7020	18.1328	17.042649	PCID2	1.063966043
7021	6.7104263	6.3078472	SGPP1	1.063821952
7022	0.9803191	0.9217529	CEP85L	1.063537887
7023	31.708725	29.817004	AKAP8L	1.063444398
7024	42.143525	39.636996	MICALL1	1.063237113
7025	16.149855	15.190974	COMMD3	1.063121793
7026	16.810519	15.814436	GGA3	1.062985687
7027	7.4789345	7.0375582	IFT43	1.062717256
7028	14.155677	13.321926	SMG8	1.062584897
7029	14.137337	13.306309	GTF2B	1.062453751
7030	15.376139	14.473304	NT5C2	1.06237935
7031	5.377589	5.0620997	FEM1C	1.062323803
7032	13.046655	12.282137	SUPT20H	1.062246403
7033	1.2973757	1.2217022	ANKRD44	1.061940984
7034	35.405082	33.341406	KDM2A	1.061895283
7035	23.509405	22.149727	LCMT1	1.061385769
7036	14.237503	13.416348	C16orf72	1.061205568
7037	1.9925424	1.8776215	CCDC149	1.061205568
7038	32.752478	30.86623	MED24	1.061110426
7039	17.03213	16.052483	TBP	1.061027722
7040	12.019131	11.328447	PACSIN3	1.060968956
7041	28.353327	26.73192	DGKZ	1.060654344
7042	2.5214701	2.3776062	USP53	1.060507865
7043	36.069144	34.021966	DHRS7	1.060172261
7044	18.534446	17.487883	C9orf89	1.059845048
7045	9.0994457	8.5856378	TCP11L1	1.059845048
7046	141.75497	133.76227	PFN2	1.059753055
7047	8.444064	7.9704497	PDCL	1.059421279
7048	2.3247753	2.1948106	ZSCAN2	1.059214563
7049	6.6058709	6.2365749	CDK11A	1.059214563
7050	21.447923	20.252782	LLPH	1.059011181

7051	36.268963	34.254918	TRPC4AP	1.058795791
7052	2.3947564	2.2621618	CACNB1	1.058614101
7053	5.9149693	5.5874651	SERGEF	1.058614101
7054	74.370336	70.274483	NAP1L4	1.058283646
7055	1.7005894	1.6072992	PTPRE	1.058041568
7056	13.136931	12.416271	CHCHD5	1.058041567
7057	10.2106	9.6529935	DCAF6	1.057765173
7058	51.902847	49.107552	15-Sep	1.056921891
7059	12.410488	11.742669	TBC1D13	1.056871168
7060	4.9612543	4.6943972	CUL9	1.05684589
7061	21.867138	20.691108	NUP214	1.056837477
7062	14.922433	14.120791	TAB1	1.056770392
7063	163.00322	154.34561	PCBP1	1.056092339
7064	35.021769	33.162026	SPSB1	1.056080502
7065	72.548631	68.701677	PDIA6	1.055995066
7066	5.807921	5.4999509	TTC7A	1.055995066
7067	2.5158893	2.3824821	CBLB	1.055995065
7068	5.9948848	5.678246	TRIM68	1.055763488
7069	5.1327346	4.8626774	PRR5L	1.055536734
7070	6.1626889	5.838441	ZNF830	1.055536734
7071	24.578829	23.289195	PPP1R10	1.055374768
7072	20.297585	19.232878	MTMR14	1.055358705
7073	6.9819122	6.615953	MED21	1.055314657
7074	1.771502	1.6789943	FAM13A	1.055097111
7075	2.144469	2.032485	PNPLA8	1.05509711
7076	52.360809	49.629896	UBE2J2	1.055025579
7077	9.5173838	9.0234044	RNA45S5	1.054744235
7078	20.091445	19.049891	TAF11	1.054675072
7079	3.0236862	2.867493	SYNJ1	1.05447032
7080	5.2190554	4.9500245	BAHCC1	1.054349404
7081	7.2925801	6.9171872	C1orf131	1.054269584
7082	137.65335	130.60831	RANGAP1	1.053940254
7083	10.277236	9.7552839	INO80C	1.053504512
7084	12.719879	12.074713	ZNF185	1.053431167
7085	11.598462	11.016827	TLDC1	1.052795081
7086	5.6310721	5.3495483	IFT122	1.052625712
7087	22.047785	20.952076	CETN2	1.052295968
7088	33.348954	31.704839	CIRBP	1.051856889
7089	4.337827	4.1241048	SUCO	1.051822671
7090	57.371604	54.566737	RAB8A	1.051402494
7091	5.296103	5.0386962	LOC100129361	1.051085998
7092	26.926104	25.627495	DNPEP	1.05067251
7093	27.338121	26.024035	ATG4B	1.050495092

7094	23.947662	22.80673	PDE6D	1.050026101
7095	9.6365459	9.1774346	ARL6IP6	1.050026101
7096	9.6887164	9.2276635	ENC1	1.049964206
7097	33.033021	31.47717	COASY	1.049427932
7098	18.676043	17.801512	CCNC	1.049126805
7099	21.382156	20.383144	SH3KBP1	1.049011696
7100	26.018963	24.804045	HAT1	1.048980606
7101	7.5509646	7.1993676	ABCC5	1.048837204
7102	68.03584	64.876576	C7orf50	1.048696521
7103	23.084742	22.017832	TROAP	1.04845664
7104	45.786006	43.674092	AMOTL2	1.048356213
7105	14.148223	13.498159	UBE2J1	1.048159486
7106	13.62682	13.003087	SDHAF1	1.047968077
7107	22.595367	21.591303	TRABD	1.046503174
7108	4.2489218	4.0603086	KIFAP3	1.046452942
7109	4.0784516	3.8990248	HIVEP1	1.046018368
7110	45.453389	43.461346	C12orf10	1.045834808
7111	13.924591	13.315473	HIAT1	1.045745113
7112	28.432554	27.190525	RBM23	1.04567873
7113	17.626313	16.858441	ASPSCR1	1.045548187
7114	12.079207	11.553698	METTL22	1.045484004
7115	63.830273	61.067362	ACTR2	1.045243651
7116	12.487857	11.947424	ATPAF2	1.045234247
7117	2.8751155	2.7506901	KIAA1644	1.045234247
7118	159.50061	152.60557	PDLIM7	1.045182133
7119	9.3150514	8.9129576	PDE9A	1.045113396
7120	5.6008824	5.3603151	ADAM17	1.044879328
7121	9.7259854	9.308238	TRIP4	1.044879328
7122	7.7389735	7.4081626	VPS33B	1.044654912
7123	30.524397	29.224438	ERCC1	1.044481935
7124	4.0620579	3.8899928	CNST	1.044232744
7125	9.5710082	9.1673342	PRKACB	1.044033957
7126	9.1657853	8.7810726	LMO7	1.043811578
7127	22.065829	21.142764	BFAR	1.043658675
7128	67.889955	65.086137	FDFT1	1.043078581
7129	11.792362	11.306343	DOHH	1.04298643
7130	14.723878	14.118086	GCAT	1.04290892
7131	12.599404	12.081463	MED22	1.042870663
7132	7.1232598	6.8321304	NRF1	1.042611795
7133	5.2526923	5.0386962	HPS5	1.042470539
7134	1.6707246	1.602869	KIAA1109	1.04233384
7135	141.84824	136.1708	PRRC2A	1.041693495
7136	8.4898976	8.1517007	TRUB1	1.041487887

7137	7.6561567	7.3519277	SHPK	1.041380848
7138	6.7210693	6.4552691	ZBTB40	1.04117569
7139	30.253189	29.060847	SH3GLB2	1.041029149
7140	21.363229	20.521573	MAPK9	1.041013235
7141	21.617033	20.769676	COMMD5	1.040797768
7142	14.072955	13.523397	PRR12	1.040637535
7143	38.03429	36.554873	CPSF3L	1.04047113
7144	13.610601	13.096972	TXNDC11	1.039217366
7145	18.42359	17.728331	RMDN3	1.039217366
7146	19.504056	18.77668	PMM2	1.038738266
7147	8.7045511	8.3804467	AMBRA1	1.03867388
7148	34.067133	32.809045	CDH2	1.038345779
7149	10.836909	10.447969	PTPRK	1.037226403
7150	541.26593	522.08562	GNAS	1.036737861
7151	32.863506	31.740488	GLUL	1.035381221
7152	286.53346	276.99029	FKBP1A	1.034453093
7153	0.0217581	0.0210428	NRXN1	1.033995207
7154	0.0325751	0.0315042	DNAH6	1.033995196
7155	0.040364	0.039037	LOC100996295	1.03399519
7156	0.0201527	0.0194902	COL4A4	1.033995188
7157	0.0605285	0.0585384	ADIPOQ-AS1	1.033995184
7158	0.0560063	0.054165	NEFH	1.033995183
7159	0.0168731	0.0163183	DNAH3	1.033995182
7160	0.0190563	0.0184298	ALPK3	1.033995181
7161	0.026568	0.0256945	AVPR1A	1.033995181
7162	0.0758644	0.0733702	LOC401010	1.03399518
7163	0.0618213	0.0597887	C6orf132	1.03399518
7164	0.062246	0.0601995	LRRC66	1.03399518
7165	0.064922	0.0627875	FAM13A-AS1	1.033995179
7166	0.0416716	0.0403015	LOC100130872	1.033995179
7167	0.0603357	0.058352	TTLL6	1.033995178
7168	0.0683501	0.0661029	DUOXA1	1.033995178
7169	0.0456616	0.0441604	SLC6A14	1.033995177
7170	0.0587537	0.0568221	C11orf44	1.033995177
7171	0.0385354	0.0372685	GALNT4	1.033995177
7172	0.0624137	0.0603617	TVP23A	1.033995177
7173	0.0713208	0.068976	LOC283688	1.033995177
7174	0.0582122	0.0562983	LRRC37A11P	1.033995177
7175	0.0619131	0.0598776	SCG3	1.033995176
7176	0.086437	0.0835951	HYAL4	1.033995176
7177	0.0815335	0.0788528	UBASH3A	1.033995176
7178	0.0609712	0.0589666	CELF6	1.033995176
7179	0.0614748	0.0594536	POSTN	1.033995176

7180	0.0779355	0.0753732	LOC400655	1.033995175
7181	0.0748024	0.0723431	MAP4K1	1.033995175
7182	0.0907663	0.0877822	MS4A10	1.033995175
7183	0.0907663	0.0877822	SERPINF2	1.033995175
7184	0.0959924	0.0928364	ZNF681	1.033995175
7185	0.1309865	0.12668	LOC440896	1.033995175
7186	0.1155849	0.1117847	SLC16A11	1.033995175
7187	0.0774144	0.0748692	MRS2P2	1.033995174
7188	0.0688128	0.0665504	LOC100128164	1.033995174
7189	0.1035268	0.1001231	ZNF843	1.033995174
7190	0.0561421	0.0542963	ZNF418	1.033995174
7191	0.0736134	0.0711932	FGF14	1.033995174
7192	0.1368349	0.1323361	GIMAP2	1.033995174
7193	0.0624886	0.0604341	TMEM105	1.033995174
7194	0.0978401	0.0946234	PP2D1	1.033995174
7195	0.0978401	0.0946234	LOC729020	1.033995174
7196	0.0880065	0.0851131	MCHR2	1.033995174
7197	0.0665388	0.0643512	ZNF345	1.033995174
7198	0.0902162	0.0872502	RASGRP2	1.033995174
7199	0.0644003	0.062283	HEATR8-TTC4	1.033995173
7200	0.0805564	0.0779079	C9orf163	1.033995173
7201	0.0677942	0.0655653	CAGE1	1.033995173
7202	0.1337609	0.1293632	C6orf164	1.033995173
7203	0.0732254	0.0708179	RAG2	1.033995173
7204	0.0930563	0.0899968	ZNF667	1.033995173
7205	0.1412399	0.1365963	CPT1B	1.033995173
7206	0.1186785	0.1147767	PFKFB1	1.033995173
7207	0.1561045	0.1509722	ARL14	1.033995173
7208	0.0520348	0.0503241	SIAH3	1.033995173
7209	0.1377393	0.1332107	WDR63	1.033995172
7210	0.1161647	0.1123455	UBOX5-AS1	1.033995172
7211	0.1084849	0.1049182	SLC22A1	1.033995172
7212	0.2588814	0.25037	SRRM5	1.033995172
7213	0.1334184	0.1290319	LOC100129046	1.033995172
7214	0.1154568	0.1116609	LRRC73	1.033995172
7215	0.1362088	0.1317306	PRR15L	1.033995172
7216	0.131982	0.1276427	CRLF2	1.033995172
7217	0.1356768	0.131216	ERP27	1.033995172
7218	0.1061097	0.1026211	PLD4	1.033995172
7219	0.1824066	0.1764095	CES5A	1.033995171
7220	0.1335038	0.1291146	CA9	1.033995171
7221	0.2336317	0.2259505	NSUN5P2	1.033995171
7222	0.227759	0.2202709	SPDYA	1.033995171

7223	0.2094467	0.2025607	TP53TG5	1.033995171
7224	0.0487941	0.0471898	DSC1	1.033995171
7225	0.0602659	0.0582845	LGR6	1.033995171
7226	0.0655757	0.0634197	ZSCAN23	1.033995171
7227	0.1000478	0.0967584	HCG4	1.033995171
7228	0.0949861	0.0918632	RAG1	1.033995171
7229	0.2238448	0.2164853	LOC100507600	1.033995171
7230	0.1053587	0.1018948	TMEM191A	1.033995171
7231	0.2293886	0.2218468	CNTD2	1.033995171
7232	0.2450798	0.2370222	MLK7-AS1	1.033995171
7233	0.0927457	0.0896964	GNG12-AS1	1.033995171
7234	0.2210968	0.2138277	NLRC3	1.033995171
7235	0.1569667	0.1518061	CARNS1	1.033995171
7236	0.3728077	0.3605507	LOC730091	1.033995171
7237	0.1242692	0.1201836	BMPER	1.033995171
7238	0.2346841	0.2269683	PGAM2	1.033995171
7239	0.0831934	0.0804582	EXOC3L1	1.033995171
7240	0.201742	0.1951092	NPW	1.033995171
7241	0.3293986	0.3185688	NTN5	1.033995171
7242	0.1293925	0.1251384	NPAP1	1.033995171
7243	0.0444633	0.0430015	NKPD1	1.033995171
7244	0.221466	0.2141847	HNRNPKP3	1.033995171
7245	0.2434574	0.2354531	LOC144486	1.03399517
7246	0.0844749	0.0816975	SERPINA10	1.03399517
7247	0.1037846	0.1003724	RPL17-C18orf32	1.03399517
7248	0.2512351	0.2429751	CNTD1	1.03399517
7249	0.3780245	0.365596	ATAD3C	1.03399517
7250	0.0355813	0.0344114	VWA2	1.03399517
7251	0.1754205	0.1696531	SH2D3A	1.03399517
7252	0.1689497	0.1633951	HIPK4	1.03399517
7253	0.2155114	0.2084259	RDH12	1.03399517
7254	0.1829671	0.1769516	C12orf60	1.03399517
7255	0.2998554	0.2899969	LCE1C	1.03399517
7256	0.6251985	0.6046435	WFDC3	1.03399517
7257	0.3126774	0.3023974	MYLK3	1.03399517
7258	0.4651775	0.4498836	HIST2H2AB	1.03399517
7259	0.1763109	0.1705143	THRSP	1.03399517
7260	0.3526218	0.3410285	FCER1G	1.03399517
7261	0.3039001	0.2939086	LOC100128338	1.03399517
7262	0.6105454	0.5904722	LOC100507634	1.03399517
7263	0.0420754	0.0406921	SLC4A1	1.03399517
7264	0.1559877	0.1508592	C22orf43	1.03399517
7265	0.5678461	0.5491767	HIST1H4D	1.03399517

7266	0.1135692	0.1098353	PTCD1	1.03399517
7267	0.1135692	0.1098353	CCDC74B-AS1	1.03399517
7268	0.5529934	0.5348123	LOC100130954	1.03399517
7269	0.4003833	0.3872197	EPHA10	1.03399517
7270	0.3964983	0.3834624	DAPK2	1.03399517
7271	0.3133827	0.3030795	TSSK4	1.03399517
7272	0.4288056	0.4147075	NCRUPAR	1.03399517
7273	0.0771279	0.0745921	KCNH1	1.03399517
7274	0.3329066	0.3219614	PPIAP30	1.03399517
7275	0.1454796	0.1406966	ENO4	1.03399517
7276	0.2918761	0.2822799	ANKRD61	1.03399517
7277	0.197348	0.1908597	LOC100289561	1.03399517
7278	0.0704886	0.0681711	NUP210L	1.03399517
7279	0.1542557	0.1491842	SPIN2A	1.03399517
7280	0.4872942	0.4712732	LOC100507577	1.03399517
7281	0.2004805	0.1938892	STAU2-AS1	1.033995169
7282	0.141672	0.1370142	ABHD1	1.033995169
7283	0.272062	0.2631173	TTC25	1.033995169
7284	0.2415759	0.2336335	HYAL1	1.033995169
7285	0.1348864	0.1304517	RASA4CP	1.033995169
7286	0.2894438	0.2799276	C1orf189	1.033995169
7287	0.1736663	0.1679565	SGPP2	1.033995169
7288	0.1395844	0.1349952	FLJ25363	1.033995169
7289	0.5942952	0.5747562	FAM132A	1.033995169
7290	0.1630669	0.1577057	TCTEX1D4	1.033995169
7291	0.8683313	0.8397827	LOC100287177	1.033995169
7292	0.2067455	0.1999483	CCDC73	1.033995169
7293	0.195987	0.1895434	ZNF235	1.033995169
7294	0.5070547	0.4903841	QRFP	1.033995169
7295	0.1455304	0.1407457	FAM66B	1.033995169
7296	0.1017079	0.098364	TRIM31	1.033995169
7297	0.6524718	0.6310202	CHMP3	1.033995169
7298	0.1240473	0.119969	LOC727924	1.033995169
7299	0.1904061	0.184146	TFAP2E	1.033995169
7300	0.0565535	0.0546941	IGF1	1.033995169
7301	0.1815327	0.1755643	APOD	1.033995169
7302	1.2126322	1.1727639	C20orf196	1.033995169
7303	0.6263617	0.6057685	MTCP1	1.033995169
7304	0.1206714	0.116704	KIAA1984	1.033995169
7305	0.1038363	0.1004224	LIN28A	1.033995169
7306	0.5154151	0.4984695	SIRT4	1.033995169
7307	0.1492833	0.1443752	ZBTB7C	1.033995169
7308	0.6544401	0.6329237	LOC100131257	1.033995169

7309	0.0961696	0.0930078	LOC100653515	1.033995169
7310	0.1111464	0.1074922	DNAJB13	1.033995169
7311	0.1111464	0.1074922	FAM181B	1.033995169
7312	0.250681	0.2424393	TMC4	1.033995169
7313	0.6179886	0.5976707	ZNF136	1.033995169
7314	0.3283359	0.3175411	ANKAR	1.033995169
7315	0.4992668	0.4828521	ANKRD20A9P	1.033995169
7316	0.6512484	0.629837	GPR68	1.033995169
7317	0.3723636	0.3601212	COX6B2	1.033995169
7318	0.7923935	0.7663416	FLJ27354	1.033995169
7319	0.995539	0.9628082	LOC100144603	1.033995169
7320	0.2003841	0.193796	TTLL3	1.033995169
7321	0.2003841	0.193796	DPPA3	1.033995169
7322	1.8309	1.7707046	ZBTB26	1.033995169
7323	0.8975	0.8679925	HOXA-AS4	1.033995169
7324	0.3929101	0.3799922	LOC644961	1.033995169
7325	12.048233	11.652117	ALG12	1.033995169
7326	0.1738836	0.1681667	LOC100506207	1.033995169
7327	0.4796306	0.4638616	EPS8L1	1.033995169
7328	0.3398742	0.3286999	GLUD1P7	1.033995169
7329	0.2835367	0.2742148	ADAMTS4	1.033995169
7330	0.2624679	0.2538386	LOC389247	1.033995169
7331	0.9580291	0.9265315	ZNF554	1.033995169
7332	0.5361908	0.5185622	RDM1	1.033995169
7333	0.3656132	0.3535927	KATNAL2	1.033995169
7334	2.7360985	2.6461424	HIST2H3D	1.033995169
7335	0.3020283	0.2920983	GSTTP2	1.033995169
7336	0.3902612	0.3774304	RDH14	1.033995169
7337	10.714325	10.362064	AFMID	1.033995169
7338	0.6693774	0.64737	TNFRSF13C	1.033995169
7339	0.2549229	0.2465417	HPX	1.033995169
7340	1.169589	1.1311359	BMS1P4	1.033995169
7341	0.8369458	0.8094291	HIST1H2AG	1.033995169
7342	0.1076444	0.1041053	CNBD2	1.033995169
7343	0.0594408	0.0574866	SLA	1.033995169
7344	1.9330342	1.8694809	SLC25A16	1.033995169
7345	0.2260298	0.2185985	WAS	1.033995169
7346	4.5322485	4.3832395	GALM	1.033995169
7347	2.1708281	2.0994567	RNY4	1.033995169
7348	0.102458	0.0990894	LOC100270804	1.033995169
7349	2.3389394	2.2620409	ZNF783	1.033995169
7350	46.747896	45.210943	SSNA1	1.033995169
7351	0.3618047	0.3499095	LOC284009	1.033995169

7352	12.715644	12.297585	ZC3H12A	1.033995169
7353	1.6082277	1.5553532	DUSP28	1.033995169
7354	3.6019667	3.483543	FLJ20021	1.033995169
7355	1.4057302	1.3595133	HERC2P7	1.033995169
7356	32.696424	31.621447	HERPUD1	1.033995169
7357	2.0633614	1.9955232	HFE	1.033995169
7358	0.4801832	0.464396	ZNRF3-AS1	1.033995169
7359	0.2912642	0.2816881	LOC100289511	1.033995169
7360	1.9053669	1.8427232	MCEE	1.033995169
7361	0.1885968	0.1823962	CTSL1P2	1.033995169
7362	0.4214348	0.4075791	MFAP4	1.033995169
7363	4.9279654	4.7659462	SLC12A8	1.033995169
7364	5.7525583	5.5634287	CDKN2AIP	1.033995169
7365	5.2854946	5.1117208	C21orf119	1.033995169
7366	0.6065178	0.586577	LOC100129518	1.033995169
7367	1.3485731	1.3042354	PGBD4	1.033995169
7368	1.1504704	1.1126458	PHOSPHO2	1.033995169
7369	1.4487278	1.4010973	LINC00842	1.033995169
7370	7.1639345	6.9284023	CWC25	1.033995169
7371	4.8092192	4.6511042	SNORA61	1.033995169
7372	0.6465341	0.6252777	ZNF700	1.033995169
7373	2.4809464	2.3993791	RNY5	1.033995169
7374	6.6401802	6.4218677	CCDC23	1.033995169
7375	0.4671682	0.4518089	ZC3H12D	1.033995169
7376	0.3399666	0.3287893	FLJ14107	1.033995169
7377	4.8111671	4.652988	C9orf156	1.033995169
7378	3.7516751	3.6283294	XPA	1.033995168
7379	3.3787622	3.2676769	IPP	1.033995168
7380	4.8465	4.6871592	LOC642852	1.033995168
7381	4.8465	4.6871592	SNORD35A	1.033995168
7382	1.5552202	1.5040884	SNORA52	1.033995168
7383	1.5552202	1.5040884	SNORA16A	1.033995168
7384	0.9322758	0.9016249	C14orf28	1.033995168
7385	0.1520609	0.1470615	LOC284551	1.033995168
7386	0.2787953	0.2696292	C1orf210	1.033995168
7387	1.5566723	1.5054928	H3F3C	1.033995168
7388	0.2858704	0.2764717	MPL	1.033995168
7389	21.088773	20.395427	SLC35E1	1.033995168
7390	9.1103607	8.8108348	IFI27L1	1.033995168
7391	4.1191039	3.9836781	ASTN2	1.033995168
7392	0.0725878	0.0702013	GRIK4	1.033995168
7393	0.4004602	0.3872941	LOC646999	1.033995168
7394	0.4539195	0.4389958	C21orf62	1.033995168

7395	7.6058212	7.3557608	SNORA67	1.033995168
7396	2.5876923	2.5026155	KIF18A	1.033995168
7397	93.628761	90.550482	PSMA6	1.033995168
7398	2.7020017	2.6131667	LIFR	1.033995168
7399	1.3217727	1.2783162	HIST1H3H	1.033995168
7400	0.6636927	0.6418721	DKFZP686I15217	1.033995168
7401	2.0486731	1.9813178	FER	1.033995168
7402	0.6862018	0.6636412	POLI	1.033995168
7403	5.1909961	5.0203291	C5orf55	1.033995168
7404	7.3291819	7.0882167	NEK2	1.033995168
7405	0.1232404	0.1191886	ZNF582-AS1	1.033995168
7406	2.7343889	2.6444891	MORC2-AS1	1.033995168
7407	0.2083995	0.2015478	C16orf3	1.033995168
7408	3.8316558	3.7056805	HIBCH	1.033995168
7409	1.5669135	1.5153973	SNORA68	1.033995168
7410	0.4415244	0.4270082	HIST1H3B	1.033995168
7411	3.94447	3.8147858	TMEM212	1.033995168
7412	1.7512563	1.6936794	RNU5E-1	1.033995168
7413	7.7884298	7.5323657	LOC148413	1.033995168
7414	0.3917284	0.3788493	LCTL	1.033995168
7415	1.2569331	1.2156082	DBP	1.033995168
7416	0.368197	0.3560916	LINC00568	1.033995168
7417	2.083995	2.0154785	RNU5D-1	1.033995168
7418	2.1583377	2.087377	PMS2CL	1.033995168
7419	0.101857	0.0985082	LOC341056	1.033995168
7420	0.1708193	0.1652032	OTX2-AS1	1.033995168
7421	2.1613223	2.0902635	CCDC142	1.033995168
7422	0.9165454	0.8864116	AARD	1.033995168
7423	0.4747141	0.4591067	LINC00115	1.033995168
7424	0.3164761	0.3060711	ACY3	1.033995168
7425	0.4191041	0.405325	SYTL1	1.033995168
7426	2.5205708	2.4377007	MEF2C	1.033995168
7427	0.6682531	0.6462826	METTL20	1.033995168
7428	0.5343577	0.5167894	FAM215A	1.033995168
7429	1.0459197	1.0115325	HOXB-AS1	1.033995168
7430	11.094043	10.729299	LZIC	1.033995168
7431	3.0228596	2.9234756	BLZF1	1.033995168
7432	0.5262614	0.5089592	PFN4	1.033995168
7433	1.5787841	1.5268776	SNORA18	1.033995168
7434	2.3681761	2.2903164	MIR1227	1.033995168
7435	1.9293623	1.8659297	MID2	1.033995168
7436	0.1295211	0.1252628	LOC100507053	1.033995168
7437	3.1816718	3.0770664	LOC439994	1.033995168

7438	0.3734758	0.3611969	FXYD1	1.033995168
7439	2.9376381	2.8410559	KAT2B	1.033995168
7440	0.0886806	0.085765	UBQLNL	1.033995168
7441	0.1496048	0.1446862	C9orf66	1.033995168
7442	0.326645	0.3159057	MEIG1	1.033995168
7443	0.5633803	0.5448578	GLUD1P3	1.033995168
7444	14.854472	14.366094	CENPA	1.033995168
7445	1.9516488	1.8874835	CDADC1	1.033995168
7446	1.2258794	1.1855756	LOC100130987	1.033995168
7447	1.6616571	1.607026	OSCP1	1.033995168
7448	0.1034497	0.1000486	ZNF204P	1.033995168
7449	0.3615536	0.3496666	TBX19	1.033995168
7450	0.3377626	0.3266578	MIR181A2HG	1.033995168
7451	0.8037003	0.7772767	ZNF432	1.033995168
7452	0.1575204	0.1523415	ZFP42	1.033995168
7453	0.8093184	0.7827101	BOLA2	1.033995168
7454	1.2372002	1.1965242	LYZ	1.033995168
7455	0.2179911	0.2108241	LOC100216479	1.033995168
7456	1.6539643	1.5995861	SNORD22	1.033995168
7457	0.1617695	0.1564509	LOC400891	1.033995168
7458	0.0724616	0.0700792	TYRP1	1.033995168
7459	1.2565	1.2151894	ZKSCAN7	1.033995168
7460	1.0215662	0.9879796	C7orf61	1.033995168
7461	0.2466266	0.2385182	PTK6	1.033995168
7462	0.7939029	0.7678013	LOC100128573	1.033995168
7463	0.407561	0.3941614	DFNB59	1.033995168
7464	0.4193149	0.4055289	ZNF487P	1.033995168
7465	0.4560164	0.4410237	HP07349	1.033995168
7466	0.1140041	0.1102559	KRT8P41	1.033995168
7467	1.5035096	1.4540779	SPRN	1.033995168
7468	0.3820339	0.3694736	GNMT	1.033995168
7469	1.3384682	1.2944627	CCDC74A	1.033995168
7470	1.1937778	1.1545294	ANG	1.033995168
7471	1.4519	1.4041651	BBS9	1.033995168
7472	0.0700738	0.06777	SFRP4	1.033995168
7473	0.1922505	0.1859297	GGN	1.033995168
7474	0.0775873	0.0750364	C14orf182	1.033995168
7475	0.1167504	0.112912	ATP2A1	1.033995168
7476	1.132606	1.0953687	DKFZP586I1420	1.033995168
7477	0.0817787	0.0790901	NWD1	1.033995168
7478	0.1949481	0.1885387	CYP46A1	1.033995168
7479	0.1029642	0.099579	IGSF9	1.033995168
7480	0.9021623	0.8725015	LUST	1.033995168

7481	0.1997599	0.1931923	MYO1F	1.033995168
7482	0.0607756	0.0587774	LOC339524	1.033995168
7483	0.5744198	0.5555343	TMEFF2	1.033995168
7484	0.1013616	0.0980291	LINC00637	1.033995168
7485	12.895439	12.471469	TRIM11	1.033995168
7486	0.1498199	0.1448942	PRSS22	1.033995168
7487	0.3609691	0.3491014	DAND5	1.033995168
7488	0.6360107	0.6151002	NEK5	1.033995168
7489	0.0806812	0.0780286	MYCBP	1.033995168
7490	0.3361282	0.3250772	RPL23AP32	1.033995168
7491	0.4253051	0.4113221	LOC729739	1.033995168
7492	0.059799	0.057833	HLA-DOA	1.033995168
7493	0.4237485	0.4098167	NLGN1	1.033995168
7494	0.0848705	0.0820802	C9orf47	1.033995168
7495	0.1399594	0.1353579	CCBP2	1.033995168
7496	0.2492817	0.2410859	C6orf123	1.033995168
7497	0.5788875	0.5598551	RPSAP9	1.033995168
7498	0.1929625	0.1866184	C8orf74	1.033995168
7499	0.1195637	0.1156327	LINC00221	1.033995168
7500	0.2727742	0.2638061	LOC100507118	1.033995168
7501	0.6392623	0.6182449	UNC5B-AS1	1.033995168
7502	0.6392623	0.6182449	ASAH2B	1.033995168
7503	0.5375739	0.5198999	IL18	1.033995168
7504	0.5435563	0.5256856	LOC653160	1.033995168
7505	0.1007247	0.0974132	FFAR2	1.033995168
7506	0.3924661	0.3795628	TEX21P	1.033995168
7507	0.1097995	0.1061896	RAD9B	1.033995167
7508	0.1599791	0.1547194	LOC100289495	1.033995167
7509	0.2682104	0.2593923	B3GNT4	1.033995167
7510	0.1683356	0.1628012	CORT	1.033995167
7511	0.3366712	0.3256023	SCGB1B2P	1.033995167
7512	0.0678605	0.0656294	MALL	1.033995167
7513	0.2598498	0.2513065	HIATL2	1.033995167
7514	0.5971332	0.577501	MRPL45P2	1.033995167
7515	0.1940405	0.1876609	LOC643669	1.033995167
7516	0.5209988	0.5038696	PRR7-AS1	1.033995167
7517	0.1223002	0.1182793	NEURL3	1.033995167
7518	0.3669005	0.3548378	ADAM1A	1.033995167
7519	0.2812409	0.2719944	C20orf141	1.033995167
7520	0.2580266	0.2495434	PRODH	1.033995167
7521	0.6382833	0.6172982	RPL13AP6	1.033995167
7522	0.1010667	0.0977439	SHE	1.033995167
7523	0.5281667	0.5108019	ISPD	1.033995167

7524	0.2162942	0.209183	HMGN2P46	1.033995167
7525	0.4792997	0.4635415	DDX39B	1.033995167
7526	0.2135241	0.2065039	LOC100289509	1.033995167
7527	0.0572841	0.0554007	RUFY4	1.033995167
7528	0.0667305	0.0645366	LOC100505776	1.033995167
7529	0.1720888	0.1664309	CRTAM	1.033995167
7530	0.0429247	0.0415135	FAT2	1.033995167
7531	0.3811024	0.3685727	ANP32A-IT1	1.033995167
7532	0.2453683	0.2373012	ZDHHC8P1	1.033995167
7533	0.2417628	0.2338142	WDR88	1.033995167
7534	0.4136279	0.4000288	CCDC157	1.033995167
7535	0.2541457	0.2457901	PATE2	1.033995167
7536	0.0658867	0.0637205	PROSER2-AS1	1.033995167
7537	0.162685	0.1573363	SULT2B1	1.033995167
7538	0.0775007	0.0749527	HHLA2	1.033995167
7539	0.1681998	0.1626698	GLTPD2	1.033995167
7540	0.3228497	0.3122352	HHIPL2	1.033995167
7541	0.3269867	0.3162362	DNAJC28	1.033995167
7542	0.2285709	0.221056	SLC9A9	1.033995167
7543	0.2032175	0.1965362	LOC152225	1.033995167
7544	0.1880862	0.1819024	LOC284260	1.033995166
7545	0.0922938	0.0892595	GNN	1.033995166
7546	0.1877473	0.1815746	LOC442028	1.033995166
7547	0.237267	0.2294662	GGT6	1.033995166
7548	0.1705397	0.1649328	LINC00578	1.033995166
7549	0.132654	0.1282927	SLC15A1	1.033995166
7550	0.2536817	0.2453413	APOBEC2	1.033995166
7551	0.1951306	0.1887152	ZNF790-AS1	1.033995166
7552	0.0935366	0.0904613	LOC100130894	1.033995166
7553	0.1870732	0.1809227	CRYAA	1.033995166
7554	0.1073394	0.1038104	NLRP12	1.033995166
7555	0.2553915	0.2469949	HAVCR2	1.033995166
7556	0.115329	0.1115373	C3orf35	1.033995166
7557	0.074455	0.0720071	ASB11	1.033995166
7558	0.14891	0.1440142	CD300LG	1.033995166
7559	0.1706794	0.1650679	LOC100133286	1.033995166
7560	0.3782205	0.3657856	FLJ31662	1.033995166
7561	0.0695825	0.0672948	VSX2	1.033995166
7562	0.0963029	0.0931367	GSDMA	1.033995166
7563	0.2814308	0.2721781	PAPL	1.033995166
7564	0.2695983	0.2607346	CCDC153	1.033995166
7565	0.2307857	0.2231981	CAHM	1.033995166
7566	0.1089386	0.105357	CACTIN-AS1	1.033995166

7567	0.1959256	0.189484	XKR9	1.033995166
7568	0.2717073	0.2627742	ZNF717	1.033995165
7569	0.108768	0.105192	HPD	1.033995165
7570	0.0578566	0.0559544	CLDN19	1.033995165
7571	0.1620525	0.1567246	RTDR1	1.033995165
7572	0.1536869	0.1486341	MMD2	1.033995165
7573	0.0241036	0.0233111	CACNA1A	1.033995165
7574	0.2710007	0.2620908	LECT1	1.033995165
7575	0.1574014	0.1522265	LINC00598	1.033995165
7576	0.134625	0.1301989	CD72	1.033995165
7577	0.1008222	0.0975074	FOXD4L1	1.033995165
7578	0.0685525	0.0662986	RS1	1.033995165
7579	0.0454326	0.0439389	STEAP4	1.033995165
7580	0.0857611	0.0829415	HRC	1.033995165
7581	0.0710534	0.0687173	LINC00472	1.033995165
7582	0.1932309	0.1868779	SPATA18	1.033995165
7583	0.1999995	0.193424	KLRC3	1.033995165
7584	0.1141915	0.1104372	LOC643770	1.033995165
7585	0.1086546	0.1050823	HTR3B	1.033995164
7586	0.0799384	0.0773103	SKOR1	1.033995164
7587	0.0918565	0.0888365	PKD1L1	1.033995164
7588	0.1663868	0.1609164	PBOV1	1.033995164
7589	0.1220852	0.1180714	PDE6B	1.033995164
7590	0.1279309	0.1237249	IL10	1.033995164
7591	0.0900603	0.0870993	EREG	1.033995164
7592	0.1378304	0.1332988	MS4A7	1.033995164
7593	0.1046445	0.101204	CCDC85A	1.033995164
7594	0.0915639	0.0885535	TPRXL	1.033995164
7595	0.0965707	0.0933957	ACSM3	1.033995164
7596	0.0501201	0.0484723	MAP7D2	1.033995164
7597	0.1952677	0.1888478	TTC21A	1.033995164
7598	0.0463935	0.0448682	C1orf140	1.033995164
7599	0.1621786	0.1568466	C19orf35	1.033995164
7600	0.0386498	0.0373791	GPR133	1.033995164
7601	0.0833265	0.0805869	C1orf204	1.033995164
7602	0.0398927	0.0385811	POM121L9P	1.033995164
7603	0.1458359	0.1410412	CBX3P2	1.033995163
7604	0.0874342	0.0845596	PCDH11X	1.033995163
7605	0.1677935	0.1622769	CMAHP	1.033995163
7606	0.0779938	0.0754296	SLAMF7	1.033995163
7607	0.0749638	0.0724992	CDRT1	1.033995163
7608	0.078023	0.0754578	ANKRD45	1.033995163
7609	0.078023	0.0754578	LOC440600	1.033995163

7610	0.0302203	0.0292268	KIAA0319	1.033995163
7611	0.0993798	0.0961125	LOC644669	1.033995162
7612	0.0697221	0.0674299	SPAG6	1.033995162
7613	0.0881741	0.0852752	CD36	1.033995162
7614	0.1165545	0.1127225	HLA-DPA1	1.033995162
7615	0.078671	0.0760845	LRRC10	1.033995162
7616	0.1208113	0.1168393	FLJ35390	1.033995162
7617	0.0903336	0.0873636	PRR23A	1.033995161
7618	0.0842018	0.0814335	FAM92A1P2	1.03399516
7619	0.0944266	0.0913221	WDR16	1.03399516
7620	0.0431648	0.0417456	HNF4A	1.03399516
7621	0.0981627	0.0949354	LOC100130899	1.03399516
7622	0.1021066	0.0987496	LOC340515	1.03399516
7623	0.112103	0.1084173	LOC145474	1.033995159
7624	0.0570021	0.055128	MUC21	1.033995159
7625	0.0329746	0.0318905	KLHL6	1.033995159
7626	0.0733629	0.0709509	TRIM67	1.033995159
7627	0.0873243	0.0844533	DCDC5	1.033995159
7628	0.0338201	0.0327082	TNNI1	1.033995158
7629	0.0676622	0.0654376	LOC399715	1.033995158
7630	0.0693278	0.0670485	HNRNPA3P1	1.033995158
7631	0.0349605	0.0338111	NDST3	1.033995158
7632	0.0414808	0.040117	NPHS1	1.033995157
7633	0.0359309	0.0347496	MYOM3	1.033995154
7634	0.0210952	0.0204016	UNC5C	1.033995151
7635	0.0233344	0.0225672	DCHS2	1.033995147
7636	0.0337872	0.0326764	CACNA1S	1.033995147
7637	0.0333333	0.0322373	AMER3	1.033995144
7638	0.0110364	0.0106735	USH2A	1.033995103
7639	47.924293	46.440442	TERF2IP	1.0319517
7640	18.908386	18.342992	NSMAF	1.030823404
7641	12.148288	11.789257	SPECC1	1.030454089
7642	10.924488	10.603737	GOSR1	1.030248809
7643	21.643031	21.00913	MBD2	1.030172635
7644	23.041485	22.374157	C16orf45	1.029825833
7645	13.511616	13.122758	WFS1	1.02963232
7646	22.196396	21.558372	MICA	1.029595189
7647	11.767549	11.433107	PAGR1	1.029252072
7648	98.866968	96.091499	PDIA4	1.02888361
7649	102.41812	99.543172	MORF4L2	1.028881444
7650	10.904303	10.599329	CTDP1	1.028772971
7651	43.18459	41.983071	SMARCD2	1.028619111
7652	22.39169	21.772144	EPAS1	1.028455909

7653	16.820817	16.363766	SNX9	1.02793068
7654	57.367115	55.81325	XBP1	1.027840435
7655	19.285848	18.764956	ARHGAP35	1.027758768
7656	62.225795	60.546918	VMP1	1.027728531
7657	14.993824	14.595333	PEX26	1.027302643
7658	4.1111513	4.0026714	AIM1	1.027101867
7659	8.2612889	8.0444023	AP3M2	1.026961188
7660	56.744924	55.267819	HMGA2	1.02672631
7661	30.462618	29.673799	MSANTD3	1.026583017
7662	40.730542	39.6876	E2F1	1.026278787
7663	7.0958447	6.91534	SCYL2	1.026102076
7664	6.9984907	6.8221153	MAP2K4	1.025853474
7665	42.730433	41.655762	KIF1C	1.025798865
7666	7.7438239	7.5507396	VPS53	1.025571583
7667	17.896621	17.451269	RUSC2	1.025519798
7668	120.38833	117.42257	BCAP31	1.025257181
7669	70.886253	69.144152	EMD	1.02519521
7670	64.533598	62.955395	VAMP3	1.025068592
7671	0.1349738	0.1316876	SPOCK3	1.02495441
7672	53.172386	51.891704	ALKBH5	1.024679897
7673	12.079494	11.791532	FAM219A	1.024421139
7674	45.160299	44.086026	AKT1S1	1.024367653
7675	106.69129	104.21624	PRPF19	1.023749138
7676	18.77388	18.339121	PLEKHG3	1.02370666
7677	65.528827	64.021081	MRPL24	1.023550773
7678	6.4580268	6.3117952	IFNAR1	1.023167994
7679	59.211668	57.883182	BAG3	1.022951161
7680	108.32064	105.89802	ID1	1.022876941
7681	6.0199791	5.8874736	KCTD11	1.022506333
7682	69.085862	67.587552	LSM4	1.022168426
7683	31.82535	31.153985	GBF1	1.021549891
7684	3.4031353	3.3323894	GOLGA1	1.021229796
7685	28.798695	28.2078	DIABLO	1.020947911
7686	5.01393	4.9112523	ELF2	1.020906622
7687	12.114143	11.866064	HIP1	1.020906622
7688	11.900092	11.662884	ABHD2	1.020338629
7689	64.152355	62.906611	MPDU1	1.019803078
7690	7.9153163	7.7628985	SH3RF3	1.019634124
7691	4.1907397	4.1108581	PPP1R3D	1.019431856
7692	4.4394294	4.3548074	CD3EAP	1.019431856
7693	73.656918	72.262695	RRP36	1.019293815
7694	16.965595	16.64446	NR2F2	1.019293815
7695	7.5325121	7.3904397	MYPOP	1.019223809

7696	9.2058678	9.0322339	ASPHD1	1.019223809
7697	3.0549321	2.9973124	ARMC5	1.019223809
7698	16.974276	16.655857	PPA2	1.01911754
7699	38.467251	37.747636	TACO1	1.01906383
7700	48.47709	47.574593	PRC1	1.01897014
7701	12.58226	12.348862	MFSD2A	1.018900349
7702	64.254143	63.076502	FOXM1	1.018670044
7703	12.319068	12.094222	CCDC50	1.018591144
7704	154.04891	151.2415	NEDD8	1.018562405
7705	24.030669	23.593265	CDC42EP3	1.018539336
7706	16.110837	15.818129	CD46	1.018504604
7707	17.081926	16.774474	MYC	1.018328575
7708	7.1668823	7.039554	LIG3	1.01808755
7709	13.335875	13.102145	MFSD10	1.017838994
7710	31.806361	31.270896	SETD8	1.017123453
7711	21.068338	20.718112	MAVS	1.016904339
7712	9.2378441	9.0855528	ALKBH7	1.016761916
7713	9.2457631	9.0950733	OPA3	1.016568284
7714	4.1751886	4.1075382	C12orf29	1.016469827
7715	15.981275	15.722331	FBXO5	1.016469826
7716	18.930444	18.62646	APIP	1.016320037
7717	65.401212	64.3546	MGAT4B	1.016263204
7718	2.2738843	2.2377058	SESTD1	1.016167666
7719	2.5829031	2.541808	C17orf75	1.016167665
7720	5.9330819	5.8404816	PAWR	1.015854902
7721	5.1366074	5.0564381	ADRA1B	1.015854902
7722	27.969702	27.538006	FBXO18	1.015676357
7723	3.6329548	3.5773944	PGBD1	1.015530969
7724	2.5420314	2.5039827	RECK	1.015195256
7725	113.59723	111.92022	PSMD13	1.014984021
7726	3.2524068	3.2048244	KCTD21	1.01484711
7727	5.4924567	5.4130571	SKAP2	1.014668156
7728	3.5287444	3.4783575	SCRN3	1.014485826
7729	119.23662	117.53404	SHMT2	1.014485826
7730	1.904195	1.8770051	ATXN7L1	1.014485825
7731	1409.785	1389.6672	EEF1G	1.01447671
7732	9.2317322	9.1050124	PANK2	1.013917592
7733	7.4798683	7.3771955	BBS2	1.013917592
7734	6.0264021	5.9464097	UNKL	1.013452218
7735	83.384075	82.285988	SMARCB1	1.013344766
7736	295.60213	291.76931	HDGF	1.013136476
7737	5.1277664	5.061782	MAST3	1.013035807
7738	48.194462	47.574292	CKS2	1.013035807

7739	14.968777	14.776675	GNPAT	1.013000343
7740	9.4592681	9.3388601	CDC25C	1.012893226
7741	1.5209329	1.5015728	ZBTB34	1.012893226
7742	8.2262961	8.1269293	TOM1L1	1.012226849
7743	40.717695	40.230131	ABCF2	1.012119389
7744	2.7891699	2.7561096	RABGAP1L	1.011995271
7745	53.719713	53.092876	ERI3	1.011806431
7746	13.078984	12.927592	PRKD2	1.01171079
7747	20.218421	19.988216	PPME1	1.011517013
7748	41.695371	41.222295	LRRC47	1.011476218
7749	12.025113	11.88935	STAM	1.011418855
7750	16.547542	16.364482	TBCC	1.011186451
7751	19.743633	19.526297	DCAF8	1.01113041
7752	37.494471	37.105043	TWIST2	1.010495278
7753	3.6345422	3.5980802	WDR7	1.010133741
7754	59.2026	58.637683	SCAMP2	1.009634026
7755	136.90559	135.61299	DDX5	1.00953156
7756	17.892511	17.735062	SPATA20	1.008877877
7757	57.397296	56.902946	EMC4	1.008687594
7758	267.77144	265.51906	COX5B	1.008482951
7759	26.220028	26.01597	ADIPOR2	1.007843539
7760	9.8416201	9.7652744	FAM120B	1.007818076
7761	2.7904091	2.769685	ZSCAN26	1.007482472
7762	4.4130293	4.3802541	BICD1	1.007482472
7763	11.090127	11.01229	IFRD1	1.007068211
7764	57.927997	57.526538	SUCLG1	1.006978668
7765	7.450129	7.4025899	TRAIP	1.006421964
7766	26.846393	26.677661	PPFIA1	1.006324875
7767	9.6761009	9.6168912	RHOBTB2	1.006156837
7768	4.1889347	4.1637467	LOC389906	1.006049353
7769	79.496423	79.038371	VPS25	1.0057953
7770	14.708609	14.624606	COG8	1.005743935
7771	24.356156	24.218918	PLEKHJ1	1.005666534
7772	7.3880759	7.3473964	FBXO46	1.005536586
7773	56.497051	56.185973	IK	1.005536586
7774	4.4313381	4.4080939	NME7	1.005273081
7775	263.19157	261.87465	YWHAZ	1.005028821
7776	40.618149	40.41703	EXT1	1.004976102
7777	38.232358	38.044026	C19orf70	1.00495036
7778	2.676762	2.6637932	PAN2	1.004868544
7779	14.563267	14.49629	GFER	1.004620306
7780	16.54425	16.468162	PRR14	1.004620306
7781	7.4467504	7.4137411	FZD6	1.004452449

7782	1.5033125	1.497287	FGD6	1.004024294
7783	111.02433	110.59375	ID3	1.003893417
7784	11.755683	11.721172	PPP1R12A	1.002944263
7785	13.571084	13.53314	SSR1	1.00280376
7786	2.6947304	2.6875761	SETBP1	1.002661982
7787	7.051318	7.0325974	VGLL4	1.002661981
7788	0.5859576	0.584402	PTPN4	1.002661981
7789	44.597701	44.487143	DDX42	1.002485158
7790	10.04527	10.02342	C1orf35	1.002179933
7791	8.7843323	8.7673823	CLIP1	1.001933303
7792	38.219392	38.152784	FAM168B	1.001745807
7793	5.5741022	5.5647378	THAP9-AS1	1.001682819
7794	5.3369554	5.3279894	ZNF385A	1.001682819
7795	5.3791711	5.3701341	NEIL3	1.001682819
7796	3.71565	3.7106677	CYP1B1	1.001342689
7797	3.0383292	3.0347788	FTSJD1	1.001169925
7798	18.000443	17.980749	PPP2R2D	1.001095323
7799	30.609249	30.57784	RNF7	1.001027207
7800	39.706072	39.676958	RNF145	1.000733785
7801	14.234939	14.225827	PPIE	1.000640486
7802	4.0942927	4.0916721	PSTPIP2	1.000640486
7803	1.3786075	1.3777251	FIGN	1.000640486
7804	2.998554	2.9966347	SCNM1	1.000640485
7805	2.3805558	2.3803328	PRDM1	1.000093688
7806	1.2846694	1.284549	SHROOM4	1.000093687
7807	29.448846	29.451021	IST1	0.999926135
7808	8.7507412	8.7528688	TBC1D22B	0.999756918
7809	11.376371	11.379876	SRPK2	0.999692012
7810	89.610632	89.675965	YWHAH	0.99927145
7811	11.480481	11.490066	SNX1	0.999165858
7812	7.4638841	7.4717706	FAM222A	0.998944485
7813	120.10811	120.26201	SF3B5	0.99872028
7814	288.19401	288.57054	TMA7	0.998695191
7815	9.3801094	9.3924321	TAOK3	0.998688016
7816	282.94074	283.36155	SF3B2	0.998514942
7817	6.8009161	6.8122233	MSRA	0.998340163
7818	2.4404793	2.4445368	FAM46B	0.998340162
7819	7.0600647	7.0747408	CCDC102A	0.99792557
7820	25.700881	25.756114	PIK3R2	0.997855532
7821	0.7926127	0.7944283	UBN2	0.997714637
7822	6.7423368	6.7577808	C15orf40	0.997714636
7823	183.41728	183.87133	CCT3	0.997530604
7824	6.4388693	6.4549989	RPAIN	0.997501221

7825	3.0025542	3.0113873	PECR	0.99706677
7826	5.2390936	5.2545063	HMX3	0.99706677
7827	3.0851429	3.094219	CYP20A1	0.99706677
7828	26.171856	26.254673	HEBP2	0.996845642
7829	8.535314	8.5623227	PDE8A	0.996845641
7830	36.322353	36.445471	DDX49	0.996621849
7831	34.108906	34.226905	SLC52A2	0.996552446
7832	41.900429	42.046263	RWDD1	0.996531575
7833	4.9730633	4.9910543	NDUFAF7	0.996395344
7834	131.72307	132.21069	PRDX6	0.996311789
7835	5.282015	5.3018539	KLF9	0.996258118
7836	8.9281782	8.9625398	BPHL	0.996166077
7837	78.569616	78.892563	SGTA	0.995906506
7838	10.229863	10.272928	GAPVD1	0.995807847
7839	3.3052381	3.3195152	TRO	0.995699051
7840	1.5147853	1.5213285	JRKL	0.995699051
7841	4.7950327	4.815745	GPATCH1	0.995699051
7842	4.2310408	4.2524028	CABLES1	0.994976483
7843	53.717416	53.988629	WDR74	0.994976483
7844	30.826029	30.981666	NPC1	0.994976483
7845	8.0113111	8.0537582	EVA1C	0.994729529
7846	22.953885	23.076658	CHAF1B	0.994679763
7847	5.4630793	5.4924176	INPP4A	0.994658396
7848	94.898303	95.422088	ATXN2L	0.994510866
7849	0.7527796	0.7571513	TMEM56	0.994226125
7850	34.554948	34.7639	AKIRIN2	0.993989403
7851	78.714542	79.20752	2-Sep	0.99377612
7852	8.2183836	8.2712807	ALDH16A1	0.993604732
7853	4.9699979	5.0041282	BET1	0.99317957
7854	30.838675	31.054664	TMEM205	0.993044865
7855	58.07739	58.494029	SMAP2	0.992877232
7856	4.0449559	4.0749666	STK36	0.992635362
7857	2.378311	2.3959563	OASL	0.992635362
7858	1.071922	1.0798749	ERCC6L2	0.992635362
7859	3.0478903	3.0705035	SMN1	0.992635362
7860	71.91893	72.477797	PPP6R1	0.992289123
7861	10.44941	10.532868	FBXO21	0.992076446
7862	28.864764	29.103668	CDC45	0.991791284
7863	3.8156512	3.847232	ANKMY2	0.991791284
7864	26.850188	27.07635	H2AFY2	0.991647243
7865	20.446331	20.620058	PRKRA	0.991574853
7866	5.6850994	5.7338242	FAM131A	0.991502216
7867	14.70433	14.83474	TSEN54	0.991209162

7868	1.7192211	1.7349886	ZNF211	0.990912037
7869	2.2387616	2.2592939	ZNF75A	0.990912036
7870	8.1883947	8.263493	ITSN1	0.990912036
7871	11.199039	11.301748	GALNT1	0.990912036
7872	90.26276	91.097937	SHISA5	0.990832105
7873	51.00905	51.492159	KIAA1967	0.99061781
7874	17.50958	17.677628	SCARF2	0.990493754
7875	20.378341	20.57944	PPID	0.990228177
7876	8.6102901	8.6956658	RAB40C	0.990181814
7877	15.682237	15.840717	TRIAP1	0.989995375
7878	29.676865	29.980562	INPPL1	0.989870197
7879	6.8446808	6.9154949	ARHGEF9	0.989760081
7880	8.0103558	8.0934057	DOCK6	0.989738566
7881	13.14183	13.278082	TSC22D2	0.989738566
7882	7.0626032	7.1362414	ZNF526	0.98968109
7883	24.691884	24.95468	TMEM11	0.989469061
7884	3.8063836	3.8485683	NDUFAF5	0.989038857
7885	5.5890125	5.6537775	ATG5	0.988544831
7886	5.2644473	5.3254512	E2F6	0.988544831
7887	4.1148139	4.1631998	LOC493754	0.988377734
7888	97.34854	98.49934	COPZ1	0.988316667
7889	20.311613	20.552203	C12orf52	0.988293725
7890	12.993492	13.150778	FUT11	0.988039828
7891	19.181546	19.420478	RNF34	0.987696878
7892	25.956993	26.280323	LIG1	0.987696877
7893	7.7865164	7.8857282	ZNF3	0.98741881
7894	11.270585	11.414189	AP5S1	0.987418809
7895	12.203338	12.359305	EARS2	0.987380632
7896	7.996725	8.1020895	SLC35E2B	0.986995388
7897	1.3391645	1.3568093	ZMYM5	0.986995388
7898	0.3174057	0.3215878	DNAH14	0.986995387
7899	19.845607	20.110892	ATP2C1	0.986808881
7900	7.1756042	7.273684	KIF3C	0.986515799
7901	4.1422793	4.1994891	FECH	0.98637697
7902	6.3174522	6.4053832	SYNRG	0.986272315
7903	4.0953309	4.1523328	FDX1	0.986272315
7904	0.6991555	0.7091529	SCAI	0.98590237
7905	3.3612823	3.409346	SLC35E4	0.98590237
7906	44.347991	44.982132	PSMD14	0.98590237
7907	31.918399	32.375993	INCENP	0.985866264
7908	27.965974	28.387021	RHOT2	0.985167619
7909	14.069165	14.286936	HAUS1	0.984757303
7910	5.190523	5.2708652	CIDECP	0.984757303

7911	23.587946	23.953055	NRM	0.984757303
7912	0.7412396	0.7527129	LAMC2	0.984757303
7913	12.718289	12.917278	ERC1	0.98459507
7914	28.813496	29.268253	EXOSC1	0.984462466
7915	5.5943473	5.6828864	FKBP14	0.984420058
7916	6.5147719	6.6191513	SIK2	0.984230695
7917	18.925542	19.233278	SUGP2	0.983999798
7918	7.0727889	7.1888013	PIGK	0.983862069
7919	33.257679	33.805736	SAMD4B	0.983788054
7920	115.58458	117.49362	HNRNPUL1	0.983752038
7921	5.2301033	5.3175429	MTHFSD	0.98355638
7922	2.0691396	2.1037326	HEATR5A	0.983556379
7923	59.502428	60.546173	TAF15	0.982761174
7924	12.071496	12.283725	CAND1	0.98272268
7925	84.211376	85.713122	PPP2CA	0.982479395
7926	63.309702	64.448303	CLTC	0.98233312
7927	11.567603	11.776094	DBF4B	0.98229541
7928	7.5213834	7.6569465	DDX51	0.98229541
7929	0.6097306	0.6207202	CXorf23	0.982295409
7930	15.636371	15.927982	DSCR3	0.981691911
7931	4.6379711	4.7247121	C2CD2L	0.981640983
7932	144.96684	147.68205	EWSR1	0.981614461
7933	136.32351	138.89049	GADD45GIP1	0.98151797
7934	15.591533	15.885963	SNX8	0.981466003
7935	16.569388	16.88309	UCKL1	0.981419143
7936	41.203359	41.984451	FTSJ3	0.981395682
7937	10.626355	10.829056	WDR62	0.981281689
7938	188.61357	192.24839	EIF3G	0.981093091
7939	22.153518	22.58208	SEC16A	0.981022017
7940	1.468161	1.4966424	ATP8B3	0.980969776
7941	10.78431	10.993519	MRPS24	0.980969775
7942	341.79417	348.462	RPL39	0.980864982
7943	10.646945	10.855646	ASF1A	0.980774829
7944	11.501134	11.726969	PTBP3	0.980742198
7945	4.3269587	4.4121285	CTNS	0.980696448
7946	4.7917945	4.8870297	ZNF747	0.98051266
7947	106.9509	109.0886	SNRPG	0.980403956
7948	10.267653	10.475687	WDR85	0.980141253
7949	29.94222	30.553266	JAK1	0.980000643
7950	15.954555	16.28132	OIP5-AS1	0.979930062
7951	19.433134	19.831939	NDNL2	0.97989077
7952	2.2401857	2.286897	CENPC1	0.97957437
7953	8.6273942	8.8072886	ZNF703	0.97957437

7954	15.450625	15.772794	CREBBP	0.97957437
7955	12.595159	12.863201	SPAG9	0.979162091
7956	5.1167385	5.2259997	RAP2A	0.97909277
7957	16.7594	17.129941	NCDN	0.978368846
7958	5.7667353	5.8945377	C16orf52	0.978318505
7959	0.888968	0.908869	LRRC27	0.978103538
7960	8.0359704	8.2158689	RNF121	0.978103538
7961	43.749115	44.740763	STX10	0.977835687
7962	44.86245	45.890609	AZIN1	0.977595432
7963	44.190491	45.205149	DCTD	0.977554384
7964	32.273284	33.023167	SEC22B	0.977292208
7965	59.846504	61.257618	MYOF	0.97696426
7966	244.92709	250.75725	HNRNPC	0.976749761
7967	3.3406803	3.420897	ZC3H10	0.976550992
7968	4.3389976	4.4431859	CYP2R1	0.976550992
7969	33.616614	34.443879	SAMM50	0.975982238
7970	5.835186	5.9792528	DNAJC24	0.975905552
7971	78.790455	80.738098	FLNC	0.975877023
7972	101.93736	104.4709	FAM129B	0.975748931
7973	6.4718926	6.6337782	THSD4	0.97559677
7974	5.9257138	6.0761256	SIRT3	0.975245443
7975	14.289703	14.654652	YARS2	0.975096709
7976	20.036296	20.548724	BCL2L12	0.975062801
7977	0.6400953	0.6565688	PRDM11	0.97490973
7978	7.7972602	7.9979304	ANAPC15	0.97490973
7979	9.3144698	9.5541869	WASL	0.97490973
7980	71.146511	73.001582	ARF3	0.974588614
7981	38.926558	39.942284	PET100	0.974570159
7982	30.86782	31.681794	GLYR1	0.974307841
7983	14.546005	14.933478	ABI1	0.97405342
7984	39.412341	40.466213	MAGOH	0.973956739
7985	23.013005	23.6345	EIF1AD	0.973703905
7986	15.524225	15.943476	ZMYND11	0.973703905
7987	21.471464	22.054899	NPRL3	0.97354622
7988	44.036118	45.243178	SLC25A11	0.973320635
7989	1.1253432	1.1563663	ZNF564	0.973171924
7990	3.5776738	3.6763019	ITPKA	0.973171923
7991	2.3732327	2.4386572	RP9P	0.973171923
7992	9.4014812	9.6606581	RAD9A	0.973171923
7993	4.5489659	4.6743703	LOC100288911	0.973171923
7994	0.8508273	0.8742826	ZNF430	0.973171923
7995	1.9229481	1.9759593	SLMO1	0.973171923
7996	1611.1965	1655.9531	VIM	0.972972311

7997	27.493947	28.265857	NR2F6	0.972691107
7998	37.294971	38.345427	FAF2	0.972605467
7999	3.5943995	3.6957757	ZNF398	0.972569713
8000	11.316717	11.637352	RUNX1	0.972447837
8001	7.4386224	7.6508248	CROCCP2	0.972264114
8002	150.98331	155.42101	NDUFB8	0.971447257
8003	27.467533	28.27506	DRG2	0.9714403
8004	8.3252378	8.570978	SMIM10	0.971328795
8005	10.713739	11.029982	CIR1	0.971328794
8006	24.341062	25.072552	ALDH9A1	0.970825045
8007	2.1470049	2.2118353	SSBP2	0.970689342
8008	55.279445	56.948647	C22orf28	0.970689342
8009	10.630146	10.952592	DCAF5	0.970559882
8010	6.5871325	6.7906013	MTMR11	0.970036704
8011	18.881572	19.468119	MRP63	0.969871437
8012	0.6024268	0.6214619	GOLGA8O	0.969370471
8013	1.7026103	1.7564083	ISL2	0.969370471
8014	2.0592836	2.1243515	PMS2P3	0.969370471
8015	0.9591876	0.9894954	ST6GALNAC3	0.96937047
8016	6.9159126	7.1344371	ABCD3	0.96937047
8017	13.879592	14.31815	EIF2B3	0.96937047
8018	17.130821	17.677041	NAB2	0.969100074
8019	53.159677	54.855781	ALAS1	0.969080674
8020	9.334	9.6347162	LINC00839	0.968788266
8021	8.2786662	8.5453824	DCK	0.968788266
8022	62.985502	65.02308	GTF2F1	0.968663761
8023	7.5525617	7.799456	ENKD1	0.968344682
8024	4.7461584	4.9030791	C17orf59	0.967995477
8025	151.66945	156.68772	EEF1D	0.96797282
8026	12.258794	12.666406	COQ10B	0.967819478
8027	6.1867277	6.3931403	RABL5	0.967713427
8028	19.820178	20.483533	CASC4	0.967615232
8029	29.03253	30.010509	IRF3	0.967412146
8030	2.1889118	2.2629421	C5orf28	0.967285803
8031	56.636528	58.561565	CDC42EP1	0.967127973
8032	6.1631439	6.3726785	ZDHHC3	0.967119859
8033	3.3581969	3.4738997	MCTS1	0.966693708
8034	6.6329967	6.862473	POLB	0.966560701
8035	6.378063	6.5987196	FAM104B	0.966560701
8036	13.145794	13.601675	RNF123	0.966483456
8037	16.615794	17.201164	ERGIC2	0.965969171
8038	33.619775	34.807126	BOD1	0.965887702
8039	10.239121	10.602116	PEX5	0.965761985

8040	17.71538	18.35111	UQCRB	0.965357373
8041	11.103454	11.50202	AFAP1L1	0.965348186
8042	20.816561	21.56479	CD47	0.965303182
8043	1.2898289	1.3365242	LACE1	0.965062158
8044	16.794587	17.402596	KAT2A	0.965062157
8045	0.725048	0.7512966	SEMA6C	0.965062157
8046	1.4768884	1.5303557	ZNF2	0.965062157
8047	35.436352	36.719243	BCL7B	0.965062157
8048	14.337066	14.856107	10-Sep	0.965062157
8049	0.4952628	0.5131926	TLR6	0.965062157
8050	22.384323	23.194696	ZFP91	0.965062157
8051	11.419151	11.832555	RPS17	0.965062157
8052	9.9350949	10.297506	SH3RF1	0.964805901
8053	2.7813438	2.884564	CELSR1	0.964216353
8054	6.4612741	6.7016584	HPS3	0.96413063
8055	4.4340319	4.598995	CEP41	0.96413063
8056	7.5602342	7.841504	FGFR1OP	0.96413063
8057	8.7251757	9.0520094	5-Mar	0.963893801
8058	10.22695	10.611229	TMED5	0.96378562
8059	15.756474	16.350736	MVD	0.963655361
8060	11.831013	12.281822	C12orf43	0.963294644
8061	22.255262	23.110555	MED8	0.962991208
8062	46.872596	48.676095	ANKRD13A	0.962948989
8063	37.703372	39.170076	TXNL4A	0.962555502
8064	81.942524	85.136591	IER2	0.962483031
8065	7.7691951	8.0731006	TENM3	0.96235578
8066	20.583931	21.392794	SLC25A10	0.962189949
8067	11.988573	12.461292	DYRK4	0.96206507
8068	4.4892624	4.6665533	RAPGEF2	0.962008163
8069	20.320025	21.128156	URM1	0.961750965
8070	10.338453	10.75113	PTPRM	0.961615507
8071	29.888933	31.084702	EDC4	0.961531895
8072	83.233688	86.595444	SSSCA1	0.961178607
8073	11.511591	11.977701	HEATR3	0.961085253
8074	10.10947	10.522017	ATRN	0.96079197
8075	21.821937	22.718359	AGAP3	0.96054196
8076	0.8991681	0.9364985	MAP3K8	0.960138371
8077	1.0460207	1.0894478	SDHAP1	0.960138371
8078	3.9150195	4.0775576	HOXD-AS2	0.960138371
8079	9.4039535	9.7943731	ARL5A	0.960138371
8080	17.562155	18.291275	RXRB	0.960138371
8081	1.0076346	1.0494681	ATAD2B	0.960138371
8082	21.944289	22.876542	CRCP	0.95924853

8083	14.937982	15.579946	TUG1	0.95879552
8084	42.428914	44.25837	SEC24C	0.958664183
8085	19.248536	20.081495	SPC25	0.958521068
8086	22.996942	23.992528	CUL4A	0.958504371
8087	26.755917	27.91766	MOV10	0.958386826
8088	8.2706851	8.6302472	C1GALT1C1	0.958336985
8089	7.4992244	7.8252479	OSBPL1A	0.958336985
8090	2.2506789	2.3490927	STXBP5	0.958105615
8091	31.266466	32.642401	SOD2	0.957848237
8092	15.539855	16.228973	GPATCH8	0.957537779
8093	34.334556	35.859458	RAB35	0.957475602
8094	4.9761103	5.197509	TMEM255B	0.957402934
8095	2.6800347	2.7992757	WDSUB1	0.957402934
8096	60.235071	62.941853	SRSF9	0.956995528
8097	85.18758	89.015651	CCDC85B	0.956995528
8098	40.995159	42.843314	INTS1	0.956862464
8099	2.2978426	2.4015127	NLRP1	0.95683135
8100	15.547792	16.250978	GIT2	0.956729596
8101	21.4536	22.430551	FTO	0.956445531
8102	3.5109651	3.6708469	CLOCK	0.956445531
8103	8.7059843	9.1038584	GDNF	0.95629611
8104	5.4475249	5.6972492	DYRK1A	0.956167575
8105	17.33714	18.135884	CHIC2	0.955957797
8106	116.99621	122.39748	NDUFB10	0.955871089
8107	6.658131	6.9660743	NUAK2	0.955793853
8108	10.456401	10.941524	ZNF655	0.955662202
8109	3.3836491	3.5406329	SLC1A1	0.955662201
8110	3.6196952	3.7876304	ZNF544	0.955662201
8111	12.800306	13.396954	JKAMP	0.95546389
8112	19.551829	20.466227	LENG1	0.955321623
8113	2.8435436	2.9768637	RTTN	0.955214584
8114	21.172409	22.168571	BLVRA	0.95506424
8115	36.049092	37.745201	NUBP2	0.955064239
8116	13.325545	13.953313	ZNF444	0.955009426
8117	11.949928	12.520131	RAD51	0.954457079
8118	1.1658713	1.2215021	SLC30A4	0.954457079
8119	1.8307423	1.9180981	FLJ37453	0.954457079
8120	4.2968969	4.5019279	SLC29A4	0.954457079
8121	2.518423	2.6385922	RPS18P9	0.954457079
8122	10.581639	11.086553	DUS3L	0.954457079
8123	1.7574097	1.8412663	CLEC11A	0.954457078
8124	1.0015194	1.049308	LOC100128361	0.954457078
8125	1.5134924	1.5857103	ZNF654	0.954457078

8126	1.1583113	1.2135813	GPR75	0.954457078
8127	0.4253051	0.445599	KLHL4	0.954457077
8128	12.093569	12.676914	ZNF76	0.953983638
8129	5.1220487	5.3714262	SLC9B2	0.953573322
8130	5.3109708	5.5704179	PTEN	0.953424116
8131	6.4304061	6.744539	DYRK3	0.953424116
8132	8.0429182	8.4398161	SEC24A	0.952973159
8133	5.8025927	6.0894196	KPTN	0.952897508
8134	5.6601002	5.9432112	CDC40	0.952363971
8135	276.41604	290.27479	TUBA1C	0.95225642
8136	8.5795279	9.0112141	TOB1	0.952094561
8137	36.130745	37.949674	PLEKHB2	0.952069981
8138	45.417618	47.707363	GSS	0.952004362
8139	6.2692796	6.5858477	ZNF691	0.95193206
8140	58.111399	61.045742	PPP1R11	0.95193206
8141	78.000577	81.979042	C11orf68	0.951469732
8142	5.1113776	5.3722015	NXPE3	0.951449336
8143	1.3087204	1.3757532	PLXNB1	0.951275555
8144	2.3989932	2.52187	ZNF669	0.951275555
8145	66.217261	69.620695	CTNNB1	0.951114622
8146	35.410809	37.232962	ANKRD11	0.951060761
8147	28.749088	30.232177	JAGN1	0.950943348
8148	10.34465	10.878727	TXNDC17	0.950906271
8149	2.5375515	2.6685612	UBE2W	0.950906271
8150	21.231048	22.334167	MRPL13	0.950608461
8151	36.788273	38.70657	NT5C	0.950440003
8152	21.859191	23.000385	ZNF384	0.950383699
8153	522.38928	549.72327	LDHA	0.95027681
8154	2.6778469	2.8183183	ANKRD16	0.950157723
8155	23.668313	24.912123	PTPN23	0.950072131
8156	16.522851	17.392454	CNTROB	0.950001163
8157	4.8345604	5.0893831	FBXW8	0.949930521
8158	9.6115071	10.118544	TBC1D8	0.949890298
8159	4.5795718	4.8214506	SECISBP2L	0.949832771
8160	22.390029	23.575845	TEX264	0.949702084
8161	633.25428	666.97094	S100A11	0.949448078
8162	14.154669	14.920351	DESI2	0.948682035
8163	10.865519	11.453478	CACTIN	0.94866547
8164	2.1285	2.2456585	H3F3A	0.947828905
8165	2.2657717	2.390486	DHFRL1	0.947828905
8166	2.040195	2.1524929	VPS13C	0.947828904
8167	3.9410794	4.1580072	FBXO4	0.947828904
8168	1.1461973	1.2092871	RMDN2	0.947828904

8169	2.1118328	2.2280739	LINC00857	0.947828904
8170	1.1853126	1.2505554	CGREF1	0.947828904
8171	2.8932198	3.0524706	SPAG16	0.947828904
8172	3.4945038	3.6868509	SYTL4	0.947828904
8173	4.1591675	4.3880995	SNN	0.947828904
8174	0.477433	0.5037122	KIAA1456	0.947828904
8175	6.268064	6.6154714	LRRK1	0.947485613
8176	16.490962	17.40751	SRC	0.947347528
8177	7.3063832	7.71444	UBE2Q2	0.947104818
8178	6.7831585	7.1656971	ABTB1	0.946615295
8179	23.09082	24.395922	DR1	0.946503269
8180	81.929215	86.608457	NCOR2	0.945972458
8181	9.0909948	9.6117088	CEP95	0.945825038
8182	2.1113012	2.232463	EVI5	0.945727288
8183	36.918455	39.041553	ESD	0.945619513
8184	8.5615025	9.0541702	JAG1	0.94558666
8185	60.77232	64.323781	NDUFS7	0.944787742
8186	5.4998442	5.8221739	POLL	0.944637561
8187	4.094945	4.3349378	USP30	0.944637561
8188	5.7585668	6.0968517	KCTD1	0.944514817
8189	22.95759	24.30824	GET4	0.944436532
8190	9.4242573	9.9824506	DCTN5	0.944082545
8191	1.3225716	1.4009068	ZNF559	0.944082545
8192	14.288557	15.137591	GTF2H1	0.943912256
8193	2.4662663	2.6131769	ZNF652	0.943780825
8194	41.840053	44.336654	ARPC5L	0.943689914
8195	5.2959051	5.6121731	H2AFJ	0.943646076
8196	38.839556	41.204413	MCRS1	0.942606707
8197	209.1685	221.9588	PGK1	0.942375344
8198	3.4132766	3.6219927	UST	0.942375343
8199	16.984597	18.025195	ZCCHC3	0.942269791
8200	52.69046	55.924809	KLHDC3	0.942166113
8201	5.4044146	5.736656	HIPK3	0.942084487
8202	7.3658444	7.8186665	GOPC	0.942084487
8203	20.95923	22.247718	HDGFRP3	0.942084487
8204	9.4413033	10.021716	LEF1	0.942084487
8205	102.06565	108.40151	PIGT	0.941551892
8206	5.5853996	5.9330869	TADA1	0.941398586
8207	3.9467497	4.1933134	DZIP1	0.94120073
8208	12.264273	13.032146	PACS2	0.941078552
8209	3.3037748	3.511507	SNX13	0.940842451
8210	8.0036939	8.5109393	ZKSCAN1	0.940400778
8211	1.163593	1.2378706	HOXA2	0.939995608

8212	3.1647608	3.3667826	TMEM168	0.939995608
8213	1.4645081	1.5579946	GRTP1	0.939995608
8214	1.3358942	1.4211707	C17orf100	0.939995608
8215	2.524743	2.6859094	KDM6A	0.939995608
8216	0.9076633	0.9656038	FAM185A	0.939995608
8217	2.3681761	2.5193481	SFT2D2	0.939995608
8218	1.0435628	1.1101784	MAP3K7CL	0.939995608
8219	14.529517	15.457005	GNA13	0.939995608
8220	1.3847143	1.4731072	CCDC24	0.939995607
8221	0.6136617	0.6528346	FOXA1	0.939995607
8222	1.0555436	1.122924	BEND3P3	0.939995607
8223	45.022156	47.915149	BUD31	0.939622594
8224	26.666547	28.399669	NUMA1	0.938973873
8225	39.057217	41.605169	B4GALT2	0.938758772
8226	15.009985	15.989182	NRBF2	0.938758772
8227	6.7006363	7.1377616	C19orf55	0.938758771
8228	21.13266	22.516022	EP400	0.938561021
8229	3.1845559	3.3930614	ABCB10	0.938549461
8230	2.159352	2.3014556	SLC22A15	0.938254875
8231	8.588373	9.1535608	NME6	0.938254875
8232	2.4839639	2.6474298	LYPD6	0.938254875
8233	0.8472227	0.9029772	SLC16A7	0.938254875
8234	8.2945075	8.8422167	PPM1M	0.938057472
8235	20.98325	22.372207	SRI	0.937915971
8236	4.687186	4.9980147	RBM48	0.937809571
8237	240.76358	256.74142	HSPA5	0.937766809
8238	15.525718	16.5597	SLC41A3	0.937560386
8239	11.550092	12.325908	CCDC9	0.937058121
8240	9.9270685	10.600763	TMEM87A	0.936448454
8241	218.63575	233.53151	PPDPF	0.936215191
8242	14.071466	15.033011	IL4R	0.936037731
8243	5.6396772	6.0256573	KIAA0430	0.935943902
8244	12.067677	12.895145	RAB3GAP1	0.93583107
8245	7.6097191	8.134218	CBX8	0.935519438
8246	3.9695143	4.2431126	ZNF446	0.935519438
8247	1.6806411	1.7964791	FTX	0.935519438
8248	10.136105	10.83776	POM121	0.935258229
8249	18.646271	19.942629	COX14	0.934995631
8250	18.281791	19.555014	CUL1	0.9348902
8251	30.243698	32.352991	CYB5B	0.934803772
8252	3.6036705	3.8559559	ZNF133	0.934572556
8253	15.108652	16.181792	TELO2	0.933682204
8254	92.962522	99.574234	CENPB	0.933600173

8255	31.22534	33.447959	CCDC137	0.933549924
8256	59.686624	63.935118	CDK2AP1	0.933549924
8257	2.0069707	2.1508229	TRIQK	0.933117591
8258	19.54898	20.957409	C20orf111	0.932795641
8259	8.1125899	8.69868	C4orf46	0.932623093
8260	1.5620023	1.6754454	PLAG1	0.932290726
8261	36.68198	39.356153	WDR34	0.932051983
8262	80.351216	86.225575	MRPL11	0.931872189
8263	22.669411	24.328056	RHNO1	0.931821733
8264	10.064051	10.801445	VPS4B	0.931731911
8265	3.011406	3.2332559	BRIP1	0.931384961
8266	233.26843	250.49068	SLC25A3	0.931245963
8267	544.03559	584.28219	S100A6	0.931117871
8268	1.5944422	1.7133566	ERI2	0.930595652
8269	0.5199877	0.5587686	TMEM86A	0.930595652
8270	5.7584072	6.1878725	WDR92	0.930595652
8271	0.8585926	0.9226269	PRDM16	0.930595652
8272	49.289508	52.965548	C9orf16	0.930595652
8273	27.332021	29.370459	PTPRA	0.930595652
8274	6.7629645	7.2673503	PTRH1	0.930595652
8275	5.0329039	5.4082607	ABHD14A	0.930595652
8276	1.6130686	1.7333722	LINC00265	0.930595652
8277	3.9353661	4.228868	FBXL15	0.930595651
8278	0.8358269	0.8981633	CCT6P3	0.930595651
8279	18.588595	19.981691	POLR1A	0.930281367
8280	11.300882	12.151251	ZNF512B	0.930018001
8281	35.348079	38.019858	RAB10	0.929726748
8282	53.730423	57.821104	TXN2	0.929252801
8283	13.640067	14.694458	BCL2L2	0.928245663
8284	100.62492	108.45681	U2AF2	0.927787873
8285	9.0222637	9.7269366	TRIM35	0.927554489
8286	6.4236329	6.9253429	C8orf82	0.927554489
8287	109.70098	118.29982	PRELID1	0.927313127
8288	24.074277	25.965356	EHMT2	0.927169157
8289	89.81157	96.874141	DAZAP1	0.927095393
8290	1.966033	2.1207865	CARD8	0.927030151
8291	6.7364944	7.2667479	ABHD6	0.927030151
8292	4.5724785	4.9323946	HHIP	0.927030151
8293	13.096143	14.134048	MTIF3	0.926567099
8294	2.0160132	2.1764447	TSTD2	0.926287339
8295	26.464683	28.574259	SCRIB	0.926172143
8296	15.467472	16.709454	CDK11B	0.925671866
8297	13.045438	14.094624	CAMK2G	0.925561218

8298	1.0157086	1.097881	PANK1	0.925153572
8299	2.808767	3.0360009	LOC730101	0.925153572
8300	6.4064946	6.9247904	HSCB	0.925153572
8301	2.4182877	2.6139311	FAM200B	0.925153571
8302	70.572487	76.299893	CORO1C	0.924935598
8303	25.37589	27.438866	ARHGAP23	0.924815554
8304	15.748073	17.030262	PPP6C	0.924711126
8305	23.984597	25.945205	CMTM3	0.924432766
8306	3.8387669	4.1530309	ANKRD13B	0.924329014
8307	10.04877	10.878797	FAM120AOS	0.92370235
8308	73.175453	79.251854	KXD1	0.923327954
8309	1.8172262	1.968378	ADAMTSL5	0.923209972
8310	28.372381	30.747885	VAC14	0.922742524
8311	37.598253	40.765045	CTDSP1	0.922315988
8312	12.51179	13.56572	HMGXB4	0.922309279
8313	7.0898799	7.6879076	PIGP	0.922211907
8314	1.557686	1.689076	SEMA4G	0.922211907
8315	133.96298	145.29938	SUMO3	0.921979025
8316	14.267848	15.479625	YTHDF3	0.921717915
8317	25.899856	28.100581	ELAVL1	0.921684005
8318	3.6718434	3.9841861	EZH1	0.921604389
8319	148.27557	160.88852	ERGIC3	0.921604389
8320	68.223721	74.035118	EFTUD2	0.921504865
8321	51.432641	55.818214	NCOA4	0.921431147
8322	5.5462776	6.0192517	PIK3C2A	0.921423114
8323	6.6949688	7.2662262	PHKB	0.921381833
8324	6.9561141	7.5511796	MFSD3	0.921195696
8325	57.688851	62.637853	BCAR1	0.920990232
8326	14.70145	15.964186	TMEM208	0.920901947
8327	13.108856	14.235561	SOX12	0.92085284
8328	49.265514	53.502433	SMG5	0.920808866
8329	9.7382944	10.577277	KIAA0196	0.92068063
8330	6.4239492	6.9773915	SLC9A8	0.920680629
8331	5.0142266	5.4472391	MAGOHB	0.920507894
8332	2.1575895	2.343912	GAREM	0.920507894
8333	99.551991	108.16884	LSM3	0.920338863
8334	5.2691919	5.7257911	RELT	0.9202557
8335	7.5084372	8.1595157	RBM33	0.920206227
8336	5.6380366	6.1311152	ZNF318	0.91957767
8337	0.9195786	1.0005133	CCDC103	0.919106817
8338	14.549112	15.829621	DFNA5	0.919106817
8339	4.4975198	4.8933592	TMEM64	0.919106816
8340	2.6508743	2.8841852	VCIP1	0.919106816

8341	4.7634171	5.1826589	ACYP1	0.919106816
8342	24.313275	26.453155	PPP1R35	0.919106816
8343	1.0079782	1.0966932	NOXO1	0.919106816
8344	1.3169005	1.4328046	INO80B	0.919106816
8345	2.3681761	2.576606	RBP7	0.919106816
8346	0.5007346	0.5448057	RTKN2	0.919106816
8347	0.6424647	0.6990099	ALG11	0.919106815
8348	43.344478	47.165	EPB41L2	0.918996665
8349	55.489453	60.451786	DDX17	0.917912551
8350	101.22708	110.30659	PSMD4	0.917688442
8351	9.8719956	10.761085	NDUFC2	0.917379172
8352	3.3247062	3.6242927	PRR14L	0.917339304
8353	22.795441	24.855767	SLC25A37	0.917108758
8354	19.499151	21.264717	CDC27	0.916972067
8355	10.848884	11.834226	LINC00116	0.916737984
8356	2.7881923	3.0422317	CEP57L1	0.916495718
8357	11.023276	12.029482	C12orf49	0.916355
8358	0.9133868	0.9973384	PFKFB2	0.915824292
8359	28.028223	30.611905	ERLIN1	0.915598775
8360	3.8924389	4.25195	ENPP1	0.915447952
8361	68.824955	75.198864	PDAP1	0.915239288
8362	6.9181985	7.5596735	NBN	0.915145149
8363	29.21767	31.933368	PAIP1	0.91495735
8364	3.7578899	4.1083842	PARP16	0.914688033
8365	48.12304	52.617045	UBE2E3	0.914590321
8366	141.70144	154.96076	NDUFV1	0.914434324
8367	6.5477633	7.1622637	ERI1	0.914203045
8368	37.461444	40.985359	COPB2	0.914020157
8369	3.5297777	3.8619947	RIMKLB	0.913977872
8370	14.397796	15.754094	NEDD4L	0.913908248
8371	7.5277386	8.2381725	HOTAIRM1	0.913763172
8372	28.735911	31.451141	KLF10	0.913668315
8373	25.751236	28.188723	PEG10	0.913529712
8374	17.513913	19.175794	UBN1	0.913334468
8375	18.352503	20.09611	ELP6	0.913236609
8376	7.7031162	8.4358675	ZSWIM6	0.91313859
8377	7.9425352	8.6988092	TAF1C	0.913060061
8378	6.4206231	7.0324787	GMEB1	0.912995734
8379	3.0134423	3.3006093	MECOM	0.912995734
8380	3.8873185	4.2596319	PSD3	0.912594926
8381	2.4166931	2.64887	GORAB	0.912348678
8382	6.8514904	7.509728	NBAS	0.912348678
8383	15.290539	16.763784	ZRANB2	0.912117411

8384	14.486446	15.885545	TMEM161A	0.911926294
8385	15.921381	17.460148	SPR	0.911869755
8386	19.935652	21.864165	ARMC1	0.911795739
8387	10.495889	11.512764	MICAL3	0.911674114
8388	20.755336	22.768601	ODF2	0.911577136
8389	16.426784	18.020749	CNOT8	0.911548372
8390	30.433433	33.392101	RIN1	0.911396189
8391	70.572169	77.433311	POLR2C	0.911392884
8392	27.948359	30.679771	MAP2K3	0.910970248
8393	2.9554548	3.2445418	TATDN3	0.910900506
8394	15.68166	17.221564	HECTD3	0.910582841
8395	18.873669	20.733543	CDC23	0.91029636
8396	13.253973	14.568143	WDFY1	0.909791545
8397	31.028792	34.111866	RAF1	0.90961873
8398	59.624774	65.553048	ACOT7	0.909565242
8399	7.1788222	7.893303	NDOR1	0.909482661
8400	1.3562596	1.4913499	C5orf42	0.909417438
8401	5.9149158	6.5040713	C6orf211	0.909417437
8402	23.042072	25.340399	PHF19	0.909301874
8403	55.040704	60.53458	TMED3	0.909244011
8404	1.6604241	1.826242	KIN	0.909202648
8405	20.931959	23.024506	CMAS	0.909116525
8406	0.6294746	0.6927486	TET1	0.908662422
8407	21.96778	24.180485	RGMB	0.908492132
8408	27.457615	30.226058	GALE	0.908408711
8409	9.648125	10.622892	YEATS4	0.908238999
8410	11.903661	13.109699	TRIM5	0.90800416
8411	58.246258	64.150621	AK2	0.907960931
8412	1.08322	1.1931074	HECW2	0.907898196
8413	45.73945	50.420576	CDK2AP2	0.907158428
8414	4.8442469	5.3408774	PPAPDC1B	0.907013306
8415	13.909569	15.340293	WRAP73	0.906734224
8416	41.324842	45.593881	CEBPB	0.90636815
8417	3.1202793	3.4427194	SLC9A6	0.906341444
8418	10.90267	12.030627	CBLL1	0.906242869
8419	6.6976354	7.3909182	LRRC57	0.906198013
8420	4.5481704	5.0189587	FAM111B	0.906198013
8421	7.291075	8.0463403	HDAC9	0.906135551
8422	6.580222	7.262311	NR4A1	0.90607824
8423	7.8867423	8.7055302	AGO1	0.905946231
8424	62.319741	68.841446	UROD	0.905264846
8425	116.47988	128.68595	HM13	0.905148419
8426	0.1109942	0.12268	TNXB	0.904745775

8427	0.4284278	0.4735339	ZNF519	0.904745774
8428	0.615007	0.6797567	MTHFD2L	0.904745773
8429	11.322003	12.514016	IFT52	0.904745773
8430	0.8761541	0.9683981	RND1	0.904745773
8431	14.477761	16.002021	DOCK1	0.904745773
8432	0.9038392	0.998998	TM7SF2	0.904745773
8433	25.931205	28.661316	CD24	0.904745772
8434	5.0427533	5.5736689	ZNF74	0.904745772
8435	1.7958102	1.9848783	TRIM13	0.904745772
8436	24.15226	26.695079	AIDA	0.904745772
8437	10.170892	11.241713	SWI5	0.904745772
8438	6.4222557	7.0984092	RPS6KA3	0.904745772
8439	2.2076218	2.4400466	LOC100652772	0.904745772
8440	8.7879307	9.7131493	RN7SK	0.904745772
8441	123.57574	136.58614	SEC61G	0.904745772
8442	3.818839	4.2208973	RAB3A	0.904745772
8443	0.4300697	0.4753487	ZNF695	0.904745772
8444	0.9203763	1.0172762	DHRS4L2	0.904745772
8445	0.5525744	0.6107511	PPIEL	0.904745772
8446	0.7649693	0.8455075	OSR2	0.904745772
8447	0.2911769	0.3218329	SMG1P1	0.90474577
8448	65.112006	71.989887	AP3D1	0.904460454
8449	21.2138	23.459261	REXO4	0.904282513
8450	11.607605	12.837111	MTRF1L	0.904222496
8451	7.3769735	8.1613636	NAPG	0.903889816
8452	158.511	175.37058	ATP5G2	0.903863093
8453	19.344928	21.40999	GTPBP1	0.903546798
8454	15.527806	17.190846	CHCHD6	0.903260147
8455	19.776131	21.895905	RBMX2	0.903188551
8456	18.380735	20.350938	EXOSC9	0.90318855
8457	2.1748475	2.4086681	ACADSB	0.902925358
8458	7.2792943	8.0635851	ARID1B	0.902736714
8459	57.78914	64.025789	MAT2A	0.902591616
8460	20.123017	22.297905	PRPS2	0.902462214
8461	6.2506335	6.9273903	LTBP1	0.902307105
8462	11.419151	12.659871	ALG2	0.901995785
8463	10.594854	11.752637	MGAT5	0.901487384
8464	1453.3123	1612.3828	RPS11	0.901344473
8465	185.02924	205.28651	RPN1	0.901321947
8466	5.6972525	6.3219325	ATP2B1	0.90118845
8467	5.6340218	6.2517687	PLD2	0.90118845
8468	5.3548313	5.9428599	HINFP	0.901052933
8469	30.940696	34.356546	LRFN4	0.900576437

8470	4.572145	5.0769095	ACSS1	0.900576437
8471	6.6679878	7.4112628	STAMBPL1	0.899710082
8472	85.217625	94.719676	AURKAIP1	0.899682394
8473	11.613311	12.911797	SLC25A17	0.899434153
8474	7.2164061	8.0260211	BRAP	0.899126233
8475	1.4135967	1.5721894	SLC4A8	0.899126233
8476	1.1207287	1.2464642	HKDC1	0.899126233
8477	47.274207	52.59471	RAVER1	0.898839583
8478	14.351095	15.968619	FJX1	0.898706081
8479	4.0871966	4.5503391	CAPN7	0.898218025
8480	45.328772	50.477913	BLCAP	0.8979922
8481	4.3803717	4.8782281	ZFAT	0.897943173
8482	8.6800833	9.6689001	CASP3	0.897732239
8483	17.484846	19.482501	IDS	0.897464116
8484	11.247216	12.532698	SLC25A43	0.897429769
8485	17.404461	19.393674	MAD1L1	0.897429769
8486	5.3762879	5.9926776	MTERFD2	0.897142867
8487	21.949123	24.475826	CRKL	0.896767415
8488	39.12304	43.646356	TCEAL4	0.896364412
8489	20.671004	23.066993	BCAT2	0.896129146
8490	5.8324941	6.5085419	RCAN3	0.896129146
8491	20.23464	22.580049	WDR61	0.896129146
8492	18.267796	20.385226	ATG16L1	0.896129146
8493	0.4558629	0.5087023	MMAA	0.896129145
8494	13.024969	14.538174	POLG	0.895915068
8495	12.961016	14.46696	FAM122B	0.895904609
8496	9.1347385	10.204087	C16orf70	0.895203871
8497	48.88482	54.616454	CBS	0.895056638
8498	12.378978	13.831715	SBDSP1	0.894970608
8499	18.893186	21.111597	EML3	0.894919795
8500	8.6342542	9.6517398	ACSF3	0.89458009
8501	6.9661921	7.7877581	BMPR2	0.894505456
8502	0.984613	1.1010291	FMN1	0.894266092
8503	1.5516017	1.7350559	PDXDC2P	0.894266091
8504	13.25802	14.825587	ISCA1	0.894266091
8505	10.37622	11.606835	ABHD11	0.893974989
8506	90.831938	101.61032	TLN1	0.893924327
8507	4.5401002	5.0795912	GAS2L3	0.893792434
8508	2.0778836	2.3247943	STRIP2	0.893792434
8509	39.277787	43.951244	UBE2N	0.893667253
8510	109.56464	122.62411	PSMB5	0.893499996
8511	36.483445	40.83307	HSPBP1	0.893477877
8512	3.2533863	3.6413661	STAM2	0.893452136

8513	13.911575	15.575056	TBL2	0.893195827
8514	7.0111201	7.849477	BCAP29	0.893195826
8515	5.1951767	5.8176943	PRSS12	0.892995827
8516	1.5287994	1.7119894	ADORA2A	0.892995827
8517	2.9179002	3.2675406	TEFM	0.892995827
8518	174.98913	196.01376	RPL31	0.892738998
8519	11.392615	12.764432	TRRAP	0.892528168
8520	36.037029	40.377574	CAMK2N1	0.892501093
8521	28.792036	32.265335	C19orf24	0.892351995
8522	11.136393	12.485265	SKA1	0.891962865
8523	53.007538	59.428525	KIF22	0.891954473
8524	19.870372	22.284871	PNPO	0.891652976
8525	9.9112001	11.119	COPG2	0.891375145
8526	54.707643	61.38731	ZWINT	0.891188143
8527	22.308781	25.035252	TJP1	0.891094713
8528	20.000426	22.447272	CHCHD1	0.890995836
8529	32.189095	36.137401	SORBS3	0.890741841
8530	20.704918	23.245475	TSEN34	0.890707448
8531	29.531802	33.155837	BCKDK	0.890696925
8532	38.62724	43.377319	TFPI2	0.890493938
8533	3.034469	3.4080425	PABPC1L	0.890384728
8534	134.99038	151.65904	MYBL2	0.890091218
8535	5.0176445	5.6383484	ZKSCAN5	0.889913874
8536	6.7254963	7.559143	ZNF584	0.889716773
8537	3.3730453	3.791145	LOC729852	0.889716773
8538	43.088852	48.438681	OGDH	0.88955461
8539	4.8528016	5.4556096	XIAP	0.889506761
8540	7.8567201	8.8331526	C1orf159	0.889458209
8541	5.4112171	6.0837227	ITPKB	0.889458209
8542	12.167126	13.67998	STX2	0.889411098
8543	12.621378	14.19351	COA6	0.889235845
8544	10.662047	11.995017	URGCP	0.888873039
8545	4.4168633	4.969898	KDM3A	0.88872312
8546	2.3034911	2.5919109	DENND5B	0.88872312
8547	8.7351222	9.830869	KDM6B	0.888540191
8548	105.86784	119.1649	NDUFV2	0.888414621
8549	18.053069	20.321747	LLGL1	0.888362046
8550	3.1147987	3.5066125	ENTPD7	0.888264306
8551	21.161389	23.833422	SEC23A	0.887887156
8552	28.873865	32.527515	CBX6	0.887675098
8553	17.42651	19.63354	CCDC101	0.887588773
8554	4.0862647	4.604177	TTC39C	0.887512519
8555	12.379044	13.949088	PHF13	0.887444672

8556	13.327875	15.020215	HSD17B11	0.887329187
8557	62.182426	70.08333	AP1S1	0.887264147
8558	3.6566139	4.121361	TRERF1	0.887234564
8559	285.95117	322.50847	PRDX1	0.88664702
8560	44.191092	49.853327	FAM65A	0.886422119
8561	0.4177738	0.4713782	LOC100288198	0.886281574
8562	0.5336735	0.6021489	TRABD2B	0.886281574
8563	0.3773075	0.4257197	LINC00547	0.886281574
8564	0.6700949	0.7560745	TMEM133	0.886281574
8565	1.4229269	1.6055021	MRPS31P5	0.886281573
8566	0.3008655	0.3394694	FGD4	0.886281573
8567	1.4522613	1.6386004	SETD9	0.886281573
8568	3.5982648	4.0599566	TCTEX1D2	0.886281573
8569	8.3984299	9.476029	RBBP9	0.886281573
8570	1.2343504	1.3927294	TOPORS-AS1	0.886281573
8571	14.669678	16.551938	MCU	0.886281573
8572	74.583102	84.152829	PPIF	0.886281573
8573	1.2924	1.4582273	CDK5R1	0.886281573
8574	11.206709	12.644637	BAIAP2L1	0.886281573
8575	0.3196856	0.3607043	ZNF738	0.886281573
8576	0.7944072	0.8963373	KDM4A-AS1	0.886281572
8577	0.3978988	0.448953	P2RX5-TAX1BP3	0.886281572
8578	0.4561828	0.5147154	YY2	0.886281572
8579	96.145663	108.56321	NDUFA3	0.88561918
8580	3.3506757	3.785647	FNIP1	0.885099864
8581	15.576674	17.60174	DUSP12	0.88495082
8582	41.066378	46.41534	CIAPIN1	0.884758752
8583	41.306599	46.698197	THOP1	0.884543766
8584	28.448186	32.162344	INF2	0.884518413
8585	1.8945409	2.1423268	MTHFR	0.884337973
8586	3.1961452	3.6149731	PARD6G	0.884140796
8587	5.5749583	6.3055096	WWC2	0.884140796
8588	18.920696	21.400985	PIP4K2A	0.884103977
8589	48.992884	55.428141	TRIP10	0.883899096
8590	59.049607	66.824944	TEX261	0.883646183
8591	141.76195	160.50869	ATP5G1	0.883204206
8592	10.788358	12.215021	ELP4	0.883204206
8593	32.740658	37.074196	POLRMT	0.883111753
8594	5.7751638	6.5400415	FBXO38	0.883046969
8595	26.806575	30.357622	PIM3	0.883026177
8596	219.24448	248.32055	DAD1	0.882909116
8597	6.6988831	7.5879969	RHOQ	0.882826284
8598	5.1865247	5.8758913	C6orf120	0.882678802

8599	6.58698	7.462488	LINC00294	0.882678802
8600	6.4406027	7.296655	ZNF7	0.882678802
8601	8.5537108	9.6930142	MZT1	0.882461394
8602	52.231773	59.188735	INO80E	0.882461394
8603	5.6455314	6.3983444	KIAA1467	0.882342544
8604	103.68263	117.65293	AUP1	0.881258369
8605	33.78234	38.334749	SCAND1	0.881245882
8606	15.792399	17.920536	HIF1AN	0.881245882
8607	14.413798	16.360032	LUC7L	0.881037303
8608	11.087644	12.587999	DLG4	0.880810699
8609	6.4963025	7.3753673	VPS36	0.880810699
8610	10.25281	11.640197	RBM34	0.880810699
8611	68.943353	78.277324	AAMP	0.880757657
8612	10.958908	12.44639	SMURF1	0.880488883
8613	12.596968	14.308671	RNF166	0.880373029
8614	11.0931	12.60325	TAZ	0.880177705
8615	43.802861	49.769713	WDR18	0.88011077
8616	69.920954	79.488964	GSTO1	0.879630961
8617	61.171993	69.546183	CRIM1	0.879588063
8618	1.3027364	1.4822419	LINC00641	0.878895894
8619	2.3905476	2.719944	RPL23AP7	0.878895893
8620	10.112212	11.505586	ANTXR2	0.878895892
8621	82.708303	94.127378	UBE2D3	0.878684874
8622	37.553471	42.778674	NABP2	0.877854959
8623	3.1695741	3.6127534	TUBG2	0.877329234
8624	5.0173568	5.7188985	PIGL	0.877329234
8625	52.355363	59.681275	MRPS16	0.877249404
8626	6.6302587	7.5597317	TMEM184C	0.877049473
8627	3.6495647	4.1630898	PIGB	0.876648078
8628	95.109985	108.49278	CSTB	0.876648078
8629	6.954464	7.9349546	COQ7	0.876434
8630	20.461414	23.350659	HADH	0.876267092
8631	45.929316	52.417206	SNRNP40	0.876225953
8632	50.967677	58.221169	ATP2A2	0.875414862
8633	24.598688	28.105819	TRIM25	0.875216884
8634	0.4135657	0.4726902	NUTM2D	0.87491899
8635	1.1983244	1.3696404	DPY19L1P1	0.874918989
8636	1.4601239	1.6688675	ZNF180	0.874918989
8637	9.7289952	11.119881	RAP1GDS1	0.874918989
8638	1.0782665	1.2324186	SCN5A	0.874918989
8639	3.4026212	3.88907	OXR1	0.874918989
8640	1.7895351	2.0453724	ARHGAP5-AS1	0.874918988
8641	14.541969	16.630264	UBE4B	0.874428013

8642	59.172501	67.669951	HSPE1	0.874428013
8643	6.1704628	7.0565704	SGK223	0.874428013
8644	6.1566178	7.0438618	HPS4	0.874040115
8645	13.213893	15.118177	HYAL2	0.874040115
8646	10.693457	12.243839	INPP5K	0.873374559
8647	21.719385	24.871314	GTSE1	0.873270531
8648	6.1861697	7.0848758	LMTK2	0.873151476
8649	2.5422732	2.9116061	MC1R	0.873151475
8650	9.2305144	10.572129	TOR1AIP2	0.873098895
8651	5.5132143	6.3150326	TAOK1	0.873030221
8652	35.766505	40.970634	NELFB	0.872979035
8653	36.058476	41.311049	APOBEC3C	0.872853064
8654	16.941002	19.410373	EFCAB14	0.872780868
8655	80.112932	91.814345	GPS2	0.872553544
8656	1.8787267	2.1534328	ZNF324B	0.872433423
8657	4.8939852	5.610741	EVC	0.872252907
8658	10.091417	11.575383	CKLF	0.871799848
8659	6.2797023	7.2041209	DIAPH3	0.871681973
8660	33.94266	38.943489	TIMM13	0.87158755
8661	5.1188886	5.8735842	GMCL1	0.871510213
8662	174.25527	200.00426	SEC61A1	0.871257781
8663	93.912514	107.83452	HIGD2A	0.87089474
8664	9.0800387	10.428043	ZNF263	0.870732774
8665	19.348503	22.220943	TMEM97	0.870732774
8666	1.8217403	2.0921922	TTC21B	0.870732774
8667	6.1136634	7.0212855	PGGT1B	0.870732773
8668	3.742303	4.2978778	RAD52	0.870732773
8669	6.6231578	7.6064184	HERC4	0.870732773
8670	9.1805947	10.543527	DALRD3	0.870732773
8671	21.516173	24.715728	CAPZA2	0.87054576
8672	3.4079205	3.9151019	MDM4	0.870455116
8673	8.6349105	9.9238535	SNX18	0.870116689
8674	25.851397	29.715727	FGD5-AS1	0.869956719
8675	33.181399	38.148374	TCEAL8	0.869798511
8676	73.132278	84.089059	SRRT	0.869700284
8677	3.3279161	3.8274084	TMEM68	0.869495937
8678	24.441024	28.113197	CCDC94	0.869379022
8679	18.004664	20.71014	7-Mar	0.869364653
8680	7.5119427	8.6409099	BCL6	0.869346256
8681	50.089406	57.664461	PSMD12	0.868635633
8682	2.882997	3.3192992	ADPRM	0.868555941
8683	2.2746307	2.618865	ANKRD49	0.868555941
8684	1.0104801	1.1634025	LTB4R	0.868555941

8685	28.495507	32.809392	PRR11	0.868516775
8686	138.74614	159.80155	MRPL37	0.868240217
8687	8.0476637	9.2720131	TGFBRAP1	0.867952149
8688	4.5812799	5.279083	METTL14	0.867817373
8689	3.4446198	3.9720173	EXOG	0.867221754
8690	4.6403543	5.3508278	PFKFB4	0.867221754
8691	21.517783	24.814011	REXO1	0.867162615
8692	32.317953	37.270848	MARVELD1	0.867110757
8693	14.836101	17.117303	UPF3A	0.866731244
8694	9.7649499	11.267231	DCAF11	0.86666815
8695	4.6266019	5.3414235	LRRC8C	0.866173963
8696	11.551556	13.337725	INTS12	0.866081423
8697	7.9029918	9.1254714	RAB22A	0.866036562
8698	44.515852	51.446646	SMARCE1	0.865281902
8699	7.2074057	8.330531	EME1	0.865179631
8700	23.720268	27.419246	GMPPA	0.865095559
8701	34.103544	39.424913	DNAJC7	0.865025226
8702	264.5639	305.89531	CSNK2B	0.864883809
8703	3.4286041	3.9646393	SMAD9	0.864795959
8704	1.8235452	2.1086421	COG6	0.864795959
8705	6.7055991	7.756731	CCDC90B	0.864487764
8706	21.487519	24.861363	SLC25A38	0.864293672
8707	52.486494	60.731755	THRAP3	0.864234768
8708	9.8492953	11.402554	ZAK	0.863779748
8709	4.6661509	5.4025755	STK3	0.863690082
8710	28.551711	33.072436	BAZ2A	0.863308381
8711	17.910358	20.751932	SOLH	0.863069437
8712	25.630028	29.720787	RNF115	0.862360343
8713	31.984576	37.095437	C21orf33	0.862223984
8714	20.278164	23.518877	PUM1	0.862207997
8715	28.84346	33.467093	TAP2	0.86184539
8716	0.2691804	0.3123966	WDR78	0.861662641
8717	0.8170393	0.9482126	LOC441155	0.861662641
8718	0.3406891	0.3953857	DOCK2	0.861662641
8719	0.210888	0.2447454	ADAMTS13	0.861662641
8720	0.34687	0.4025589	DBIL5P	0.861662641
8721	0.1714094	0.1989286	KLB	0.861662641
8722	0.5045993	0.5856112	AMH	0.861662641
8723	9.2367866	10.719725	FAM109B	0.861662641
8724	4.1928364	4.8659838	ORC4	0.861662641
8725	3.7296166	4.3283954	ZNF343	0.86166264
8726	1.0077345	1.1695233	RAB11B-AS1	0.86166264
8727	7.8345677	9.0923841	SNORA10	0.86166264

8728	1.0922406	1.2675965	LOC100270746	0.86166264
8729	1.4871515	1.7259093	BSCL2	0.86166264
8730	29.893371	34.692662	PITPNA	0.86166264
8731	9.9913967	11.595486	GPT2	0.86166264
8732	7.9072323	9.1767148	CNIH3	0.86166264
8733	3.0646985	3.5567267	FBXO30	0.86166264
8734	18.924928	21.963268	FAM134A	0.86166264
8735	3.2224354	3.7397878	PTPDC1	0.86166264
8736	1.5536742	1.803112	LOC100505549	0.86166264
8737	10.989427	12.753747	MCAT	0.86166264
8738	0.5310895	0.6163543	ASB9	0.86166264
8739	1.0430405	1.2104976	HIST2H2BF	0.86166264
8740	0.2057251	0.2387536	ARGFX	0.861662638
8741	25.733028	29.872562	NUDCD3	0.861426891
8742	40.514129	47.04639	KIF2C	0.861152781
8743	16.222858	18.841192	EP300	0.861031386
8744	17.00896	19.755071	PCGF2	0.860992086
8745	18.494552	21.483781	HOXC10	0.860861094
8746	12.339695	14.335576	RRP7A	0.860774329
8747	0.3086828	0.3587857	ABCB5	0.860354432
8748	11.850168	13.775259	SLU7	0.860250079
8749	5.1443154	5.9808053	TTC23	0.860137574
8750	22.280556	25.916668	SFSWAP	0.859699855
8751	5.3856523	6.2665795	CDK14	0.859424556
8752	31.101446	36.209642	SNAPIN	0.858927204
8753	2.8117099	3.2738555	MKLN1	0.858837517
8754	4.7884675	5.5768094	BTRC	0.858639263
8755	16.894339	19.679756	ITPRIPL2	0.858462832
8756	11.64243	13.562768	SLC2A3	0.858411084
8757	4.9272316	5.7399441	IL15RA	0.858411083
8758	32.465321	37.822604	ZNF787	0.858357633
8759	6.3683197	7.4198317	SPATA2	0.858283571
8760	1.6314643	1.9011508	STARD9	0.85814565
8761	14.645587	17.066552	GTF2H4	0.85814565
8762	39.486975	46.020554	CNOT3	0.858029123
8763	6.4249648	7.488339	COQ3	0.857995991
8764	7.5255375	8.7710637	C11orf74	0.857995991
8765	137.80955	160.62259	ALYREF	0.857971188
8766	6.7047878	7.8167521	USP20	0.857745992
8767	16.985876	19.806482	NFATC2IP	0.857591793
8768	5.1795197	6.0405422	GEMIN2	0.857459408
8769	29.447755	34.357175	LSM14A	0.857106415
8770	27.813969	32.458087	CLINT1	0.856919543

8771	14.36384	16.765715	FOXC2	0.856738854
8772	13.401642	15.645267	DYM	0.856594037
8773	29.211554	34.108097	ISOC2	0.856440443
8774	29.52486	34.487488	SMAGP	0.856103526
8775	97.578007	114.04309	PUF60	0.855624034
8776	52.460178	61.328529	RPL26L1	0.855396003
8777	7.0376847	8.2285159	C6orf136	0.855279954
8778	10.560785	12.35356	NRN1	0.854877895
8779	14.70561	17.202	DNAJC19	0.854877895
8780	8.7916996	10.285978	GIGYF1	0.854726656
8781	53.356592	62.436773	DIAPH1	0.854569978
8782	47.969074	56.13474	GNG12	0.854534531
8783	12.983473	15.196351	UIMC1	0.854380985
8784	1.7037825	1.9946646	PLEKHH2	0.854169922
8785	11.503749	13.467753	RABEPK	0.854169921
8786	10.328216	12.093696	CPNE3	0.854016487
8787	8.6775367	10.161851	TANC1	0.853932686
8788	4.3707699	5.1184929	SAMD8	0.853917358
8789	25.697795	30.102596	RFTN1	0.853673715
8790	88.656911	103.87466	KHDRBS1	0.853498932
8791	8.6809939	10.171574	POLR3E	0.85345633
8792	2.3000614	2.6962923	C1orf115	0.853046014
8793	2.3970664	2.8100083	FOXO1	0.853046014
8794	4.6757973	5.482061	SOCS7	0.85292689
8795	21.936789	25.721523	CCDC43	0.852857329
8796	11.773743	13.811968	RBL2	0.85243054
8797	42.769359	50.17832	RNF10	0.852347369
8798	16.365114	19.206809	CTNNAL1	0.85204752
8799	245.40958	288.03142	PSMD2	0.852023634
8800	9.6602829	11.3382	MED9	0.852012019
8801	11.083064	13.008997	DPH1	0.851953766
8802	47.066728	55.254632	USP39	0.851815067
8803	44.373382	52.098532	MRPS18A	0.851720379
8804	2.4374211	2.8624172	NUDT6	0.851525433
8805	2.8450444	3.341115	IRF7	0.851525433
8806	24.783186	29.111389	B3GNT1	0.851322689
8807	10.588294	12.438477	PDPK1	0.851253293
8808	173.66625	204.02774	NUTF2	0.851189392
8809	115.80137	136.09674	SNRNP70	0.850875413
8810	60.112914	70.652873	DNAJB11	0.850820528
8811	10.502378	12.344767	CDK5RAP2	0.850755518
8812	25.461924	29.932491	11-Sep	0.850645
8813	2.5853502	3.0396416	ZBTB6	0.850544413

8814	9.4273987	11.085533	TMEM39A	0.850423563
8815	5.6932397	6.6965598	KDM5A	0.850173805
8816	10.672305	12.561736	METTL2A	0.849588387
8817	31.190551	36.713376	H2AFV	0.84956913
8818	4.3452296	5.1147259	DIP2A	0.849552787
8819	99.67967	117.3395	NHP2L1	0.849497991
8820	3.059482	3.6021317	BMF	0.849353174
8821	2.1767432	2.5628246	LOC400027	0.849353174
8822	5.0441342	5.9387948	PTPRN	0.849353174
8823	60.605977	71.364391	TMEM147	0.849246752
8824	6.6830765	7.871213	CLCN3	0.849052943
8825	4.4599115	5.2543602	UPRT	0.848802004
8826	72.712598	85.704926	MCM5	0.848406292
8827	290.72835	342.845	RHOC	0.847987729
8828	3.4501835	4.0692075	MGC45800	0.847876038
8829	9.317753	10.989523	FAM168A	0.847876038
8830	139.33762	164.35232	KHSRP	0.847798294
8831	20.51792	24.203538	NFKBIB	0.84772398
8832	5.8208577	6.8667314	TPMT	0.847689733
8833	60.426708	71.293713	PHC2	0.847574142
8834	2.7464352	3.2404899	KIRREL3	0.847537023
8835	1.8065144	2.1314873	SLC24A1	0.847537023
8836	3.1385467	3.7031382	FBXO44	0.847537023
8837	8.7104928	10.279645	WHSC1L1	0.847353441
8838	47.479595	56.048301	ICMT	0.847119268
8839	6.1481845	7.2588114	DDX28	0.846996042
8840	10.816148	12.77159	LYRM1	0.846891281
8841	10.16844	12.007987	MICU2	0.846806389
8842	4.7004008	5.5511986	WDR37	0.846736201
8843	43.741874	51.693828	NDUFS2	0.846172076
8844	5.245373	6.2002334	CYP27B1	0.845996047
8845	11.542126	13.643239	ASCC1	0.845996047
8846	2.1231554	2.5096517	ZNF304	0.845996047
8847	5.0545403	5.9746619	USP38	0.845996047
8848	4.0436842	4.7797908	LUC7L2	0.845996047
8849	2.787746	3.2952234	TYW1	0.845996047
8850	1.8209665	2.1524527	SCYL3	0.845996047
8851	1.1527938	1.3626468	CABP1	0.845996047
8852	0.7478451	0.8839818	WDR31	0.845996046
8853	0.2448878	0.2894668	DNM3	0.845996045
8854	120.62147	142.7473	CCDC124	0.845000025
8855	7.2987843	8.6377649	GCDH	0.844985299
8856	8.4954871	10.060625	OARD1	0.844429388

8857	17.205687	20.37552	C11orf73	0.844429388
8858	2.8918181	3.4249961	MAP3K13	0.844327416
8859	5.8539185	6.9352841	DLX5	0.844077689
8860	3.68197	4.3621221	STARD4	0.844077689
8861	3.4234994	4.0570714	PIGM	0.843835137
8862	6.4323819	7.6241121	HAUS5	0.843689309
8863	10.316807	12.230626	MED13L	0.843522375
8864	13.743507	16.29767	EML4	0.843280504
8865	2.577379	3.0564529	TRIM2	0.843258195
8866	3.7774191	4.4803728	ARMCX5	0.843103753
8867	92.142771	109.30605	TMED2	0.842979582
8868	8.2813402	9.8255608	SGSM2	0.842836398
8869	1.6229342	1.926298	RFXAP	0.842514582
8870	8.0718116	9.5806195	NOL12	0.842514582
8871	1.4522613	1.7237225	TRMT13	0.842514582
8872	4.0041825	4.7526567	ENDOG	0.842514582
8873	5.4429628	6.4603782	FAM86DP	0.842514582
8874	217.08281	257.68069	COX7B	0.842448894
8875	26.812571	31.831517	DDRGK1	0.842327772
8876	9.9940675	11.867751	ZRANB1	0.842119776
8877	21.154366	25.123366	SETD5	0.842019567
8878	4.9931784	5.9303696	UFSP2	0.841967494
8879	5.0462071	5.9933514	WBP4	0.841967494
8880	9.6054756	11.408369	KBTBD2	0.841967494
8881	4.3123936	5.1238958	SPATA13	0.841623974
8882	9.9018547	11.766237	EFR3A	0.841548118
8883	11.458946	13.618625	OCRL	0.841417272
8884	20.948738	24.900102	CREB3L2	0.841311345
8885	3.6454723	4.3335725	SLC30A7	0.841216408
8886	75.971724	90.325791	RN7SL2	0.841085622
8887	16.998942	20.211018	DCBLD2	0.841072993
8888	4.458916	5.3020304	FAM63A	0.840982737
8889	19.826591	23.577831	GOLIM4	0.840899685
8890	23.493413	27.941694	TCTN3	0.840801334
8891	20.428442	24.305135	MED27	0.840498997
8892	27.331082	32.522346	PPP3R1	0.840378543
8893	15.155057	18.033939	SMG6	0.840363115
8894	3.1056861	3.6967125	ZHX1	0.840121075
8895	1.6529552	1.9675202	TMEM38A	0.840121075
8896	5.2402195	6.2374576	ACN9	0.840121074
8897	3.5321949	4.2043879	TRIM3	0.840121074
8898	1.2612633	1.5012875	ZNF674-AS1	0.840121074
8899	33.387948	39.741829	ASCC2	0.840121074

8900	3.5670751	4.2459059	DHRS4-AS1	0.840121074
8901	0.7618654	0.906852	ZNF169	0.840121074
8902	8.2497882	9.8243411	PKN3	0.83972941
8903	226.46079	269.69021	PSMA7	0.839707108
8904	26.007349	30.973566	C9orf78	0.839662743
8905	42.97289	51.183836	DDX23	0.83957931
8906	11.317059	13.481015	INSIG1	0.839481226
8907	41.639926	49.61376	CD164	0.839281793
8908	14.820644	17.659896	USP4	0.839225996
8909	4.3807703	5.2202708	ZNF35	0.839184485
8910	13.035481	15.534963	PRMT7	0.839106027
8911	23.053493	27.474829	NT5E	0.839076869
8912	5.8761826	7.0046137	C6orf203	0.838901741
8913	4.3228068	5.1529358	HYLS1	0.83890174
8914	21.23502	25.312881	SURF2	0.83890174
8915	5.7992422	6.9135437	OSBPL8	0.83882339
8916	8.9253355	10.641434	UNC119B	0.838734279
8917	66.190261	78.940811	RIC8A	0.838479619
8918	18.096463	21.583478	NCAPG2	0.838440527
8919	13.431418	16.031748	ARNT	0.837801213
8920	27.969777	33.386898	MAPK1	0.837747106
8921	25.401929	30.33245	PTRH2	0.837450633
8922	11.629315	13.887106	ZC3H7A	0.83741814
8923	152.0151	181.54776	APP	0.837328406
8924	0.9374944	1.1200066	SGK494	0.837043708
8925	0.8788865	1.0499888	ZNF484	0.837043708
8926	5.0975417	6.089935	EFCAB11	0.837043708
8927	570.06956	681.14468	ALDOA	0.836928751
8928	10.841592	12.960808	TMEM167B	0.836490473
8929	4.9454793	5.9133843	HLCS	0.836319622
8930	69.4665	83.082979	TTC1	0.836109886
8931	220.06084	263.39466	NDUFS6	0.835479521
8932	4.7897029	5.7329573	SMAD4	0.835468096
8933	10.077186	12.062515	CHN1	0.835413315
8934	19.60036	23.464391	SRP19	0.835323608
8935	1.0104801	1.2099386	CCDC18	0.835149944
8936	7.3183771	8.7629499	ACOT2	0.835149944
8937	41.00279	49.113645	BOLA3	0.834855358
8938	6.0088978	7.1981374	USP16	0.83478509
8939	5.146615	6.1660418	TOMM40L	0.834670799
8940	65.075444	77.969205	MRPL14	0.834630087
8941	7.4428393	8.9180463	RAD17	0.834581815
8942	9.6442425	11.557573	C16orf91	0.834452242

8943	4.3328258	5.1924191	ELMOD2	0.834452241
8944	4.8436117	5.8054108	KIAA0922	0.834327136
8945	2.8101335	3.3700018	DNAJC22	0.833867072
8946	5.2467145	6.2920275	SLC30A6	0.833867071
8947	8.8907637	10.662087	SMIM3	0.833867071
8948	143.43285	172.17245	YBX3	0.833076676
8949	2.7346541	3.2831324	ZNF8	0.832940552
8950	1.6585032	1.9911423	PIGA	0.832940552
8951	11.984322	14.392882	MESDC2	0.832656175
8952	829.03707	995.86352	RPL23	0.832480608
8953	16.935084	20.348798	MORC2	0.832240014
8954	16.553719	19.89749	LUC7L3	0.831950135
8955	30.693984	36.896778	GJA1	0.83188791
8956	3.6090304	4.3416854	CSPP1	0.831251018
8957	22.821337	27.460466	ATXN2	0.831061537
8958	71.320588	85.831171	PSMD7	0.830940407
8959	33.671988	40.530462	RNF40	0.830782245
8960	14.828081	17.849895	EXOC2	0.830709711
8961	1.8502583	2.2276533	TTC33	0.830586283
8962	4.5617402	5.4953522	8-Mar	0.830108797
8963	16.968996	20.445111	AP5B1	0.829978185
8964	7.2270856	8.7099639	USP40	0.829749209
8965	47.82125	57.647488	PPP1R18	0.829546124
8966	2.4841874	2.9949141	MEX3A	0.829468652
8967	20.685163	24.938314	WAC	0.82945315
8968	6.6669862	8.0416287	PANX1	0.829059189
8969	12.96708	15.641491	AGK	0.829018153
8970	14.854163	17.921686	MAPK8	0.828837397
8971	8.1299148	9.8088176	ORC3	0.828837397
8972	46.456217	56.074886	PITRM1	0.82846744
8973	3.8291668	4.6234807	HDAC4	0.828200014
8974	16.279064	19.663205	ATAD1	0.82789478
8975	15.383585	18.582426	NME2	0.827856659
8976	0.2118958	0.2561615	NLRP8	0.827196137
8977	0.1494439	0.1806632	CXXC4	0.827196137
8978	0.1883837	0.2277377	ARHGAP30	0.827196136
8979	0.2993171	0.3618453	LOC100507173	0.827196135
8980	10.391322	12.562102	SP100	0.827196135
8981	0.7920171	0.957472	METTL25	0.827196135
8982	0.3677097	0.4445255	TRAF3IP3	0.827196135
8983	4.3416563	5.2486419	SAYSD1	0.827196135
8984	1.530661	1.8504209	THEMIS2	0.827196135
8985	4.184929	5.0591738	ZCCHC8	0.827196135

8986	25.632738	30.987497	SNAPC2	0.827196135
8987	0.7318683	0.8847579	REP15	0.827196135
8988	7.69111	9.297807	EID2	0.827196135
8989	9.1103607	11.013544	GEMIN7	0.827196135
8990	2.8094299	3.3963287	AMDHD2	0.827196135
8991	0.3334392	0.4030957	WDR11-AS1	0.827196135
8992	0.7697119	0.9305071	LOC100289388	0.827196135
8993	2.5022238	3.0249462	RRP7B	0.827196135
8994	0.3898962	0.4713467	CDKL3	0.827196135
8995	0.3775353	0.4564036	KLRAP1	0.827196135
8996	3.0039568	3.6314927	GPNMB	0.827196135
8997	1.9031918	2.3007745	HIST1H2BC	0.827196135
8998	9.6545943	11.671469	SMARCAL1	0.827196135
8999	1.2831165	1.5511635	ZNF792	0.827196134
9000	0.2851858	0.344762	GMCL1P1	0.827196134
9001	0.4779805	0.5778321	GAD1	0.827196134
9002	0.6102474	0.73773	FBXO48	0.827196134
9003	0.2535274	0.30649	ERV3-1	0.827196134
9004	0.3602411	0.4354966	UCA1	0.827196134
9005	13.972496	16.905072	TRIM41	0.826526882
9006	31.603596	38.239952	PITPNB	0.82645492
9007	9.2079516	11.141876	ATMIN	0.826427365
9008	9.1042623	11.020373	SMAD5	0.826130161
9009	12.006901	14.535481	SCCPDH	0.826040833
9010	16.110375	19.505922	KCTD20	0.825922219
9011	4.0153349	4.8628972	DAGLA	0.825708372
9012	18.01706	21.82806	SLC27A4	0.825408247
9013	9.7490436	11.812134	PARP12	0.825341435
9014	101.75757	123.34308	HNRNPH1	0.824996145
9015	9.9427242	12.051843	HMMR	0.824996145
9016	91.465382	110.87481	SRSF2	0.824942876
9017	4.6195331	5.6002992	RBM18	0.82487255
9018	4.0494411	4.9095716	EGLN1	0.824805395
9019	22.39669	27.157475	SMPD4	0.824697053
9020	11.965831	14.512446	PNISR	0.82452201
9021	14.921809	18.100171	NSDHL	0.824401553
9022	125.2925	152.03951	SSU72	0.824078562
9023	18.309301	22.218761	GAMT	0.824046911
9024	7.0618962	8.5727199	PCGF3	0.823763786
9025	1.5198742	1.8459267	DAAM1	0.823366523
9026	1.6886725	2.0519103	TTC14	0.822975746
9027	7.2418704	8.8020426	FYCO1	0.822748844
9028	4.9160226	5.9751195	KLHL2	0.822748844

9029	8.3455177	10.146574	DLEU1	0.822496157
9030	6.7788753	8.2442439	HBS1L	0.822255543
9031	40.959729	49.818642	RAD23B	0.822176741
9032	27.434871	33.370201	RPF1	0.822136831
9033	2.7632098	3.3620043	FOXQ1	0.821893595
9034	8.4525671	10.288806	LCMT2	0.821530408
9035	15.649665	19.055037	CCDC71	0.821287591
9036	62.560874	76.175564	SSBP1	0.821272204
9037	1266.7371	1542.6069	TPT1	0.821166533
9038	9.0462806	11.017085	C12orf45	0.82111381
9039	3.6969688	4.5023829	ZSCAN32	0.82111381
9040	2.5947828	3.1600769	GRAMD4	0.82111381
9041	3.9449487	4.8057459	COL4A3BP	0.82088166
9042	5.9484396	7.247488	ANKS6	0.820758811
9043	17.614013	21.464531	ZNFX1	0.820610179
9044	4.949617	6.0334709	RCBTB1	0.820359803
9045	1.2693825	1.5479045	PIPSL	0.820065134
9046	6.3374022	7.7279254	RAD51AP1	0.820065134
9047	1.5537078	1.8946151	ELOVL4	0.820065134
9048	2.4669009	3.0081768	SMA4	0.820065134
9049	10.687154	13.033731	PURA	0.819961184
9050	13.456861	16.414601	QPCT	0.819810455
9051	4.8137767	5.8731071	ZNF212	0.819630316
9052	95.010673	115.93126	GPI	0.819543141
9053	5.587258	6.8175794	RC3H2	0.819536911
9054	2.6709731	3.2596997	SYNE3	0.819392397
9055	0.9976293	1.2187323	C7orf60	0.818579509
9056	4.9598211	6.0590585	PPM1D	0.818579508
9057	15.339818	18.739558	UTP6	0.818579508
9058	7.2046545	8.8038887	MPHOSPH8	0.818349117
9059	46.54053	56.879976	RFC2	0.81822345
9060	119.04663	145.51369	PPM1G	0.818112904
9061	2.2072321	2.6979779	TAB3	0.818106067
9062	12.681845	15.503681	SPATS2	0.817989328
9063	6.4553907	7.8922886	PRPF18	0.817936477
9064	0.1282853	0.1568466	TCF21	0.817903227
9065	864.62069	1057.1555	RPL30	0.817874642
9066	100.68169	123.11746	SLC1A5	0.817769398
9067	13.233069	16.183277	FAM203A	0.817700261
9068	11.279354	13.794369	RASSF3	0.817678188
9069	3.235426	3.9573322	ODF2L	0.817577575
9070	4.7519666	5.8138802	MAP3K12	0.817348562
9071	74.059327	90.671184	YKT6	0.816790113

9072	16.660853	20.409909	GPSM3	0.816311975
9073	15.097574	18.494858	MICAL2	0.816311975
9074	12.649249	15.503681	MON1B	0.815886813
9075	78.538618	96.261658	LSM7	0.815886813
9076	38.497795	47.192696	CHTF8	0.815757469
9077	11.501255	14.099733	E4F1	0.815707299
9078	4.0791234	5.0017117	GSPT2	0.815545485
9079	260.98268	320.08341	XRCC6	0.81535834
9080	6.9899639	8.5801922	CSNK1G3	0.81466286
9081	25.884014	31.77267	MRPL54	0.81466286
9082	858.02289	1053.84	RPS13	0.814187085
9083	51.81061	63.659777	NDUFA9	0.813867291
9084	15.607193	19.177874	DLG1	0.813812481
9085	29.237483	35.929943	EXOSC4	0.813735843
9086	1.6666405	2.048387	ZNF557	0.813635543
9087	3.7948316	4.6640435	RTN4IP1	0.813635542
9088	148.51715	182.57864	TGFBI	0.813442087
9089	57.92574	71.217865	ADSL	0.813359671
9090	71.840355	88.328223	SMTN	0.813334093
9091	1.4602568	1.7955714	TRPS1	0.813254627
9092	36.776382	45.227513	PTPLAD1	0.813141831
9093	76.383715	93.941793	SNHG6	0.813096201
9094	6.5157423	8.0155347	NCOA3	0.812889283
9095	31.857248	39.203574	PHF23	0.812610812
9096	3.0578851	3.7638993	ADCK1	0.812424775
9097	2.7865411	3.4299066	KBTBD4	0.812424775
9098	7.1851658	8.8440999	NEDD4	0.812424775
9099	3.6015625	4.4331027	HS3ST3A1	0.812424775
9100	3.2701776	4.0252066	C6orf52	0.812424775
9101	1.3801291	1.6987778	SH3BP5	0.812424775
9102	18.006291	22.166794	ITGA6	0.812309193
9103	10.407966	12.81451	PHC1	0.812201643
9104	7.8306223	9.6421302	LTBP4	0.81212576
9105	3.17112	3.9056612	DOPEY2	0.811929092
9106	3.2592735	4.0151964	FNBP1L	0.811734525
9107	5.5476337	6.8342956	ZNF24	0.811734525
9108	14.530242	17.902531	PINX1	0.811630616
9109	9.4758951	11.676063	VIPAS39	0.811565975
9110	80.769826	99.547335	IDH3B	0.811371047
9111	1.4684132	1.8099765	LRRC37A4P	0.811288517
9112	15.690513	19.350223	RASSF1	0.810869895
9113	3.1792097	3.9213377	COX18	0.810746212
9114	5.7403455	7.0803236	ATF1	0.810746212

9115	21.464163	26.477647	THAP11	0.810652212
9116	39.828417	49.137698	MRPL34	0.81054706
9117	2.0818414	2.5688151	KIAA1586	0.810428646
9118	15.226572	18.792084	TMEM39B	0.81026522
9119	120.4767	148.72387	PDIA3	0.810069731
9120	25.968349	32.058282	DHX38	0.810035596
9121	32.539571	40.191705	ZNF622	0.80960912
9122	14.562267	17.989003	ORC6	0.809509375
9123	12.228998	15.116089	PJA1	0.809005479
9124	3.1702926	3.9188731	AKAP11	0.808980676
9125	3.8421311	4.7495334	NT5DC3	0.808949161
9126	3.8382355	4.7449605	PPIP5K2	0.808907785
9127	2.2292404	2.7560579	ADRBK2	0.808851059
9128	4.6577768	5.7585099	MKL2	0.808851059
9129	15.306887	18.929238	GLRX	0.808637247
9130	4.1696919	5.1573425	ATP11B	0.808496222
9131	10.001315	12.371799	EMC9	0.808396223
9132	38.906238	48.139486	DNAJA3	0.808198031
9133	8.8211429	10.91781	NABP1	0.807959015
9134	1.5657363	1.9382513	ATP7B	0.807808726
9135	1.0759991	1.3319973	PTPRN2	0.807808726
9136	3.6080246	4.4664343	SOCS2	0.807808725
9137	5.3600694	6.6353201	KLHDC4	0.807808725
9138	6.0370655	7.4733848	TSPAN15	0.807808725
9139	3.7787761	4.6778104	CCP110	0.807808725
9140	18.396033	22.776196	SNTA1	0.807686792
9141	24.115795	29.861437	VPS52	0.807589906
9142	11.132674	13.78535	SQRDL	0.807572869
9143	91.159257	112.90523	SF3A2	0.807396228
9144	8.1028455	10.036148	HERPUD2	0.807366091
9145	2.8182139	3.490627	POT1	0.80736609
9146	33.635991	41.675603	RPA2	0.807090673
9147	1.7489599	2.1671812	ANKRD32	0.80702062
9148	51.43477	63.752014	PFDN6	0.806794436
9149	14.02499	17.384696	TMEM246	0.806743483
9150	3.6372577	4.5085679	GPD1L	0.806743483
9151	5.4677805	6.7785946	RBFA	0.806624503
9152	41.6799	51.696453	POLR2I	0.806242929
9153	3.334392	4.1361123	FAM173B	0.806165725
9154	6.0046207	7.4483702	NT5DC1	0.806165725
9155	2.4633511	3.0559957	LOC388692	0.806071502
9156	21.165574	26.26014	CCNY	0.805996234
9157	14.165444	17.580182	GGA2	0.80576207

9158	4.4110203	5.4760634	CCDC88A	0.805509345
9159	33.866888	42.046263	CNOT7	0.805467251
9160	12.624935	15.674051	AKAP8	0.805467251
9161	20.244523	25.145493	C8orf59	0.805095475
9162	20.455241	25.409004	ESRRA	0.805039096
9163	83.116058	103.24907	LAMTOR5	0.805005371
9164	27.042716	33.618354	KIAA0100	0.804403321
9165	0.2452171	0.3049135	RGS5	0.804218465
9166	0.9002138	1.1193648	GCA	0.804218465
9167	0.6165666	0.7666655	PIN4P1	0.804218465
9168	1.349488	1.6780117	CMTM1	0.804218464
9169	13.974311	17.376262	CPSF3	0.804218464
9170	1.4587965	1.8139306	GCSHP3	0.804218464
9171	3.4044259	4.2332103	CENPI	0.804218464
9172	4.0310619	5.0123966	TROVE2	0.804218464
9173	2.6143306	3.2507717	ZEB1-AS1	0.804218464
9174	1.2779645	1.5890763	SLC29A3	0.804218464
9175	1.5765092	1.9602997	ZNF620	0.804218464
9176	1.3716939	1.7056235	ELL3	0.804218464
9177	0.7384442	0.9182134	FBXL19-AS1	0.804218464
9178	0.8777356	1.0914143	SWSAP1	0.804218464
9179	0.2866568	0.3564415	XK	0.804218464
9180	0.3339736	0.4152772	FAM110B	0.804218464
9181	154.91469	192.68229	WDR1	0.803990285
9182	14.803072	18.417432	PCIF1	0.803753329
9183	46.338013	57.652277	DPP9	0.803749915
9184	4.8298957	6.0115037	NOTCH2NL	0.803442192
9185	20.481749	25.493271	TOX4	0.803417849
9186	4.4302988	5.5148853	REV1	0.803334708
9187	8.4219918	10.484647	PPP1R16A	0.803268974
9188	14.806918	18.433325	THYN1	0.803268974
9189	23.24456	28.940204	GTF2E2	0.803192675
9190	5.3239534	6.6292282	PHF20L1	0.803103043
9191	4.4049214	5.4864906	SCAMP1	0.802866837
9192	6.9431434	8.6479389	SHOC2	0.802866837
9193	12.229728	15.232573	PLRG1	0.802866836
9194	12.793967	15.947851	TAF12	0.802237631
9195	19.4567	24.256305	ZMYND19	0.802129585
9196	12.364992	15.417521	OBSL1	0.802009073
9197	65.258727	81.388631	TP53	0.801816253
9198	23.214911	28.958767	IRF2BP2	0.801653992
9199	12.866895	16.053534	LUZP1	0.801499214
9200	12.14358	15.153973	HDDC3	0.801346256

9201	53.480004	66.737698	PDCD5	0.801346256
9202	647.8354	808.44836	RPL18	0.801331822
9203	37.907794	47.316788	MRPS12	0.801148928
9204	16.6659	20.80683	ERCC3	0.800982173
9205	14.399463	17.977258	CWC15	0.800982173
9206	6.9006457	8.6152301	MBIP	0.800982173
9207	3.4072451	4.2538339	TRAPPC6B	0.800982173
9208	12.670775	15.821868	HMG20A	0.800839395
9209	10.529874	13.149798	ELL	0.800763176
9210	3.3748907	4.2159131	C1orf63	0.800512388
9211	2.1793412	2.7224328	CCDC111	0.800512388
9212	1.5489588	1.9349592	MAB21L3	0.800512388
9213	0.9878704	1.2340477	ZNF805	0.800512388
9214	5.2180152	6.521579	CRBN	0.800115309
9215	19.744378	24.684081	REEP4	0.799883055
9216	1.4044016	1.7557587	PRKX	0.799883055
9217	13.735175	17.181718	PTPN14	0.799406387
9218	12.227522	15.296042	PGP	0.799391223
9219	14.64889	18.32647	ZMYND8	0.799329598
9220	1.614028	2.0200695	PEX1	0.798996267
9221	2.1661825	2.7111297	ZNF23	0.798996267
9222	2.1504046	2.6913825	ANKRD46	0.798996266
9223	1.6103598	2.0154785	C4orf29	0.798996266
9224	1.1952738	1.4959692	ZNF530	0.798996266
9225	35.842077	44.865699	NIPA2	0.79887482
9226	6.7145915	8.4061269	ATG7	0.798773519
9227	5.0000495	6.2615603	POC1B	0.798530922
9228	4.9079592	6.1462357	RNF139	0.798530922
9229	101.84938	127.54594	SARS	0.798530922
9230	12.941297	16.208226	EXOC4	0.798440056
9231	7.1861897	9.0076077	TNKS	0.797791144
9232	6.0189733	7.5501195	FBXO22	0.797202382
9233	4.2742691	5.3626922	NDUFAF6	0.797037942
9234	13.960377	17.515323	SIRT6	0.797037942
9235	2.9611296	3.7151677	BRMS1L	0.797037942
9236	51.718999	64.907868	BRD4	0.796806313
9237	5.5348144	6.9473076	GFOD2	0.796684802
9238	7.4242322	9.3215879	NKAP	0.796455738
9239	6.2609292	7.8637475	TXNL4B	0.79617628
9240	8.5309737	10.714931	TTC5	0.796176279
9241	12.853523	16.145051	PPP1R8	0.796127736
9242	5.8656605	7.3681355	ARFIP1	0.796084776
9243	239.43772	300.8693	PHB2	0.79581973

9244	0.455319	0.572454	ZNF765	0.7953809
9245	1.7751235	2.2317905	PET117	0.795380899
9246	1.0809103	1.3589844	TSTD3	0.795380899
9247	2.1810518	2.7421476	CACNA2D1	0.795380899
9248	7.441276	9.3556132	POP1	0.795380899
9249	7.3863114	9.2865084	TRABD2A	0.795380899
9250	1.5334768	1.9279779	IDI2	0.795380898
9251	83.209331	104.63025	SNRPD3	0.795270276
9252	46.805353	58.863675	REXO2	0.795148331
9253	4.9243434	6.194289	TGFBR1	0.79498121
9254	76.374442	96.152059	ZNF207	0.794308957
9255	6.8562202	8.6343575	STAU2	0.794062588
9256	11.609444	14.620909	SEC23IP	0.794030252
9257	10.934934	13.772641	DUSP11	0.793960576
9258	2.5045217	3.154466	TRIM39	0.793960576
9259	11.809305	14.876151	CNPPD1	0.793841452
9260	3.7379193	4.7117191	STARD8	0.793323879
9261	57.022807	71.885578	FKBP4	0.793244055
9262	6.2522083	7.882242	ANKRD33B	0.793201773
9263	10.634964	13.410277	ZNF592	0.793045836
9264	18.952036	23.898118	MKRN2	0.793034642
9265	20.95772	26.429534	AFAP1	0.792965932
9266	1.1087644	1.3986665	ZNF550	0.79272963
9267	1.4766446	1.8627343	BCL2	0.792729629
9268	20.926145	26.397581	DYNC1LI2	0.792729629
9269	5.7518262	7.2557225	LARP7	0.792729629
9270	5.652914	7.1338471	PAQR7	0.792407512
9271	5.6479424	7.128702	POLR3F	0.792282012
9272	18.773268	23.702278	POGK	0.792044864
9273	3.2143882	4.0585899	ANGEL2	0.791996299
9274	5.4541026	6.8912518	PLK3	0.791453092
9275	167.72358	211.9715	UBA52	0.791255312
9276	8.4047461	10.622366	SUCLA2	0.791231086
9277	15.407251	19.473307	IPO9	0.791198479
9278	10.428186	13.182405	DONSON	0.791068593
9279	7.5781636	9.5800665	PPM1B	0.791034555
9280	59.90431	75.73344	SRRM1	0.790988897
9281	24.031876	30.393082	TIMM22	0.790702188
9282	0.6989663	0.8839818	ZNF841	0.790702188
9283	2.3712853	2.9989614	ZNF586	0.790702188
9284	33.110201	41.880593	UBTF	0.790585779
9285	51.958299	65.722124	ERP29	0.790575473
9286	42.000136	53.126678	SNRPB2	0.790565813

9287	11.397916	14.418321	ST5	0.790516184
9288	36.7355	46.474111	CASC3	0.790450852
9289	6.8391512	8.6577384	CREBL2	0.789946619
9290	9.0513076	11.45942	FAM111A	0.78985742
9291	6.2736576	7.9427723	RAB28	0.78985742
9292	3.8022498	4.8154351	ZNF707	0.78959631
9293	37.79371	47.865769	ADD1	0.789576989
9294	10.383132	13.151467	GFM2	0.7895037
9295	9.8312962	12.45325	VANGL1	0.789456274
9296	5.8024959	7.3505685	HTRA2	0.789394161
9297	57.849351	73.290126	RNH1	0.789319841
9298	1.1705569	1.4834046	ZNF331	0.789101576
9299	5.3768554	6.8138952	PCGF6	0.789101576
9300	3.6806245	4.6643229	SLC7A6OS	0.789101576
9301	1.4499965	1.8375284	EDNRA	0.789101576
9302	24.051842	30.485015	PSMG3	0.788972617
9303	48.401627	61.367931	TMEM106C	0.788712061
9304	65.225146	82.703352	TMX2	0.788663875
9305	3.1433421	3.9875284	ARHGAP12	0.788293346
9306	1.2028831	1.5268776	AGAP6	0.787805843
9307	1.2076755	1.5329608	LOC90246	0.787805843
9308	1.1732555	1.4892698	PRMT10	0.787805842
9309	7.9005519	10.028552	KAT6A	0.787805842
9310	107.47794	136.4411	CTDNEP1	0.78772412
9311	11.088104	14.078103	TOLLIP	0.787613507
9312	8.7745064	11.141513	MED13	0.787550724
9313	12.498868	15.870557	ACTR10	0.787550724
9314	14.997493	19.046198	CSTF2T	0.78742709
9315	8.8931406	11.294969	MUTYH	0.787354119
9316	9.2376887	11.734178	NCKIPSD	0.787246322
9317	3.5870316	4.5574437	MYRF	0.787070949
9318	6.4807162	8.2339669	HOXC9	0.787070949
9319	19.399172	24.649983	ZFAND5	0.786985211
9320	26.03102	33.079022	HOXB7	0.786934376
9321	20.543647	26.108884	ZMPSTE24	0.786845104
9322	7.2153706	9.1749715	DENND6A	0.786418861
9323	10.698427	13.606606	TUT1	0.786267159
9324	1.8331438	2.3327297	ATG16L2	0.785836328
9325	3.327387	4.2341985	ATPBD4	0.785836328
9326	5.1503519	6.5539753	ISY1	0.785836328
9327	5.8151065	7.4024251	ALG13	0.785567758
9328	2.5510297	3.2473706	ZNF451	0.785567758
9329	36.508391	46.501881	ITPK1	0.785094923

9330	2.7544744	3.5085699	C10orf88	0.785070406
9331	7.5214608	9.5806195	C18orf21	0.785070406
9332	42.990074	54.77561	SUMO1	0.784839706
9333	11.525021	14.686636	IMPA2	0.784728476
9334	12.028036	15.332436	GNB4	0.784483042
9335	1.9762022	2.5193481	ZNF426	0.784410128
9336	2.8637033	3.650773	AUH	0.784410128
9337	15.585138	19.874672	PAK1IP1	0.784170832
9338	3.5195029	4.4901065	LOC648987	0.783835047
9339	2.5865823	3.3006457	FNIP2	0.783659496
9340	6.5608938	8.3721231	MED4	0.783659496
9341	2.3217413	2.9639389	TRIM52	0.783329673
9342	1145.5072	1462.4978	ENO1	0.783253976
9343	22.441347	28.657091	C7orf26	0.783099282
9344	5.233471	6.6836537	PIGX	0.783025467
9345	3.9125659	4.9976441	MIER1	0.782882056
9346	4.8478919	6.1934577	NFYB	0.782744006
9347	5.8386892	7.4617474	PKNOX1	0.78248283
9348	391.21199	500.01666	ATP5B	0.782397907
9349	41.918633	53.578589	CARM1	0.782376572
9350	6.8109529	8.7069881	CAPN10	0.782239823
9351	9.1986047	11.762724	SLC30A5	0.782013153
9352	82.11542	105.0059	HADHA	0.782007681
9353	25.216177	32.253949	CFDP1	0.781801225
9354	6.966626	8.910994	C3orf38	0.781801225
9355	10.693302	13.680767	GATC	0.781630246
9356	7.521359	9.6241615	STK11IP	0.781507976
9357	35.817076	45.842063	ETV5	0.781314677
9358	3.497614	4.478841	ALG9	0.780919428
9359	199.44983	255.42348	UCHL1	0.780859422
9360	16.354776	20.945638	IVNS1ABP	0.78082015
9361	2.3274318	2.9809371	ASPHD2	0.780771862
9362	2.0090624	2.5731747	FAR2	0.780771862
9363	17.229158	22.069223	DCAF15	0.780687115
9364	4.4325735	5.6789603	ARHGAP29	0.780525536
9365	31.866914	40.847683	POLR3K	0.780140067
9366	4.4376835	5.6907627	XPR1	0.77980469
9367	21.712673	27.848914	RAB11A	0.779659607
9368	11.626856	14.927035	HIRIP3	0.77891266
9369	28.658605	36.813041	RNF26	0.778490571
9370	55.737677	71.630076	RCC2	0.778132326
9371	4.9452701	6.3581537	EXOC1	0.777783976
9372	36.46482	46.889919	LSMD1	0.777668635

9373	108.8553	139.98652	SNRPC	0.777612741
9374	23.356561	30.042588	PDLIM5	0.777448363
9375	30.36334	39.056271	VPS37B	0.777425472
9376	13.242052	17.058086	RECQL4	0.776291757
9377	54.85312	70.664642	TUBG1	0.776245648
9378	84.552402	108.98075	TIMM23	0.775847121
9379	0.1472788	0.1899155	ZNF492	0.77549638
9380	0.2459475	0.3171485	SMIM17	0.775496379
9381	0.1768095	0.2279953	VWCE	0.775496378
9382	0.1497661	0.1931229	ANKHD1-EIF4EBP3	0.775496378
9383	0.201417	0.2597266	DMGDH	0.775496378
9384	0.359723	0.4638616	USP44	0.775496378
9385	0.3237693	0.4174994	PRRT1	0.775496378
9386	0.2763919	0.3564064	CCNB3	0.775496377
9387	0.3524231	0.4544484	TRPC3	0.775496377
9388	0.1032192	0.1331008	ZDHHC15	0.775496377
9389	0.574631	0.7409847	LOC642799	0.775496377
9390	0.8880661	1.1451582	HOXC-AS5	0.775496377
9391	0.5025711	0.6480638	CCR10	0.775496377
9392	0.6962121	0.8977632	LOC100132356	0.775496377
9393	0.4007683	0.5167894	LTB4R2	0.775496377
9394	0.3593095	0.4633284	LOC400752	0.775496377
9395	20.051597	25.856467	ARHGDIB	0.775496377
9396	1.4365774	1.8524618	ZFP69	0.775496377
9397	0.9325753	1.2025528	TMOD1	0.775496377
9398	0.2431733	0.3135711	RGL3	0.775496377
9399	0.1911922	0.2465417	SP140	0.775496377
9400	0.5607161	0.7230416	GDPD3	0.775496377
9401	0.8269821	1.0663907	ZNF124	0.775496377
9402	0.8051494	1.0382375	CCDC74B	0.775496377
9403	0.6156558	0.7938862	LOC100507117	0.775496376
9404	16.142599	20.815828	PDCD7	0.775496376
9405	0.8132135	1.048636	LRTOMT	0.775496376
9406	12.734402	16.420969	DNAJC17	0.775496376
9407	2.6683675	3.440851	DET1	0.775496376
9408	0.9180595	1.1838346	C17orf67	0.775496376
9409	0.1333615	0.1719692	C17orf104	0.775496376
9410	0.5699166	0.7349056	ZNF665	0.775496376
9411	1.4150098	1.8246504	FRAT1	0.775496376
9412	4.6311	5.9717881	SNORA70	0.775496376
9413	5.2845127	6.8143615	FAIM	0.775496376
9414	0.763679	0.9847615	ASB3	0.775496376
9415	3.086406	3.9799103	DGKQ	0.775496376

9416	13.371881	17.242996	MED20	0.775496376
9417	4.8758982	6.2874545	BACH1	0.775496376
9418	0.9866362	1.2722642	C8orf44	0.775496376
9419	0.9753487	1.2577089	GNB3	0.775496376
9420	6.5260804	8.415359	FITM2	0.775496376
9421	1.1672862	1.5052117	ZNF17	0.775496376
9422	9.2456688	11.922259	NCK1	0.775496376
9423	1.7093602	2.2042143	SCN1B	0.775496376
9424	4.0823119	5.2641276	AFTPH	0.775496376
9425	8.1676764	10.532192	NSRP1	0.775496376
9426	0.4986429	0.6429984	ANKRD36	0.775496376
9427	0.8528148	1.0997018	KLHL24	0.775496376
9428	0.2210744	0.2850747	CCDC102B	0.775496376
9429	1.265584	1.6319664	LOC400927	0.775496376
9430	2.3808016	3.0700358	C15orf61	0.775496376
9431	1.0265985	1.3237954	CBWD2	0.775496376
9432	0.5805	0.7485528	PRORSD1P	0.775496376
9433	0.448171	0.577915	MTRNR2L6	0.775496376
9434	0.3677638	0.4742302	WWTR1-AS1	0.775496376
9435	17.846627	23.013166	DYNC1I2	0.775496376
9436	1.1818497	1.5239913	FAM212A	0.775496376
9437	0.3807543	0.4909814	PRDM7	0.775496376
9438	0.3909445	0.5041217	ICA1L	0.775496376
9439	0.3591031	0.4630623	LOC100130691	0.775496376
9440	0.4543594	0.5858949	ZFP37	0.775496375
9441	0.1104786	0.1424618	PDE6A	0.775496375
9442	0.3605528	0.4649316	AP4B1-AS1	0.775496375
9443	0.10527	0.1357453	NPAS3	0.775496375
9444	0.1152227	0.1485793	RGAG1	0.775496375
9445	0.1583984	0.2042542	CECR1	0.775496375
9446	0.2577075	0.332313	ZNF221	0.775496375
9447	0.2726553	0.351588	TNFSF13	0.775496375
9448	0.12484	0.1609807	CELF5	0.775496373
9449	60.068552	77.483445	CSNK1A1	0.775243689
9450	42.214673	54.461802	TIMM50	0.775124436
9451	20.404045	26.325898	CPSF1	0.775056004
9452	14.703718	18.974803	CENPN	0.774907541
9453	14.551469	18.779155	ADD3	0.774873488
9454	19.424892	25.069069	BCAR3	0.77485494
9455	11.797279	15.231013	CNOT2	0.774556381
9456	13.05482	16.859808	UFM1	0.774316017
9457	12.280819	15.860215	RHOBTB3	0.774316016
9458	4.8748421	6.2964317	STX3	0.774222983

9459	7.4698061	9.6496769	KLHL5	0.774099086
9460	12.575004	16.246372	LONP2	0.774019241
9461	10.039179	12.973387	TOE1	0.773828642
9462	6.9256631	8.9509165	CRY1	0.773737881
9463	74.525473	96.319424	HNRNPR	0.773732548
9464	24.83298	32.113017	SV2A	0.773299503
9465	18.135316	23.46398	PDCD6IP	0.772900262
9466	18.897522	24.453199	PPP3CB	0.772803681
9467	2.6107528	3.3789341	MAML2	0.77265573
9468	36.635477	47.423023	TSPAN4	0.772525126
9469	54.18387	70.151654	SRPR	0.772381933
9470	3.2248077	4.177454	ADARB1	0.771955297
9471	13.44092	17.416157	COQ4	0.771750017
9472	40.196907	52.085399	WRNIP1	0.771750017
9473	11.486587	14.889709	NCEH1	0.771444671
9474	1.7562877	2.2775949	SPAST	0.771115041
9475	29.010514	37.631152	RACGAP1	0.770917493
9476	13.0648	16.949743	FAM58A	0.770796398
9477	20.422981	26.499962	TM9SF1	0.770679629
9478	9.2648061	12.02255	SYF2	0.770619041
9479	3.77325	4.896783	UBA6	0.770556909
9480	1.5350225	1.9924288	ZMYM6	0.770427772
9481	18.772017	24.368261	TGIF1	0.770346998
9482	11.685953	15.173644	PRTFDC1	0.770148125
9483	15.598222	20.260032	SRP54	0.769901165
9484	7.5045343	9.7501065	CAMSAP2	0.769687415
9485	8.4156395	10.934778	PHTF1	0.769621404
9486	15.225534	19.786661	SDF2L1	0.769484777
9487	5.3392986	6.938797	OIP5	0.769484776
9488	9.2291207	11.996896	PLTP	0.769292406
9489	62.751405	81.570893	SRSF3	0.769286728
9490	2.2942374	2.9826608	WDR90	0.769191528
9491	21.80925	28.357313	SS18	0.769087315
9492	17.004492	22.109963	SNRNP25	0.769087315
9493	17.790201	23.140235	RGL2	0.768799516
9494	5.3244157	6.9260429	C22orf29	0.76875293
9495	51.871867	67.476632	MAF1	0.768738238
9496	24.310834	31.632012	SCAF4	0.768551633
9497	2.614501	3.4020394	ZFP90	0.768509923
9498	37.201894	48.415902	SPG21	0.768381731
9499	15.317931	19.935313	ZNF22	0.76838173
9500	32.42549	42.208086	NAA20	0.768229342
9501	4.9505592	6.4451116	THAP1	0.768110697

9502	3.9926275	5.1989552	MTF1	0.767967285
9503	60.125039	78.352342	DTYMK	0.767367484
9504	1.7192211	2.241027	LOC100009676	0.767157706
9505	0.6823044	0.8893926	ZNF84	0.767157705
9506	28.522011	37.195525	PMPCA	0.766812954
9507	2.4469609	3.1912041	HECA	0.766782934
9508	8.0804134	10.540825	METRN	0.766582625
9509	1.8275066	2.3839656	PLEKHM3	0.766582625
9510	17.513622	22.847776	BTBD1	0.766535085
9511	7.7293027	10.084444	TMEM129	0.766457957
9512	43.401561	56.630925	MLEC	0.766393289
9513	8.2990951	10.829056	RAD54L	0.76637289
9514	8.025693	10.47414	CDK13	0.766238844
9515	5.2434333	6.8448637	UHRF1BP1	0.766039103
9516	0.6080219	0.7938427	CUX2	0.765922347
9517	9.2648227	12.099149	ERBB2IP	0.765741705
9518	201.54747	263.27128	HNRNPL	0.76555054
9519	7.7014708	10.060717	MASTL	0.765499185
9520	4.1830641	5.4669398	LYRM2	0.765156425
9521	28.090279	36.711812	ZNF511	0.765156425
9522	37.671796	49.248423	MRPL10	0.76493406
9523	41.284499	53.98603	TMEM230	0.764725593
9524	5.0326992	6.5818315	FBXL5	0.764635083
9525	15.943131	20.852309	FAM98A	0.764573892
9526	2.4520982	3.2084721	ZFYVE16	0.764257298
9527	0.4089095	0.5350416	ZNF780B	0.764257298
9528	7.9494712	10.405463	ITGA4	0.763970952
9529	5.8756908	7.6975882	HELZ2	0.763315805
9530	39.576966	51.855463	GORASP2	0.763216896
9531	5.1703758	6.7747176	METTL6	0.76318691
9532	3.0323326	3.9732502	WHAMM	0.76318691
9533	3.14527	4.1212315	DCLRE1C	0.76318691
9534	121.65702	159.47105	BAG6	0.762878362
9535	7.6023887	9.9674847	KDM2B	0.762718869
9536	62.133463	81.487048	AARS	0.762494958
9537	19.94425	26.160795	MTHFD1L	0.762371714
9538	37.76845	49.544627	DAXX	0.762311739
9539	36.414634	47.78074	FXR2	0.762119516
9540	18.8069	24.681208	SART3	0.761992708
9541	0.4874028	0.6397275	ANKRD36B	0.761891177
9542	13.851326	18.18019	CDC16	0.761891177
9543	0.8227843	1.0799236	NR4A2	0.761891177
9544	11.639334	15.276898	TPRKB	0.761891177

9545	1.5054659	1.9759593	RAB33B	0.761891177
9546	4.5468982	5.9679103	FAM206A	0.761891177
9547	2.8395066	3.7269188	C1orf56	0.761891177
9548	5.2854946	6.9373353	ZNF692	0.761891177
9549	2.7524462	3.6126501	LRRC1	0.761891177
9550	3.85925	5.0653559	ZNF251	0.761891177
9551	0.9200861	1.2076345	TYW1B	0.761891177
9552	1313.1362	1723.5523	RPL35	0.761877779
9553	107.35037	140.93945	TOMM40	0.761677258
9554	32.849756	43.130064	PPP6R3	0.761644034
9555	15.668444	20.574722	IL6ST	0.761538559
9556	4.4985123	5.9076738	VTI1A	0.76146931
9557	93.028612	122.19537	CLPTM1L	0.761310467
9558	6.1919142	8.1334164	SOCS5	0.761293146
9559	9.2224349	12.11417	AKIP1	0.761293146
9560	16.533767	21.718004	SMYD2	0.761293146
9561	12.719822	16.712815	MRPL42	0.761081946
9562	35.831922	47.088849	CDC42SE1	0.760942825
9563	18.622934	24.482021	SMYD5	0.760677974
9564	15.366657	20.204879	ARL16	0.760541901
9565	1323.865	1741.0205	RPL37A	0.76039602
9566	20.075819	26.402768	IWS1	0.760367948
9567	4.3111192	5.6703573	TANGO6	0.760290565
9568	2.5060065	3.2961168	CLUAP1	0.760290565
9569	1.2152992	1.5984667	CDC14A	0.760290565
9570	23.086544	30.370224	IGBP1	0.760170361
9571	64.819911	85.270243	MAD2L2	0.760170361
9572	10.736799	14.128776	RP9	0.75992416
9573	15.699157	20.668199	NAMPT	0.759580315
9574	14.781051	19.464867	UGGT1	0.759370743
9575	5.4153992	7.133638	TRMT12	0.759135693
9576	9.9869141	13.163006	USP21	0.758710741
9577	27.672319	36.472818	SCAP	0.758710741
9578	4.7183217	6.2188677	C22orf46	0.758710741
9579	2.7420987	3.6162891	ARL9	0.758263124
9580	5.55732	7.3290126	IBTK	0.758263124
9581	2.6733464	3.5256183	ZNF195	0.758263123
9582	1.9762022	2.6062222	RGS4	0.758263123
9583	1.0458004	1.3792052	LOC284889	0.758263123
9584	8.0097642	10.563304	KIAA1704	0.758263123
9585	1.8302551	2.4137467	TUBD1	0.758263123
9586	10.506125	13.859209	PPP1R26	0.758060974
9587	15.56021	20.528406	6-Mar	0.757984325

9588	5.1043511	6.7346077	FAM126A	0.757928497
9589	4.056156	5.3518197	DOCK7	0.757902218
9590	6.52219	8.6105886	NKIRAS1	0.757461577
9591	8.9883962	11.867247	FNBP4	0.757412099
9592	17.065786	22.535271	OXLD1	0.757292236
9593	3.6635477	4.8376934	LRRC40	0.757292236
9594	1242.0698	1640.4285	PKM	0.757161787
9595	7.0093351	9.2589659	ZNF32	0.757032177
9596	3.1610727	4.1756121	LDB2	0.757032177
9597	12.17851	16.093367	RNF20	0.756740943
9598	104.54184	138.15887	GLTSCR2	0.756678457
9599	31.534135	41.694474	DTD1	0.756314487
9600	4.3416563	5.7413715	CCDC91	0.756205422
9601	5.4753756	7.2405929	RALGDS	0.756205422
9602	14.279563	18.88408	DHX37	0.756169364
9603	226.54177	299.65176	MDH2	0.756016834
9604	2.380614	3.1491851	SNX30	0.755946047
9605	2.2063097	2.9198982	TYW5	0.755611854
9606	5.0973993	6.7485743	FILIP1L	0.755329804
9607	65.461779	86.696857	RAB13	0.7550652
9608	22.518046	29.822978	MAD2L1	0.755056937
9609	9.3046872	12.327085	TBPL1	0.754816473
9610	2.8451774	3.7693631	TEP1	0.754816473
9611	25.206685	33.402483	ZMIZ2	0.75463507
9612	3.6231723	4.8018483	LACTB2	0.754537015
9613	3.286243	4.3566331	GNPTAB	0.754307951
9614	146.17879	193.8331	PPIB	0.75414772
9615	2.6125299	3.4660269	EIF4E3	0.753753487
9616	6.0339736	8.0056107	MLL5	0.75371809
9617	9.115244	12.099785	RANGRF	0.753339337
9618	4.5657521	6.0621521	CENPL	0.753156975
9619	6.8346695	9.0746946	FBXL6	0.753156975
9620	10.369674	13.770815	PISD	0.75301822
9621	4.6670536	6.1986946	LRIF1	0.752909103
9622	9.6125231	12.767176	GPN3	0.752909103
9623	46.596214	61.894965	RALY	0.75282721
9624	2.8093485	3.7317613	NCOA2	0.752821044
9625	16.400279	21.788956	ZNF581	0.752687659
9626	5.6407156	7.4963478	PRKAB2	0.752461831
9627	11.217403	14.907603	VWA9	0.75246183
9628	19.286189	25.640081	C1orf198	0.752189108
9629	0.6738868	0.8961303	ARHGEF35	0.751996487
9630	1.4176837	1.8852265	ZNF552	0.751996487

9631	1.8111852	2.4085022	LOC254128	0.751996486
9632	0.1712228	0.2276909	ST8SIA1	0.751996486
9633	2.0971019	2.7887124	IMMP1L	0.751996486
9634	2.7973087	3.7198428	RHEBL1	0.751996486
9635	13.967369	18.573716	OSTF1	0.751996486
9636	0.7672324	1.0202606	PHOSPHO1	0.751996486
9637	0.12384	0.1646816	OBSCN	0.751996484
9638	22.898735	30.475985	CXXC1	0.751369822
9639	37.279798	49.619853	SPRY4	0.751308125
9640	2.9725696	3.9567676	GLTSCR1L	0.751262115
9641	6.9388535	9.2366671	MFAP3	0.751229143
9642	6.174206	8.2201123	NGLY1	0.751109698
9643	12.066486	16.067491	KPNA1	0.750987547
9644	3.0843824	4.1086269	NDRG4	0.750708821
9645	4.8824172	6.504652	TRAF3IP1	0.7506039
9646	23.138751	30.83354	AAR2	0.750440938
9647	14.042313	18.717882	GNL1	0.750208451
9648	5.6950303	7.5926625	KLHL42	0.750070266
9649	4.9356515	6.5807759	SMYD4	0.75001058
9650	1.7543835	2.3393947	RNF169	0.749930562
9651	16.498294	22.002307	SLC25A24	0.749843824
9652	41.050639	54.747602	SON	0.74981619
9653	1.0527061	1.40427	CCDC132	0.749646497
9654	4.1911134	5.5907863	UBXN8	0.749646497
9655	5.4792253	7.3090788	LINC00339	0.749646497
9656	92.457443	123.38411	RPL17	0.749346416
9657	34.132383	45.553164	TCF3	0.749286757
9658	0.951389	1.2706277	ZNF234	0.748755122
9659	8.4722533	11.315119	SEC24B	0.748755122
9660	2.6166753	3.4947011	ARSK	0.748755122
9661	4.0773815	5.4455474	PPP1R21	0.748755122
9662	7.235695	9.6636333	VARS2	0.748755122
9663	5.3577396	7.1580736	MCPH1	0.74848904
9664	31.550203	42.168224	PLCG1	0.748198524
9665	12.01178	16.055827	EDEM2	0.748125916
9666	6.4190138	8.5806534	RMND5A	0.748079838
9667	12.885771	17.231572	SRRD	0.747800077
9668	7.2560531	9.7060121	RBBP5	0.747583361
9669	56.163932	75.129409	UBE2I	0.747562545
9670	9.3849943	12.554327	ADCK2	0.747550561
9671	26.785875	35.858739	NCKAP1	0.746983176
9672	0.51486	0.6894453	ZNF566	0.746774289
9673	4.5918534	6.1489174	ARL14EP	0.746774288

9674	2.4673893	3.3040631	CCDC138	0.746774288
9675	0.569637	0.7627967	GAB3	0.746774288
9676	66.096024	88.514235	STT3A	0.746727624
9677	174.42132	233.58738	NDUFB9	0.746706945
9678	90.937964	121.79662	TFG	0.746637841
9679	9.6711062	12.953691	MTMR2	0.746590761
9680	46.411132	62.171156	APEH	0.746505858
9681	40.060608	53.669965	NUSAP1	0.746425084
9682	11.438738	15.326394	C15orf57	0.746342377
9683	207.36062	277.90695	RPL34	0.746151249
9684	18.508207	24.805889	UBALD1	0.746121514
9685	3.5185935	4.7163151	ESCO2	0.746047147
9686	9.3798349	12.574286	MON1A	0.745953658
9687	14.112327	18.919934	ADSS	0.745897278
9688	3.9763998	5.3314912	TAF1B	0.745832581
9689	34.452526	46.203474	DHPS	0.745669593
9690	16.900923	22.66823	CELF1	0.745577535
9691	1.9957938	2.6773424	LMBRD2	0.745438377
9692	15.058908	20.209331	PDXK	0.745146316
9693	4.9522675	6.6465827	C8orf58	0.745084754
9694	6.3366897	8.5046563	IFT57	0.745084754
9695	4.8660109	6.5316432	USP25	0.744990308
9696	63.457287	85.198715	EIF4A3	0.744815074
9697	3.8612362	5.1865118	CORO1A	0.744476521
9698	94.060019	126.34931	ILF3	0.74444426
9699	8.2081749	11.028391	CNOT4	0.744276715
9700	13.345435	17.933479	UGDH-AS1	0.744163189
9701	10.700776	14.38579	NEIL2	0.743843463
9702	5.2840351	7.1040174	ATP11A	0.743809428
9703	4.3722039	5.8785863	SPG11	0.743750911
9704	14.076408	18.926239	PBDC1	0.743750911
9705	9.098782	12.235112	SMAP1	0.74366155
9706	11.19863	15.06109	PPIL3	0.743547087
9707	21.370188	28.748151	AGTRAP	0.743358689
9708	0.9117726	1.2268463	DSC2	0.743184028
9709	2.7997596	3.7672495	LOC642361	0.743184027
9710	90.928407	122.37741	PSMC1	0.743016258
9711	10.257564	13.80578	PSPC1	0.742990541
9712	11.50851	15.489871	POLE	0.742970038
9713	43.489044	58.546453	LAMTOR1	0.742812621
9714	92.203599	124.1335	PGD	0.742777753
9715	5.8301561	7.8496419	FAM122A	0.742728924
9716	16.048179	21.608123	AXIN1	0.742691961

9717	5.2801669	7.1104115	NFYA	0.74259653
9718	8.3170298	11.199931	BLOC1S6	0.74259653
9719	3.8476385	5.1820289	NKTR	0.74249653
9720	24.937906	33.591308	DDX54	0.742391626
9721	4.2211096	5.6877408	WDR47	0.742141693
9722	8.8429774	11.916124	ZNF281	0.742101795
9723	68.641972	92.500693	CCDC86	0.742069808
9724	6.2208806	8.3834436	ZNF623	0.742043591
9725	252.93539	340.86932	ACTN1	0.742030373
9726	6.9511058	9.3728152	BCL10	0.741624121
9727	3.8105497	5.1386137	RABL3	0.74155209
9728	29.860227	40.271967	UPF1	0.741464322
9729	3.7266734	5.0268404	ZNF232	0.741355026
9730	412.73617	556.79904	RPS19	0.741265943
9731	9.7828562	13.198005	BID	0.7412375
9732	47.648206	64.31241	STARD7	0.740886658
9733	23.817086	32.174532	G3BP2	0.740246541
9734	10.845058	14.652276	FYTTD1	0.740162009
9735	3.7659174	5.0916969	TULP4	0.73961932
9736	7.1112626	9.6157109	WDR24	0.739546213
9737	5.2808561	7.1412851	JRK	0.739482613
9738	108.42594	146.67162	AKT1	0.739242777
9739	27.417183	37.094124	SREBF2	0.739124687
9740	20.562862	27.832126	CCNF	0.738817494
9741	0.2542077	0.34419	LMOD3	0.738567979
9742	0.5305486	0.7183477	LOC646903	0.738567978
9743	0.138896	0.1880612	ZFP14	0.738567978
9744	0.2422687	0.3280249	LOC595101	0.738567978
9745	3.2630402	4.4180635	WBSCR27	0.738567978
9746	2.9720408	4.0240586	CENPQ	0.738567978
9747	7.3071354	9.8936531	ZNF689	0.738567978
9748	1.7660975	2.3912456	PI4KAP1	0.738567978
9749	1.9199743	2.5995906	LOC643406	0.738567977
9750	1.1150321	1.5097217	ESAM	0.738567977
9751	3.8551705	5.219791	DLX6	0.738567977
9752	0.4495244	0.6086432	LOC100506688	0.738567977
9753	0.7458822	1.0099033	RPSAP58	0.738567977
9754	2.9499058	3.9940884	FBXO10	0.738567977
9755	2.5072125	3.3946943	C6orf70	0.738567977
9756	0.2483903	0.3363135	MMRN2	0.738567977
9757	2.6209064	3.5486326	DNAJB14	0.738567977
9758	1.4100102	1.9091136	ALX3	0.738567977
9759	0.320516	0.4339695	MROH6	0.738567977

9760	0.6522676	0.8831518	PRRT2	0.738567977
9761	0.9408555	1.2738916	BFSP1	0.738567977
9762	0.497611	0.6737512	ZNF699	0.738567977
9763	0.7246158	0.9811091	LOC100128822	0.738567977
9764	0.4418989	0.5983185	ZNF350	0.738567976
9765	0.1078337	0.1460038	ADAMTS5	0.738567974
9766	47.91811	64.898787	DDX41	0.738351389
9767	25.551437	34.609571	KIF5B	0.738276629
9768	24.758982	33.536186	MEPCE	0.738276629
9769	9.4852015	12.851556	STX6	0.73805862
9770	5.7071322	7.7330439	ZMYM4	0.738018856
9771	14.008597	18.983967	UBLCP1	0.737917257
9772	7.4584427	10.110591	ZFYVE20	0.737686106
9773	15.478143	20.985615	ZNF410	0.737559694
9774	9.6174387	13.040592	STK35	0.737500168
9775	31.930517	43.302854	NRD1	0.737376739
9776	68.438889	92.823318	EIF4E2	0.737302765
9777	5.6107558	7.6108977	N4BP2L2	0.737200259
9778	6.0852338	8.2545193	FBLN5	0.737200259
9779	33.535552	45.498847	CHTOP	0.737063766
9780	9.5417539	12.946809	DHX34	0.736996556
9781	9.1516227	12.417959	ETV6	0.736966746
9782	113.80232	154.4228	HMGN1	0.736952818
9783	14.042509	19.062034	ARHGEF18	0.736674213
9784	3.3200901	4.5076571	ZNF337	0.736544503
9785	28.496617	38.69496	MRPS28	0.736442602
9786	306.27793	415.9143	DRAP1	0.736396749
9787	5.5683778	7.5623411	TMEM177	0.736329893
9788	6.9042128	9.3765211	ZNF740	0.736329893
9789	17.086456	23.205841	OXSR1	0.736299823
9790	52.195432	70.892925	BRD2	0.736257278
9791	5.2512898	7.1329221	GMIP	0.73620456
9792	9.4658749	12.860119	CDIP1	0.736064357
9793	6.1457513	8.3494755	N4BP1	0.736064357
9794	31.984391	43.455028	SRXN1	0.736034296
9795	325.72106	442.89952	CCND1	0.73542879
9796	10.19375	13.865311	GCLC	0.735198085
9797	10.099739	13.738289	SPTSSA	0.735152634
9798	52.508132	71.43468	PTPN1	0.735050987
9799	3.9220321	5.3360355	PPP2R5A	0.735008614
9800	73.930456	100.5965	NAA10	0.734920728
9801	4.7928335	6.5236952	RPP40	0.734680778
9802	3.0170437	4.1066049	ZBTB12	0.734680777

9803	189.69937	258.23318	RAB7A	0.73460493
9804	18.17467	24.74546	DCTN4	0.734464822
9805	123.15989	167.69533	EIF3H	0.734426475
9806	28.07595	38.237031	YTHDF2	0.73426072
9807	921.08121	1254.5545	RPL19	0.734189868
9808	103.43317	140.88364	SRRM2	0.734174422
9809	4.7458529	6.4685563	SRGAP1	0.733680395
9810	13.780477	18.783075	PROSC	0.733664621
9811	5.3124417	7.241826	ZBTB1	0.733577653
9812	13.656015	18.616305	GGNBP2	0.733551289
9813	5.5468576	7.5626237	CRAMP1L	0.73345678
9814	4.2516286	5.7970284	RINT1	0.733415178
9815	13.373483	18.235281	ADCY3	0.733385044
9816	26.99978	36.822745	LYPLA1	0.733236489
9817	1.7422466	2.376234	EIF2AK3	0.733196574
9818	5.9586367	8.1269293	GTPBP8	0.733196574
9819	17.868188	24.37492	PAFAH1B3	0.733056276
9820	2.6039088	3.5535994	TMTC3	0.732752482
9821	6.3151364	8.618376	ST7	0.732752482
9822	117.4846	160.37947	TKT	0.732541414
9823	1.6537069	2.2578878	FAM73A	0.732413244
9824	2.3020088	3.1430463	IMMP2L	0.732413244
9825	2.8162095	3.84511	DNAJC25	0.732413244
9826	1.9047266	2.6006174	RBM45	0.732413244
9827	14.31272	19.546338	SNAP23	0.732245606
9828	19.419631	26.523397	MAGT1	0.732169837
9829	644.00712	879.67995	RPS15A	0.732092526
9830	12.930014	17.663182	SETD1A	0.732031978
9831	6.3582523	8.6862951	ACVR1B	0.731986679
9832	7.0539934	9.6398897	CXorf40B	0.731750427
9833	6.5210197	8.9151243	PKN2	0.731455841
9834	0.6672097	0.9122833	NBEAL1	0.731362437
9835	5.9778294	8.1735527	HUS1	0.731362436
9836	2.6159788	3.5775385	ITPR1	0.731223102
9837	15.136385	20.700328	RBM15B	0.731214739
9838	17.543835	23.993791	NXT1	0.731182297
9839	38.788013	53.05059	MRPL32	0.731151395
9840	12.844167	17.56969	SUGT1	0.73104117
9841	147.34388	201.63691	ATP5A1	0.730738635
9842	3.4467697	4.7171442	TP73-AS1	0.730689919
9843	27.272985	37.329195	ERF	0.730607365
9844	1.5441766	2.1156613	ICOSLG	0.729878943
9845	4.1158558	5.6390938	SIAE	0.729878943

9846	9.6981259	13.287307	BRD3	0.729878943
9847	0.6914001	0.9472805	IFIH1	0.729878942
9848	3.3680727	4.6145635	GCFC2	0.729878942
9849	1.4593235	1.9994048	RBM41	0.729878942
9850	0.6161109	0.8441275	ZNF449	0.729878942
9851	4.8750472	6.6820811	CDC73	0.729570195
9852	19.66033	26.955067	METTL17	0.729374185
9853	2.6870508	3.6842433	LIN54	0.729335878
9854	17.225224	23.620831	CIAO1	0.729238698
9855	13.069524	17.925578	USE1	0.729099157
9856	16.656687	22.84809	MTOR	0.729018816
9857	49.222233	67.529141	MGLL	0.728903586
9858	4.7538727	6.5235894	HIVEP2	0.728720404
9859	2.9405483	4.0364612	PLEKHA5	0.728496596
9860	3.7702857	5.1754335	THAP5	0.728496596
9861	6.3383709	8.7006184	DSE	0.728496596
9862	19.903323	27.336747	AIFM1	0.728079438
9863	24.728255	33.976151	MRPL47	0.727812133
9864	5.9237509	8.1395527	KLHDC10	0.727773522
9865	1.5968237	2.1945659	EPM2AIP1	0.72762623
9866	71.775684	98.683387	PARP1	0.727332998
9867	23.212816	31.928372	RALA	0.727027853
9868	16.904153	23.257894	CFL2	0.72681358
9869	0.803915	1.1064199	C4orf21	0.7265912
9870	1.4612694	2.0111301	ZFAND4	0.7265912
9871	3.250681	4.4747615	PTPN3	0.726447888
9872	4.5186362	6.2229864	KIAA1715	0.726120203
9873	7.1070842	9.7880996	GPBP1	0.726094385
9874	16.732807	23.048051	SS18L2	0.725996607
9875	48.482429	66.792343	SNRNP200	0.725868061
9876	12.107529	16.683395	ZC3HC1	0.725723317
9877	3.2391062	4.4645894	RASGRF2	0.725510422
9878	2.3907192	3.2959985	TCEANC2	0.725339894
9879	5.2562228	7.2485681	ARMCX1	0.725139469
9880	12.00781	16.564769	RAB6A	0.724900528
9881	2.8461872	3.9265196	NINL	0.724862592
9882	15.445243	21.308455	TMOD3	0.724841057
9883	30.068833	41.485668	CDCA8	0.724800497
9884	13.728557	18.942134	LRCH3	0.724762968
9885	2.4684037	3.4061965	CCNT2	0.724680374
9886	8.8407038	12.203244	RWDD4	0.724455214
9887	4.9883658	6.8877923	ALS2	0.724232903
9888	15.249488	21.060319	ASB1	0.724086252

9889	0.3409997	0.4711263	ZIC4	0.723796619
9890	0.1401207	0.1935912	AKAP6	0.723796619
9891	0.5995875	0.8283923	MSS51	0.723796619
9892	0.4756428	0.6571498	ZNF140	0.723796618
9893	0.4274235	0.5905299	ARMC2	0.723796618
9894	0.7919634	1.0941794	GTF2H2D	0.723796618
9895	0.6249307	0.8634065	RAB27B	0.723796618
9896	0.3376062	0.466438	PRICKLE1	0.723796618
9897	1.5847871	2.1895475	C8orf4	0.723796618
9898	0.191017	0.2639097	MCTP2	0.723796618
9899	0.9016048	1.2456604	FBXL8	0.723796618
9900	0.5660832	0.7821026	ZNF510	0.723796618
9901	0.6025595	0.8324983	LOC90834	0.723796618
9902	1.1745543	1.6227685	SPINT1	0.723796618
9903	2.716567	3.7532188	ABHD5	0.723796618
9904	33.089073	45.715981	EIF1B	0.723796618
9905	2.784722	3.8473819	AGO3	0.723796618
9906	5.2640264	7.2798246	SETD1B	0.723097973
9907	16.810988	23.255521	INPP5A	0.722881578
9908	4.6461902	6.4290687	NPTXR	0.722684795
9909	8.0005655	11.071331	NSD1	0.72263808
9910	2.6174039	3.6224494	SPRTN	0.722550841
9911	135.05122	186.92344	RAC1	0.722494825
9912	77.075342	106.6855	POLD2	0.722453767
9913	8.7010843	12.045021	RNF216P1	0.722380186
9914	18.365448	25.427187	RPF2	0.722276037
9915	169.51336	234.77403	NOP10	0.722027731
9916	20.035438	27.755847	THOC5	0.721845683
9917	3.9427957	5.4630943	RPS6KB1	0.721714749
9918	4.8497534	6.7197649	INPP5B	0.721714748
9919	11.812831	16.369368	CDR2L	0.721642461
9920	11.954318	16.567209	RNASEH2B	0.721564974
9921	12.209531	16.92354	BRPF1	0.721452548
9922	3.8711981	5.3662894	C20orf96	0.721391978
9923	3.2957222	4.5685595	LOC100506668	0.721391978
9924	71.435911	99.042413	CAPN2	0.721265861
9925	70.291251	97.456487	ATP1B3	0.72125779
9926	16.627913	23.056861	FAM107B	0.72116984
9927	5.9051732	8.1888256	RB1CC1	0.721125782
9928	26.138543	36.247763	TGFBR2	0.721107742
9929	4.1445304	5.747706	GSKIP	0.721075578
9930	22.987463	31.884928	RPUSD1	0.72095076
9931	5.1207621	7.1034486	SDE2	0.720883955

9932	13.064387	18.124493	MAPKAPK5	0.720813939
9933	23.688171	32.864027	CMPK1	0.720793312
9934	140.48269	194.90126	NME1	0.720789009
9935	4.6930697	6.512153	RMDN1	0.720663299
9936	35.949134	49.898181	GLTP	0.720449785
9937	9.4471946	13.116893	PLSCR1	0.720231118
9938	1.929625	2.6796485	SCML2	0.720103778
9939	21.604556	30.008617	UAP1	0.71994506
9940	1.7229116	2.3937914	PTPN13	0.719741735
9941	5.6898152	7.9053568	E2F2	0.719741735
9942	13.735422	19.08597	THG1L	0.719660637
9943	33.17673	46.107781	NACA	0.719547323
9944	39.802427	55.319739	RNF126	0.719497732
9945	4.6357826	6.444844	C17orf51	0.719300987
9946	138.1577	192.10029	COX7C	0.719195703
9947	48.423102	67.348557	SRSF1	0.718992417
9948	38.059279	52.937328	CDC34	0.718949766
9949	2.04118	2.8407365	PCMTD1	0.718539015
9950	5.1360594	7.1497556	PYCRL	0.718354538
9951	2.7302555	3.8011471	ZNF587	0.718271453
9952	86.39035	120.28162	NME4	0.718233986
9953	0.993514	1.3836237	ACVR2A	0.7180522
9954	2.5724209	3.5863756	ICK	0.717275928
9955	14.066618	19.620497	WIPI2	0.716934865
9956	4.5003214	6.2774454	MKS1	0.716903317
9957	4.3670112	6.092824	AP1AR	0.716746651
9958	9.5885617	13.381744	GTF2H3	0.716540512
9959	649.48877	906.56489	RPS5	0.716428337
9960	4.6053885	6.4286435	FBXW2	0.716385731
9961	20.797506	29.041772	EMC8	0.716123862
9962	0.5040568	0.7041446	ZNF483	0.715842809
9963	0.92622	1.2938874	ATP6V1E2	0.715842809
9964	35.011116	48.908944	PCNP	0.715842809
9965	3.9391445	5.5028065	CLK1	0.715842809
9966	1.3556888	1.8938359	ZNF18	0.715842809
9967	6.9984907	9.7765747	ZBTB48	0.715842809
9968	4.113148	5.7458816	BCAS4	0.715842809
9969	4.8527697	6.7790996	BLOC1S4	0.715842809
9970	1.7561756	2.4532978	DPH3P1	0.715842809
9971	0.4324638	0.6041324	VASH2	0.715842808
9972	119.0027	166.26472	HSP90B1	0.71574234
9973	33.075998	46.213092	IMMT	0.715727869
9974	11.788359	16.473385	HIRA	0.715600315

9975	7.6155867	10.649689	RBM7	0.715099463
9976	16.550365	23.146037	PERP	0.715041013
9977	67.210761	94.006081	PCNA	0.714961849
9978	32.172786	45.003739	ZC3H18	0.714891396
9979	6.0830712	8.5094478	AGPS	0.714860857
9980	17.79251	24.89601	MPHOSPH6	0.714673131
9981	2.4428923	3.4181952	EEA1	0.714673131
9982	39.452021	55.212439	ATP5E	0.714549507
9983	20.659648	28.917103	EXOC3	0.714443898
9984	40.10755	56.150409	FAM120A	0.714287756
9985	9.0102992	12.618213	NFATC3	0.714070921
9986	14.533771	20.369197	PHF10	0.713517134
9987	5.1063798	7.1572389	NUDT9	0.713456666
9988	9.9163141	13.899852	ESPL1	0.713411514
9989	1160.7593	1627.092	RPL11	0.713395012
9990	1.1571322	1.6226784	FAM161B	0.713100116
9991	19.824166	27.807899	CNOT11	0.712896954
9992	16.195008	22.719313	MRPL39	0.712830003
9993	4.6652779	6.5447271	DPH3	0.712830003
9994	27.996905	39.279902	PEMT	0.712753951
9995	41.91944	58.816964	CCNB2	0.712710018
9996	5.6612645	7.9434355	DHX29	0.712697235
9997	6.3549795	8.9188278	ZZEF1	0.712535284
9998	54.078351	75.899348	MRPL41	0.712500872
9999	7.8158458	10.970411	ZNF639	0.712447891
10000	9.8182135	13.783667	NR2C2AP	0.712307783
10001	9.0165566	12.660936	RAPH1	0.712155613
10002	5.5425399	7.786563	FBXL3	0.711808268
10003	15.078999	21.189322	CCDC59	0.711631969
10004	0.01	0.0140549	CSMD1	0.711493559
10005	11.319282	15.910451	GNL3L	0.711436909
10006	106.27695	149.3894	GARS	0.711408901
10007	19.046075	26.773093	ABRACL	0.711388676
10008	17.381523	24.437028	SRCAP	0.711278123
10009	3.3751009	4.7455517	XPNPEP3	0.711213608
10010	8.0681849	11.344676	TTC19	0.711186921
10011	2.0241894	2.8474751	TMEM143	0.710871679
10012	10.178367	14.31815	TOR1AIP1	0.710871678
10013	3.1759414	4.4676719	C7orf31	0.710871678
10014	2.2430475	3.1553479	FLJ46906	0.710871678
10015	1.511635	2.1264527	ACSS3	0.710871678
10016	3.5458538	4.9880364	THEM4	0.710871678
10017	4.2977025	6.0456797	TSNAX	0.710871678

10018	3.1252822	4.3964084	IPPK	0.710871678
10019	100.26727	141.07924	AXL	0.710715956
10020	200.73572	282.45274	HNRNPD	0.710687795
10021	9.6154195	13.53774	FZD8	0.710267709
10022	2.4764601	3.4884283	NAPB	0.709907131
10023	3.8765783	5.4620889	STARD13	0.709724494
10024	9.4291185	13.292266	WDR41	0.709368779
10025	575.38772	811.15039	PPIA	0.709347777
10026	15.865384	22.372207	PLOD2	0.709155979
10027	13.560093	19.124978	ZC3H11A	0.709025258
10028	1.6048945	2.2645826	IBA57	0.708693318
10029	4.9953715	7.0498803	KLF3	0.708575369
10030	44.159662	62.326593	LAMB1	0.708520394
10031	10.110148	14.272363	RFT1	0.708372381
10032	1.9180241	2.7076494	CEP350	0.708372381
10033	13.518452	19.090353	PPHLN1	0.708130024
10034	21.282457	30.058956	PTPN9	0.70802381
10035	8.8342576	12.47765	VPS11	0.708006544
10036	1.140233	1.6117042	EFCAB7	0.707470379
10037	10.504567	14.848066	FBXL18	0.707470379
10038	0.4687997	0.6626422	CLYBL	0.707470378
10039	8.0905949	11.435949	FBXO9	0.707470378
10040	14.654774	20.717535	EPN2	0.70736088
10041	18.874371	26.683144	POLD1	0.707351814
10042	9.6697368	13.673006	ZNF777	0.707213677
10043	4.9681658	7.0252365	PRKCE	0.707188405
10044	48.65358	68.816434	THOC7	0.707005243
10045	11.497903	16.262826	ARV1	0.707005243
10046	26.49458	37.486239	COQ9	0.706781507
10047	3.1338271	4.4353093	SGTB	0.706563365
10048	12.289709	17.398408	ARAP1	0.706369733
10049	6.8293079	9.6688494	TERF1	0.706320643
10050	11.137604	15.768482	CDK10	0.706320643
10051	33.65625	47.652786	MALSU1	0.706280853
10052	11.122002	15.753077	PPP1R15B	0.706020914
10053	1.3076286	1.8528403	EFHC1	0.705742734
10054	24.425123	34.614542	SEC23B	0.705631838
10055	10.339408	14.65832	VCAN	0.705361045
10056	13.990968	19.835992	FAM213B	0.705332418
10057	4.8371961	6.8589581	SATB2	0.70523773
10058	7.4428393	10.55366	COMMD2	0.70523773
10059	0.3796906	0.5385707	ANKHD1	0.704996706
10060	4.1615165	5.9028879	FCHSD2	0.704996706

10061	12.627954	17.912075	EZH2	0.704996706
10062	61.59714	87.388317	LSM12	0.704866992
10063	10.552673	14.972475	TAF3	0.70480487
10064	197.29159	280.08355	CANX	0.704402636
10065	30.318006	43.042422	TPP1	0.704375015
10066	13.02096	18.486075	SHCBP1	0.704365836
10067	6.3316644	8.9903	PCM1	0.704277321
10068	201.02554	285.4804	HNRNPAB	0.70416581
10069	30.993853	44.023391	EIF2B5	0.704031465
10070	2.5337325	3.5990687	ZNF16	0.703996711
10071	40.367942	57.341095	RNF114	0.70399671
10072	6.4784439	9.2041108	CHURC1	0.703864181
10073	20.316333	28.864893	PPP4R1	0.703842325
10074	8.1432022	11.572125	RRN3	0.703691156
10075	1.8756808	2.6660495	ZBTB8A	0.703543104
10076	39.410794	56.017596	DPM1	0.703543104
10077	15.580294	22.147004	TNIP2	0.70349443
10078	8.5002807	12.08294	CCDC25	0.703494429
10079	21.738687	30.904499	CLK3	0.703414982
10080	3.9588224	5.6287108	BTAF1	0.703326663
10081	2.2940588	3.2626999	MANEA	0.703116715
10082	0.7070029	1.0055271	XRRA1	0.703116714
10083	17.52589	24.932943	MYO1E	0.702921006
10084	7.0192631	9.9876595	FEM1B	0.702793591
10085	21.38203	30.436055	PDRG1	0.702523033
10086	17.823134	25.370703	IDH3A	0.702508482
10087	3.4362055	4.8925355	ATP6V0A2	0.702336341
10088	11.411463	16.252886	HDAC2	0.702119168
10089	3.0256677	4.312282	C1orf27	0.701639579
10090	19.409757	27.66343	NUDT1	0.701639579
10091	7.5229712	10.721988	NUP107	0.701639579
10092	2.5745062	3.6692716	PLEKHH3	0.701639579
10093	10.730598	15.293604	S100A3	0.701639579
10094	4.2821815	6.1031071	PEX3	0.701639578
10095	1.0502893	1.4969071	GRAMD1C	0.701639578
10096	26.591034	37.902359	CRTC2	0.701566742
10097	4.7323191	6.7506801	PAPD4	0.701013674
10098	12.462614	17.782044	XPC	0.700853868
10099	4.1034117	5.8551666	TBK1	0.700818948
10100	13.41013	19.139071	TP53BP2	0.700667779
10101	10.86843	15.511531	METTL3	0.700667778
10102	3.9790389	5.6797229	CREB1	0.70056919
10103	7.2745836	10.38561	FAM175B	0.70044834

10104	8.677663	12.391849	GCLM	0.70027186
10105	97.258743	138.9223	SLC39A1	0.700094548
10106	6.9892862	9.984615	SUV420H1	0.700005579
10107	3.4759583	4.9661145	METTL10	0.699935191
10108	3.7426849	5.3471878	APLP1	0.699935191
10109	9.6809556	13.832093	TRAK1	0.69989088
10110	35.548704	50.793606	AKIRIN1	0.699865726
10111	1.5587605	2.2284965	ZNF227	0.69946732
10112	2.6228118	3.7497274	SLC19A2	0.69946732
10113	25.155403	35.968216	API5	0.699378656
10114	8.8077153	12.59674	TMEM164	0.699205902
10115	29.993366	42.909702	XAB2	0.698987977
10116	1.5697462	2.2468425	DOCK11	0.698645384
10117	1.2857817	1.8403925	OPLAH	0.698645384
10118	4.5454336	6.5105185	RELL1	0.698167678
10119	43.920452	62.92137	CYFIP1	0.698021234
10120	281.42501	403.22318	RPS3A	0.697938566
10121	13.569739	19.445919	ATP6V0A1	0.6978194
10122	23.519492	33.713163	STT3B	0.697635294
10123	6.2841364	9.0102912	TTC37	0.697439878
10124	5.4916724	7.875109	RASA1	0.697345579
10125	4.4598525	6.3964814	WDR81	0.697235274
10126	46.144071	66.213968	NDUFS3	0.696893295
10127	22.40659	32.152519	ADK	0.696884415
10128	3.5006147	5.0236797	CD274	0.696822831
10129	11.39415	16.362346	PRDM4	0.696364093
10130	3.6874979	5.295359	LOC645212	0.696364093
10131	30.698213	44.091224	ANLN	0.696243165
10132	6.0827809	8.7413352	MNT	0.695864047
10133	3.5606939	5.1176126	CNNM2	0.69577245
10134	5.0661858	7.2815925	NFAT5	0.695752443
10135	4.2666314	6.1327482	STK17B	0.695712799
10136	3.0073251	4.3233743	RBMXL1	0.69559675
10137	3.3079286	4.7555262	HEY1	0.69559675
10138	12.532807	18.020244	RBM6	0.695484846
10139	5.8638882	8.4320277	MIS12	0.695430379
10140	242.09622	348.19769	TUBB6	0.695283811
10141	6.3357637	9.1152293	THUMPD3	0.69507453
10142	5.6065482	8.0672039	MTO1	0.694980359
10143	18.89089	27.18721	PRPF38A	0.694844753
10144	8.2495561	11.872517	SPRYD4	0.694844753
10145	3.9730341	5.7189369	TMEM192	0.694715504
10146	16.497699	23.760267	DDX19A	0.694339779

10147	9.4961702	13.6774	HERC2	0.694296467
10148	6.6115737	9.5260556	FRA10AC1	0.694051551
10149	1020.448	1470.3081	RPS14	0.694036861
10150	8.4959029	12.244314	C20orf24	0.693865179
10151	31.266606	45.099508	KLF6	0.693280428
10152	188.94702	272.55149	TRIM28	0.693252575
10153	3.327624	4.800057	POGLUT1	0.693246761
10154	6.7152215	9.6866251	PVRL1	0.693246761
10155	29.910389	43.170896	CAV1	0.692836879
10156	0.7615694	1.0992999	KCNQ1OT1	0.692776763
10157	78.668219	113.55868	FXYD5	0.692753937
10158	7.4155761	10.704922	DDX52	0.692725827
10159	21.653242	31.261709	DDAH1	0.692644199
10160	39.878917	57.585099	SNHG1	0.692521455
10161	66.953052	96.687645	TYMS	0.692467506
10162	41.838681	60.425964	ANAPC5	0.692395748
10163	17.174291	24.815989	ANAPC7	0.692065549
10164	4.3836452	6.3349178	SGOL1	0.691981382
10165	37.851715	54.702873	COMMD4	0.691951139
10166	38.973795	56.325298	EXOSC10	0.691941211
10167	8.2526819	11.934736	CDR2	0.691484269
10168	3.7942397	5.4870947	CEP68	0.691484269
10169	7.1304991	10.314629	SAMD4A	0.691299627
10170	16.092112	23.280255	CHPT1	0.691234339
10171	4.559765	6.5971377	CLCN6	0.691173241
10172	11.748011	17.007364	GPRC5A	0.690760258
10173	28.867652	41.791666	COPS8	0.690751412
10174	107.80414	156.07282	CERS2	0.690729768
10175	85.14085	123.31122	PPT1	0.690454998
10176	156.04842	226.13396	VDAC1	0.69007069
10177	0.0742692	0.1077412	ZNF662	0.689330117
10178	0.13169	0.1910406	LOC100507584	0.689330116
10179	0.1617381	0.2346308	C1orf220	0.689330115
10180	0.1421067	0.2061519	CCL22	0.689330115
10181	0.0598849	0.0868741	LONRF2	0.689330115
10182	0.2072596	0.3006681	FAM83E	0.689330114
10183	0.0797549	0.1156991	ITGAL	0.689330114
10184	0.1991395	0.2888885	ZC2HC1C	0.689330114
10185	0.3143281	0.4559906	TTC30B	0.689330114
10186	0.155871	0.2261195	PCSK4	0.689330114
10187	0.1868216	0.2710191	LINC00470	0.689330114
10188	0.1344947	0.1951092	C17orf107	0.689330114
10189	0.1195294	0.1733994	SALL4	0.689330114

10190	0.1650036	0.239368	SEC14L4	0.689330113
10191	0.084492	0.1225712	SYNPO2L	0.689330113
10192	0.2092364	0.3035359	IAPP	0.689330113
10193	0.2131964	0.3092806	RHBG	0.689330113
10194	0.260608	0.3780597	TSG1	0.689330113
10195	0.0991434	0.1438258	LOC728377	0.689330113
10196	0.0641229	0.0930221	CDHR3	0.689330113
10197	0.2683831	0.389339	FAM186B	0.689330113
10198	0.197348	0.2862896	LOC100287792	0.689330113
10199	0.1624314	0.2356366	LINC00574	0.689330113
10200	0.4220037	0.6121939	NEK11	0.689330113
10201	0.2916718	0.4231235	DNAJC9-AS1	0.689330113
10202	0.736394	1.0682748	RPS26P11	0.689330113
10203	0.3691754	0.5355567	C1orf229	0.689330113
10204	0.2884422	0.4184384	LINC00862	0.689330113
10205	0.3490779	0.5064016	LOC254100	0.689330113
10206	0.7258777	1.0530191	B3GALT5	0.689330113
10207	0.1312753	0.1904389	ASB14	0.689330113
10208	0.925192	1.342161	OAZ3	0.689330113
10209	0.9353658	1.35692	ZNF256	0.689330113
10210	0.696406	1.0102649	TDRG1	0.689330113
10211	0.3990416	0.5788832	PLSCR3	0.689330113
10212	0.4434032	0.6432378	TMED6	0.689330113
10213	0.6337542	0.9193769	GRASP	0.689330113
10214	13.695022	19.867146	BRAT1	0.689330113
10215	1.9805655	2.8731742	EIF4A1	0.689330113
10216	1.2672943	1.8384432	ZBTB49	0.689330113
10217	1.0992501	1.5946643	LOC202781	0.689330113
10218	1.0742242	1.5583596	KRTAP2-1	0.689330112
10219	1.5174721	2.2013721	GDPD1	0.689330112
10220	1.1545679	1.6749129	RNASE4	0.689330112
10221	0.2510837	0.3642431	TLR8-AS1	0.689330112
10222	0.4214348	0.6113686	ZNF14	0.689330112
10223	0.3213562	0.4661862	LOC100506804	0.689330112
10224	1.23496	1.7915364	TMEM81	0.689330112
10225	0.1747585	0.2535193	AGAP8	0.689330112
10226	22.339916	32.408153	ZNF593	0.689330112
10227	4.3231606	6.2715389	FGF5	0.689330112
10228	6.414327	9.30516	FNDC3A	0.689330112
10229	2.1374308	3.1007361	RPP21	0.689330112
10230	0.2676077	0.3882142	SLC25A5-AS1	0.689330112
10231	0.6237172	0.9048164	LOC100527964	0.689330112
10232	4.6011737	6.6748479	BCL7A	0.689330112

10233	1.3097734	1.900067	ZNF627	0.689330112
10234	28.915978	41.94794	C21orf59	0.689330112
10235	1.6184274	2.3478263	BBS7	0.689330112
10236	3.6142583	5.2431459	ITSN2	0.689330112
10237	2.0336168	2.9501348	SCAND2P	0.689330112
10238	0.1471228	0.2134287	SIRPB2	0.689330112
10239	4.2675666	6.1908895	TMEM186	0.689330112
10240	0.5397552	0.7830142	ZNF431	0.689330112
10241	4.7628835	6.9094377	RNGTT	0.689330112
10242	1.4820984	2.1500561	KDM8	0.689330112
10243	2.8436579	4.1252483	TARBP1	0.689330112
10244	0.8948986	1.2982148	LURAP1	0.689330112
10245	2.998554	4.3499535	SNORA8	0.689330112
10246	0.1313166	0.1904989	ABCA10	0.689330112
10247	1.6694219	2.4218032	SLC9A5	0.689330112
10248	2.8353673	4.1132214	SNORA12	0.689330112
10249	1.0767218	1.5619828	FAM161A	0.689330112
10250	0.339828	0.4929829	GOLGA2P5	0.689330112
10251	0.147383	0.2138061	HSP90AB4P	0.689330112
10252	0.1986649	0.2882	NANOG	0.689330112
10253	1.1746332	1.7040213	LINC00507	0.689330112
10254	0.244744	0.3550461	TDO2	0.689330112
10255	1.2181169	1.7671024	FLJ35946	0.689330112
10256	1.0372435	1.5047123	MCM9	0.689330112
10257	0.311509	0.451901	NNAT	0.689330112
10258	0.7320826	1.0620203	DUSP2	0.689330112
10259	0.261973	0.3800399	LOC100130557	0.689330112
10260	0.8859985	1.2853036	ZNF75D	0.689330112
10261	0.3611776	0.5239545	ZBED3-AS1	0.689330112
10262	0.1809023	0.2624321	LOC158572	0.689330112
10263	0.791641	1.1484208	TMEM9B-AS1	0.689330112
10264	0.2272623	0.3296857	GPR135	0.689330112
10265	0.3238531	0.4698085	C16orf86	0.689330111
10266	0.2083995	0.3023218	FOXE3	0.689330111
10267	0.1258831	0.1826166	RASIP1	0.689330111
10268	0.209763	0.3042997	SULT2A1	0.689330111
10269	0.0569475	0.0826129	LOC440970	0.689330111
10270	0.1981926	0.2875148	C2orf83	0.689330111
10271	0.1067347	0.1548383	WNT4	0.68933011
10272	0.2590423	0.3757884	SULT1A1	0.68933011
10273	0.0553444	0.0802873	IYD	0.68933011
10274	0.0688355	0.0998586	KDR	0.68933011
10275	0.1605543	0.2329135	ZNF385C	0.68933011

10276	0.1665198	0.2415675	C15orf62	0.689330109
10277	0.0599107	0.0869115	MTUS2	0.689330107
10278	1317.9012	1912.617	RPS15	0.689056514
10279	82.169669	119.26343	SNRPA	0.688976247
10280	9.7423155	14.14788	C20orf112	0.688606026
10281	64.829217	94.181237	MRPL20	0.688345355
10282	12.073373	17.543406	PSMD5	0.688200063
10283	15.916174	23.134344	EEFSEC	0.687989003
10284	4.6492943	6.7587955	BCORL1	0.687887999
10285	20.304918	29.519634	CTBP2	0.687844487
10286	1029.9289	1498.7907	RPL8	0.68717326
10287	3.7700021	5.487875	ZKSCAN8	0.686969393
10288	106.27996	154.72729	PLK1	0.68688568
10289	50.637422	73.724081	PSMG1	0.686850508
10290	7.7304297	11.25891	GTF2I	0.686605487
10291	12.57069	18.310877	TEAD1	0.686514878
10292	3.9458531	5.7477413	DCUN1D4	0.686504989
10293	37.196793	54.19642	CD320	0.686333025
10294	5.0451783	7.351487	ZNF219	0.686279979
10295	14.434597	21.036981	SLC35G2	0.686153476
10296	2.9463091	4.2942293	NPHP4	0.686108944
10297	43.533988	63.476813	WLS	0.685825044
10298	4.5502074	6.6347629	PLEKHF2	0.685813122
10299	30.041726	43.812735	GCN1L1	0.685684792
10300	9.9449271	14.512819	GLT8D1	0.685251236
10301	15.107278	22.046334	SAAL1	0.685251236
10302	11.853126	17.29935	SNRNP35	0.685177521
10303	21.106726	30.808895	CEP55	0.685085469
10304	5.541384	8.0913369	CRIPT	0.684853943
10305	2.2517743	3.2879628	FAM172A	0.684853943
10306	9.5543155	13.953313	SNX33	0.684734578
10307	19.785702	28.900252	PAFAH1B1	0.684620404
10308	4.1264371	6.0280165	PCGF5	0.684543098
10309	1.5970612	2.3332622	IPMK	0.684475675
10310	62.606711	91.501043	ELOVL5	0.684218554
10311	12.099216	17.683318	GINS4	0.684216388
10312	13.493231	19.732695	SMCR7L	0.683800726
10313	1.3681953	2.0009554	ATAD5	0.683770998
10314	45.345926	66.32806	PSMF1	0.683661279
10315	2.3823893	3.4848942	GCC2	0.683633169
10316	2.9019948	4.244959	RNF8	0.683633169
10317	16.644646	24.352494	USP19	0.683488332
10318	10.198133	14.922354	DGKD	0.683413116

10319	7.7921018	11.405029	PRDM2	0.68321632
10320	2.3316545	3.4129663	ZFP1	0.683175379
10321	97.329437	142.48621	UQCRH	0.68307971
10322	23.760288	34.78909	YME1L1	0.682981019
10323	10.772341	15.77331	CHMP5	0.682947426
10324	8.8011322	12.887303	IDE	0.682930496
10325	12.898611	18.887778	TIMELESS	0.682907782
10326	37.244952	54.551174	EHD4	0.682752535
10327	4.1237425	6.0404677	HELZ	0.682685967
10328	9.6881651	14.19521	SREBF1	0.682495394
10329	7.8061107	11.438584	CHMP2B	0.682436811
10330	4.7211786	6.9181183	S100PBP	0.682436811
10331	6.8248596	10.00072	GPD2	0.682436811
10332	15.079694	22.102326	PKMYT1	0.682267304
10333	5.550272	8.1368926	RAD54L2	0.682111996
10334	105.63088	154.88471	DBN1	0.681996813
10335	1.502415	2.2029648	MBLAC2	0.681996813
10336	1.6786106	2.4621901	AGL	0.681755056
10337	26.990368	39.609245	FADS1	0.681415875
10338	6.6812142	9.8050304	CUL4B	0.681406778
10339	2.9812452	4.375427	UBR3	0.681360978
10340	2.346275	3.4442234	ASB7	0.681220346
10341	45.736532	67.139879	IARS	0.681212603
10342	26.022136	38.215938	SRSF11	0.680923647
10343	93.89428	137.90893	GLRX3	0.680842643
10344	2.7969545	4.1085342	UBR2	0.680767005
10345	51.131476	75.149625	WBP11	0.680395624
10346	219.47956	322.61335	RANBP1	0.680317669
10347	2.3457846	3.4483648	ING3	0.680259979
10348	5.4223154	7.9730862	LATS1	0.680077359
10349	82.68391	121.58238	POLR2L	0.680064923
10350	11.079235	16.293851	C19orf25	0.679964214
10351	9.7152755	14.292296	SOGA2	0.679756083
10352	7.0086539	10.3119	TNKS2	0.679666606
10353	52.789741	77.683525	ABCF1	0.679548729
10354	5.4592124	8.0343675	PSMC3IP	0.679482539
10355	9.03158	13.29185	TFCP2	0.679482539
10356	22.573886	33.222173	TRAPPC2L	0.679482539
10357	32.57661	47.9719	DDX47	0.679076918
10358	4.408451	6.4921662	WDR44	0.679041603
10359	128.38618	189.08722	PRPF8	0.678978603
10360	6.4859893	9.5526136	CNNM3	0.678975368
10361	19.612543	28.891246	NCOA5	0.678840306

10362	10.328131	15.217483	PARD3	0.678701674
10363	11.121627	16.387377	DLD	0.678670368
10364	4.1403874	6.1017324	ALG14	0.678559329
10365	3.08305	4.5435231	ZNF274	0.678559329
10366	2.0770714	3.061002	CTU1	0.678559329
10367	24.01585	35.395195	CCDC47	0.67850592
10368	3.8723678	5.7089238	C9orf91	0.678300831
10369	15.25208	22.48975	COA1	0.678179184
10370	6.8943169	10.166514	HNRPLL	0.678139688
10371	43.111922	63.590223	DHX30	0.677964623
10372	96.162304	141.86081	MRPL21	0.67786376
10373	17.611225	25.980883	DOT1L	0.677853232
10374	8.4621958	12.484037	MAPKBP1	0.677841277
10375	7.997986	11.799202	DNM1L	0.677841277
10376	34.098385	50.327543	ITGB5	0.677529302
10377	10.696778	15.789881	PCK2	0.67744511
10378	1.0271311	1.5161835	CA13	0.67744511
10379	211.83366	312.72255	SNRPB	0.67738532
10380	2.7076213	3.9982948	ZFC3H1	0.677194019
10381	20.738512	30.630041	ATPAF1	0.677064452
10382	22.801079	33.677	ASXL1	0.677051968
10383	4.7629223	7.0351212	ZFP41	0.677020646
10384	9.8159185	14.500596	RIPK1	0.676932089
10385	3.420279	5.0536274	ACER3	0.676796837
10386	20.798353	30.735041	ARMC6	0.676698409
10387	5.3852626	7.9597151	KATNA1	0.67656474
10388	4.2144447	6.2296186	TECPR2	0.676517285
10389	12.246515	18.102294	PRUNE	0.676517285
10390	1803.9033	2666.6853	RPL41	0.676458985
10391	1566.127	2315.6294	EEF1A1	0.676328857
10392	0.7517062	1.1118702	CYP4V2	0.676073764
10393	36.457298	53.925029	PSMD6	0.676073764
10394	6.6627831	9.8576228	DDHD2	0.675901604
10395	17.63158	26.090336	ABI2	0.675789699
10396	85.426155	126.48529	RTFDC1	0.675384127
10397	30.358653	44.958321	COPS7B	0.675262151
10398	8.1942418	12.137136	ABHD10	0.675138022
10399	13.379031	19.816735	EXOSC6	0.675138022
10400	7.4541678	11.043718	VPS33A	0.674969068
10401	18.870821	27.970715	ENOPH1	0.674663514
10402	11.864328	17.585549	ELP3	0.674663514
10403	28.072639	41.613703	MAGEF1	0.674600836
10404	0.9542102	1.4150185	TTC39B	0.674344675

10405	9.7322307	14.432131	HMOX1	0.674344675
10406	2.5031971	3.712044	C1GALT1	0.674344675
10407	28.529022	42.354696	QRICH1	0.673573995
10408	2.1020123	3.1219587	TRHDE	0.67329918
10409	19.980711	29.678184	SRSF10	0.673245743
10410	26.952658	40.035185	LMAN1	0.673224269
10411	6.8804464	10.221453	CASP8	0.673137794
10412	4.063592	6.0387671	YTHDC2	0.672917491
10413	13.532435	20.118858	ADAM10	0.672624408
10414	344.40554	512.17309	RPS27A	0.672439747
10415	18.905207	28.120469	PCCB	0.672293437
10416	53.312448	79.310438	TCOF1	0.672199642
10417	0.9727804	1.4473813	SLC25A35	0.67209686
10418	1.455773	2.1660166	KRT15	0.67209686
10419	3.3654578	5.0073999	EMC6	0.67209686
10420	1.4853035	2.2099545	CRLF1	0.67209686
10421	4.7100026	7.0079224	C2orf43	0.67209686
10422	1.4679997	2.1842086	CAB39L	0.672096859
10423	43.362896	64.525207	HNRNPH3	0.672030322
10424	105.30434	156.75944	HNRNPM	0.671757588
10425	8.1995625	12.210768	CHST10	0.671502609
10426	3.9625827	5.9048748	C1orf21	0.671069712
10427	4.0770909	6.0761699	PABPN1	0.670996865
10428	7.6401023	11.387245	KHNYN	0.670935067
10429	4.9422806	7.3688442	IL31RA	0.670699569
10430	6.7749245	10.101281	TTC27	0.670699569
10431	19.329809	28.820368	CCDC12	0.670699569
10432	0.8734873	1.3023525	TBC1D8B	0.670699569
10433	0.6028915	0.8988995	ZNF518A	0.670699568
10434	7.2634353	10.832376	MKKS	0.6705302
10435	50.986466	76.044582	C11orf48	0.670481242
10436	44.740585	66.742692	RAB12	0.670344325
10437	3.7433834	5.5856216	C2orf44	0.670182054
10438	4.7683477	7.1150035	INTS7	0.670182054
10439	670.60851	1000.6811	RPL27	0.670152041
10440	2.5785986	3.8487313	ZNF445	0.669986665
10441	35.374085	52.804049	PPIH	0.669912363
10442	111.39673	166.30892	CS	0.66981814
10443	9.804222	14.639104	DIP2B	0.669728308
10444	346.28789	517.12934	RPS12	0.669634966
10445	3.753212	5.6076	MYO6	0.669308076
10446	14.88151	22.237427	RNF31	0.669210071
10447	0.5152606	0.7701311	ESYT3	0.669055697

10448	7.911629	11.825068	SECISBP2	0.669055697
10449	7.4775217	11.176232	TRIT1	0.669055697
10450	1.1589457	1.732211	RSBN1L-AS1	0.669055697
10451	7.1739231	10.72246	NOL10	0.669055697
10452	9.2809494	13.871714	PCNXL4	0.669055697
10453	49.0076	73.274274	SMARCA4	0.6688241
10454	27.134193	40.582028	CCNA2	0.668625851
10455	51.5003	77.030098	SRSF5	0.668573738
10456	11.175745	16.717896	SH3BP1	0.6684899
10457	56.138625	83.97827	SUMF2	0.6684899
10458	1014.5228	1517.7579	EEF2	0.668435182
10459	26.790888	40.083872	EIF2AK1	0.668370751
10460	22.316797	33.390289	FUBP3	0.668361896
10461	2.3732646	3.5511224	FAM21C	0.66831395
10462	23.278668	34.842048	PMF1	0.668119955
10463	21.346239	31.959163	DVL2	0.667922345
10464	1.7001012	2.5458675	GLYCTK	0.667788546
10465	18.49788	27.700205	MNAT1	0.667788546
10466	36.344254	54.43341	RAD21	0.667682847
10467	8.889395	13.315862	STK16	0.667579405
10468	3.4653976	5.1914835	ZC4H2	0.667515868
10469	88.077153	131.95898	TOMM22	0.667458588
10470	6.3466648	9.5088725	RAP2C	0.667446617
10471	47.649132	71.402629	TOMM7	0.667330215
10472	10.029513	15.030938	SPIRE1	0.667257966
10473	4.6605931	6.9849224	PTCD3	0.667236198
10474	3.2506551	4.8728617	PHLPP1	0.667093657
10475	1.4162385	2.1229981	SLC35A3	0.667093657
10476	1.3363225	2.0032008	ZNF184	0.667093657
10477	52.099875	78.109156	NDUFB5	0.66701367
10478	8.2468561	12.369362	FOXP1	0.666716342
10479	4.8609401	7.2918903	TANC2	0.666622767
10480	14.018399	21.030151	TARBP2	0.666585718
10481	16.741234	25.123693	RBM14	0.666352442
10482	3.2562422	4.8866666	TRMT61B	0.666352442
10483	6.076333	9.1193652	ABL2	0.666310966
10484	35.357323	53.073277	MAPKAP1	0.666198229
10485	13.600097	20.416535	SNX6	0.666131503
10486	12.37372	18.580192	CMC1	0.66596299
10487	31.049882	46.633332	PSMG2	0.665830222
10488	4.3726681	6.5683004	KCNN4	0.665722917
10489	44.755907	67.232197	PES1	0.66569158
10490	0.4413166	0.6639223	ZNF624	0.66471118

10491	0.8376934	1.2602367	TMEM198	0.66471118
10492	0.7126123	1.072063	THAP7-AS1	0.66471118
10493	31.789754	47.824913	MTRNR2L10	0.66471118
10494	4.2306665	6.3646689	STEAP1	0.66471118
10495	1.0232381	1.5393725	DOK5	0.66471118
10496	0.7786862	1.1714655	SORBS1	0.66471118
10497	0.7433989	1.1183789	FAM122C	0.664711179
10498	9.3988461	14.143168	SEC62	0.664550272
10499	10.191193	15.337349	SLC20A2	0.664469026
10500	39.425286	59.344644	LMNB1	0.664344463
10501	14.733446	22.180121	RDX	0.664263563
10502	30.504507	45.922294	RPA1	0.664263563
10503	2.7108878	4.0816046	ERCC4	0.664172079
10504	28.854357	43.455741	CHAF1A	0.663994123
10505	8.8296605	13.304422	RPP30	0.663663566
10506	177.64895	267.70131	RPL28	0.663608839
10507	4.1753583	6.2946588	SMPD2	0.663317655
10508	3.1519497	4.7517953	ZNF134	0.663317655
10509	7.636104	11.513697	ZCCHC14	0.663219123
10510	3.2858286	4.9552116	C2orf69	0.663105597
10511	31.246428	47.123558	SAR1A	0.663074464
10512	32.629407	49.216065	IMPDH1	0.662982862
10513	2.9720408	4.483951	FANCL	0.662817416
10514	1.1897665	1.7950139	PUS7L	0.662817416
10515	1.221568	1.8429932	GPR173	0.662817415
10516	11.71157	17.672589	SENP3	0.662696904
10517	13.753141	20.756294	MFSD1	0.662600986
10518	4.5423933	6.8558217	C5orf30	0.662560011
10519	0.6154286	0.9299919	NPHP3	0.661756908
10520	8.8133709	13.31814	ING2	0.661756908
10521	2.6393604	3.9884139	ZNF266	0.661756908
10522	1.604616	2.4247816	IRX5	0.661756908
10523	12.592367	19.03373	RSAD1	0.661581655
10524	7.3852581	11.166364	DIS3L2	0.661384297
10525	2.3387008	3.5366486	FAN1	0.66127598
10526	42.093084	63.686123	SNRPD1	0.660945931
10527	21.744815	32.901618	BET1L	0.660904129
10528	77.264143	116.92603	FARSA	0.660795052
10529	6.7245731	10.176751	SRGAP2C	0.660777978
10530	48.274117	73.057731	IL1B	0.66076671
10531	1.3809244	2.0903839	SNX16	0.660608025
10532	2.7626447	4.1819726	XRCC4	0.660608024
10533	2.7875478	4.21967	MLLT3	0.660608024

10534	1.3117648	1.9856931	OFD1	0.660608024
10535	7.657863	11.594364	KCTD3	0.660481495
10536	2.1451104	3.2488777	FBXO33	0.660261975
10537	1.0709121	1.6226017	AGAP2	0.659996916
10538	11.26321	17.067491	URI1	0.659921831
10539	54.170676	82.093706	QARS	0.659863929
10540	13.911184	21.088769	SLC39A9	0.659648939
10541	2.1717452	3.2924307	ETNK1	0.659617607
10542	34.005131	51.557424	RQCD1	0.65955838
10543	10.513528	15.941151	SMOX	0.659521242
10544	10.171468	15.426291	SNUPN	0.659359238
10545	6.3038995	9.5633617	BROX	0.65917192
10546	2.8327224	4.2973955	GOLGB1	0.65917192
10547	23.279459	35.317628	MATR3	0.659145596
10548	19.926207	30.237501	UTP18	0.658989878
10549	5.7839315	8.7779165	USP32	0.65891849
10550	3.8882519	5.9017639	BMP2K	0.65882878
10551	67.84028	102.98445	DNMT1	0.658742953
10552	38.263949	58.133877	JTB	0.658203973
10553	35.444997	53.856228	SNX12	0.658141097
10554	0.6636927	1.0086562	C11orf70	0.657996926
10555	0.3102502	0.4715071	FAM49A	0.657996926
10556	2.3009409	3.4968869	RPL13P5	0.657996925
10557	3.9651984	6.0261656	PRKD1	0.657996925
10558	2.3378149	3.5529268	TRAPPC13	0.657996925
10559	0.813153	1.2358006	FCRLB	0.657996925
10560	9.8139522	14.914891	TMEM167A	0.657996925
10561	1.9639156	2.9846881	MAGEE1	0.657996925
10562	3.9353809	5.98085	PIK3CB	0.657996925
10563	6.7622257	10.276987	WDR11	0.657996925
10564	0.7698135	1.1699347	ABCA11P	0.657996925
10565	0.5518428	0.8386708	LOC441081	0.657996925
10566	0.8293329	1.2603902	SMA5	0.657996925
10567	0.607073	0.9226077	SLC2A11	0.657996925
10568	197.29318	300.02871	PTBP1	0.657580998
10569	5.6324189	8.5675023	SLC33A1	0.657416681
10570	45.454483	69.153534	RBM22	0.657298044
10571	5.2227259	7.9461101	CDKAL1	0.657268247
10572	8.9685626	13.646619	SLC23A2	0.657200319
10573	6.439033	9.7994546	SMAD2	0.65708075
10574	18.164551	27.644739	ILKAP	0.657070821
10575	8.8464645	13.464574	CCDC127	0.657017763
10576	10.608375	16.146253	ZC3HAV1	0.657017763

10577	21.856714	33.268315	WNK1	0.656982906
10578	5.4655527	8.3203337	CYB5D2	0.656891048
10579	6.5797201	10.016904	NR2C2	0.656861665
10580	6.7094031	10.215499	GATAD1	0.656786631
10581	2.4542161	3.7370435	KLHL13	0.656726661
10582	23.011677	35.0518	UBAP1	0.656504869
10583	3.9333796	5.992236	N4BP3	0.656412663
10584	10.367025	15.793934	ADO	0.656392837
10585	17.580857	26.787497	DPH2	0.656308311
10586	5.9478586	9.0625984	CSRP2BP	0.656308311
10587	27.924689	42.550227	DDX3X	0.656275922
10588	61.889569	94.304729	NAP1L1	0.656272163
10589	1.8139896	2.7664753	ZBTB43	0.655704253
10590	12.749146	19.443439	GMDS	0.655704253
10591	27.972747	42.66628	CHERP	0.65561719
10592	40.211985	61.34626	MFN2	0.655492028
10593	75.221772	114.75996	CCT8	0.655470524
10594	2.8656166	4.3721261	MLL3	0.655428631
10595	7.1130633	10.852905	PER1	0.655406386
10596	2.0647242	3.1505718	IFT80	0.65534905
10597	9.1228235	13.92362	RMI2	0.655204859
10598	6.0187581	9.1860707	PTPRG	0.655204859
10599	16.419501	25.0707	ZNF609	0.65492791
10600	98.846707	150.93774	RNPS1	0.654883989
10601	10.502893	16.03829	ADORA2B	0.654863607
10602	1.5762701	2.4070205	LINS	0.654863607
10603	9.8315704	15.018844	FMNL3	0.654615646
10604	25.026141	38.230282	KAT7	0.654615646
10605	1202.8782	1837.6598	RPL18A	0.654570658
10606	9.447444	14.434665	CCNL1	0.654496942
10607	5.6815567	8.681723	HSDL1	0.654427322
10608	19.001131	29.046602	MPV17L2	0.654160209
10609	9.187558	14.044813	TNRC6A	0.654160209
10610	3.0985058	4.7366161	LOH12CR1	0.654160209
10611	24.192313	36.989434	C3orf37	0.654033088
10612	22.758136	34.796612	CDK1	0.654033088
10613	18.380886	28.106196	DYNC1LI1	0.65397985
10614	32.504878	49.709458	MAP2K1	0.653897256
10615	9.87045	15.097471	COG2	0.653781698
10616	16.990823	25.991963	TOB2	0.653695251
10617	3.4461733	5.2719972	KLHL7	0.653675106
10618	10.131744	15.500432	RB1	0.653642722
10619	47.465948	72.619088	SLC43A3	0.653629085

10620	6.4529794	9.8727753	TMEM57	0.653613526
10621	67.083744	102.63518	MRPL23	0.653613526
10622	2.5226406	3.8608884	KAT6B	0.65338345
10623	15.433215	23.624294	KNSTRN	0.653277282
10624	7.4541947	11.411592	PHF20	0.653212516
10625	1.7711006	2.7120461	DYNC2LI1	0.65304958
10626	1.8008465	2.7598283	MAP3K1	0.652521223
10627	19.755673	30.280065	RERE	0.652431663
10628	15.062189	23.102322	MPI	0.651977249
10629	1.5592326	2.3919507	LRRCC1	0.651866519
10630	67.81873	104.04499	NCAPD2	0.651821218
10631	14.166271	21.756972	DTL	0.651114112
10632	38.725013	59.475137	ACIN1	0.651112632
10633	0.8326185	1.278917	ZNF780A	0.651033995
10634	5.6395917	8.6625149	YIPF6	0.651033995
10635	1.7040844	2.6175045	RPL32P3	0.651033995
10636	24.058113	36.965547	BTBD6	0.650825297
10637	6.6452374	10.212384	CERS6	0.650703856
10638	24.094161	37.031818	PICALM	0.650634036
10639	5.3780516	8.2662481	POC5	0.650603701
10640	573.45959	881.59728	RPL7	0.650477953
10641	2.8537853	4.3876287	ZCCHC6	0.650416316
10642	15.913031	24.465916	EDC3	0.650416316
10643	116.79077	179.59908	KPNA2	0.650286024
10644	5.8139524	8.940608	PHF17	0.650286024
10645	18.332276	28.192184	PKP4	0.650260937
10646	4.9862089	7.6691599	IRAK2	0.650163629
10647	117.24982	180.38338	U2AF1	0.650003421
10648	122.40603	188.33001	TUFM	0.649954955
10649	17.18222	26.436632	MBNL1	0.64993982
10650	2.1035967	3.2366023	NAA16	0.64993982
10651	4.4657036	6.8709493	TBCCD1	0.64993982
10652	13.229979	20.355698	PATZ1	0.64993982
10653	11.303179	17.393128	MRPL30	0.649864648
10654	10.254476	15.786136	RAB3B	0.649587422
10655	17.149959	26.402381	IGF2BP3	0.649561067
10656	1.4513249	2.2353772	DACT1	0.64925278
10657	38.531647	59.357174	DHFR	0.649148953
10658	1.7215865	2.652688	ZKSCAN2	0.648996968
10659	3.5462824	5.4660676	NLRX1	0.648781282
10660	2.9744799	4.5847191	CAMK1D	0.648781282
10661	121.97295	188.06639	HAX1	0.648563278
10662	12.90324	19.895786	RWDD2B	0.648541348

10663	29.940995	46.182921	GTF3C5	0.648313134
10664	8.5811559	13.238927	PLGRKT	0.648176076
10665	12.69783	19.592326	IDI1	0.648102235
10666	4.4340319	6.8429554	NAGPA	0.647970306
10667	4.2660176	6.5836623	C3orf52	0.647970306
10668	10.203536	15.74904	NUDT15	0.647883049
10669	5.4871145	8.4692978	ELF1	0.647883049
10670	4.3249983	6.6784254	LINC00094	0.6476075
10671	8.3584565	12.90667	SYS1	0.6476075
10672	109.30194	168.8137	MCM7	0.647470832
10673	3.0202826	4.6659863	SOS2	0.647297788
10674	46.851409	72.402417	HSPA4	0.647097305
10675	2.7661927	4.2766275	USP31	0.646816361
10676	85.266244	131.84947	HK1	0.646693866
10677	0.4712788	0.729255	H1FX-AS1	0.646246981
10678	0.1518615	0.2349898	DNAH17	0.646246981
10679	0.3332259	0.5156325	CYP1A2	0.646246981
10680	0.1525395	0.2360391	PECAM1	0.646246981
10681	0.7196115	1.113524	PDIK1L	0.646246981
10682	1.7124035	2.6497663	IGFLR1	0.646246981
10683	15.092299	23.353764	HAUS4	0.64624698
10684	16.970643	26.260306	NUP37	0.64624698
10685	0.7367411	1.1400302	ZSCAN20	0.64624698
10686	1.2053181	1.8651044	RFX8	0.64624698
10687	2.2865866	3.5382549	ZNF561	0.64624698
10688	2.2123089	3.423318	FLVCR1-AS1	0.64624698
10689	46.348049	71.718786	KDM1A	0.64624698
10690	3.8674351	5.9844536	AP4M1	0.64624698
10691	4.253051	6.5811542	ULBP3	0.64624698
10692	14.071169	21.773671	PHLDB1	0.64624698
10693	1.2806196	1.9816257	SMIM8	0.64624698
10694	4.9089078	7.5960244	SNX21	0.64624698
10695	10.812645	16.731443	MEMO1	0.64624698
10696	1.2434338	1.9240845	TTC32	0.64624698
10697	2.7776019	4.2980501	PWWP2B	0.64624698
10698	2.5901927	4.0080538	GLIS1	0.64624698
10699	2.7220415	4.2120762	MAFG-AS1	0.64624698
10700	1.8690538	2.8921664	LIN9	0.64624698
10701	7.1272059	11.02861	MIF4GD	0.64624698
10702	0.7589202	1.1743502	CIRBP-AS1	0.64624698
10703	0.2417628	0.3741027	FSD1L	0.646246979
10704	0.1698724	0.2628599	MYPN	0.646246977
10705	18.982654	29.386891	HEG1	0.645956532

10706	13.448656	20.831546	ZNF330	0.645590892
10707	12.150069	18.821998	SP1	0.645524916
10708	5.3376903	8.2706222	BMPR1A	0.645379535
10709	20.365571	31.561064	RAB5A	0.64527518
10710	22.560945	34.966663	GSTM4	0.645212985
10711	11.63883	18.039211	OGT	0.645196172
10712	3.0000424	4.6522586	CLSTN3	0.644857202
10713	6.2435478	9.6830005	WDR48	0.64479474
10714	61.993209	96.189983	MANBAL	0.644487155
10715	10.710344	16.619181	PDDC1	0.644456822
10716	36.924216	57.304652	PRPS1	0.644349355
10717	38.650534	59.984478	TIMMDC1	0.644342252
10718	23.080075	35.82656	MCMBP	0.644216885
10719	8.7302032	13.555255	PUM2	0.644045652
10720	6.4186339	9.9663389	CHUK	0.644031276
10721	2.3363966	3.6290253	AASS	0.643808313
10722	5.0306171	7.81626	FOXD2-AS1	0.643609237
10723	14.093887	21.900716	SIAH2	0.643535455
10724	10.979129	17.061382	ZC3H4	0.64350759
10725	2.2431225	3.4858321	MAP3K2	0.643496993
10726	2.060447	3.2025611	FBXO36	0.643374772
10727	85.018702	132.15924	ATP5D	0.643305037
10728	1.5814714	2.4585979	VPS13B	0.64324118
10729	5.4333779	8.4519044	IREB2	0.642858419
10730	2.4669009	3.8380187	MUT	0.642753753
10731	0.4358237	0.678057	RORA	0.642753753
10732	17.334042	26.973883	PSMC6	0.642623166
10733	28.850809	44.898572	PARL	0.642577439
10734	9.2046708	14.326728	ADM	0.642482435
10735	7.8226205	12.175618	KDELR3	0.642482435
10736	1.5067382	2.3466604	SPEG	0.642077645
10737	27.5354	42.888485	NDUFAF2	0.642023144
10738	521.73302	812.64712	YBX1	0.642016701
10739	124.302	193.62862	SSRP1	0.641960892
10740	1.7342538	2.7022134	MTERF	0.641790105
10741	1.306125	2.035128	OTX1	0.641790105
10742	2.4966329	3.8901082	ICAM5	0.641790105
10743	17.042015	26.572563	TERF2	0.641338775
10744	20.692149	32.263992	KNOP1	0.641338775
10745	9.0528152	14.115496	TP53RK	0.641338775
10746	15.592733	24.314515	ECD	0.64129317
10747	22.105678	34.482095	POLR2D	0.641077005
10748	4.3988092	6.8615926	CRNKL1	0.641077004

10749	25.960676	40.501159	ITPR3	0.640986004
10750	10.109788	15.777174	YAE1D1	0.640785738
10751	17.766953	27.7285	CHKA	0.640747006
10752	505.14792	788.59993	RPS28	0.640562972
10753	80.640314	125.93316	CPNE1	0.640342186
10754	3.5930948	5.6134016	PEX13	0.640092247
10755	1.0763582	1.6815673	LOC728730	0.640092247
10756	111.28655	173.87027	SFPQ	0.640055101
10757	12.544518	19.607411	PMEPA1	0.639784511
10758	14.647425	22.896789	FAM115A	0.639715235
10759	15.349396	23.99495	SLC16A2	0.639692751
10760	8.3644547	13.080817	DUS2L	0.639444381
10761	17.712864	27.704898	MPLKIP	0.639340524
10762	16.400752	25.653155	GAK	0.639326892
10763	4.985634	7.8051574	TICRR	0.638761494
10764	1.368477	2.1427851	ACPL2	0.638644075
10765	2.5097946	3.9338658	BEND7	0.637997019
10766	35.414813	55.522611	POFUT1	0.637844876
10767	2.9119266	4.5667941	HOXA10	0.637630354
10768	2.0784525	3.2608583	POLK	0.637394282
10769	22.434878	35.202069	FADS2	0.637317022
10770	3.8231822	5.9996936	SHISA2	0.63722958
10771	10.848193	17.025731	LETMD1	0.63716459
10772	6.3046199	9.9011848	ADAT1	0.636754087
10773	70.796912	111.20515	CCT2	0.636633389
10774	9.6555753	15.174452	SCD5	0.636304719
10775	9.5087224	14.943662	GPRIN1	0.636304719
10776	1.548	2.4327967	TM4SF19	0.636304719
10777	4.228985	6.6461632	CYB561D1	0.636304719
10778	1.8082386	2.8417809	TAPT1	0.636304719
10779	2.3564608	3.7033527	GUSBP1	0.636304719
10780	1.2731546	2.0008568	LOC100130744	0.636304719
10781	10.997335	17.283127	FAM89A	0.636304719
10782	23.174574	36.441312	PNRC2	0.635942359
10783	14.043277	22.086532	GORASP1	0.635829865
10784	5.8913876	9.2668148	CHD2	0.63575109
10785	1.9669273	3.0940368	CNOT6L	0.635715548
10786	3.3698001	5.3019706	ZNF621	0.635575012
10787	13.259987	20.862978	FRG1	0.635575012
10788	2.5134036	3.9551499	ZNF322	0.635476197
10789	28.676562	45.127113	FERMT2	0.635461931
10790	12.395991	19.507325	CLCC1	0.635453133
10791	32.180208	50.645356	ALDH1A3	0.635402927

10792	36.467762	57.394971	OGFR	0.635382538
10793	25.013378	39.376112	DNAJA2	0.635242441
10794	19.45062	30.622774	CRK	0.635168461
10795	7.4209244	11.683396	AGAP1	0.635168461
10796	26.033585	40.992782	SNRPE	0.635077279
10797	16.452592	25.907466	EBPL	0.635052151
10798	23.915775	37.668927	SPATS2L	0.634893993
10799	14.004446	22.062771	MLLT6	0.634754647
10800	7.9586796	12.543289	ZNF414	0.634497035
10801	62.122335	97.911028	MCM4	0.634477403
10802	26.284622	41.432031	HEBP1	0.63440341
10803	4.2243142	6.6631407	MTF2	0.633982439
10804	3.3492196	5.2832015	NHLRC2	0.633937514
10805	97.564514	153.9074	VDAC2	0.633916985
10806	2.4639642	3.8879796	TMEM116	0.633738974
10807	29.64431	46.77936	HCFC1	0.633704904
10808	9.1966474	14.516144	KANSL2	0.633546173
10809	6.0471796	9.5449706	C17orf80	0.633546173
10810	14.046184	22.17858	PDCD10	0.633322041
10811	454.32105	717.43187	RPL38	0.633260199
10812	17.602557	27.797651	MAP2K7	0.633239011
10813	16.052651	25.354241	EXOSC2	0.633134723
10814	1.9141824	3.0243175	RC3H1	0.632930376
10815	22.693811	35.858206	HINT2	0.632876353
10816	9.4105139	14.878072	LPIN2	0.632508975
10817	2.9697282	4.6956409	TRMT1L	0.632443647
10818	59.681573	94.381548	EMG1	0.632343659
10819	2.9149181	4.6100297	RAB11FIP2	0.632299204
10820	211.94673	335.34131	ADRM1	0.632032852
10821	5.4629598	8.6454841	KRR1	0.631885936
10822	5.4645876	8.6480602	PSIMCT-1	0.631885936
10823	1.9143169	3.0295292	TTC4	0.631885936
10824	3.6766552	5.8185425	SDR39U1	0.631885936
10825	1.2122657	1.9184882	GKAP1	0.631885936
10826	841.63715	1332.4884	RPL12	0.631628108
10827	17.366625	27.511899	GMPR2	0.631240496
10828	11.701782	18.539126	VPS26A	0.631193838
10829	28.523366	45.195578	HMGN4	0.631109664
10830	8.387596	13.290926	WASF1	0.631076864
10831	18.909744	29.974172	ATAD3A	0.630867938
10832	3.4276815	5.4338462	TTC28	0.630802084
10833	14.012789	22.219114	CTHRC1	0.63066372
10834	2.9725449	4.7136608	RRP15	0.630623427

10835	7.2342215	11.472322	ME2	0.630580387
10836	9.5693648	15.187279	PPIL4	0.630090806
10837	13.542582	21.509855	TYRO3	0.629598932
10838	0.01	0.0158837	XIRP2	0.62957754
10839	0.5565801	0.8843191	ZNF529	0.629388364
10840	1.877473	2.9830119	DNASE1	0.629388364
10841	10.510061	16.698849	BNIP1	0.629388363
10842	2.4641833	3.9152031	TASP1	0.629388363
10843	1.7425401	2.7686246	C18orf54	0.629388363
10844	87.237	138.66179	MRPL12	0.629135088
10845	9.1492463	14.544988	TRAPPC10	0.629030858
10846	7.4689212	11.878417	ZEB1	0.628780846
10847	134.07635	213.2639	SRM	0.628687485
10848	19.507472	31.031152	PTX3	0.628641581
10849	2.8573129	4.546192	FRMD5	0.628506867
10850	4.2100909	6.7012963	GZF1	0.628250229
10851	13.811814	21.987038	C1orf174	0.62817986
10852	0.8071979	1.2857917	HTR7P1	0.627782781
10853	37.483215	59.724305	EIF2S3	0.627604037
10854	4.765119	7.5967481	DNAJC3	0.627257732
10855	2.31555	3.6920176	ZNF341	0.627177397
10856	9.1982259	14.667903	RNF149	0.627098922
10857	10.716386	17.08967	DPH5	0.627068038
10858	2.1295754	3.3964678	EPB41L4B	0.62699707
10859	8.9911299	14.344845	GBA2	0.626784716
10860	25.577774	40.810569	MLST8	0.626743874
10861	2.254186	3.5971222	UBE3D	0.626663738
10862	27.577341	44.017443	ANAPC13	0.626509388
10863	12.534542	20.00695	TOP1MT	0.626509388
10864	3.4649555	5.5364951	TBKBP1	0.625839181
10865	19.651984	31.411641	VPS35	0.625627427
10866	0.8825908	1.4110091	SCN8A	0.62550325
10867	9.1218636	14.590164	MEX3C	0.625206381
10868	74.40407	119.05155	UQCRFS1	0.624973566
10869	4.0530313	6.4854505	BBS4	0.624942135
10870	78.985891	126.39441	HNRNPF	0.624915999
10871	2.1794768	3.4879421	USP37	0.624860389
10872	135.32747	216.65812	NHP2	0.624612961
10873	9.8647461	15.79643	GTF3C4	0.624492131
10874	7.0059904	11.218701	ACTR8	0.624492131
10875	8.1366325	13.033231	ZNF317	0.62429897
10876	4.4144061	7.0729722	INTS4	0.62412321
10877	10.315026	16.529823	PPP1R2	0.624025154

10878	3.2679133	5.2373545	ZNRF3	0.623962602
10879	35.350481	56.654807	UPP1	0.623962602
10880	8.5346035	13.686645	CEP170B	0.623571648
10881	2.0355877	3.2650991	RAPGEF6	0.623438263
10882	30.756892	49.344473	DNAJC9	0.623309764
10883	491.40893	788.45939	RPL6	0.623252042
10884	25.217291	40.493631	DDIT4	0.62274709
10885	11.6948	18.781602	ITPRIP	0.622673191
10886	20.410875	32.794031	VAPA	0.622396037
10887	25.754446	41.392513	TIMM17A	0.622200581
10888	29.161705	46.873878	ISG20L2	0.622131265
10889	7.3668323	11.851764	FXR1	0.621581065
10890	11.665892	18.773825	PET112	0.621391327
10891	20.053279	32.279319	TNPO3	0.621242329
10892	23.795657	38.311565	MCM3AP	0.621108974
10893	3.2393044	5.217229	VPS13D	0.620885988
10894	39.489068	63.629479	PPP2R5D	0.620609639
10895	0.1237772	0.1995128	ABCC2	0.620397103
10896	0.3385857	0.5457564	TINCR	0.620397102
10897	0.9041193	1.4573236	DUSP15	0.620397102
10898	1.2380168	1.9955232	SUSD4	0.620397101
10899	0.3786787	0.6103811	LOC150381	0.620397101
10900	0.7017943	1.1312018	SETDB2	0.620397101
10901	1.28027	2.0636298	NSUN3	0.620397101
10902	0.5762198	0.9287919	RNF39	0.620397101
10903	5.2933081	8.532129	SAMHD1	0.620397101
10904	12.233807	19.719316	ZNF496	0.620397101
10905	0.8918666	1.4375738	LOC100505622	0.620397101
10906	7.4396762	11.991797	AVL9	0.620397101
10907	13.857072	22.335811	CDK7	0.620397101
10908	3.0587011	4.9302311	ERCC8	0.620397101
10909	2.1200356	3.4172236	BRAF	0.620397101
10910	2.5606319	4.127408	LOC401321	0.620397101
10911	5.178602	8.3472376	C9orf85	0.620397101
10912	0.773282	1.2464307	ZNF649	0.620397101
10913	2.8744759	4.6332838	BARD1	0.620397101
10914	8.2597363	13.313628	ORC5	0.620397101
10915	1.312064	2.1148777	C15orf48	0.620397101
10916	0.2765142	0.4457051	LOC153684	0.620397101
10917	0.3639107	0.586577	PPM1N	0.6203971
10918	0.1960484	0.3160048	LOC100131096	0.6203971
10919	0.3020283	0.4868305	LOC442132	0.6203971
10920	0.3073739	0.495447	ZNRF2P1	0.6203971

10921	0.4300838	0.6932396	RAPGEF4	0.6203971
10922	0.2477996	0.399421	L1CAM	0.6203971
10923	42.784975	68.99538	HNRPDL	0.620113621
10924	9.7483585	15.731366	BRF1	0.619676547
10925	167.68031	270.66018	RPS24	0.619523379
10926	380.49715	614.17914	YWHAE	0.619521452
10927	35.723803	57.664563	ARHGEF1	0.619510503
10928	79.359562	128.12536	MANF	0.619389963
10929	28.265679	45.637846	IDH2	0.61934736
10930	36.967131	59.690778	PGRMC1	0.619310591
10931	16.612629	26.825229	CENPH	0.619291224
10932	3.3434762	5.3994202	TBC1D24	0.619228745
10933	58.308038	94.181237	SF3A1	0.619104607
10934	8.2656342	13.351482	SLC25A29	0.61907991
10935	5.7323062	9.2640523	MTFMT	0.618768762
10936	22.252944	35.978898	CDC6	0.618499862
10937	9.1239117	14.751818	TRIM14	0.618494042
10938	5.0406531	8.150272	SLFN11	0.6184644
10939	3.1658535	5.1215031	NARG2	0.618149286
10940	3.5644905	5.7666799	PHC3	0.618118324
10941	13.461384	21.790838	SMIM15	0.617754302
10942	5.4669957	8.8504045	FAM160A2	0.617711399
10943	4.6495846	7.5289084	SNTB2	0.617564238
10944	4.7984892	7.7705194	CDCA2	0.617524892
10945	1.6881865	2.7342525	ZMYM2	0.617421575
10946	10.153874	16.457032	CDCA7L	0.616993002
10947	8.5998387	13.94059	CBX2	0.616892033
10948	3.9903734	6.470655	CCDC93	0.616687701
10949	18.839687	30.554594	WAC-AS1	0.616590984
10950	0.7426735	1.2056445	NYNRIN	0.615997122
10951	48.07005	78.039475	AATF	0.615970962
10952	3.8630756	6.2733965	LRP6	0.615786932
10953	33.135521	53.813275	HCFC1R1	0.615749932
10954	0.01	0.0162434	DNAH7	0.615635454
10955	7.2758807	11.829761	RNF111	0.61504885
10956	13.997345	22.758445	LARP4B	0.615039613
10957	24.463624	39.777235	TSTA3	0.615015714
10958	1.5706711	2.5547346	TXNRD3	0.614807938
10959	473.24374	770.04665	EIF5A	0.614565025
10960	46.806305	76.164249	MIF	0.614544298
10961	23.763949	38.670807	TIMM44	0.614519092
10962	12.043813	19.600508	SCAF8	0.614464342
10963	33.9255	55.24152	PLS3	0.614130464

10964	71.544172	116.49937	DNAJA1	0.614116365
10965	30.416123	49.532945	MRPS26	0.614058433
10966	13.699406	22.318263	MTDH	0.613820453
10967	15.90468	25.915958	STX18	0.613702168
10968	18.937548	30.859401	RBM38	0.613671929
10969	27.047848	44.077788	SELT	0.613638963
10970	7.6537563	12.473943	FMR1	0.613579551
10971	15.976625	26.043791	JMJD8	0.613452357
10972	1.5154753	2.4706676	NUDT4	0.613386965
10973	2.8609463	4.6641785	EXOC8	0.613386964
10974	4.2816065	6.9825896	SBF2	0.613183181
10975	1.3738739	2.2421887	LOC283070	0.612737878
10976	1.7281119	2.8203119	ROBO4	0.612737878
10977	13.609763	22.211395	TMEM256	0.612737878
10978	1.548	2.5263658	MAP6D1	0.612737878
10979	1.2726687	2.0770198	C12orf66	0.612737877
10980	8.4481824	13.787596	WDR33	0.612737877
10981	0.7504821	1.2248012	TRPV1	0.612737877
10982	0.3914065	0.6387829	PGM2L1	0.612737877
10983	13.070831	21.33462	BAHD1	0.61265826
10984	7.099379	11.589366	TAF5L	0.61257697
10985	66.211414	108.10749	NOC2L	0.612459084
10986	10.576464	17.274039	NUP43	0.612275085
10987	6.0360293	9.8590236	SNAPC4	0.612233981
10988	3.4823533	5.6879452	SLC38A9	0.612233981
10989	8.2093046	13.410752	DNAJC10	0.612143481
10990	1.7917538	2.9279112	PGBD5	0.611956324
10991	1.195093	1.9529058	LCORL	0.611956324
10992	27.288099	44.61101	MAX	0.611689773
10993	2.1405671	3.499608	SRD5A3	0.611659114
10994	58.939026	96.364392	PHLDA2	0.611626606
10995	8.4555726	13.825071	ANKRD27	0.611611521
10996	7.5074325	12.277026	PSEN1	0.611502519
10997	14.037933	22.95646	DPP7	0.611502519
10998	28.463876	46.558703	MFF	0.611354558
10999	16.753036	27.403786	RTF1	0.611340209
11000	11.730009	19.189984	DRAM2	0.611256812
11001	29.098506	47.610219	CHP1	0.611181941
11002	22.846215	37.383875	CAB39	0.611124862
11003	0.6550274	1.072063	ZNF549	0.610997145
11004	1.4139841	2.3142237	HSF2BP	0.610997145
11005	5.1092758	8.3621926	WDR25	0.610997145
11006	8.76289	14.341949	WDR73	0.610997145

11007	0.8516798	1.3939178	ACOT11	0.610997145
11008	2.0377537	3.335128	PIF1	0.610997145
11009	0.7038694	1.1520012	ZNF460	0.610997145
11010	0.4903518	0.8025435	STARD4-AS1	0.610997145
11011	2.5663673	4.2041898	LARP1B	0.610430882
11012	10.356824	16.978879	C16orf59	0.609982761
11013	8.2834508	13.579811	PPT2	0.609982761
11014	6.6999227	10.984543	PIGG	0.60994097
11015	6.5810368	10.792265	RBM27	0.609792023
11016	0.7860263	1.2890072	ZNF490	0.609792023
11017	11.357023	18.632301	NEK9	0.609534117
11018	25.139707	41.248561	ITPA	0.609468697
11019	32.335348	53.0574	NARS	0.609440861
11020	2.4266703	3.9825968	TMEM180	0.609318581
11021	24.298152	39.880537	UTP11L	0.609273437
11022	343.93204	564.89774	RPL15	0.60883947
11023	2.8535467	4.6871592	COG3	0.608800894
11024	28.46043	46.753441	AEN	0.608734432
11025	0.5084155	0.8358901	GPR153	0.608232453
11026	0.8362741	1.3749251	RRN3P3	0.608232452
11027	1.7152222	2.820011	TSPY26P	0.608232452
11028	63.711879	104.74923	PSMA3	0.608232452
11029	2.0258249	3.3306754	PARP8	0.608232452
11030	9.3410802	15.357747	ZNF335	0.608232452
11031	0.4955993	0.8148189	KIAA1161	0.608232452
11032	45.505179	74.884205	ENSA	0.607673928
11033	2.3471787	3.8638456	SNRNP48	0.607472161
11034	38.249155	62.970466	NUP188	0.607414202
11035	6.9184832	11.390058	ALG1	0.607414202
11036	3.7614005	6.1928109	ZBTB5	0.607381777
11037	5.5274419	9.1021608	CD58	0.607267004
11038	60.396033	99.490179	C14orf2	0.607055228
11039	12.168063	20.055337	TRAPPC4	0.606724438
11040	3.9551897	6.5189226	HOMEZ	0.606724438
11041	31.467978	51.896055	MRPL4	0.606365503
11042	5.6958063	9.3969254	AP5M1	0.606135099
11043	21.15731	34.912793	AGPAT2	0.606004494
11044	26.961159	44.497117	GATAD2A	0.605907997
11045	14.856824	24.526068	BTF3L4	0.605756462
11046	5.1087941	8.4374169	NSUN4	0.605492666
11047	72.260771	119.3435	ACLY	0.605485596
11048	1.4665263	2.4229473	CASP8AP2	0.605265465
11049	10.060666	16.625313	GSE1	0.605141435

11050	18.701638	30.920405	NDRG3	0.604831583
11051	113.94715	188.44441	KPNB1	0.604672512
11052	6.5239165	10.790888	PTPN2	0.60457641
11053	505.33532	835.88409	RPL10	0.604551905
11054	6.2224031	10.292784	IKBKAP	0.604540306
11055	5.4092766	8.9485041	LRRC28	0.604489483
11056	17.921507	29.663568	RYK	0.60415884
11057	2.4449335	4.0477893	MALT1	0.60401698
11058	29.389673	48.664331	HMOX2	0.603926382
11059	1046.1448	1732.3756	RPS16	0.603878714
11060	15.393145	25.494838	AAGAB	0.603774957
11061	727.29084	1204.7579	RPL7A	0.603682141
11062	7.0537364	11.684912	NUDT19	0.603661919
11063	4.4946832	7.4500127	BIRC6	0.603312155
11064	0.691209	1.1459721	CCDC136	0.603163849
11065	4.2014754	6.9657282	RAD50	0.603163848
11066	4.827631	8.0038468	MLLT10	0.603163848
11067	4.2814198	7.09827	PPTC7	0.603163848
11068	0.7849322	1.3013582	ZRANB3	0.603163848
11069	0.6334331	1.0501842	MIR210HG	0.603163848
11070	47.938543	79.52447	PTDSS1	0.602814997
11071	5.55732	9.219522	CTDSPL2	0.602777453
11072	61.199511	101.55044	H1F0	0.602651389
11073	7.6312656	12.665077	ZFAND1	0.602543947
11074	19.03925	31.613174	SSBP4	0.602256835
11075	2.932866	4.8701153	LRRC8E	0.602216966
11076	39.936207	66.366633	TAF9	0.601751287
11077	28.223039	46.922309	FAM192A	0.601484446
11078	69.105448	114.91579	STRAP	0.601357263
11079	4.919044	8.1802667	AZI2	0.601330524
11080	102.25648	170.05195	YWHAB	0.601324934
11081	5.4384003	9.0465109	RABGEF1	0.601159982
11082	2.0451374	3.4019853	YAF2	0.601159982
11083	67.196068	111.85842	PRDX3	0.60072426
11084	5.9712191	9.9435371	ANGPT2	0.600512579
11085	4.336637	7.223102	C15orf41	0.600384291
11086	7.2720989	12.112407	SNAPC3	0.600384291
11087	111.20265	185.22818	CYC1	0.600354904
11088	17.100236	28.494567	PIK3CD	0.600122686
11089	19.506458	32.507717	PARVB	0.600056213
11090	5.5020287	9.174372	COX15	0.599717198
11091	4.16799	6.9499258	FZD1	0.599717198
11092	10.82307	18.049799	SPEN	0.599622769

11093	11.581591	19.32559	SIPA1L1	0.599287857
11094	97.188949	162.1955	EIF3E	0.599208675
11095	21.647131	36.134312	DFFA	0.599074124
11096	7.3676591	12.299848	C1orf109	0.599004098
11097	4.9297691	8.2305396	PWWP2A	0.598960616
11098	0.9885272	1.6513191	SDHAP2	0.598628782
11099	7.7092296	12.878147	MOCS1	0.598628782
11100	1.6587514	2.7709183	METTL12	0.598628782
11101	2.3958138	4.0021694	SPOPL	0.598628782
11102	2.8392302	4.7428896	ZFP62	0.598628782
11103	4.7153684	7.8769491	ZNF275	0.598628782
11104	35.463438	59.261938	NDRG1	0.59841846
11105	6.359848	10.6285	DPP8	0.598376834
11106	1.6564057	2.7690802	ZC3H12C	0.598179023
11107	59.290815	99.121892	PSME3	0.598160646
11108	2.9736913	4.9745709	UBE2V1	0.597778457
11109	76.611719	128.2105	CALM1	0.597546379
11110	28.41392	47.554229	NIPSNAP1	0.597505651
11111	2.9479799	4.9345229	CBWD1	0.597419431
11112	7.7999813	13.056123	RSPRY1	0.597419431
11113	3.5591695	5.9608014	SKIL	0.597095802
11114	25.945927	43.455325	DDX1	0.597071284
11115	20.074232	33.624666	NTMT1	0.597009115
11116	4.5753875	7.6679106	ROCK1	0.596692864
11117	2.2413904	3.7573451	ENOX2	0.596535674
11118	2.9165819	4.8891995	FAM102B	0.596535674
11119	3.4839705	5.8403388	ARL5B	0.596535674
11120	1.9314133	3.2377164	PLCD4	0.596535674
11121	33.55731	56.253652	POLE3	0.596535674
11122	4.115314	6.8986888	TYSND1	0.596535674
11123	2.5781381	4.3218507	UEVLD	0.596535674
11124	7.6318176	12.793565	PXK	0.596535674
11125	6.9197399	11.599876	RABEP1	0.596535674
11126	12.348412	20.705377	KDSR	0.596386726
11127	12.753615	21.388216	CGGBP1	0.596291693
11128	1.4567399	2.4447621	EVC2	0.595861622
11129	4.8733422	8.1859433	C17orf58	0.595330552
11130	5.1557168	8.6602591	PHLDB2	0.595330551
11131	8.0484731	13.528098	EIF4E	0.594944912
11132	6.8707522	11.551238	NEURL4	0.594806585
11133	5.50639	9.2593988	SIRPA	0.594681159
11134	19.789196	33.2808	NFYC	0.594612998
11135	17.867653	30.056099	UQCC	0.594476786

11136	19.66033	33.072746	MRPL15	0.594457153
11137	3.305067	5.559807	TRPM7	0.594457153
11138	2.9448632	4.9548154	UVRAG	0.59434368
11139	2.8810991	4.8503578	GABPB2	0.593997224
11140	16.508814	27.799703	DUT	0.593848577
11141	40.554465	68.308581	IMP4	0.593695028
11142	5.9652673	10.049477	TMEM243	0.593589819
11143	1.681516	2.8327912	LGALSL	0.593589819
11144	7.2447409	12.206727	DHX40	0.593503953
11145	1.9427307	3.2745821	GXYLT1	0.593275916
11146	31.562019	53.199562	GNPDA1	0.593275916
11147	0.8209214	1.3847644	NBPF10	0.592823896
11148	74.391683	125.50143	CHCHD3	0.592755646
11149	1.597291	2.6957858	PRDM15	0.592514085
11150	292.73508	494.09556	RPL13A	0.592466536
11151	0.01	0.0168786	ERBB4	0.592464761
11152	10.698475	18.065729	ZMYM3	0.592197233
11153	12.931711	21.838359	ACD	0.592155823
11154	4.2401208	7.1644698	PARG	0.591826182
11155	5.2311951	8.8422167	ATF2	0.591615792
11156	6.5088305	11.00654	FAM91A1	0.591360251
11157	43.110472	72.916197	RRM1	0.591233135
11158	11.823805	20.001807	AP3M1	0.591136817
11159	0.1647427	0.2788211	GRIP1	0.590854383
11160	0.4188935	0.7089623	DLX6-AS1	0.590854383
11161	0.1650036	0.2792627	LOC100271836	0.590854383
11162	0.2572833	0.4354429	FHDC1	0.590854383
11163	0.3939499	0.6667462	MAPK13	0.590854382
11164	0.3845009	0.6507541	CCDC96	0.590854382
11165	6.3451798	10.738991	IFI30	0.590854382
11166	2.0592836	3.4852642	CCDC122	0.590854382
11167	10.461584	17.705858	TMEM9B	0.590854382
11168	3.33606	5.6461628	UBE2D4	0.590854382
11169	3.400458	5.7551541	PVT1	0.590854382
11170	1.5566723	2.6346124	LOC642236	0.590854382
11171	0.3897139	0.6595769	ASB16	0.590854382
11172	1.5908359	2.6924331	LOC645513	0.590854382
11173	0.4149318	0.7022573	TMEM217	0.590854382
11174	0.3251162	0.5502476	KLHL7-AS1	0.590854382
11175	0.5885952	0.9961765	LRRC37A3	0.590854382
11176	0.52726	0.8923687	TPT1-AS1	0.590854381
11177	0.4840871	0.8193002	C9orf117	0.590854381
11178	0.0948672	0.1605593	ITGB8	0.590854381

11179	0.1236609	0.2092916	ZNF578	0.59085438
11180	115.80126	196.06707	EIF3D	0.590620658
11181	14.340877	24.286834	NCOR1	0.590479474
11182	4.9667795	8.416022	LCLAT1	0.59015762
11183	25.135207	42.64814	SEPHS1	0.589362325
11184	20.815746	35.346951	MRPL45	0.58889791
11185	50.442152	85.657835	LSM2	0.588879601
11186	50.198532	85.250159	PRKDC	0.588837991
11187	6.3326882	10.755194	TMEM181	0.588802804
11188	17.535233	29.786467	PRKAG1	0.588697979
11189	4.5048383	7.6537157	USP42	0.588581865
11190	4.8313167	8.2084023	DCUN1D1	0.588581865
11191	4.0287117	6.8462815	ATXN7	0.588452535
11192	26.321931	44.735612	TRIM27	0.588388753
11193	10.054362	17.090315	NAT14	0.588307596
11194	3.4998389	5.948995	NAP1L5	0.588307596
11195	83.469196	141.98279	EIF4A2	0.587882499
11196	26.049938	44.324359	ORAI1	0.58771154
11197	2.8234771	4.8111422	METAP1D	0.586862123
11198	0.4749202	0.8092534	BACH2	0.586862123
11199	14.717649	25.084103	TWF1	0.586732142
11200	26.646713	45.420746	FH	0.586663925
11201	97.835583	166.80901	PTGES3	0.586512582
11202	47.33843	80.752614	NSA2	0.586215443
11203	11.666468	19.90596	RPAP1	0.586079158
11204	13.054911	22.278373	MGEA5	0.58599033
11205	1.5865614	2.7077633	LNP1	0.585930595
11206	6.2154237	10.607781	PITPNA-AS1	0.585930595
11207	34.00458	58.068331	TRA2B	0.585595969
11208	17.253627	29.483778	SPRED2	0.585190517
11209	29.936712	51.18837	XPOT	0.584834245
11210	5.1632844	8.8327742	ZFHX3	0.584559769
11211	10.131422	17.333115	SEC63	0.584512474
11212	9.450675	16.170699	MED11	0.584432052
11213	1.821919	3.1174179	METTL18	0.584432052
11214	1.7456144	2.986856	SERPINF1	0.584432052
11215	0.8839131	1.5124308	ZNF33A	0.584432052
11216	11.314426	19.37107	EAPP	0.584088874
11217	4.4875492	7.6848583	FAM178A	0.583946912
11218	6.3889688	10.944011	AMIGO2	0.583786746
11219	34.280067	58.745656	WDR82	0.583533644
11220	5.6780009	9.7321458	SACM1L	0.583427441
11221	1.0960528	1.8791217	WDR5B	0.583279326

11222	1.6081336	2.7570558	MPP3	0.583279326
11223	33.151041	56.835619	MTRNR2L3	0.583279326
11224	2.9082074	4.9859601	ABHD17B	0.583279326
11225	3.8430754	6.5887393	CCDC77	0.583279326
11226	2.4194137	4.1479504	ATP5S	0.583279326
11227	21.156417	36.274078	HMGB3	0.5832379
11228	23.522201	40.332783	NDUFS1	0.58320303
11229	13.189268	22.618245	ZNF687	0.583125182
11230	14.378212	24.6618	NDUFA10	0.583015518
11231	40.164032	68.915956	CTPS1	0.582797277
11232	4.8170201	8.275231	ARNT2	0.582100984
11233	30.309541	52.084468	SF3B3	0.581930508
11234	0.4249197	0.7305767	ZNF273	0.581622283
11235	3.3779298	5.8077723	THTPA	0.581622282
11236	0.5335976	0.9174297	DZANK1	0.581622282
11237	1.7345975	2.9823436	MN1	0.581622282
11238	19.090903	32.835826	ATP6V1D	0.581404691
11239	18.151818	31.223835	ARL8B	0.581344923
11240	17.527906	30.159779	SKI	0.581168244
11241	35.723721	61.542806	SAFB	0.580469481
11242	7.7413764	13.340946	LHFPL2	0.580271915
11243	4.4111699	7.6024832	EFTUD1	0.580227505
11244	2.943315	5.074278	TMEM106B	0.58004607
11245	7.0163639	12.10331	CNOT6	0.579706186
11246	0.6026402	1.0396372	PDK3	0.579663959
11247	44.522662	76.818638	SFXN1	0.579581502
11248	23.243947	40.109407	TBL3	0.579513607
11249	7.3654709	12.710225	LOC92249	0.579491797
11250	14.12346	24.37281	MACF1	0.579476074
11251	834.78041	1441.1386	RPS8	0.579250616
11252	1.3420391	2.3177075	MTRF1	0.579037295
11253	3.4476727	5.9541461	HS2ST1	0.579037294
11254	2.5070617	4.3297067	CALML4	0.579037294
11255	40.621261	70.15587	PRRC2C	0.579014431
11256	15.558091	26.876681	KIF23	0.578869506
11257	30.832475	53.267185	RSL24D1	0.578826812
11258	8.1508762	14.087498	SMARCA5	0.578589354
11259	26.769281	46.275336	AFG3L2	0.578478378
11260	42.913668	74.186209	OCIAD2	0.578458836
11261	7.0874444	12.254885	PBX3	0.578336281
11262	11.218402	19.402157	TP53INP2	0.578203847
11263	298.27626	515.99019	RPL21	0.578065755
11264	37.342779	64.618394	SRSF7	0.577897049

11265	2.743999	4.7488751	POLR2M	0.577820829
11266	2.9920962	5.1797652	KNTC1	0.577650932
11267	5.2845681	9.150025	ARMC8	0.577546851
11268	6.3110797	10.93097	NUFIP2	0.577357708
11269	25.311323	43.840708	WDR46	0.577347497
11270	14.951477	25.904484	PRPF4	0.577177197
11271	12.115335	20.999323	COIL	0.576939333
11272	10.607649	18.387098	MLF1IP	0.576907176
11273	6.1656657	10.689147	FBRSL1	0.576815487
11274	1.1993621	2.0798746	PSD4	0.576651152
11275	16.931765	29.367787	GPBP1L1	0.576542096
11276	6.5867551	11.426955	GABPB1	0.576422594
11277	433.03039	751.24928	RPL10A	0.576413725
11278	3.5605945	6.1780998	RIOK2	0.576325176
11279	5.0262624	8.7277252	C18orf25	0.575896043
11280	28.082202	48.778867	ZNF598	0.57570427
11281	4.8758982	8.4705984	IRX3	0.575626176
11282	13.582045	23.602854	TXNRD2	0.57544079
11283	2.6866124	4.670332	ATP11C	0.575250833
11284	14.540689	25.283071	HIPK1	0.575115592
11285	51.771219	90.02753	PSMD1	0.575059855
11286	21.508116	37.404035	WDR6	0.575021273
11287	74.967469	130.40759	GLO1	0.574870448
11288	27.083142	47.112872	SLC39A14	0.57485652
11289	51.405848	89.432807	CCNB1	0.574798556
11290	11.258223	19.586711	TSPAN5	0.574788855
11291	0.1880183	0.3273061	ARHGAP28	0.574441761
11292	0.2917943	0.5079615	UCKL1-AS1	0.574441761
11293	0.4258265	0.7412875	SNAI3-AS1	0.574441761
11294	0.3578288	0.6229157	KCNJ5	0.57444176
11295	0.9429842	1.6415662	SMPDL3A	0.57444176
11296	2.7293299	4.7512735	C8orf42	0.57444176
11297	0.8883184	1.5464029	CFD	0.57444176
11298	2.1429255	3.7304486	HYAL3	0.57444176
11299	2.8315149	4.9291593	KCTD6	0.57444176
11300	4.0231564	7.0035931	C1orf50	0.57444176
11301	3.5719797	6.2181755	RNF141	0.57444176
11302	0.9966499	1.7349886	PCED1B	0.57444176
11303	14.091627	24.530993	MLH1	0.57444176
11304	12.847758	22.365641	SURF6	0.57444176
11305	1.0985741	1.9124203	LOC401052	0.57444176
11306	0.1917552	0.3338113	TMEM63C	0.57444176
11307	0.6368205	1.1085901	PPM1E	0.57444176

11308	0.6536998	1.137974	LOC100134368	0.57444176
11309	0.4166323	0.7252821	MGC16275	0.57444176
11310	39.892373	69.571956	PIP5K1A	0.573397321
11311	5.8929816	10.2777	SETD2	0.573375511
11312	8.621785	15.041228	AGFG1	0.573210185
11313	6.0692479	10.590992	AIMP1	0.573057563
11314	6.8365975	11.933542	INO80	0.572889215
11315	12.667675	22.114976	SGK1	0.572809823
11316	34.007962	59.388809	RPL7L1	0.572632495
11317	12.336292	21.54477	CTU2	0.572588722
11318	49.745208	86.933538	EI24	0.572221131
11319	83.588511	146.09191	APLP2	0.572163873
11320	21.416549	37.441695	MYEOV2	0.571997327
11321	7.1060813	12.423277	C2orf47	0.571997327
11322	2291.528	4007.2824	RPS2	0.5718409
11323	5.0142266	8.7687264	SLC48A1	0.571830661
11324	5.3117009	9.2904376	C7orf25	0.571738505
11325	16.871233	29.515545	STX16	0.571605011
11326	1.5691608	2.7460804	USP15	0.571418383
11327	6.6033791	11.556119	ARMC10	0.571418383
11328	0.7876162	1.378353	PGAP1	0.571418382
11329	3.2756561	5.7351013	LOC100294145	0.571159236
11330	5.006139	8.7661393	ZADH2	0.571076829
11331	15.367818	26.933263	TATDN2	0.570588797
11332	6.1069451	10.704922	FANCA	0.570480093
11333	2.3075377	4.0449049	LOC283683	0.570480093
11334	3.2981128	5.7812933	HRSP12	0.570480093
11335	10.849432	19.018072	EIF2AK4	0.570480093
11336	13.778479	24.152428	KLF16	0.570480093
11337	1.021149	1.7899819	TET2	0.570480093
11338	0.3880358	0.6801917	ZNF678	0.570480092
11339	30.997844	54.36373	MTHFD2	0.570193477
11340	11.293736	19.809016	RAP2B	0.570131104
11341	8.6698619	15.208634	TONSL	0.570061853
11342	22.723214	39.862412	MCCC2	0.570041112
11343	21.890704	38.415728	LSM14B	0.569837017
11344	61.415747	107.78138	LARP1	0.569817771
11345	7.26975	12.759489	CHTF18	0.56975244
11346	6.1787553	10.844632	MTMR1	0.56975244
11347	10.546929	18.511424	NTHL1	0.56975244
11348	0.9076926	1.5931351	RPGRIP1L	0.56975244
11349	6.8127577	11.95881	FAR1	0.569685236
11350	5.0356757	8.8411323	DBR1	0.56957361

11351	58.660266	102.99784	HYOU1	0.569529091
11352	11.689367	20.525732	TBC1D1	0.5694982
11353	1.8453321	3.2421363	LOC643837	0.569171652
11354	20.498311	36.014287	XPO7	0.569171652
11355	6.5202614	11.456505	CCDC14	0.569131795
11356	27.346541	48.06141	POLR1D	0.568991649
11357	5.3960937	9.4850527	SH3D19	0.568904972
11358	16.38567	28.803416	VAPB	0.568879384
11359	437.36569	768.91408	NPM1	0.568809571
11360	8.0718116	14.19351	TMEM41A	0.568697343
11361	2.3249437	4.0881916	HSPBAP1	0.568697343
11362	0.4010487	0.7052059	SULF1	0.568697343
11363	28.536031	50.177887	FAM207A	0.568697343
11364	53.320708	93.817396	VPS72	0.568345644
11365	17.632011	31.024697	SPDL1	0.5683218
11366	5.981616	10.528619	NDUFAF1	0.568129214
11367	3.3798167	5.9490282	PIK3C3	0.568129213
11368	25.853062	45.510804	SH2B3	0.568064284
11369	7.1559271	12.600821	TUBGCP4	0.56789372
11370	28.581124	50.34317	TOP2A	0.567725948
11371	108.23618	190.65597	EIF3L	0.567704122
11372	4.4189216	7.7841274	TPRN	0.567683622
11373	10.654257	18.773729	CKAP2	0.567508842
11374	12.168656	21.44497	RABGGTA	0.567436373
11375	5.1786649	9.1282191	RIOK1	0.567324783
11376	4.9203876	8.67428	EEF2K	0.567238729
11377	32.821106	57.866002	HPRT1	0.567191524
11378	3.3486065	5.9042273	TNRC6B	0.567154066
11379	3.6114083	6.3689942	TMEM42	0.567029609
11380	0.5428734	0.9573986	GOLGA8A	0.567029608
11381	10.482435	18.489664	LBR	0.566934851
11382	16.850696	29.729739	PAPD7	0.566795951
11383	41.759727	73.680527	UBQLN1	0.566767489
11384	437.32604	771.81326	ANXA2	0.566621568
11385	23.487117	41.47669	METTL9	0.566272694
11386	3.0944293	5.4666512	KCTD7	0.566055749
11387	21.676711	38.296003	WDR5	0.566030633
11388	3.932066	6.9499258	OXSM	0.565770941
11389	2.4922002	4.4049633	RDH13	0.565770941
11390	1.7067454	3.0166721	TBCK	0.565770941
11391	4.8984328	8.6619826	MCC	0.565509422
11392	476.02542	841.79015	RPS9	0.565491793
11393	53.88975	95.300131	VARS	0.565474039

11394	2.7090614	4.7926723	TTF1	0.565250692
11395	24.264142	42.930349	TRAF7	0.56519787
11396	11.840277	20.950706	OSBPL9	0.56514932
11397	48.924264	86.569599	E2F4	0.565143701
11398	4.4378859	7.8540303	KIAA0226	0.565045687
11399	76.495679	135.41109	GDI2	0.564914434
11400	5.6371067	9.9809379	DLX1	0.564787277
11401	57.73839	102.26964	MCM3	0.564570184
11402	12.631675	22.374317	KIF4A	0.564561362
11403	176.87864	313.32447	HNRNPK	0.564522276
11404	13.386245	23.714973	AP1G1	0.564463864
11405	39.148732	69.358571	GRHPR	0.564439716
11406	520.09893	921.75941	RPL24	0.564245853
11407	0.2346841	0.4161085	ZNF286B	0.563997365
11408	0.4585248	0.8129909	BEND6	0.563997365
11409	1.4218289	2.5209851	CPPED1	0.563997365
11410	8.0325289	14.242139	SMIM4	0.563997365
11411	3.6375185	6.4495311	DTWD1	0.563997365
11412	0.7196115	1.2759129	ZNF37BP	0.563997365
11413	1.5494387	2.7472445	TMEM53	0.563997365
11414	0.4140387	0.7341147	DKFZP434I0714	0.563997365
11415	3.5489035	6.2924115	PER2	0.563997365
11416	2.0124415	3.5681754	FKTN	0.563997364
11417	0.697377	1.2364899	DUSP19	0.563997364
11418	0.3434213	0.6089059	B3GNT7	0.563997364
11419	0.1704467	0.3022119	ADAMTSL3	0.563997363
11420	27.701765	49.132613	IFRD2	0.563816248
11421	32.541346	57.724869	AIMP2	0.563731829
11422	8.6888823	15.416665	TP53BP1	0.563603237
11423	22.148386	39.305718	PGAM5	0.563490173
11424	8.2146276	14.580057	USP48	0.563415324
11425	7.5671337	13.432406	TRAK2	0.563349092
11426	22.792193	40.458382	MRPS10	0.563349092
11427	13.234287	23.508691	CCAR1	0.562952925
11428	6.4271454	11.417932	DOCK5	0.562899239
11429	6.1481845	10.926828	EPS15	0.562668749
11430	25.097507	44.604605	CDC42BPB	0.562666285
11431	4.7922351	8.5171315	ZNF462	0.56265834
11432	13.399025	23.843637	R3HDM1	0.561953896
11433	15.661926	27.872542	TMEM201	0.561912347
11434	22.978199	40.896003	MRPL52	0.561869073
11435	9.6811504	17.247371	ALG5	0.561311663
11436	1.059564	1.8876571	ZNF551	0.561311663

11437	1.6957561	3.0210598	ZNF517	0.561311663
11438	0.8731181	1.5554961	CEP44	0.561311663
11439	72.258698	128.80078	TCP1	0.561011356
11440	7.3859448	13.165738	PIGH	0.560997379
11441	11.684286	20.83193	IVD	0.560883507
11442	75.229062	134.1362	MAPRE1	0.560840863
11443	2.6728593	4.7660613	ZNF791	0.560810939
11444	0.9482133	1.6907896	STEAP2	0.560810939
11445	7.5309477	13.436523	PIN4	0.560483363
11446	43.300579	77.262999	POLR2A	0.560430986
11447	38.049183	67.921536	MRTO4	0.560193205
11448	4.786561	8.5461985	RPL21P28	0.560080716
11449	0.6212322	1.1091833	DPY19L2P2	0.560080716
11450	2.1761804	3.887725	TMF1	0.559756783
11451	68.491843	122.36834	CCT4	0.559718673
11452	50.590092	90.385353	NT5DC2	0.559715605
11453	8.7985314	15.721867	ASNSD1	0.55963656
11454	8.2158585	14.685923	WDR59	0.559437685
11455	19.879896	35.5452	ZC3H15	0.559284986
11456	20.112975	35.973947	F8A1	0.559098365
11457	11.900317	21.285581	NAV1	0.559078783
11458	16.058948	28.736237	MYO1B	0.558839638
11459	12.264961	21.94925	GTF3C2	0.558787227
11460	1.7951244	3.2127924	ZNF292	0.558742731
11461	13.43846	24.060441	NXN	0.558529246
11462	23.204947	41.563864	MAP1S	0.558296208
11463	9.3123676	16.693311	EIF1AX	0.557850255
11464	3.5879397	6.4339162	B3GNT2	0.557660316
11465	1.2435574	2.229955	KIAA1731	0.557660315
11466	2.9287964	5.252509	RAB3GAP2	0.557599489
11467	3.892011	6.9806046	CTBP1-AS1	0.557546414
11468	2.1890704	3.9262568	SNRK	0.557546414
11469	4.36029	7.8211522	TMEM237	0.557499699
11470	3.5189913	6.3129836	UBR1	0.557421264
11471	7.1719219	12.871851	LSM5	0.557178744
11472	15.91329	28.565389	IARS2	0.557082884
11473	11.729367	21.058914	LSG1	0.556978731
11474	0.5777412	1.0376721	LOC284023	0.55676663
11475	0.5289327	0.950008	NPHP1	0.556766629
11476	0.9998605	1.7958341	TSSK6	0.556766629
11477	1.3639986	2.449857	TMEM260	0.556766629
11478	7.8939205	14.178149	LANCL1	0.556766629
11479	3.9576682	7.1083071	PPP2R3C	0.556766629

11480	5.6215665	10.096809	NME3	0.556766629
11481	15.28263	27.448897	UCHL3	0.556766629
11482	0.2205953	0.3962078	SKIDA1	0.556766628
11483	54.277277	97.538904	H2AFX	0.556467988
11484	27.866555	50.079932	RBM39	0.556441542
11485	4.4592205	8.0145896	EXD2	0.556387876
11486	9.1249255	16.406468	GRSF1	0.556178547
11487	4.2247602	7.5971472	ABCD4	0.556098242
11488	3.2357031	5.8185817	GSTCD	0.556098242
11489	8.7547381	15.749626	PRR3	0.555869583
11490	85.041949	153.04381	EIF3M	0.555670632
11491	2.6698213	4.8079445	AP4E1	0.555293702
11492	7.2351086	13.034068	GTF2F2	0.555092143
11493	16.343117	29.456256	UBE2G1	0.554826676
11494	4.8619184	8.7629499	TADA2A	0.554826676
11495	1.4336426	2.5839468	MEIS1	0.554826676
11496	1.406377	2.5348042	LOC100505738	0.554826676
11497	15.709018	28.313378	SMNDC1	0.554826676
11498	12.882878	23.221398	NUP50	0.554784783
11499	67.373651	121.4425	HIST1H2BK	0.554778177
11500	1254.4389	2262.2143	RPLP0	0.554518144
11501	2.4478671	4.4148576	C2CD3	0.554461177
11502	7.0034346	12.631064	SNHG11	0.554461177
11503	14.235741	25.690254	NSMCE4A	0.554130007
11504	5.4275914	9.7967929	TCF4	0.554017164
11505	2.0592836	3.7176151	SFR1	0.553925983
11506	2.1214744	3.8298878	CEP76	0.553925983
11507	7.1099147	12.835496	DDX55	0.553925983
11508	2.6660917	4.8130829	THUMPD2	0.553925983
11509	3.2404898	5.8500412	PRPF39	0.553925983
11510	8.873458	16.019213	ATAD3B	0.553925983
11511	1.6530896	2.984315	LOC100506060	0.553925983
11512	7.035172	12.706571	AMPD3	0.55366408
11513	7.6516616	13.822817	RCOR1	0.553552969
11514	13.031282	23.544853	MFAP1	0.553466294
11515	14.765684	26.679223	NCOA6	0.553452542
11516	6.6169181	11.958419	TTPAL	0.553327144
11517	10.369056	18.743084	WWC1	0.553220345
11518	679.30816	1228.1261	RPL3	0.553125733
11519	39.613128	71.624442	WBSCR22	0.553067183
11520	0.729779	1.3195124	KLHL28	0.553067183
11521	8.2039622	14.834894	CPSF6	0.553017908
11522	8.3862978	15.16678	C9orf64	0.552938593

11523	2.9275168	5.29463	ZXDC	0.552921888
11524	31.267299	56.556066	NOP2	0.55285492
11525	5.5600657	10.057477	SIK3	0.5528291
11526	155.41257	281.16039	PGAM1	0.55275413
11527	34.569354	62.549332	CSK	0.552673441
11528	7.2263809	13.075811	SMEK2	0.55265259
11529	45.668841	82.635713	RAE1	0.55265259
11530	6.7612606	12.234197	ACSL1	0.55265259
11531	18.900061	34.19881	RBM17	0.55265259
11532	11.355735	20.548926	ARIH1	0.552619392
11533	4.35108	7.8744275	ZFYVE26	0.552558265
11534	12.355702	22.36697	GINS1	0.552408378
11535	9.0106214	16.311522	C2orf49	0.552408378
11536	5.9491867	10.769545	C17orf85	0.552408378
11537	20.551214	37.205043	NOL11	0.55237711
11538	12.258794	22.199704	BNIP2	0.552205305
11539	6.2648833	11.345605	HSDL2	0.552185901
11540	9.6995817	17.56701	ELMSAN1	0.552147562
11541	10.597925	19.198675	ULK3	0.552013357
11542	3.9573053	7.1692601	PAQR3	0.551982383
11543	18.281254	33.125751	BAZ1B	0.551874405
11544	9.4468934	17.124328	WDR76	0.551665061
11545	0.2472851	0.4484156	KIAA1024	0.551464091
11546	0.4207966	0.7630534	ITGA9	0.551464091
11547	0.8567297	1.5535548	C12orf76	0.55146409
11548	4.0125054	7.2760956	TMEM80	0.55146409
11549	2.0627232	3.7404488	HMBOX1	0.55146409
11550	4.8421689	8.7805697	TNK2	0.55146409
11551	0.7968119	1.4449025	C10orf118	0.551464089
11552	21.354384	38.765181	MYH10	0.550865073
11553	7.0960403	12.882688	ECHDC3	0.550819856
11554	33.678444	61.15294	PELP1	0.550724862
11555	1.3525266	2.4559473	GTPBP10	0.550714818
11556	11.105025	20.178031	NUPL1	0.550352267
11557	5.5028829	10.000138	MSL2	0.550280691
11558	10.244647	18.618739	SDAD1	0.550233143
11559	4.4001351	7.9973481	MOCOS	0.550199264
11560	7.8910395	14.343734	METTL2B	0.550138455
11561	1.7172009	3.122198	DFFB	0.54999743
11562	42.723139	77.71243	MRPS5	0.54975941
11563	46.147223	83.941718	IGF2BP2	0.549753146
11564	125.539	228.39407	HSPA9	0.549659652
11565	2.3554733	4.2857876	MIER3	0.549601036

11566	1.6470439	2.9983873	TTC26	0.549309933
11567	2.8359348	5.1627225	KRIT1	0.549309933
11568	2.694186	4.9058712	PIKFYVE	0.549175857
11569	35.113079	63.998992	ETV4	0.548650498
11570	5.9645713	10.87135	SP3	0.548650497
11571	5.2597418	9.5893331	ZCCHC11	0.548499229
11572	1.4605492	2.6636279	FCHO2	0.548330771
11573	20.897203	38.113215	ELP5	0.548292838
11574	17.116385	31.219587	LDOC1	0.548257903
11575	19.462038	35.498622	TLE3	0.548247703
11576	6.0068661	10.962854	C9orf114	0.547929064
11577	46.311	84.55178	UBE2E1	0.547723539
11578	49.177871	89.790099	MRPS21	0.547698153
11579	52.87341	96.54934	APH1A	0.547630987
11580	10.701596	19.54197	C14orf119	0.547621134
11581	0.6381747	1.1658093	LOC100506599	0.547409207
11582	18.066573	33.003779	TIAL1	0.547409207
11583	1.2038482	2.1991742	FAM126B	0.547409207
11584	2.4630276	4.4994267	DCUN1D2	0.547409207
11585	2.3513525	4.2954201	SIAH1	0.547409207
11586	0.7320826	1.3373589	ANKRD42	0.547409207
11587	58.742186	107.39252	NUCKS1	0.546985843
11588	15.897696	29.077442	PDCD2	0.546736383
11589	37.061957	67.793367	USP10	0.546690013
11590	10.294768	18.836247	WDR70	0.546540303
11591	1.8250003	3.3422319	MYNN	0.546042392
11592	3.2102134	5.8806095	C11orf30	0.54589807
11593	141.95063	260.0343	EIF4G1	0.545891942
11594	15.320694	28.069449	7-Sep	0.545813843
11595	1.7574747	3.2204716	NOD1	0.545719672
11596	32.699687	59.945144	C16orf13	0.545493514
11597	8.2670876	15.157731	TMEM126B	0.545404045
11598	4.477333	8.2103755	RNF6	0.545326219
11599	19.675745	36.082398	DENR	0.545300372
11600	9.316207	17.089455	TOP3A	0.545143584
11601	26.600957	48.806627	CDT1	0.545027574
11602	5.8036143	10.648295	LIMD1	0.545027574
11603	14.701683	26.978367	MTRR	0.5449434
11604	6.7975304	12.473828	ASXL2	0.544943399
11605	12.364977	22.694864	PIAS1	0.544835916
11606	42.807672	78.576009	KLC1	0.544793153
11607	10.941553	20.087527	NCAPH	0.544693883
11608	18.903899	34.710037	TRMT1	0.544623409

11609	16.381707	30.09129	MCM6	0.544400283
11610	8.019245	14.730748	TIPARP	0.544388185
11611	18.38517	33.773419	NAT10	0.544368045
11612	15.202163	27.927084	MGAT2	0.544351954
11613	0.6363344	1.1692852	INTU	0.544207984
11614	1.2039255	2.2122525	STAG3L2	0.544207983
11615	0.959482	1.7630797	KPNA5	0.544207983
11616	7.6150853	13.992969	OSGEP	0.544207983
11617	1.5763956	2.8966786	ZNF397	0.544207983
11618	1.4372379	2.6409718	IL12A	0.544207983
11619	7.6430135	14.044288	TMEM126A	0.544207983
11620	2.4498374	4.5016565	CCDC112	0.544207983
11621	1.0260931	1.8854796	SPATA7	0.544207983
11622	0.1042519	0.1917677	NR2E3	0.543636278
11623	10.788614	19.853869	EDEM1	0.543401085
11624	29.590514	54.480663	DNTTIP1	0.543137911
11625	3.7434651	6.8941457	ING5	0.542991876
11626	3.2533282	5.9921255	PTAR1	0.542933918
11627	3.473325	6.3983444	PYGO1	0.542847463
11628	2.0810221	3.8335301	PTCD2	0.542847463
11629	1.6372576	3.0160546	LOC646719	0.542847463
11630	0.9586834	1.7660271	ZNF548	0.542847463
11631	1.8957719	3.4922737	AP4S1	0.542847463
11632	6.6306909	12.222846	RAD1	0.54248338
11633	27.864	51.369437	CCDC58	0.542423695
11634	15.236218	28.090869	NSMF	0.542390393
11635	23.614835	43.595676	HSPH1	0.541678374
11636	2.1464368	3.9630195	TMEM170A	0.541616517
11637	3.1401615	5.8022472	CSNK1G1	0.541197471
11638	48.191488	89.0528	FHL1	0.54115635
11639	19.690207	36.387809	SMG7	0.541120985
11640	84.407468	156.00503	VCP	0.541056059
11641	3.3290655	6.1530404	DNAJB5	0.541043984
11642	1.3017737	2.4060404	KIAA2026	0.541043983
11643	4.7269521	8.7430736	MFN1	0.540651068
11644	65.015433	120.283	FUS	0.540520551
11645	1.4825823	2.7429958	TMEM154	0.540497474
11646	7.3346419	13.570169	ITCH	0.540497474
11647	13.569009	25.10682	FBXO11	0.540451124
11648	3.3674896	6.2343979	MIR31HG	0.54014673
11649	21.9945	40.733749	PBX2	0.539957661
11650	6.4850055	12.012082	TMCO6	0.539873583
11651	3.969161	7.3544515	AMMECR1	0.539695039

11652	3.9915565	7.3962513	LYRM7	0.539672917
11653	1.0376739	1.9234857	NUDT3	0.53947574
11654	3.243572	6.012452	GNPDA2	0.53947574
11655	4.8192551	8.9353651	MRPL19	0.539346183
11656	160.19273	297.01788	LDHB	0.539336986
11657	3.2173725	5.9665605	TRAPPC8	0.539234039
11658	5.1226399	9.5019406	USP13	0.539115128
11659	2.8985454	5.3776604	CBR4	0.538997481
11660	3.2804856	6.0880226	KIAA0753	0.538842553
11661	0.01	0.0185639	SLC24A2	0.538681014
11662	4.9827731	9.2523878	TMEM87B	0.53853915
11663	29.02637	53.907864	TARDBP	0.538444086
11664	6.6662445	12.382478	PMPCB	0.538361121
11665	17.233501	32.022933	RBMS1	0.538161228
11666	7.480007	13.902021	SCFD2	0.538051784
11667	5.139898	9.5544783	PPP1R13L	0.537956946
11668	1.9481318	3.6213526	C12orf5	0.537956946
11669	5.4082915	10.054146	FBXO34	0.537916562
11670	91.30784	169.74858	TOMM6	0.537900452
11671	5.4095636	10.057075	CRLS1	0.537886376
11672	8.9262918	16.597767	MED1	0.537800766
11673	2.540436	4.7248323	INTS6	0.537677488
11674	2.8556289	5.3156129	KIAA1432	0.537215367
11675	8.7422681	16.274353	COPS2	0.537180673
11676	5.0027827	9.3155401	AP4B1	0.53703625
11677	6.322232	11.775829	N6AMT2	0.536882107
11678	13.295128	24.765936	FAM43A	0.536831251
11679	32.720259	60.952826	POP7	0.536812823
11680	3.0477544	5.6794293	ZSCAN29	0.536630404
11681	15.454612	28.800762	METAP2	0.536604279
11682	12.492613	23.285092	LTV1	0.536506927
11683	3.0911468	5.7616159	PNMAL1	0.536506927
11684	12.494938	23.294719	RNF44	0.536384994
11685	8.9818153	16.752566	MTMR4	0.536145643
11686	9.3662697	17.469637	EIF2AK2	0.536145643
11687	0.7574229	1.4127186	DDX26B	0.536145643
11688	2.4968703	4.6570748	DDX59	0.536145643
11689	1.5347675	2.8625944	RNPC3	0.536145643
11690	7.383641	13.771708	FAM204A	0.536145643
11691	0.5984806	1.116265	KIAA1328	0.536145643
11692	2.1249767	3.9634318	CCDC82	0.536145643
11693	3.351244	6.2506224	DNMT3B	0.536145643
11694	15.705147	29.300382	YRDC	0.536004849

11695	7.5619247	14.108349	RPP14	0.535989332
11696	3.6000887	6.7182616	SIRT1	0.535866109
11697	25.299299	47.21425	POLR2B	0.535840321
11698	31.316029	58.451835	UHRF1	0.535757834
11699	32.550312	60.786651	LINC00657	0.535484551
11700	65.914098	123.11714	G3BP1	0.535377085
11701	39.964357	74.661317	NOP16	0.535275277
11702	61.537321	115.02731	SNRPF	0.534980109
11703	3.6630324	6.8474437	HCG18	0.534948889
11704	21.971744	41.080327	EIF4EBP2	0.534848315
11705	0.6064001	1.1338288	KIAA1009	0.534825088
11706	1.4084219	2.6334254	CEP97	0.534825087
11707	0.8971136	1.6773963	PAX6	0.534825087
11708	4.2205119	7.8913874	AKTIP	0.534825087
11709	7.1033915	13.28816	YTHDC1	0.534565463
11710	5.3167585	9.9467361	AARS2	0.534522926
11711	2.7045781	5.0607303	CTC1	0.534424469
11712	14.738555	27.578368	PFKFB3	0.534424469
11713	4.5975887	8.6082914	COG5	0.534088414
11714	1.2231239	2.2903164	ZNF407	0.534041461
11715	45.490909	85.191289	AHCYL1	0.533985457
11716	4.1086999	7.6957376	TRIM24	0.53389293
11717	695.85756	1303.5734	MT2A	0.533807725
11718	85.879872	160.8901	IRAK1	0.533779705
11719	21.999771	41.217603	ABT1	0.533746966
11720	6.2301794	11.674109	ATRIP	0.533674926
11721	8.1445823	15.261317	NOP9	0.533674926
11722	20.621834	38.64119	OAT	0.533674926
11723	1.0707746	2.0064172	PIBF1	0.533674926
11724	32.462322	60.848123	CMIP	0.533497507
11725	9.0728015	17.017232	SNHG12	0.533153759
11726	25.286969	47.435424	METTL5	0.533081953
11727	7.0108011	13.161766	CRYL1	0.532664178
11728	0.9065485	1.7019138	GPR137C	0.532664178
11729	4.7379358	8.8947896	PDHX	0.532664178
11730	1.5915505	2.9879061	ZUFSP	0.532664178
11731	3.1671657	5.9473972	FAM105B	0.5325297
11732	1.7924657	3.3689432	INTS2	0.532055766
11733	8.8442107	16.624987	RPP38	0.531983022
11734	18.747235	35.249791	SDCCAG3	0.53183962
11735	4.0670665	7.6481836	CEP78	0.531768944
11736	22.161508	41.685994	PCBD1	0.531629591
11737	3.2241835	6.0662784	NLK	0.531492844

11738	3.4795945	6.548486	ACAD8	0.531358628
11739	2.9725449	5.5942348	TMEM161B	0.531358628
11740	12.769948	24.04251	NMD3	0.531140375
11741	27.241177	51.299927	UBL4A	0.531017858
11742	2.6327065	4.9582915	CCDC28A	0.530970492
11743	2.4351725	4.5862671	ANKRD9	0.530970492
11744	19.542339	36.81688	PNMA1	0.530798341
11745	8.9890629	16.943503	FOXD1	0.530531552
11746	41.125566	77.518403	BUB3	0.530526493
11747	24.387908	45.972897	RNF220	0.530484478
11748	11.957348	22.556584	TCHP	0.530104565
11749	5.1627671	9.7424941	EBAG9	0.529922524
11750	39.615209	74.758585	CTBP1	0.52990849
11751	5.3871091	10.167383	ANKRD28	0.529842245
11752	10.313286	19.466908	FAM83G	0.52978551
11753	1.722988	3.2533314	C5orf34	0.529607282
11754	1.8504818	3.4940642	HNRNPA1L2	0.529607281
11755	2.3643379	4.4643229	MRM1	0.529607281
11756	0.9275942	1.7514756	RPGR	0.529607281
11757	24.067724	45.465192	PWP1	0.529365947
11758	9.8363033	18.581638	KIAA0317	0.529356092
11759	58.709867	110.93867	SNHG16	0.529210126
11760	5.6090963	10.598997	ZNF131	0.529210125
11761	25.616882	48.421741	SMAD3	0.529036783
11762	4.1819298	7.9050387	TTC13	0.529020784
11763	1.541883	2.9145981	FLVCR1	0.529020784
11764	29.341446	55.480229	FAM96A	0.528863103
11765	12.21928	23.10917	EIF4G3	0.528763259
11766	15.725004	29.747526	RFX5	0.528615507
11767	12.406411	23.47892	KIAA0368	0.528406358
11768	34.663584	65.604113	NUP98	0.528375172
11769	0.7710171	1.4602665	AKAP5	0.527997533
11770	4.4498114	8.4277125	TTC8	0.527997533
11771	1.9867281	3.7627602	ITGA11	0.527997533
11772	9.1269854	17.286038	MTFR1	0.527997533
11773	0.899728	1.7040383	ZNF286A	0.527997533
11774	0.3847375	0.7290718	ALG1L9P	0.527708714
11775	8.4171911	15.953655	FAM193A	0.527602663
11776	15.919883	30.195677	HTATSF1	0.52722391
11777	4.7404961	8.9929486	TST	0.527134792
11778	45.16286	85.6788	FUBP1	0.527118255
11779	1.6726476	3.1753971	C19orf44	0.526752256
11780	0.709915	1.3477209	GAB1	0.526752255

11781	9.1854208	17.440857	PTDSS2	0.526661091
11782	13.59589	25.818396	PREP	0.526597009
11783	8.9372105	16.97245	FANCE	0.526571613
11784	8.0246618	15.24115	KDELC2	0.526512877
11785	4.4532379	8.4584523	CHD6	0.526483778
11786	132.21235	251.1583	RPL13	0.526410457
11787	130.47398	247.90633	RPS10	0.526303541
11788	4.3447246	8.2613252	AEBP2	0.525911336
11789	6.9727652	13.259727	MBD4	0.5258604
11790	52.474694	99.807267	TAF10	0.525760255
11791	81.140933	154.35171	CD44	0.525688593
11792	1.7049173	3.2462008	LRRC8B	0.525203895
11793	43.9245	83.650778	RARS	0.525093743
11794	9.8264937	18.724567	MGME1	0.524791518
11795	86.31932	164.48579	KARS	0.524782824
11796	2.9364206	5.5962311	NUDT11	0.524713966
11797	3.1353987	5.9779917	KIAA0754	0.524490303
11798	11.658997	22.237053	PRKAA1	0.524304971
11799	87.213638	166.42565	HNRNPU	0.524039653
11800	5.3417747	10.19635	MTIF2	0.523890885
11801	7.2508524	13.843568	CCDC8	0.523770478
11802	1.8658357	3.5627145	LZTFL1	0.523711838
11803	3.7006984	7.0670652	SENP6	0.523654206
11804	3.3437545	6.386795	SLC36A4	0.523541857
11805	14.991878	28.638487	NOC4L	0.523487094
11806	23.39471	44.695872	OLA1	0.523419914
11807	723.49002	1382.5618	HSP90AB1	0.52329668
11808	1.3610538	2.6019998	FZD5	0.523079909
11809	5.9017245	11.282644	PBRM1	0.523079909
11810	1.8071695	3.4557869	ZNF12	0.522940085
11811	2.6452475	5.0609421	MPHOSPH9	0.522678876
11812	1.6788751	3.2120585	MME	0.522678876
11813	3.9893371	7.6324819	PARS2	0.522678876
11814	100.16566	191.66968	TPD52L2	0.522595219
11815	5.620267	10.762264	TGS1	0.522219782
11816	7.9265689	15.183073	GTPBP3	0.522066188
11817	6.082144	11.653372	LAGE3	0.521921371
11818	5.2796726	10.117036	ANKS1A	0.52185963
11819	3.818839	7.3206188	SMAD1	0.52165522
11820	7.2748547	13.950047	MRPL35	0.521493215
11821	17.370031	33.321207	PTP4A1	0.521290565
11822	4.5362677	8.7092588	USP54	0.520855775
11823	3.4775556	6.6773465	PPM1A	0.520799037

11824	23.758901	45.627281	GRWD1	0.520716991
11825	34.476552	66.227775	PSRC1	0.520575422
11826	1.63922	3.1495154	RICTOR	0.520467367
11827	4.4334194	8.5196355	ARFGEF1	0.520376653
11828	132.76347	255.24804	FBL	0.520135129
11829	6.1915266	11.905483	SHQ1	0.520056741
11830	2.2809896	4.3869245	TRAF6	0.519951856
11831	2.3198944	4.4620349	CEP192	0.519918474
11832	15.742714	30.285671	RSRC2	0.519807354
11833	73.057344	140.5531	C14orf166	0.519784633
11834	19.852521	38.198594	BYSL	0.519718624
11835	3.4463288	6.632714	KAZN	0.519595562
11836	14.632003	28.164491	LIX1L	0.519519524
11837	5.4939168	10.581932	AGGF1	0.519179009
11838	3.6432452	7.0185138	CCNT1	0.519090692
11839	7.324007	14.128342	KIF1B	0.518391109
11840	148.1671	286.03564	NUDC	0.518002243
11841	25.464377	49.196261	ALDH18A1	0.517607971
11842	9.8797119	19.088967	BCAT1	0.517561376
11843	0.0318022	0.0615132	ADCY2	0.516997592
11844	0.0197385	0.0381792	TMEM178B	0.516997592
11845	0.0412183	0.0797262	TRIL	0.516997592
11846	0.0261152	0.0505132	GPR179	0.516997591
11847	0.0419822	0.0812038	PCDH11Y	0.51699759
11848	0.0537944	0.1040515	LOC440354	0.51699759
11849	0.0371876	0.07193	FAM83H	0.516997589
11850	0.0482965	0.0934173	NEURL	0.516997589
11851	0.0718619	0.1389985	MFAP5	0.516997588
11852	0.0858318	0.1660196	VENTX	0.516997588
11853	0.0420245	0.0812857	OPRK1	0.516997588
11854	0.0797549	0.1542655	DLX3	0.516997588
11855	0.0681936	0.131903	CALHM1	0.516997588
11856	0.0868693	0.1680266	PLA2G4E	0.516997588
11857	0.0227262	0.0439581	FLG2	0.516997588
11858	0.0674214	0.1304095	LOC728342	0.516997588
11859	0.0624886	0.1208683	GAL3ST3	0.516997587
11860	0.1066528	0.2062926	ANXA8L1	0.516997587
11861	0.058261	0.112691	IGSF11	0.516997587
11862	0.072689	0.1405984	C2	0.516997587
11863	0.0854797	0.1653387	UCP3	0.516997587
11864	0.0493955	0.0955429	SRL	0.516997586
11865	0.0940431	0.1819024	ASPRV1	0.516997586
11866	0.0270087	0.0522415	POM121L8P	0.516997586

11867	0.1005304	0.1944504	TRIM54	0.516997586
11868	0.2148448	0.4155626	LOC100507410	0.516997586
11869	0.0923757	0.1786772	FBXW4P1	0.516997586
11870	0.0686654	0.1328157	FNDC5	0.516997586
11871	0.0957278	0.1851611	TBC1D3G	0.516997586
11872	0.0996174	0.1926844	LOC400084	0.516997586
11873	0.084304	0.1630646	TIGD4	0.516997586
11874	0.0942558	0.1823137	MGC2889	0.516997585
11875	0.0508168	0.098292	RUNDC3B	0.516997585
11876	0.1546564	0.2991434	STAR	0.516997585
11877	0.2053197	0.3971386	RHPN1-AS1	0.516997585
11878	0.0717135	0.1387115	ANKS4B	0.516997585
11879	0.0655344	0.1267597	ENPP3	0.516997585
11880	0.0409912	0.0792871	GLDN	0.516997585
11881	0.172231	0.3331369	SUMO1P3	0.516997585
11882	0.2341567	0.4529165	LINC00114	0.516997585
11883	0.1170784	0.2264583	LRG1	0.516997585
11884	0.1474525	0.2852092	P2RY8	0.516997585
11885	0.1987597	0.3844499	NUTMG	0.516997585
11886	0.0648412	0.1254187	LINC00617	0.516997585
11887	0.1187462	0.2296842	MAGEA1	0.516997585
11888	0.2846016	0.5504892	GPLD1	0.516997585
11889	0.2121115	0.4102755	GHRLOS	0.516997585
11890	0.1046182	0.2023573	PCAT1	0.516997585
11891	0.1583583	0.3063037	PRSS35	0.516997585
11892	0.3394129	0.6565076	PMS2L2	0.516997585
11893	0.105412	0.2038926	P2RY11	0.516997585
11894	0.2304212	0.445691	ADAMTS17	0.516997585
11895	0.1074778	0.2078884	HSD11B2	0.516997585
11896	0.3360198	0.6499447	PRICKLE2-AS3	0.516997585
11897	0.1890245	0.3656197	ANKRD65	0.516997585
11898	0.0439754	0.0850592	LSAMP	0.516997585
11899	0.2454647	0.4747888	BOK-AS1	0.516997585
11900	0.1049872	0.2030709	LOC256880	0.516997585
11901	0.1353244	0.2617505	LRP4-AS1	0.516997585
11902	0.2328486	0.4503863	LOC100289656	0.516997585
11903	0.1269696	0.2455904	LINC00261	0.516997585
11904	0.1241952	0.2402239	PGAM4	0.516997585
11905	0.196789	0.380638	LOC554206	0.516997585
11906	0.2451759	0.4742302	SIGLEC10	0.516997585
11907	0.2682104	0.5187847	SPG20OS	0.516997585
11908	0.1157775	0.2239421	FRMD8P1	0.516997585
11909	0.1490697	0.2883374	RP1-177G6.2	0.516997585

11910	0.0815335	0.1577057	TIPARP-AS1	0.516997585
11911	0.147644	0.2855797	ZNF491	0.516997585
11912	0.3026863	0.5854694	LOC100288846	0.516997585
11913	0.0978861	0.1893357	LOC440925	0.516997585
11914	0.5878688	1.1370824	RBM12B-AS1	0.516997585
11915	0.390062	0.7544755	CPEB3	0.516997585
11916	0.2656463	0.513825	WDFY3-AS2	0.516997585
11917	0.278237	0.5381785	SSTR2	0.516997585
11918	0.087306	0.1688713	AGAP9	0.516997585
11919	0.9908059	1.9164613	LOC254896	0.516997585
11920	0.1535737	0.2970491	RFPL3S	0.516997585
11921	0.2097278	0.4056649	AIPL1	0.516997584
11922	0.3774214	0.7300254	ZNF782	0.516997584
11923	0.8140605	1.5745926	EAF2	0.516997584
11924	0.8140605	1.5745926	LOC255512	0.516997584
11925	0.9693	1.8748637	ZSCAN16	0.516997584
11926	0.6432083	1.2441225	ZNF107	0.516997584
11927	0.3924661	0.7591256	DKFZp566F0947	0.516997584
11928	0.6402125	1.2383279	ALG10B	0.516997584
11929	1.0400714	2.011753	KANSL1L	0.516997584
11930	1.0687154	2.0671574	LOC728819	0.516997584
11931	0.4261748	0.8243266	HM13-AS1	0.516997584
11932	0.7016818	1.3572246	EFNA3	0.516997584
11933	1.8174375	3.5153694	DDT	0.516997584
11934	2.019375	3.905966	ENTPD5	0.516997584
11935	3.5061956	6.7818413	B3GALNT2	0.516997584
11936	0.7616513	1.4732201	FAM171B	0.516997584
11937	2.4984425	4.8326	SRFBP1	0.516997584
11938	1.857945	3.5937209	LOC100506233	0.516997584
11939	0.4801832	0.9287919	NS3BP	0.516997584
11940	3.992916	7.7232779	FKBPL	0.516997584
11941	7.0817855	13.697908	FARP2	0.516997584
11942	1.0632628	2.0566107	FAM200A	0.516997584
11943	0.1601226	0.3097162	BSN-AS2	0.516997584
11944	1.2139777	2.3481303	HIST2H2BA	0.516997584
11945	0.1905367	0.3685446	GOLGA6L9	0.516997584
11946	0.5700651	1.1026455	ARHGAP6	0.516997584
11947	4.1737469	8.0730491	ZZZ3	0.516997584
11948	4.3042926	8.3255565	PIGF	0.516997584
11949	0.3359927	0.6498923	LOC100507266	0.516997584
11950	2.0973417	4.0567728	NUDT17	0.516997584
11951	0.935514	1.8095133	THNSL1	0.516997584
11952	4.2327258	8.1871288	DIRC2	0.516997584

11953	0.7799382	1.5085917	SFN	0.516997584
11954	3.1345873	6.0630599	AUNIP	0.516997584
11955	1.2231385	2.3658495	THAP6	0.516997584
11956	2.1086624	4.0786697	BCL2L11	0.516997584
11957	2.4164676	4.6740404	ARL15	0.516997584
11958	2.9067733	5.6224119	KLHL17	0.516997584
11959	2.6049938	5.0386962	SNORD14E	0.516997584
11960	0.6089102	1.1777814	FNDC8	0.516997584
11961	1.362898	2.6361787	TMEM187	0.516997584
11962	3.7544826	7.2620893	DMTF1	0.516997584
11963	0.5505931	1.064982	C9orf169	0.516997584
11964	2.8310557	5.4759553	PHF16	0.516997584
11965	0.7850308	1.5184418	LOC729732	0.516997584
11966	0.6111422	1.1820988	LOC389634	0.516997584
11967	2.3681761	4.5806329	SNORD14C	0.516997584
11968	0.8208295	1.5876854	CISH	0.516997584
11969	0.4491369	0.8687407	FLJ35024	0.516997584
11970	15.662754	30.295604	PAQR4	0.516997584
11971	2.9182797	5.6446679	SLC35G1	0.516997584
11972	0.3081921	0.596119	SLC40A1	0.516997584
11973	0.1500356	0.2902057	CLDN6	0.516997584
11974	0.196975	0.3809978	LOC388948	0.516997584
11975	0.1575204	0.3046831	LRRC69	0.516997584
11976	0.0787602	0.1523415	SLC44A4	0.516997584
11977	0.2622477	0.5072513	LFNG	0.516997584
11978	1.9716942	3.8137396	KIF14	0.516997584
11979	0.3599301	0.6961929	RPS6KA2-IT1	0.516997584
11980	0.6627881	1.2819945	SLC5A10	0.516997584
11981	0.0549577	0.1063016	APOBR	0.516997584
11982	1.4874495	2.8770917	ASTE1	0.516997584
11983	1.2490519	2.4159724	CCDC163P	0.516997584
11984	1.2831763	2.4819774	AASDH	0.516997584
11985	0.1004819	0.1943567	SOX3	0.516997584
11986	0.0856906	0.1657466	ASAH2	0.516997584
11987	0.4283649	0.8285626	LOC100128531	0.516997584
11988	0.0552344	0.1068369	RPL34-AS1	0.516997584
11989	0.3473325	0.6718262	LOC100130093	0.516997584
11990	0.4394296	0.8499646	LOC100128398	0.516997584
11991	0.3971405	0.7681671	RAET1E	0.516997584
11992	0.999518	1.9333127	LINC00662	0.516997584
11993	0.9646811	1.8659297	SHPRH	0.516997584
11994	0.4271828	0.8262763	RALGPS1	0.516997584
11995	0.099665	0.1927765	KRT86	0.516997584

11996	0.3136185	0.606615	LOC113230	0.516997584
11997	0.370159	0.7159781	LOC391322	0.516997584
11998	0.2738495	0.5296921	LOC100132111	0.516997584
11999	0.0528396	0.1022048	HAP1	0.516997584
12000	0.0850263	0.1644617	DNAAF1	0.516997584
12001	0.3479124	0.6729477	PCBP1-AS1	0.516997584
12002	0.5988491	1.158321	SCARNA9L	0.516997584
12003	0.0394696	0.0763439	SLC26A7	0.516997584
12004	0.1283248	0.2482116	LOC339568	0.516997584
12005	0.325726	0.6300339	LNX1	0.516997584
12006	0.2710007	0.5241817	LOH12CR2	0.516997584
12007	0.3157568	0.6107511	MURC	0.516997583
12008	0.0945122	0.1828098	SLC6A4	0.516997583
12009	0.1716635	0.3320393	TPI1P3	0.516997583
12010	0.3414146	0.6603796	CYP2B6	0.516997583
12011	0.1196323	0.2313982	HCG27	0.516997583
12012	0.1358979	0.2628599	RNF222	0.516997583
12013	0.2678657	0.5181179	APCDD1L	0.516997583
12014	0.1396779	0.2701714	CDC20B	0.516997583
12015	0.1975351	0.3820812	ST8SIA4	0.516997583
12016	0.2680379	0.5184511	PROZ	0.516997583
12017	0.1723734	0.3334125	CLDN20	0.516997583
12018	0.244744	0.4733948	DLG1-AS1	0.516997583
12019	0.3063197	0.5924973	LPAL2	0.516997583
12020	0.0842018	0.1628669	AGMO	0.516997583
12021	0.1146943	0.2218468	SPINT2	0.516997583
12022	0.1897128	0.366951	CHST4	0.516997583
12023	0.2592034	0.5013628	REREP3	0.516997583
12024	0.0761694	0.1473303	ZNF443	0.516997583
12025	0.2297679	0.4444274	SRSF12	0.516997583
12026	0.319405	0.6178076	GREB1	0.516997583
12027	0.0750989	0.1452597	FRZB	0.516997583
12028	0.1250147	0.2418091	BRE-AS1	0.516997583
12029	0.1500897	0.2903102	SLC9A3	0.516997583
12030	0.1500897	0.2903102	TGM1	0.516997583
12031	0.1887677	0.3651229	LOC653712	0.516997583
12032	0.2362806	0.4570246	LOC389765	0.516997583
12033	0.1797322	0.3476461	HOXD3	0.516997583
12034	0.063614	0.1230451	ZNF572	0.516997583
12035	0.0433894	0.0839258	SLC28A3	0.516997583
12036	0.0643506	0.1244699	TPTE2P1	0.516997583
12037	0.0933271	0.1805176	TMPRSS5	0.516997583
12038	0.1410965	0.2729152	LOC650368	0.516997583

12039	0.1009688	0.1952983	GCNT7	0.516997582
12040	0.0599711	0.1159988	SNRK-AS1	0.516997582
12041	0.0967051	0.1870514	CAPS2	0.516997582
12042	0.0822738	0.1591377	LOC284080	0.516997582
12043	0.048465	0.0937432	GPR83	0.516997582
12044	0.1336751	0.2585604	CD160	0.516997582
12045	0.1086546	0.2101646	TDRD5	0.516997582
12046	0.1534983	0.2969033	PARK2	0.516997582
12047	0.1170126	0.2263311	NIPSNAP3B	0.516997582
12048	0.0715658	0.1384257	CRTAC1	0.516997582
12049	0.1480461	0.2863573	PPIL6	0.516997582
12050	0.0836946	0.1618858	ERVV-1	0.516997582
12051	0.1059479	0.2049292	LOC100128714	0.516997582
12052	0.1179397	0.2281243	SLFN12L	0.516997582
12053	0.111543	0.2157515	NFAM1	0.516997582
12054	0.06458	0.1249134	LRRC37A6P	0.516997582
12055	0.072512	0.140256	LOC100506421	0.516997581
12056	0.0687333	0.1329471	POU2AF1	0.51699758
12057	0.052139	0.1008496	PIWIL2	0.51699758
12058	0.0335263	0.0648481	TAF1L	0.51699758
12059	0.0528128	0.102153	GPRASP2	0.516997579
12060	0.0220926	0.0427325	DCX	0.516997577
12061	0.0317973	0.0615038	ACSL6	0.516997576
12062	0.0388443	0.0751343	WDR96	0.516997576
12063	0.0303878	0.0587774	IPCEF1	0.516997575
12064	10.338356	20.024611	BCLAF1	0.516282512
12065	9.0848678	17.602043	CUL3	0.51612575
12066	83.086105	160.99249	RBMX	0.516086843
12067	51.415169	99.690333	UQCRC2	0.515748798
12068	5.6411311	10.950299	MAP3K7	0.515157735
12069	82.77866	160.70239	EIF3B	0.51510536
12070	59.859431	116.24226	PPA1	0.514954115
12071	26.864876	52.180672	AAAS	0.514843428
12072	11.018605	21.404944	ACSL3	0.514769146
12073	4.0661391	7.9030885	MTR	0.514500011
12074	2.3665297	4.6008025	CENPE	0.514373231
12075	19.158954	37.271088	CDCA7	0.514043312
12076	10.982075	21.367717	SLC25A13	0.513956422
12077	21.88506	42.584547	ASAP1	0.513920218
12078	3.4390741	6.6925729	MTMR10	0.513864266
12079	20.286677	39.494205	BCCIP	0.513662116
12080	18.059706	35.161711	MKI67IP	0.513618515
12081	6.9775142	13.592167	WAPAL	0.51334819

12082	11.423622	22.253913	CHAMP1	0.513330935
12083	5.6242624	10.958302	SCAF11	0.513242154
12084	1377.3995	2683.7687	RPS27	0.5132333
12085	7.1024695	13.843053	TLK1	0.51307102
12086	27.169301	52.964488	TTLL12	0.512972019
12087	7.4083657	14.446285	RALGAPB	0.512821513
12088	28.036349	54.696661	XRN2	0.512578801
12089	93.387689	182.24338	EIF4EBP1	0.512433923
12090	33.505223	65.385088	TMPO	0.512429122
12091	3.2087908	6.2620035	TMEM194B	0.512422384
12092	10.712864	20.925454	STK17A	0.511953706
12093	7.1812371	14.029175	TMEM5	0.511878796
12094	1.1343321	2.216017	TMOD2	0.511878796
12095	2.8082606	5.4869167	TET3	0.511810317
12096	2.6562419	5.1936685	TIAM1	0.51143847
12097	18.596516	36.379591	MDC1	0.511179907
12098	8.9564954	17.550515	TMEM209	0.510326648
12099	1.9745919	3.8709574	USPL1	0.510104283
12100	6.2974667	12.350022	TRMT2B	0.509915426
12101	22.940699	44.996149	SAMD1	0.509836953
12102	199.90094	392.19639	EEF1B2	0.509696024
12103	3.9367618	7.724068	SH3BP2	0.509674672
12104	4.9935982	9.8019365	CEP164	0.509450174
12105	60.476718	118.75515	MRPS34	0.509255524
12106	82.22115	161.46876	CD151	0.509207771
12107	29.5606	58.075796	ELAC2	0.509000351
12108	1.8661729	3.6688099	HIC2	0.508658913
12109	18.183878	35.764863	MRPL46	0.508428564
12110	13.782331	27.109151	ANKRD17	0.508401425
12111	6.5176626	12.82043	CCNYL1	0.508380958
12112	48.259664	94.983663	RNF167	0.508083833
12113	8.6115496	16.954291	MB21D2	0.507927451
12114	15.898124	31.306605	ISCA2	0.507820112
12115	22.053968	43.429681	SLC38A2	0.507808665
12116	3.6001663	7.0958241	PIAS2	0.507364089
12117	10.57306	20.845693	TEX2	0.507205963
12118	4.9171291	9.6974213	TAF8	0.507055323
12119	2.4494535	4.8326	FAM86C1	0.506860377
12120	6.9122796	13.647046	BPTF	0.506503723
12121	1.9506974	3.8517334	C19orf26	0.506446613
12122	353.10048	697.29249	CHCHD2	0.506387895
12123	12.975265	25.623576	DENND5A	0.50637994
12124	19.042536	37.623075	DNAJC11	0.5061398

12125	4.052378	8.0111955	MED17	0.505839363
12126	6.2927025	12.44012	CIDEB	0.505839363
12127	136.51237	269.87377	HNRNPA2B1	0.505837862
12128	6.498108	12.847007	RBM26	0.50580716
12129	6.7298009	13.306352	ZSWIM1	0.505758506
12130	50.573382	100.00368	PLAUR	0.505715224
12131	6.9810263	13.809902	ACADM	0.505508749
12132	1.9063572	3.7711655	ARL10	0.505508749
12133	1.4508826	2.8701434	IFT81	0.505508749
12134	64.391322	127.46291	MRPL9	0.505176942
12135	22.586906	44.712562	ASNS	0.505157945
12136	4.4675807	8.8447227	NUDCD1	0.505112582
12137	14.144762	28.010851	MPST	0.504974385
12138	0.7006708	1.3875372	KIAA1551	0.504974385
12139	6.9191929	13.702067	DIS3L	0.504974385
12140	50.430007	99.869542	PRPF6	0.504958831
12141	7.0879213	14.063121	ATP6V1C1	0.504007695
12142	0.7630739	1.5148133	ITGA10	0.503741236
12143	9.6048284	19.066989	NIF3L1	0.503741236
12144	2.9763898	5.9085688	ARL13B	0.503741236
12145	0.8467901	1.6810021	ZNF614	0.503741236
12146	1.9489371	3.868925	TMCC1	0.503741236
12147	27.7866	55.17551	GEMIN6	0.503603864
12148	2.4109151	4.7876552	OBFC1	0.503569076
12149	61.85281	122.86404	ODC1	0.503424855
12150	2.9685396	5.8970689	UHRF1BP1L	0.503392385
12151	17.072939	33.932144	KATNB1	0.503149434
12152	1.2953008	2.5750243	ARID4A	0.503024677
12153	1.9953144	3.9666332	METTL21D	0.503024676
12154	29.771357	59.201008	ATG3	0.502885988
12155	6.4513237	12.828959	FASTKD5	0.502871967
12156	2.1941147	4.3640674	NBPF9	0.502768293
12157	347.38485	690.96333	MSN	0.502754392
12158	4.0435724	8.046873	UBE2D1	0.502502325
12159	2.4298982	4.8382542	ZNF101	0.502226225
12160	16.763689	33.387863	CBX5	0.502089312
12161	3.6792534	7.3290126	RECQL	0.502012147
12162	234.74264	467.66081	RPSA	0.50195064
12163	1.4194217	2.8278747	DENND4A	0.501939402
12164	17.819176	35.517064	TM9SF3	0.501707452
12165	21.60083	43.065357	ARIH2	0.501582519
12166	99.640251	198.66691	HMGB1	0.501544268
12167	4.2401925	8.4650755	MYO5A	0.500904274

12168	2.0968466	4.1866479	PARPBP	0.50084141
12169	24.325945	48.575237	PSMA5	0.500789011
12170	15.358137	30.670325	PTK2	0.500749089
12171	7.7558055	15.488694	TSPAN6	0.500739799
12172	24.984752	49.896407	GSPT1	0.500732492
12173	5.4685551	10.922445	CENPK	0.500671345
12174	27.011437	53.976763	BRD7	0.500427149
12175	1.0672559	2.1331455	ZNF749	0.500320243
12176	6.7814606	13.558444	BBS5	0.500165105
12177	126.12357	252.21275	EIF6	0.500068192
12178	3.8351168	7.6701253	ZBTB24	0.500007053
12179	2.1550125	4.310424	SLC4A3	0.499953708
12180	5.7826969	11.567551	KLF7	0.499906755
12181	5.7410331	11.484458	ZBED4	0.499895883
12182	19.591765	39.194104	IGFBP3	0.499865111
12183	1.9795563	3.9609796	SCLT1	0.499764332
12184	54.764482	109.61157	CHD4	0.499623195
12185	18.708591	37.446674	UBE2E2	0.499606228
12186	357.20631	715.06063	RPL32	0.499546877
12187	1.6712857	3.3467706	DMXL2	0.499372667
12188	5.840408	11.69549	GTF2E1	0.499372666
12189	7.3952655	14.815122	PURB	0.499170081
12190	0.9325853	1.8682716	FAM179B	0.499170081
12191	4.5889658	9.195089	SLC7A2	0.499067032
12192	12.553198	25.153806	SHMT1	0.499057605
12193	3.7707054	7.5566344	KIAA1598	0.498992693
12194	4.7262821	9.4750819	STRN3	0.49881174
12195	1.8478329	3.7049237	NBPF8	0.498750611
12196	10.837569	21.731781	FAM57A	0.498696785
12197	6.0533691	12.151731	ARHGEF28	0.498148714
12198	4.3339171	8.7022045	TAF2	0.498025196
12199	0.8217147	1.6505283	ZNF26	0.497849525
12200	1008.8798	2026.5714	MTRNR2L8	0.497825906
12201	21.22804	42.642948	UBE2R2	0.497808915
12202	10.64815	21.393004	STK39	0.497739794
12203	25.512356	51.268161	MYO10	0.497625726
12204	6.8296078	13.732569	ADNP2	0.497329198
12205	7.6893736	15.464678	MAP2K5	0.49722172
12206	2.4488191	4.9299473	PLCL2	0.496723169
12207	4.2320223	8.5238759	USP34	0.496490373
12208	23.842316	48.038417	POLR3C	0.496317681
12209	5.631362	11.346285	GNE	0.496317681
12210	10.401178	20.966793	BCS1L	0.496078607

12211	9.153528	18.457029	CDK12	0.495937236
12212	4.3186845	8.7088576	PPCDC	0.495895642
12213	1.9756736	3.9861539	GAB2	0.495634048
12214	0.9712641	1.9603438	ZNF536	0.495456018
12215	18.945409	38.238327	CHD1L	0.495456018
12216	5.9908313	12.093996	INTS8	0.495355825
12217	6.7263712	13.581084	RSRC1	0.495274997
12218	51.728617	104.44423	PPP1CC	0.495274996
12219	2.4822598	5.0146897	NRIP1	0.494997687
12220	5.3842865	10.881848	TTK	0.494795234
12221	491.50066	993.52378	RPL5	0.49470447
12222	11.384352	23.021041	GNAQ	0.494519428
12223	8.7045086	17.601955	UBIAD1	0.494519428
12224	21.806908	44.128166	AMPD2	0.494172084
12225	3.7539685	7.596868	ARHGAP5	0.494146862
12226	3.1952223	6.4664698	PRKCH	0.494121585
12227	7.4180022	15.020807	LIN52	0.493848439
12228	6.6888506	13.544339	ANKRD1	0.493848438
12229	18.658878	37.797842	TCEB3	0.493649306
12230	4.3720175	8.859246	ETV3	0.493497694
12231	1.425534	2.8886336	GATA3	0.493497694
12232	15.233882	30.88043	FNTA	0.493318306
12233	13.306451	26.975338	AP3B1	0.493282099
12234	4.2550473	8.6323591	EXOC5	0.492918245
12235	4.2142643	8.5509991	STXBP3	0.492838819
12236	43.74593	88.785302	NELFCD	0.492715897
12237	6.3874552	12.96586	LDLRAD3	0.492636441
12238	7.9181032	16.07732	E2F3	0.49250144
12239	1.1915352	2.419957	ECE2	0.492378652
12240	3.5262183	7.1615986	LOC100289092	0.492378652
12241	0.3514918	0.7138649	ALS2CR8	0.492378651
12242	0.6928175	1.4070827	FANCB	0.492378651
12243	0.3274662	0.6650699	SLC4A5	0.492378651
12244	12.791629	25.98517	STRIP1	0.492266493
12245	60.655662	123.24288	CHCHD10	0.492163639
12246	11.154174	22.667305	SLC25A33	0.492082038
12247	36.95974	75.149785	CD68	0.491814321
12248	5.9630084	12.125402	NLE1	0.49177819
12249	1.6431717	3.3426913	PRKAR2B	0.491571474
12250	9.4994346	19.336694	ATP6V1A	0.491264673
12251	16.082638	32.739289	EVL	0.491233585
12252	19.012625	38.715983	CTCF	0.4910795
12253	57.88875	117.89133	CDC123	0.491034824

12254	14.57014	29.681275	APBB1	0.490886595
12255	14.575701	29.698427	MYO19	0.490790333
12256	42.853982	87.318476	NOB1	0.490777941
12257	2.6791488	5.4597443	SIX2	0.490709572
12258	40.473132	82.504442	THAP4	0.490557011
12259	24.151436	49.243172	CSNK2A2	0.490452476
12260	71.024289	144.84116	PXN	0.490359828
12261	1.8245864	3.7210014	ARHGEF26	0.490348224
12262	7.2983061	14.886107	DCP1A	0.490276361
12263	3.1829971	6.492516	PI4K2B	0.49025633
12264	4.9182461	10.031989	ATF7IP	0.49025633
12265	19.433076	39.643943	PYCR1	0.490190302
12266	30.310091	61.86527	PHLDA1	0.489937105
12267	1.5261151	3.115874	FAM86B1	0.489787185
12268	0.6109432	1.2473645	ZNF616	0.489787185
12269	3.8616337	7.8843094	C3orf17	0.489787185
12270	3.6173491	7.3855527	TLCD1	0.489787185
12271	2.0730539	4.2325605	RHOJ	0.489787185
12272	3.16623	6.4645015	USP6NL	0.489787185
12273	8.7222511	17.808247	SHB	0.489787185
12274	0.6578729	1.343181	KCNJ14	0.489787185
12275	29.993366	61.245897	UBA2	0.489720411
12276	9.5778919	19.582841	SLC25A32	0.489096127
12277	16.347365	33.439654	MRPL44	0.488861661
12278	1.5267363	3.1234521	FUT10	0.488797716
12279	2.8002	5.7287502	PLK4	0.488797716
12280	4.1225577	8.4351205	DOCK10	0.488737266
12281	2.8418114	5.8154122	VDR	0.488668949
12282	7.6641444	15.691714	GTDC2	0.488419828
12283	1.404198	2.8758314	XRN1	0.488275496
12284	9.8089912	20.098509	NFS1	0.48804572
12285	8.7659279	17.964705	MSL3P1	0.487952776
12286	0.7282622	1.4931557	LMLN	0.48773357
12287	2.0754855	4.2553674	SLC9A7	0.48773357
12288	12.111675	24.840174	SYNJ2	0.487584155
12289	9.4162634	19.314168	PUS1	0.487531401
12290	9.4727046	19.430749	CPOX	0.48751103
12291	29.516016	60.548449	SF3B1	0.48747766
12292	1.9830402	4.0681515	ZNF518B	0.487454865
12293	50.584242	103.88875	SPCS1	0.486907778
12294	22.06447	45.322197	USP7	0.486835852
12295	0.7779729	1.5988397	AMDHD1	0.486585962
12296	1.2160438	2.4991345	LOC100288069	0.486585962

12297	0.9726931	1.999016	FAM86EP	0.486585961
12298	49.324387	101.38746	AURKB	0.486493945
12299	6.5446078	13.454197	NOM1	0.486436151
12300	11.926817	24.524869	PHRF1	0.486315235
12301	6.4965614	13.366712	SLC30A9	0.486025379
12302	2.1541184	4.4325456	GABBR1	0.485977729
12303	4.0642226	8.3629812	HOOK3	0.485977729
12304	6.5958091	13.572246	GSTZ1	0.485977729
12305	13.735595	28.265407	SPCS2	0.485950732
12306	22.902237	47.132804	SRPX	0.485908643
12307	64.061581	131.88016	COX5A	0.485756015
12308	2080.5753	4284.059	MTRNR2L2	0.485655149
12309	7.9897572	16.455806	VPS45	0.485528166
12310	9.3313209	19.22174	HAUS2	0.485456618
12311	15.248744	31.431335	POLR1C	0.485144653
12312	1.4954202	3.0835238	PAPOLG	0.484971185
12313	2.5686052	5.2995325	GSC	0.484685235
12314	3.1768216	6.5544015	C11orf1	0.484685235
12315	0.6954377	1.4348234	PPP1R3E	0.484685235
12316	29.870773	61.660284	SRP72	0.484441061
12317	9.97568	20.59258	RFC5	0.484430807
12318	12.437082	25.673597	PNO1	0.484430807
12319	1.2182995	2.5150996	GPR180	0.484394133
12320	47.686456	98.448905	HNRNPA3	0.484377716
12321	5.5208459	11.4002	PDE4DIP	0.484276218
12322	3.6718007	7.5820381	FAM118B	0.484276218
12323	5.024753	10.378026	VPS41	0.484172341
12324	10.641904	21.980455	STC1	0.484153032
12325	0.9406625	1.9435266	SCAPER	0.483997739
12326	2.3966487	4.9517767	PFN1P2	0.483997738
12327	1.6732807	3.4572076	LOC100131067	0.483997738
12328	18.017873	37.230366	SRPRB	0.483956385
12329	3.38766	7.0044655	PDSS2	0.483642901
12330	3.12191	6.4565916	GALNT7	0.483522921
12331	2.209182	4.5698422	HEATR5B	0.483426313
12332	3.2505194	6.7300687	ST7L	0.482984585
12333	118.58366	245.54545	CLTA	0.482939772
12334	17.659963	36.582117	RNF4	0.482748533
12335	6.4165029	13.293394	RBBP6	0.482683585
12336	10.048981	20.821526	GSK3B	0.48262461
12337	0.3695963	0.7659533	SLC25A21	0.482531079
12338	0.5966448	1.2364899	ZNRD1-AS1	0.482531079
12339	0.6134552	1.2713279	SLC27A5	0.482531079

12340	0.3768526	0.7809914	SWT1	0.482531078
12341	0.9200861	1.9067914	IFT88	0.482531078
12342	30.893176	64.094719	XPO1	0.481992539
12343	2.1144221	4.3890647	TBCEL	0.481747749
12344	3.7719367	7.8323092	C11orf57	0.481586791
12345	10.216684	21.223523	USO1	0.481384924
12346	993.15387	2064.2908	RPLP1	0.481111406
12347	31.65562	65.821955	SIVA1	0.480927985
12348	0.978861	2.0353587	ARHGEF4	0.480927985
12349	3.7872537	7.876508	DNAJC21	0.480829029
12350	1.506805	3.1371677	ZBTB10	0.480307433
12351	4.0302885	8.39106	PAXIP1	0.480307433
12352	4.9578224	10.333667	DHODH	0.479773758
12353	22.07858	46.029575	CKAP5	0.479660738
12354	6.692441	13.960839	ZDHHC20	0.479372423
12355	4.6177977	9.6349299	POMT2	0.47927673
12356	18.957213	39.55612	FTSJ2	0.479248554
12357	3.4276234	7.1532737	ZNF644	0.479168493
12358	1.4593085	3.0455019	ZNF236	0.479168493
12359	3.9635541	8.2717335	PLEKHA1	0.479168493
12360	1.9149896	3.998032	SREK1IP1	0.478983056
12361	9.7620947	20.387393	MRPS9	0.478829977
12362	2.9602202	6.1838544	HERC5	0.478701467
12363	1.4671888	3.0649349	CYB5RL	0.478701467
12364	37.306083	77.935389	CIRH1A	0.478679633
12365	19.391908	40.53888	SLC35C2	0.47835332
12366	2.2138333	4.6292931	R3HCC1L	0.478222765
12367	5.5384292	11.584641	COQ6	0.478083788
12368	8.6612176	18.120502	NUP54	0.477978899
12369	7.6582499	16.023139	SMCR8	0.477949428
12370	0.8992904	1.8820257	INO80D	0.4778311
12371	23.806174	49.863701	ARFGAP1	0.47742493
12372	0.3784495	0.7930151	CERKL	0.47722854
12373	0.6773548	1.419351	CLDN4	0.47722854
12374	1.2222845	2.5612141	DLGAP1-AS1	0.477228539
12375	0.6647512	1.392941	FAM72A	0.477228539
12376	0.2709419	0.5677404	NOX4	0.477228539
12377	4.0131953	8.4093783	MAP4K3	0.477228539
12378	4.6311	9.7041556	SCARNA12	0.477228539
12379	1.9411796	4.0676101	SUPT3H	0.477228539
12380	1.7685955	3.7059717	LOC728752	0.477228539
12381	1.1914216	2.4965431	PDIA3P	0.477228539
12382	0.379368	0.7949399	LOC727849	0.477228539

12383	18.787448	39.385327	MRPS35	0.477016438
12384	9.8936337	20.744195	CWF19L1	0.476935041
12385	5.7449452	12.048547	CHD1	0.476816425
12386	3.6511403	7.6583596	DNMT3A	0.476752263
12387	4.6097118	9.6756684	GLI3	0.47642309
12388	24.650719	51.762362	TOMM20	0.476228632
12389	4.8561801	10.19816	ZNF710	0.476181986
12390	3.5434559	7.44292	IMPACT	0.476084106
12391	1.772192	3.7259277	PAPD5	0.475637778
12392	17.30393	36.380478	TIMM9	0.475637778
12393	1.7838439	3.7504251	ZNF277	0.475637777
12394	4.6507859	9.7811035	ARID3A	0.475486829
12395	3.1890042	6.707114	APC	0.475465922
12396	1.3377164	2.8138751	AKAP9	0.475400077
12397	3.4631393	7.2896093	GPR137B	0.475078861
12398	13.718174	28.886643	PSPH	0.474896804
12399	6.7788565	14.280363	ZBTB2	0.474697782
12400	6.9828819	14.717102	EPB41L1	0.474473973
12401	4.4324198	9.3427924	MAP3K4	0.474421313
12402	6.5334248	13.771356	MPHOSPH10	0.474421312
12403	6.8575025	14.457858	GPSM2	0.47430971
12404	115.76647	244.0851	RPL22	0.47428732
12405	11.978642	25.263269	THOC6	0.474152481
12406	1.3969497	2.9476833	MORN1	0.473914452
12407	0.955961	2.0171594	LOC100134229	0.473914452
12408	10.65301	22.478761	TTC7B	0.473914452
12409	1.6356721	3.451408	BORA	0.473914452
12410	18.607098	39.266762	RALBP1	0.473863826
12411	8.7263703	18.433577	RNMTL1	0.473395378
12412	8.2250376	17.389167	APTX	0.47299779
12413	5.1928792	10.978654	LOX	0.47299779
12414	4.8687011	10.294359	GTF3C3	0.472948463
12415	4.4164912	9.3398833	TYW3	0.472863644
12416	6.94665	14.693176	NUP133	0.472780686
12417	12.36734	26.161731	SSFA2	0.472726383
12418	6.3560656	13.446768	TBC1D5	0.472683506
12419	3.118197	6.5967967	GALK2	0.472683506
12420	58.184022	123.09298	UBE2C	0.472683506
12421	2.4145948	5.109367	CHD9	0.472581984
12422	25.93457	54.894564	CLN6	0.472443321
12423	9.5085085	20.133019	MMGT1	0.47228428
12424	9.4478418	20.005704	FAM49B	0.472257409
12425	9.9352782	21.045625	TTL	0.472082822

12426	9.1933361	19.480461	RIOK3	0.471926
12427	14.571929	30.882875	SQLE	0.471844957
12428	8.3706771	17.742922	METTL21A	0.471775573
12429	25.630743	54.363864	FAF1	0.471466547
12430	60.967565	129.33842	DDX24	0.47138015
12431	1.4292886	3.0321358	RUFY2	0.47138015
12432	2.0605279	4.3730513	EPC2	0.471187672
12433	5.9448352	12.616704	PUSL1	0.471187672
12434	5.550592	11.781939	GOLGA5	0.471110225
12435	27.815128	59.043449	UBL7	0.47109593
12436	27.650057	58.716993	CSNK2A1	0.470903824
12437	11.374571	24.159364	RBM12	0.470814177
12438	20.723433	44.028224	CHRAC1	0.470685186
12439	14.515388	30.861666	CECR5	0.470337152
12440	20.670716	43.957071	RRS1	0.470247803
12441	11.810976	25.117734	POLR3A	0.470224582
12442	60.840541	129.39532	EBNA1BP2	0.470191219
12443	0.189282	0.4027296	ARHGEF5	0.469997805
12444	0.4367131	0.9291812	KIAA1984-AS1	0.469997804
12445	6.2327202	13.261169	RPUSD2	0.469997804
12446	0.7281604	1.5492846	ADRB1	0.469997804
12447	1.7761321	3.7790221	WDR67	0.469997804
12448	3.6950266	7.8617955	MLF1	0.469997804
12449	13.873828	29.518921	EXOSC8	0.469997804
12450	97.838081	208.2156	RPS23	0.469888343
12451	11.392448	24.256122	NUS1	0.469673107
12452	6.7975064	14.47949	HDAC8	0.469457576
12453	5.2579485	11.20092	USP8	0.469421119
12454	18.889564	40.25016	VTI1B	0.46930408
12455	1.4314046	3.0518485	GPAM	0.469028736
12456	1.5188817	3.2403191	UVSSA	0.468744476
12457	2.7601664	5.888819	MYCBP2	0.468713072
12458	2.4393887	5.2064832	ADAMTS12	0.468529061
12459	8.4185774	17.973398	RNF113A	0.468390974
12460	106.47164	227.43909	CDC20	0.468132538
12461	4.1597732	8.890356	GPR56	0.467897255
12462	11.717456	25.044713	RPRD1B	0.46786145
12463	32.213172	68.878076	S100A13	0.467683969
12464	4.477452	9.57367	ZNF638	0.467683968
12465	6.8756341	14.704002	KIAA1429	0.467602911
12466	21.408926	45.793611	CNOT1	0.467509017
12467	324.232	693.68843	RPL9	0.467402923
12468	0.01	0.021398	ZNF831	0.467333199

12469	5.1934128	11.113985	SMO	0.467286278
12470	0.7570298	1.6211685	KCNG3	0.46696556
12471	0.6786678	1.4533574	DGKH	0.46696556
12472	28.82851	61.7869	ETF1	0.466579638
12473	4.1107963	8.8155576	CXCL3	0.466311547
12474	2.090357	4.4827476	TRMT10B	0.466311547
12475	2.401397	5.1497696	OXNAD1	0.466311547
12476	6.4654329	13.876222	PRIM2	0.465936094
12477	6.3410518	13.616735	ZHX3	0.465680787
12478	16.153028	34.688125	PWP2	0.465664491
12479	822.58965	1767.6737	RPS18	0.465351512
12480	0.3981311	0.8556478	SYDE2	0.465297827
12481	0.3559005	0.7648875	LOC399744	0.465297827
12482	0.5039214	1.0830083	TRMT10A	0.465297826
12483	0.9713079	2.0874971	C16orf46	0.465297826
12484	1.1177566	2.4022389	ZNF587B	0.465297826
12485	0.867528	1.8644574	LOC100132077	0.465297826
12486	15.304338	32.908984	GAR1	0.465050458
12487	25.353567	54.53777	PIGU	0.464880892
12488	268.03794	576.64718	MTRNR2L1	0.464821384
12489	2.9134799	6.2707458	KIAA1147	0.464614569
12490	1.8934304	4.0758504	ETV1	0.464548554
12491	5.1582171	11.106069	AGPAT5	0.464450289
12492	3.9773761	8.5641505	SOS1	0.464421559
12493	6.4108732	13.805258	BBX	0.46437909
12494	0.9529805	2.0527634	GLCCI1	0.464242729
12495	379.89871	818.41492	PABPC1	0.464188389
12496	6.9793017	15.039117	PANK3	0.464076572
12497	2.5706063	5.5391856	ZNF276	0.464076572
12498	16.938876	36.514689	CDH11	0.463892092
12499	9.3425169	20.14819	PPFIBP1	0.463690141
12500	13.013633	28.065845	SPCS3	0.463682208
12501	756.96806	1632.6196	RPL29	0.46365244
12502	0.6025116	1.2998749	ERCC6	0.463515076
12503	2.6262781	5.6660037	MAGI3	0.463515075
12504	29.991072	64.703552	CTNNBL1	0.463515075
12505	0.3508863	0.7570117	ZNF850	0.463515075
12506	5.8519346	12.636382	ANGEL1	0.463102083
12507	17.179312	37.11089	MTFP1	0.462918339
12508	11.460884	24.763797	SLC19A1	0.462808036
12509	72.347599	156.34664	GAS5	0.462738432
12510	12.632877	27.302461	GLS	0.462701034
12511	2.9172643	6.3049931	HERC1	0.462691115

12512	11.912677	25.747376	AKAP1	0.462675374
12513	1.8356433	3.9682996	LENG9	0.462576786
12514	4.4354197	9.5885048	UGDH	0.462576786
12515	4.9150826	10.630525	SPATC1L	0.462355563
12516	1.9731242	4.2708516	AGTPBP1	0.461997841
12517	8.163907	17.677041	UBR5	0.461836754
12518	31.767105	68.788263	SMARCD1	0.461809969
12519	2.0999731	4.5475199	C10orf12	0.46178425
12520	30.14584	65.281221	RRP9	0.46178425
12521	5.0694749	10.978016	CSGALNACT2	0.46178425
12522	20.468529	44.354582	ARPP19	0.461474956
12523	15.471679	33.548187	AP2A2	0.461177794
12524	8.33598	18.078231	NUTM2A-AS1	0.461105953
12525	24.272581	52.644131	PIAS3	0.461069086
12526	3.8157655	8.2795477	GDF11	0.460866418
12527	3.1948037	6.9321685	UBXN2B	0.460866418
12528	25.067162	54.411006	HIF1A	0.460700207
12529	2.1256402	4.614959	LNX2	0.460597848
12530	21.88162	47.519926	CBFB	0.460472515
12531	6.7718551	14.710541	VPRBP	0.460340315
12532	6.6343786	14.412396	ZNF142	0.460324471
12533	3.6943088	8.0266748	QRSL1	0.460253947
12534	17.694297	38.450428	LPCAT4	0.460184663
12535	37.36553	81.215214	ALG3	0.460080419
12536	15.173074	33.005277	UBAP2	0.459716602
12537	0.5678461	1.2356476	CHRM4	0.459553409
12538	0.8289009	1.8037096	LOC145783	0.459553408
12539	0.4183679	0.9103792	TMPPE	0.459553408
12540	0.3457478	0.7523561	ZNF165	0.459553408
12541	0.5670735	1.2339664	TEX9	0.459553408
12542	0.8683313	1.8895111	LIPT2	0.459553408
12543	1.2604808	2.7428386	LOC100505812	0.459553408
12544	1.2231812	2.6616737	IQCD	0.459553408
12545	1.3350384	2.9050779	PAX9	0.459553408
12546	0.4987125	1.0852113	IQCA1	0.459553408
12547	0.7449491	1.6210283	INPP5J	0.459553408
12548	1.4931387	3.2491081	SASS6	0.459553408
12549	1.4701905	3.1991722	FAM175A	0.459553408
12550	0.6575845	1.4309208	PIK3C2B	0.459553408
12551	0.4104372	0.8931219	DDX11-AS1	0.459553408
12552	0.7318683	1.5925642	LOC284751	0.459553408
12553	0.5174413	1.1259656	NBPF7	0.459553408
12554	0.3782205	0.8230175	LOC151009	0.459553407

12555	7.1734227	15.620741	EAF1	0.459224215
12556	5.838232	12.716417	GRPEL2	0.459109824
12557	3.9854775	8.6815069	LRCH1	0.459076693
12558	6.1149263	13.32323	RNF13	0.458967243
12559	10.623986	23.155294	RFK	0.458814576
12560	34.941885	76.19235	MAP4K4	0.458600966
12561	1.5630871	3.4101066	ZNF280B	0.458368992
12562	7.2413776	15.808172	SEL1L	0.458078116
12563	2.6156474	5.713033	MOB1B	0.457838657
12564	0.01	0.0218456	COL6A5	0.457757309
12565	2.7745496	6.0623366	SERTAD4	0.457669993
12566	4.5695863	9.9860308	KLHL18	0.457597862
12567	8.1977435	17.916228	EMP2	0.457559684
12568	5.4535038	11.920316	WDR36	0.457496584
12569	33.460083	73.145811	NASP	0.457443605
12570	1.4752812	3.2257581	SPIRE2	0.457344017
12571	4.7517801	10.394704	THOC3	0.457134706
12572	6.5307818	14.287377	BNC1	0.457101523
12573	16.220939	35.488491	B4GALT1	0.457076031
12574	2.7776854	6.0796615	PMS2	0.456881586
12575	8.913517	19.520718	CNOT10	0.456618304
12576	1.7383668	3.8074544	SPATA5	0.456569295
12577	1.6045251	3.514308	METTL15	0.456569295
12578	105.26446	230.58556	SET	0.456509329
12579	6.4562982	14.147868	CAMSAP1	0.456344236
12580	6.9569566	15.250653	WDYHV1	0.456174339
12581	20.814947	45.641459	SAFB2	0.456053498
12582	19.435226	42.617227	UNG	0.456041537
12583	6.6650054	14.617421	OSBPL2	0.455963147
12584	27.269562	59.843338	LAS1L	0.455682507
12585	0.9514288	2.0880286	NEK1	0.455658888
12586	4.6430051	10.189651	B9D1	0.455658888
12587	12.27204	26.949266	ZFP36L2	0.45537565
12588	0.4892007	1.0752659	TTC30A	0.454957874
12589	1.1871541	2.6093714	C3orf33	0.454957874
12590	4.1592337	9.1458687	ATG14	0.454766394
12591	29.451597	64.790018	DNAJC5	0.454569974
12592	36.572222	80.458822	SRSF6	0.454545821
12593	1.2420028	2.7336946	PEAR1	0.45433121
12594	2.2267197	4.9037774	MYSM1	0.454082534
12595	2.3859879	5.2551336	PHIP	0.45402993
12596	1.0449	2.3017999	LLGL2	0.453949098
12597	3.9183115	8.6365059	MRPS25	0.453691758

12598	10.279006	22.665612	REPS1	0.453506653
12599	9.8736339	21.771751	FANCI	0.453506653
12600	1.8471858	4.0731173	CEP152	0.453506653
12601	7.730598	17.058909	E2F7	0.453170722
12602	14.085928	31.087969	TAF13	0.453099006
12603	5.1351006	11.339653	MOAP1	0.452844599
12604	3.3696009	7.4487243	APPL1	0.452372886
12605	1.777936	3.9302444	HOXC5	0.452372886
12606	16.632824	36.784848	TSN	0.45216509
12607	11.355775	25.117214	WRAP53	0.452111248
12608	19.197868	42.504876	RABGGTB	0.451662725
12609	13.737607	30.424823	CCNH	0.451526274
12610	17.378694	38.516788	TRUB2	0.451197892
12611	0.01	0.0221774	ZNF208	0.450910298
12612	2.4636937	5.4661794	COMMD8	0.450715843
12613	0.8565743	1.9004753	PYROXD1	0.450715843
12614	2.3665942	5.2507455	NAT6	0.450715843
12615	6.2407312	13.852086	HACL1	0.450526466
12616	4.7661963	10.583026	CUL2	0.45036234
12617	5.5159061	12.248764	LRP8	0.450323477
12618	10.080627	22.394205	UTP3	0.450144448
12619	6.4500951	14.332621	TAF9B	0.450028985
12620	12.220557	27.158038	MTX2	0.449979379
12621	0.4834134	1.0752959	ZNF845	0.449563117
12622	2.519184	5.6036271	FANCF	0.449563117
12623	1.670671	3.7162101	KIF24	0.449563117
12624	0.4410571	0.9810795	KCNMB4	0.449563116
12625	67.58003	150.35598	MRPL36	0.449466851
12626	4.0125054	8.9334284	INPP5F	0.449156272
12627	6.517061	14.517677	CSTF3	0.44890522
12628	12.824585	28.580698	PPP4R2	0.448714885
12629	7.7434386	17.256924	AK3	0.448714884
12630	0.2109307	0.4701738	LEKR1	0.448622735
12631	2.032615	4.5322328	METTL4	0.448479832
12632	45.118355	100.64196	TBRG4	0.448305597
12633	26.022603	58.053394	RAP1A	0.448252914
12634	1.4329808	3.1981569	RALGAPA2	0.448064573
12635	0.6609401	1.4751001	BHLHB9	0.448064573
12636	2.370248	5.2899697	SEC22A	0.448064573
12637	6.8065344	15.19814	URB1	0.447853122
12638	5.8553085	13.080272	RPUSD4	0.44764425
12639	0.7639471	1.7069531	MLH3	0.447550148
12640	17.423705	38.959845	LARS	0.44722214

12641	1.0670054	2.3863265	ZNF92	0.447133046
12642	10.00993	22.394205	TGIF2	0.446987495
12643	4.973995	11.130778	FAM53B	0.446868574
12644	13.956649	31.245947	BUB1	0.446670707
12645	3.1685173	7.0963766	PDS5B	0.446497914
12646	10.113897	22.65161	ADA	0.446497914
12647	1.1679503	2.6185047	PLEKHA8	0.446037132
12648	16.173243	36.270864	UBP1	0.445901788
12649	12.335991	27.668407	NGDN	0.445851128
12650	19.585144	43.950222	FIP1L1	0.445621052
12651	13.77678	30.923227	CSTF2	0.445515603
12652	2.0517532	4.606403	HCG11	0.445413303
12653	3.933263	8.8349741	DDX31	0.445192364
12654	9.3973249	21.117082	DCPS	0.445010579
12655	5.4958953	12.350033	KIAA1143	0.445010579
12656	9.0194237	20.271499	ZFP64	0.444931254
12657	22.186723	49.878763	PPRC1	0.444813016
12658	9.107789	20.481416	PRPF40A	0.444685504
12659	2.1945139	4.9351351	MTX3	0.444671497
12660	19.651707	44.199089	FAM136A	0.444617922
12661	8.8565269	19.927541	TRIM65	0.44443652
12662	13.100191	29.50245	KLHL21	0.444037379
12663	7.0726004	15.928933	MSI2	0.44400969
12664	13.668377	30.788546	FCF1	0.443943578
12665	3.8690203	8.7167318	UBXN7	0.443861341
12666	10.492272	23.647181	IGF1R	0.443700732
12667	15.373169	34.669163	KRI1	0.443424851
12668	0.1754697	0.3959683	CD101	0.443140787
12669	0.554254	1.2507402	LOC728323	0.443140787
12670	0.742516	1.6755759	SERP2	0.443140787
12671	0.3972036	0.8963373	LINC00652	0.443140787
12672	7.1913559	16.228152	LOC152217	0.443140787
12673	1.2110383	2.7328522	ZNF713	0.443140787
12674	0.4911222	1.1082757	PARD6A	0.443140787
12675	1.5058621	3.3981572	ZNF37A	0.443140787
12676	0.5412974	1.2215021	DEPDC4	0.443140786
12677	1.8612134	4.2000499	CTAGE5	0.443140786
12678	0.5436509	1.226813	ZBTB37	0.443140786
12679	0.1868495	0.4216482	DUXA	0.443140786
12680	1.5315985	3.4562345	ZFP69B	0.443140786
12681	0.1549055	0.3495627	CABP4	0.443140786
12682	0.2789819	0.629556	NKX3-2	0.443140786
12683	0.3858059	0.8706171	HNF1A	0.443140786

12684	0.3011553	0.6795929	FLJ44313	0.443140786
12685	0.4159671	0.9386793	ELMOD1	0.443140786
12686	0.2435522	0.5496046	LINC00649	0.443140786
12687	0.1008222	0.2275173	SGSM1	0.443140785
12688	101.29994	228.72004	ANP32B	0.442899293
12689	26.706424	60.302251	COPS3	0.442876067
12690	5.8379013	13.195804	DHX32	0.442405893
12691	5.65425	12.783162	AHRR	0.442320156
12692	2.4841149	5.6200017	C10orf137	0.442013202
12693	1.356068	3.0683773	BAZ2B	0.441949548
12694	138.3022	312.95965	PA2G4	0.441917038
12695	72.556397	164.22115	GOT2	0.44182126
12696	2.4071704	5.4485778	PALB2	0.441797936
12697	3.7759354	8.5467476	IQCB1	0.441797936
12698	11.516814	26.070663	UCK2	0.441753804
12699	3.807393	8.6235111	CLSPN	0.441513088
12700	16.730853	37.927758	DIMT1	0.441124219
12701	4.1772728	9.4714022	NAA30	0.441040593
12702	17.534581	39.763792	OCIAD1	0.440968528
12703	0.5355196	1.2151036	CLCN5	0.440719252
12704	2.0641474	4.6835881	LONRF3	0.440719252
12705	1.8794266	4.2644532	LRRC49	0.440719252
12706	29.16718	66.218834	RCC1	0.440466525
12707	5.6454739	12.817544	PREPL	0.440448967
12708	21.820152	49.542027	FAM83D	0.44043721
12709	0.4302683	0.9769824	NBEA	0.44040535
12710	2.7802718	6.3129836	CDC37L1	0.440405349
12711	0.8909272	2.022971	RAD54B	0.440405349
12712	54.14116	122.9454	TWF2	0.440367508
12713	2.8883593	6.5602512	FAM8A1	0.440281814
12714	1.4132122	3.2102717	PRDM10	0.440215765
12715	13.050863	29.651174	EXOSC5	0.440146592
12716	2.1686767	4.927169	CEP63	0.440146592
12717	1.4269052	3.2429815	DCAF17	0.439997944
12718	2.7602583	6.2733436	FASTKD1	0.439997944
12719	32.312982	73.476142	MCFD2	0.43977516
12720	736.668	1675.3246	RPL26	0.43971657
12721	430.66511	979.73552	RPL35A	0.439572825
12722	1.7512563	3.985128	MNS1	0.439447947
12723	17.280469	39.328732	TCERG1	0.439385356
12724	2.5260545	5.7528809	RBM12B	0.439093839
12725	8.119461	18.503049	SCFD1	0.438817462
12726	2.4517588	5.589142	DUS4L	0.438664617

12727	1.7793818	4.0563604	ABTB2	0.438664617
12728	2.6143306	5.9597482	NKX6-1	0.438664617
12729	0.01	0.0228099	AHCTF1P1	0.438407072
12730	1.0392953	2.3720971	FAM63B	0.438133546
12731	4.4056925	10.057574	POLD3	0.438047214
12732	27.464939	62.72571	IGFBP4	0.437857754
12733	6.4858157	14.81484	PRKCI	0.437791826
12734	2.3325301	5.3284552	GABPA	0.437749779
12735	2.1495565	4.9113645	LTN1	0.437669912
12736	22.238845	50.820585	GLUD1	0.437595225
12737	0.7838586	1.7918427	PAPLN	0.437459494
12738	4.3416563	9.9247046	WDR53	0.437459494
12739	2.7869175	6.3745764	ZNF473	0.437192588
12740	3.405492	7.7920053	DTNB	0.437049504
12741	1.6143911	3.6957685	DCP2	0.436821488
12742	2.5348332	5.8035374	USP46	0.436773821
12743	8.0993925	18.55209	CDK6	0.436575738
12744	10.527196	24.132951	SENP2	0.436216712
12745	11.794562	27.04918	TCF12	0.436041373
12746	17.609979	40.395152	PTGES2	0.435942878
12747	8.0681849	18.51119	FOXA2	0.435854466
12748	6.3566833	14.585699	NPRL2	0.435816145
12749	33.389975	76.639501	TMED10	0.435675791
12750	0.8602663	1.9759593	ZSCAN5A	0.435366387
12751	0.5878688	1.3502853	FNTB	0.435366387
12752	5.6181837	12.904496	PTPLB	0.435366387
12753	0.6631647	1.5232335	KAZALD1	0.435366386
12754	0.3971405	0.9121985	ZNF670	0.435366386
12755	37.077654	85.181697	DDX56	0.435277245
12756	3.9967027	9.1837231	SREK1	0.435194169
12757	26.21925	60.258502	SNX5	0.435112874
12758	34.009641	78.17977	ARCN1	0.435018428
12759	7.7318538	17.783634	DHTKD1	0.434773568
12760	3.2330321	7.4365663	FAM117A	0.434747969
12761	5.368497	12.353366	USP12	0.434577679
12762	15.34725	35.327293	DARS	0.434430402
12763	227.85511	525.03757	RPL36	0.433978675
12764	9.2526918	21.336709	TRAF3	0.433651307
12765	2.3033353	5.3158814	RDH10	0.433293213
12766	11.15629	25.750938	NMRAL1	0.433238199
12767	18.867945	43.558822	HEATR2	0.433160138
12768	4.9867885	11.512575	SP110	0.433160138
12769	18.652839	43.066322	PLAT	0.43311892

12770	3.177217	7.3369938	PHACTR2	0.433040712
12771	0.7897244	1.8245384	PALM2	0.432835187
12772	1.5962515	3.6878968	ZNF804A	0.432835187
12773	3.7709887	8.712297	LINC00667	0.432835187
12774	37.76896	87.314523	SCRN1	0.432562171
12775	89.953477	207.98809	C1orf43	0.432493411
12776	149.93658	346.70407	SLC25A5	0.432462695
12777	14.359683	33.205542	THEM6	0.432448381
12778	2.639421	6.1041474	TSPAN7	0.432397979
12779	2.7051093	6.2582941	PCMTD2	0.432243882
12780	20.372838	47.24373	MMS19	0.4312284
12781	0.01	0.0231957	ZAN	0.431113514
12782	4.2996673	9.9734347	NUDT16	0.431111992
12783	3.4919487	8.1051413	MAP3K14	0.43083132
12784	0.740581	1.7189582	SLX4IP	0.43083132
12785	1.0255881	2.3804864	UFSP1	0.43083132
12786	3.9474478	9.1706968	BTBD3	0.430441428
12787	19.285589	44.819342	GPATCH4	0.430296126
12788	1.6192317	3.7668539	NFATC1	0.42986316
12789	2.0425066	4.7531958	EIF3C	0.429712278
12790	1.4732612	3.4325308	RPAP2	0.429205542
12791	1.683109	3.9214522	IER5L	0.429205542
12792	90.832549	211.82772	HNRNPA1	0.428803879
12793	40.785038	95.115272	BOP1	0.428795897
12794	21.701632	50.618449	SNHG8	0.428729704
12795	8.104425	18.904876	BCL9L	0.428694966
12796	15.989038	37.304261	NOA1	0.42861158
12797	2.1183844	4.9488976	CREBZF	0.428051763
12798	6.7833472	15.854125	DNAJC2	0.42786007
12799	2.4958024	5.8332211	HSD17B8	0.42786007
12800	0.9410916	2.1995312	CEP290	0.42786007
12801	6.3127892	14.757452	NCBP1	0.427769575
12802	21.42686	50.094145	KCNG1	0.427731814
12803	14.056855	32.864547	NUP205	0.427720928
12804	23.275788	54.444094	MRPS22	0.427517233
12805	3.906793	9.1400024	ATRX	0.427438948
12806	17.579278	41.132214	RPL39L	0.42738467
12807	4.3948194	10.283053	NVL	0.427384669
12808	2.6679614	6.2431115	ELK4	0.427344824
12809	31.251292	73.138324	PDCD6	0.427290241
12810	6.8958886	16.144947	IGSF3	0.42712365
12811	2.2967462	5.3777268	COX16	0.427084961
12812	1.2550208	2.938574	CRMP1	0.427084961

12813	6.2086876	14.537359	MCCC1	0.427084961
12814	1.8680872	4.3776985	ZNF562	0.426728165
12815	1.6195803	3.7983566	ARHGAP39	0.42638976
12816	48.564288	113.93518	MARCKSL1	0.426244894
12817	42.493607	99.735581	STAU1	0.426062667
12818	15.830049	37.162122	ADNP	0.425972693
12819	0.2603599	0.6115141	PTGIS	0.425762717
12820	0.7236094	1.6995602	ZNF57	0.425762717
12821	0.6603877	1.5510699	ZNF675	0.425762717
12822	0.5956703	1.3990663	TPK1	0.425762717
12823	1.7373519	4.0805638	LOC440894	0.425762716
12824	3.7025292	8.6962269	BDH1	0.425762716
12825	1.0151681	2.3843517	ZBED3	0.425762716
12826	6.6199511	15.548452	SWAP70	0.425762716
12827	259.90808	610.48919	RPL37	0.425737395
12828	4.617974	10.853775	LOC100506548	0.425471696
12829	1.7644809	4.150871	C3orf58	0.425086903
12830	26.900515	63.287377	YTHDF1	0.425053407
12831	9.4760961	22.29799	GPANK1	0.42497536
12832	22.14745	52.128893	TSG101	0.424859401
12833	2.9200837	6.873309	TACC2	0.424843936
12834	24.694738	58.144912	PRSS23	0.424710219
12835	12.145836	28.600201	ZNF664	0.424676587
12836	4.3338052	10.204954	MND1	0.424676587
12837	11.815975	27.831481	ZDHHC6	0.424554308
12838	26.828265	63.192796	DDX27	0.424546251
12839	33.819529	79.668896	NUP93	0.424501042
12840	3.5476126	8.3575057	DSCC1	0.424482227
12841	25.685834	60.52411	RPL36A	0.424390118
12842	5.5293554	13.030846	RBBP8	0.424328206
12843	22.398475	52.796146	ABHD14B	0.424244508
12844	1.5878057	3.7430314	LOC646278	0.424203146
12845	4.0113561	9.4585241	POLA1	0.424099581
12846	16.661381	39.290419	TRIO	0.424057107
12847	7.3760793	17.39419	GMFB	0.424054198
12848	7.72634	18.220171	PRKD3	0.424054198
12849	8.7594306	20.657635	SETDB1	0.42402872
12850	2.6141663	6.1653061	CAPRIN2	0.424012407
12851	0.9343225	2.203913	JMY	0.423938019
12852	10.678392	25.198645	DPY19L1	0.423768512
12853	3.9599601	9.3446304	DDX11	0.423768512
12854	1.8461818	4.3577913	BEND3	0.423650798
12855	23.00202	54.307561	ANP32E	0.423550962

12856	8.9834348	21.211443	DDX50	0.423518316
12857	1.4343844	3.3880313	ALDH1L2	0.4233681
12858	1.1098198	2.6236998	LIPT1	0.422998023
12859	31.391256	74.225122	ADAM19	0.42291956
12860	4.1317116	9.7785142	HIVEP3	0.422529587
12861	22.322568	52.84388	HRAS	0.422424855
12862	3.1104403	7.3648467	TBC1D23	0.422336055
12863	9.9611353	23.591035	DCAF13	0.4222424
12864	4.2847335	10.14946	DHX35	0.422163708
12865	1.7001795	4.0284905	FAM115C	0.422038844
12866	7.1033915	16.83597	SARS2	0.421917569
12867	9.4727046	22.451553	TIMM21	0.421917569
12868	1.1579825	2.7455882	NMNAT2	0.421761187
12869	1.3136931	3.1185054	PPAN	0.421257291
12870	0.595118	1.4127186	TM4SF18	0.421257291
12871	2.1171327	5.0297939	BLM	0.420918387
12872	4.4998543	10.698446	PPWD1	0.420608204
12873	0.01	0.0237927	UNC79	0.420297229
12874	23.37524	55.623192	TRIP12	0.420242681
12875	0.8020111	1.909275	C8orf37	0.420060537
12876	2.1050455	5.0112907	PRR5	0.420060537
12877	12.734165	30.31507	C9orf142	0.420060537
12878	9.5992946	22.865394	SORT1	0.419817587
12879	8.4251879	20.070906	NOL9	0.419771173
12880	6.6912822	15.943757	ACAT1	0.419680392
12881	3.4143862	8.1373911	LYSMD2	0.419592242
12882	14.810117	35.29645	C14orf1	0.419592242
12883	11.125128	26.518024	UBR7	0.419530827
12884	12.078059	28.79255	ANGPTL4	0.419485584
12885	3.3318361	7.9453816	SS18L1	0.419342485
12886	0.3220017	0.7681572	LAMA2	0.419187231
12887	2.2107443	5.2738828	ZNF800	0.41918723
12888	2.0219874	4.8235901	ZNF507	0.41918723
12889	6.3884235	15.247379	HECTD1	0.418985018
12890	5.2427547	12.517516	BRI3BP	0.418833486
12891	51.867494	123.87731	LAPTM4B	0.418700511
12892	7.4850482	17.906898	MIS18A	0.417998047
12893	4.7846824	11.451582	TADA2B	0.417818456
12894	2.0979815	5.0242033	ZMYM1	0.417574972
12895	26.908727	64.451015	RRP1	0.41750664
12896	15.929739	38.17643	PRKAR2A	0.417266343
12897	4.8100236	11.528577	CCDC106	0.417226121
12898	7.2816754	17.4569	ILF3-AS1	0.417123051

12899	0.572652	1.373485	TMEM135	0.416933536
12900	6.1514143	14.762758	GTF2A1	0.41668462
12901	27.740936	66.575378	GGCT	0.41668462
12902	13.88215	33.321715	EXOSC7	0.416609704
12903	7.5707895	18.173954	PARN	0.416573593
12904	14.446017	34.684696	POGZ	0.416495427
12905	4.8996097	11.765993	STIL	0.416421262
12906	2.8273018	6.7917655	MAPKAPK5-AS1	0.416283769
12907	3.7531664	9.017117	SMC6	0.416226869
12908	2.6507673	6.3693983	FAM199X	0.416172329
12909	3.8082003	9.1525205	REST	0.41608214
12910	1.9828203	4.7694904	ROR2	0.415730016
12911	2.7504503	6.615953	ZNF143	0.415730016
12912	15.249551	36.694505	PTPN11	0.415581319
12913	15.768088	37.944069	SLC5A6	0.415561349
12914	2.6225281	6.3112729	FAM35A	0.415530769
12915	63.792303	153.52641	TPX2	0.415513555
12916	265.04057	638.02191	BTF3	0.415409824
12917	9.9547009	23.963893	LPXN	0.415404173
12918	2.0817803	5.0119231	IKZF5	0.41536558
12919	4.6412116	11.177544	HOXC8	0.415226406
12920	12.678297	30.551486	PPP2R2A	0.414981339
12921	2.9602202	7.1352166	PDK1	0.414874605
12922	4.6771357	11.277426	NAV3	0.414734326
12923	10.895848	26.28057	IKBIP	0.414597096
12924	11.336488	27.348387	CHD8	0.414521277
12925	28.389564	68.518979	MRGBP	0.414331397
12926	7.9666541	19.24207	KIF11	0.414022706
12927	0.0454079	0.1097875	F5	0.413598072
12928	0.0478804	0.1157656	SPTBN4	0.413598071
12929	0.0592718	0.1433076	REXO1L1	0.41359807
12930	0.0845434	0.2044096	SLC26A4	0.413598069
12931	0.1466053	0.3544633	LILRB3	0.413598069
12932	0.1092527	0.2641518	C9orf139	0.413598068
12933	0.1465538	0.3543387	CHIAP2	0.413598068
12934	0.2101861	0.5081892	CSF2RA	0.413598068
12935	0.3063572	0.7407124	FAM149A	0.413598068
12936	0.2735799	0.6614632	ENOX1	0.413598068
12937	0.2506308	0.6059767	ACP5	0.413598068
12938	0.12924	0.3124773	ARSI	0.413598068
12939	0.8269821	1.9994826	KTN1-AS1	0.413598068
12940	10.329591	24.97495	HOXB2	0.413598068
12941	1.3997219	3.3842563	RBAK	0.413598068

12942	0.1481689	0.3582436	FSIP1	0.413598068
12943	0.2268911	0.5485788	CCDC11	0.413598067
12944	0.9090491	2.1979045	SENP8	0.413598067
12945	1.8817111	4.5496128	OPHN1	0.413598067
12946	1.5008194	3.6286907	COX10-AS1	0.413598067
12947	6.6955663	16.188582	MSMO1	0.413598067
12948	0.5784055	1.3984724	KIF9	0.413598067
12949	7.4663678	18.052231	ELP2	0.413598067
12950	1.7105294	4.1357287	C9orf116	0.413598067
12951	0.5640041	1.3636526	TMEM161B-AS1	0.413598067
12952	0.6365773	1.5391206	TBXA2R	0.413598067
12953	1.7936482	4.3366939	FAM86C2P	0.413598067
12954	8.2849157	20.031321	PDPR	0.413598067
12955	0.74645	1.8047714	CRIPAK	0.413598067
12956	0.094128	0.2275834	CD209	0.413598067
12957	0.65329	1.5795286	HERC2P10	0.413598067
12958	0.2675218	0.6468159	LOC339666	0.413598067
12959	0.1332904	0.3222703	LRRC4C	0.413598067
12960	0.1624947	0.3928808	ACADL	0.413598067
12961	0.7785785	1.8824519	C10orf25	0.413598067
12962	0.2987806	0.7223937	FANK1	0.413598067
12963	0.326773	0.7900739	C3orf67	0.413598067
12964	0.0960809	0.232305	MAP6	0.413598067
12965	0.2262138	0.5469412	CCDC150	0.413598067
12966	0.1247154	0.3015378	LANCL3	0.413598067
12967	0.1743929	0.4216482	SAMD3	0.413598067
12968	0.1200804	0.2903311	GOLGA8T	0.413598067
12969	0.1423494	0.3441732	POU3F1	0.413598067
12970	0.1608641	0.3889383	LOC286297	0.413598067
12971	0.0822738	0.1989221	NPC1L1	0.413598066
12972	0.1820878	0.4402531	PCDHGB5	0.413598066
12973	0.1610506	0.3893892	ZNF799	0.413598066
12974	0.1415757	0.3423027	NIM1	0.413598066
12975	0.1329078	0.3213454	COLQ	0.413598065
12976	31.276005	75.6582	EIF2S2	0.41338553
12977	10.250116	24.811118	CHID1	0.413125923
12978	93.628761	226.93018	SF3B4	0.412588396
12979	6.4747152	15.696353	GINS3	0.412498072
12980	7.4321686	18.023723	ATAD2	0.412354787
12981	11.431196	27.744719	DENND4B	0.4120134
12982	14.554886	35.333079	METAP1	0.411933689
12983	1.8339755	4.4522962	DZIP3	0.411916774
12984	10.414819	25.297363	ELL2	0.411695842

12985	9.461677	22.993817	ZNRD1	0.411487873
12986	14.805314	35.981855	YLPM1	0.411466118
12987	3.5476569	8.6265616	SPIN4	0.411248078
12988	2.8561368	6.9532102	GID4	0.410765204
12989	2.5201248	6.1361843	SOCS4	0.410699016
12990	17.557707	42.767934	SERP1	0.410534378
12991	2.7910647	6.8044893	LONRF1	0.410179901
12992	9.9596548	24.282873	SUPV3L1	0.410151417
12993	23.496022	57.302819	NUDT5	0.410032567
12994	10.787862	26.31087	GIGYF2	0.410015408
12995	124.01087	302.58829	CCT5	0.409833669
12996	1.2539798	3.0607553	GPR126	0.409696199
12997	0.6125965	1.4952458	FANCM	0.409696199
12998	6.5668787	16.030476	NR1D2	0.409649637
12999	2.5917512	6.3303881	SETX	0.409414272
13000	5.2697927	12.875457	SLAIN2	0.409289754
13001	2.2294992	5.4472391	ABHD16B	0.409289754
13002	4.0155479	9.8166926	PPP2R1B	0.409053034
13003	10.128577	24.761037	PROSER1	0.409053033
13004	0.3490091	0.8537639	APC2	0.408788788
13005	0.8559535	2.0938771	GABPB1-AS1	0.408788788
13006	9.2041219	22.528522	RPIA	0.408554188
13007	20.875332	51.09985	C20orf27	0.408520413
13008	18.539114	45.464328	NUP88	0.407772743
13009	2.8209012	6.9253217	NBPF15	0.40733143
13010	6.7939764	16.684913	ZBTB9	0.407192788
13011	6.1959266	15.218337	NHEJ1	0.407135598
13012	7.6392779	18.765819	TCF7L2	0.407084712
13013	6.9780828	17.145247	SLMAP	0.406998098
13014	4.16799	10.243808	HMGCS1	0.406878965
13015	5.6823066	13.966237	METTL16	0.406860254
13016	6.7289176	16.53905	C2CD2	0.406850306
13017	1.801076	4.4272303	FBXL4	0.406817771
13018	4.8522378	11.930614	NFRKB	0.406704766
13019	2.5846855	6.3563991	STRN	0.406627313
13020	5.9923179	14.738678	ASB13	0.406570916
13021	22.178898	54.5776	GTPBP4	0.406373647
13022	6.4093043	15.77821	IPO8	0.406212388
13023	9.6574091	23.783565	USP1	0.406053897
13024	2.2204898	5.4739831	ZNF148	0.405644258
13025	11.923073	29.401503	DCAF12	0.405525986
13026	2.4186327	5.9699778	CPS1	0.40513261
13027	7.4309914	18.351698	SAV1	0.404921185

13028	30.635507	75.6748	SNRPA1	0.404831026
13029	9.1007488	22.484604	DNTTIP2	0.404754688
13030	1.752893	4.3323369	CTH	0.404606805
13031	3.8832205	9.5975165	C4orf48	0.404606805
13032	0.4187532	1.0349633	ZNF100	0.404606805
13033	1.1285754	2.7910463	BNC2	0.404355652
13034	32.269899	79.809136	C19orf48	0.404338409
13035	2.4071022	5.9542123	APBB2	0.404268788
13036	3.0045863	7.4334495	SMC5	0.404198111
13037	2.597443	6.4261632	RBM15	0.404198111
13038	15.423715	38.158801	ALG8	0.404198111
13039	42.762041	105.83794	LY6E	0.4040332
13040	4.5088267	11.160148	AGPAT9	0.404011357
13041	8.3638595	20.703673	CWC27	0.403979508
13042	0.5406795	1.3386324	RAB3IP	0.403904363
13043	10.162524	25.16072	GCSH	0.403904363
13044	4.2915161	10.628445	ACAP2	0.403776477
13045	6.0588785	15.009549	CDC5L	0.40366826
13046	8.9741412	22.240178	EFNB1	0.40351031
13047	2.7264039	6.7567144	MIPEP	0.40351031
13048	1.3702654	3.3994634	PTBP2	0.403082862
13049	10.610157	26.330939	SLTM	0.402953999
13050	8.7594501	21.739677	ARHGAP21	0.402924569
13051	3.3289714	8.262493	SYNJ2BP	0.402901566
13052	4.9009204	12.169732	PM20D2	0.402713908
13053	2.8779785	7.1494091	UTP23	0.402547737
13054	4.8477122	12.048508	SLC25A12	0.402349584
13055	3.5503121	8.8239486	FBXO28	0.402349584
13056	15.506503	38.542444	RASA3	0.402322779
13057	0.7609789	1.892468	LOC731275	0.402109232
13058	0.6547561	1.628304	LOC646329	0.402109232
13059	0.8015365	1.9933304	KITLG	0.402109232
13060	2.7182544	6.7599899	DUSP4	0.402109232
13061	8.687006	21.603597	CLPX	0.402109232
13062	0.3931006	0.9775967	NSUN7	0.402109232
13063	4.276428	10.634991	ZNF326	0.402109232
13064	7.8858149	19.622076	UBE2K	0.401884841
13065	2.737883	6.8168524	CLASP2	0.401634487
13066	4.185126	10.421592	SENP5	0.401582221
13067	8.4032057	20.926843	BATF3	0.401551522
13068	88.710598	220.95364	RPL36AL	0.401489649
13069	0.1472008	0.3668954	CNGB1	0.401206436
13070	19.255392	48.010676	USP14	0.401064793

13071	2.0568682	5.1316717	PIK3R1	0.400818352
13072	8.9881429	22.439128	MRPL48	0.400556687
13073	73.391161	183.32883	SLIRP	0.400325252
13074	1.0104218	2.5244377	MPP4	0.400256194
13075	0.9849523	2.4608048	KLF15	0.400256194
13076	15.502745	38.741594	SUB1	0.400157658
13077	41.774573	104.43635	MCM2	0.400000323
13078	2.095303	5.2429459	RLF	0.399642304
13079	0.6605988	1.6535717	NAPEPLD	0.399498133
13080	6.233015	15.613125	NUP160	0.39921636
13081	15.416237	38.628875	TMEM69	0.399085854
13082	22.372663	56.065208	MRPS30	0.399047176
13083	20.940464	52.493169	RPUSD3	0.398917889
13084	3.0399927	7.6209071	ZFYVE9	0.398901689
13085	21.906288	54.926833	TEAD4	0.398826708
13086	6.4106207	16.077592	NAF1	0.398730163
13087	24.650684	61.846397	PPIL1	0.398579143
13088	2.4006525	6.0254293	CHRNA5	0.398420157
13089	2.2301404	5.5985513	FAM210A	0.398342401
13090	6.4196628	16.123828	PGAP2	0.398147565
13091	7.5792374	19.039094	TNRC18	0.39808814
13092	8.4555958	21.241807	PRPSAP2	0.398063868
13093	9.7411618	24.479496	PPIG	0.397931474
13094	0.8830487	2.2204424	PDF	0.39769045
13095	0.6089991	1.5313396	ZNF625	0.39769045
13096	0.2727742	0.6858958	PUS10	0.39769045
13097	4.3807703	11.015528	ALDOC	0.39769045
13098	0.5513214	1.3863079	SETD5-AS1	0.39769045
13099	2.3104157	5.8095832	TERC	0.397690449
13100	40.613788	102.12412	POLR2H	0.397690449
13101	0.2833825	0.7125706	ATF7IP2	0.397690449
13102	0.1611752	0.405278	GOLGA7B	0.397690449
13103	14.567823	36.649181	RRP12	0.397493816
13104	10.367555	26.099451	FOXJ3	0.397232676
13105	0.01	0.025184	MUC6	0.397076926
13106	1.4324134	3.6082735	ZBTB39	0.396980288
13107	26.836313	67.619033	C8orf33	0.396875139
13108	5.7151675	14.410375	SMIM20	0.396600887
13109	4.3336564	10.928891	ASCC3	0.396532128
13110	3.7393177	9.4324094	ENOSF1	0.39643293
13111	13.31134	33.578574	UBE3A	0.396423631
13112	7.0786529	17.856852	GMEB2	0.396411016
13113	9.0195683	22.771693	GEM	0.396086859

13114	14.444918	36.484772	RFC4	0.395916366
13115	6.0769931	15.354561	TBL1XR1	0.39577771
13116	1.8625996	4.7081053	COBLL1	0.395615543
13117	0.5855184	1.4810086	NANOS1	0.395351094
13118	1.9546851	4.9441752	HES6	0.395351094
13119	3.1068733	7.858517	BOLA1	0.395351094
13120	1.8895198	4.7793464	VWA8	0.395351094
13121	0.4342352	1.0983534	RAPGEF3	0.395351094
13122	1.0763582	2.7225375	SHF	0.395351094
13123	4.9008359	12.400401	ORC2	0.395215931
13124	7.5614603	19.136452	GTPBP5	0.395133868
13125	2.8754202	7.2832665	ARHGEF19	0.394798155
13126	1.4583446	3.6948452	ZDBF2	0.39469708
13127	0.8866763	2.2473058	ANKRD26	0.394550788
13128	17.492801	44.366074	MAPK1IP1L	0.39428327
13129	4.3246568	10.969127	UCHL5	0.39425715
13130	2.0923645	5.3089027	ARID2	0.394123719
13131	23.824734	60.45141	EIF2B2	0.39411379
13132	1.4264779	3.6213945	SH3BGRL2	0.393902921
13133	6.1503937	15.613983	LRRC8D	0.393902921
13134	1.6622094	4.2198453	MGC12916	0.393902921
13135	7.935497	20.154785	LPIN1	0.393727698
13136	36.175661	91.901801	TRIP13	0.393633862
13137	15.032095	38.194969	SDHD	0.393562175
13138	1.5552202	3.9526044	LOC100288637	0.393467188
13139	4.065288	10.346399	ISG20	0.392918164
13140	2.4639642	6.2709349	POLG2	0.392918164
13141	8.1199936	20.676998	HIPK2	0.392706605
13142	8.2425643	20.9918	C1QL1	0.392656393
13143	9.7549836	24.848718	RPRD2	0.392574929
13144	11.57775	29.500059	MTA3	0.392465319
13145	8.2467214	21.018025	HAUS7	0.392364238
13146	1.3409737	3.4190626	ZNF267	0.392205064
13147	2.4669298	6.2898978	SRBD1	0.392205064
13148	10.890014	27.815223	KPNA3	0.391512734
13149	29.725325	75.960244	RDH11	0.391327402
13150	9.5197878	24.328853	MAPK6	0.391296211
13151	1.9031918	4.8644947	HSD17B7	0.391241415
13152	1.119854	2.8623095	BCL11B	0.391241415
13153	0.4873214	1.2455771	CELSR3	0.391241415
13154	34.640131	88.616212	ATP5I	0.390900613
13155	8.0467473	20.598315	TCF20	0.390650751
13156	3.5321949	9.0425255	LINC00263	0.390620397

13157	6.6855935	17.118943	CKAP2L	0.390537743
13158	17.020159	43.601685	MRFAP1L1	0.390355525
13159	8.3258197	21.372151	ATP13A3	0.389563952
13160	4.8870383	12.553466	MET	0.389297941
13161	3.344301	8.5949825	OSBPL3	0.389099227
13162	16.019921	41.179365	PROCR	0.389027885
13163	29.356586	75.502444	MRPL2	0.388816365
13164	24.120313	62.14392	PTPN12	0.388136324
13165	586.80328	1512.3601	RPL14	0.388004999
13166	0.0528799	0.1363768	PRLR	0.387748189
13167	0.1547138	0.3990059	ANKRD24	0.387748189
13168	0.1664828	0.4293581	KCNQ3	0.387748189
13169	0.1903771	0.4909814	CASC2	0.387748189
13170	0.1598156	0.4121633	CES3	0.387748189
13171	0.2568605	0.6624416	NKAPP1	0.387748189
13172	0.4373547	1.1279348	BAALC	0.387748188
13173	0.2314693	0.5969577	C1RL-AS1	0.387748188
13174	0.1721362	0.443938	SYT12	0.387748188
13175	0.3079796	0.7942772	GPR89A	0.387748188
13176	13.397111	34.55106	LAP3	0.387748188
13177	0.1828066	0.471457	NGFR	0.387748188
13178	1.0019207	2.5839468	LOC100289341	0.387748188
13179	6.9894975	18.025868	ANKZF1	0.387748188
13180	0.2379895	0.6137734	LPAR4	0.387748188
13181	5.1862173	13.37522	NDUFAF4	0.387748188
13182	0.4396614	1.1338838	LOC100129961	0.387748188
13183	3.4149498	8.8071328	C14orf93	0.387748188
13184	3.0146812	7.7748428	DTD2	0.387748188
13185	0.4423053	1.1407024	DPY19L2P1	0.387748188
13186	1.9515277	5.0329769	ADAL	0.387748188
13187	3.071949	7.9225359	ARHGAP19	0.387748188
13188	4.8294828	12.455204	MTERFD1	0.387748188
13189	1.5380037	3.9665013	ARHGEF3	0.387748188
13190	1.8017248	4.6466362	MMP17	0.387748188
13191	10.061463	25.948446	ZCCHC9	0.387748188
13192	0.2279251	0.5878173	ZNF19	0.387748188
13193	0.3536191	0.9119812	CCDC113	0.387748188
13194	0.1085414	0.2799276	MYLK4	0.387748188
13195	9.2116886	23.776167	GATA2	0.387433713
13196	3.0674793	7.9245549	LRBA	0.387085371
13197	13.239258	34.20962	PAK2	0.38700395
13198	18.011912	46.561134	NRGN	0.386844346
13199	7.3393136	18.997629	CACUL1	0.386327865

13200	2.7764824	7.1868551	GLIS3	0.386327865
13201	20.246263	52.411274	CMTM7	0.386295948
13202	38.547884	99.803077	MT1E	0.38623944
13203	7.8891727	20.429315	ZNF217	0.386169225
13204	3.0582736	7.9224783	C14orf79	0.386024863
13205	32.796254	84.991911	CCNK	0.385875009
13206	11.754061	30.465213	MARK3	0.385819093
13207	7.0943665	18.392418	ZFP106	0.385722335
13208	2.9325678	7.603302	MIOS	0.385696611
13209	33.669896	87.318388	ZFP36L1	0.385599148
13210	6.7758759	17.581493	MLKL	0.385398199
13211	2.8661974	7.4392881	NFIB	0.385278454
13212	17.686256	45.956206	ACTL6A	0.38485022
13213	0.7286696	1.8939155	RANBP17	0.384742388
13214	1.8182507	4.7343454	LNPEP	0.384055348
13215	4.1311276	10.756594	CWC22	0.384055348
13216	0.01	0.0260431	PLCXD3	0.383978306
13217	6.0458632	15.74635	ARNTL2	0.383953301
13218	4.3434481	11.312632	PNMA2	0.383946735
13219	5.2175492	13.602286	ZBED5	0.383578853
13220	20.25557	52.819729	B4GALT5	0.383484934
13221	1.3984361	3.6484941	CUL5	0.383291313
13222	0.3802911	0.9930277	C5	0.382961174
13223	4.2865543	11.202003	SRD5A1	0.382659629
13224	1.182902	3.0955596	FAM169A	0.382128649
13225	30.021537	78.635114	COA4	0.381782831
13226	26.625555	69.74982	IPO5	0.381729373
13227	9.1248369	23.912456	MZT2A	0.381593455
13228	12.434092	32.597482	ZGPAT	0.38144334
13229	4.8302928	12.666464	SMCHD1	0.381345007
13230	5.3153786	13.944677	RAB15	0.381176185
13231	9.9369298	26.072194	SIN3A	0.381131325
13232	8.64472	22.692795	YDJC	0.380945588
13233	1.6131181	4.2345106	NXT2	0.380945588
13234	7.4210824	19.495992	TDP1	0.380646573
13235	2.6172008	6.8756716	EFNA5	0.380646573
13236	14.667168	38.54253	RBM8A	0.380545015
13237	59.240467	155.67951	GLRX5	0.380528342
13238	3.3843106	8.8985855	AKAP10	0.380320062
13239	2.7477478	7.2248301	EED	0.380320062
13240	1.2374043	3.2577419	FUT4	0.37983496
13241	12.975473	34.170638	AMD1	0.379725812
13242	31.156877	82.073004	SSB	0.37962394

13243	1.9358057	5.1000256	KLHL26	0.379567847
13244	0.01	0.0263461	FLJ16779	0.379562474
13245	3.3639952	8.8655026	GPATCH2L	0.379447768
13246	4.3541067	11.476916	DSN1	0.379379522
13247	14.390424	37.95628	RGS19	0.379131562
13248	11.765151	31.031844	FRMD6	0.379131562
13249	15.623274	41.240984	FAM101B	0.378828845
13250	1.2192887	3.2189014	SAMD5	0.378790309
13251	12.098417	31.970795	WDR54	0.378420912
13252	33.5732	88.776085	DUSP5	0.378178429
13253	8.1163608	21.470733	NDUFB6	0.378019739
13254	41.47757	109.72436	CAPRIN1	0.378016067
13255	3.4537753	9.1383483	ZNF503	0.377943062
13256	0.9425352	2.4947603	ZNF619	0.377805927
13257	17.979565	47.602656	ABR	0.377700877
13258	855.89589	2266.7912	RPL4	0.37758038
13259	24.341606	64.508463	HJURP	0.377339725
13260	11.73036	31.090209	TARS2	0.377300777
13261	59.131618	156.74946	NOP56	0.377236496
13262	1.8573931	4.9270673	FBXW7	0.376977405
13263	25.662956	68.075582	EIF3J	0.376977405
13264	10.672228	28.313344	DIDO1	0.376932787
13265	3.3160263	8.7987024	FASTKD3	0.376876744
13266	5.2283185	13.874336	LARP4	0.376833795
13267	5.94732	15.79127	SASH1	0.376620757
13268	10.920052	29.018152	HOXB4	0.376317969
13269	166.37098	442.18149	NONO	0.37625043
13270	5.7357661	15.247656	SNX27	0.376173615
13271	0.2545337	0.6769546	HAS2	0.375998244
13272	0.1809023	0.4811255	MAP7	0.375998243
13273	0.2917739	0.775998	HTR1D	0.375998243
13274	0.574499	1.5279299	HSD17B2	0.375998243
13275	0.3119753	0.8297254	HEY2	0.375998243
13276	0.1949025	0.5183601	KLLN	0.375998243
13277	0.2844074	0.7564061	C9orf172	0.375998243
13278	1.2707287	3.3796133	TTLL11	0.375998243
13279	5.2427547	13.943562	UMPS	0.375998243
13280	2.2481068	5.9790354	RSC1A1	0.375998243
13281	1.432299	3.8093236	HIST2H2BC	0.375998243
13282	1.1453144	3.0460632	ZSCAN12	0.375998243
13283	0.5596495	1.4884366	ZNF33B	0.375998243
13284	4.3443446	11.564562	DHX57	0.375660115
13285	7.0001758	18.635775	SVIL	0.375631057

13286	3.167529	8.4387685	NUFIP1	0.37535441
13287	12.472788	33.23529	CYP51A1	0.375287471
13288	5.9542714	15.866104	SUSD5	0.37528251
13289	11.602189	30.950092	DDX18	0.374867664
13290	0.6850954	1.8299585	USP45	0.374377561
13291	4.9150826	13.131508	ELOVL6	0.374296894
13292	9.3032871	24.863528	PSME4	0.374174051
13293	28.722249	76.78389	AURKA	0.374066084
13294	17.392202	46.503431	CNRIP1	0.373998252
13295	0.8413383	2.2511736	CNTRL	0.373733193
13296	43.340342	116.01589	VOPP1	0.373572448
13297	42.20108	112.99001	DEK	0.373493918
13298	62.691608	167.86426	MRFAP1	0.373466094
13299	1.2176151	3.2609989	LOC285074	0.373387144
13300	2.7701365	7.4189392	FRG1B	0.373387144
13301	3.7227581	9.9795301	CEP250	0.373039419
13302	17.251614	46.249225	OGFOD1	0.37301413
13303	2.6532344	7.1148256	HOXA4	0.37291629
13304	1.2090008	3.2427253	ALMS1	0.372834796
13305	13.180823	35.37136	FAHD1	0.372641116
13306	23.808174	63.898058	RAB31	0.372596213
13307	45.198333	121.36496	TRAP1	0.372416664
13308	42.215581	113.3573	FASN	0.372411677
13309	1.1865851	3.1877032	ZNF480	0.372238261
13310	3.3500753	8.9998146	ZBTB38	0.372238261
13311	4.8534674	13.038604	EXO1	0.372238261
13312	7.6906223	20.667516	SLC7A6	0.372111584
13313	4.3882294	11.797662	DNAJC13	0.371957538
13314	2.2808341	6.1340649	EDEM3	0.371830775
13315	3.8282342	10.297221	PUS7	0.371773544
13316	0.6320609	1.7028523	HTATSF1P2	0.371177753
13317	2.6427473	7.1198968	LOC645166	0.371177753
13318	1.2690705	3.419037	C7orf13	0.371177753
13319	7.003773	18.879348	GSG2	0.370975366
13320	9.991218	26.933914	FANCG	0.370953069
13321	23.306561	62.831086	SNW1	0.370939971
13322	7.8149813	21.070911	PP7080	0.370889571
13323	5.9937909	16.163959	KLHDC2	0.37081206
13324	261.74877	706.02163	FOSL1	0.370737614
13325	14.879408	40.156753	GNAI3	0.370533152
13326	4.2697207	11.526253	URB2	0.370434406
13327	2.059538	5.560513	KIF15	0.370386329
13328	7.4868977	20.24118	RFC1	0.36988445

13329	2.6461714	7.1552358	FAM155A	0.369823089
13330	8.7413302	23.641099	MAN1A2	0.369751437
13331	0.9278695	2.5126179	PARP11	0.369283989
13332	33.553997	90.862313	DLST	0.369283989
13333	2.6875396	7.2777041	LOC652276	0.369283989
13334	5.181203	14.030403	HSPA14	0.369283989
13335	0.1013912	0.2745616	WDR52	0.369283987
13336	0.0757541	0.2053119	MSH5	0.368970768
13337	20.05164	54.3908	ARF6	0.368658672
13338	2.3595268	6.4041865	ATG4C	0.36843506
13339	2.7206201	7.3860783	MAST4	0.368344335
13340	5.067025	13.756218	SMARCA2	0.368344335
13341	0.7491203	2.0341481	RIMS3	0.368272252
13342	5.5149828	14.977359	PRPF4B	0.368221301
13343	15.823548	42.979509	RFWD3	0.368164946
13344	3.3761925	9.1736339	SMARCAD1	0.368032179
13345	2.3561737	6.4039569	NOL8	0.367924661
13346	57.167673	155.42738	AHSA1	0.367809534
13347	28.850809	78.466274	CNIH	0.367684203
13348	3.7353159	10.160179	DEPDC1	0.367642727
13349	12.493179	33.999408	EPRS	0.367452828
13350	1.1888414	3.2363483	PPFIA3	0.367340389
13351	19.48563	53.06001	KIAA0101	0.367237579
13352	10.179177	27.734	PARP2	0.367028819
13353	0.2198307	0.5989535	NEIL1	0.367024656
13354	32.019715	87.258671	MRPS18B	0.366951669
13355	28.457915	77.556402	MTA1	0.366931861
13356	2.3296692	6.3495765	BRCC3	0.366901511
13357	2.7301245	7.4410281	NARS2	0.366901511
13358	61.18938	166.91886	CNBP	0.366581591
13359	3.4425876	9.3936755	PAAF1	0.3664793
13360	1.0459969	2.8563025	ZNF200	0.366206622
13361	16.391407	44.784246	XPO5	0.366008332
13362	58.453518	159.76354	IFITM2	0.365875213
13363	2.8900586	7.9023425	DCAF10	0.365721764
13364	2.7522229	7.5272769	PDP2	0.365633272
13365	0.7478707	2.0458563	TRIM66	0.365553848
13366	7.6664439	20.976546	YEATS2	0.365476937
13367	2.2593845	6.1849486	CHM	0.365303682
13368	8.1602214	22.340243	QDPR	0.365270032
13369	0.3415452	0.9358955	SNX22	0.364939472
13370	6.5229378	17.874027	RPE	0.364939471
13371	2.8504491	7.8107448	SDCCAG8	0.364939471

13372	0.1984757	0.5438593	PAIP2B	0.36493947
13373	2.8469877	7.8096036	NRDE2	0.364549579
13374	39.228141	107.61378	PHF5A	0.36452711
13375	2.4252175	6.6546245	ZBTB14	0.36444092
13376	14.064985	38.604459	CDK5RAP1	0.364335767
13377	5.51663	15.1544	RNF2	0.364028262
13378	17.219865	47.311701	CINP	0.363966299
13379	1.440899	3.9605472	NEU3	0.363813115
13380	2.1759977	5.9810865	KIAA1468	0.363813115
13381	2.0284787	5.5756064	TGDS	0.363813115
13382	0.723345	1.9882323	ZNF260	0.363813115
13383	139.44784	383.35702	ATP1A1	0.363754493
13384	18.268787	50.241545	CYCS	0.36361914
13385	1.120804	3.0824965	FAM135A	0.363602697
13386	4.475907	12.310843	NIPA1	0.363574361
13387	1.5444429	4.2482174	LCOR	0.363550813
13388	2.1057727	5.7963065	SLC5A3	0.3632956
13389	4.7286068	13.019035	KIAA1430	0.363207164
13390	6.4328326	17.724942	RGP1	0.362925457
13391	282.26072	777.93702	C17orf76-AS1	0.362832352
13392	5.7676314	15.90194	TMEM2	0.362699856
13393	1.5712892	4.3337571	TRIM45	0.362569734
13394	1.7294564	4.7738684	LOC646762	0.362275679
13395	3.016033	8.3252427	RIF1	0.362275679
13396	4.2554195	11.747916	UPF2	0.362227607
13397	3.6654513	10.121062	DBF4	0.362160744
13398	18.590054	51.34305	MYBBP1A	0.362075363
13399	2.3499687	6.4909908	FRYL	0.362035444
13400	0.9910302	2.7384218	RGS17	0.361898309
13401	0.9729634	2.6884995	IPW	0.361898309
13402	2.6752182	7.3921822	ASPM	0.361898309
13403	2.2803876	6.3011833	BAG4	0.361898309
13404	0.01	0.0276472	UNC5D	0.361700712
13405	10.446762	28.885071	FAM98B	0.361666472
13406	1.8465311	5.1074503	PAN3	0.361536772
13407	0.01	0.0276699	TLL1	0.361403011
13408	7.5495318	20.896115	AMOTL1	0.361288773
13409	4.1976631	11.619011	USP3	0.36127542
13410	3.5039849	9.7003903	PAQR8	0.36122102
13411	7.0634759	19.555095	SAPCD2	0.361208979
13412	9.7748358	27.07472	PRMT6	0.361031833
13413	16.206893	44.892526	MRPL17	0.361015391
13414	12.149682	33.681004	PPP2R5C	0.360728008

13415	4.4637828	12.378939	KDM1B	0.360594954
13416	8.2783912	22.974366	TATDN1	0.36033165
13417	6.4952099	18.043077	COX10	0.359983503
13418	4.8387394	13.447281	CBL	0.359830319
13419	46.233166	128.49381	RUVBL1	0.359808512
13420	5.0412831	14.012374	C5orf51	0.359773661
13421	7.2510135	20.154785	ZNF146	0.35976636
13422	2.1050455	5.8530309	CAMKMT	0.359650493
13423	0.2367167	0.6581855	KIAA1377	0.359650493
13424	5.9151818	16.461622	FUT8	0.359331654
13425	2.5849214	7.1967846	PPARGC1B	0.359177269
13426	12.534356	34.89741	CHCHD4	0.359177269
13427	0.9237318	2.5717993	SHROOM2	0.359177269
13428	22.897112	63.751401	CBX3	0.359162501
13429	63.59469	177.10031	ATIC	0.35908854
13430	3.7879025	10.550995	SMG1	0.359009024
13431	6.7796876	18.887465	TMX1	0.358951691
13432	4.7792952	13.329031	IFT46	0.358562841
13433	0.6151666	1.7161772	ZNF714	0.358451659
13434	1.2175239	3.4016514	FDXACB1	0.357921404
13435	16.818205	47.027831	ECSIT	0.357622389
13436	4.9618929	13.879485	C4orf32	0.357498329
13437	1.360337	3.8073028	ARHGAP32	0.35729676
13438	1.0185852	2.8523696	KIAA2018	0.357101424
13439	5.0021077	14.01034	GOLPH3L	0.357029722
13440	22.100431	61.927201	PRPF3	0.356877598
13441	6.3832774	17.888862	KIAA0391	0.356829705
13442	16.824914	47.151102	ELK3	0.356829705
13443	5.1033976	14.307023	MSH2	0.356705757
13444	1.2761758	3.579233	NT5M	0.356550058
13445	3.9712179	11.141357	NAA25	0.356439328
13446	8.8680638	24.890507	LEO1	0.356282979
13447	2.639046	7.4098473	ZDHHC13	0.356153891
13448	3.9931189	11.219297	SMC2	0.355915251
13449	1.7039205	4.7886031	VGLL3	0.355828297
13450	1.4231293	3.9999893	ZNF696	0.355783284
13451	4.4515716	12.517014	ABCB7	0.35564165
13452	9.7631793	27.46819	PDCD2L	0.355435839
13453	2.8388786	7.9870354	SGK196	0.355435839
13454	5.7619632	16.224429	PIGO	0.355141198
13455	0.01	0.0281623	CCDC168	0.35508524
13456	80.113155	225.64873	AHCY	0.355034821
13457	1.8336605	5.1659165	KBTBD6	0.354953565

13458	16.805035	47.355082	PNPLA2	0.354872906
13459	13.907787	39.195697	NAE1	0.354829441
13460	1.481513	4.1790134	SLC45A4	0.354512629
13461	4.7679139	13.452938	ARFGEF2	0.354414317
13462	1.5455317	4.3645781	ALG6	0.354107934
13463	2.5801843	7.2941126	BMP2	0.353735189
13464	3.5949731	10.166946	PTER	0.353594203
13465	11.755187	33.248102	EIF5B	0.353559638
13466	15.858185	44.900522	NET1	0.353184866
13467	4.865358	13.780092	NUPL2	0.353071521
13468	71.066496	201.28995	APEX1	0.353055365
13469	2.8312818	8.0209792	BRCA1	0.352984558
13470	3.1984916	9.0737776	ADCK3	0.352498353
13471	9.072158	25.755607	ZC3H13	0.352240112
13472	1.710822	4.8603124	NADKD1	0.351998355
13473	72.831032	207.10093	XRCC5	0.351669257
13474	1.4666631	4.1724328	SYNE2	0.351512706
13475	1.6127218	4.5924488	AADAT	0.35116817
13476	5.6296525	16.036715	MRRF	0.351047742
13477	2.9669633	8.4552153	RNF38	0.350903338
13478	0.9353658	2.6686093	MMP15	0.350506837
13479	1.5565108	4.449106	SSX2IP	0.349847989
13480	5.0705966	14.496753	CENPF	0.349774646
13481	2.2787952	6.5168322	DIEXF	0.349678366
13482	100.6874	288.10522	ERH	0.349481337
13483	327.19532	936.37404	RPS25	0.349428015
13484	6.9363166	19.862469	TOPBP1	0.349217236
13485	3.2667124	9.3564214	PPP1R13B	0.349141226
13486	33.096605	94.852943	MRPS2	0.34892544
13487	1.7844606	5.1157405	ATHL1	0.348817647
13488	2.0550048	5.891344	ANKRD12	0.348817647
13489	1.4412137	4.1350296	ZNF197	0.348537697
13490	1.7907583	5.1415267	PIK3CA	0.348293109
13491	2.0587024	5.916166	MAML3	0.347979143
13492	85.940083	247.18037	UBAP2L	0.347681665
13493	17.077768	49.140063	KTN1	0.347532486
13494	3.9677149	11.420436	KLHL9	0.347422377
13495	2.0135217	5.7977093	ROBO3	0.347296087
13496	3.927763	11.322362	ANKH	0.346903141
13497	82.069623	237.24764	C1QBP	0.345923877
13498	60.935526	176.3249	DANCR	0.34558662
13499	5.6638929	16.407438	LPHN1	0.345202755
13500	17.239576	49.961636	CAD	0.345056277

13501	0.0531903	0.1543245	RIMS1	0.344665059
13502	0.067269	0.1951722	CCDC30	0.344665059
13503	0.0218815	0.0634863	FAT3	0.344665059
13504	0.0733801	0.2129027	GPAT2	0.344665058
13505	0.082649	0.2397952	CECR6	0.344665058
13506	0.0499879	0.1450332	RND2	0.344665058
13507	0.0887562	0.2575143	MST1	0.344665058
13508	0.0769002	0.2231157	AKR1D1	0.344665058
13509	0.0880065	0.2553393	FLJ13197	0.344665058
13510	0.0285518	0.0828392	RGPD8	0.344665058
13511	0.0999518	0.2899969	TRIM17	0.344665058
13512	0.0993325	0.2882	C11orf35	0.344665058
13513	0.0395896	0.1148639	LINC00176	0.344665058
13514	0.0481515	0.1397051	FAM21B	0.344665058
13515	0.1234594	0.3582012	KCTD14	0.344665058
13516	0.079119	0.2295534	KBTBD12	0.344665058
13517	0.1068168	0.3099147	TEX19	0.344665057
13518	0.089808	0.2605661	PCDHGC5	0.344665057
13519	0.0853397	0.2476018	MYRIP	0.344665057
13520	0.1194266	0.3465006	PPP1R3G	0.344665057
13521	0.206132	0.5980648	LOC100505875	0.344665057
13522	0.2749334	0.7976828	ATP6AP1L	0.344665057
13523	0.2305304	0.6688535	RAD21L1	0.344665057
13524	0.1240289	0.3598533	COL4A6	0.344665057
13525	0.0623391	0.1808685	DGKB	0.344665057
13526	0.2947659	0.8552242	LEAP2	0.344665057
13527	0.1164243	0.3377897	PROC	0.344665057
13528	0.1935899	0.5616754	BAIAP2L2	0.344665057
13529	0.0586876	0.1702742	HEATR4	0.344665057
13530	0.1763109	0.5115428	GLYATL1	0.344665057
13531	0.227759	0.6608126	TBX6	0.344665057
13532	0.1393977	0.4044438	ZNF420	0.344665057
13533	0.4190338	1.2157712	GAPDHS	0.344665057
13534	0.1655936	0.4804478	ACVR2B-AS1	0.344665057
13535	0.3152791	0.9147406	LOC100133985	0.344665057
13536	0.181217	0.525777	TAS2R5	0.344665057
13537	0.2248107	0.6522584	LARS2-AS1	0.344665056
13538	0.183936	0.533666	LOC285540	0.344665056
13539	0.0875996	0.2541587	EGR4	0.344665056
13540	0.1921087	0.5573779	PPFIA2	0.344665056
13541	0.2346841	0.6809049	GPR146	0.344665056
13542	0.6053726	1.7564083	RPH3AL	0.344665056
13543	0.5289327	1.5346283	LOC389641	0.344665056

13544	0.6127144	1.7777098	PIGZ	0.344665056
13545	0.2507816	0.7276096	NPPA-AS1	0.344665056
13546	0.1251649	0.3631493	DUSP26	0.344665056
13547	0.5937308	1.7226312	C5orf54	0.344665056
13548	0.2482917	0.7203855	LINC00638	0.344665056
13549	0.5223045	1.5153973	BACE1-AS	0.344665056
13550	0.348203	1.0102649	CTRL	0.344665056
13551	0.0805564	0.2337238	CPNE4	0.344665056
13552	0.1920733	0.5572752	MOG	0.344665056
13553	0.084304	0.2445969	DUSP5P1	0.344665056
13554	0.6868157	1.9927047	TUBA8	0.344665056
13555	0.516693	1.4991162	LOC100134259	0.344665056
13556	0.6047138	1.754497	ZNF138	0.344665056
13557	0.5874545	1.7044215	ARHGAP40	0.344665056
13558	0.338861	0.9831602	SLC7A5P1	0.344665056
13559	0.677722	1.9663205	UCN	0.344665056
13560	0.0785228	0.2278235	AQP6	0.344665056
13561	0.0391214	0.1135055	AATK	0.344665056
13562	0.8746259	2.5376112	RCOR2	0.344665056
13563	0.1930519	0.5601144	LOC100272217	0.344665056
13564	0.3847375	1.116265	LOC100271722	0.344665056
13565	0.1340621	0.3889634	BCDIN3D-AS1	0.344665056
13566	0.8982737	2.6062222	APOC1	0.344665056
13567	1.2554187	3.642431	MRGPRX3	0.344665056
13568	0.3937638	1.1424536	LINC00630	0.344665056
13569	17.45134	50.632752	CMSS1	0.344665056
13570	0.2731317	0.7924555	TMEM253	0.344665056
13571	0.1638361	0.4753487	SCLY	0.344665056
13572	0.0732254	0.2124538	CCDC148	0.344665056
13573	2.706487	7.8525135	SNORD47	0.344665056
13574	0.5602137	1.6253859	MILR1	0.344665056
13575	0.3992328	1.158321	ARHGAP27	0.344665056
13576	0.1656594	0.4806387	CD79A	0.344665056
13577	0.1061097	0.3078633	TUBA3FP	0.344665056
13578	1.0997335	3.1907311	TDRD3	0.344665056
13579	13.35342	38.743179	GNL2	0.344665056
13580	1.4708284	4.2674136	MMS22L	0.344665056
13581	0.0571427	0.165792	LOC100507391	0.344665056
13582	0.7750911	2.2488242	PDE10A	0.344665056
13583	0.8465268	2.4560853	SBF2-AS1	0.344665056
13584	0.2522996	0.732014	EPN2-IT1	0.344665056
13585	0.0908851	0.263691	GPR35	0.344665056
13586	0.1659232	0.4814041	LOC388906	0.344665056

13587	0.0601442	0.1745003	FAM107A	0.344665056
13588	0.0904512	0.2624321	CRYM-AS1	0.344665056
13589	0.1548288	0.4492151	THAP9	0.344665056
13590	0.1243434	0.3607658	AMACR	0.344665056
13591	0.0739267	0.2144887	NOSTRIN	0.344665056
13592	0.0637307	0.1849063	GRIN3B	0.344665056
13593	0.3392287	0.9842271	ANXA9	0.344665056
13594	0.1020066	0.2959587	LOC285972	0.344665056
13595	0.0945551	0.2743392	LOC257358	0.344665056
13596	0.0436622	0.12668	ATP1B4	0.344665056
13597	0.2710007	0.7862725	LST1	0.344665055
13598	0.1564561	0.4539366	FBP2	0.344665055
13599	0.0722105	0.2095092	RTEL1-TNFRSF6B	0.344665055
13600	0.278609	0.808347	EGR2	0.344665055
13601	0.0965856	0.2802303	PLCH1	0.344665055
13602	0.1376028	0.3992364	ESRRB	0.344665055
13603	0.1036813	0.3008177	SPAG8	0.344665055
13604	0.1115032	0.3235118	AGPAT4-IT1	0.344665055
13605	0.025042	0.072656	IL16	0.344665055
13606	0.0433894	0.1258887	C20orf203	0.344665055
13607	0.0314044	0.0911157	LINC00643	0.344665055
13608	0.1398654	0.405801	ETV2	0.344665055
13609	0.0702036	0.2036866	C11orf87	0.344665055
13610	0.0760305	0.2205923	LOC730227	0.344665055
13611	0.1179397	0.3421865	FBXO15	0.344665055
13612	0.0812157	0.2356366	LOC283731	0.344665054
13613	0.0879694	0.2552316	HSH2D	0.344665054
13614	0.0905691	0.2627742	PDZK1	0.344665054
13615	0.0338586	0.0982362	CALCRL	0.344665054
13616	0.045113	0.1308894	LRRK2	0.344665054
13617	0.0834934	0.242245	CHST8	0.344665054
13618	0.0520218	0.1509345	MEI1	0.344665053
13619	0.06458	0.1873702	LRRN2	0.344665053
13620	0.0699562	0.2029686	PLIN1	0.344665053
13621	0.043653	0.1266534	PCDHGA12	0.344665053
13622	0.068881	0.1998491	FER1L6	0.344665053
13623	0.0441524	0.1281024	BEND2	0.344665053
13624	0.0127019	0.0368528	FCGBP	0.344665052
13625	0.0218288	0.0633334	POM121L10P	0.344665051
13626	12.83288	37.285048	FHOD1	0.344183007
13627	8.3887632	24.384464	ECT2	0.344020822
13628	0.01	0.0290834	MYH14	0.343838954
13629	161.99077	471.19565	EIF4G2	0.343786649

13630	7.3511582	21.384836	OXCT1	0.34375565
13631	7.8408393	22.813693	DDX6	0.343690049
13632	3.7608519	10.944982	EYA3	0.343614248
13633	35.372156	102.96466	GINS2	0.343536856
13634	3.646316	10.61662	USP24	0.34345358
13635	1.4833373	4.3218657	ATG2B	0.343216884
13636	40.46234	117.93136	NSUN2	0.343100767
13637	6.4578283	18.823276	TAF4	0.343076738
13638	7.7869706	22.701223	NFE2L3	0.343019877
13639	4.0180623	11.715875	NOC3L	0.342958793
13640	7.0441849	20.542578	TMA16	0.342906561
13641	6.9695008	20.331581	DAB2IP	0.342791877
13642	4.1520207	12.123759	SPATA5L1	0.342469737
13643	6.2241984	18.20201	THOC2	0.341951158
13644	8.9444648	26.203746	ORC1	0.341342983
13645	2.4577253	7.2062222	MINA	0.341055998
13646	9.1377433	26.797026	PLAGL2	0.340998407
13647	29.353073	86.084363	TACC3	0.340980315
13648	4.6778788	13.723048	ACACA	0.340877528
13649	89.760986	263.64134	CSDE1	0.340466276
13650	1.8186123	5.3416042	XRCC2	0.340461824
13651	25.556941	75.065512	SOX9	0.340461824
13652	69.157072	203.2935	EIF4B	0.340183396
13653	2.0633614	6.0697165	NEK3	0.339943617
13654	2.1404669	6.2983702	KLHL8	0.339844566
13655	12.062764	35.497842	GID8	0.339816826
13656	5.4067783	15.917743	SLC37A4	0.33966991
13657	11.088637	32.657218	GATAD2B	0.339546268
13658	3.5287576	10.39513	RSF1	0.339462565
13659	1.3439793	3.9593684	MGC27345	0.339442858
13660	1.7427418	5.1347273	LEPR	0.339402994
13661	16.180085	47.709394	SORD	0.339138357
13662	112.94482	333.07	FSCN1	0.339102354
13663	2.5240933	7.4485074	RSBN1L	0.338872366
13664	19.553436	57.72099	SMC4	0.338757811
13665	8.8409097	26.09956	KPNA4	0.338737884
13666	1.1591307	3.4220653	CCDC69	0.338722555
13667	89.820302	265.19006	TPM3	0.338701611
13668	3.1156071	9.2069199	ABHD17C	0.338398419
13669	5.8549204	17.303452	SETD7	0.33836719
13670	5.2913936	15.638567	PTPRU	0.338355398
13671	1.0807784	3.1972203	LOC344595	0.338036882
13672	5.7742935	17.091799	C14orf142	0.337840006

13673	1.6599526	4.9134281	LIPG	0.337840006
13674	17.827686	52.776541	NUDT21	0.337795649
13675	2.6492607	7.843346	TAF1	0.337771755
13676	12.013472	35.577891	DARS2	0.337666781
13677	4.6755578	13.848126	QSER1	0.337631075
13678	9.0983238	26.95332	SNHG7	0.33755856
13679	3.0509394	9.0486053	GNG4	0.337172338
13680	5.6279886	16.698773	FBXL19	0.337030071
13681	17.486809	51.886366	SMARCC1	0.337021275
13682	287.38809	852.73096	RAN	0.337020821
13683	10.943353	32.473789	FAM210B	0.336990328
13684	1.8280658	5.4258192	OTUD6B	0.336919774
13685	1.3438936	3.9919657	PACRGL	0.33664959
13686	3.2629185	9.6923289	RNF168	0.33664959
13687	2.4579554	7.3012278	FAM86A	0.33664959
13688	2.9398519	8.7326763	PHF3	0.33664959
13689	26.887081	79.914293	PLIN2	0.336448964
13690	10.537453	31.330465	SKA3	0.336332494
13691	1.3569715	4.0380235	EHHADH	0.33604843
13692	6.4667863	19.265372	OTUD7B	0.335668905
13693	0.945644	2.8178139	METTL8	0.335594923
13694	4.7310642	14.101599	SIX1	0.335498432
13695	0.7681919	2.2924874	NFIA	0.335091027
13696	5.8171528	17.369965	MMACHC	0.33489722
13697	4.5012142	13.449518	WDR20	0.334674765
13698	0.7179438	2.1461406	GDPD5	0.334527849
13699	1.2448518	3.7212201	XYLB	0.334527849
13700	3.9388222	11.774273	RASSF7	0.334527849
13701	48.429681	144.83702	PRMT5	0.334373649
13702	7.9727758	23.845902	RBM28	0.334345743
13703	9.9237857	29.69433	TEX10	0.334198005
13704	6.2453377	18.68814	HTT	0.334187238
13705	3.867098	11.578499	TUBGCP5	0.33398959
13706	2.9805696	8.9266867	SRSF8	0.333894273
13707	1.3098849	3.9240498	OTUD3	0.333809464
13708	1.0077345	3.0212685	FAM86JP	0.333546829
13709	6.2215584	18.665904	HMGCR	0.333311384
13710	5.0753796	15.237734	CDK17	0.333079676
13711	4.6141391	13.857044	PODXL	0.332981495
13712	37.519209	112.73972	SF3A3	0.332794938
13713	4.261559	12.812333	NAB1	0.33261383
13714	0.5121779	1.541054	LOC283788	0.33235559
13715	2.4379491	7.3353636	DLX2	0.33235559

13716	2.9329968	8.8344995	MIB1	0.331993547
13717	2.4528687	7.3903919	TXLNG	0.331899684
13718	3.1207413	9.4026641	FASTKD2	0.331899684
13719	21.599493	65.101332	SLC16A1	0.331782653
13720	19.063006	57.48163	BHLHE40	0.33163648
13721	9.971268	30.069992	WDR12	0.331601949
13722	6.2562997	18.882071	NT5C3A	0.331335468
13723	154.19215	465.42795	ILF2	0.331291135
13724	5.0662165	15.29486	UBA5	0.331236548
13725	2.570689	7.7608856	EPB41	0.331236547
13726	0.8437227	2.5499475	PGM5P2	0.330878454
13727	1.431684	4.3269181	PLK1S1	0.330878454
13728	2.9665409	8.9656516	HOXB6	0.330878454
13729	3.2341909	9.7816675	FIGNL1	0.33063799
13730	2.1436442	6.4899128	MSH3	0.330304012
13731	2.857184	8.6562939	FLJ42627	0.330070119
13732	8.0277157	24.326525	SMEK1	0.329998458
13733	49.650558	150.47835	IMP3	0.3299515
13734	3.1330832	9.5048676	KIF20B	0.329629332
13735	7.1210068	21.6123	BMS1	0.329488611
13736	28.276428	85.830098	DHX9	0.329446529
13737	0.8693662	2.6424628	NDUFA6-AS1	0.328998463
13738	0.6152663	1.8701192	LOC283922	0.328998463
13739	1.433606	4.3574854	FLT1	0.328998463
13740	0.01	0.0304085	DLGAP1	0.32885491
13741	8.5671909	26.054477	TUBB2B	0.328818387
13742	8.6419915	26.306721	PRIM1	0.328508882
13743	8.0180705	24.419736	STAC	0.32834387
13744	3.2091384	9.7811368	POLR3B	0.328094621
13745	15.381868	46.883868	PAFAH1B2	0.328084444
13746	7.5572038	23.041118	CCDC51	0.327987715
13747	6.9384871	21.157392	AQR	0.327946229
13748	4.6758882	14.268184	ALKBH1	0.327714316
13749	8.0846098	24.671953	KIF3B	0.327684221
13750	4.4998543	13.735717	ZW10	0.32760243
13751	3.7955227	11.599524	PRDM8	0.327213661
13752	11.636706	35.602657	DDX46	0.326849367
13753	0.01	0.0305978	EVPL	0.326820658
13754	12.023048	36.821241	SCO1	0.32652479
13755	9.9705451	30.535339	SCARB1	0.32652479
13756	0.424583	1.3003087	LOC100507412	0.32652479
13757	4.3358751	13.284445	RBPJ	0.326387364
13758	0.01	0.0306537	CSMD3	0.326225265

13759	2.065155	6.3421697	SPTY2D1	0.325622788
13760	4.5131102	13.864438	SNAPC5	0.325516998
13761	13.36687	41.082453	TOMM70A	0.325366901
13762	1.99191	6.1266503	NCR3LG1	0.325122192
13763	2.066151	6.3579763	C9orf41	0.32496991
13764	18.821612	57.947946	STEAP3	0.324802054
13765	1.7393803	5.3619928	SH2D5	0.324390641
13766	2.4299775	7.4978933	MYEF2	0.324088038
13767	14.95655	46.166576	CCDC85C	0.323969233
13768	9.152286	28.286756	ACSL4	0.323553748
13769	0.9427208	2.9152738	BRWD1	0.323373007
13770	0.2449453	0.7580549	IQCH	0.323123491
13771	0.2770533	0.8574224	XKR6	0.323123491
13772	1.7124035	5.2995325	ZBTB42	0.32312349
13773	3.638577	11.260639	TRNT1	0.32312349
13774	0.288562	0.8930395	HELB	0.32312349
13775	0.9971268	3.0859001	ARHGAP11B	0.32312349
13776	1.1734206	3.6314927	RNF219	0.32312349
13777	5.0796756	15.733383	STAG2	0.322859716
13778	1.1707837	3.6279537	MYO9A	0.322711868
13779	1.1721306	3.6326533	DENND4C	0.322665159
13780	4.6481732	14.414851	CD2AP	0.322457256
13781	16.914663	52.616183	NAA50	0.321472641
13782	6.3391483	19.723213	TTLL4	0.321405451
13783	0.01	0.0311175	FER1L5	0.321362897
13784	8.2997124	25.834279	ASUN	0.321267428
13785	13.139943	40.91955	SEC11C	0.321116512
13786	1.0226802	3.1869616	CCZ1	0.320895052
13787	0.9308166	2.9006886	PDCD4-AS1	0.320895052
13788	0.6594921	2.0551644	GAS1	0.320895052
13789	6.4705176	20.190206	MOB2	0.320478035
13790	5.7238315	17.86504	DDX10	0.320392869
13791	9.875648	30.833812	TOP2B	0.320286308
13792	3.022786	9.4384197	ATXN7L2	0.32026399
13793	4.2322027	13.220109	KIAA1524	0.320133735
13794	1.4743202	4.6130385	C11orf54	0.319598507
13795	4.6776688	14.657367	SIKE1	0.319134311
13796	2.5176869	7.8938264	ZBTB11	0.318943783
13797	0.01	0.0313547	DUOX2	0.318931717
13798	8.9587431	28.095568	RFC3	0.318866774
13799	9.4853113	29.773504	GEMIN4	0.318582295
13800	0.2194256	0.6896873	RAB9B	0.31815236
13801	0.4030938	1.2669836	KREMEN2	0.31815236

13802	0.795671	2.5009119	LTK	0.31815236
13803	0.6162931	1.9371004	PLXNA3	0.31815236
13804	0.5643859	1.7739486	SRGAP2D	0.31815236
13805	2.5136766	7.9008579	HOXA11	0.31815236
13806	7.1247692	22.394205	WDR4	0.31815236
13807	0.3695027	1.1614016	FLJ43681	0.318152359
13808	0.2564128	0.8059434	SYBU	0.318152359
13809	7.8824934	24.799373	RPRD1A	0.317850507
13810	4.5776158	14.422355	THUMPD1	0.317397251
13811	566.41042	1788.0574	PTMA	0.316774188
13812	9.9185085	31.349506	PRPF38B	0.316384846
13813	2.4623572	7.7831157	MXI1	0.316371656
13814	5.1071721	16.143585	ANAPC1	0.31635923
13815	5.2853031	16.714721	GOLGA4	0.316206474
13816	23.693615	74.940923	MRPS27	0.316163907
13817	2.9128266	9.2194695	SUV39H2	0.315942968
13818	1.2472223	3.9476183	ZNF597	0.315942968
13819	3.6070878	11.424824	PNPT1	0.315723715
13820	6.4491446	20.43397	SEMA7A	0.31560899
13821	3.3187742	10.525801	RBL1	0.315298975
13822	2.5868856	8.2060196	PAXBP1	0.315242429
13823	12.220673	38.769533	CEPT1	0.315213308
13824	5.5759064	17.709738	ZWILCH	0.31484974
13825	0.2190056	0.6959316	LOC441666	0.314694182
13826	0.01	0.031815	RIMBP2	0.314317423
13827	3.9363925	12.529749	ZNF770	0.314163724
13828	11.368427	36.188142	PDCD11	0.314147838
13829	16.945662	53.962238	INTS3	0.314028162
13830	2.0863131	6.6510042	TSEN2	0.313683927
13831	1.002885	3.2007117	LOC143666	0.313331869
13832	0.8386298	2.6764905	ZCWPW1	0.313331869
13833	0.5649214	1.802949	NBPF14	0.313331869
13834	52.817712	168.58988	CAPZA1	0.313291124
13835	2.0508656	6.5606037	MFI2	0.31260319
13836	1.6960285	5.4333855	TAF1A	0.312149485
13837	1.4822814	4.7486268	ESCO1	0.312149485
13838	7.8780239	25.242435	NCAPD3	0.312094451
13839	3.5902811	11.521529	TMEM251	0.311614982
13840	1.747918	5.6092233	CENPJ	0.311614982
13841	1.5869593	5.0954832	TAF5	0.311444328
13842	2.8508163	9.1591417	VEPH1	0.311253648
13843	3.1414877	10.099974	LMBR1	0.311039197
13844	3.8467356	12.367366	EMILIN2	0.311039197

13845	0.01	0.0321602	ALK	0.310943533
13846	4.7468323	15.267584	LRRC58	0.3109092
13847	14.864778	47.825872	ACO1	0.310810382
13848	2.5146934	8.0919825	SFMBT1	0.310763575
13849	55.399464	178.29545	ANP32A	0.310717198
13850	5.5271172	17.788788	HOXC4	0.310707908
13851	1.4400022	4.6374095	POU2F1	0.310518673
13852	68.197937	219.65624	MCL1	0.310475761
13853	0.379138	1.2222429	CATSPER2P1	0.310198551
13854	0.8471524	2.7310006	LY6G5C	0.310198551
13855	4.0576227	13.080727	ABLIM1	0.310198551
13856	24.248846	78.172015	LOC388796	0.310198551
13857	0.3017367	0.9727213	ZNF485	0.310198551
13858	0.1501437	0.4840246	PDZD7	0.31019855
13859	0.2599578	0.8380368	LPPR3	0.31019855
13860	111.88796	360.85607	EIF3F	0.310062558
13861	4.9261958	15.898044	TWISTNB	0.309861744
13862	17.614013	56.846829	YY1	0.309850404
13863	8.881061	28.675691	MAP4K5	0.309706952
13864	14.749113	47.679406	HMBS	0.309339275
13865	13.219724	42.738737	MAEA	0.309314794
13866	1.607913	5.206033	PPAPDC2	0.3088557
13867	3.46196	11.211194	TMEM41B	0.308794937
13868	2.3544873	7.6255623	NANP	0.308762446
13869	0.01	0.0324188	IKZF1	0.308462734
13870	4.2968969	13.933569	NUP35	0.308384524
13871	3.9529054	12.818106	RNF138	0.308384524
13872	2.4253236	7.8646088	ABCC4	0.308384524
13873	13.317008	43.231863	GOLPH3	0.308036888
13874	1.6110398	5.230673	MTFR2	0.307998561
13875	10.489549	34.057135	EIF2A	0.307998561
13876	12.497873	40.58472	GYG1	0.307945292
13877	19.573434	63.582221	ZNF259	0.307844446
13878	16.488752	53.598438	RGS10	0.307634926
13879	43.028367	139.89792	BZW1	0.307569749
13880	3.4163852	11.113667	RMND1	0.307403969
13881	3.9388222	12.813179	STRADB	0.307403969
13882	4.9618929	16.151918	ATL2	0.307201463
13883	5.7220172	18.665904	MAP7D3	0.306549156
13884	28.508705	93.022083	SDHA	0.306472442
13885	3.3302477	10.879003	BTBD7	0.306116991
13886	8.8630574	28.97677	XRCC3	0.305867681
13887	9.949495	32.559451	GEMIN5	0.305579328

13888	8.2655516	27.055942	KIF2A	0.305498573
13889	4.4077074	14.433668	RNF214	0.305376811
13890	5.4631117	17.893447	TIMM10B	0.305313534
13891	12.457123	40.810693	ATP8B2	0.305241635
13892	5.3680264	17.589755	CTTNBP2NL	0.305179156
13893	8.0153654	26.270125	MT1X	0.305113328
13894	10.349898	33.938864	SMURF2	0.3049571
13895	2.1391629	7.0231422	TCAIM	0.304587724
13896	4.9071831	16.120815	HLTF	0.304400447
13897	1.0440857	3.4331798	TMEM102	0.304116226
13898	1.0672559	3.5093685	POLQ	0.304116226
13899	1.2952113	4.2589352	ZBTB25	0.304116226
13900	1.44421	4.7488751	EPHX4	0.304116226
13901	0.2714242	0.8925015	ANKEF1	0.304116226
13902	0.3361824	1.1054407	ARHGAP20	0.304116226
13903	0.6330483	2.0815999	PRICKLE4	0.304116226
13904	0.3818239	1.2555198	ISL1	0.304116226
13905	4.4543405	14.66735	LRR1	0.303690889
13906	13.058055	43.005976	CENPV	0.303633502
13907	5.5728	18.358258	MAK16	0.303558215
13908	0.01	0.0329596	PCDHA11	0.303401897
13909	0.5527838	1.8265831	TMC7	0.302632732
13910	2.678446	8.854889	MPDZ	0.302482169
13911	18.483326	61.17938	LRRFIP1	0.302116923
13912	1.8756753	6.2139417	STIM2	0.301849521
13913	0.706439	2.3424447	C1QL4	0.301581924
13914	1.4911388	4.9479425	TRIP11	0.301365426
13915	3.1319539	10.394919	RANBP2	0.301296605
13916	28.04712	93.239201	CDV3	0.30080824
13917	0.7535349	2.5051145	ULK4	0.300798595
13918	1.8371059	6.1101368	OSBPL11	0.300665262
13919	0.5828451	1.9415734	INHBB	0.300192146
13920	1.1058936	3.6839524	JPX	0.300192146
13921	7.4746191	24.899449	SEH1L	0.300192146
13922	0.4237676	1.4116546	LINC00174	0.300192145
13923	11.796198	39.318128	SLC25A22	0.300019323
13924	93.120201	310.5232	SERPINE1	0.299881618
13925	0.6075787	2.0272305	SEMA6A	0.299708745
13926	4.1868986	13.976422	PRR7	0.299568694
13927	13.700094	45.736838	ZFR	0.299541776
13928	1.5251401	5.0922526	HSPA12A	0.299502049
13929	28.120764	93.913245	CDCA4	0.299433421
13930	0.01	0.0333965	SHISA7	0.29943262

13931	0.8940696	2.9870586	LOC100506385	0.299314391
13932	4.1646247	13.91388	PLAA	0.299314391
13933	25.577135	85.456803	MKI67	0.299298998
13934	8.9676118	29.97765	STX1A	0.299143256
13935	4.4992182	15.043049	TPR	0.299089512
13936	2.061982	6.8988165	ZBTB21	0.298889228
13937	41.6799	139.52353	PABPC4	0.298730268
13938	8.6833125	29.105512	ARFIP2	0.298339108
13939	4.3686518	14.644354	SLK	0.298316454
13940	3.0747467	10.308677	MOCS3	0.298267837
13941	4.8939217	16.407809	SLC43A1	0.298267837
13942	17.099155	57.35749	SYNCRIP	0.298115465
13943	17.85032	59.979901	ETS1	0.29760502
13944	8.5931743	28.883875	GFM1	0.297507664
13945	9.6663864	32.496323	TNPO1	0.297460929
13946	3.5280991	11.872253	PCNX	0.29717184
13947	57.708914	194.20706	SSR3	0.297151474
13948	0.1400534	0.4713297	NOS1AP	0.297145325
13949	4.8060439	16.196828	OTUD4	0.296727487
13950	2.275706	7.6697559	PIDD	0.296711657
13951	8.6115496	29.024556	TMEM48	0.296698757
13952	12.198995	41.141474	DHCR7	0.29651332
13953	2.0988405	7.0796714	PRRX1	0.296460153
13954	0.01	0.0337319	CCDC108	0.296455662
13955	205.25493	692.93016	RPS3	0.296213006
13956	9.0871875	30.700893	VRK1	0.295990983
13957	5.0200101	16.970106	RIN2	0.295814891
13958	24.790651	83.866829	FEZ2	0.29559543
13959	0.1640941	0.5554468	KIF9-AS1	0.295427192
13960	0.3646535	1.2343263	LOC100507547	0.295427191
13961	0.702273	2.3771439	FAM72B	0.295427191
13962	0.4967807	1.6815673	ACY1	0.295427191
13963	8.0795878	27.348829	RIN3	0.295427191
13964	15.2074	51.475967	RBM25	0.295427191
13965	0.5082915	1.7205304	SUGT1P1	0.295427191
13966	0.0774144	0.2620421	PCDH10	0.295427191
13967	0.2228872	0.7544572	COL17A1	0.295427191
13968	4.3972766	14.884468	PRRX2	0.295427191
13969	1.794438	6.0740447	ZNF354A	0.295427191
13970	0.5924648	2.0054512	PRR22	0.295427191
13971	0.1445713	0.4893635	PRKXP1	0.295427191
13972	0.2111444	0.7147087	NRL	0.295427191
13973	0.2044134	0.6919249	ENTPD3-AS1	0.295427191

13974	0.1702958	0.5764392	SCAND3	0.295427191
13975	0.2910608	0.9852199	LOC100630918	0.295427191
13976	0.200577	0.6789389	FOXD3	0.295427191
13977	33.355482	113.10161	TMEM123	0.294916071
13978	0.01	0.0339249	GRIN2B	0.294768713
13979	47.990674	163.02517	OXA1L	0.294375849
13980	13.070352	44.417775	VKORC1L1	0.294259495
13981	4.9935982	16.971966	LOC100506710	0.294226268
13982	41.77351	142.02277	MRPL3	0.29413249
13983	0.7690997	2.6161721	ADAMTSL1	0.293979019
13984	13.988951	47.594431	SETD3	0.293919909
13985	8.0325289	27.333398	LARS2	0.293872311
13986	0.01	0.0340798	PART1	0.293429081
13987	3.0179806	10.292459	CITED4	0.29322251
13988	4.4645002	15.234215	TCFL5	0.293057454
13989	8.296302	28.344029	TRMT6	0.292700171
13990	0.9787296	3.3467355	NPAT	0.292443078
13991	1.531483	5.2409276	PMS1	0.292216026
13992	3.7137677	12.70898	TIMM8A	0.292216026
13993	12.01932	41.153258	DPAGT1	0.292062417
13994	20.207915	69.192411	PAPOLA	0.292053924
13995	3.467078	11.871748	ATE1	0.292044437
13996	2.002237	6.8581133	FLJ31306	0.291951577
13997	5.55349	19.029673	LANCL2	0.291833186
13998	3.9498875	13.536176	ISYNA1	0.291802318
13999	3.6894761	12.650804	PDCD4	0.291639663
14000	0.01	0.0343177	RGPD3	0.291394828
14001	5.326121	18.280281	FANCD2	0.291358808
14002	1.4484761	4.9730312	PELI2	0.291266245
14003	23.630728	81.177558	TRNP1	0.291099277
14004	24.151864	83.0243	SLBP	0.290901148
14005	6.0091165	20.663296	C14orf169	0.290811141
14006	1.9456385	6.690385	OMA1	0.290811141
14007	0.7499382	2.5787809	AMIGO1	0.290811141
14008	3.2407935	11.155785	ZNF839	0.290503404
14009	4.6728981	16.087446	SMU1	0.290468607
14010	1.5328001	5.2773635	ZNF589	0.290448081
14011	1.5913881	5.480012	AHCTF1	0.290398643
14012	0.0772422	0.2660112	MARVELD3	0.290372008
14013	1.5934968	5.4901923	ACBD5	0.290244258
14014	12.002268	41.391956	PFAS	0.289966183
14015	4.8481828	16.725672	AASDHPPT	0.289864754
14016	0.01	0.0345294	EPHA3	0.289608653

14017	0.6618859	2.2861598	NIPAL1	0.289518647
14018	6.4806597	22.384257	WNT5B	0.289518647
14019	0.6417934	2.2167603	IQCG	0.289518647
14020	17.732687	61.300988	PTPRF	0.289272458
14021	20.292064	70.217935	WWTR1	0.288986911
14022	12.445631	43.067923	FGFRL1	0.288976816
14023	2.2845665	7.9280469	RNMT	0.288162588
14024	0.01	0.0347257	ATP8B4	0.287971323
14025	5.975137	20.771178	BCL9	0.287664807
14026	2.51002	8.7258671	NUBPL	0.287652791
14027	7.1330079	24.797284	CTR9	0.287652791
14028	11.54791	40.171049	NIP7	0.287468484
14029	4.0958671	14.251728	SOWAHC	0.287394427
14030	7.1663322	24.93822	TRMT61A	0.287363422
14031	0.463522	1.6138173	LOC100129534	0.28722088
14032	0.5019256	1.7475247	SYNGR3	0.28722088
14033	21.13877	73.640424	BZW2	0.287053891
14034	15.198784	53.011799	RRP1B	0.286705686
14035	1.182044	4.1253937	ZNF202	0.286528782
14036	0.7935711	2.7704171	CRYBG3	0.286444607
14037	1.8811932	6.5673891	SGOL2	0.286444607
14038	0.9406922	3.2891489	CEP135	0.285998663
14039	2.928706	10.243263	NIPBL	0.285915331
14040	3.1463512	11.008164	MSANTD2	0.285819803
14041	5.370446	18.790864	TTLL5	0.285800912
14042	1.5550359	5.4427229	AMER1	0.285709191
14043	0.01	0.0350092	SORCS3	0.285639367
14044	0.8401288	2.9434233	ATM	0.28542575
14045	0.64197	2.2506306	LOC100287042	0.285240047
14046	0.01	0.0350945	KLHL31	0.284944748
14047	4.0371852	14.173163	TBRG1	0.284847154
14048	2.3308778	8.1888877	JMJD1C	0.284639119
14049	4.74957	16.686287	DBNDD1	0.284639119
14050	2.0880627	7.3433178	CASC5	0.284348671
14051	2.9568601	10.409111	FUZ	0.284064607
14052	1.4440098	5.0833853	ZNF778	0.284064607
14053	2.4455935	8.6160438	PHF6	0.283841811
14054	3.3640412	11.855756	UTP15	0.283747511
14055	2.6328411	9.2897245	RFX7	0.283414338
14056	1.8171595	6.4130262	DTNA	0.283354445
14057	8.2417881	29.117889	CDC25A	0.283048957
14058	2.2614303	7.9912544	MIS18BP1	0.282988151
14059	7.0848918	25.06814	ALKBH2	0.282625346

14060	0.4255946	1.5092078	C21orf88	0.281998683
14061	0.2208402	0.7831248	FAM211A	0.281998683
14062	0.3069212	1.0883782	LY86-AS1	0.281998682
14063	1.1664151	4.1362431	DDI2	0.281998682
14064	3.634875	12.889688	ARHGAP24	0.281998682
14065	15.018329	53.256735	SLMO2	0.281998682
14066	0.237267	0.8413762	ZFP82	0.281998682
14067	0.2221743	0.7878558	RFESD	0.281998682
14068	0.1218236	0.4320005	C6orf170	0.281998682
14069	34.668419	122.96958	DHCR24	0.28192679
14070	11.988966	42.648694	ARRB2	0.281109798
14071	1.0496827	3.7343818	ZCCHC2	0.281086065
14072	2.328915	8.2916037	EPT1	0.2808763
14073	1.5480436	5.5122259	GDAP2	0.280838194
14074	19.165157	68.295832	CXCL1	0.280619716
14075	15.006354	53.486589	ABCE1	0.28056293
14076	0.7424609	2.6478118	DUSP8	0.280405469
14077	0.4311943	1.5397575	MIPOL1	0.280040358
14078	3.2476828	11.617572	RLIM	0.279549182
14079	6.9815578	24.98248	EPB41L4A-AS1	0.279458154
14080	1.2331331	4.412586	LOC440288	0.279458154
14081	2.6434438	9.4591759	NLN	0.279458154
14082	4.4631784	15.98365	TPP2	0.279233989
14083	18.957632	67.941129	GOLM1	0.279030275
14084	4.8113963	17.244248	NDC80	0.279014569
14085	1.4242378	5.1045284	SNTB1	0.279014569
14086	4.5003955	16.160026	SLC25A36	0.278489365
14087	0.7997788	2.8729408	TBX20	0.278383315
14088	0.7371382	2.6479252	FAM76B	0.278383315
14089	12.960633	46.608505	NRAS	0.278074429
14090	5.4730172	19.722154	TRIM33	0.277506056
14091	0.1609489	0.5801771	ANK2	0.277413337
14092	14.158439	51.079302	BCAS2	0.277185452
14093	0.01	0.0361003	VWDE	0.277006185
14094	0.9717104	3.5084487	ANKLE1	0.276962991
14095	2.5694942	9.2861111	MIR100HG	0.276702932
14096	5.4673468	19.768933	OSBPL10	0.276562563
14097	5.5028159	19.919101	POLM	0.276258251
14098	0.1833696	0.6650281	CDS1	0.275732045
14099	0.5448353	1.9759593	MCOLN2	0.275732045
14100	0.191017	0.692763	PCDH7	0.275732045
14101	1.5542536	5.6368261	ZC3H8	0.275732045
14102	1.9771076	7.1703948	MEIS2	0.275732045

14103	2.4387176	8.8445199	CYB5D1	0.275732045
14104	0.5825283	2.1126609	APITD1	0.275732045
14105	0.145125	0.5263262	CXADR	0.275732044
14106	38.024803	138.11872	RPS29	0.275305215
14107	29.958011	108.94234	FARSB	0.274989602
14108	30.169805	109.87302	EIF3A	0.274587929
14109	1.1647436	4.2458489	KLHL11	0.274325249
14110	5.7432933	20.94828	TAMM41	0.274165386
14111	1.3064629	4.7652366	COL27A1	0.274165386
14112	14.321542	52.245602	NOP58	0.274119577
14113	2.5283531	9.2307325	ARHGAP22	0.273906005
14114	85.696991	312.91716	IMPDH2	0.273864785
14115	0.01	0.0365189	LRP1B	0.273830758
14116	1.3454774	4.9158012	ZNF711	0.273704603
14117	1.1422628	4.1733415	HSPA4L	0.273704603
14118	0.01	0.0365454	STRC	0.273632294
14119	4.488444	16.40447	PDE12	0.273611029
14120	2.4837184	9.0827894	IL27RA	0.273453268
14121	9.2081787	33.674425	SLC38A1	0.273447241
14122	1.1076663	4.0554426	KIF16B	0.273130799
14123	1.1299741	4.1371169	MRE11A	0.273130799
14124	1.3878496	5.0825421	VPS13A	0.273062104
14125	0.01	0.0366317	C1orf116	0.272987291
14126	58.857265	215.62371	STOML2	0.272962856
14127	4.8083489	17.616288	C10orf2	0.272949035
14128	1.5461111	5.6663198	LSM11	0.272859836
14129	2.6861375	9.8500828	PUS3	0.272702022
14130	5.2372649	19.205083	BCL2A1	0.272702022
14131	48.218509	176.85968	ATP5L	0.272637093
14132	12.92029	47.390355	NUP155	0.272635445
14133	24.879235	91.355021	CSE1L	0.27233571
14134	0.2847766	1.0465726	C11orf45	0.272103992
14135	0.4788591	1.7598387	CADM4	0.272103992
14136	0.2922854	1.074168	MMP25	0.272103992
14137	1.1143167	4.0951867	ZBTB44	0.272103992
14138	0.3487274	1.2815961	KDM4D	0.272103992
14139	0.927044	3.4069476	HAUS3	0.272103992
14140	0.8274747	3.0410237	SPTB	0.272103992
14141	1.6332249	6.0022086	C12orf39	0.272103992
14142	1.4851732	5.4581087	RMI1	0.272103992
14143	2.2554058	8.2887643	MAPK8IP1	0.272103992
14144	4.8571563	17.859231	DCAF16	0.271968953
14145	4.8474554	17.838554	BAG5	0.271740378

14146	0.01	0.0368663	EHF	0.271250724
14147	0.9019183	3.3255122	FZD4	0.271211847
14148	1.6896055	6.2352118	KDM4C	0.270978044
14149	3.1752411	11.719356	ESF1	0.27093991
14150	0.6467308	2.3914951	GPCPD1	0.270429506
14151	4.0809302	15.091656	FBN2	0.270409695
14152	4.1000464	15.181232	CPVL	0.270073365
14153	21.611205	80.028636	DKC1	0.270043398
14154	7.3940871	27.393785	ACP6	0.269918418
14155	0.3619094	1.3417078	LOC613037	0.26973787
14156	1.5688796	5.8163118	LOC100268168	0.26973787
14157	2.2014032	8.1612685	CAMK4	0.26973787
14158	0.276759	1.0260293	KCNAB1	0.26973787
14159	0.1727784	0.6405417	PLEKHG1	0.269737869
14160	3.9735801	14.736559	THADA	0.269640982
14161	4.593253	17.070174	SLC25A15	0.269080614
14162	0.9760254	3.6359022	WRN	0.268441053
14163	2.3065327	8.5923247	STYX	0.268441053
14164	16.105626	60.028851	RSL1D1	0.268298093
14165	0.843235	3.1455444	PSTK	0.268072822
14166	4.1354968	15.426766	LOC400043	0.268072821
14167	0.168355	0.6280198	RAB11FIP4	0.268072821
14168	33.829411	126.37325	APRT	0.267694401
14169	0.01	0.0374068	SYT13	0.267331059
14170	5.1100091	19.12341	PSMG4	0.267212235
14171	1.074465	4.0210174	CEP128	0.267212234
14172	694.73766	2601.7803	RPS4X	0.267023953
14173	9.4365584	35.377261	TTI1	0.266740783
14174	9.9724914	37.465643	TSR1	0.266176974
14175	1.7263478	6.4893493	UNC13B	0.266027883
14176	0.01	0.0376022	PCDHA12	0.265941816
14177	1.136036	4.2726679	LOC100506469	0.265884472
14178	2.8980153	10.899528	CEP57	0.265884472
14179	0.6135412	2.3075481	ZDHHC21	0.265884472
14180	7.2205323	27.210163	SNHG15	0.265361592
14181	3.7820291	14.255595	GPR3	0.265301392
14182	2.31298	8.7240467	ZC3HAV1L	0.265126966
14183	18.685132	70.53446	PFDN4	0.264907853
14184	0.01	0.0377572	LINC00861	0.26485026
14185	11.250889	42.525697	GBAS	0.264566839
14186	1.4029342	5.3038907	SMN2	0.264510392
14187	1.6553472	6.2632623	FXN	0.264294729
14188	1.7067699	6.4627364	APAF1	0.264094004

14189	3.0557111	11.572125	APOOL	0.264057906
14190	0.1680415	0.6365239	DNHD1	0.263998767
14191	1.021149	3.8680069	LBX2-AS1	0.263998766
14192	2.8936646	10.978841	NEK4	0.263567396
14193	4.9383768	18.74586	LOC93622	0.263438259
14194	747.32365	2837.2334	HSPA8	0.26339872
14195	22.269604	84.554374	RAC2	0.263376128
14196	6.2688037	23.807193	MED28	0.26331554
14197	4.5045358	17.125308	QTRTD1	0.263033859
14198	0.4985634	1.8965427	ADAT2	0.262880128
14199	5.2247746	19.92597	SELRC1	0.262209301
14200	36.737477	140.19961	MTHFD1	0.262036934
14201	2.1507422	8.2125368	MTPAP	0.261885239
14202	4.8184856	18.400407	RASSF8	0.261868423
14203	1.8353104	7.0111316	DPF3	0.261770929
14204	2.0487377	7.833364	GTF2H2B	0.261539954
14205	13.217456	50.573121	DUSP7	0.26135338
14206	0.01	0.0382734	KMO	0.261277911
14207	28.668714	109.75621	GMPS	0.261203563
14208	2.2648149	8.6907573	KANK1	0.260600408
14209	1.7653017	6.7745649	BDP1	0.260577871
14210	1.499931	5.7585099	DCLRE1A	0.260472065
14211	0.01	0.0383974	LOC100129316	0.260434436
14212	79.475141	305.38075	HSPD1	0.260249348
14213	0.01	0.0384267	FP588	0.260235972
14214	2.8010686	10.768181	DNAAF2	0.260124571
14215	4.8152491	18.519442	TRMT10C	0.260010481
14216	34.277433	131.89658	DCTPP1	0.259881139
14217	3.2258263	12.425288	NKRF	0.259617835
14218	13.41203	51.707106	NUP153	0.259384669
14219	11.80705	45.544578	PTGFRN	0.259241605
14220	6.0141231	23.200222	DLGAP5	0.259226958
14221	5.7517668	22.214764	ARHGAP11A	0.258916399
14222	0.01	0.0386848	PCDH12	0.258499409
14223	0.0183434	0.0709613	SV2B	0.258498797
14224	0.0320615	0.1240294	SCN11A	0.258498796
14225	0.0716149	0.2770417	PTGDR2	0.258498794
14226	0.0628467	0.2431216	CAPN3	0.258498794
14227	0.0537944	0.2081031	ABCG4	0.258498794
14228	0.0904119	0.3497577	LOC100130357	0.258498793
14229	0.0954647	0.3693043	LOC100288748	0.258498793
14230	0.060546	0.2342218	LOC157273	0.258498793
14231	0.0959041	0.3710039	CCDC7	0.258498793

14232	0.0366063	0.141611	MPPED2	0.258498793
14233	0.0731997	0.2831722	CCDC13	0.258498793
14234	0.0666665	0.2578987	MGC70870	0.258498793
14235	0.1228771	0.4753487	C6orf141	0.258498793
14236	0.1029642	0.3983159	STAG3L1	0.258498793
14237	0.187522	0.7254272	ITGA2B	0.258498793
14238	0.2471385	0.9560526	PAR5	0.258498792
14239	0.2001916	0.7744394	CFLAR-AS1	0.258498792
14240	0.2346841	0.9078732	LOC646626	0.258498792
14241	0.4884363	1.8895111	ANXA2R	0.258498792
14242	0.074455	0.2880284	LOC100129055	0.258498792
14243	0.5448353	2.1076899	C19orf73	0.258498792
14244	0.0871235	0.3370365	AQP4-AS1	0.258498792
14245	0.2280082	0.8820475	RPS15AP10	0.258498792
14246	0.9140329	3.5359271	MT1F	0.258498792
14247	0.1638361	0.6337983	SMCO2	0.258498792
14248	0.0673125	0.2603977	ZNF577	0.258498792
14249	0.2029206	0.7849965	ECSCR	0.258498792
14250	0.0795114	0.3075892	ZNF876P	0.258498792
14251	1.6015331	6.195515	COMTD1	0.258498792
14252	1.3427803	5.1945321	PLS1	0.258498792
14253	0.9057566	3.5039104	CNTLN	0.258498792
14254	0.1688129	0.6530509	LOC100499405	0.258498792
14255	1.5211642	5.8846087	SNORA4	0.258498792
14256	0.2952818	1.1422949	PRTG	0.258498792
14257	0.9723149	3.7613906	FAHD2B	0.258498792
14258	0.1319402	0.5104092	PPARGC1A	0.258498792
14259	0.4110444	1.5901211	ARVCF	0.258498792
14260	0.0735614	0.2845716	RHD	0.258498792
14261	2.2873116	8.8484421	RNF144A	0.258498792
14262	1.0312166	3.9892511	ACTR3B	0.258498792
14263	0.3381736	1.3082213	DGAT2	0.258498792
14264	0.761045	2.9440952	FAM86B3P	0.258498792
14265	3.6086494	13.960024	MSMP	0.258498792
14266	0.2270147	0.8782041	CCDC19	0.258498792
14267	2.6049938	10.077392	SNORD104	0.258498792
14268	3.9718785	15.365172	PDSS1	0.258498792
14269	0.171805	0.664626	AGAP5	0.258498792
14270	0.296022	1.1451582	NACAP1	0.258498792
14271	0.1660774	0.6424689	WDR17	0.258498792
14272	0.1370148	0.5300404	OTUD7A	0.258498792
14273	0.0684624	0.2648461	MICALCL	0.258498792
14274	0.2137431	0.826863	ZNF565	0.258498792

14275	0.0594069	0.2298151	CPB2-AS1	0.258498792
14276	0.1229133	0.4754889	OPRL1	0.258498792
14277	0.1866264	0.7219624	ATP1B2	0.258498792
14278	0.3506441	1.3564634	CCT6P1	0.258498792
14279	0.1250897	0.4839084	LINC00693	0.258498792
14280	0.0815654	0.3155348	PSMD6-AS2	0.258498792
14281	0.2051176	0.7934955	LOC91450	0.258498792
14282	0.2083995	0.8061914	CYP2B7P1	0.258498792
14283	0.3225998	1.2479743	C14orf178	0.258498792
14284	0.0644402	0.2492861	TECTA	0.258498792
14285	0.1126788	0.4358969	LOC100294362	0.258498792
14286	0.1760131	0.6809049	MESP1	0.258498792
14287	0.1606781	0.6215816	PLXDC2	0.258498791
14288	0.0729435	0.2821811	TSC22D1-AS1	0.258498791
14289	0.1195637	0.4625309	GPR19	0.258498791
14290	0.0509161	0.1969683	PITRM1-AS1	0.258498791
14291	0.0458525	0.1773798	FGF9	0.258498791
14292	0.0909247	0.3517414	FBXO24	0.258498791
14293	0.0330269	0.1277641	ABCA9	0.258498791
14294	0.0761416	0.2945529	LOC100996307	0.258498791
14295	0.0424266	0.1641269	NAALADL2	0.258498791
14296	0.0893268	0.34556	CEACAM8	0.258498791
14297	0.0342537	0.1325101	SLC12A5	0.258498791
14298	0.0967051	0.3741027	AGXT2L1	0.25849879
14299	0.012253	0.0474007	KSR2	0.25849879
14300	0.0398393	0.154118	PTCHD2	0.25849879
14301	0.0480515	0.1858869	POTEF	0.25849879
14302	0.0381474	0.147573	SPOCK2	0.25849879
14303	0.038514	0.1489912	GUCY1A2	0.258498789
14304	2.6138043	10.139879	UACA	0.257774706
14305	10.595441	41.118894	ALDH1B1	0.257678161
14306	4.1295753	16.037141	OPA1	0.257500727
14307	1.5890388	6.1756401	CHML	0.257307553
14308	0.01	0.0389314	LRRC37A	0.256862083
14309	6.157258	23.991065	AGO2	0.256647965
14310	0.01	0.0389992	ABCC12	0.256415543
14311	2.069166	8.0712525	KIAA0586	0.256362438
14312	8.9639519	35.012233	ZC3H14	0.256023428
14313	7.8567813	30.707866	DNMBP	0.255855655
14314	5.0998765	19.953013	C16orf74	0.255594311
14315	1.5431285	6.0441968	ANAPC4	0.255307449
14316	9.2715047	36.32952	PAK1	0.255205814
14317	20.584824	80.916577	EIF5	0.254395637

14318	2.4868675	9.7807627	GPATCH11	0.254261107
14319	2.1708281	8.5409717	ERCC6L	0.25416641
14320	3.4106013	13.421358	CDK8	0.254117457
14321	1.880979	7.4020062	MREG	0.254117457
14322	12.483165	49.129384	PNN	0.25408755
14323	12.854831	50.606354	CLNS1A	0.254016154
14324	0.9160418	3.6069787	ENTPD1	0.253963726
14325	6.3462454	25.04681	4-Mar	0.253375393
14326	2.0639137	8.146072	E2F8	0.253363054
14327	4.5143649	17.820989	PIK3R4	0.253317302
14328	4.3643874	17.235331	C14orf80	0.253223307
14329	2.2917468	9.0583302	TOPORS	0.252998818
14330	3.7073766	14.667903	MCM8	0.252754375
14331	9.0479075	35.807701	WDR43	0.252680491
14332	1.7928267	7.0960768	SIX4	0.252650403
14333	3.4473189	13.65177	FBXO45	0.2525181
14334	4.2372528	16.785803	SCUBE3	0.252430745
14335	0.3539394	1.4034412	RNF125	0.252193944
14336	5.5507413	22.009812	CLPB	0.252193944
14337	12.481277	49.518221	SRPK1	0.252054224
14338	0.01	0.0396826	SYK	0.251999712
14339	6.3569344	25.229731	SLC25A23	0.251962041
14340	4.031538	16.045848	MRPS31	0.251251162
14341	1.1666872	4.654359	DNA2	0.250665495
14342	4.0619285	16.204578	IQSEC1	0.250665495
14343	2.5200781	10.066796	HOXD9	0.250335672
14344	8.9314072	35.702762	SFXN4	0.250160121
14345	2.8534626	11.412781	PDE4B	0.250023422
14346	2.3581927	9.4356769	ZFPM2	0.249923003
14347	3.2023564	12.817109	GUF1	0.249850134
14348	14.56682	58.305724	MSH6	0.249835154
14349	17.248805	69.339995	PREX1	0.248756945
14350	0.01	0.0402131	KL	0.248675443
14351	1.3994057	5.6301306	SLC25A51	0.248556531
14352	20.312154	81.825642	BRIX1	0.248237025
14353	0.4111795	1.6569208	C1orf170	0.248158841
14354	0.516693	2.0821059	MAPK11	0.248158841
14355	1.1728886	4.7263624	BRWD3	0.24815884
14356	1.0308302	4.1539128	NSUN6	0.24815884
14357	6.7945808	27.411258	TFAP4	0.247875554
14358	19.190149	77.508132	DHX15	0.247588852
14359	3.5102753	14.204835	RLTPR	0.247118342
14360	0.01	0.0404715	ABCC8	0.247087731

14361	16.288697	65.977962	NCBP2	0.246880869
14362	1.7794031	7.2075373	KIF21B	0.246880869
14363	42.932086	174.29996	TOMM5	0.246311504
14364	1.337037	5.43093	ADAP1	0.246189326
14365	1.8817111	7.6433495	C2CD5	0.246189326
14366	3.6129755	14.695423	CMTM4	0.245857198
14367	9.5094047	38.695798	CSTF1	0.245747731
14368	3.4692909	14.123175	STAG1	0.245645261
14369	1.8365448	7.4785843	HOMER1	0.245573852
14370	0.6769357	2.7590077	FGFR3	0.245354786
14371	2.0114208	8.2017645	DIS3	0.245242444
14372	0.01	0.0408322	HFM1	0.244904625
14373	1.9639743	8.0197049	SPA17	0.244893593
14374	43.137515	176.21149	ATP5F1	0.244805343
14375	43.737629	178.7139	DYNC1H1	0.244735467
14376	1.6836962	6.8797387	XPO4	0.244732584
14377	1.9967102	8.1656396	LOC90784	0.244525884
14378	1.4828645	6.0673955	LOC728554	0.244398858
14379	1.5543279	6.3598001	LIAS	0.244398858
14380	3.1652758	12.955476	RPAP3	0.244319533
14381	1.3668177	5.5985513	RASA2	0.244137748
14382	3.8158423	15.657785	ALDH3A2	0.243702565
14383	2.905774	11.935624	PITPNC1	0.243453889
14384	0.5612195	2.306764	TRIM36	0.243292981
14385	1.2246787	5.0337611	GLMN	0.243292981
14386	0.3476222	1.4288213	PPP4R1L	0.243292981
14387	9.6136674	39.589756	MED6	0.242832199
14388	4.1561154	17.13061	FOXRED1	0.242613391
14389	0.8514122	3.5153694	EPHA4	0.242197067
14390	0.01	0.0413516	FLJ45445	0.24182843
14391	0.6033071	2.5005936	RGS14	0.241265539
14392	0.01	0.0414537	ABCC11	0.241233042
14393	3.0380364	12.605626	ARID3B	0.241006393
14394	18.903494	78.478808	NPM3	0.240873874
14395	0.8292857	3.4486898	ARRB1	0.240463993
14396	0.4448228	1.8498522	CYP27C1	0.240463993
14397	1.0089542	4.1958642	B3GNT5	0.240463993
14398	2.3204075	9.659466	ZNHIT6	0.2402211
14399	3.2870584	13.694103	NEMF	0.240034593
14400	3.3843766	14.099537	PRKRIR	0.240034593
14401	1.1060735	4.6108128	SPICE1	0.239886879
14402	10.913317	45.543452	PPAP2C	0.239624277
14403	6.3788108	26.63596	WDR75	0.23948117

14404	2.7371875	11.431072	SLC25A19	0.239451513
14405	1.5856484	6.6269808	WDR89	0.239271609
14406	3.4714586	14.512311	POLE2	0.239207837
14407	7.5164228	31.438807	LETM1	0.239081042
14408	0.01	0.0418583	CDH1	0.238901086
14409	0.1979103	0.829415	SLC2A4	0.23861427
14410	0.4132178	1.7317396	GREB1L	0.23861427
14411	0.2046476	0.8576504	ZCCHC18	0.23861427
14412	0.9067418	3.8000319	HAGHL	0.23861427
14413	21.797323	91.349619	PNP	0.23861427
14414	0.2721805	1.1406713	MARVELD2	0.23861427
14415	2.0859817	8.7420658	HSD11B1L	0.23861427
14416	0.7974471	3.3419924	LOC100134868	0.23861427
14417	0.233196	0.9772928	AOC2	0.23861427
14418	0.1910753	0.8007708	RAMP2-AS1	0.238614269
14419	0.2552873	1.0698742	SYP	0.238614269
14420	2.736803	11.495519	SLC4A7	0.238075637
14421	3.0167614	12.688527	NIN	0.237755062
14422	15.319819	64.498532	SLC29A1	0.237521986
14423	0.01	0.0421032	PCSK2	0.237511841
14424	0.01	0.0421472	KCNT1	0.237263759
14425	142.09057	600.78617	RPS21	0.236507723
14426	1.6376423	6.924524	FOXN2	0.236498895
14427	0.01	0.0422976	VWA3B	0.23642029
14428	0.7146147	3.0236497	ITGAX	0.236341753
14429	21.504216	91.137519	TUBB3	0.235953497
14430	0.3835212	1.6263062	FAM105A	0.23582346
14431	0.6003827	2.5483803	SOBP	0.235593836
14432	4.5025946	19.125913	NFX1	0.235418543
14433	5.2099875	22.144858	VMA21	0.235268499
14434	8.7960828	37.400113	NPAS2	0.235188672
14435	39.751427	169.05954	PAICS	0.235132709
14436	0.3140439	1.3363631	TMEM144	0.234998902
14437	0.7843414	3.3376384	CYP2S1	0.234998902
14438	1.0797902	4.5948732	NOP14-AS1	0.234998902
14439	0.01	0.0425566	MAPK4	0.234981421
14440	0.01	0.0425655	CXorf36	0.234931809
14441	0.8847528	3.7727019	MYO1D	0.234514368
14442	5.3022866	22.638997	FRMD3	0.234210315
14443	0.5407122	2.3096294	PARD6B	0.234112114
14444	1.375196	5.8740918	EXOC6	0.234112114
14445	6.2687675	26.780754	SKIV2L2	0.234077338
14446	2.558476	10.932171	IPO11	0.234031828

14447	1.7356106	7.4161307	RSBN1	0.234031828
14448	9.3076871	39.77918	YAP1	0.233983883
14449	0.361715	1.5492148	TMED10P1	0.23348278
14450	1.5779302	6.7582296	CREB3L4	0.23348278
14451	5.5844409	23.928218	DHX33	0.233383065
14452	0.01	0.0428734	ITPK1-AS1	0.233244861
14453	0.01	0.0428825	SEMA3G	0.233195245
14454	1.4403876	6.1837	PCF11	0.232932977
14455	1.4477773	6.2230134	PLD6	0.232648913
14456	5.5978642	24.094843	BAZ1A	0.232326238
14457	1.5174721	6.5342315	CHST11	0.232234209
14458	1.7223099	7.4198682	ZBTB33	0.232121364
14459	2.7698841	11.935325	C14orf132	0.232074471
14460	3.5398309	15.312159	PGM2	0.231177782
14461	9.0743859	39.320114	YES1	0.230782291
14462	3.9308888	17.047086	RAP1GAP2	0.230590073
14463	3.1958554	13.872587	MPP5	0.230371981
14464	2.4464723	10.62303	CHEK1	0.230298924
14465	20.038413	87.035514	CLUH	0.23023261
14466	12.171127	52.873279	WHSC1	0.23019429
14467	0.183531	0.7987365	CTSL2	0.229776705
14468	0.0882768	0.3841852	SESN3	0.229776704
14469	0.3256242	1.4171333	HLA-DRB1	0.229776704
14470	0.1758646	0.7653716	NEUROG2	0.229776704
14471	3.6401659	15.842189	GPR125	0.229776704
14472	0.2182194	0.9497019	RNF32	0.229776704
14473	22.17765	96.518268	GART	0.229776704
14474	4.9067247	21.354317	FAM216A	0.229776704
14475	2.2217431	9.6691398	ZCCHC4	0.229776704
14476	0.1909295	0.8309348	LRRC48	0.229776704
14477	0.5959948	2.5937997	3-Mar	0.229776704
14478	0.1460403	0.6355749	XKRX	0.229776703
14479	0.0808063	0.3516733	LRP2BP	0.229776703
14480	0.0494365	0.2151501	TRPM6	0.229776702
14481	28.622581	124.70103	SERBP1	0.229529632
14482	6.1293971	26.705389	PPP2R5E	0.229519107
14483	0.01	0.0436062	RAPGEF4-AS1	0.229325198
14484	12.236981	53.435283	CCNB1IP1	0.229005641
14485	6.0974956	26.650018	HK2	0.228798931
14486	3.8203885	16.704585	OTUB2	0.228702981
14487	1.8708776	8.1814895	SFXN2	0.228672008
14488	1.7024838	7.4467177	SLC39A10	0.228622047
14489	7.312889	32.015817	CCDC6	0.228414881

14490	512.6383	2244.6356	RPLP2	0.228383751
14491	5.1772399	22.682428	CEBPZ	0.228248933
14492	1.1703454	5.131132	KCNK6	0.228087169
14493	5.0857994	22.305238	RRP8	0.228009191
14494	33.250733	145.93785	NOLC1	0.227841742
14495	1.2181169	5.3503934	TMEM218	0.227668661
14496	3.1277301	13.780092	TFB2M	0.226974549
14497	2.2697808	10.000155	ACTR5	0.226974549
14498	4.5416719	20.015786	DHX36	0.226904495
14499	45.55952	200.82906	EMP1	0.226857208
14500	2.4481865	10.810644	SBNO1	0.226460741
14501	1.5571569	6.8772242	LRIG2	0.2264223
14502	0.9952221	4.3974076	SNHG4	0.226320178
14503	1.7926839	7.9222511	PHF14	0.226284657
14504	0.8637043	3.8185501	HTR7	0.226186443
14505	93.289203	412.53272	HSP90AA1	0.226137707
14506	0.01	0.0442962	SCUBE2	0.225752846
14507	12.759153	56.568892	EIF2S1	0.2255507
14508	14.064153	62.386736	ERO1L	0.225434993
14509	4.8748421	21.637763	PLXNA2	0.225293256
14510	0.01	0.0443938	SCML4	0.225256685
14511	26.913625	119.52757	ANPEP	0.225166667
14512	5.3331722	23.686778	PDS5A	0.225153977
14513	0.1215725	0.5408471	CDH6	0.224781559
14514	0.3604281	1.6034592	TMC6	0.224781558
14515	0.3315296	1.4748968	ALG10	0.224781558
14516	1.4230079	6.3306254	C11orf63	0.224781558
14517	0.2777179	1.2355012	GARNL3	0.224781558
14518	16.892039	75.148688	GNL3	0.224781558
14519	0.5537631	2.4635609	SEPSECS	0.224781558
14520	5.9470918	26.516611	U2SURP	0.224277974
14521	13.542825	60.458828	NUMB	0.224000781
14522	3.591698	16.062191	TMEM33	0.223611963
14523	1.0578655	4.7317705	CCDC66	0.223566523
14524	0.4537823	2.0297415	VSIG10L	0.223566523
14525	0.0479079	0.2142986	WDR72	0.223556886
14526	1.5713017	7.033768	USP28	0.223394018
14527	22.364535	100.28626	TBX15	0.223006967
14528	0.8417181	3.7829128	ADAMTSL4	0.222505289
14529	3.2669262	14.695432	PRMT3	0.222308961
14530	10.778062	48.621104	NOP14	0.221674564
14531	6.3482136	28.641741	SPRED1	0.221642029
14532	0.227759	1.0279307	ESR2	0.221570394

14533	0.5567217	2.5126179	FRMD6-AS1	0.221570393
14534	0.8540963	3.8547402	SAMD10	0.221570393
14535	0.3208614	1.4481241	ZNF486	0.221570393
14536	0.6334331	2.8588347	CHIC1	0.221570393
14537	0.282002	1.2727424	NPEPL1	0.221570393
14538	0.2163317	0.9763564	PALM	0.221570393
14539	7.0153316	31.693494	PITPNM1	0.221349265
14540	2.4329616	11.06248	RAD18	0.219929131
14541	5.847246	26.596187	MCM10	0.219852793
14542	3.7992402	17.290968	FSD1	0.219723973
14543	0.7703395	3.5059421	CAND2	0.219723973
14544	3.885169	17.713619	FAM208B	0.219332308
14545	7.7115339	35.198112	BUD13	0.219089416
14546	1.1447663	5.2337019	AP1S3	0.218729747
14547	0.01	0.045744	UNC13D	0.218608139
14548	1.2707287	5.8170314	CPE	0.218449684
14549	1.0325697	4.732597	SEMA4D	0.218182467
14550	2.4188748	11.098908	SLC12A6	0.217938092
14551	7.9707386	36.591839	SMC3	0.217828315
14552	0.100871	0.4633845	CACNA1G	0.217683194
14553	0.1138951	0.5232148	PDE3A	0.217683194
14554	0.3413587	1.5681446	SHC3	0.217683194
14555	1.4463817	6.6444345	ZNF121	0.217683193
14556	0.6309162	2.8983229	JAK2	0.217683193
14557	4.7775379	21.987038	DCLRE1B	0.21728884
14558	1.3853043	6.3896198	TMCC3	0.216805438
14559	1.9158279	8.8524591	SMCO4	0.216417593
14560	3.5016719	16.20704	FLI1	0.216058692
14561	0.2066635	0.959371	RTEL1	0.215415661
14562	0.7086008	3.2894582	GCH1	0.21541566
14563	0.9654084	4.4816075	APLN	0.21541566
14564	1.5722331	7.2986018	TRMT5	0.21541566
14565	5.1383214	23.853054	PDZD8	0.21541566
14566	0.4526488	2.1012808	DMRTA1	0.21541566
14567	0.3472168	1.6118455	CABP7	0.21541566
14568	14.797166	68.804022	PYGL	0.21506252
14569	5.9796447	27.827345	SKP2	0.21488377
14570	1.1253778	5.2440039	L2HGDH	0.214602771
14571	6.177283	28.805781	DLAT	0.214445943
14572	0.01	0.0466546	KIAA0087	0.214341164
14573	0.7509892	3.510443	TNFRSF25	0.213930035
14574	3.6668534	17.140433	RPPH1	0.213930035
14575	0.4635392	2.1667795	LPCAT2	0.213930035

14576	0.01	0.046752	FAM205A	0.21389462
14577	51.128587	239.12202	CCT6A	0.213817983
14578	0.3938926	1.8461056	CPEB2	0.213364082
14579	1.0609429	4.9837286	RGS2	0.212881358
14580	0.0851305	0.4005323	ZNF573	0.212543452
14581	0.1913458	0.9021423	CCDC144B	0.212101573
14582	2.8728908	13.558774	HOXB3	0.211884256
14583	4.8830478	23.06971	PPAT	0.211664893
14584	1.6334944	7.7326526	ASRGL1	0.211246325
14585	6.1230781	29.08938	MSX1	0.210491874
14586	3.8000455	18.056451	AHNAK2	0.210453617
14587	10.83288	51.50296	LRPPRC	0.210335102
14588	0.01	0.0475461	GRM7	0.210322268
14589	4.140837	19.695229	IRX2	0.210245684
14590	0.01	0.047591	BMS1P5	0.210123803
14591	0.7968216	3.7938418	FBXO32	0.210030269
14592	0.01	0.0476247	LOC100507351	0.209974953
14593	1.3322342	6.3501377	SETMAR	0.209796121
14594	1.8395921	8.7684751	TCEA3	0.209796121
14595	0.4108418	1.9618383	CDON	0.209416743
14596	9.6458349	46.165125	IRF2BPL	0.208942029
14597	0.9633934	4.6162461	DDX58	0.208696273
14598	0.01	0.0479334	MUC4	0.208622919
14599	1.7117002	8.2179967	PLEKHG4	0.208286797
14600	3.0862569	14.849323	ZCCHC7	0.207838225
14601	1.1965522	5.7585099	OSBPL6	0.207788503
14602	6.9774742	33.65906	NELFA	0.207298548
14603	0.0449913	0.2175603	PCDHB19P	0.206799036
14604	0.0159559	0.0771564	PLXNA4	0.206799035
14605	0.0768435	0.3715853	LOC100507462	0.206799035
14606	0.0704052	0.3404524	ZSCAN18	0.206799035
14607	0.0856202	0.414026	LOC100506178	0.206799035
14608	0.0904119	0.4371971	LOC100131347	0.206799035
14609	0.1592051	0.7698543	GALR2	0.206799034
14610	0.1357651	0.6565076	C11orf93	0.206799034
14611	0.1493903	0.7223937	IL17RE	0.206799034
14612	0.1269954	0.6141007	DDTL	0.206799034
14613	0.0535181	0.2587928	CCDC39	0.206799034
14614	0.2100096	1.0155249	HCAR1	0.206799034
14615	0.2155114	1.0421295	GRAPL	0.206799034
14616	0.0715166	0.3458268	VWA7	0.206799034
14617	0.3839696	1.8567282	EGR3	0.206799034
14618	0.4895838	2.3674375	HSD17B14	0.206799034

14619	0.2727742	1.3190304	TSACC	0.206799034
14620	0.2598498	1.2565327	HOXA6	0.206799034
14621	0.2096574	1.0138222	LRRC6	0.206799034
14622	0.1381044	0.6678192	KLF14	0.206799034
14623	0.0996174	0.4817109	LINC00327	0.206799034
14624	0.3033472	1.4668693	BMI1	0.206799034
14625	0.8591825	4.1546739	IFT74	0.206799034
14626	0.1135074	0.5488776	C22orf26	0.206799034
14627	0.9460418	4.5746917	LOC100129269	0.206799034
14628	0.0506562	0.2449536	MFSD4	0.206799034
14629	0.7899905	3.8200881	FLI1-AS1	0.206799034
14630	0.172231	0.8328423	C19orf57	0.206799034
14631	0.1268408	0.6133532	ZNF141	0.206799034
14632	0.8880661	4.2943433	ARL6	0.206799034
14633	0.9648125	4.6654594	SLC43A2	0.206799034
14634	0.6157745	2.9776468	DGKE	0.206799034
14635	1.4851924	7.1818151	MTBP	0.206799034
14636	8.1677724	39.496183	UTP14A	0.206799034
14637	0.1775124	0.858381	IL18R1	0.206799034
14638	2.2575776	10.916771	CHST2	0.206799034
14639	3.0947161	14.964848	HELLS	0.206799034
14640	1.0182386	4.9238073	PRRT3-AS1	0.206799034
14641	0.1736663	0.8397827	GUSBP2	0.206799034
14642	0.0851479	0.4117423	GPR64	0.206799034
14643	0.803597	3.8858839	PXN-AS1	0.206799034
14644	0.1831279	0.8855354	SLC2A1-AS1	0.206799034
14645	0.0729946	0.3529735	GDAP1L1	0.206799034
14646	0.1726591	0.8349124	ZNF823	0.206799033
14647	0.0783751	0.3789918	RPL36A-HNRNPH2	0.206799033
14648	0.0457418	0.2211895	KCNS1	0.206799033
14649	0.0848878	0.4104844	TSNAXIP1	0.206799033
14650	0.1941309	0.9387417	TCP11	0.206799033
14651	0.1326878	0.6416269	EML6	0.206799033
14652	0.0486801	0.2353981	SLITRK3	0.206799033
14653	0.1350612	0.6531038	FAM95B1	0.206799033
14654	0.1005789	0.4863606	LSP1	0.206799033
14655	0.0913232	0.4416035	MYH16	0.206799033
14656	0.0522305	0.2525662	TLR10	0.206799033
14657	0.0542283	0.2622272	C1QTNF3	0.206799032
14658	0.0409108	0.1978287	NR5A2	0.206799032
14659	0.0365934	0.1769516	CASKIN1	0.206799032
14660	10.259247	49.675889	MAPKAPK3	0.206523669
14661	13.289584	64.490761	HHEX	0.206069584

14662	0.01	0.0486243	ADCY8	0.205658361
14663	6.7334249	32.777302	EXOSC3	0.205429504
14664	16.767776	81.900106	PLEK2	0.204734484
14665	3.3522708	16.390397	RPP25L	0.204526517
14666	0.01	0.0489194	C20orf26	0.204417963
14667	2.8649155	14.032378	PIGW	0.204164651
14668	1.5803804	7.7440022	RPL22L1	0.204077994
14669	12.468346	61.189295	EGFR	0.20376679
14670	0.01	0.049218	PTPRVP	0.203177563
14671	3.7471141	18.467488	POU2F2	0.20290329
14672	3.1524506	15.570687	LOC339166	0.202460592
14673	0.7346633	3.6314927	SLCO3A1	0.202303403
14674	5.0764547	25.102761	KIAA0040	0.202226946
14675	3.8260942	18.975907	HEATR1	0.201629058
14676	1.3364654	6.6349655	OSGEPL1	0.20142763
14677	0.01	0.0496546	NLRP11	0.201391385
14678	0.7287854	3.6202363	YOD1	0.201308794
14679	0.01	0.0497649	IL2RB	0.200944839
14680	5.3105664	26.458019	TIPIN	0.200716709
14681	2.5486298	12.703392	HMGN5	0.200625928
14682	0.01	0.0498634	TSPEAR	0.200547913
14683	0.8258897	4.1268053	PLLP	0.200128097
14684	0.01	0.0500057	DLG2	0.199977327
14685	1.9298917	9.6664966	MAGI1	0.199647482
14686	3.4330934	17.223651	BAG1	0.19932437
14687	4.1035063	20.598889	THOC1	0.199210078
14688	2.5271375	12.689318	SPTLC2	0.199154711
14689	9.1720239	46.072687	BUB1B	0.199077249
14690	166.67383	837.54492	NCL	0.199002858
14691	0.4568161	2.297345	NOTUM	0.198845225
14692	0.4854403	2.441297	BOC	0.198845225
14693	1.4614271	7.3495709	GCHFR	0.198845225
14694	1.1892686	5.9808758	CC2D2A	0.198845225
14695	0.1128008	0.5676262	ANKS1B	0.198723757
14696	12.989048	65.533622	IPO4	0.198204333
14697	0.01	0.0506084	STAB1	0.197595761
14698	0.8651506	4.380405	MSANTD4	0.197504695
14699	3.2074798	16.264677	UPF3B	0.197205264
14700	0.1397248	0.7094376	IQCH-AS1	0.196951461
14701	0.515362	2.6166954	CCDC41	0.196951461
14702	0.4541284	2.3057882	JHDM1D	0.196951461
14703	0.0698565	0.3546891	CUBN	0.19695146
14704	12.30403	62.720302	DNPH1	0.196172996

14705	6.4097638	32.743578	CPSF2	0.195756367
14706	0.1625038	0.8307085	SRGAP3	0.195620708
14707	9.025678	46.197484	NOL6	0.195371636
14708	12.50397	64.159398	MRPS17	0.194889142
14709	2.2882756	11.749246	TBC1D14	0.194759364
14710	5.2888272	27.194981	STK4	0.194478063
14711	40.249707	207.08053	ZFAS1	0.194367412
14712	2.6775096	13.810559	C9orf40	0.193874094
14713	0.1463137	0.7546842	HES2	0.193874094
14714	0.2241658	1.1562444	LRRC56	0.193874094
14715	0.1850795	0.9546375	MAFB	0.193874094
14716	7.4187524	38.374536	POLR1E	0.193324876
14717	0.01	0.0517586	SLC44A5	0.193204742
14718	0.01	0.0517852	DISC2	0.193105511
14719	3.6772653	19.100138	PDGFRA	0.192525585
14720	0.01	0.0519721	PROX2	0.192410887
14721	8.0947884	42.17886	MELK	0.19191577
14722	0.01	0.0521469	SLC11A1	0.19176588
14723	0.3142333	1.6410711	ADAM8	0.191480587
14724	0.4464428	2.3315304	CSPG5	0.191480587
14725	4.1088532	21.502894	POLR1B	0.191083736
14726	2.3687613	12.400646	CMBL	0.191019188
14727	6.5733487	34.454636	DCAF4	0.190782708
14728	14.052384	73.657276	ERRFI1	0.190780658
14729	0.01	0.0525001	SLC6A10P	0.190475864
14730	0.86396	4.535871	PITPNM2	0.190472794
14731	0.1251219	0.6569018	PDZD2	0.190472794
14732	0.2470861	1.2972253	LOC100129550	0.190472794
14733	2.4185629	12.734809	MARS2	0.18991748
14734	2.4536013	12.924912	CA12	0.189835051
14735	3.1920192	16.830895	NCAPG	0.18965238
14736	13.628666	71.874735	TJP2	0.189616918
14737	0.7760508	4.0990825	ATXN3	0.189323059
14738	19.924644	105.46987	TOP1	0.18891314
14739	0.01	0.0529414	KLHL32	0.188888148
14740	3.261095	17.271239	SNAPC1	0.188816509
14741	0.01	0.0531088	NUTM1	0.188292758
14742	0.01	0.0531368	LOC100506810	0.188193525
14743	26.178685	139.20747	WDR77	0.188055174
14744	66.054061	351.25032	RPL27A	0.188054092
14745	0.0704648	0.3748143	NR3C2	0.187999122
14746	0.095618	0.5086089	CCDC171	0.187999122
14747	0.0968624	0.5152281	DKFZP434L187	0.187999122

14748	0.085296	0.4537044	SLC24A4	0.187999122
14749	0.5107831	2.716944	CCL28	0.187999122
14750	0.4669145	2.4835993	IRF8	0.187999122
14751	0.5455484	2.9018669	TEX40	0.187999122
14752	0.9025531	4.8008366	MPV17L	0.187999122
14753	1.5718378	8.3608786	ECHDC2	0.187999122
14754	0.0802154	0.4266794	DNAH5	0.187999121
14755	0.2870517	1.5268776	SPAG4	0.187999121
14756	0.1398185	0.743719	GPER	0.187999121
14757	0.0923347	0.4911445	DENND2C	0.187999121
14758	0.01	0.0531929	TMEM132B	0.18799506
14759	1.994838	10.630576	FAM217B	0.187650975
14760	1.9432294	10.359293	KIAA1737	0.187583194
14761	0.01	0.0534468	PNMA3	0.187101972
14762	2.5896732	13.841837	BOD1L1	0.187090286
14763	0.01	0.0536032	LOC400680	0.186556197
14764	0.6278813	3.3674113	SRRM3	0.186458145
14765	0.01	0.0537461	ATP12A	0.186060036
14766	29.353463	157.84338	TOMM34	0.185965757
14767	0.01	0.0538322	LRRN3	0.185762342
14768	1.7345975	9.3464519	NUDT8	0.185588876
14769	3.5917772	19.363966	ATP6V0E2	0.185487683
14770	7.314867	39.452443	ARHGEF12	0.185409737
14771	1.6968596	9.1778423	CDC7	0.184886553
14772	0.2634633	1.4268874	TRHDE-AS1	0.184641995
14773	0.2277092	1.2332473	FAM160A1	0.184641994
14774	0.5962134	3.2378946	GAN	0.184136126
14775	3.3971466	18.458294	WEE1	0.184044447
14776	3.8806147	21.098114	KIAA0947	0.183931833
14777	1.2610458	6.8658164	TMEM136	0.183670194
14778	25.522136	139.38653	PSAT1	0.183103311
14779	16.600619	90.756239	GRPEL1	0.18291435
14780	0.7873834	4.3047848	DDHD1	0.182908893
14781	2.9313032	16.027396	UTP20	0.182893296
14782	0.01	0.0547238	ZNF705D	0.182735765
14783	0.01	0.0547982	FLJ31485	0.182487686
14784	0.6915913	3.7901697	SNX10	0.182469736
14785	1.3291491	7.2842166	ARHGEF39	0.182469736
14786	0.6023107	3.30088	HOXA9	0.182469736
14787	0.1348573	0.7390667	JAKMIP2	0.182469735
14788	0.01	0.0548206	GRIN2A	0.18241326
14789	0.01	0.0549177	ANKRD20A8P	0.182090755
14790	2.8632683	15.754111	TFAM	0.181747369

14791	3.6458975	20.098368	ARMCX2	0.181402661
14792	0.7682178	4.2364567	CLMN	0.181334973
14793	3.4068906	18.813768	FOCAD	0.181084968
14794	5.2225417	28.848053	GREM1	0.181036191
14795	0.8362741	4.6370024	NFXL1	0.180347995
14796	0.01	0.0554617	ERN2	0.180304581
14797	0.8481101	4.7163016	NBEAL2	0.179825247
14798	1.834099	10.199341	TBC1D4	0.179825247
14799	0.01	0.0556147	GMNC	0.179808421
14800	1.459741	8.1383144	CEP72	0.179366509
14801	81.436487	454.18902	TM4SF1	0.179300873
14802	0.700412	3.9079857	PPP4R4	0.179225829
14803	2.4073735	13.436523	MDN1	0.179166404
14804	4.8365462	27.071738	EPHB2	0.178656658
14805	0.2780143	1.5594684	USP2	0.178275029
14806	0.472347	2.6495411	ZNF114	0.178275029
14807	0.5706449	3.2009242	ADSSL1	0.178275029
14808	0.6424152	3.6035065	LOC100505761	0.178275029
14809	0.3843658	2.1595677	BRCA2	0.177982775
14810	15.786034	89.025618	TFRC	0.177320122
14811	0.01	0.0565193	FAM13C	0.17693069
14812	1.4731884	8.3293292	C17orf97	0.176867595
14813	1.3508818	7.6378142	ASIC1	0.176867595
14814	0.1429072	0.8095084	EXPH5	0.176535761
14815	0.6364732	3.6053498	TNFAIP8L3	0.17653576
14816	2.8836004	16.339057	CTSC	0.176485119
14817	12.367142	70.109131	LYAR	0.17639845
14818	0.01	0.0567581	GRIK5	0.176186452
14819	1.60258	9.1133601	PROB1	0.175849518
14820	0.01	0.0568702	BCL6B	0.17583914
14821	8.844012	50.354807	FAM60A	0.175633918
14822	0.01	0.0570042	THSD7A	0.175425673
14823	0.01	0.0570957	CNTN6	0.175144517
14824	0.01	0.0571011	EYS	0.175127978
14825	2.554388	14.589379	CADM1	0.175085444
14826	0.01	0.0571281	UG0898H09	0.175045282
14827	8.3007646	47.437333	RRAGA	0.174983798
14828	0.2073627	1.1855756	SCO2	0.174904656
14829	2.0096384	11.491337	FAM208A	0.174882904
14830	4.50269	25.765029	NAA15	0.174759747
14831	612.88056	3507.2729	RPS6	0.174745614
14832	0.01	0.0573068	ANKRD20A4	0.174499507
14833	4.9756682	28.536758	CEP170	0.17435997

14834	0.01	0.0574866	CLVS1	0.17395373
14835	0.7068173	4.0654957	MAP3K9	0.173857595
14836	0.01	0.0575851	SLC6A12	0.173656036
14837	0.01	0.0576345	SCNN1A	0.173507188
14838	0.01	0.057684	FBXW10	0.173358339
14839	20.380859	118.13018	TARS	0.172528807
14840	0.0824039	0.4781681	NOX1	0.172332529
14841	0.0756167	0.4387834	EOMES	0.172332529
14842	0.1505777	0.8737623	GNAT2	0.172332529
14843	0.1989494	1.1544507	RTBDN	0.172332528
14844	0.021324	0.1237376	PCDH19	0.172332528
14845	0.6340261	3.6790854	SH3TC1	0.172332528
14846	0.5075178	2.9449914	CWF19L2	0.172332528
14847	0.5051749	2.931396	KIAA1211	0.172332528
14848	0.3994028	2.3176286	ZNF721	0.172332528
14849	0.4797226	2.7837032	SYT14	0.172332528
14850	0.1899722	1.1023583	FABP3	0.172332528
14851	0.1509594	0.8759776	NAP1L3	0.172332528
14852	0.1132298	0.6570427	MYB	0.172332528
14853	0.8001687	4.6431671	CXCL16	0.172332528
14854	0.1413837	0.8204119	LTA	0.172332528
14855	0.120323	0.6982027	WNT7A	0.172332528
14856	0.1819289	1.0556849	ANKRD29	0.172332528
14857	0.1223002	0.7096755	LOC440311	0.172332528
14858	0.0819825	0.4757227	TTC3P1	0.172332528
14859	0.0893651	0.5185622	MDH1B	0.172332527
14860	0.0496781	0.2882687	WIPF3	0.172332527
14861	0.0755892	0.4386243	CHRM3	0.172332527
14862	0.0251296	0.1458202	TTC40	0.172332527
14863	0.0833265	0.4835214	TMEFF1	0.172332527
14864	0.0546263	0.3169822	GPR37	0.172332527
14865	0.0757541	0.4395809	PA2G4P4	0.172332527
14866	0.0430845	0.2500077	PLCH2	0.172332527
14867	0.01	0.058083	ASIC2	0.172167555
14868	0.01	0.0582004	PDE1B	0.171820243
14869	43.892992	256.12629	SLC20A1	0.171372458
14870	1.9362961	11.322913	RANBP6	0.171006893
14871	0.4527612	2.6481043	N4BP2	0.170975579
14872	1.6267691	9.5233952	MGA	0.170818183
14873	7.1861897	42.108374	FKBP11	0.170659397
14874	5.614301	32.902463	C9orf123	0.170634671
14875	0.01	0.0586919	SLC26A8	0.170381378
14876	0.01	0.0588461	COL18A1-AS1	0.169934834

14877	0.6346607	3.7385675	ANKRD34A	0.169760401
14878	0.01	0.0590011	FAM133A	0.169488291
14879	2.1175843	12.499639	TDRKH	0.169411638
14880	1.1271251	6.6551524	ZNF488	0.169361278
14881	0.01	0.059053	BHLHE22	0.169339441
14882	2.5323072	14.966424	FRAT2	0.169199209
14883	0.01	0.0593661	CYP4F11	0.168446353
14884	4.5235631	26.859481	ARG2	0.16841588
14885	0.8387503	4.9829279	WDR35	0.168324795
14886	17.796712	105.76529	SUPT16H	0.168266097
14887	5.2913936	31.491851	UBE4A	0.168024215
14888	3.2942337	19.646553	NAA35	0.167674892
14889	0.2142558	1.2778051	ADAMTS3	0.167674892
14890	0.01	0.059931	ANKRD35	0.16685864
14891	2.2120617	13.286394	SLC6A8	0.166490748
14892	26.31752	158.07197	SIGMAR1	0.166490747
14893	1.037961	6.2460871	RILP	0.166177795
14894	2.2564984	13.582011	SC5D	0.166138759
14895	0.2545337	1.5385332	RFX3	0.165439227
14896	0.2991023	1.8079283	VILL	0.165439227
14897	8.293719	50.169832	GNPNAT1	0.165312873
14898	0.01	0.0604976	GPR158	0.165295737
14899	0.8196377	4.9607057	RALGAPA1	0.165226032
14900	3.2527371	19.688339	SMOC1	0.165211349
14901	0.4802997	2.9092909	LRP4	0.165091666
14902	0.537944	3.2602818	SP4	0.164999229
14903	2.7333798	16.582037	DEPDC1B	0.164839809
14904	0.5769933	3.5003276	FMNL1	0.164839809
14905	7.8287897	47.611556	LAMB3	0.164430451
14906	22.437723	136.48942	DDX21	0.164391664
14907	0.01	0.0610196	TMC3	0.163881681
14908	0.8433633	5.1513827	ZNF280C	0.163715902
14909	0.01	0.0611121	LAX1	0.163633601
14910	1.6596092	10.14618	SCAMP5	0.163569857
14911	2.7907533	17.071177	CAAP1	0.163477497
14912	1.3421897	8.2157817	DCUN1D5	0.163367252
14913	0.0707158	0.4331421	TMTC1	0.163262396
14914	0.0812685	0.4977784	SLC4A4	0.163262395
14915	2.5101653	15.375037	UHRF2	0.163262395
14916	0.3727336	2.283034	DISP2	0.163262395
14917	0.3575628	2.190111	LOC100507032	0.163262395
14918	311.65112	1914.4295	HMGA1	0.162790599
14919	2.766365	17.000026	C5orf22	0.162727109

14920	1.0355255	6.3844349	LINC00622	0.162195321
14921	0.01	0.0617298	MYH7B	0.161996274
14922	0.6786118	4.1918565	MYO5B	0.161888132
14923	2.2781314	14.081527	C1orf233	0.161781557
14924	0.156951	0.9714612	NRXN2	0.161561745
14925	0.01	0.0619004	KLHDC8A	0.16154973
14926	7.3725501	45.741784	NRP2	0.161177582
14927	1.9001131	11.796477	CHORDC1	0.161074624
14928	0.9238429	5.7370129	ZYG11A	0.161032034
14929	11.896616	73.978253	LPCAT1	0.160812345
14930	2.5525028	15.926341	GALNT18	0.160269251
14931	13.240399	82.703934	NRIP3	0.160093942
14932	1.1861618	7.4124427	REEP2	0.160023062
14933	2.8225666	17.652498	FBXO17	0.15989616
14934	0.9319655	5.8440735	BIRC3	0.159471892
14935	0.1068441	0.6716539	USP32P1	0.15907618
14936	0.1532349	0.9632802	B3GNT3	0.15907618
14937	0.1477487	0.9287919	ZNF815P	0.15907618
14938	0.1742471	1.0953687	HNRNPH2	0.15907618
14939	0.2735799	1.7198044	RASD2	0.15907618
14940	1.7586456	11.055367	SNORD17	0.15907618
14941	0.1827264	1.1486725	FAM106A	0.15907618
14942	0.223365	1.4041383	CCDC181	0.15907618
14943	1.349261	8.4818547	SLC45A3	0.15907618
14944	2.5209617	15.847512	HIST3H2A	0.15907618
14945	0.2136335	1.3429636	GTF2H2	0.159076179
14946	7.4428393	46.886498	BIRC2	0.158741635
14947	0.01	0.0630428	RAX	0.158622384
14948	0.01	0.0630625	NAALAD2	0.158572769
14949	0.01	0.0631152	CADM2	0.158440459
14950	0.8672684	5.4765755	ALKBH8	0.15835962
14951	0.01	0.0632605	TBR1	0.158076607
14952	0.1056793	0.6695942	PURG	0.157825835
14953	1.9078037	12.101554	LRP3	0.157649488
14954	0.01	0.0634597	FLJ42289	0.157580447
14955	2.6818662	17.037881	PTGS2	0.157406089
14956	0.01	0.0638214	RPS16P5	0.156687358
14957	0.01	0.0639023	OCA2	0.156488895
14958	0.3324135	2.1355759	DOCK3	0.155655186
14959	0.01	0.0643717	CRHR2	0.155347728
14960	1.8779857	12.108281	TNFRSF21	0.155099275
14961	0.01	0.0647647	UGT3A1	0.154405024
14962	0.6241375	4.0442365	C9orf72	0.154327637

14963	10.211277	66.173435	SLCO4A1	0.154310826
14964	15.893837	103.32591	PSIP1	0.153822371
14965	0.9655575	6.2772946	SLC18A2	0.153817463
14966	0.01	0.0651626	CLCN1	0.153462319
14967	4.0076827	26.148813	NETO2	0.15326442
14968	0.4560164	2.9769102	ATP1A1OS	0.153184469
14969	0.8028876	5.2413117	CCNJ	0.153184469
14970	0.01	0.0654376	C15orf54	0.15281731
14971	0.01	0.0656187	GRM6	0.152395575
14972	0.01	0.0656864	PEX5L	0.152238457
14973	0.3604281	2.370331	EDARADD	0.152058113
14974	0.01	0.0657793	FNDC7	0.152023456
14975	0.01	0.0658008	AKNAD1	0.151973838
14976	0.01	0.0658438	LOC100128770	0.151874606
14977	0.01	0.0659084	SPARCL1	0.151725758
14978	1.2541235	8.2823135	TNIK	0.151421887
14979	0.01	0.0661898	LOC255167	0.151080751
14980	1.0074561	6.6811441	CCNO	0.150790962
14981	0.01	0.0666054	FMR1-AS1	0.150138047
14982	0.8914427	5.9391476	MCIDAS	0.150096073
14983	0.01	0.0668484	LINC00319	0.14959227
14984	0.01	0.0671826	ASZ1	0.148848029
14985	1.6174798	10.867776	TIGD1	0.148832638
14986	0.01	0.067205	PCDP1	0.148798415
14987	0.01	0.067205	LOC286177	0.148798415
14988	0.01	0.0675881	LRRN4	0.147954941
14989	0.0547411	0.3705897	SYT2	0.147713596
14990	0.0884924	0.5990807	LOC285768	0.147713596
14991	0.1159708	0.7851057	PAR-SN	0.147713596
14992	0.2202955	1.4913688	SYCE2	0.147713596
14993	0.079059	0.5352181	GLS2	0.147713596
14994	0.2702476	1.8295377	SDK2	0.147713596
14995	0.0492554	0.3334519	KBTBD3	0.147713595
14996	0.2864598	1.939292	SP9	0.147713595
14997	0.5552385	3.7588852	RGCC	0.147713595
14998	0.148475	1.0051546	SLC2A13	0.147713595
14999	0.1211625	0.8202529	TMEM74B	0.147713595
15000	0.260956	1.7666353	KIAA0226L	0.147713595
15001	0.2348163	1.5896732	NUP62CL	0.147713595
15002	0.1705397	1.1545294	C2orf27A	0.147713595
15003	0.1806671	1.2230905	ANKRD19P	0.147713595
15004	0.0958159	0.6486597	TXNDC2	0.147713595
15005	0.1104103	0.7474622	SLC19A3	0.147713595

15006	0.0948996	0.6424567	FAM131B	0.147713595
15007	0.0812791	0.5502476	CNKSR1	0.147713595
15008	0.0995698	0.6740731	LOC494141	0.147713595
15009	0.0808063	0.5470473	APCDD1	0.147713595
15010	0.0664327	0.4497402	SYT16	0.147713595
15011	0.0321208	0.217453	JAKMIP3	0.147713594
15012	0.0107795	0.0729755	GPR98	0.14771359
15013	0.01	0.0678156	CTAGE10P	0.147458782
15014	5.1212074	34.742149	FMNL2	0.147406179
15015	0.01	0.0679298	SIGLEC8	0.147210702
15016	0.01	0.0680215	PRSS30P	0.147012238
15017	1.146138	7.7974134	PLEKHG5	0.146989509
15018	0.01	0.0681365	ZCCHC16	0.146764158
15019	0.01	0.0683677	SSTR5-AS1	0.146267998
15020	0.01	0.0684605	VAV1	0.146069533
15021	0.4373037	3.0074755	PLCG2	0.14540557
15022	0.01	0.0688582	FAM163A	0.145226062
15023	0.01	0.0688817	CCDC135	0.145176444
15024	0.01	0.0692366	C7orf65	0.144432205
15025	0.01	0.0692366	GPR116	0.144432205
15026	0.01	0.0694274	LINC00312	0.144035277
15027	1.004413	6.9877441	TUB	0.143739239
15028	0.01	0.0697156	SLC35F3	0.143439886
15029	1.0201909	7.1285434	ABCA2	0.143113518
15030	0.01	0.0698848	C1orf145	0.143092573
15031	0.01	0.0700062	GRHL3	0.142844493
15032	0.01	0.0700549	TTLL2	0.142745261
15033	0.1968819	1.3804647	SLCO2A1	0.142620024
15034	1.0240762	7.1804516	HMX2	0.142620023
15035	4.5085004	31.611974	DDX20	0.142620023
15036	4.8434557	34.055509	SLC35F2	0.142222384
15037	0.01	0.070336	CHRNB2	0.142174676
15038	0.4140844	2.9145345	INSR	0.142075672
15039	0.01	0.0703974	LINC00167	0.142050636
15040	0.2396183	1.6883936	GPR63	0.141920906
15041	0.01	0.0704959	BTN1A1	0.141852173
15042	0.01	0.0704959	TRIM55	0.141852173
15043	0.8580265	6.0527811	ATR	0.141757402
15044	0.01	0.0705453	ASIC4	0.14175294
15045	22.884725	161.48133	SCD	0.141717468
15046	0.807437	5.7005009	MGC21881	0.141643174
15047	1.3012457	9.1964676	TBX1	0.141494076
15048	2.2269913	15.756553	MAPK12	0.141337469

15049	0.1570062	1.113524	CASZ1	0.140999341
15050	0.1419293	1.0065954	PDE8B	0.140999341
15051	2.7143206	19.25059	CNIH2	0.140999341
15052	0.2954624	2.095488	FAM86HP	0.140999341
15053	0.1055187	0.7483633	ITGB4	0.140999341
15054	0.0878335	0.6229352	DRP2	0.14099934
15055	0.01	0.0710176	CCDC144NL	0.140810237
15056	0.01	0.071168	SULT1C2	0.140512541
15057	6.0913263	43.37292	DEAF1	0.14044077
15058	1.3425141	9.5880046	WDHD1	0.140020179
15059	0.01	0.0717763	TF	0.139321756
15060	0.3052514	2.1930295	PCDH1	0.139191657
15061	0.01	0.0718787	DENND1C	0.139123292
15062	0.1855289	1.3375626	SVEP1	0.138706669
15063	0.01	0.072136	AICDA	0.138627132
15064	0.01	0.072136	MBP	0.138627132
15065	2.1085126	15.270213	TPD52	0.1380801
15066	2.1802912	15.795041	DICER1	0.138036438
15067	0.01	0.0725253	C1orf127	0.137882891
15068	0.1055989	0.7659533	CHST1	0.137866023
15069	0.3714786	2.6944899	FHIT	0.137866022
15070	0.6315136	4.5806329	SNHG10	0.137866022
15071	0.3208614	2.3273423	CCDC15	0.137866022
15072	0.3051237	2.2131901	DPYSL4	0.137866022
15073	0.1803544	1.3081859	LOC440028	0.137866022
15074	0.3541198	2.5685792	CDKL1	0.137866022
15075	0.0773713	0.5612062	PDE7B	0.137866022
15076	0.01	0.0727872	P2RY13	0.137386731
15077	0.5584814	4.0716737	CHST15	0.137162624
15078	3.7804898	27.573817	TAF1D	0.137104332
15079	0.01	0.0730246	KCNJ1	0.136940188
15080	0.2837684	2.0857348	PTPN22	0.136051996
15081	0.1778153	1.3069656	ZSWIM5	0.136051996
15082	0.2385525	1.7533924	IL17RD	0.136051996
15083	0.01	0.0735576	ABCC6P1	0.135947868
15084	0.01	0.0736382	FOLH1	0.13579902
15085	0.5190523	3.8276521	CDK18	0.135605924
15086	0.01	0.073773	C20orf197	0.135550939
15087	0.01	0.0738271	LRRIQ1	0.135451708
15088	0.01	0.0738271	ADPGK-AS1	0.135451708
15089	0.5455484	4.0450266	TPPP3	0.134868935
15090	0.01	0.0743719	TDGF1P3	0.134459388
15091	0.01	0.0745645	NAALADL1	0.134112076

15092	0.01	0.0747027	RASSF10	0.133863995
15093	4.5882194	34.296311	TTF2	0.133781721
15094	1.6789053	12.573322	SPOCK1	0.133529177
15095	0.01	0.0749388	GOLGA6C	0.13344226
15096	0.455518	3.4141985	HOXB5	0.133418731
15097	0.01	0.0750923	LOC145820	0.133169372
15098	0.7191701	5.4096431	MGC57346	0.132942236
15099	0.01	0.0752606	LOC283856	0.132871675
15100	0.3378721	2.5487568	ATP6V0E2-AS1	0.132563483
15101	0.01	0.0756561	CD226	0.13217705
15102	0.972052	7.3572526	SALL2	0.132121605
15103	0.01	0.0757272	KCNJ10	0.132053011
15104	0.01	0.0757414	LOC653786	0.132028203
15105	0.01	0.0757699	LOC643387	0.131978587
15106	0.01	0.0758269	CLDN10	0.131879355
15107	1.5692733	11.913785	LEPREL2	0.13171913
15108	0.2488564	1.8910153	ZNF268	0.131599385
15109	0.01	0.0760271	CASC1	0.131532042
15110	5.805	44.164616	RAC3	0.131440064
15111	0.01	0.0761996	TMPRSS11B	0.131234347
15112	0.7120285	5.4364641	RAB6B	0.130972721
15113	109.67384	839.07062	S100A16	0.13070871
15114	0.6907607	5.2863104	FBXO41	0.130669719
15115	0.01	0.0766342	FAM24B-CUZD1	0.130490107
15116	0.01	0.0766342	RBPJL	0.130490107
15117	0.01	0.0767217	ZIM3	0.130341258
15118	0.01	0.0768387	DACH1	0.130142794
15119	0.01	0.0771918	MAOB	0.129547402
15120	0.0563699	0.4361328	GPM6A	0.129249397
15121	0.051141	0.3956768	C3orf72	0.129249397
15122	0.0655757	0.5073577	HSPA12B	0.129249397
15123	0.057937	0.4482577	KCNB2	0.129249396
15124	0.3544209	2.7421476	LOC257396	0.129249396
15125	0.2484644	1.922364	PRKG1	0.129249396
15126	0.0515203	0.3986113	ZMAT1	0.129249396
15127	0.1090241	0.843517	LRRN1	0.129249396
15128	1.6409409	12.695927	SNORA40	0.129249396
15129	0.03225	0.2495176	MUC20	0.129249396
15130	0.1219424	0.9434656	KCNJ11	0.129249396
15131	0.1795	1.3887879	LOC644656	0.129249396
15132	0.1524503	1.1795046	C15orf59	0.129249396
15133	0.104776	0.81065	CXCL14	0.129249396
15134	0.0889836	0.688464	KBTBD8	0.129249396

15135	0.136031	1.0524692	CN5H6.4	0.129249396
15136	0.0854096	0.6608126	RGS7	0.129249396
15137	0.0691668	0.535142	POU2F3	0.129249395
15138	0.0609355	0.471457	DLL4	0.129249395
15139	0.0549142	0.4248703	CD1D	0.129249395
15140	0.0614929	0.4757695	GRM2	0.129249395
15141	0.0441244	0.3413895	KGFLP2	0.129249395
15142	0.0348378	0.2695391	KCNA6	0.129249395
15143	0.01	0.0774439	MRC1	0.129125667
15144	0.01	0.077608	SCNN1B	0.128852779
15145	0.9479508	7.3825283	SIM2	0.128404629
15146	4.8027171	37.530112	DROSHA	0.127969699
15147	0.01	0.0781799	DNAJB7	0.127910074
15148	0.01	0.0783166	EFCAB5	0.127686802
15149	0.01	0.0784538	SND1-IT1	0.127463529
15150	0.01	0.0789352	PKDREJ	0.126686212
15151	0.01	0.0789455	CHIT1	0.126669674
15152	0.1274355	1.0065068	IKZF3	0.126611653
15153	1.5955891	12.608502	ERMP1	0.126548662
15154	0.01	0.0791937	CSDC2	0.126272746
15155	0.01	0.0792248	TDRD10	0.126223129
15156	0.01	0.0792559	ANKK1	0.126173513
15157	0.4146795	3.2974885	TNFRSF19	0.125756169
15158	0.01	0.0797262	CATSPERD	0.125429274
15159	0.0999518	0.7974915	NTRK2	0.125332748
15160	1.0738783	8.5682177	BMP6	0.125332748
15161	0.01	0.0798842	FAM129C	0.125181194
15162	0.01	0.0798842	KPRP	0.125181194
15163	0.01	0.0800111	GABRA6	0.124982729
15164	0.01	0.0800746	EPHA1-AS1	0.124883498
15165	1.0563335	8.4647177	LOC441454	0.12479252
15166	0.01	0.0801383	CDRT4	0.124784265
15167	0.01	0.080266	CLEC19A	0.1245858
15168	0.01	0.080282	SIDT1	0.124560993
15169	4.9637688	39.852372	CLMP	0.124553911
15170	0.2491823	2.0082488	SHC2	0.12407942
15171	0.6360107	5.1258354	CALHM2	0.12407942
15172	0.1506502	1.2141437	ITGA7	0.12407942
15173	0.1486268	1.1978358	TMEM170B	0.12407942
15174	0.1332762	1.0741198	KIF27	0.12407942
15175	0.3883703	3.1550539	KIAA1958	0.123094663
15176	0.9477012	7.6989628	LAT2	0.123094663
15177	0.01	0.0812693	MMP28	0.123047705

15178	0.01	0.0813349	PNPLA1	0.122948473
15179	3.7471556	30.47842	RCL1	0.122944547
15180	1.1188582	9.1035267	LTBP2	0.122903818
15181	7.3910645	60.172456	IGF2BP1	0.122831359
15182	9.7446063	79.592738	KRT8	0.122430846
15183	0.01	0.0818302	SLC28A2	0.122204233
15184	0.01	0.0819633	FLJ38576	0.122005768
15185	0.01	0.0821303	SLC2A5	0.121757689
15186	0.2121115	1.7436709	CCNA1	0.121646491
15187	0.1985703	1.6323551	C9orf43	0.121646491
15188	0.330968	2.7207359	ZDHHC23	0.121646491
15189	1.1680495	9.6019993	STK32C	0.12164649
15190	0.3990416	3.2803383	DOC2A	0.12164649
15191	0.7913905	6.5056584	ULBP1	0.12164649
15192	0.5860091	4.8173123	PKP3	0.12164649
15193	0.1111464	0.9136836	EFR3B	0.12164649
15194	0.1043041	0.8574358	KIAA1407	0.12164649
15195	0.0866346	0.7121832	ANO1	0.12164649
15196	0.01	0.0822644	ZAP70	0.121559225
15197	0.01	0.0822644	KRT6A	0.121559225
15198	0.01	0.0822644	FA2H	0.121559225
15199	0.01	0.082399	SRD5A2	0.121360761
15200	0.01	0.0824327	PLIN5	0.121311145
15201	1.0415241	8.6076773	MPP6	0.120999435
15202	0.01	0.0828052	DGCR9	0.120765368
15203	0.01	0.0829074	AGAP7	0.12061652
15204	0.01	0.0829415	LRRC25	0.120566904
15205	0.01	0.0831468	CLEC4M	0.120269208
15206	0.01	0.0832498	DCST2	0.12012036
15207	0.01	0.0833187	LHX3	0.120021128
15208	0.01	0.0834221	LRRC36	0.11987228
15209	0.01	0.0835258	ZNF664-FAM101A	0.119723432
15210	0.4101199	3.4280699	GPC6	0.119635805
15211	0.6198413	5.1810685	EPB41L4A	0.119635805
15212	0.01	0.0838037	CSNK1A1L	0.119326504
15213	0.2655898	2.226102	TNFRSF11B	0.119307135
15214	0.01	0.0841536	HSD17B13	0.118830343
15215	0.7852608	6.6240343	TAF4B	0.118547217
15216	0.01	0.0843885	DISC1	0.118499571
15217	0.01	0.0844179	RBFOX1	0.118458225
15218	0.01	0.0845419	RGS11	0.118284568
15219	0.01	0.0847196	FLJ41941	0.118036488
15220	0.01	0.0847552	ALDH1A1	0.117986872

15221	0.01	0.0849696	LOC100996455	0.117689176
15222	0.01	0.0850054	TAF7L	0.11763956
15223	0.01	0.0851491	ZNF730	0.117441096
15224	0.01	0.0853564	MAP2	0.117155804
15225	0.01	0.0854016	CYP4F2	0.117093784
15226	0.01	0.0858381	RNF144A-AS1	0.116498392
15227	0.01	0.0859113	PIFO	0.11639916
15228	0.7837842	6.7379138	FAM72D	0.116324456
15229	0.01	0.0859846	VSX1	0.116299928
15230	0.6320883	5.4406304	NIPAL4	0.116179232
15231	0.01	0.0865012	WBP2NL	0.115605303
15232	0.01	0.0865012	MEOX1	0.115605303
15233	0.01	0.0865012	TRIM34	0.115605303
15234	0.01	0.0865384	FBLN7	0.115555687
15235	0.01	0.0866127	NME8	0.115456455
15236	1.265387	10.966989	HMHA1	0.115381435
15237	0.01	0.0866872	RERG	0.115357223
15238	0.01	0.0867619	SLC7A4	0.115257991
15239	0.01	0.0867992	CTSE	0.115208375
15240	0.01	0.0868741	KRT5	0.115109143
15241	0.01	0.0869303	TMEM151B	0.115034719
15242	0.01	0.0870241	GRID2IP	0.114910679
15243	0.0415304	0.3614848	MIR17HG	0.114888353
15244	0.0605285	0.526846	CHRNA7	0.114888353
15245	0.0541438	0.4712732	EIF5AL1	0.114888353
15246	0.058605	0.5101042	ACAD11	0.114888352
15247	0.0707398	0.6157266	FAM83A	0.114888352
15248	0.1894885	1.6493277	C11orf92	0.114888352
15249	0.1016583	0.8848442	HCLS1	0.114888352
15250	0.1700526	1.4801555	RHOF	0.114888352
15251	0.0827968	0.7206717	KCNK13	0.114888352
15252	0.3407076	2.9655542	SRGAP2B	0.114888352
15253	0.1305761	1.136548	ZNF391	0.114888352
15254	0.6291306	5.476017	RAD51B	0.114888352
15255	0.4575638	3.982682	MTSS1	0.114888352
15256	0.1461427	1.2720411	ACER2	0.114888352
15257	0.3837928	3.3405721	CNFN	0.114888352
15258	0.142026	1.236209	ADAM11	0.114888352
15259	0.2111444	1.8378223	TMEM52	0.114888352
15260	0.0857788	0.7466271	USP32P2	0.114888352
15261	0.0812791	0.7074612	FGFBP3	0.114888352
15262	0.0298524	0.2598382	KLHL3	0.114888352
15263	0.0981627	0.8544186	FUT1	0.114888352

15264	0.0379115	0.3299856	SYCP2	0.114888351
15265	0.0450594	0.3922012	LGR5	0.114888351
15266	0.01	0.087137	PCDH15	0.114761832
15267	2.089218	18.223625	MTMR12	0.114643388
15268	0.01	0.0873636	LOC100133161	0.114464134
15269	0.01	0.0874394	LOC644172	0.114364903
15270	0.01	0.0875344	RPH3A	0.114240863
15271	0.01	0.0876486	IGSF1	0.114092015
15272	1.4676021	12.910635	FKBP5	0.113673887
15273	0.01	0.0880698	ATG9B	0.113546239
15274	0.01	0.0881276	MINOS1P1	0.113471815
15275	0.01	0.0881469	RNF43	0.113447006
15276	5.1691782	45.632056	RAI14	0.113279537
15277	1.571979	13.899852	FAM195A	0.113093222
15278	0.01	0.0887094	CD200R1	0.112727574
15279	0.01	0.0887876	MATN4	0.112628343
15280	0.01	0.0887876	DYDC2	0.112628343
15281	0.01	0.0888659	PRKG1-AS1	0.11252911
15282	0.01	0.0888659	DES	0.11252911
15283	0.01	0.0889443	FLJ42393	0.112429879
15284	0.01	0.0890229	LOC100506025	0.112330646
15285	0.01	0.0890819	LOC440905	0.112256223
15286	0.01	0.0891805	PALM3	0.112132183
15287	0.01	0.0892199	LOC440040	0.112082567
15288	0.5772839	5.153578	ENDOD1	0.112016143
15289	0.01	0.0893584	FAM20A	0.111908911
15290	0.2146789	1.920492	RPS6KA5	0.111783262
15291	3.4182531	30.579293	TRIB1	0.111783261
15292	1.335181	11.944373	PDIA5	0.111783261
15293	0.9581586	8.5715751	PTGR2	0.111783261
15294	0.01	0.0894973	SHANK2-AS1	0.111735254
15295	0.01	0.0896167	COL6A4P1	0.111586406
15296	0.7040524	6.3226883	IL17RB	0.111353326
15297	0.01	0.0902184	CNPY1	0.110842166
15298	0.01	0.0902318	CD93	0.110825628
15299	0.2160327	1.9500137	ZNF595	0.110785197
15300	0.2573186	2.3226806	ADAMTS6	0.110785197
15301	1.0144621	9.1803783	AP1G2	0.1105033
15302	0.01	0.0906648	TFAMP1	0.11029639
15303	0.01	0.0906648	ACVR1C	0.11029639
15304	0.4675054	4.2431126	FAM117B	0.110179813
15305	0.01	0.0908692	SLX1A-SULT1A3	0.11004831
15306	0.4614692	4.1951943	OVGP1	0.109999486

15307	0.01	0.0909923	AMY2B	0.109899462
15308	0.01	0.0909923	RBMXL2	0.109899462
15309	1.9286597	17.560238	KCNMA1	0.109831066
15310	0.01	0.0913635	CCKBR	0.109452918
15311	0.2141824	1.9678361	DMKN	0.108841597
15312	0.1974415	1.8140261	IGFBP5	0.108841597
15313	0.288842	2.6537832	KHDC1	0.108841597
15314	0.1375347	1.2636229	SORCS2	0.108841596
15315	0.01	0.0919051	FOXH1	0.10880791
15316	0.01	0.0922838	SMIM5	0.108361366
15317	0.5373639	4.9607417	CREB5	0.108323303
15318	0.01	0.0924002	COL19A1	0.108224922
15319	0.01	0.0926231	LOC100505782	0.107964437
15320	0.01	0.0926657	IL9R	0.107914822
15321	0.2857724	2.6486119	CACNG8	0.107895148
15322	0.01	0.0927083	KCNV2	0.107865206
15323	0.01	0.0928792	TLX2	0.107666742
15324	0.01	0.0930937	RSPO3	0.107418661
15325	0.01	0.0932444	PRH2	0.107245006
15326	0.01	0.0934245	CNTN5	0.107038273
15327	0.103151	0.9643438	MYH3	0.106965018
15328	0.154599	1.4453233	EPHB6	0.106965018
15329	0.2600659	2.4313176	PLEKHN1	0.106965017
15330	0.1933803	1.8078836	THBS4	0.106965017
15331	0.01	0.0935257	PHYHD1	0.106922502
15332	11.732555	109.78046	DUSP6	0.106872886
15333	2.1664882	20.3079	LOXL1-AS1	0.106682041
15334	0.01	0.0937432	LOC100129931	0.106674421
15335	0.01	0.0938305	DCTN1-AS1	0.106575189
15336	0.01	0.0938742	KRT4	0.106525573
15337	0.01	0.0941372	LOC399753	0.106227877
15338	0.7747193	7.3051729	HRASLS	0.106050787
15339	0.833598	7.860366	CRIP2	0.106050787
15340	5.1456667	48.520778	FRMD4A	0.106050787
15341	0.01	0.0943355	LOC100133331	0.106004605
15342	0.8718815	8.2405224	KIAA1671	0.105804157
15343	9.9685379	94.330495	IPO7	0.105676726
15344	0.01	0.094653	PCDHA9	0.105649024
15345	0.2237487	2.1206452	FGFR2	0.105509711
15346	0.01	0.094846	EIF1B-AS1	0.105434021
15347	1.1670956	11.146153	LOC202181	0.104708371
15348	0.01	0.0957472	FEZF1	0.104441701
15349	2.0969763	20.087184	SIMC1	0.104393743

15350	0.01	0.0958838	LOC146513	0.104292853
15351	0.01	0.0958838	TREM1	0.104292853
15352	0.01	0.0959295	ELAVL4	0.104243237
15353	81.8161	786.24019	KRT18	0.104059932
15354	0.01	0.0962042	FEZF2	0.103945541
15355	0.01	0.0964344	WDR65	0.103697461
15356	0.01	0.0964344	LOC400685	0.103697461
15357	0.01	0.0964575	CCDC177	0.103672652
15358	0.01	0.0966193	CCDC37	0.103498997
15359	0.050338	0.4868305	INPP4B	0.103399518
15360	0.1273836	1.2319551	NECAB2	0.103399517
15361	0.0553518	0.5353196	NFATC2	0.103399517
15362	0.0975197	0.9431345	CERS1	0.103399517
15363	0.1493903	1.4447874	PSD	0.103399517
15364	0.222649	2.153289	DDN	0.103399517
15365	1.1431679	11.055834	VLDLR	0.103399517
15366	0.1530099	1.4797933	FBXO16	0.103399517
15367	0.5112007	4.9439374	APOE	0.103399517
15368	0.1148207	1.1104565	KLRG1	0.103399517
15369	0.1744659	1.6872988	DLL3	0.103399517
15370	0.1216576	1.1765782	SNAI3	0.103399517
15371	0.0860089	0.8318112	FAM189A2	0.103399516
15372	0.01	0.0972252	SOAT2	0.102853988
15373	0.01	0.0974446	CCSER1	0.102622447
15374	0.4060785	3.9665496	CGN	0.102375759
15375	0.01	0.0982202	FADS6	0.101812053
15376	0.2419031	2.3784907	GAREML	0.101704443
15377	0.01	0.0985805	KLHL23	0.101439933
15378	1.0650741	10.529473	SIGIRR	0.101151701
15379	0.01	0.0990407	LINC00330	0.10096858
15380	0.7690018	7.6231197	ITPRIPL1	0.100877577
15381	0.5670735	5.6214025	PLA2G4A	0.100877577
15382	0.01	0.0993336	SLC5A4	0.100670884
15383	0.01	0.0996282	FAM133DP	0.100373189
15384	0.01	0.0997762	NRK	0.10022434
15385	0.4676129	4.6731363	CPNE7	0.100064049
15386	0.1602251	1.6012258	ELOVL7	0.100064048
15387	0.4416803	4.4139762	COL9A2	0.100064048
15388	3.9777602	39.792593	MLL	0.099962326
15389	0.01	0.1000734	SRPK3	0.099926644
15390	0.01	0.1002476	EXTL1	0.099752988
15391	0.01	0.1002726	RAD21-AS1	0.09972818
15392	0.3901699	3.9149242	ARHGAP44	0.099662185

15393	0.01	0.1004725	DNAJC5G	0.099529716
15394	0.01	0.1005226	KRT32	0.0994801
15395	0.3672885	3.694215	PID1	0.099422612
15396	0.01	0.1010772	GDF7	0.098934324
15397	0.01	0.1012295	LOC100506071	0.098785476
15398	0.0704767	0.7156755	MEGF11	0.098475731
15399	0.1628121	1.6533222	NXPH2	0.098475731
15400	0.1005304	1.0208646	C1orf226	0.098475731
15401	0.7011759	7.1202916	PDZRN3	0.09847573
15402	0.2246289	2.2810589	CHDH	0.09847573
15403	0.1232768	1.25185	MAPK8IP2	0.09847573
15404	0.8063076	8.2178735	PROSER2	0.09811633
15405	0.01	0.1020495	CYP8B1	0.09799162
15406	0.01	0.102153	CLEC4D	0.097892388
15407	0.01	0.1022048	ZP1	0.097842771
15408	0.01	0.1022566	DHH	0.097793156
15409	0.5959948	6.1002327	SLC37A2	0.097700331
15410	0.1198111	1.2282438	HSD3BP4	0.097546714
15411	0.01	0.1030408	ATP6V1B1	0.097048915
15412	0.01	0.1031199	NCKAP1L	0.096974492
15413	0.1953135	2.0148488	MAN1C1	0.096937047
15414	0.48465	4.9996365	CTXN1	0.096937047
15415	0.01	0.1033932	MYOM1	0.096718143
15416	0.01	0.103748	CCDC144A	0.096387369
15417	0.4389668	4.563748	OLFM2	0.096185597
15418	0.01	0.1040515	FAM154A	0.096106212
15419	0.01	0.1041053	NPY5R	0.096056595
15420	0.01	0.104213	FAM225A	0.095957363
15421	0.01	0.1042669	PLK5	0.095907747
15422	0.01	0.1043749	TESPA1	0.095808515
15423	0.2917126	3.0469159	ARMC4	0.095740293
15424	0.01	0.1045644	EGFEM1P	0.09563486
15425	0.01	0.1046458	LOC100129617	0.095560435
15426	0.01	0.1047002	C9orf153	0.09551082
15427	0.01	0.1047546	BEST2	0.095461204
15428	0.01	0.1047546	FLT3	0.095461204
15429	0.1590369	1.668227	ANK3	0.095332888
15430	0.01	0.1049182	FTCD	0.095312355
15431	0.01	0.1049455	CAPN14	0.095287547
15432	0.01	0.1052469	MATN1	0.095014659
15433	0.01	0.1053019	CXCR3	0.094965043
15434	0.01	0.1054948	ASXL3	0.094791387
15435	0.0407747	0.4337754	SLC8A2	0.093999562

15436	0.0342706	0.3645825	SCN3B	0.093999561
15437	0.1219424	1.2972653	DHRS2	0.093999561
15438	0.2648024	2.8170601	DLK2	0.093999561
15439	0.1182073	1.2575305	LOC348761	0.093999561
15440	0.1148207	1.2215021	TUBAL3	0.093999561
15441	0.0676402	0.7195801	CCDC64	0.09399956
15442	0.0549142	0.5841967	RHPN1	0.09399956
15443	0.0183515	0.1952295	WNK3	0.09399956
15444	0.01	0.1064139	PSMB11	0.093972723
15445	0.01	0.1069787	SSUH2	0.093476563
15446	0.01	0.1070355	SFRP5	0.093426947
15447	0.01	0.1072634	SLC9B1	0.093228482
15448	0.01	0.1075495	TREH	0.092980403
15449	0.01	0.107607	LOC401980	0.092930787
15450	0.1387876	1.4956457	LAMA3	0.092794438
15451	0.01	0.1078373	NOXRED1	0.092732323
15452	0.01	0.1078373	OTOP1	0.092732323
15453	0.01	0.1081555	LOC286184	0.092459435
15454	0.01	0.1083008	TMIE	0.092335395
15455	0.01	0.1083591	SLC22A31	0.092285778
15456	83.009202	901.72966	PHGDH	0.092055531
15457	0.01	0.1087391	ANKRD20A5P	0.091963274
15458	0.01	0.1090627	RPL13AP17	0.091690386
15459	0.01	0.10924	PMFBP1	0.091541538
15460	0.4089773	4.4744463	AFF3	0.091402888
15461	0.01	0.1094179	PMCHL2	0.09139269
15462	0.01	0.1094774	SLC15A2	0.091343074
15463	0.1898568	2.0809678	STAC2	0.091234868
15464	0.01	0.1099252	CXorf22	0.090970954
15465	0.01	0.1101957	MKX	0.090747682
15466	0.2604343	2.871339	SLC16A9	0.090701331
15467	0.01	0.1103163	SMAD5-AS1	0.09064845
15468	0.01	0.1104372	PCDHAC1	0.090549218
15469	1.2540451	13.892248	MMP3	0.090269419
15470	0.01	0.1111375	TMED7-TICAM2	0.089978634
15471	0.01	0.1111681	LOC285847	0.089953826
15472	0.1682677	1.8714576	CRHR1-IT1	0.089912624
15473	0.1671207	1.8587011	LINC00565	0.089912623
15474	0.7573926	8.4518247	C11orf82	0.089612915
15475	0.01	0.111599	MYBPH	0.089606514
15476	0.01	0.1117228	TINAG	0.089507282
15477	0.01	0.1118468	CAPN6	0.08940805
15478	0.01	0.1119089	GFI1B	0.089358434

15479	1.3424006	15.040884	HAUS6	0.089250109
15480	0.01	0.1125966	FAM74A2	0.088812658
15481	0.01	0.1127225	LOC100130855	0.088713426
15482	0.01	0.1127225	DUSP13	0.088713426
15483	0.7219382	8.1456982	FAM131C	0.088628157
15484	0.01	0.112912	C10orf111	0.088564578
15485	4.9990079	56.560242	WDR3	0.088383778
15486	0.9090963	10.299288	B4GALT6	0.08826788
15487	0.01	0.1134841	CYP2A13	0.088118034
15488	0.01	0.1134841	BEST3	0.088118034
15489	0.1672213	1.9002505	ZNF521	0.087999589
15490	0.4359822	4.9543665	SYT17	0.087999589
15491	0.01	0.1138045	AGBL3	0.087869954
15492	0.01	0.1139332	SERPINI2	0.087770722
15493	0.01	0.114321	LOC283585	0.087473026
15494	0.01	0.1144508	FLJ40292	0.087373794
15495	0.01	0.1147114	CA14	0.08717533
15496	0.01	0.1149731	LINC00222	0.086976865
15497	1.6938745	19.50463	AK5	0.086844739
15498	0.01	0.1152525	TCHH	0.086765997
15499	0.01	0.1153548	P2RY2	0.086689093
15500	1.2447069	14.359424	SLC22A17	0.08668223
15501	0.01	0.115401	GP5	0.086654362
15502	2.1523642	24.856752	UCP2	0.086590728
15503	0.01	0.1155333	CDHR5	0.086555129
15504	0.01	0.1156327	CBWD3	0.086480706
15505	0.01	0.1160322	LOC100507250	0.086183009
15506	0.01	0.1160322	SIGLEC9	0.086183009
15507	0.01	0.1160322	LOC407835	0.086183009
15508	0.0332429	0.385799	FAM83B	0.086166265
15509	0.0629036	0.7300254	CCDC147	0.086166264
15510	0.0644003	0.7473962	LINGO2	0.086166264
15511	0.1228408	1.4256258	PDGFB	0.086166264
15512	0.2158835	2.505429	PHACTR1	0.086166264
15513	0.9821742	11.398593	FAM69B	0.086166264
15514	0.2651393	3.0770664	CBWD5	0.086166264
15515	0.0461265	0.5353196	FLJ22184	0.086166264
15516	0.1213212	1.4079896	ST6GAL2	0.086166264
15517	0.2579202	2.9932849	PRKAG2-AS1	0.086166264
15518	0.0221137	0.2566399	MYO15B	0.086166264
15519	0.1145682	1.3296175	LOC100129794	0.086166264
15520	0.0446538	0.5182289	ADAM22	0.086166264
15521	0.0517891	0.6010373	FLRT3	0.086166264

15522	0.0719612	0.835143	DMBX1	0.086166264
15523	0.0341751	0.3966176	ADCY5	0.086166263
15524	0.01	0.1166365	LINC00086	0.085736465
15525	0.01	0.1173154	KRT37	0.085240305
15526	0.01	0.1175892	RDH8	0.085041841
15527	0.7764172	9.1431962	BRSK2	0.084917478
15528	13.108284	154.38393	TMEM158	0.084907049
15529	0.01	0.1178642	TMEM40	0.084843377
15530	0.445679	5.2585195	FBXO27	0.084753702
15531	0.8647282	10.202837	LOC100287036	0.084753702
15532	0.01	0.1182099	C12orf50	0.084595297
15533	1.4658191	17.333708	SACS	0.084564661
15534	0.01	0.1184182	KCNH8	0.084446449
15535	0.01	0.118453	ATP2C2	0.084421641
15536	0.01	0.1185576	XKR3	0.084347217
15537	0.01	0.1185924	ENKUR	0.084322409
15538	0.3627844	4.3272362	ALDH5A1	0.083837446
15539	0.01	0.1193297	KPNA7	0.083801441
15540	0.01	0.1194004	CASP12	0.083751825
15541	0.01	0.119542	TCTE1	0.083652593
15542	0.7954179	9.5109324	COCH	0.083631962
15543	0.01	0.1202553	GPR18	0.083156433
15544	0.01	0.1203271	LOC100507244	0.083106817
15545	0.01	0.120399	LOC729121	0.083057201
15546	0.01	0.1204709	KCNA3	0.083007585
15547	0.01	0.1204709	CYP2D6	0.083007585
15548	0.6566485	7.9215323	VASH1	0.082894127
15549	0.1687104	2.0395451	LRCH2	0.082719614
15550	0.4424618	5.3489344	SATB1	0.082719614
15551	0.01	0.1209771	CKM	0.082660273
15552	1.422081	17.254096	STK32B	0.082419905
15553	0.01	0.1214876	AK7	0.082312961
15554	0.1056793	1.2848602	FZD3	0.082249616
15555	0.01	0.1216158	SEMA3E	0.082226132
15556	0.01	0.1217076	DKFZp686K1684	0.082164113
15557	0.2949328	3.5939752	AIF1L	0.082063109
15558	0.01	0.1219285	ALDH1L1	0.082015265
15559	0.01	0.1220762	LOC401127	0.081916032
15560	0.01	0.1222985	LOC441204	0.081767184
15561	0.01	0.1223727	FBP1	0.081717569
15562	0.01	0.1225712	EFCAB6	0.081585259
15563	0.01	0.122596	MLXIPL	0.08156872
15564	0.01	0.1226706	STAC3	0.081519105

15565	0.7557552	9.2733937	MPP2	0.081497156
15566	0.01	0.122945	LOC619207	0.081337179
15567	0.01	0.1230075	POTEJ	0.081295832
15568	0.01	0.1231453	SSC5D	0.08120487
15569	0.01	0.1232709	DKFZp434J0226	0.081122176
15570	0.01	0.1236869	ADCYAP1	0.080849288
15571	0.3930583	4.8657345	BHMT2	0.080780872
15572	0.01	0.1238009	RRN3P1	0.080774864
15573	0.01	0.1241058	EBF2	0.0805764
15574	0.1999995	2.4822751	DAAM2	0.080571052
15575	0.01	0.1241823	TMEM179	0.080526784
15576	0.01	0.1244123	SDS	0.080377936
15577	0.01	0.1247974	KLF1	0.080129856
15578	0.01	0.1248748	ZNF890P	0.08008024
15579	0.01	0.1249522	LOC344967	0.080030624
15580	0.01	0.1254187	ODF3L2	0.079732928
15581	0.1069264	1.3443417	GBGT1	0.07953809
15582	0.0610784	0.7679138	DIRAS1	0.07953809
15583	0.0432364	0.5435938	PLEKHA7	0.07953809
15584	0.098674	1.2405881	LOC399815	0.07953809
15585	0.1200689	1.5095767	MPP7	0.07953809
15586	0.1203925	1.5136465	HHIPL1	0.07953809
15587	0.0683277	0.8590564	ZNF680	0.07953809
15588	0.1336108	1.6798346	SLC25A53	0.07953809
15589	0.1371952	1.7248993	ASGR1	0.07953809
15590	0.0808063	1.0159449	TSPAN12	0.07953809
15591	0.0576167	0.7243909	FAM5B	0.079538089
15592	0.0579854	0.7290267	SLC5A5	0.079538089
15593	0.0503137	0.6325741	ANO4	0.079538089
15594	0.01	0.1260462	DLK1	0.079336
15595	0.01	0.1263623	CD2	0.079137536
15596	0.01	0.1263623	CES1P2	0.079137536
15597	0.01	0.1264416	AHSG	0.07908792
15598	0.01	0.1266402	SIGLEC11	0.07896388
15599	0.01	0.1267597	GPR115	0.078889456
15600	0.01	0.1273202	FAM83C	0.078542144
15601	0.01	0.1273202	GSTM5	0.078542144
15602	0.6608864	8.4155814	NOG	0.078531279
15603	0.01	0.1275619	SAMD13	0.078393296
15604	3.7303013	47.62109	FAM162A	0.078332967
15605	0.01	0.1278857	TPO	0.078194832
15606	0.01	0.1280482	AMN	0.078095599
15607	0.1458104	1.8684688	XDH	0.078037371

15608	0.01	0.1282111	CD53	0.077996368
15609	0.01	0.1286202	NPSR1	0.077748288
15610	0.7807662	10.067954	DNAJA4	0.077549638
15611	0.01	0.1291973	RPA4	0.077400976
15612	0.3683271	4.7733142	TRPV2	0.077163819
15613	0.01	0.1304348	CACNA1D	0.076666659
15614	0.01	0.1304939	RSPO1	0.076631927
15615	0.07779	1.0156386	CACNA2D2	0.076592235
15616	0.01	0.1307055	HLA-DQA1	0.076507887
15617	0.331553	4.3466214	CORO2A	0.076278332
15618	0.329833	4.3417939	ACVR2B	0.075966992
15619	0.9532541	12.555584	FBLN2	0.075922722
15620	0.3406612	4.4889079	EGFLAM	0.075889554
15621	0.01	0.1318168	EGOT	0.075862879
15622	0.01	0.1326409	SDR16C5	0.075391527
15623	0.01	0.1331228	RNF224	0.075118639
15624	4.9801351	66.318134	CRABP2	0.075094621
15625	0.3233314	4.3056528	ELFN2	0.075094621
15626	0.01	0.1332988	INSM2	0.075019407
15627	0.01	0.1334754	SERPINA6	0.074920175
15628	0.01	0.1335196	LINC00696	0.074895367
15629	0.01	0.1335638	ACTA1	0.074870559
15630	3.4546512	46.142899	KIAA0020	0.074868535
15631	0.01	0.134097	LOC728024	0.074572863
15632	0.01	0.1341863	OR6W1P	0.074523247
15633	0.01	0.1343652	SIX6	0.074424015
15634	0.01	0.1347245	APITD1-CORT	0.074225551
15635	0.0385711	0.5222413	ZNF91	0.073856798
15636	0.0681936	0.9233213	PPM1L	0.073856798
15637	0.0479189	0.6488089	SPNS2	0.073856798
15638	0.052666	0.7130831	FAM78A	0.073856798
15639	0.206234	2.7923502	GPR160	0.073856798
15640	0.3034944	4.109228	F12	0.073856798
15641	0.5175484	7.0074583	LHX2	0.073856798
15642	0.3211086	4.3477194	HOXD4	0.073856798
15643	1.9611642	26.553604	RBPMS2	0.073856798
15644	0.1994254	2.7001625	LINC00189	0.073856798
15645	0.8097115	10.963263	PHKA1	0.073856798
15646	0.1043041	1.4122472	SBSN	0.073856798
15647	0.1103811	1.4945285	TMEM150C	0.073856798
15648	0.1719468	2.3281104	NUDT7	0.073856798
15649	0.0350251	0.4742302	KIAA1045	0.073856798
15650	0.0628088	0.8504129	CHRNA3	0.073856797

15651	0.5830644	7.9538835	CYFIP2	0.073305628
15652	0.01	0.1366889	EXD1	0.073158807
15653	0.01	0.1367121	FGD5	0.073146403
15654	0.1471748	2.0211719	KIAA1199	0.072816561
15655	0.01	0.1375753	KCNE1L	0.072687455
15656	0.01	0.1378105	ANKRD18DP	0.072563415
15657	0.200336	2.7609294	ZFYVE28	0.072561064
15658	0.01	0.1382991	LOC100505676	0.072307065
15659	0.01	0.1383307	P2RX3	0.072290526
15660	0.01	0.1384257	LOC644936	0.072240911
15661	0.01	0.1386638	C19orf21	0.07211687
15662	0.01	0.1389027	DUOXA2	0.071992831
15663	0.01	0.1389985	CACNA1G-AS1	0.071943215
15664	0.01	0.1390465	POLN	0.071918407
15665	0.01	0.1390944	KCNK12	0.071893599
15666	0.01	0.1390944	GAL3ST2	0.071893599
15667	0.01	0.1391905	NOTO	0.071843983
15668	0.01	0.1392867	EYA1	0.071794366
15669	0.01	0.1392867	PACSIN1	0.071794366
15670	0.2472117	3.4496412	C3orf70	0.071663031
15671	0.01	0.1397212	ARX	0.071571095
15672	0.1430333	2.0034577	KCNN2	0.071393215
15673	0.1430333	2.0057953	RNFT2	0.071310012
15674	0.01	0.1403537	INHA	0.07124859
15675	0.01	0.1408441	RBM44	0.07100051
15676	0.3199817	4.509296	PCSK6	0.070960453
15677	0.01	0.1411894	LPAR5	0.070826854
15678	1.2065742	17.036814	GPHN	0.070821587
15679	0.01	0.1418352	BOLL	0.07050435
15680	0.379829	5.3876703	CEND1	0.070499671
15681	0.3132885	4.4539287	KCNQ5	0.070339807
15682	0.01	0.1428404	PRRG2	0.07000819
15683	1.2541595	17.991734	ZIC2	0.069707539
15684	0.01	0.1436037	AQP1	0.06963607
15685	0.01	0.1438087	ADH6	0.069536838
15686	0.01	0.1441687	ST8SIA6	0.069363182
15687	0.01	0.144427	PRR23C	0.069239142
15688	0.01	0.1445133	CR2	0.069197795
15689	0.01	0.1447381	LOC401242	0.069090294
15690	0.01	0.1450332	G6PC	0.068949715
15691	0.1796547	2.6062222	DEF6	0.068933011
15692	0.0972466	1.4107409	EFNA2	0.068933011
15693	2.1708281	31.491851	CRIP1	0.068933011

15694	0.4463882	6.4756805	SLC4A11	0.068933011
15695	0.1032703	1.4981257	DPY19L2P4	0.068933011
15696	0.1287742	1.8681057	TMTC2	0.068933011
15697	0.01	0.1452772	LOC730668	0.068833945
15698	0.01	0.1456271	FAM3B	0.068668558
15699	0.3758897	5.4832647	KIF21A	0.068552166
15700	3.0223042	44.203823	SFRP1	0.06837201
15701	0.01	0.1462611	CEACAM21	0.068370862
15702	0.01	0.1466869	SERHL2	0.068172398
15703	0.01	0.1469008	KCNQ4	0.068073166
15704	0.01	0.1470079	SRRM2-AS1	0.06802355
15705	0.01	0.1471958	KANK4	0.067936721
15706	0.01	0.1472765	LOC283177	0.067899509
15707	0.01	0.147546	EMR4P	0.06777547
15708	0.4783096	7.0775351	JAM2	0.067581384
15709	0.01	0.148306	RDH5	0.067428158
15710	0.01	0.1484589	TLL2	0.067358695
15711	0.01	0.1489086	DDX43	0.06715527
15712	0.01	0.1493224	TMEM215	0.066969209
15713	0.01	0.1496272	ACPT	0.066832765
15714	0.01	0.1496642	VWA5B2	0.066816227
15715	0.3126774	4.6871592	LOC401074	0.066709366
15716	0.5158403	7.7326526	RAMP2	0.066709366
15717	0.01	0.1502967	LPPR5	0.06653507
15718	0.01	0.1504088	STL	0.066485453
15719	0.01	0.1504088	EPO	0.066485453
15720	0.01	0.1504463	ZBTB8B	0.066468915
15721	0.01	0.1507463	SSTR5	0.066336605
15722	0.01	0.1510853	TMEM191B	0.066187757
15723	0.01	0.1513501	NLGN3	0.066071987
15724	0.0680451	1.0309914	ZFHX2	0.065999691
15725	3.7106111	56.425253	ITGA2	0.065761533
15726	0.01	0.1523415	BTC	0.065641981
15727	0.01	0.1525722	WTAPP1	0.065542749
15728	0.01	0.1528035	HES5	0.065443517
15729	0.2001916	3.0762453	PRKAA2	0.065076619
15730	0.01	0.153736	SHBG	0.065046589
15731	0.01	0.1538794	CSRNP3	0.064985947
15732	0.01	0.1545612	C6orf163	0.064699277
15733	0.0248627	0.3847251	MGAT4A	0.064624699
15734	0.0412264	0.6379358	PPFIA4	0.064624699
15735	0.0591204	0.9148271	SRCIN1	0.064624698
15736	0.1798098	2.7823689	DNAAF3	0.064624698

15737	0.1903192	2.9449914	RNF208	0.064624698
15738	0.0488742	0.7562771	GNG7	0.064624698
15739	0.01	0.1551562	CCDC160	0.064451197
15740	0.01	0.1551562	SFTPD	0.064451197
15741	0.01	0.1555153	ASTL	0.064302349
15742	0.01	0.1555453	KCTD16	0.064289945
15743	0.01	0.1556955	TBX21	0.064227925
15744	0.01	0.1560572	LOC728613	0.064079077
15745	2.5222955	39.389807	GRB10	0.064034219
15746	0.01	0.1561781	ALOX5	0.064029461
15747	0.01	0.1562992	LINC00552	0.063979845
15748	0.01	0.1567246	VSTM2A	0.063806189
15749	0.01	0.1569687	TMEM252	0.063706957
15750	0.01	0.1573118	NCAN	0.063568032
15751	0.7329799	11.535432	KCTD12	0.063541603
15752	0.01	0.1575824	DNASE2B	0.063458877
15753	0.2951834	4.6628161	SLC10A4	0.063305827
15754	0.01	0.1579776	PTPRO	0.063300105
15755	1.698909	26.886323	THBD	0.063188594
15756	0.1463223	2.3349408	EYA4	0.062666374
15757	0.5873718	9.3729974	FAM196B	0.062666374
15758	0.0998201	1.592882	IRAK3	0.062666374
15759	0.01	0.1595787	PIH1D2	0.06266502
15760	0.01	0.1599586	GPR151	0.062516173
15761	0.01	0.160468	NHLH2	0.062317709
15762	0.01	0.160468	TBC1D21	0.062317709
15763	0.01	0.1606599	LOC728084	0.062243285
15764	0.01	0.1609807	LOC284379	0.062119244
15765	0.01	0.1613674	ODF3L1	0.061970396
15766	0.01	0.1616262	C22orf24	0.061871164
15767	0.01	0.1622769	RSPH6A	0.061623084
15768	0.01	0.1623422	ZNF224	0.061598276
15769	0.01	0.1628012	TTC18	0.06142462
15770	0.01	0.1629328	NAGPA-AS1	0.061375004
15771	0.01	0.1630646	KLHL41	0.061325388
15772	0.01	0.1635277	FAM95A	0.061151732
15773	0.144321	2.3727932	C6orf147	0.060823245
15774	0.2174947	3.5758489	CELSR2	0.060823245
15775	0.0797091	1.3105043	CNTNAP3	0.060823245
15776	0.0652983	1.0735746	CNTNAP3B	0.060823245
15777	0.01	0.1647979	CST13P	0.06068038
15778	0.01	0.1647979	UPK1A	0.06068038
15779	0.01	0.1650679	C9orf173	0.060581148

15780	0.3718468	6.1535227	MAN1A1	0.060428289
15781	0.4864601	8.0606593	STRBP	0.060349913
15782	0.01	0.1657466	LOC339975	0.060333068
15783	0.01	0.1657466	LOC440700	0.060333068
15784	0.01	0.165883	SPATA12	0.060283452
15785	0.01	0.166225	DACH2	0.060159412
15786	0.01	0.1666373	FLJ33360	0.060010564
15787	0.01	0.1667062	LOC100652739	0.059985756
15788	0.01	0.1667062	LINC00242	0.059985756
15789	0.01	0.1673522	AGBL2	0.059754215
15790	0.1703074	2.8549409	PHF21B	0.059653568
15791	0.4473692	7.4994548	EFCAB4B	0.059653567
15792	0.01	0.1680966	DRD5	0.059489596
15793	0.01	0.1680966	TNFAIP8L2	0.059489596
15794	0.01	0.168942	DCAF4L1	0.0591919
15795	0.01	0.1689893	ARPP21	0.059175361
15796	0.2507816	4.2443891	FKBP1AP1	0.059085438
15797	0.01	0.1695104	ACRV1	0.058993436
15798	0.01	0.1696531	FAM179A	0.05894382
15799	0.01	0.1702262	FXYD6-FXYD2	0.058745356
15800	0.01	0.1705143	HESX1	0.058646124
15801	0.3125993	5.341018	GABRQ	0.058528028
15802	0.01	0.1712386	SLC6A13	0.058398044
15803	0.01	0.1713842	CEACAM1	0.058348428
15804	0.01	0.1713842	C10orf131	0.058348428
15805	0.09564	1.6417964	NKD1	0.058253249
15806	0.01	0.1716762	KRTAP4-11	0.058249196
15807	1.9678895	33.876786	TNNT1	0.058089616
15808	0.01	0.1725581	C13orf45	0.0579515
15809	0.2118804	3.6591839	CHST6	0.057903729
15810	0.01	0.1734491	ULK4P3	0.057653804
15811	0.01	0.1735487	LOC100505495	0.057620726
15812	0.01	0.1735985	H19	0.057604188
15813	0.01	0.1737481	LOC653653	0.057554572
15814	0.0629986	1.0966932	FAM134B	0.057444176
15815	0.1167831	2.0329847	SNCAIP	0.057444176
15816	0.147045	2.5597892	NEGR1	0.057444176
15817	0.2343872	4.0802601	SHANK3	0.057444176
15818	0.4098894	7.1354394	IGFBPL1	0.057444176
15819	0.0581229	1.0118146	EML5	0.057444176
15820	0.1376938	2.3970012	PRDM6	0.057444176
15821	0.0570645	0.9933903	GPR132	0.057444176
15822	0.051444	0.8955471	C2orf88	0.057444175

15823	0.01	0.174702	C2CD4A	0.057240337
15824	0.3206146	5.6433397	TRPV4	0.056812921
15825	0.01	0.1763323	KRTAP4-8	0.056711099
15826	0.01	0.1764095	GRB7	0.056686291
15827	0.01	0.1766414	SYCP3	0.056611867
15828	0.01	0.1766414	FLJ34208	0.056611867
15829	0.01	0.1776622	DCC	0.056286607
15830	0.01	0.1784399	C2orf66	0.056041283
15831	0.01	0.1785189	MTUS2-AS1	0.056016475
15832	0.01	0.1785453	KNDC1	0.056008206
15833	0.0717506	1.2837442	KIAA1804	0.055891631
15834	0.1288405	2.305184	FCHO1	0.055891631
15835	0.2407851	4.3080707	EPCAM	0.055891631
15836	0.1077557	1.9279396	REEP1	0.055891631
15837	3.5284571	63.130331	WARS2	0.055891631
15838	0.01	0.1792811	GRIA4	0.055778318
15839	0.01	0.179313	LOC100128675	0.055768395
15840	0.01	0.1805984	LPIN3	0.055371467
15841	0.01	0.1810852	GABRB1	0.055222619
15842	1.2857553	23.315304	RIPK4	0.055146409
15843	0.01	0.1814112	PPP1R42	0.055123387
15844	0.01	0.1814112	NBPF6	0.055123387
15845	0.01	0.1817384	C3orf55	0.055024155
15846	0.01	0.1819024	HNRNPCL1	0.054974539
15847	0.01	0.1820667	GATA3-AS1	0.054924923
15848	0.01	0.1823962	FANCD2OS	0.054825691
15849	0.01	0.182727	CD6	0.054726459
15850	0.01	0.1835591	DHDH	0.054478379
15851	0.1687445	3.1007361	LIN7A	0.054420798
15852	0.5184067	9.5258933	CYP11A1	0.054420798
15853	0.4261748	7.8311024	TMED8	0.054420798
15854	0.0828297	1.5220227	C10orf55	0.054420798
15855	0.01	0.1838102	ZNF732	0.054403955
15856	0.01	0.1847368	CEP170P1	0.054131067
15857	0.01	0.1850761	C19orf38	0.054031835
15858	0.01	0.1854166	GADD45G	0.053932603
15859	1.6202099	30.06569	MOK	0.053888999
15860	0.01	0.1857584	NELL1	0.053833371
15861	0.01	0.1865033	MESTIT1	0.053618368
15862	0.01	0.186532	EMR2	0.053610099
15863	0.01	0.1869068	RASGEF1A	0.053502598
15864	0.01	0.1871382	KRTAP4-12	0.053436443
15865	3.9382583	73.834753	ZNF697	0.053338816

15866	0.01	0.1874864	GPR89B	0.053337211
15867	0.01	0.1876027	ACPP	0.053304133
15868	0.01	0.1880111	AMTN	0.053188363
15869	13.530415	254.86778	PLAU	0.053087979
15870	0.3984694	7.5146903	NMB	0.053025393
15871	0.01	0.1888921	PTPN5	0.052940283
15872	0.01	0.1892024	KLHL14	0.052853455
15873	0.01	0.1893357	MFNG	0.052816243
15874	0.01	0.1894247	FAM9B	0.052791435
15875	0.01	0.1901395	ANKRD53	0.052592971
15876	1.3677342	26.037615	TFAP2C	0.052529166
15877	0.01	0.1903789	KCNC3	0.052526816
15878	0.7261307	13.844501	HOMER2	0.05244903
15879	0.01	0.1910406	CELA3B	0.052344891
15880	0.5994233	11.497696	NAGS	0.05213421
15881	0.01	0.1923166	IL17C	0.051997578
15882	0.01	0.1932386	MYL10	0.051749498
15883	0.0757403	1.4650034	FAM84B	0.051699759
15884	0.1689497	3.2679019	S1PR5	0.051699759
15885	0.1294809	2.5044778	GPM6B	0.051699759
15886	0.3548232	6.8631503	PPM1J	0.051699758
15887	0.2598498	5.0261309	ZNF239	0.051699758
15888	0.0934108	1.8067938	VGLL2	0.051699758
15889	0.162685	3.1642085	PLEKHG4B	0.051414124
15890	0.01	0.1951092	CPNE9	0.051253338
15891	0.01	0.1952983	DBX1	0.051203722
15892	0.01	0.1962491	KHDC3L	0.050955642
15893	0.01	0.1972092	NAP1L6	0.050707562
15894	1.1174236	22.045984	RNF130	0.050686038
15895	0.01	0.1975959	PLA2G10	0.05060833
15896	0.01	0.1977898	LOC150935	0.050558714
15897	0.01	0.1981009	COL2A1	0.050479328
15898	0.120115	2.3814011	BCAN	0.050438789
15899	0.1201843	2.3827744	TTYH2	0.050438789
15900	0.01	0.1999483	LOC339894	0.050012938
15901	0.01	0.2000475	ESPNP	0.04998813
15902	0.01	0.2000475	ZNF833P	0.04998813
15903	0.01	0.2003458	ANKRD31	0.049913706
15904	0.3391367	6.8057247	KCNAB3	0.049831092
15905	5.8943764	118.63893	AKAP12	0.049683322
15906	0.01	0.2013063	NCAM2	0.049675549
15907	0.01	0.2013465	PRTN3	0.049665626
15908	0.3578512	7.2363417	NRCAM	0.049451943

15909	0.01	0.2025607	LOC731656	0.04936793
15910	0.01	0.2030368	ACHE	0.049252159
15911	0.0856554	1.7396238	KHK	0.049237865
15912	0.2114658	4.2947791	SHISA3	0.049237865
15913	0.1769096	3.5929582	ACOX2	0.049237865
15914	0.072689	1.4762835	MCOLN3	0.049237865
15915	0.7523448	15.279801	RMRP	0.049237865
15916	0.01	0.2031732	HTR6	0.049219082
15917	0.01	0.2031732	NOL4	0.049219082
15918	0.01	0.2035837	FLJ30679	0.04911985
15919	0.01	0.2037895	CDC42BPG	0.049070234
15920	0.01	0.2038926	LOC151174	0.049045426
15921	1.1444234	23.35343	NXPH4	0.04900451
15922	0.01	0.2042025	NPBWR1	0.048971002
15923	0.01	0.2042715	LOC643711	0.048954463
15924	0.01	0.2042853	FRMPD1	0.048951155
15925	0.01	0.2050334	PTENP1	0.048772538
15926	0.6810441	14.051266	FEZ1	0.048468524
15927	0.1818042	3.767719	SFMBT2	0.048253108
15928	0.01	0.2073538	LOC643923	0.048226762
15929	0.01	0.2075673	NKX1-2	0.048177146
15930	0.1916758	3.9855403	OSBP2	0.048092799
15931	0.01	0.2086417	FONG	0.047929066
15932	0.01	0.2086417	CCDC110	0.047929066
15933	0.01	0.2090745	LOC255654	0.047829834
15934	0.01	0.2104938	SPNS3	0.04750733
15935	0.01	0.2106038	MPZL2	0.047482522
15936	0.01	0.2108793	NLRP14	0.047420502
15937	0.01	0.2109344	CNGA4	0.047408098
15938	0.01	0.2117099	RAB19	0.047234442
15939	0.0667092	1.419351	C2CD4C	0.04699978
15940	0.1574906	3.3508805	MARK1	0.04699978
15941	0.01	0.2137305	SRD5A3-AS1	0.046787897
15942	0.01	0.2141847	CELA2B	0.046688665
15943	0.01	0.2142132	KCNK10	0.046682463
15944	0.01	0.2144126	FGF21	0.046639049
15945	0.01	0.2144126	CST7	0.046639049
15946	0.01	0.2147172	LAIR1	0.046572895
15947	0.01	0.2148316	RET	0.046548087
15948	0.01	0.2153289	LINC00087	0.046440585
15949	0.01	0.2154824	SRCRB4D	0.046407508
15950	12.85719	277.65778	CKB	0.046305886
15951	0.01	0.2160213	CGB	0.046291737

15952	0.01	0.2162531	LOC389705	0.046242121
15953	0.01	0.2167181	OR5B21	0.046142889
15954	0.01	0.2167181	LINC00692	0.046142889
15955	0.01	0.2170682	ANGPTL6	0.046068465
15956	0.01	0.2175368	SYT5	0.045969233
15957	0.1065778	2.3191595	FRAS1	0.045955341
15958	0.1521275	3.310334	DYNC2H1	0.045955341
15959	0.1609263	3.5017966	NAP1L2	0.045955341
15960	0.01	0.2178896	OR2L1P	0.045894809
15961	0.01	0.2181254	TAS2R10	0.045845193
15962	0.01	0.2187409	TTN-AS1	0.045716192
15963	0.01	0.2187765	TSGA10	0.045708749
15964	0.01	0.2205923	CADPS	0.045332495
15965	0.01	0.2209954	OR10A2	0.045249801
15966	0.01	0.2214812	LINC00575	0.045150569
15967	0.01	0.2216435	PHACTR3	0.045117492
15968	0.01	0.221969	C1orf68	0.045051337
15969	0.01	0.2221088	GPRIN3	0.045022985
15970	0.01	0.2222954	CTNND2	0.044985182
15971	0.4597048	10.225589	CAMK2N2	0.044956312
15972	0.545263	12.12873	PGF	0.044956312
15973	0.218907	4.8693282	TMEM108	0.044956312
15974	0.1490164	3.3146947	RASEF	0.044956312
15975	0.0613662	1.3650178	STOX1	0.044956312
15976	0.0759197	1.6887433	SH3GL2	0.044956312
15977	0.01	0.2227869	CRHR1	0.04488595
15978	0.01	0.2233217	C20orf166-AS1	0.044778449
15979	0.01	0.2236935	PEBP4	0.044704025
15980	0.01	0.2241081	ZNF311	0.044621332
15981	0.01	0.2243908	IGDCC3	0.0445651
15982	6.8153132	153.04042	OAF	0.044532766
15983	1.0821933	24.371142	CYB5R2	0.0444047
15984	0.01	0.225319	C9orf171	0.044381521
15985	0.3595575	8.1146243	UGT8	0.044309809
15986	0.01	0.2263311	PRRT4	0.044183057
15987	0.01	0.2263311	LINC00461	0.044183057
15988	0.01	0.226628	ZNF214	0.044125172
15989	0.01	0.2271388	KCNN1	0.04402594
15990	0.01	0.2279953	KIRREL3-AS3	0.043860553
15991	0.01	0.2281243	BARX2	0.043835745
15992	0.01	0.2281502	ZNF10	0.043830783
15993	0.01	0.2287717	TGIF2LX	0.043711705
15994	0.01	0.2290316	CGB7	0.043662089

15995	0.01	0.2295534	CCL21	0.043562857
15996	0.4451079	10.245277	HOXD13	0.043445175
15997	0.01	0.2306039	PRG2	0.043364393
15998	0.01	0.2315311	CNTN4	0.043190737
15999	0.01	0.2317974	MTRNR2L5	0.043141121
16000	0.01	0.2317974	LOC440910	0.043141121
16001	0.01	0.2319575	ROBO2	0.043111351
16002	0.0151607	0.351895	AFF2	0.043083133
16003	0.0622274	1.444356	FBXO43	0.043083132
16004	0.01	0.2327342	SHD	0.042967465
16005	0.5884434	13.702102	ST6GAL1	0.042945486
16006	0.01	0.2335433	IFNA1	0.042818617
16007	0.01	0.2338142	BLID	0.042769001
16008	0.01	0.2345398	PPP2R2B	0.042636691
16009	0.01	0.2355907	GPR143	0.042446497
16010	0.01	0.2357285	MYL2	0.042421689
16011	1.1802271	27.913011	WSCD1	0.04228233
16012	1.5902841	37.611079	F2RL1	0.04228233
16013	0.01	0.2368365	PACRG	0.042223225
16014	0.01	0.2368713	KCNC1	0.042217023
16015	0.3318463	7.8629335	TGFBR3L	0.042203884
16016	0.01	0.2386594	HSD3B1	0.04190072
16017	0.01	0.239084	CRYGS	0.041826296
16018	0.5896986	14.143709	LAT	0.041693354
16019	0.01	0.2398808	NPR1	0.041687372
16020	0.01	0.2402239	FOXN4	0.041627832
16021	0.01	0.2416641	ANKRD55	0.041379752
16022	0.0879323	2.1260321	WNT10B	0.041359807
16023	0.1492833	3.6093812	PTP4A3	0.041359807
16024	0.1142541	2.7624431	C4orf19	0.041359807
16025	0.424151	10.255148	ARC	0.041359807
16026	0.117276	2.8355071	MMP10	0.041359807
16027	0.0886806	2.144126	SOX17	0.041359807
16028	0.0531767	1.2857097	CXorf57	0.041359807
16029	0.01	0.242245	HLA-DQB1	0.04128052
16030	0.01	0.2436114	ZMAT4	0.041048979
16031	0.504355	12.291882	NPAS1	0.041031554
16032	0.01	0.2443004	NOXA1	0.040933208
16033	0.01	0.2448941	SIRPB1	0.040833976
16034	0.01	0.245391	PTPN6	0.040751283
16035	0.3378721	8.2997979	FGFR4	0.040708471
16036	0.01	0.2457901	GKN1	0.040685128
16037	0.01	0.246391	LOC100288974	0.040585896

16038	0.1279702	3.1559534	FIBCD1	0.04054883
16039	0.01	0.247754	FAM47E	0.040362624
16040	0.01	0.2480589	COL24A1	0.040313008
16041	0.01	0.2482116	KRTAP2-4	0.0402882
16042	0.01	0.2494404	KCNIP4	0.040089736
16043	0.01	0.2494404	AOC3	0.040089736
16044	0.01	0.2499043	ART3	0.040015312
16045	0.01	0.2505256	LOC100499484	0.03991608
16046	0.01	0.2509936	LOC100508120	0.039841656
16047	0.01	0.2513065	PRSS2	0.03979204
16048	0.0669449	1.6833421	NTNG2	0.039769045
16049	0.0789392	1.9849409	FAM43B	0.039769045
16050	0.1612999	4.0559164	TMEM200B	0.039769045
16051	0.1820878	4.5786317	SLC7A3	0.039769045
16052	0.01	0.2522501	ATP5L2	0.039643192
16053	0.01	0.2525662	MEDAG	0.039593576
16054	0.01	0.2528831	PLEKHB1	0.03954396
16055	0.01	0.2537108	KIF6	0.039414958
16056	0.01	0.2537587	CD8A	0.039407516
16057	0.4782547	12.141437	E2F5	0.039390292
16058	0.01	0.2549087	FAIM2	0.039229725
16059	0.01	0.2554472	LCN1	0.039147032
16060	0.01	0.2557714	LCA5L	0.039097416
16061	0.01	0.2564222	SPINK2	0.038998184
16062	0.01	0.2566944	FIGNL2	0.038956837
16063	0.01	0.2575362	DPP4	0.038829489
16064	0.01	0.2575691	IQCJ	0.038824528
16065	0.01	0.2575691	SLC22A18AS	0.038824528
16066	0.01	0.2577998	C7orf63	0.038789797
16067	0.1536303	3.9744968	RASSF2	0.038654025
16068	0.01	0.2587264	LOC100130238	0.038650872
16069	0.01	0.2587264	IRX6	0.038650872
16070	0.1992347	5.2024779	CYTH4	0.038296117
16071	0.0742428	1.9386505	FAM53A	0.038296117
16072	1.2388345	32.348829	TUBA4A	0.038296117
16073	0.2803677	7.3210488	CHD5	0.038296117
16074	0.0491277	1.2828364	TSPAN18	0.038296117
16075	0.0810262	2.1157822	SLC38A3	0.038296117
16076	0.01	0.261411	CD3D	0.038253944
16077	0.01	0.2617505	NACA2	0.038204328
16078	0.01	0.262034	LRRC16B	0.038162981
16079	0.01	0.2622614	AURKAPS1	0.038129904
16080	0.194584	5.1124026	AUTS2	0.038061172

16081	0.2912234	7.6608461	ZNF853	0.038014528
16082	3.4375175	90.592641	S100A2	0.037944777
16083	0.01	0.2639788	KLK6	0.037881824
16084	0.01	0.2641518	LSMEM2	0.037857016
16085	0.1378912	3.6476134	FLT4	0.037803117
16086	0.01	0.265719	REG4	0.037633744
16087	0.01	0.2658943	CHI3L2	0.037608936
16088	0.01	0.2667153	KCNA2	0.037493165
16089	0.4995793	13.335061	DOCK4	0.037463593
16090	0.01	0.2670097	SNAP91	0.037451818
16091	0.01	0.2671277	PCDHAC2	0.03743528
16092	0.01	0.2673048	PNMA6C	0.037410472
16093	0.01	0.2680158	FABP6	0.03731124
16094	0.01	0.2697581	KCNJ12	0.037070247
16095	0.01	0.2701714	HCG17	0.037013543
16096	0.0205441	0.5563229	PCLO	0.036928399
16097	0.069629	1.8855128	SLC17A7	0.036928399
16098	0.1508502	4.0849365	FGF11	0.036928399
16099	0.1122237	3.0389552	NKX2-8	0.036928399
16100	0.2448159	6.629474	ARHGAP4	0.036928399
16101	0.001908	0.0516676	TTN	0.036928399
16102	0.1874096	5.0749458	MSX2	0.036928399
16103	0.0804942	2.1797372	HTRA3	0.036928399
16104	0.042152	1.1414522	RNF180	0.036928398
16105	0.01	0.2716278	C1orf194	0.036815079
16106	0.01	0.2731001	SLN	0.036616615
16107	0.01	0.2738422	DMRT2	0.036517383
16108	0.1739078	4.7653872	C2orf72	0.036493947
16109	0.01	0.2744014	C10orf95	0.036442959
16110	0.01	0.2750568	BEAN1	0.036356131
16111	0.01	0.2760929	C21orf37	0.036219687
16112	0.01	0.2764717	CCDC173	0.036170071
16113	0.01	0.2765981	CBFA2T3	0.036153533
16114	1.3925794	38.585672	BACE2	0.036090582
16115	0.01	0.2778925	RP1L1	0.035985139
16116	0.01	0.2791521	LINC00494	0.035822759
16117	0.01	0.2791521	MRGPRX4	0.035822759
16118	0.01	0.280161	KCNK2	0.035693758
16119	0.01	0.280187	DDX11L2	0.03569045
16120	0.0345089	0.9678569	TPPP	0.035655006
16121	0.2893098	8.1141429	KCNK1	0.035655006
16122	0.4486534	12.583181	FAM20C	0.035655006
16123	0.0865267	2.426775	PIK3AP1	0.035655006

16124	0.01	0.2813168	FOXP2	0.035547115
16125	0.01	0.2814914	FAM229A	0.035525063
16126	0.01	0.2826758	SPI1	0.035376215
16127	0.01	0.2829933	USH1G	0.035336522
16128	0.01	0.283471	TMEM238	0.035276983
16129	0.01	0.2843709	PROX1	0.035165347
16130	0.01	0.2854785	BHLHA15	0.035028903
16131	0.01	0.2854785	HLA-DMB	0.035028903
16132	0.01	0.2858835	ANP32C	0.034979287
16133	0.01	0.286493	PITX3	0.034904863
16134	0.01	0.2866968	HCG25	0.034880055
16135	0.01	0.2866968	FLJ11235	0.034880055
16136	0.01	0.2866968	FAM27B	0.034880055
16137	0.01	0.2868328	SLC30A3	0.034863516
16138	0.01	0.2869009	SHISA8	0.034855247
16139	0.01	0.2885438	HAR1A	0.034656783
16140	0.01	0.2904148	HLA-DOB	0.034433511
16141	0.01	0.2925223	DRD4	0.034185431
16142	0.01	0.2948761	HNF4G	0.033912543
16143	0.01	0.295092	MYLPF	0.033887735
16144	0.01	0.2952361	ST6GALNAC5	0.033871196
16145	0.01	0.2955247	SPRR2D	0.033838119
16146	1.0080424	29.914052	WNT7B	0.033697956
16147	0.01	0.2968304	TTC36	0.033689271
16148	0.01	0.2977073	HNRNPA1P10	0.033590039
16149	0.01	0.298977	FREM2	0.033447393
16150	0.01	0.2997737	LOC100129858	0.033358497
16151	0.0371677	1.1143184	HLF	0.033354683
16152	0.054842	1.6442061	CLIC6	0.033354683
16153	0.1864038	5.588536	CDYL2	0.033354683
16154	0.0282576	0.8471842	ARAP2	0.033354682
16155	0.01	0.3003694	BEND5	0.033292343
16156	0.01	0.3006382	PARD6G-AS1	0.033262573
16157	2.3199019	69.756476	UBASH3B	0.033257155
16158	0.3618047	10.882184	BTBD11	0.033247433
16159	0.01	0.3023974	ADAM21	0.033069071
16160	0.01	0.3035359	RNF148	0.032945031
16161	0.01	0.3038792	FEZF1-AS1	0.032907819
16162	0.01	0.3039937	STARD6	0.032895415
16163	0.01	0.3041084	DNMBP-AS1	0.032883011
16164	0.01	0.3042614	SLIT1	0.032866472
16165	0.01	0.3044529	AKR7A2P1	0.032845799
16166	0.3373525	10.277227	FHOD3	0.032825243

16167	0.01	0.3053755	TIGD3	0.032746567
16168	0.01	0.3058389	LOC441242	0.032696951
16169	0.1637932	5.016255	POU4F1	0.032652479
16170	0.01	0.3063037	FLJ42875	0.032647335
16171	0.01	0.3063037	RPL13AP5	0.032647335
16172	0.01	0.3067699	RPL29P2	0.032597719
16173	0.4150113	12.751112	CDH4	0.032547063
16174	0.01	0.3077066	ANXA2P2	0.032498487
16175	0.01	0.3080201	C9orf62	0.032465409
16176	0.01	0.3083343	ARTN	0.032432332
16177	0.01	0.3094785	ZNF879	0.032312427
16178	0.0888697	2.7503331	CACNG7	0.032312349
16179	0.3593095	11.119881	PRSS3	0.032312349
16180	0.0608022	1.8817013	KIAA1324L	0.032312349
16181	0.0328343	1.0161543	MMP16	0.032312349
16182	0.01	0.3095973	PIH1D3	0.032300023
16183	0.01	0.3100736	KRT18P55	0.032250407
16184	0.01	0.3103123	CDR1	0.032225598
16185	0.01	0.3109507	ADRA2A	0.032159444
16186	0.01	0.311649	PSD2	0.032087383
16187	0.01	0.3122352	MGC16142	0.032027134
16188	0.01	0.3127197	C3orf49	0.031977518
16189	0.01	0.3132868	TMEM221	0.031919633
16190	0.2171959	6.82679	PPARG	0.031815236
16191	0.1091097	3.4294791	GLDC	0.031815236
16192	0.01	0.3149185	BTF3P11	0.031754246
16193	0.01	0.3154113	FAM157B	0.03170463
16194	0.01	0.3161535	FAM157A	0.031630206
16195	0.01	0.3164016	FAM90A25P	0.031605398
16196	0.01	0.3170986	ACRC	0.031535936
16197	0.1823587	5.7847124	LOC100288181	0.031524243
16198	0.1110872	3.5453513	CYP2J2	0.031333187
16199	0.0347217	1.1081438	MIR600HG	0.031333187
16200	0.01	0.3194102	FLJ21408	0.031307702
16201	0.01	0.3200188	RSPO2	0.031248163
16202	0.01	0.3202222	SYN3	0.031228317
16203	0.01	0.3202561	CNTN1	0.031225009
16204	0.01	0.3216189	PLA2G7	0.0310927
16205	0.01	0.3224766	RNASEH2B-AS1	0.031010006
16206	0.01	0.3226831	TOX3	0.03099016
16207	0.1780431	5.7683493	P2RX5	0.030865527
16208	0.01	0.3256023	HRH2	0.03071231
16209	0.01	0.3277201	HCG23	0.030513846

16210	0.01	0.3278978	LINC00671	0.030497307
16211	0.0447786	1.4724166	STOX2	0.030411623
16212	0.0415138	1.3650651	SPATA6	0.030411623
16213	0.2464808	8.1048218	SH2D2A	0.030411623
16214	0.0180604	0.5938666	MBNL3	0.030411622
16215	0.01	0.3288787	EPHA6	0.030406345
16216	0.01	0.3293265	ZMYND15	0.030364998
16217	1.0762055	35.46231	HOXB9	0.030347867
16218	0.01	0.3301357	KCNJ2-AS1	0.030290574
16219	0.386402	12.830302	FAM50B	0.030116364
16220	0.01	0.3331369	TPH1	0.030017686
16221	0.01	0.333965	MGC39584	0.029943262
16222	0.01	0.333965	C15orf27	0.029943262
16223	0.01	0.3342419	MAMDC2	0.029918454
16224	0.4440781	14.847665	POLR3G	0.029908951
16225	0.01	0.3346118	HSD3B2	0.029885377
16226	0.01	0.3352426	ISM2	0.029829145
16227	0.01	0.3364739	STX19	0.02971999
16228	0.01	0.3368488	HAPLN2	0.029686913
16229	0.01	0.3368957	PCDH20	0.029682778
16230	0.01	0.3372929	SEZ6L	0.029647821
16231	0.01	0.3380257	LINC00663	0.029583546
16232	0.0848878	2.8733909	SPSB4	0.029542719
16233	0.01	0.3388498	NPY1R	0.029511603
16234	0.01	0.339449	WNT10A	0.029459506
16235	0.3011191	10.241203	CSPG4	0.029402706
16236	0.01	0.3401651	SLC25A52	0.029397486
16237	0.5158403	17.647909	C17orf96	0.029229545
16238	0.1102935	3.7866888	SYPL2	0.029126624
16239	0.01	0.3437428	FAM106CP	0.029091521
16240	0.01	0.3439383	PVALB	0.029074982
16241	1.2505757	43.022215	RPP25	0.029068137
16242	0.01	0.3445262	PLA2G1B	0.029025366
16243	0.01	0.3451162	ASIP	0.02897575
16244	0.01	0.3454119	EFTUD1P1	0.028950942
16245	0.7386024	25.55064	JAM3	0.028907392
16246	0.3800599	13.15064	KCTD15	0.028900486
16247	0.01	0.3463021	CBLN2	0.028876518
16248	0.4839934	16.7729	FERMT1	0.028855679
16249	0.01	0.3480213	MAP3K15	0.028733872
16250	0.0667092	2.3225744	NSG1	0.028722088
16251	0.0716642	2.4950903	LMX1B	0.028722088
16252	0.0595597	2.073656	RAB39B	0.028722088

16253	0.01	0.3482167	RGS9BP	0.028717747
16254	0.01	0.3484977	KRT222	0.028694592
16255	0.01	0.3486987	GOLGA6L7P	0.028678054
16256	0.01	0.349606	MAL	0.02860363
16257	0.237087	8.2927347	CACHD1	0.028589728
16258	0.01	0.3517414	FDCSP	0.028429974
16259	0.01	0.3517414	CHRNA9	0.028429974
16260	0.01	0.3523564	LOC100131626	0.028380358
16261	0.2376729	8.3898458	FRMD4B	0.028328635
16262	0.01	0.3542141	VSIG2	0.02823151
16263	0.01	0.3548378	SELV	0.028181894
16264	0.01	0.3548378	B3GALT4	0.028181894
16265	0.01	0.3556727	WNT6	0.028115739
16266	0.01	0.3560916	LOC100129726	0.028082662
16267	0.01	0.3564064	FLJ40852	0.028057854
16268	0.0335912	1.20201	MFAP3L	0.027945815
16269	0.194584	6.9629042	NDRG2	0.027945815
16270	0.0722481	2.5852905	IQGAP2	0.027945815
16271	0.01	0.3579891	LOC441461	0.027933814
16272	0.01	0.3592653	LINC00235	0.027834582
16273	1.4341146	51.609681	GRAMD1B	0.027787706
16274	0.01	0.3623332	KGFLP1	0.027598906
16275	0.01	0.3627136	DGCR5	0.027569963
16276	0.01	0.3631493	GJB3	0.027536886
16277	0.01	0.3634767	C16orf98	0.027512078
16278	0.01	0.364298	TPTE2	0.027450058
16279	0.01	0.3644627	IRGM	0.027437654
16280	0.1616126	5.9002956	TRIM58	0.027390601
16281	0.2381164	8.7048773	RAVER2	0.02735437
16282	5.0988104	187.23632	KRAS	0.027231952
16283	0.1758646	6.4631377	CMTM8	0.027210399
16284	0.01	0.3684604	LOC643648	0.027139957
16285	0.01	0.3684604	EMX1	0.027139957
16286	0.5368354	19.83289	STXBP2	0.027067936
16287	0.01	0.3698126	HOXB-AS3	0.027040725
16288	0.01	0.3699823	SPON2	0.027028321
16289	0.01	0.3700842	PTPRZ1	0.027020879
16290	0.01	0.3723748	IVL	0.026854665
16291	0.01	0.3742764	RAB41	0.026718221
16292	1.6722126	62.667836	LY6K	0.026683746
16293	0.01	0.3749727	S100A14	0.026668605
16294	0.01	0.3760221	PTH1R	0.026594181
16295	0.01	0.3763732	FAM66D	0.026569373

16296	0.030575	1.1532227	MAPT	0.026512697
16297	0.01	0.3774304	SP8	0.026494949
16298	0.01	0.3779967	ADRA1D	0.026455257
16299	0.01	0.3784936	LINGO3	0.026420525
16300	0.01	0.3803986	CASS4	0.026288216
16301	0.01	0.381238	SNTN	0.026230331
16302	0.01	0.3819907	GRM4	0.026178647
16303	0.01	0.3846333	HCST	0.025998789
16304	0.01	0.3851233	FBXL22	0.025965712
16305	0.152172	5.8867571	PIANP	0.025849879
16306	0.9094741	35.182916	CAPG	0.025849879
16307	0.3307929	12.796689	HENMT1	0.025849879
16308	0.46017	17.857262	CORO2B	0.02576935
16309	0.01	0.3902185	C11orf65	0.025626669
16310	0.01	0.3921164	BRWD1-IT2	0.025502629
16311	0.01	0.3927277	PREX2	0.025462936
16312	0.01	0.3928808	ARL13A	0.025453013
16313	0.01	0.3928808	FGF22	0.025453013
16314	0.01	0.3951919	PCOLCE-AS1	0.025304165
16315	0.01	0.3960461	ADAMTS19	0.025249588
16316	0.0610247	2.4197545	ALOXE3	0.025219394
16317	0.2773114	10.995957	TAGLN3	0.025219394
16318	0.0540876	2.1446825	KCNH3	0.025219394
16319	0.01	0.3967477	TFF1	0.025204933
16320	0.01	0.3977918	NOX5	0.025138778
16321	0.01	0.4006915	PKIB	0.024956853
16322	0.01	0.4014897	CRYM	0.024907237
16323	0.01	0.4022911	LOC731223	0.024857621
16324	0.01	0.4053657	LMTK3	0.02466908
16325	0.01	0.4055289	BHLHE40-AS1	0.024659157
16326	0.0294599	1.1966369	MYH15	0.024618933
16327	0.2036146	8.2706493	PCOLCE2	0.024618933
16328	0.0824365	3.3485006	CDK5R2	0.024618933
16329	0.01	0.4076615	PABPC4L	0.024530155
16330	0.01	0.4092342	STXBP6	0.024435885
16331	0.01	0.4116021	FAM196A	0.024295306
16332	0.3924661	16.22631	SULF2	0.024187021
16333	0.01	0.4147075	PTGER1	0.024113381
16334	0.01	0.4147075	PABPC3	0.024113381
16335	0.0718619	2.9884681	TSPAN33	0.024046399
16336	0.0711747	2.9598898	KCNIP3	0.024046399
16337	0.0654316	2.7210541	VIPR1	0.024046399
16338	0.01	0.4168518	LEMD1	0.023989341

16339	0.01	0.4190184	HIST1H2AH	0.023865301
16340	0.1685398	7.0904419	TRIM9	0.023770004
16341	0.01	0.4207679	TPRG1-AS2	0.023766069
16342	3.0191887	127.30875	CLDN11	0.023715486
16343	0.01	0.4225322	UGT3A2	0.023666837
16344	0.0531631	2.2622718	TBC1D30	0.02349989
16345	0.0955961	4.0679382	PLEKHH1	0.02349989
16346	0.0130543	0.5555065	BSN	0.02349989
16347	0.01	0.4279148	MEIOB	0.023369141
16348	0.01	0.4280662	BAI3	0.023360871
16349	0.01	0.4283695	AQP3	0.023344333
16350	0.01	0.4291905	GLUD2	0.023299678
16351	0.01	0.4297395	KRTAP4-7	0.023269909
16352	0.01	0.430888	PPP1R1B	0.023207889
16353	0.1578386	6.8183316	SEMA6B	0.023149146
16354	0.01	0.4319847	MYO7A	0.02314897
16355	0.01	0.4322742	PKNOX2	0.023133465
16356	0.01	0.4334362	OR2A20P	0.023071445
16357	0.0415967	1.81031	SBK1	0.022977671
16358	0.042246	1.8385674	MAST1	0.02297767
16359	0.01	0.4375134	LOC728537	0.022856442
16360	0.01	0.4382836	SERTM1	0.022816277
16361	0.01	0.4389654	PIWIL4	0.022780837
16362	0.359723	15.887258	LPAR3	0.02264223
16363	0.01	0.4421294	LHFPL3	0.022617813
16364	0.01	0.4423142	CATSPER3	0.022608362
16365	0.01	0.4432406	FAM84A	0.022561109
16366	0.01	0.4433683	MAP10	0.022554611
16367	0.0324964	1.4456886	SYT7	0.022478156
16368	0.0748293	3.328977	ARSG	0.022478156
16369	0.01	0.4454096	ZNF32-AS2	0.022451245
16370	0.01	0.4463961	RNASE1	0.022401629
16371	0.1285095	5.7377959	DSP	0.022397007
16372	0.01	0.4484536	FAM154B	0.022298852
16373	0.01	0.4488816	PRKCG	0.022277588
16374	0.01	0.4498836	CHST13	0.022227972
16375	0.01	0.4501348	GAL3ST1	0.022215568
16376	0.01	0.450746	EFS	0.022185444
16377	0.01	0.45089	ENO1-AS1	0.022178356
16378	0.01	0.4539366	CYP1B1-AS1	0.022029508
16379	0.01	0.4539366	C8G	0.022029508
16380	0.01	0.4549613	GDF10	0.021979892
16381	0.01	0.4559906	ACAN	0.021930276

16382	0.01	0.4566264	ANKRD6	0.021899743
16383	0.1167131	5.337414	ADCY1	0.021866967
16384	0.01	0.4591067	HIST1H2AJ	0.021781428
16385	0.1475395	6.7777152	EPB49	0.021768319
16386	0.01	0.4598686	SYTL5	0.021745344
16387	0.01	0.4612079	HIST1H2BI	0.021682196
16388	0.01	0.4612079	KLK1	0.021682196
16389	1.3810437	63.709956	MGST1	0.021677048
16390	0.01	0.4633284	HIST1H2BE	0.021582964
16391	0.01	0.4634468	CDH22	0.021577451
16392	0.01	0.464396	ZNF32-AS1	0.021533348
16393	0.01	0.4658271	ACTR3C	0.021467194
16394	0.01	0.4661144	MSX2P1	0.021453963
16395	0.01	0.4665459	RFPL4AL1	0.021434116
16396	0.01	0.4687159	HAMP	0.021334884
16397	0.01	0.4689886	CBWD6	0.02132248
16398	0.01	0.4698085	SCGB1D1	0.021285268
16399	0.01	0.4709062	DIP2A-IT1	0.021235652
16400	0.01	0.4718511	CTTNBP2	0.021193124
16401	0.01	0.473117	HTRA4	0.02113642
16402	0.193201	9.1556037	EFNB3	0.021101942
16403	0.0453733	2.1501948	TNFRSF11A	0.021101942
16404	8.3483479	398.11182	EEF1A2	0.020969857
16405	0.01	0.4791153	RASGRP1	0.020871802
16406	0.3219354	15.463764	THBS2	0.020818695
16407	0.01	0.4810211	NCOR1P1	0.020789108
16408	0.0648613	3.1364433	RASL10B	0.020679903
16409	0.01	0.4839084	NBPF4	0.020665068
16410	0.01	0.4854775	KCNJ2	0.020598277
16411	0.01	0.4856575	KCNJ4	0.020590644
16412	0.01	0.4856575	IGFBP1	0.020590644
16413	0.01	0.4878617	PLD5	0.020497614
16414	0.01	0.4886008	C1QTNF9B-AS1	0.020466604
16415	0.01	0.4891938	HIST4H4	0.020441796
16416	0.01	0.4893918	COMP	0.020433527
16417	0.01	0.4919801	FAM124A	0.020326025
16418	0.01	0.4930834	FLJ33534	0.020280544
16419	0.0635558	3.134779	SLC47A1	0.020274415
16420	0.01	0.4947098	PGR	0.020213873
16421	0.01	0.4954064	CLSTN2	0.020185447
16422	0.01	0.497158	CES1	0.02011433
16423	0.01	0.4981303	TFCP2L1	0.020075069
16424	0.01	0.5004292	CCDC78	0.019982848

16425	0.01	0.5013628	CLVS2	0.019945636
16426	0.01	0.5026131	ENTPD3	0.01989602
16427	0.01	0.5042899	CCDC169	0.019829865
16428	0.139491	7.1499571	CTSH	0.019509343
16429	0.0555584	2.8477835	ELFN1	0.019509343
16430	0.01	0.5158446	MMP13	0.019385684
16431	0.01	0.5168777	GRID1	0.019346936
16432	0.01	0.5207955	PROM1	0.019201396
16433	0.01	0.5216942	OR7E14P	0.019168319
16434	0.0487256	2.5446771	HPCAL4	0.019148058
16435	0.01	0.5251206	LPHN3	0.019043245
16436	0.01	0.5273821	OLIG1	0.018961585
16437	0.4219562	22.308558	KCNC4	0.018914546
16438	0.01	0.5301101	TMEM163	0.018864007
16439	0.0196919	1.047447	RIMKLA	0.018799913
16440	0.1362534	7.2475525	ADAM23	0.018799912
16441	0.01	0.5331954	TNRC18P1	0.018754852
16442	0.01	0.5353196	LINC00634	0.018680428
16443	0.01	0.5360315	RASL10A	0.01865562
16444	0.01	0.538898	ZDHHC11	0.018556388
16445	0.01	0.5413587	LRRC4	0.018472041
16446	0.01	0.5496759	KRTAP4-9	0.018192537
16447	0.01	0.5508653	TNK1	0.018153258
16448	0.068395	3.7703404	HOXB13	0.018140266
16449	0.0769854	4.2438963	STK33	0.018140266
16450	0.01	0.5552282	NBL1	0.018010612
16451	0.01	0.5559941	PLAC8L1	0.017985804
16452	0.01	0.556762	FAM25A	0.017960996
16453	0.3596505	20.24348	CHD7	0.01776624
16454	0.01	0.5635075	ZNF295-AS1	0.017745993
16455	0.01	0.5642964	GAP43	0.017721185
16456	0.01	0.5653516	FXYD7	0.017688108
16457	0.01	0.565616	PYY	0.017679838
16458	0.01	0.5661456	SMKR1	0.0176633
16459	0.01	0.5664639	TMEM145	0.017653376
16460	0.01	0.5669419	IL17B	0.017638492
16461	0.01	0.567474	LOC100506343	0.017621953
16462	0.01	0.5685412	LOC100133669	0.017588876
16463	0.01	0.5698808	RTN4RL1	0.017547529
16464	0.01	0.5698808	PTCHD1	0.017547529
16465	0.01	0.570654	SPEF2	0.017523755
16466	0.01	0.5748655	CA3	0.017395373
16467	0.01	0.5749	ANO5	0.017394329

16468	0.01	0.5753031	CCDC144CP	0.017382142
16469	0.01	0.5833512	PLAC9	0.017142331
16470	0.1450913	8.4660386	GALNT16	0.017138042
16471	0.01	0.5849031	ATP8A1	0.01709685
16472	0.01	0.58899	DOK6	0.016978217
16473	0.042574	2.5116279	LHFPL4	0.01695074
16474	0.0599538	3.5369444	CPNE8	0.01695074
16475	0.01	0.5904722	OR7E2P	0.016935598
16476	0.01	0.5907607	RUNDC3A	0.016927329
16477	0.01	0.5935813	DOCK8	0.016846891
16478	0.821117	48.759014	STMN3	0.016840312
16479	0.01	0.596884	FUT8-AS1	0.016753673
16480	0.01	0.6011227	FLJ35282	0.016635539
16481	0.01	0.6016354	NBPF22P	0.016621363
16482	0.01	0.6026634	NPNT	0.016593011
16483	0.069839	4.2214278	DNAJC6	0.016543923
16484	0.01	0.6060577	KIRREL2	0.01650008
16485	0.01	0.6070718	ZNF385B	0.016472515
16486	0.087306	5.3194446	MMP9	0.016412622
16487	0.0592044	3.6072484	BTNL9	0.016412622
16488	0.01	0.6118207	MYO18B	0.016344659
16489	0.01	0.6119874	ANKRD34B	0.016340206
16490	0.2871835	17.682884	CCND2	0.016240762
16491	0.01	0.6182449	KHDC1L	0.016174819
16492	1.0654371	65.946126	G0S2	0.016156175
16493	0.059154	3.6613858	CHSY3	0.016156174
16494	0.0711261	4.4024103	NKAIN1	0.016156174
16495	0.01	0.6217871	FLJ44635	0.016082675
16496	0.01	0.6232154	SLC24A5	0.016045818
16497	0.01	0.6255365	ISLR2	0.015986278
16498	0.0565995	3.5580147	RADIL	0.015907618
16499	0.01	0.633002	VAV3	0.015797738
16500	0.01	0.6356153	CREG2	0.015732786
16501	0.2604994	16.57731	TIE1	0.015714212
16502	0.0526927	3.3633775	SLC16A6	0.015666594
16503	0.01	0.6391581	LRRC34	0.015645582
16504	0.01	0.639327	SEC14L6	0.015641447
16505	0.01	0.6418721	ARHGDIG	0.015579427
16506	0.01	0.6418721	TJP3	0.015579427
16507	0.01	0.6423836	TREX2	0.015567023
16508	0.01	0.6444376	RAI2	0.015517407
16509	0.01	0.6480638	OVOL2	0.015430579
16510	0.01	0.6484429	MAT1A	0.015421558

16511	0.01	0.6485852	NME5	0.015418175
16512	0.01	0.6501543	RLN2	0.015380963
16513	0.01	0.6514523	PPP1R9A	0.015350318
16514	0.01	0.6533156	LCE3D	0.015306539
16515	0.01	0.6554402	LOC100302640	0.015256923
16516	0.0759681	4.9959915	SORL1	0.015205811
16517	0.1916493	12.640761	NPTX1	0.015161219
16518	0.01	0.6669038	EMBP1	0.014994667
16519	0.01	0.6704852	POU3F3	0.014914573
16520	0.1490697	9.9956968	WNK4	0.014913392
16521	0.01	0.6735725	KCND3	0.014846213
16522	0.01	0.6736225	RPRM	0.01484511
16523	0.01	0.6800433	SLC35D3	0.014704945
16524	0.01	0.6814805	FAM150A	0.014673935
16525	0.3566449	24.374298	LOC100128881	0.014632007
16526	0.01	0.6855369	LOC100507424	0.014587107
16527	0.0606871	4.1671221	NTNG1	0.014563312
16528	0.01	0.6887426	BMPR1B	0.014519211
16529	0.01	0.6954722	HCN4	0.01437872
16530	0.1648078	11.476034	SLC29A2	0.014361044
16531	0.0405525	2.8237877	ERG	0.014361044
16532	0.5072604	35.485503	BGN	0.014294864
16533	0.01	0.699576	PRSS16	0.014294372
16534	0.01	0.7001228	ZNF891	0.014283209
16535	0.01	0.701036	EEF1DP3	0.014264603
16536	0.01	0.7047128	FAM19A3	0.014190179
16537	0.01	0.7071854	CHST9	0.014140563
16538	0.01	0.7198137	WIF1	0.013892483
16539	0.3868461	27.952671	MLPH	0.013839323
16540	0.01	0.723366	MRAP2	0.013824261
16541	0.01	0.7249923	ALOX12B	0.013793251
16542	0.0350487	2.5422281	BCL11A	0.013786602
16543	0.01	0.7254272	TIMP4	0.013784981
16544	0.01	0.7262985	MYT1	0.013768443
16545	0.01	0.7262985	SMIM1	0.013768443
16546	0.01	0.7262985	MLLT10P1	0.013768443
16547	0.01	0.7264855	GABRD	0.013764899
16548	0.01	0.7276096	RASGEF1C	0.013743635
16549	0.01	0.729695	RAPGEF5	0.013704355
16550	0.01	0.7336635	CNKSR2	0.013630227
16551	0.01	0.7336794	ADCYAP1R1	0.013629931
16552	0.372641	27.389605	SERPIND1	0.0136052
16553	0.01	0.7355761	NBEAP1	0.013594787

16554	0.01	0.7372884	RBFOX3	0.013563213
16555	0.0953119	7.0516397	JPH1	0.013516277
16556	0.01	0.7446349	FCER2	0.0134294
16557	0.0826654	6.1559636	OLIG2	0.013428509
16558	0.01	0.7471246	CAMK2B	0.013384648
16559	0.01	0.7508261	FGF12	0.013318663
16560	0.01	0.7532934	SLC27A2	0.013275039
16561	0.0789691	5.9570792	NRARP	0.013256348
16562	0.01	0.7554672	ANO2	0.013236843
16563	0.2906548	21.995982	GALNT14	0.013213996
16564	0.01	0.7568451	NTRK1	0.013212743
16565	0.01	0.7589668	ZNF354C	0.013175807
16566	0.01	0.7612761	ONECUT1	0.013135839
16567	0.01	0.7673141	SHH	0.013032472
16568	0.01	0.7685333	ZNF736	0.013011799
16569	0.01	0.7690569	FLVCR2	0.013002939
16570	0.01	0.771229	SMCP	0.012966317
16571	0.3088163	23.843275	BMP7	0.012951923
16572	0.01	0.7733993	C3orf80	0.012929932
16573	0.040154	3.1067106	TRPA1	0.01292494
16574	0.5792898	44.959596	IGFBP2	0.012884675
16575	0.01	0.7771768	SLC9A2	0.012867085
16576	0.01	0.777748	HOXD10	0.012857635
16577	0.01	0.7903837	C19orf81	0.012652083
16578	0.01	0.8010646	ABLIM2	0.012483388
16579	0.0284272	2.2818812	ASTN1	0.012457773
16580	0.01	0.8072677	SLC6A1	0.012387464
16581	0.5357314	43.262841	GAL	0.012383176
16582	0.01	0.8098356	HOXD1	0.012348184
16583	0.01	0.8174015	TSGA10IP	0.01223389
16584	0.01	0.8186346	MTRNR2L4	0.012215462
16585	0.01	0.8201337	MPZL3	0.012193134
16586	0.0979091	8.0486573	SLC7A8	0.012164649
16587	0.01	0.8245139	CPLX1	0.012128358
16588	0.01	0.8260158	SYT3	0.012106306
16589	0.01	0.8265804	LBX2	0.012098037
16590	0.01	0.8272589	IGSF9B	0.012088114
16591	0.01	0.8380368	PDE2A	0.01193265
16592	0.01	0.8420153	TTPA	0.011876269
16593	0.2549007	21.482472	LPHN2	0.011865518
16594	0.01	0.8472847	SLCO5A1	0.011802408
16595	0.0698156	5.9417791	SETD6	0.011749945
16596	0.01	0.8531126	OR2W3	0.011721782

16597	0.01	0.8540163	SNORA58	0.011709378
16598	0.01	0.8564356	LINC00659	0.011676301
16599	0.01	0.8625443	NPPC	0.011593608
16600	0.01	0.8635709	CAMSAP3	0.011579825
16601	0.01	0.8656679	SYT9	0.011551775
16602	0.01	0.8717467	LOC100499227	0.011471222
16603	0.01	0.8738771	SOX21	0.011443256
16604	0.01	0.8746652	PROK2	0.011432946
16605	0.01	0.8750268	RSPH1	0.011428221
16606	0.01	0.8774253	ZNF605	0.011396981
16607	0.0534495	4.7039893	GDAP1	0.011362584
16608	0.01	0.8927922	CRYBA2	0.011200814
16609	0.01	0.8940652	EPS8L2	0.011184866
16610	0.01	0.8987641	FBXL21	0.01112639
16611	0.01	0.9007725	LOC100506321	0.011101582
16612	0.2158015	19.514056	LAMA1	0.011058772
16613	0.01	0.9126006	SLITRK2	0.010957696
16614	0.01	0.9167677	CHRFAM7A	0.010907889
16615	0.1252777	11.510096	RTN1	0.01088416
16616	0.01	0.9207603	UBE2QL1	0.01086059
16617	0.01	0.9281978	HS6ST3	0.010773565
16618	0.0556474	5.1665136	PDZD4	0.010770783
16619	0.0837619	7.7767658	LRRC33	0.010770783
16620	0.1136311	10.549942	HOXB8	0.010770783
16621	0.01	0.9322287	FLJ42102	0.010726981
16622	0.01	0.9349857	FAM198A	0.010695351
16623	0.0201372	1.889085	MED12L	0.010659744
16624	0.01	0.9439017	FAM184A	0.010594324
16625	0.01	0.9498014	TGFB3	0.010528517
16626	0.01	0.9518198	EFCAB3	0.01050619
16627	0.01	0.9541981	FEV	0.010480004
16628	0.01	0.9552031	CLPSL2	0.010468978
16629	0.01	0.9555265	NAT16	0.010465434
16630	0.01	0.9597517	SCARNA2	0.010419362
16631	0.01	0.9666563	SPATA17	0.010344938
16632	0.01	0.9674297	SYT6	0.010336669
16633	0.01	0.9719004	GRAMD2	0.01028912
16634	0.01	0.972938	FAM124B	0.010278147
16635	0.01	0.9729896	IRF5	0.010277602
16636	0.01	0.9732337	WNK2	0.010275025
16637	0.01	0.9736611	LOC387895	0.010270514
16638	0.01	0.977956	CYP26B1	0.010225409
16639	0.01	0.9797465	NEURL2	0.010206722

16640	0.01	0.9827915	CHGB	0.010175099
16641	0.01	0.9831602	SNORA73B	0.010171282
16642	0.01	0.9856909	SCUBE1	0.010145168
16643	0.1496048	14.75799	REEP6	0.010137208
16644	0.01	0.9889492	GPR4	0.010111743
16645	0.01	0.9912189	TOMM20L	0.010088589
16646	0.0393504	3.9198316	EDA	0.010038788
16647	0.01	0.9993695	CACNB4	0.010006309
16648	0.0636918	6.467764	DNER	0.009847573
16649	0.01	1.0179184	ANP32D	0.00982397
16650	0.01	1.0179184	ERC2	0.00982397
16651	0.01	1.018314	PDX1	0.009820153
16652	0.01	1.0184328	RASL11B	0.009819008
16653	0.01	1.0225088	OPRD1	0.009779867
16654	0.01	1.0226136	B3GALT1	0.009778865
16655	0.01	1.0243855	IL37	0.00976195
16656	0.01	1.0283053	NKX2-1	0.009724738
16657	0.01	1.0378365	RIMS2	0.009635429
16658	0.01	1.0442894	GNAS-AS1	0.00957589
16659	0.1294809	13.649404	SOX7	0.009486194
16660	0.01	1.0658268	RAET1K	0.009382387
16661	0.01	1.067018	LOC100129917	0.009371913
16662	0.01	1.0682748	C17orf98	0.009360887
16663	0.01	1.0704362	CLDN3	0.009341986
16664	0.01	1.0710272	CNTFR	0.009336831
16665	1.695927	181.9623	ESM1	0.009320211
16666	0.01	1.077796	SCARNA16	0.009278194
16667	0.01	1.0873487	KIT	0.009196682
16668	0.0566611	6.2469969	NHSL2	0.009070133
16669	0.01	1.1080146	OXER1	0.009025152
16670	0.0205219	2.2824227	TEX15	0.008991262
16671	0.01	1.1123534	SP6	0.008989948
16672	0.01	1.1144036	CCM2L	0.00897341
16673	0.1652216	18.482321	VAT1L	0.008939439
16674	0.01	1.1247573	VWA1	0.008890807
16675	0.01	1.1250677	LOC389895	0.008888354
16676	0.01	1.1272251	C20orf201	0.008871343
16677	0.01	1.1280663	PGM5-AS1	0.008864727
16678	0.01	1.1379321	JAKMIP1	0.008787871
16679	0.01	1.142163	FAM19A4	0.008755317
16680	0.01	1.1461865	PGM5	0.008724584
16681	0.01	1.151702	FAM162B	0.008682802
16682	0.01	1.1523006	RTN4R	0.008678291

16683	0.01	1.1579295	GLB1L3	0.008636104
16684	0.01	1.1650165	FAM209B	0.00858357
16685	0.01	1.1709388	PAK3	0.008540156
16686	0.01	1.1740651	RAB3C	0.008517415
16687	0.01	1.1763493	FLJ42351	0.008500876
16688	0.01	1.1774947	POU3F2	0.008492607
16689	0.01	1.180615	OSTCP1	0.008470162
16690	0.01	1.1932327	GDF6	0.008380595
16691	0.01	1.1973139	GPR50	0.008352028
16692	0.01	1.2025528	FABP4	0.008315643
16693	0.01	1.2049745	HTR1F	0.008298931
16694	0.01	1.2050693	LINC00853	0.008298278
16695	0.0494423	5.977101	PKIA	0.008271961
16696	0.01	1.2228099	GFI1	0.008177886
16697	0.01	1.2235245	ZNF385D	0.00817311
16698	0.01	1.2236209	SDHAP3	0.008172466
16699	0.4704278	57.780083	MT1L	0.008141694
16700	0.01	1.230682	ABI3	0.008125576
16701	0.01	1.2351526	FSHR	0.008096165
16702	0.01	1.235858	BMP4	0.008091544
16703	0.01	1.2370319	CEBPA-AS1	0.008083866
16704	0.01	1.2405489	SYNDIG1	0.008060948
16705	0.01	1.2537969	SERPINI1	0.007975774
16706	0.01	1.2557498	ESPN	0.00796337
16707	0.01	1.2557498	VAX1	0.00796337
16708	0.01	1.2573925	ACE	0.007952966
16709	0.01	1.2574574	PPP1R16B	0.007952556
16710	0.01	1.2669836	CCDC68	0.007892762
16711	0.01	1.2675965	HIST1H3I	0.007888946
16712	0.01	1.2682101	IGSF5	0.007885129
16713	0.01	1.2725989	LOC729041	0.007857936
16714	0.01	1.2804079	B4GALNT4	0.007810011
16715	0.2016444	25.839468	GYPC	0.007803737
16716	0.01	1.2886691	LOC729737	0.007759944
16717	0.01	1.3127302	LMO2	0.007617711
16718	0.01	1.3173062	ZNF467	0.00759125
16719	0.01	1.3354615	BMP8B	0.007488048
16720	0.01	1.3510215	RAB26	0.007401807
16721	0.01	1.3526701	SNORA57	0.007392785
16722	0.01	1.3595133	CKMT1A	0.007355573
16723	0.0246073	3.355561	SLFN13	0.007333299
16724	0.01	1.377593	FOXO4	0.007259038
16725	0.01	1.3804647	SNORD15B	0.007243937

16726	0.0718371	10.004443	GABBR2	0.007180522
16727	0.0934946	13.020588	FXYD6	0.007180522
16728	0.01	1.3974812	LINC00471	0.007155731
16729	0.01	1.3980844	RORB	0.007152644
16730	0.01	1.4001634	COBL	0.007142023
16731	0.01	1.4012597	RHOV	0.007136436
16732	0.01	1.4032922	CHN2	0.007126099
16733	0.2114658	29.961197	SPTBN2	0.007057988
16734	0.0600921	8.5431181	FAM81A	0.007033981
16735	0.01	1.4294174	RNU4-2	0.006995857
16736	0.1458359	20.874095	MANEAL	0.006986454
16737	0.01	1.4352339	LINC00240	0.006967505
16738	0.01	1.443426	LOC283174	0.006927962
16739	0.0895187	12.9517	KIAA1549L	0.006911732
16740	0.01	1.448595	TMEM74	0.006903241
16741	0.01	1.4504814	SLC16A10	0.006894263
16742	0.01	1.4525971	RPRML	0.006884221
16743	0.01	1.4711522	SCARNA14	0.006797393
16744	0.01	1.4772468	TRIM71	0.00676935
16745	0.01	1.4819695	SNORA39	0.006747777
16746	0.01	1.4819695	SNORA71B	0.006747777
16747	0.01	1.4819695	SNORA65	0.006747777
16748	0.01	1.4837878	CCL7	0.006739508
16749	0.01	1.492947	SNORA48	0.006698161
16750	0.01	1.4969389	THSD1	0.0066803
16751	0.01	1.5040884	SNORA22	0.006648545
16752	0.01	1.5040884	SCARNA18	0.006648545
16753	0.01	1.5040884	SNORA14A	0.006648545
16754	0.01	1.509137	GRM8	0.006626304
16755	0.01	1.5130637	RPS6KL1	0.006609107
16756	0.1611752	24.394616	VGF	0.006606998
16757	0.01	1.5247774	EMX2	0.006558334
16758	0.01	1.5268776	SNORA51	0.006549313
16759	0.01	1.5268776	SNORA72	0.006549313
16760	0.01	1.5268776	ALX1	0.006549313
16761	0.0841338	12.937468	TUSC1	0.006503114
16762	0.01	1.5381283	KCNK5	0.006501408
16763	0.01	1.5385332	SNORA11	0.006499697
16764	0.01	1.5385332	SNORA24	0.006499697
16765	0.01	1.5511297	LAMC3	0.006446914
16766	0.01	1.5557534	CEBPA	0.006427754
16767	0.144903	22.5904	NUP210	0.006414362
16768	0.01	1.5593644	VSTM1	0.006412869

16769	0.01	1.5675944	IRAK1BP1	0.006379201
16770	0.01	1.572545	ST3GAL6-AS1	0.006359119
16771	0.01	1.5779547	RIBC2	0.006337318
16772	0.01	1.5845649	SCN4B	0.006310881
16773	0.0211638	3.3567429	UNC13A	0.006304848
16774	0.01	1.5901211	ACTL10	0.006288829
16775	0.01	1.5943757	ZNF718	0.006272047
16776	0.0608288	9.7067702	AMPH	0.006266637
16777	0.01	1.5964186	PARVG	0.006264021
16778	0.0319239	5.1251444	MOB3B	0.006228887
16779	0.01	1.6123828	SCARNA22	0.006202001
16780	0.01	1.6207179	FAM201A	0.006170105
16781	0.01	1.631023	GATA5	0.006131121
16782	0.0904119	14.777261	MYEOV	0.006118315
16783	0.042306	6.9146541	RRAGD	0.006118315
16784	0.01	1.6372352	ZNF704	0.006107858
16785	0.01	1.6489125	RNF175	0.006064603
16786	0.01	1.6634673	ANKRD18A	0.00601154
16787	0.01	1.6851295	LGI2	0.005934262
16788	0.01	1.6920066	SAMD15	0.005910142
16789	0.01	1.6936794	CACNA2D3	0.005904305
16790	0.01	1.6980862	ONECUT2	0.005888983
16791	0.01	1.7123861	WT1-AS	0.005839804
16792	0.01	1.727553	TCF24	0.005788534
16793	0.01	1.7285407	LOC100288842	0.005785227
16794	0.01	1.7374814	GABRA2	0.005755457
16795	0.0518665	9.0791838	JPH3	0.00571268
16796	0.0893396	15.667623	AK4	0.005702179
16797	0.134625	23.696194	NPDC1	0.005681292
16798	0.01	1.7623421	GPR162	0.005674267
16799	0.01	1.7691573	FAM78B	0.005652409
16800	0.01	1.780458	LXN	0.005616532
16801	0.01	1.7964498	FOXL2	0.005566535
16802	0.01	1.8025792	PAX2	0.005547606
16803	0.1391185	25.159845	BARX1	0.005529386
16804	0.01	1.812606	TFR2	0.005516919
16805	0.01	1.8225879	PIP5K1B	0.005486704
16806	0.01	1.8236323	BEGAIN	0.005483561
16807	0.01	1.8310642	C14orf37	0.005461305
16808	0.01	1.8661838	MUM1L1	0.005358529
16809	0.01	1.8705137	FBXO2	0.005346125
16810	0.01	1.8748637	LCE1D	0.005333721
16811	0.01	1.8757361	MAGEL2	0.00533124

16812	0.01	1.8836247	TMEM59L	0.005308913
16813	0.0452944	8.5420192	PALD1	0.005302539
16814	0.01	1.886143	C2orf81	0.005301825
16815	0.01	1.9013948	MIR1248	0.005259297
16816	0.01	1.9161852	CELF2	0.005218702
16817	0.01	1.9174742	C1orf162	0.005215194
16818	0.01	1.9324949	DRAXIN	0.005174658
16819	0.01	1.9353754	UNC5A	0.005166956
16820	0.01	1.9426299	DICER1-AS1	0.005147661
16821	0.01	1.9508564	RELN	0.005125954
16822	0.01	1.9649512	LMO1	0.005089185
16823	0.01	1.9680838	TAL1	0.005081084
16824	0.01	1.9701647	FOXA3	0.005075718
16825	0.01	1.9759593	DLL1	0.005060833
16826	0.3117161	61.633379	CDCP1	0.005057585
16827	0.01	2.0154785	MIR658	0.004961601
16828	0.01	2.0317323	LOC645249	0.004921908
16829	0.01	2.0330617	PRIMA1	0.00491869
16830	0.01	2.0566107	SNORD116-26	0.004862369
16831	0.01	2.0566107	SNORD46	0.004862369
16832	0.01	2.0566107	SNORD116-4	0.004862369
16833	0.01	2.0653921	HLA-DRB5	0.004841696
16834	0.01	2.0778129	MIR590	0.004812753
16835	0.01	2.0795995	VAX2	0.004808618
16836	0.01	2.084259	FAM159B	0.004797868
16837	0.0299511	6.3436301	KIF5C	0.004721439
16838	0.0205786	4.3983484	PTPRD	0.004678711
16839	0.01	2.1430897	C12orf56	0.00466616
16840	0.01	2.144126	SNORD116-22	0.004663905
16841	0.01	2.1581008	PLCXD2	0.004633704
16842	0.01	2.1671812	SNORD19B	0.004614289
16843	0.01	2.1718518	MT1M	0.004604366
16844	0.0771279	16.783222	EYA2	0.004595534
16845	0.01	2.2042143	HOXD11	0.004536764
16846	0.01	2.2148115	SNORD14B	0.004515057
16847	0.01	2.2155969	TPBGL	0.004513456
16848	0.01	2.2196098	ELMO1	0.004505296
16849	0.01	2.226102	SEMA3F	0.004492157
16850	0.01	2.2266087	PARM1	0.004491135
16851	0.01	2.2485054	LRRC4B	0.004447399
16852	0.01	2.2555315	ZFP92	0.004433545
16853	0.01	2.2645826	MIR4497	0.004415825
16854	0.01	2.2645826	MIR4721	0.004415825

16855	0.01	2.2916185	WBSCR17	0.004363728
16856	0.01	2.2940405	RAB39A	0.004359121
16857	0.01	2.3027774	EFCAB4A	0.004342582
16858	0.01	2.303725	DLGAP3	0.004340796
16859	0.01	2.3275246	GRIN2D	0.00429641
16860	0.01	2.3435796	MIR937	0.004266977
16861	0.01	2.3935507	NELL2	0.004177894
16862	0.01	2.3952399	TTC9	0.004174947
16863	0.01	2.3983217	DMC1	0.004169582
16864	0.01	2.4035871	RNF128	0.004160448
16865	0.01	2.4250409	BARHL2	0.004123642
16866	0.01	2.4282873	SNORD60	0.004118129
16867	0.01	2.4366752	PI3	0.004103953
16868	0.01	2.4493662	NPY	0.004082689
16869	0.01	2.4508085	SLC35F1	0.004080286
16870	0.01	2.4579006	MIR4712	0.004068513
16871	0.01	2.4729797	PAQR9	0.004043705
16872	0.01	2.4786822	HAAO	0.004034402
16873	0.01	2.5096954	TESC	0.003984547
16874	0.01	2.5130654	KCNJ8	0.003979204
16875	0.01	2.5193481	CECR2	0.003969281
16876	0.01	2.5193481	MIR4677	0.003969281
16877	0.0398851	10.067749	DPYSL5	0.003961667
16878	0.01	2.5289274	FAM174B	0.003954246
16879	0.01	2.5357837	TLX1	0.003943554
16880	0.01	2.6164426	SULT4A1	0.003821983
16881	0.01	2.6175045	MIR4784	0.003820433
16882	0.01	2.6209083	DBNDD2	0.003815471
16883	0.01	2.6446956	ZNF215	0.003781153
16884	0.01	2.6763248	ADORA1	0.003736467
16885	0.01	2.6808888	LINGO1	0.003730106
16886	0.01	2.6944899	HIST1H4H	0.003711278
16887	0.042969	11.594196	WASF3	0.003706076
16888	0.01	2.7032668	GJA3	0.003699228
16889	0.01	2.7694654	NDNF	0.003610805
16890	0.01	2.7709773	ICA1	0.003608835
16891	0.01	2.7871785	MCM3AP-AS1	0.003587858
16892	0.01	2.8011582	SLITRK5	0.003569952
16893	0.01	2.8776994	STON2	0.003474998
16894	0.01	2.9423043	SNORA5C	0.003398697
16895	0.01	3.0056134	GJB2	0.003327108
16896	0.0275188	8.330183	CCDC88C	0.003303499
16897	0.01	3.0307947	SNORA9	0.003299465

16898	0.01	3.0537553	SNORA41	0.003274657
16899	0.01	3.1007361	SNORA1	0.003225041
16900	0.01	3.1007361	SNORA3	0.003225041
16901	0.01	3.1450899	TMEM121	0.003179559
16902	0.01	3.1818937	GHR	0.003142783
16903	0.01	3.2013816	C7orf29	0.003123651
16904	0.01	3.2070037	RTP3	0.003118175
16905	0.01	3.2090767	TENM4	0.003116161
16906	0.01	3.2736521	CHMP4C	0.003054692
16907	0.01	3.2844834	FAM155B	0.003044619
16908	0.0139912	4.6141535	KIAA1244	0.003032244
16909	0.01	3.3251238	FAM95C	0.003007407
16910	0.01	3.3313694	SNORA32	0.003001769
16911	0.01	3.3756339	TSPYL5	0.002962407
16912	0.01	3.4371835	EPHB1	0.002909359
16913	0.01	3.4402422	GRB14	0.002906772
16914	0.01	3.4452623	MT1A	0.002902537
16915	0.01	3.4799628	C2orf70	0.002873594
16916	0.01	3.5067849	C10orf35	0.002851615
16917	0.01	3.5405713	PDE3B	0.002824403
16918	0.01	3.6703092	RTN4RL2	0.002724566
16919	0.01	3.718595	ZIC5	0.002689188
16920	0.01	3.7587701	P2RY1	0.002660445
16921	0.01	3.7672495	BCHE	0.002654457
16922	0.01	3.7966494	IRX1	0.002633901
16923	0.01	3.8842198	TMEM204	0.00257452
16924	0.01	3.8949094	ATP1A3	0.002567454
16925	0.01	3.9364814	SH3GL3	0.00254034
16926	0.01	3.9547906	IL13RA2	0.002528579
16927	0.1155849	46.614228	HPDL	0.002479605
16928	0.01	4.0580103	SNORA6	0.002464262
16929	0.0210632	8.5557	CNTNAP2	0.002461893
16930	0.01	4.0656751	EBF3	0.002459616
16931	0.01	4.0917127	KCNQ2	0.002443964
16932	0.0225712	9.3646731	KIF1A	0.002410245
16933	0.01	4.1593544	IRX4	0.002404219
16934	0.01	4.1705424	HAND2	0.00239777
16935	0.01	4.2078588	QRFPR	0.002376506
16936	0.01	4.2441515	SLC13A3	0.002356184
16937	0.01	4.2843731	PPP1R14C	0.002334064
16938	0.01	4.2894529	ST3GAL6	0.0023313
16939	0.01	4.3074341	DLX4	0.002321568
16940	0.01	4.3737358	NRG2	0.002286375

16941	0.01	4.4883402	CACNG6	0.002227995
16942	0.01	4.5027996	GLB1L2	0.002220841
16943	0.01	4.5299861	LIN28B	0.002207512
16944	0.01	4.5530387	RIPPLY2	0.002196335
16945	0.3430444	156.42767	FOXR1	0.002192991
16946	0.01	4.5719738	ALDH1A2	0.002187239
16947	0.01	4.5726388	B4GALNT3	0.002186921
16948	0.01	4.6332838	SNORD14D	0.002158296
16949	0.01	4.7943678	ENHO	0.002085781
16950	0.01	4.8356009	RIMS4	0.002067995
16951	0.01	4.8833179	MERTK	0.002047788
16952	0.01	4.9016171	AGTR1	0.002040143
16953	0.01	4.9278202	TCF15	0.002029295
16954	0.01	4.950298	WFDC2	0.00202008
16955	0.01	5.0828179	SLAIN1	0.001967413
16956	0.01	5.1868931	MNX1	0.001927936
16957	0.01	5.2391748	NOVA2	0.001908698
16958	0.01	5.3382302	HES7	0.00187328
16959	0.01	5.3518355	BEND4	0.001868518
16960	0.01	5.3746093	SNORD24	0.0018606
16961	0.01	5.3746093	SNORD28	0.0018606
16962	0.1501437	81.098323	PRAME	0.001851379
16963	0.01	5.4542588	3-Sep	0.00183343
16964	0.01	5.4569102	PRKCQ-AS1	0.001832539
16965	0.01	5.4588431	PRKCQ	0.00183189
16966	0.01	5.4777193	ANKRD18B	0.001825577
16967	0.01	5.577035	GATM	0.001793067
16968	0.01	5.6077142	B3GAT1	0.001783258
16969	0.01	5.621151	RSPO4	0.001778995
16970	0.01	5.7296211	LCE1F	0.001745316
16971	0.01	5.755547	SYN2	0.001737454
16972	0.01	5.8128909	NOVA1	0.001720314
16973	0.01	5.9824235	HID1	0.001671563
16974	0.01	6.0450615	SNCB	0.001654243
16975	0.01	6.078171	LRFN5	0.001645232
16976	0.01	6.1505212	ZDHHC22	0.001625878
16977	0.01	6.3197653	EMILIN3	0.001582337
16978	0.01	6.3983444	BEX5	0.001562904
16979	0.01	6.6081261	SNORA26	0.001513288
16980	0.01	6.6081261	MSI1	0.001513288
16981	0.01	6.6364125	WT1	0.001506838
16982	0.01	6.6596234	ZNF296	0.001501586
16983	0.01	6.7010793	CRABP1	0.001492297

16984	0.01	6.7182616	CDX2	0.00148848
16985	0.01	6.8395751	LRFN1	0.001462079
16986	0.01	6.8729519	LHX1	0.001454979
16987	0.01	6.9696251	TNFRSF10A	0.001434797
16988	0.01	7.0913401	FAM189A1	0.001410171
16989	0.01	7.2482058	KIF26A	0.001379652
16990	0.01	7.3686695	GATA4	0.001357097
16991	0.01	7.3902436	EMB	0.001353135
16992	0.01	7.5131288	PRR9	0.001331003
16993	0.01	7.5769867	SNORA33	0.001319786
16994	0.01	7.8470541	PPP2R2C	0.001274364
16995	0.01	7.8617955	GPR27	0.001271974
16996	0.01	7.9538039	ISM1	0.00125726
16997	0.01	7.9793894	SHANK1	0.001253229
16998	0.01	7.984021	NKD2	0.001252502
16999	0.01	8.0106458	OGDHL	0.001248339
17000	0.01	8.0974094	CERS4	0.001234963
17001	0.0548131	47.603884	IL10RA	0.001151442
17002	0.01	8.9430385	EGFL7	0.001118188
17003	0.01	8.9442068	APBB1IP	0.001118042
17004	0.01	9.2247974	MATK	0.001084035
17005	0.01	9.3443446	CITED1	0.001070166
17006	0.1178064	110.51521	CSF3	0.001065974
17007	0.01	9.4926638	ADD2	0.001053445
17008	0.01	9.848283	NAT8L	0.001015405
17009	0.01	9.9539327	SLC17A9	0.001004628
17010	0.01	10.227184	PLBD1	0.000977786
17011	0.01	10.25254	PRKCZ	0.000975368
17012	0.01	10.404031	CA2	0.000961166
17013	0.01	10.629298	RAB38	0.000940796
17014	0.0468735	52.540251	EPB41L3	0.000892144
17015	0.01	11.274021	CYTL1	0.000886995
17016	0.01	11.401198	ESX1	0.000877101
17017	0.0505211	57.654899	AEBP1	0.000876267
17018	0.01	12.52066	TUBB4A	0.00079868
17019	0.01	13.018451	GYLTL1B	0.000768141
17020	0.01	14.031299	COL9A3	0.000712692
17021	0.01	14.05952	DUSP9	0.000711262
17022	1.7024467	2450.8257	MMP1	0.000694642
17023	0.01	14.428231	MB21D1	0.000693086
17024	0.01	14.542347	TMEM88	0.000687647
17025	0.01	14.744588	MED12	0.000678215
17026	0.01	15.196923	ZFP57	0.000658028

17027	0.01	15.459905	KRT75	0.000646834
17028	0.01	16.490278	LCE1E	0.000606418
17029	0.01	17.315525	ABCA3	0.000577516
17030	0.01	18.085051	ERVMER34-1	0.000552943
17031	0.01	20.845524	MDFI	0.000479719
17032	0.01	20.924419	MST4	0.000477911
17033	0.01	20.979715	LRRC61	0.000476651
17034	0.01	24.042264	NTSR1	0.000415934
17035	0.01	65.28285	CYBA	0.00015318
17036	0.01	152.16728	FABP5	6.57171E-05
16952	0.01	4.9016171	AGTR1	0.002040143
16953	0.01	4.9278202	TCF15	0.002029295
16954	0.01	4.950298	WFDC2	0.00202008
16955	0.01	5.0828179	SLAIN1	0.001967413
16956	0.01	5.1868931	MNX1	0.001927936
16957	0.01	5.2391748	NOVA2	0.001908698
16958	0.01	5.3382302	HES7	0.00187328
16959	0.01	5.3518355	BEND4	0.001868518
16960	0.01	5.3746093	SNORD24	0.0018606
16961	0.01	5.3746093	SNORD28	0.0018606
16962	0.1501437	81.098323	PRAME	0.001851379
16963	0.01	5.4542588	3-Sep	0.00183343
16964	0.01	5.4569102	PRKCQ-AS1	0.001832539
16965	0.01	5.4588431	PRKCQ	0.00183189
16966	0.01	5.4777193	ANKRD18B	0.001825577
16967	0.01	5.577035	GATM	0.001793067
16968	0.01	5.6077142	B3GAT1	0.001783258
16969	0.01	5.621151	RSPO4	0.001778995
16970	0.01	5.7296211	LCE1F	0.001745316
16971	0.01	5.755547	SYN2	0.001737454
16972	0.01	5.8128909	NOVA1	0.001720314
16973	0.01	5.9824235	HID1	0.001671563
16974	0.01	6.0450615	SNCB	0.001654243
16975	0.01	6.078171	LRFN5	0.001645232
16976	0.01	6.1505212	ZDHHC22	0.001625878
16977	0.01	6.3197653	EMILIN3	0.001582337
16978	0.01	6.3983444	BEX5	0.001562904
16979	0.01	6.6081261	SNORA26	0.001513288
16980	0.01	6.6081261	MSI1	0.001513288
16981	0.01	6.6364125	WT1	0.001506838
16982	0.01	6.6596234	ZNF296	0.001501586
16983	0.01	6.7010793	CRABP1	0.001492297
16984	0.01	6.7182616	CDX2	0.00148848

16985	0.01	6.8395751	LRFN1	0.001462079
16986	0.01	6.8729519	LHX1	0.001454979
16987	0.01	6.9696251	TNFRSF10A	0.001434797
16988	0.01	7.0913401	FAM189A1	0.001410171
16989	0.01	7.2482058	KIF26A	0.001379652
16990	0.01	7.3686695	GATA4	0.001357097
16991	0.01	7.3902436	EMB	0.001353135
16992	0.01	7.5131288	PRR9	0.001331003
16993	0.01	7.5769867	SNORA33	0.001319786
16994	0.01	7.8470541	PPP2R2C	0.001274364
16995	0.01	7.8617955	GPR27	0.001271974
16996	0.01	7.9538039	ISM1	0.00125726
16997	0.01	7.9793894	SHANK1	0.001253229
16998	0.01	7.984021	NKD2	0.001252502
16999	0.01	8.0106458	OGDHL	0.001248339
17000	0.01	8.0974094	CERS4	0.001234963
17001	0.0548131	47.603884	IL10RA	0.001151442
17002	0.01	8.9430385	EGFL7	0.001118188
17003	0.01	8.9442068	APBB1IP	0.001118042
17004	0.01	9.2247974	MATK	0.001084035
17005	0.01	9.3443446	CITED1	0.001070166
17006	0.1178064	110.51521	CSF3	0.001065974
17007	0.01	9.4926638	ADD2	0.001053445
17008	0.01	9.848283	NAT8L	0.001015405
17009	0.01	9.9539327	SLC17A9	0.001004628
17010	0.01	10.227184	PLBD1	0.000977786
17011	0.01	10.25254	PRKCZ	0.000975368
17012	0.01	10.404031	CA2	0.000961166
17013	0.01	10.629298	RAB38	0.000940796
17014	0.0468735	52.540251	EPB41L3	0.000892144
17015	0.01	11.274021	CYTL1	0.000886995
17016	0.01	11.401198	ESX1	0.000877101
17017	0.0505211	57.654899	AEBP1	0.000876267
17018	0.01	12.52066	TUBB4A	0.00079868
17019	0.01	13.018451	GYLTL1B	0.000768141
17020	0.01	14.031299	COL9A3	0.000712692
17021	0.01	14.05952	DUSP9	0.000711262
17022	1.7024467	2450.8257	MMP1	0.000694642
17023	0.01	14.428231	MB21D1	0.000693086
17024	0.01	14.542347	TMEM88	0.000687647
17025	0.01	14.744588	MED12	0.000678215
17026	0.01	15.196923	ZFP57	0.000658028
17027	0.01	15.459905	KRT75	0.000646834

17028	0.01	16.490278	LCE1E	0.000606418
17029	0.01	17.315525	ABCA3	0.000577516
17030	0.01	18.085051	ERVMER34-1	0.000552943
17031	0.01	20.845524	MDFI	0.000479719
17032	0.01	20.924419	MST4	0.000477911
17033	0.01	20.979715	LRRC61	0.000476651
17034	0.01	24.042264	NTSR1	0.000415934
17035	0.01	65.28285	CYBA	0.00015318
17036	0.01	152.16728	FABP5	6.57171E-05
16952	0.01	4.9016171	AGTR1	0.002040143
16953	0.01	4.9278202	TCF15	0.002029295
16954	0.01	4.950298	WFDC2	0.00202008
16955	0.01	5.0828179	SLAIN1	0.001967413
16956	0.01	5.1868931	MNX1	0.001927936
16957	0.01	5.2391748	NOVA2	0.001908698
16958	0.01	5.3382302	HES7	0.00187328
16959	0.01	5.3518355	BEND4	0.001868518
16960	0.01	5.3746093	SNORD24	0.0018606
16961	0.01	5.3746093	SNORD28	0.0018606
16962	0.1501437	81.098323	PRAME	0.001851379
16963	0.01	5.4542588	3-Sep	0.00183343
16964	0.01	5.4569102	PRKCQ-AS1	0.001832539
16965	0.01	5.4588431	PRKCQ	0.00183189
16966	0.01	5.4777193	ANKRD18B	0.001825577
16967	0.01	5.577035	GATM	0.001793067
16968	0.01	5.6077142	B3GAT1	0.001783258
16969	0.01	5.621151	RSPO4	0.001778995
16970	0.01	5.7296211	LCE1F	0.001745316
16971	0.01	5.755547	SYN2	0.001737454
16972	0.01	5.8128909	NOVA1	0.001720314
16973	0.01	5.9824235	HID1	0.001671563
16974	0.01	6.0450615	SNCB	0.001654243
16975	0.01	6.078171	LRFN5	0.001645232
16976	0.01	6.1505212	ZDHHC22	0.001625878
16977	0.01	6.3197653	EMILIN3	0.001582337
16978	0.01	6.3983444	BEX5	0.001562904
16979	0.01	6.6081261	SNORA26	0.001513288
16980	0.01	6.6081261	MSI1	0.001513288
16981	0.01	6.6364125	WT1	0.001506838
16982	0.01	6.6596234	ZNF296	0.001501586
16983	0.01	6.7010793	CRABP1	0.001492297
16984	0.01	6.7182616	CDX2	0.00148848
16985	0.01	6.8395751	LRFN1	0.001462079

16986	0.01	6.8729519	LHX1	0.001454979
16987	0.01	6.9696251	TNFRSF10A	0.001434797
16988	0.01	7.0913401	FAM189A1	0.001410171
16989	0.01	7.2482058	KIF26A	0.001379652
16990	0.01	7.3686695	GATA4	0.001357097
16991	0.01	7.3902436	EMB	0.001353135
16992	0.01	7.5131288	PRR9	0.001331003
16993	0.01	7.5769867	SNORA33	0.001319786
16994	0.01	7.8470541	PPP2R2C	0.001274364
16995	0.01	7.8617955	GPR27	0.001271974
16996	0.01	7.9538039	ISM1	0.00125726
16997	0.01	7.9793894	SHANK1	0.001253229
16998	0.01	7.984021	NKD2	0.001252502
16999	0.01	8.0106458	OGDHL	0.001248339
17000	0.01	8.0974094	CERS4	0.001234963
17001	0.0548131	47.603884	IL10RA	0.001151442
17002	0.01	8.9430385	EGFL7	0.001118188
17003	0.01	8.9442068	APBB1IP	0.001118042
17004	0.01	9.2247974	MATK	0.001084035
17005	0.01	9.3443446	CITED1	0.001070166
17006	0.1178064	110.51521	CSF3	0.001065974
17007	0.01	9.4926638	ADD2	0.001053445
17008	0.01	9.848283	NAT8L	0.001015405
17009	0.01	9.9539327	SLC17A9	0.001004628
17010	0.01	10.227184	PLBD1	0.000977786
17011	0.01	10.25254	PRKCZ	0.000975368
17012	0.01	10.404031	CA2	0.000961166
17013	0.01	10.629298	RAB38	0.000940796
17014	0.0468735	52.540251	EPB41L3	0.000892144
17015	0.01	11.274021	CYTL1	0.000886995
17016	0.01	11.401198	ESX1	0.000877101
17017	0.0505211	57.654899	AEBP1	0.000876267
17018	0.01	12.52066	TUBB4A	0.00079868
17019	0.01	13.018451	GYLTL1B	0.000768141
17020	0.01	14.031299	COL9A3	0.000712692
17021	0.01	14.05952	DUSP9	0.000711262
17022	1.7024467	2450.8257	MMP1	0.000694642
17023	0.01	14.428231	MB21D1	0.000693086
17024	0.01	14.542347	TMEM88	0.000687647
17025	0.01	14.744588	MED12	0.000678215
17026	0.01	15.196923	ZFP57	0.000658028
17027	0.01	15.459905	KRT75	0.000646834
17028	0.01	16.490278	LCE1E	0.000606418

17029	0.01	17.315525	ABCA3	0.000577516
17030	0.01	18.085051	ERVMER34-1	0.000552943
17031	0.01	20.845524	MDFI	0.000479719
17032	0.01	20.924419	MST4	0.000477911
17033	0.01	20.979715	LRRC61	0.000476651
17034	0.01	24.042264	NTSR1	0.000415934
17035	0.01	65.28285	CYBA	0.00015318
17036	0.01	152.16728	FABP5	6.57171E-05
16952	0.01	4.9016171	AGTR1	0.002040143
16953	0.01	4.9278202	TCF15	0.002029295
16954	0.01	4.950298	WFDC2	0.00202008
16955	0.01	5.0828179	SLAIN1	0.001967413
16956	0.01	5.1868931	MNX1	0.001927936
16957	0.01	5.2391748	NOVA2	0.001908698
16958	0.01	5.3382302	HES7	0.00187328
16959	0.01	5.3518355	BEND4	0.001868518
16960	0.01	5.3746093	SNORD24	0.0018606
16961	0.01	5.3746093	SNORD28	0.0018606
16962	0.1501437	81.098323	PRAME	0.001851379
16963	0.01	5.4542588	3-Sep	0.00183343
16964	0.01	5.4569102	PRKCQ-AS1	0.001832539
16965	0.01	5.4588431	PRKCQ	0.00183189
16966	0.01	5.4777193	ANKRD18B	0.001825577
16967	0.01	5.577035	GATM	0.001793067
16968	0.01	5.6077142	B3GAT1	0.001783258
16969	0.01	5.621151	RSPO4	0.001778995
16970	0.01	5.7296211	LCE1F	0.001745316
16971	0.01	5.755547	SYN2	0.001737454
16972	0.01	5.8128909	NOVA1	0.001720314
16973	0.01	5.9824235	HID1	0.001671563
16974	0.01	6.0450615	SNCB	0.001654243
16975	0.01	6.078171	LRFN5	0.001645232
16976	0.01	6.1505212	ZDHHC22	0.001625878
16977	0.01	6.3197653	EMILIN3	0.001582337
16978	0.01	6.3983444	BEX5	0.001562904
16979	0.01	6.6081261	SNORA26	0.001513288
16980	0.01	6.6081261	MSI1	0.001513288
16981	0.01	6.6364125	WT1	0.001506838
16982	0.01	6.6596234	ZNF296	0.001501586
16983	0.01	6.7010793	CRABP1	0.001492297
16984	0.01	6.7182616	CDX2	0.00148848
16985	0.01	6.8395751	LRFN1	0.001462079
16986	0.01	6.8729519	LHX1	0.001454979

16987	0.01	6.9696251	TNFRSF10A	0.001434797
16988	0.01	7.0913401	FAM189A1	0.001410171
16989	0.01	7.2482058	KIF26A	0.001379652
16990	0.01	7.3686695	GATA4	0.001357097
16991	0.01	7.3902436	EMB	0.001353135
16992	0.01	7.5131288	PRR9	0.001331003
16993	0.01	7.5769867	SNORA33	0.001319786
16994	0.01	7.8470541	PPP2R2C	0.001274364
16995	0.01	7.8617955	GPR27	0.001271974
16996	0.01	7.9538039	ISM1	0.00125726
16997	0.01	7.9793894	SHANK1	0.001253229
16998	0.01	7.984021	NKD2	0.001252502
16999	0.01	8.0106458	OGDHL	0.001248339
17000	0.01	8.0974094	CERS4	0.001234963
17001	0.0548131	47.603884	IL10RA	0.001151442
17002	0.01	8.9430385	EGFL7	0.001118188
17003	0.01	8.9442068	APBB1IP	0.001118042
17004	0.01	9.2247974	MATK	0.001084035
17005	0.01	9.3443446	CITED1	0.001070166
17006	0.1178064	110.51521	CSF3	0.001065974
17007	0.01	9.4926638	ADD2	0.001053445
17008	0.01	9.848283	NAT8L	0.001015405
17009	0.01	9.9539327	SLC17A9	0.001004628
17010	0.01	10.227184	PLBD1	0.000977786
17011	0.01	10.25254	PRKCZ	0.000975368
17012	0.01	10.404031	CA2	0.000961166
17013	0.01	10.629298	RAB38	0.000940796
17014	0.0468735	52.540251	EPB41L3	0.000892144
17015	0.01	11.274021	CYTL1	0.000886995
17016	0.01	11.401198	ESX1	0.000877101
17017	0.0505211	57.654899	AEBP1	0.000876267
17018	0.01	12.52066	TUBB4A	0.00079868
17019	0.01	13.018451	GYLTL1B	0.000768141
17020	0.01	14.031299	COL9A3	0.000712692
17021	0.01	14.05952	DUSP9	0.000711262
17022	1.7024467	2450.8257	MMP1	0.000694642
17023	0.01	14.428231	MB21D1	0.000693086
17024	0.01	14.542347	TMEM88	0.000687647
17025	0.01	14.744588	MED12	0.000678215
17026	0.01	15.196923	ZFP57	0.000658028
17027	0.01	15.459905	KRT75	0.000646834
17028	0.01	16.490278	LCE1E	0.000606418
17029	0.01	17.315525	ABCA3	0.000577516

17030	0.01	18.085051	ERVMER34-1	0.000552943
17031	0.01	20.845524	MDFI	0.000479719
17032	0.01	20.924419	MST4	0.000477911
17033	0.01	20.979715	LRRC61	0.000476651
17034	0.01	24.042264	NTSR1	0.000415934
17035	0.01	65.28285	CYBA	0.00015318
17036	0.01	152.16728	FABP5	6.57171E-05
16952	0.01	4.9016171	AGTR1	0.002040143
16953	0.01	4.9278202	TCF15	0.002029295
16954	0.01	4.950298	WFDC2	0.00202008
16955	0.01	5.0828179	SLAIN1	0.001967413
16956	0.01	5.1868931	MNX1	0.001927936
16957	0.01	5.2391748	NOVA2	0.001908698
16958	0.01	5.3382302	HES7	0.00187328
16959	0.01	5.3518355	BEND4	0.001868518
16960	0.01	5.3746093	SNORD24	0.0018606
16961	0.01	5.3746093	SNORD28	0.0018606
16962	0.1501437	81.098323	PRAME	0.001851379
16963	0.01	5.4542588	3-Sep	0.00183343
16964	0.01	5.4569102	PRKCQ-AS1	0.001832539
16965	0.01	5.4588431	PRKCQ	0.00183189
16966	0.01	5.4777193	ANKRD18B	0.001825577
16967	0.01	5.577035	GATM	0.001793067
16968	0.01	5.6077142	B3GAT1	0.001783258
16969	0.01	5.621151	RSPO4	0.001778995
16970	0.01	5.7296211	LCE1F	0.001745316
16971	0.01	5.755547	SYN2	0.001737454
16972	0.01	5.8128909	NOVA1	0.001720314
16973	0.01	5.9824235	HID1	0.001671563
16974	0.01	6.0450615	SNCB	0.001654243
16975	0.01	6.078171	LRFN5	0.001645232
16976	0.01	6.1505212	ZDHHC22	0.001625878
16977	0.01	6.3197653	EMILIN3	0.001582337
16978	0.01	6.3983444	BEX5	0.001562904
16979	0.01	6.6081261	SNORA26	0.001513288
16980	0.01	6.6081261	MSI1	0.001513288
16981	0.01	6.6364125	WT1	0.001506838
16982	0.01	6.6596234	ZNF296	0.001501586
16983	0.01	6.7010793	CRABP1	0.001492297
16984	0.01	6.7182616	CDX2	0.00148848
16985	0.01	6.8395751	LRFN1	0.001462079
16986	0.01	6.8729519	LHX1	0.001454979
16987	0.01	6.9696251	TNFRSF10A	0.001434797

16988	0.01	7.0913401	FAM189A1	0.001410171
16989	0.01	7.2482058	KIF26A	0.001379652
16990	0.01	7.3686695	GATA4	0.001357097
16991	0.01	7.3902436	EMB	0.001353135
16992	0.01	7.5131288	PRR9	0.001331003
16993	0.01	7.5769867	SNORA33	0.001319786
16994	0.01	7.8470541	PPP2R2C	0.001274364
16995	0.01	7.8617955	GPR27	0.001271974
16996	0.01	7.9538039	ISM1	0.00125726
16997	0.01	7.9793894	SHANK1	0.001253229
16998	0.01	7.984021	NKD2	0.001252502
16999	0.01	8.0106458	OGDHL	0.001248339
17000	0.01	8.0974094	CERS4	0.001234963
17001	0.0548131	47.603884	IL10RA	0.001151442
17002	0.01	8.9430385	EGFL7	0.001118188
17003	0.01	8.9442068	APBB1IP	0.001118042
17004	0.01	9.2247974	MATK	0.001084035
17005	0.01	9.3443446	CITED1	0.001070166
17006	0.1178064	110.51521	CSF3	0.001065974
17007	0.01	9.4926638	ADD2	0.001053445
17008	0.01	9.848283	NAT8L	0.001015405
17009	0.01	9.9539327	SLC17A9	0.001004628
17010	0.01	10.227184	PLBD1	0.000977786
17011	0.01	10.25254	PRKCZ	0.000975368
17012	0.01	10.404031	CA2	0.000961166
17013	0.01	10.629298	RAB38	0.000940796
17014	0.0468735	52.540251	EPB41L3	0.000892144
17015	0.01	11.274021	CYTL1	0.000886995
17016	0.01	11.401198	ESX1	0.000877101
17017	0.0505211	57.654899	AEBP1	0.000876267
17018	0.01	12.52066	TUBB4A	0.00079868
17019	0.01	13.018451	GYLTL1B	0.000768141
17020	0.01	14.031299	COL9A3	0.000712692
17021	0.01	14.05952	DUSP9	0.000711262
17022	1.7024467	2450.8257	MMP1	0.000694642
17023	0.01	14.428231	MB21D1	0.000693086
17024	0.01	14.542347	TMEM88	0.000687647
17025	0.01	14.744588	MED12	0.000678215
17026	0.01	15.196923	ZFP57	0.000658028
17027	0.01	15.459905	KRT75	0.000646834
17028	0.01	16.490278	LCE1E	0.000606418
17029	0.01	17.315525	ABCA3	0.000577516
17030	0.01	18.085051	ERVMER34-1	0.000552943

17031	0.01	20.845524	MDFI	0.000479719
17032	0.01	20.924419	MST4	0.000477911
17033	0.01	20.979715	LRRC61	0.000476651
17034	0.01	24.042264	NTSR1	0.000415934
17035	0.01	65.28285	CYBA	0.00015318
17036	0.01	152.16728	FABP5	6.57171E-05
16952	0.01	4.9016171	AGTR1	0.002040143
16953	0.01	4.9278202	TCF15	0.002029295
16954	0.01	4.950298	WFDC2	0.00202008
16955	0.01	5.0828179	SLAIN1	0.001967413
16956	0.01	5.1868931	MNX1	0.001927936
16957	0.01	5.2391748	NOVA2	0.001908698
16958	0.01	5.3382302	HES7	0.00187328
16959	0.01	5.3518355	BEND4	0.001868518
16960	0.01	5.3746093	SNORD24	0.0018606
16961	0.01	5.3746093	SNORD28	0.0018606
16962	0.1501437	81.098323	PRAME	0.001851379
16963	0.01	5.4542588	3-Sep	0.00183343
16964	0.01	5.4569102	PRKCQ-AS1	0.001832539
16965	0.01	5.4588431	PRKCQ	0.00183189
16966	0.01	5.4777193	ANKRD18B	0.001825577
16967	0.01	5.577035	GATM	0.001793067
16968	0.01	5.6077142	B3GAT1	0.001783258
16969	0.01	5.621151	RSPO4	0.001778995
16970	0.01	5.7296211	LCE1F	0.001745316
16971	0.01	5.755547	SYN2	0.001737454
16972	0.01	5.8128909	NOVA1	0.001720314
16973	0.01	5.9824235	HID1	0.001671563
16974	0.01	6.0450615	SNCB	0.001654243
16975	0.01	6.078171	LRFN5	0.001645232
16976	0.01	6.1505212	ZDHHC22	0.001625878
16977	0.01	6.3197653	EMILIN3	0.001582337
16978	0.01	6.3983444	BEX5	0.001562904
16979	0.01	6.6081261	SNORA26	0.001513288
16980	0.01	6.6081261	MSI1	0.001513288
16981	0.01	6.6364125	WT1	0.001506838
16982	0.01	6.6596234	ZNF296	0.001501586
16983	0.01	6.7010793	CRABP1	0.001492297
16984	0.01	6.7182616	CDX2	0.00148848
16985	0.01	6.8395751	LRFN1	0.001462079
16986	0.01	6.8729519	LHX1	0.001454979
16987	0.01	6.9696251	TNFRSF10A	0.001434797
16988	0.01	7.0913401	FAM189A1	0.001410171

16989	0.01	7.2482058	KIF26A	0.001379652
16990	0.01	7.3686695	GATA4	0.001357097
16991	0.01	7.3902436	EMB	0.001353135
16992	0.01	7.5131288	PRR9	0.001331003
16993	0.01	7.5769867	SNORA33	0.001319786
16994	0.01	7.8470541	PPP2R2C	0.001274364
16995	0.01	7.8617955	GPR27	0.001271974
16996	0.01	7.9538039	ISM1	0.00125726
16997	0.01	7.9793894	SHANK1	0.001253229
16998	0.01	7.984021	NKD2	0.001252502
16999	0.01	8.0106458	OGDHL	0.001248339
17000	0.01	8.0974094	CERS4	0.001234963
17001	0.0548131	47.603884	IL10RA	0.001151442
17002	0.01	8.9430385	EGFL7	0.001118188
17003	0.01	8.9442068	APBB1IP	0.001118042
17004	0.01	9.2247974	MATK	0.001084035
17005	0.01	9.3443446	CITED1	0.001070166
17006	0.1178064	110.51521	CSF3	0.001065974
17007	0.01	9.4926638	ADD2	0.001053445
17008	0.01	9.848283	NAT8L	0.001015405
17009	0.01	9.9539327	SLC17A9	0.001004628
17010	0.01	10.227184	PLBD1	0.000977786
17011	0.01	10.25254	PRKCZ	0.000975368
17012	0.01	10.404031	CA2	0.000961166
17013	0.01	10.629298	RAB38	0.000940796
17014	0.0468735	52.540251	EPB41L3	0.000892144
17015	0.01	11.274021	CYTL1	0.000886995
17016	0.01	11.401198	ESX1	0.000877101
17017	0.0505211	57.654899	AEBP1	0.000876267
17018	0.01	12.52066	TUBB4A	0.00079868
17019	0.01	13.018451	GYLTL1B	0.000768141
17020	0.01	14.031299	COL9A3	0.000712692
17021	0.01	14.05952	DUSP9	0.000711262
17022	1.7024467	2450.8257	MMP1	0.000694642
17023	0.01	14.428231	MB21D1	0.000693086
17024	0.01	14.542347	TMEM88	0.000687647
17025	0.01	14.744588	MED12	0.000678215
17026	0.01	15.196923	ZFP57	0.000658028
17027	0.01	15.459905	KRT75	0.000646834
17028	0.01	16.490278	LCE1E	0.000606418
17029	0.01	17.315525	ABCA3	0.000577516
17030	0.01	18.085051	ERVMER34-1	0.000552943
17031	0.01	20.845524	MDFI	0.000479719

17032	0.01	20.924419	MST4	0.000477911
17033	0.01	20.979715	LRRC61	0.000476651
17034	0.01	24.042264	NTSR1	0.000415934
17035	0.01	65.28285	CYBA	0.00015318
17036	0.01	152.16728	FABP5	6.57171E-05
