# Supplementary figures and images for: The miR-34a-5p promotes the multi-chemoresistance of osteosarcoma via repression of the AGTR1 gene
Source: BMC Cancer. 2017 Jan 10;17:45. doi: 10.1186/s12885-016-3002-x (PMC5223322; doi:10.1186/s12885-016-3002-x)

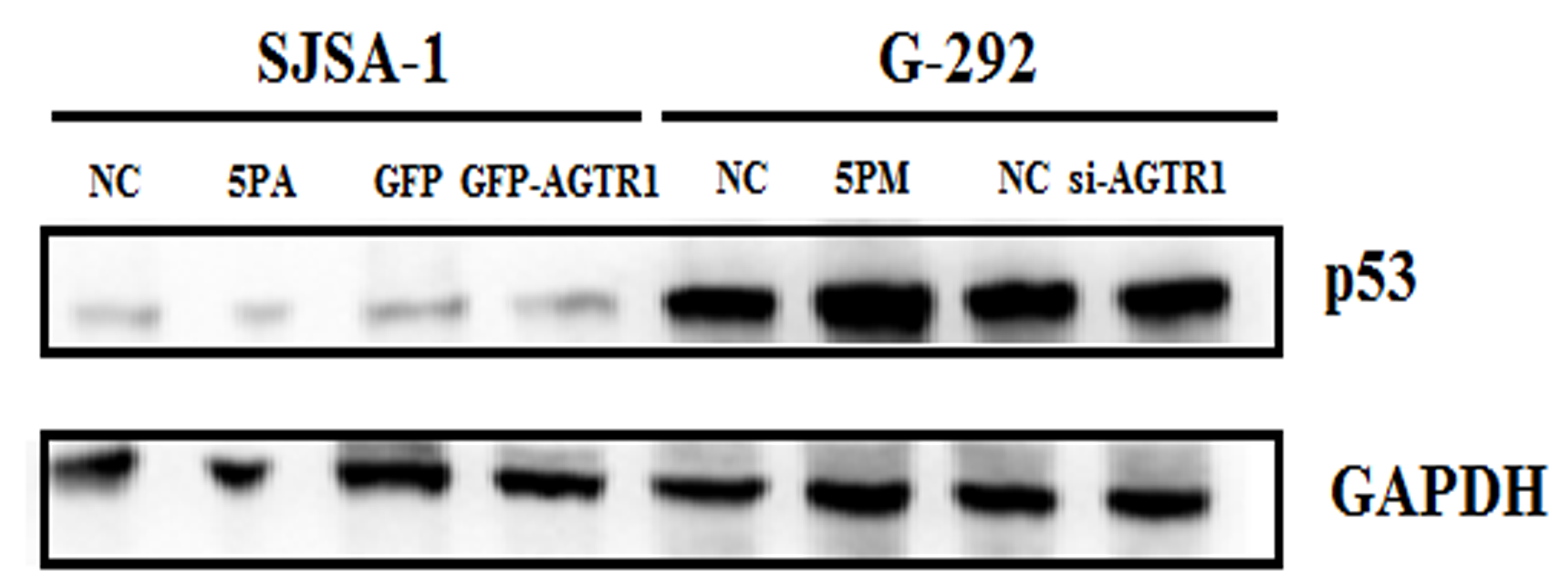

Supplement: Additional file 4: Figure S4. — The protein level of p53 detected by western in NC, 5PA, GFP, GFP-AGTR1 transfected SJSA-1 cells, and the NC, 5PM, NC, si-AGTR1 transfected G-292 cells. (TIF 17.8 mb) [file 12885_2016_3002_MOESM4_ESM.tif]
